# The oldest case of paedomorphosis in rove beetles and description of a new genus of Paederinae from Cretaceous amber (Coleoptera: Staphylinidae)

**DOI:** 10.1038/s41598-023-32446-2

**Published:** 2023-03-31

**Authors:** Alexandra Tokareva, Katarzyna Koszela, Vinicius S. Ferreira, Shûhei Yamamoto, Dagmara Żyła

**Affiliations:** 1 Museum and Institute of Zoology, Polish Academy of Sciences, Wilcza 64, 00-679 Warsaw, Poland; 2 Natural History Museum of Denmark, Universitetsparken 15, 2100 Copenhagen, Denmark; 3 The Hokkaido University Museum, Hokkaido University, Kita 10, Nishi 8, Kita-Ku, Sapporo, Hokkaido 060-0810 Japan; 4 Leibniz Institute for the Analysis of Biodiversity Change, Martin-Luther-King-Platz 3, 20146 Hamburg, Germany

**Keywords:** Computational biology and bioinformatics, Evolution, Genetics, Zoology

## Abstract

The ecology of extinct species from the Cretaceous is largely unknown. Morphological features of specimens preserved in amber can help to reveal habitats and evolutionary strategies that occurred in fossil lineages. An unusually small rove beetle (Staphylinidae) from the subfamily Paederinae with a Y-shaped suture on the head and modified tarsi and antennae is newly described here as *Midinudon juvenis* Tokareva & Żyła gen. et sp. nov. We hypothesise that such a combination of characters represents the earliest example of paedomorphosis in Staphylinidae and discuss other possible reasons that could explain the small size and morphological modifications of the new species. We provide the results of total-evidence phylogenetic analysis and discuss the relationships of *Midinudon juvenis* Tokareva & Żyła gen. et sp. nov. within Paederinae.

## Introduction

Paedomorphosis, or paedomorphic syndrome, is a term that describes different heterochronic processes which result in adults displaying features resembling those of their immatures^1–3^. Currently, there is no common view on the mechanisms of such heterochrony, and understanding of the term “paedomorphosis” may vary in works of different authors. In this paper, we define paedomorphosis not as evolutionary process, but as a descriptive term that describes a certain appearance of the descendant morphology, according to the view of McNamara^2^. The syndrome is known to occur in various groups of animals, including mammals, birds, and several invertebrates^2,4^. In insects, the paedomorphic syndrome has been observed in many lineages, including moths (Lepidoptera), aphids (Hemiptera), wasps (Hymenoptera), and beetles (Coleoptera)^5^. In some groups affected by paedomorphosis, adults may display reduced sclerotisation, as well as miniaturisation and simplification of morphological features^5,6^.

In beetles, paedomorphosis has been observed in many groups, although with a higher predominance in the Elateroidea, a lineage that comprises click-beetles (Elateridae), fireflies (Lampyridae), and net-winged beetles (Lycidae)^3,7^. In such groups, the syndrome can be expressed as a mosaic of features of adult and immature stages, including reduced sclerotisation, wing reduction or complete absence, miniaturisation of morphological structures, the predominance of the K-reproductive strategy, and increased female fecundity^3,6,7^. Occasionally, paedomorphic female beetles can be fully larviform^6^. Despite the remarkable biological modifications associated with this syndrome, little is known about the processes leading to the repeated and independent development of paedomorphosis in Coleoptera and insects in general.

Rove beetles (Staphylinidae) are no exception, and very little is known about the paedomorphic syndrome in the group. Currently, the number of formally described species in this family is 66,444^8^, which makes them the most species rich animal family on Earth as known by today^9^. Although some myrmeco- and termitophilous Staphylinidae display features that could be associated with the paedomorphic syndrome^10^, little has been added to the debate on this phenomenon in the family. Crowson^10^ pointed out the similarity in the head morphology of adults and larvae of some Staphylinidae, the dorsal defensive gland of many Aleocharinae larvae and the pygidial glands of adults of the family, as well as the physogastry of some Aleocharinae, also resulting in wing and elytral reductions^10–13^. Certain works studying rove beetles that can be considered paedomorphic [e.g. ^5,11,14^] tend to omit the current debate focusing on paedomorphosis in favour of other processes that could also explain the modified morphology of these beetles, such as parasitism and convergent evolution, and the study of paedomorphosis in Staphylinidae remains largely unexplored.

Among Staphylinidae, the subfamily Paederinae is quite remarkable, being one of the most speciose in the group with more than 7400 species in ca. 230 genera, and worldwide distribution^8,15,16^. Despite the size of the subfamily, little is known about their internal phylogenetic relationships and evolutionary history, although some research has recently been done. To date, five phylogenies for Paederinae, all confirming their monophyly, have been published^16–21^, but all of these studies included a limited set of ingroup species (60 terminal taxa at maximum) focusing only on certain lineages. Three of them were total-evidence analyses including both morphological and molecular data. The taxonomic impediment in Paederinae regularly prevents the data from numerous unrevised or poorly-defined genera and species from being used in many routine analyses that would push forward the understanding of the subfamily^22^.

At the same time, this subfamily is quite abundant in the fossil record, which makes it possible to include data on extinct taxa in phylogenetic analyses and shed some light on the evolution of the group^16,18^. Overall, 41 species of fossil Paederinae, representing 15 genera, have been described to date, where 24 species are known from rock imprints and 17 as amber inclusions (Supplementary Table 1; Catalogue of Life Checklist^8^; https://fossilinsectdatabase.co.uk by Mitchell^23^), with the oldest specimen known from Early Cretaceous (Yixian Formation) in the genus *Mesostaphylinus* Zhang, 1989^24^. At the same time, a large number of fossils remain undescribed [^18,25^, D. Żyła, pers. obs.], while many existing descriptions are incomplete and probably require revision^16,25^.

Although the majority of Paederinae fossils are found as rock imprints from the Cenozoic^25^, the most informative specimens are often found in amber [e.g. ^16,18,22^]. Precise outlines of cavities, which preserve the beetle’s surface, and sometimes internal structures are a great source of characters to be compared with those of extant fauna. By now, 13 species from Cenozoic ambers (Baltic, Rovno, Mexican) and three species from mid-Cretaceous (Burmese) amber are described in Paederinae.

Recently, a new pair of amber Paederinae specimens from the mid-Cretaceous Kachin (Burmese) amber from northern Myanmar was brought to our attention. These beetles seem to belong to an extinct group, closely related to one of the previously described genera from Burmese amber, *Diminudon* Żyła, Yamamoto & Jenkins Shaw, 2019, but unexpectedly also exhibit some features that can be associated with paedomorphosis syndrome.

In our study, we describe a new genus and species and test its phylogenetic position within Paederinae. We also discuss possible scenarios that could lead to the unprecedented set of morphological characters, including paedomorphic ones, and propose explanations for this phenomenon, such as paleoenvironmental factors, independent miniaturisation of the lineage, inquilinism, or their combinations as evolutionary drivers to explain it.

## Results

### Phylogenetic analyses

**Figure 1:**
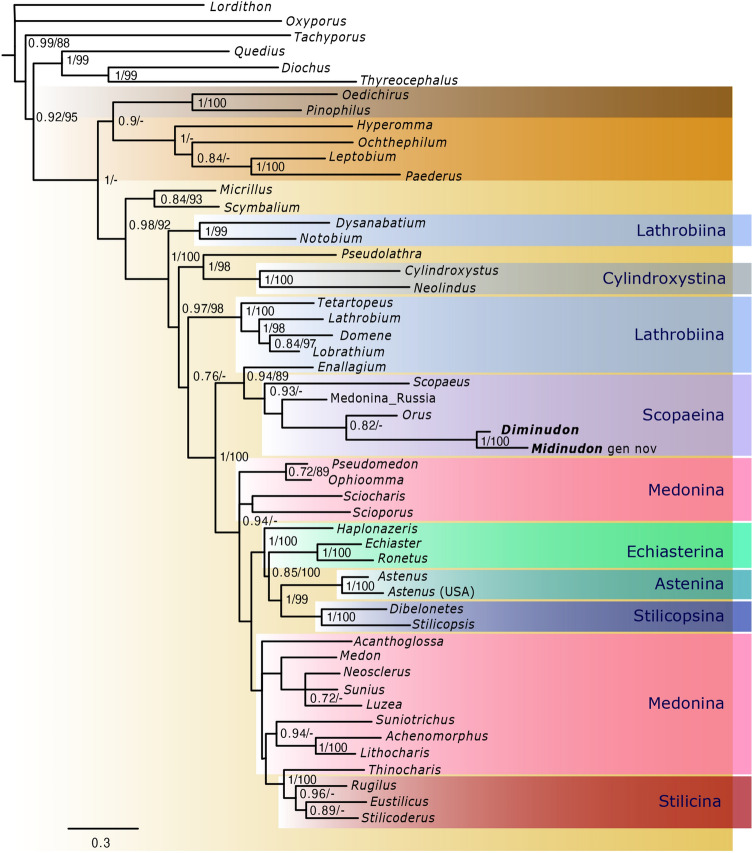
A 50% majority-rule consensus tree from a Bayesian Inference analysis of combined molecular and morphological datasets. Posterior probabilities (PP) and ultrafast bootstrap supports (UFB) values are shown at the corresponding nodes in PP/UFB format. Tribes and subtribes of Paederinae highlighted in colour. Extinct genera names written in bold.

PartitionFinder found the following four partitions: (1) 28S, CADC2, TP2, CADA1, Wg1, Wg2, ArgK2, COI1, CADC3, CADA2, TP3, COI2, ArgK3; (2) Wg3, ArgK1, TP1; (3) CADA3, CADC1, and 4) COI3. For both methods, Bayesian Inference and Maximum Likelihood, GTR + I + G was selected as the best-supported model for the first and third partitions. The differences were in the case of the second partition, where GTR + I + G model was found as best for MrBayes, while TVM + I + G for IQTREE. For the fourth partition, the HKY + G model was found to be the best supported. However, this partition was excluded as it has been shown that the 3rd codon position of COI can potentially bias phylogenetic analyses [e.g. 26, 27]. All independent Markov chains converged on the same stationary distribution as visualised in Tracer, and both combined and individual traces were inspected. The effective sample size (ESS) values were greater than 200 for all parameters indicating good mixing of the chains. The tree topology presented in Fig. 1 is the 50% majority-rule consensus tree of BI analysis with support values of both BI and ML. The ML tree is available as Supplementary Data 1.

As in previous phylogenetic reconstructions^16–21^, the subfamily Paederinae was recovered as monophyletic and well supported (PP = 0.92, UFB = 95). All currently recognised tribes of Paederinae, i.e. Pinophilini, Paederini, and Lathrobiini (PP = 1, UFB = 100; PP = 1, UFB = 100; PP = 0.98, UFB = 92, respectively) were also resolved as separated clades. Resolving the position of Pinophilini still requires more data as BI analysis recovered it as a sister group to Paederini (PP = 0.90) while ML analysis showed it as sister to Lathrobiini, but with no support.

Within the tribe Lathrobiini, several subtribes were resolved as non-monophyletic. Two genera which are currently included in Lathrobiini *incertae sedis*^18^, *Micrillus* Raffray, 1873 and *Scymbalium* Erichson, 1839, were the first clade that branched off (PP = 0.84, UFB = 93) as a sister group to all other Lathrobiini (PP = 0.98, UFB = 92). The second clade to branch off was also well supported (PP = 1, UFB = 99) and consisted of *Dysanabatium* Bernhauer, 1915 and *Notobium* Solsky, 1864, currently classified in Lathrobiina and sister to the rest of Lathrobiini (PP = 1, UFB = 100). Next, in both analyses, the genus *Pseudolathra* Casey, 1905 (Lathrobiini *incertae sedis* after Żyła et al.^19^) and the subtribe Cylindroxystina were resolved within the tribe Lathrobiini as sister to each other (PP = 1, UFB = 98) and together as sister to the rest of Lathrobiini (PP = 0.97, UFB = 98). ‘True’ Lathrobiina (clade from *Tetartopeus* to *Lobrathium*, PP = 1, UFB = 100), which comprised four taxa, were recovered as the next clade, sister to the rest of the analysed subtribes with weak (PP = 0.76) or no support.

Within the ‘Medonina and allied taxa’ clade (sensu Żyła et al.^16^), the first clade to branch off was well-supported (PP = 0.94, UFB = 89) and consisted of six taxa, including the new genus *Midinudon* Tokareva & Żyła gen. nov*.* which was resolved as sister to *Diminudon* (PP = 1, UFB = 100) within well-supported Scopaeina (PP = 0.93). As in the most recently published phylogenies^19–21^, Scopaeina was resolved as sister to *Enallagium* Bernhauer, 1915 (Lathrobiina). In the BI tree, the extinct taxa were recovered as sister to the Scopaeina genus *Orus* Casey, 1885 (PP = 0.82), then together to an unidentified genus of ‘Medonina’ from Far East Russia and all of them sister to *Scopaeus* Erichson, 1840. In this part of the tree, the ML analysis resulted in a different topology (Supplem. Data 1), and the fossil clade was resolved as sister directly to *Scopaeus* and then to *Orus* with no and weak support (UFB = 86), respectively. In the ML analysis, the subtribe Scopaeina was recovered as sister to the unidentified genus of Medonina (UFB = 89). The subtribe Medonina was not resolved as monophyletic and its members were recovered in a few positions on the tree. The genera *Pseudomedon* Mulsant & Rey, 1878 and *Ophioomma* Notman, 1920 were resolved as a separate clade (P = 0.72, UFB = 89). In the BI tree, the position of *Sciocharis* Lynch, 1885 and *Scioporus* Sharp, 1886 was unsolved, while in the ML analysis, both taxa were resolved in isolated positions, the first as sister to *Pseudomedon* + *Ophioomma* clade and the remaining Lathrobiini (UFB = 96), and the second sister to the remaining Lathrobiini (UFB = 71). The genus *Haplonazeris*, currently listed in Echiasterina^8^, was resolved as sister to the other two taxa of Echiasterina, Astenina and Stilicopsina with strong support (PP = 1, UFB = 100). The BI analysis recovered the rest of Medonina mainly with poorly resolved topology and not well supported. In the BI analysis, the position of *Achenomorphus* Motschulsky, 1858 remained unsolved, while ML analysis recovered it inside the clade containing two other representatives of Medonina, but with no support. The rest of the taxa belonging to this subtribe formed a separate clade (*Medon* Stephens, 1833, *Neosclerus* Cameron, 1924, *Sunius* Curtis, 1829, *Luzea* Blackwelder, 1952) or remained unresolved (*Acanthoglossa* Kraatz, 1859). *Thinocharis* Kraatz, 1859, which is formally included in Medonina, in both analyses was resolved as sister to monophyletic Stilicina with strong support (PP = 1, UFB = 100).

### Systematic Palaeontology

Order Coleoptera Linnaeus, 1758.

Suborder Polyphaga Emery, 1886.

Family Staphylinidae Latreille, 1802.

Subfamily Paederinae Fleming, 1821.

Tribe Lathrobiini Laporte, 1835.

Genus *Midinudon* Tokareva & Żyła gen. nov.

**LSID.** ZooBank ID http://zoobank.org/urn:lsid:zoobank.org:act:423D73EC-7199-4710-9E40-7E0167E90CDC.

### Etymology

The name is derived from *Diminudon*, a previously described amber fossil genus, which shares many common traits with the new one, and the prefix “midi-”, which specifies that specimens of the new genus are of middle size, being slightly larger than *Diminudon* but on average smaller than extant Scopaeina*.* Gender masculine.

### Type species

*Midinudon juvenis* Tokareva & Żyła sp. nov., here designated.

### Diagnosis

**Figure 2:**
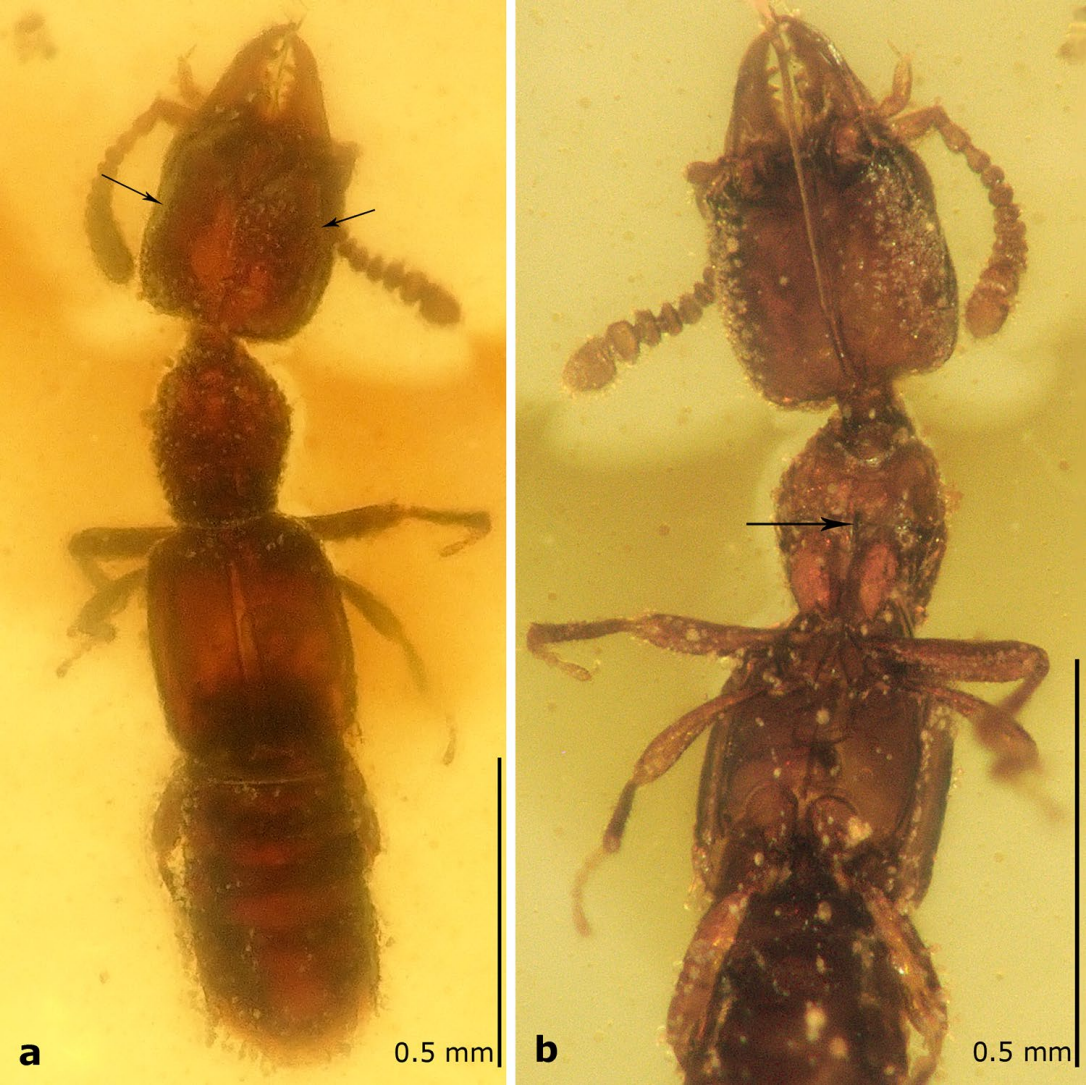
*Midinudon juvenis* gen. and sp. nov., holotype, collection number GPIH05515. (a) Photo of habitus, dorsal view. Ridges separating the flat area above and behind the eyes are marked with arrows. (b) Photo of head and thorax, ventral view. Longitudinal ridge of basisternum marked with an arrow.

The new genus and species can be distinguished from all extant Paederinae by the following combination of characters: body small (< 2 mm); antenna with discoidal transverse antennomeres; area of head behind antenna flattened (Fig. 2a); Y-shaped suture present on frons; tarsal formula 4–4-4.

Compared to the closely related extinct genus *Diminudon*, the body of *Midinudon* is slightly larger; posterior angles of head straighter; Y-shaped sutures present on frons; maxillary palpomere 3 of regular form, not fusiform; neck narrower, equal to or less than 1/4 of head width; punctation of head and pronotum less evident; pronotum with posterior edge slightly elevated, pronotal angles obtuse.

## Description

As in *Midinudon juvenis* Tokareva & Żyła sp. nov. type species description.

*Midinudon juvenis* sp. nov.

**Figure 3:**
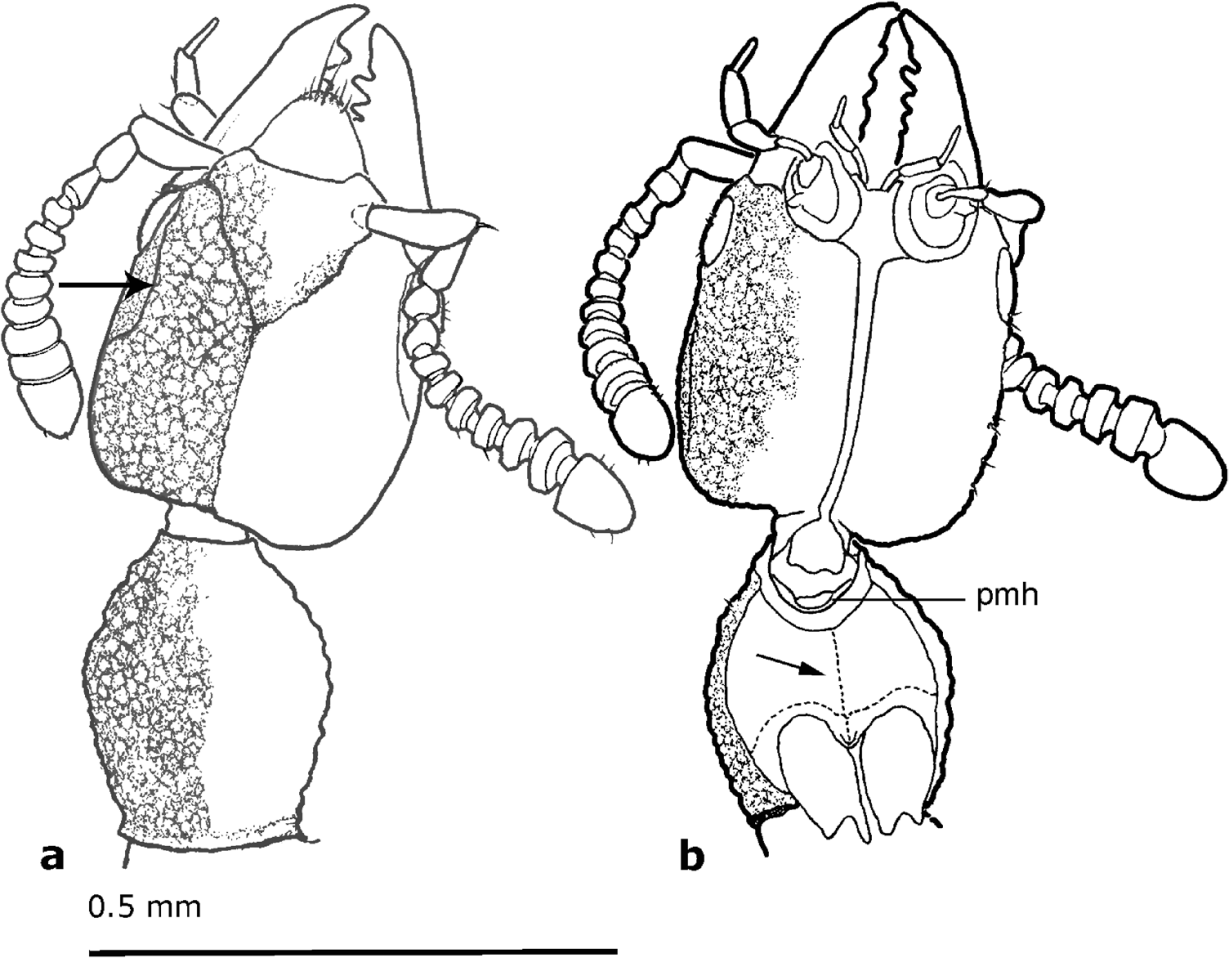
Schematic drawings of *Midinudon juvenis* gen. and sp. nov. based on holotype, collection number GPIH05515 specimen. Head and prothorax. (a) Dorsal view; ridge separating the flat area above and behind the eye marked with an arrow. (b) Ventral view; longitudinal ridge of basisternum marked with an arrow. Pmh – posterior margin of head.

**Figure 4:**
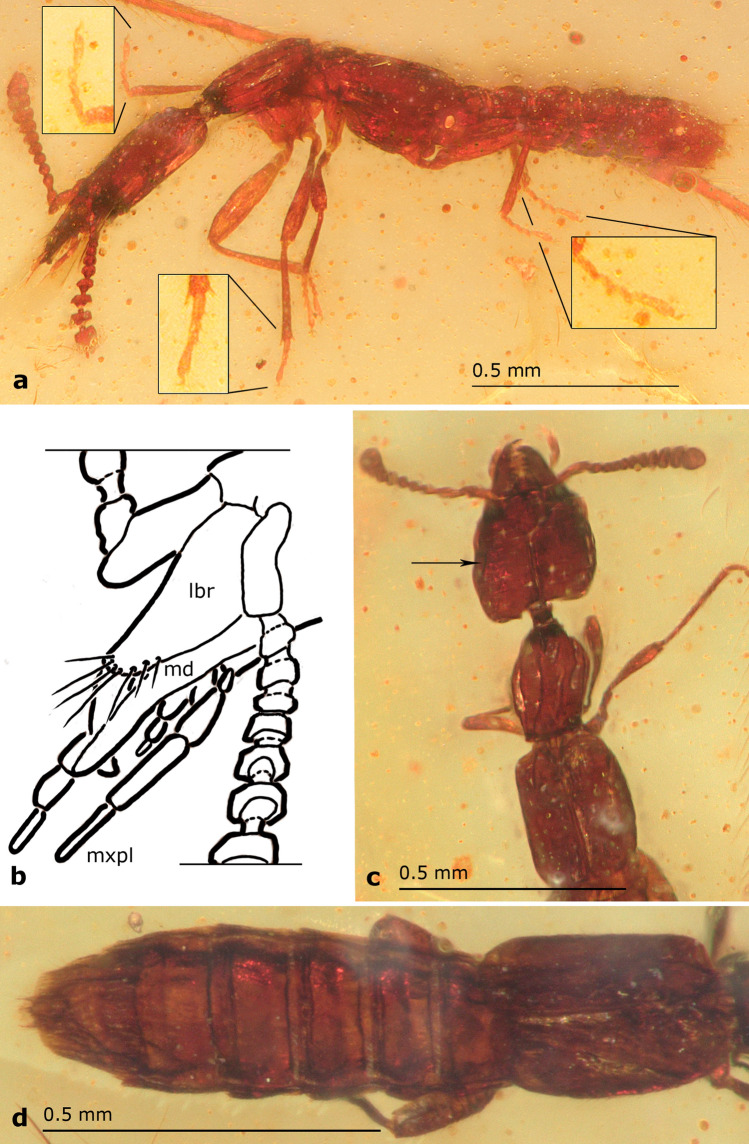
*Midinudon juvenis* gen. and sp. nov., paratype, collection number GPIH05516. (a) Photo of habitus, lateral view. Close-ups of tarsi in frames. (b) Drawing of head and mouthparts: lbr – labrum, md – mandibula, mxlp – maxillary palpus. (c) Photo of forebody, dorsal view. Ridge separating flat area above and behind eye marked with an arrow. (d) Photo of meso-, metathorax, and abdomen, dorsal view.

**Figure 5:**
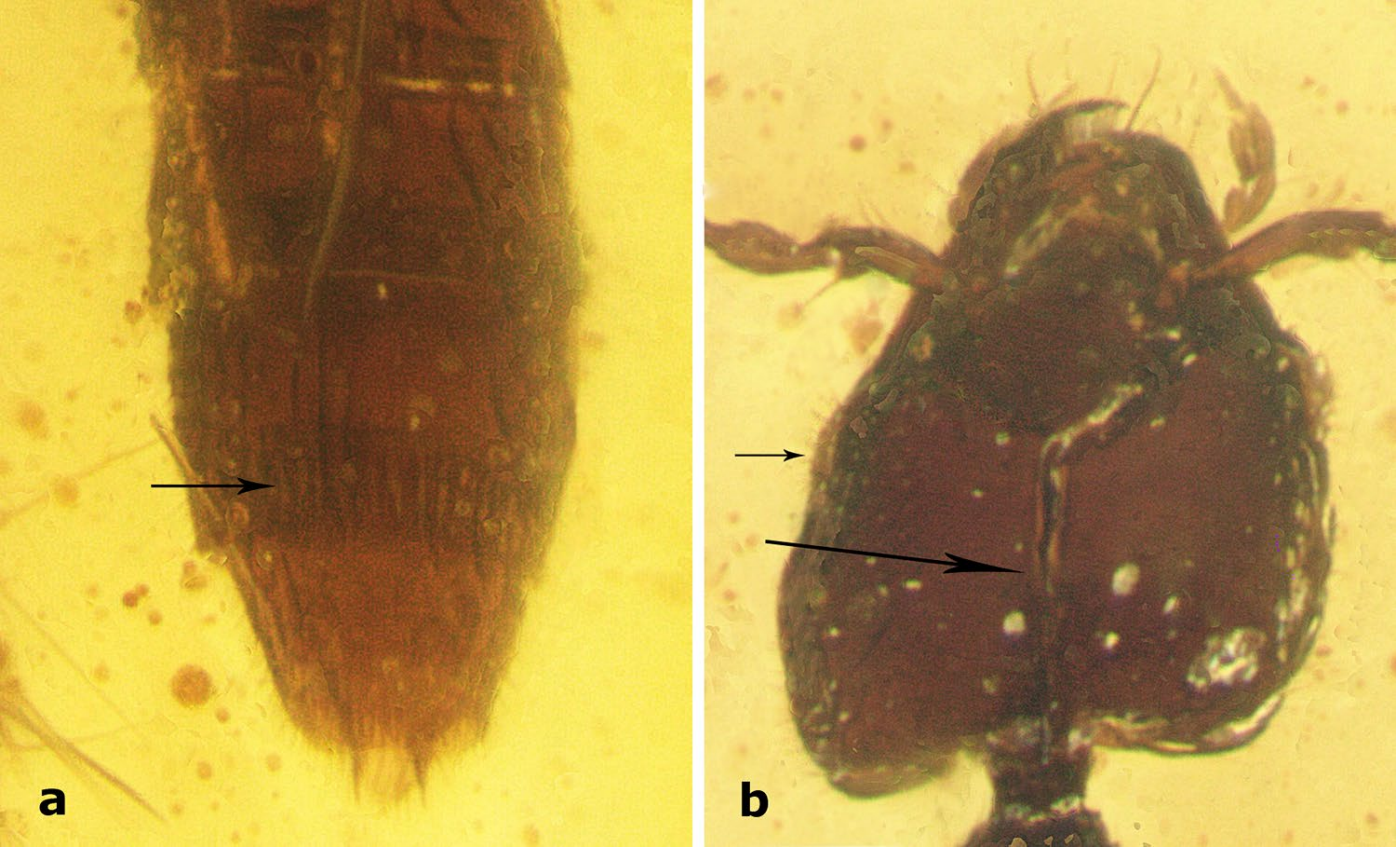
*Midinudon juvenis* gen. and sp. nov., photos of paratype, collection number. GPIH05516. (a) Apex of abdomen, ventral view, line of setae on sternite VII marked with an arrow. (b) Head, dorsal view, large arrow: stem of Y-shaped suture, small arrow: setae between ommatidia.

Figures 2, 3, 4 and 5.

**LSID.** ZooBank ID http://zoobank.org/urn:lsid:zoobank.org:act:B8B61199-E97F-4A3F-A6FC-3A2D2F99C989.

**Etymology.** The specific epithet refers to some remarkable characters of the species that looks “larval", or juvenile, to the authors, in particular, the Y-shaped head dorsal sutures.

**Examined material.** *Holotype*: sex unknown, GPIH05515, Leibniz Institute for the Analysis of Biodiversity Change, collected in Hukawng Valley, Kachin, Myanmar. *Paratype*: sex unknown, GPIH05516, Leibniz Institute for the Analysis of Biodiversity Change, collected in Hukawng Valley, Kachin, Myanmar.

**Locality and horizon.** Kachin amber from the Hukawng Valley, Kachin State, northern Myanmar; unnamed horizon, mid-Cretaceous (near Albian–Cenomanian boundary).

**Diagnosis**. As for the genus.

**Description.** Very small (BL: 1.73); body narrow with slender legs and large head. Punctation visible on head and pronotum of paratype but scarce on holotype. Colouration light brown.

*Head*. Subquadrate (HL: 0.30–0.36; HW: 0.31–0.32), dorso-ventrally flattened, widest just anterior to posterior angles, wider than pronotum; temples straight, slightly rounded at posterior angles; posterior margin straight or with angles slightly protruding posteriorly; vertex area flattened dorso-posteriorly to eye, separated from dorsal side by longitudinal ridge (arrows in Fig. 2a,3a,4c), frons rugose, with Y-shaped occipital and frontal sutures (Fig. 2a,3a,4c,5b), integument slightly wrinkled. Eye small (0.09), not protruding, almost flat, shorter than 1/5 of head length, 1/3 of temple length, with setae between ommatidia (Fig. 5b). Antennae inserted between two frontal sutures relatively close to each other, with distance between bases longer than distance from base to anterior margin of eye. Antenna (Fig. 2a,b,3a,b,4a–c) longer than temples, slightly moniliform, 11-segmented with at least apical antennomeres with tomentose pubescence; antennomeres 4–11 wider than long. Antennomere 1 elongate, widest at middle, almost as long as antennomeres 2–4 combined; antennomere 2 elongate, with base forming long stem, widest at tip (Fig. 2a,b); antennomere 3 slightly longer than wide, with base forming long stem, widest at tip; antennomeres 4–6 almost discoidal; antennomeres 7–10 transverse, with bases forming visible stems; antennomere 11 longer than wide, twice longer than 10, rectangular, tapering towards apex, radially symmetrical. Clypeal margin straight. Labrum entire, large, longer than wide, with width tapering to apex, not covering mandibles when closed, with group of setae on anterior margin (Fig. 3a,4b,5b). Mandibles stout, symmetrical, protruding anterad, with four relatively small teeth. Maxillary palpus 4-segmented; palpomere 1 short; palpomere 2 longer than wide, slightly bent in middle, widened towards apex; palpomere 3 as long and as wide as 2, cylindrical; palpomere 4 almost as long as 3, fusiform and narrow, 1/3 of palpomere 3 width (Fig. 2b,3a,b,4a,b,5b). Labial palpus 3-segmented (Fig. 2b,3b,4b); palpomere 1 short; palpomere 2 elongated, slightly expanded towards apex; palpomere 3 thin, acicular, almost as long as palpomere 2. Gular sutures narrowly separated (Fig. 2b,3b), not reaching posterior margin of head. Neck narrow, less than 1/4 of head width.

*Thorax*. Pronotum longer than wide (PL: 0.29–0.31; PW: 0.21), narrowed in front, widest anterior to middle part, with anterior angles obtuse; disc punctured. Postcoxal process well-developed, slightly curved under pronotal disc (Fig. 2b,3b,4a). Basisternum of prosternum with longitudinal carina (Fig. 2b,3b). Furcasternum of prosternum longer than 1/2 of basisternum length, with sharp longitudinal carina. Mesoventrite with longitudinal carina, reaching more than 1/3 of distance between coxae. Elytra rectangular, longer and wider than pronotum (EL: 0.39–0.41; EW: 0.31–0.32), without epipleural ridge; humeral angle distinct, rounded; surface shiny, covered with short setae. Scutellum with two transverse ridges. Procoxae large, conical. Protibia narrow. All tarsi 4-segmented (Fig. 4a). Protarsus with tarsomeres 1–3 not inflated, equal to meso- and metatarsomeres, without adhesive setae on ventral side. Protarsomere 1 longer than 2, protarsomere 3 equal to 2, not bilobed, protarsomere 4 longest, equal to 1–2 combined. Mesotarsus with mesotarsomere 1 longer than 2, mesotarsomere 3 longer than 2, not bilobed, mesotarsomere 4 longer than 1. Metatarsus with metatarsomere 1 longer than 2, length of metatarsomere 2 equal to 3, metatarsomere 3 not bilobed, metatarsomere 4 longer than 2–3 combined. Hind wings present, entirely developed.

*Abdomen*. Slightly widened posteriorly. Tergites III–VII with two pairs of paratergites (Fig. 4d) on each side. Sternite III with keel between coxae. Posterior part of sternite VII with regular line of middle-length setae (Fig. 5a). Tergite VIII with posterior margin rounded. Anterior margin of sternite VIII without distinct emargination.

## Discussion

### Conditions leading to size reduction and morphological modifications

Because of the uncommon combination of morphological features found in *Midinudon juvenis* Tokareva & Żyła gen. et sp. nov., we hypothesise that the species has been affected by several evolutionary processes resulting in size reduction as well as the display of paedomorphic characters in the head, which are presented below.

The main difference in morphology of *Midinudon juvenis* Tokareva & Żyła gen. et sp. nov. from all other known extinct and extant Paederinae is the presence of a distinct Y-shaped suture on the dorsal plate of the head in both specimens (Figs. 2a,3a,4c,5b). Careful observations of different angles allowed us to conclude that the suture is invaginated and easily distinguishable, and unlikely to be an artefact. The homology of the Y-shaped suture on *Midinudon*’s head is unknown. There are limited data on similar structures in adult Staphylinidae, although a groove that looks like such a structure is has been described in *Homalolinus* Sharp, 1885 (Xantholininae)^28^ and a similar suture can be seen in *Cephalochetus* Kraatz, 1859 (Paederinae, AT and DŻ personal observations). Within rove beetles, head sutures reminiscent of the discussed one can be seen in species of *Glypholoma* Jeannel, 1962 (Glypholomatinae)^29^ and in some lineages of Tachyporinae^30^. However, these structures seem to have different anatomy than Y-shaped suture in *Midinudon juvenis* Tokareva & Żyła gen. et sp. nov. At the same time, the vast majority of Staphylinidae larvae have Y-shaped ecdysial sutures (e.g. Figures 12, 13 in Staniec et al.^31^; Figs. 15, 24 in Grebennikov & Newton^32^; Figs. 7, 28, 46 in Tokareva et al.^33^), with antennae inserted within the triangular frontal part, as in *Midinudon juvenis* Tokareva & Żyła gen. et sp. nov. However, without proper histological and embryological experiments, it would be difficult to confidently consider these Y-shaped structures of different genera as homologous. Although the “suture” is reminiscent of the ecdysial lines of larvae, the latter are functional splitting points of the head capsule during molting, so preservation of this structure in the adult state seems debatable or perhaps irrelevant. Also, none of the other traits typical for life forms that are called paedomorphic (soft abdomen or wing reduction) are present: both specimens of *Midinudon juvenis* Tokareva & Żyła gen. et sp. nov. have fully developed hind wings and a well-sclerotised abdomen (Figs. 2–5). Still, due to the limited knowledge of the origin of paedomorphic features in Staphylinidae, we believe that the presence of a larval-looking character in the extinct genus needs to be reported and discussed.

### Palaeoenvironmental effect

A literature search shows numerous notes on some Burmese amber insects being much smaller than their extant relatives, not only in Coleoptera but also other orders, such as Hymenoptera, Thysanoptera, and Hemiptera^16,34–41^. As the trend is present among different orders, it seems likely to be a result of palaeoenvironmental phenomena, such as a different climate than in recent times^37^. Li and colleagues^37^ mentioned that average minimum mean equatorial temperatures in the mid-Cretaceous were higher than today, while oxygen levels were lower. According to Bergmann’s rule, the higher the temperature, the smaller the animal can be. This concept is usually applied to mammals, while for insects the mechanisms may differ, or there is no correlation^42^. At the same time, the authors suggested that the sizes of insects could expand with time simply due to random variation during the evolution of a group. These two ideas do not seem controversial and could work in parallel.

On the other hand, it is also known that not all Paederinae described so far from the Cretaceous are distinctly smaller than extant representatives. For example, *Cretoprocirrus trichotos* Jenkins Shaw & Żyła, 2020^22^, a ~ 99 million year old beetle from the tribe Pinophilini, has a body length (13 mm) similar to an average extant species of *Procirrus* and related lineages^43,44^.

### Miniaturisation

If not an environmental effect, could it be miniaturisation within the lineage, rather than a general process affecting the whole fauna? On the one hand, such phenomena can be connected with the reduction of metamere exoskeleton elements, a shorter number of antennomeres or tarsomeres, whole head capsules without sutures, and other modifications. On the other hand, radical miniaturisation of certain species or genera leads to profound changes in morphology due to different influences of usual physical processes and forces on a microscopic level^45^. From entirely different wing morphology and action to changes in the number, size, and ultrastructure of ganglia neurons, reduction of heart, and other peculiar morphological modifications, miniaturisation can involve taxa of different sizes^45,46^.

Which factors could be drivers of miniaturisation in the Cretaceous is an open question. Although bringing certain limitations, miniaturisation provides opportunities to conquer new ecological niches, adopt special diets, avoid predators, or even disperse more effectively across the globe^47,48^. Sometimes insects, in particular beetles, become minute while getting adapted to a peculiar habitat, like pore tubes of bracket fungi^49^, or nests of other organisms, e.g. social insects^47,50^.

At the same time, it should be kept in mind that possibly not miniaturisation but an increase of body size could have been an evolutionary trend for some groups—in this case, minute size in extinct species would be plesiomorphic for such lineages. In the case of morphological modifications, especially complex ones, it is difficult to imagine that the fusion of all head sutures could be a plesiomorphic state, especially if the sutures in extant taxa are separated.

As mentioned before, *Midinudon juvenis* Tokareva & Żyła gen. et sp. nov. and *Diminudon* (both known species) are quite small beetles compared to extant Scopaeina and all other Paederinae, also having a reduced tarsal formula, which has not been reported from extant Paederinae. Aside from this, there are no other signs of reduction of simplification or fusion of metamere structures in these beetles. Potentially, the evolution of a new life strategy, like inquilinism of social insect colonies, or a change of habitat to one with a higher density than leaf litter (like in soil mesofauna) could be the factors that led to these reductions^47^.

### Inquilinism

Although inquilinism does not always lead to miniaturisation, there can be a certain correlation between these two phenomena. For example, some morphological changes, including paedomorphic ones^14,52^, which can be seen in inquilines, can also result from miniaturisation [^3^ and references therein]. Thus, decreased number of antennomeres, simplified tarsi, fused abdominal segments, and relatively small size are characteristic for some obligate myrmeco- or termitophile lineages of rove beetles, e.g. the subfamily Pselaphinae (Clavigeritae)^51^. At the same time, the reduction of metamere organs can happen without any significant decrease in linear sizes, e.g. in some inquiline thrips (Thysanoptera), linear sizes can be larger than in free-living representatives^34^. Nevertheless, for ant inquilines, sizes are often comparable to or smaller than those of their host species^52^.

The most notable inquiline-containing Staphylinidae group is Aleocharinae. In such tribes as Trilobitideini, Phyllodinardini, Termitopaediini, and Corotocini, morphological adaptations for their lifestyle are significant^53^, and only some of them, such as compact legs and antennae, may be comparable to similar changes during miniaturisation. Other modifications seem to be specific to the strategy and levels of integration into the host colony. Such genera as *Baeostethus* Broun, 1909, *Neodioxeuta* Seevers, 1957, *Corotoca* Schiødte, 1853, *Dilacera* Zilberman & Pires-Silva, 2022 can also represent certain characters typical for larvae, e.g. weakly sclerotised abdomen, underdeveloped genitalia, and wing reduction, which some authors^3,5,7,14^ call paedomorphic features (Fig. 1: I, K, L in Orlov et al.^53^).

Not only have social insects like ants and termites experienced an increase in diversity in the Late Cretaceous^54,55^, but also inquilines from different groups, like Aleocharinae rove beetles^56,57^, Histeridae beetles^58,59^, cockroaches^60^, and some Collembola^61^. A histerid beetle of the extinct genus *Promyrmister* Zhou, Ślipiński & Parker, 2019 is among the earliest examples of myrmecophily and demonstrates morphological modifications typical for inquiline members of the family already ~ 99 Mya^58^, as well as *Amplectister terapoides* Yamamoto & Caterino, 2022 also from Kachin amber^59^. The potentially termitophilous mid-Cretaceous aleocharine genera *Mesosymbion* Yamamoto, Maruyama & Parker, 2016 and *Cretotrichopsenius* Cai, Huang, Newton, Eldredge & Engel, 2017 have a highly specialised habitus that resembles extant termitophilous Aleocharinae^56,57^. These findings provide some evidence that inquilinism could have occurred in other groups of Staphylinidae at that time.

Some features of the subtribe Scopaeina, where the new species was placed by our phylogenetic analysis, may also be seen as characteristic for inquilines. Although their life strategies are generally poorly known, we could expect inquilinism in this group, especially since Paederinae is the third subfamily in terms of number of inquiline genera^21^. A small flattened body with a telescopic, typical staphylinid abdomen is a good body groundplan for a social insect nest-dweller. Our extinct genera both have extremely flattened bodies of tiny size, as well as antennae of a certain form: moniliform, cylindrical, with antennomeres 6–11 transverse, discoidal, widening to the apex (Figs. 2a,b,3a,b,4a,c). Additionally, *Midinudon* Tokareva & Żyła gen. nov. has a longitudinal ridge above each antenna (Figs. 2a,b,3a,b,4c), which separates a slightly flat or excavated area from the dorsal part of the head. Interestingly, many species of staphylinids known to be inquilines have such moniliform antennae with discoidal terminal antennomeres. For example, species of *Claviger* Preyssler, 1790 (Pselaphinae) and many species of inquiline Paederinae, like *Ecitonides tuberculosus* Wasmann, 1894, *Ecitosaurus lujae* Wasmann, 1925, *Monista darlingtoni* Blackwelder, 1943 or the recently described termite inquiline *Ruptor cordatus* Żyła et al., 2022 have similar antennal structure and folding area to *Midinudon juvenis* Tokareva & Żyła gen. et sp. nov.^21^. Aleocharine inquilines usually have short, easy-to-fold cylindrical antennae, e.g. *Phyllodinarda xenocephala* Wasmann, 1916, *Baeostethus chiltoni* Broun, 1909, *Dinarda dentata* (Gravenhorst, 1806), and other species^52^. Since size reduction and morphological modification can be driven by inquiline adaptations, hypothesising such a life style for *Midinudon juvenis* Tokareva & Żyła gen. et sp. nov. seems plausible. Żyła et al.^16^ presented the idea that Late Cretaceous *Diminudon*, minute beetles with flattened body and transverse antennae, could have lived under bark, like some other rove beetles, e.g. certain genera of Osoriinae, Xantholininae, and Piestinae^62^. This hypothesis suggests that adaptations for dwelling in narrow spaces can be common for deadwood habitats as well as for living in termites’ galleries.

### Phylogenetic placement of *Midinudon* Tokareva & Żyła gen. nov

The specimens of the new genus described here share a wide range of common characters with the extinct genus *Diminudon*, which together makes them distinct from any other known Paederinae. All of the specimens are comparatively small, gracile, distinctly punctured, with mandibles protruding anterad, pronotum longer than wide and narrower than the head, slightly moniliform antennae with a transverse distal part, which have a certain aleocharine-like appearance due to distinct stems connecting the almost discoidal bodies of antennomeres, and reduced number of tarsomeres. At the same time, there are noticeable differences between the species of the genus *Diminudon* and the new genus: slightly larger body size, a Y-shaped frontal-occipital suture, and a more obtuse pronotum with a neck-like anterior part.

The results of the phylogenetic analysis which places *Midinudon* Tokareva & Żyła gen. nov. and *Diminudon* within Scopaeina seem reasonable, as this Paederinae subtribe is known to include beetles with small size. Moreover, such characters as narrow neck, obtuse pronotum, dorso-ventrally flattened body, triangle head, and all tibiae of the same width are relatively common in the representatives of the subtribe. Nevertheless, the extinct genera are smaller than most extant Scopaeina (< 2 mm versus 2–4 mm, acc. to Frisch et al.^63^) and have some features not typical for the lineage, nor Paederinae as a whole.

Along with their size, they have a reduced tarsal formula compared to extant Scopaeina and other Paederinae, i.e. 4–4-4 vs 5–5-5. If we take into account the modified but fully developed antennae and Y-shaped dorsal head suture, such tarsi could be a sign of some other hypothetical evolutionary “syndrome” connected to size reduction.

### Future directions

Our studies show that Paederinae rove beetles were already highly specialised at the early stage of their evolution. We present hypotheses that could explain peculiar morphology of the new genus and species and discuss possible evolutionary scenarios. One of the main obstacles to reaching final conclusions is almost complete lack of knowledge on how morphological changes depend on evolutionary trends of miniaturisation or inquiline adaptation, especially since body size may decrease with any of these processes. Detailed studies would require not only experiments in physiology and embryology, but also more basic data on taxonomy, life cycles, and morphology of all stages of target organisms. Paederinae, and Staphylinidae as a whole, remains a largely understudied group, especially in terms of their biology and immature stages. Further research on evolutionary trends in this diverse and worldwide distributed beetle group, especially in a palaeoenvironmental context, would be a great contribution and a necessary step forward to a better understanding of the processes that drive morphological modifications.

## Material and methods

### Examination and deposition of taxa

Both amber specimens are deposited in the palaeontological collection of the Leibniz Institute for the Analysis of Biodiversity Change and are accessible for further research under accession codes GPIH05515 and GPIH05516.

Habitus photos of each specimen were taken with the Keyence VHX-7000 photosystem at the Museum and Institute of Zoology, Polish Academy of Sciences, while detailed images were taken with a Leica M205 microscope system and the Leica Application Suite (LAS Version 4.7) software in the Laboratory of Evolutionary Entomology and Museum of Amber Inclusions, University of Gdańsk, Poland.

Drawings of the head and pronotum were prepared with the digitisation of pencil sketches made during the examination of the specimens via camera lucida attached to an Olympus SZH10 stereo microscope. Measurements of specimens and structures were taken with the Keyence VHX-7000 photosystem or an ocular micrometre attached to the Olympus SZH10. Most examinations were made with an Olympus SZX7 stereo microscope. Photos and drawings were processed and arranged in Adobe Photoshop CS5.5, while the phylogenetic tree scheme was colourised with Adobe Illustrator CS5.5 (Adobe Systems Inc., San Jose, California, USA, 2007). Original photos of the specimens are available in the Zenodo repository via the link: https://doi.org/10.5281/zenodo.7270297.for measurements are as follows: BL—body length, taken from level of closed mandibles edge to posterior edge of visible apical abdominal sclerite; HL—head length, taken from level of posterior angles to anterior margin of the frons; HW—maximum head width, including eyes; PL—pronotum length; PW—maximum pronotum width; EL—elytra length, taken from level of humeri to posterior edge from dorsal view; EW—maximum elytra width. For the newly described genus and species, measurements were taken from both specimens. All measurements are given in millimetres (mm).

### Taxon sampling and outgroup for phylogenetic analysis

All three currently recognised tribes of Paederinae were represented in the combined morphological and molecular analysis. A reasonable proportion in the number of taxa of particular subtribes was maintained. In total, 52 taxa were included in the final combined dataset, including six outgroups. The sampling largely overlaps with those used in the recent publications by our research group^16,19–21^. Three new taxa were newly sequenced, two genera of Medonina (*Sciocharis* and *Scioporus*) and one of Echiasterina (*Haplonazeris* Coiffait & Sáiz, 1968). For most representatives, both molecular and morphological data were available. The exceptions were five extant taxa for which no DNA-grade specimens were available and only morphological characters were included in the matrix, i.e. three representatives of Medonina (*Acanthoglossa*, *Luzea*, and *Ophioomma*), and two genera classified as Lathrobiini *incertae sedis* (*Micrillus* and *Scymbalium*). Since one of the main goals of our studies was testing the phylogenetic position of the new genus *Midinudon* Tokareva & Żyła gen. nov., morphological data for the probably closely related extinct *Diminudon* were added. Representatives of the subfamily Staphylininae were chosen as the closest related outgroup, and taxa from Mycetoporinae, Oxyporinae and Tachyporinae as more distant outgroups.

### Morphological characters

The morphological matrix was constructed in Mesquite v3.5^64^ using 121 characters, which were primarily derived from the matrix of Żyła et al.^19^.

Unknown character states were coded using ‘?’, and inapplicable states as ‘–’. The list of characters is provided in Supplementary Data 2. The nexus file containing the complete character matrix is available as Supplementary Data 3 in PDF and in MorphoBank (project no 4412) under this permalink: http://morphobank.org/permalink/?P4412.

### GenBank data

The molecular matrix was constructed using seven gene fragments: the nuclear protein-encoding genes carbamoylphosphate synthetase (CADA and CADC), topoisomerase I (TP), arginine kinase (ArgK), and wingless (Wg), the mitochondrial protein-encoding cytochrome c oxidase I (COI), and the nuclear ribosomal 28S. The Genbank accession numbers of all sequences are given in Supplementary Data 4. Almost all sequences were already used in Żyła et al.^19^; thus, the amplification, sequencing, sequence editing and assembly protocols are described there and the same protocols were applied to the newly sequenced specimens.

### DNA extraction, amplification, and sequence alignment

Whole genomic DNA was extracted non-destructively from the abdomens of beetle specimens.

Following the extraction, the resulting physical voucher included the non-extracted portion of the specimen frozen in 96% ethanol and the extracted part, also frozen in 96% ethanol, in a separate vial. All vouchers of the extracted specimens are deposited in the original collections. The sequences were obtained following a previously used protocol for extraction and amplification (see Żyła et al.^16^ for the details). All extracts were stored in a – 20 °C freezer, to avoid the fast fragmentation of DNA.

All sequences were aligned in Geneious v9.1.7 (Biomatters Ltd, Auckland, New Zealand, 2005) using the MAFFT plugin v1.3.6, based on MAFFT^65^. Sequences of 28S were aligned using the E-INS-i algorithm of MAFFT. In addition, the server version of Gblocks^66^ was used for the identification and removal of its ambiguously aligned regions. The gap positions within the final blocks and less strict flanking positions were allowed but many contiguous non-conserved positions were blocked. The final resulting 28S alignment was 847 bp and had very few scattered and usually single nucleotide gaps. Individual gene alignments were concatenated with the ‘concatenate’ function of Geneious. The concatenated sequence alignment is provided in Supplementary Data 5 in PDF format.

### Data matrix and partitioning

Bayesian inference (BI) and maximum likelihood (ML) methods were used to analyse the combined matrix of molecular (4831 bp) and morphological (121 characters) data for all of the studied taxa (52 OTUs). For the molecular data matrix, the alignment was initially partitioned by gene and, for protein-encoding genes, by codon position. The optimal partitioning scheme and the corresponding models of nucleotide evolution were determined by PartitionFinder v2.1.1^67^ using the Bayesian Information Criterion. Models for MrBayes and IQ-TREE were considered, and in the first case, selected models only for MrBayes, in the second, all models were taken into account. In addition, branch lengths were unlinked, and the search was set to the ‘greedy’ algorithm^68^. The morphological dataset in the combined matrix was analysed as a single, separate partition using the maximum likelihood model for discrete morphological character data, under the assumption that only characters that varied among taxa were included (Mkv)^69^.

### Phylogenetic analysis

Bayesian analysis was performed using MrBayes v3.2.6^70^ running on CIPRES^71^. The analysis used four chains (one cold and three heated) and two runs of 8 million generations with default prior settings. An exception was made for the temperature, which was set to ‘temp = 0.08’ for better mixing. In the case of the morphological partition, the analysis was conducted with a gamma distribution and autapomorphic characters were included. As in previous studies [e.g.^19^], the third COI codon positions were excluded. A script for the combined analysis in MrBayes is given in Supplementary Data 6 and is also available on GitHub under the following link https://github.com/DagmaraZyla/Midinudon. The convergence of both runs was assessed in Tracer v1.7.1^72^, as well as by the examination of Potential Scale Reduction Factor (PSRF) values and Average Standard Deviation of Split Frequencies in the MrBayes output.

Maximum likelihood (ML) analysis was performed using IQ-TREE v2.0.7^73^ with the same set of partitions. Node support was evaluated by 1000 ultrafast bootstrap replicates (UFB) ^74^ (command line: iqtree2 -p scheme.nex -B 1000 -nt AUTO -o Lordithon).

Both trees were examined in FigTree v1.4.4 (http://tree.bio.ed.ac.uk/software/figtree/) and later edited and annotated in Adobe Illustrator CS5.5 (Adobe Systems, San Jose, California, USA). Clade support was estimated by BI posterior probability values (PP) and ultrafast bootstrap approximation (UFB) in ML. Nodes with PP > 0.80 and UFB > 95 were considered well supported, nodes with PP: 0.70–0.79 and UFB: 80–94 were considered to be weakly supported and nodes with PP < 0.70 and UFB < 80 were considered unsupported.

## Supplementary Information

Supplementary Information 1.

Supplementary Information 2.

Supplementary Information 3.

Supplementary Information 4.

Supplementary Information 5.

Supplementary Information 6.

Supplementary Information 7.

Supplementary Information 8.

## Data Availability

The datasets generated and/or analysed during the current study are available in this article, its Supplementary Files, and, alternatively, in the following repositories: Zenodo, https://doi.org/10.5281/zenodo.7270297; MorphoBank, http://morphobank.org/permalink/?P4412; Github, https://github.com/DagmaraZyla/Midinudon.
