# Supplementary material for: The oldest case of paedomorphosis in rove beetles and description of a new genus of Paederinae from Cretaceous amber (Coleoptera: Staphylinidae)
Source: Sci Rep. 2023 Mar 31;13:5317. doi: 10.1038/s41598-023-32446-2 (PMC10066364; doi:10.1038/s41598-023-32446-2)
Supplement: Supplementary file 1 — Supplementary Information 1. [file 41598_2023_32446_MOESM1_ESM.docx]

**File Name**: Supplementary Table 1.
**Description**: List of fossil Paederinae in .xlsx format.

**File Name**: Supplementary Data 1.
**Description**: Maximum Likelihood tree in .pdf format. Ultrafast bootstrap supports (UFB) values are shown at the corresponding nodes.

**File Name**: Supplementary Data 2.
**Description**: The list of characters for the morphological matrix in .docx format.

**File Name**: Supplementary Data 3.
**Description**: The file containing the complete morphological characters matrix in .pdf format.

**File Name**: Supplementary Data 4.
**Description**: The Genbank accession numbers as a table in .docx format.

**File Name**: Supplementary Data 5.
**Description**: The concatenated alignment of all sequences in .pdf format.

**File Name**: Supplementary Data 6.
**Description**: A script for the combined analysis in MrBayes in .pdf format.
