# Supplementary figures and images for: The oldest case of paedomorphosis in rove beetles and description of a new genus of Paederinae from Cretaceous amber (Coleoptera: Staphylinidae)
Source: Sci Rep. 2023 Mar 31;13:5317. doi: 10.1038/s41598-023-32446-2 (PMC10066364; doi:10.1038/s41598-023-32446-2)

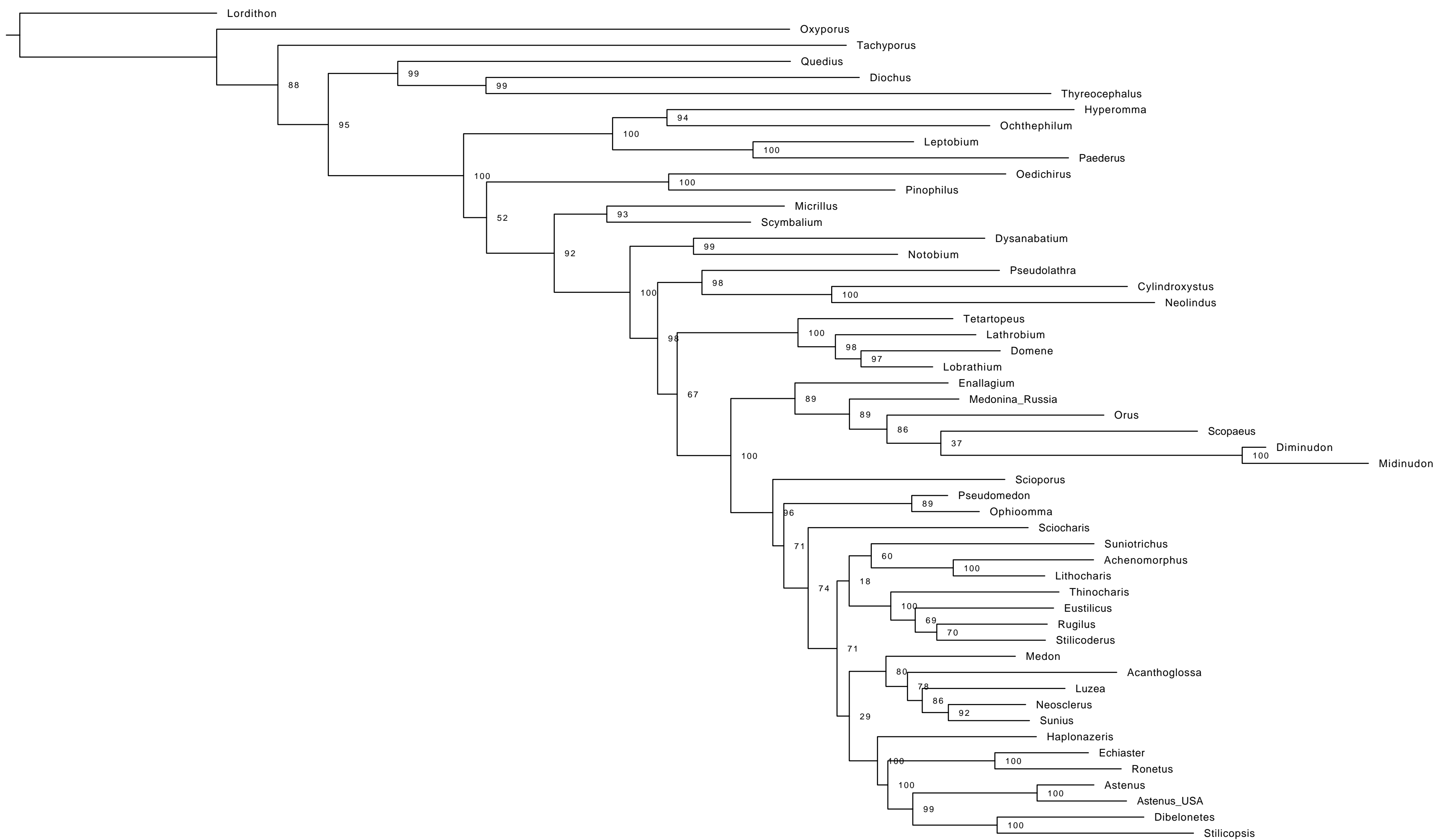

0.2

Supplement: Supplementary file 3 — Supplementary Information 3. [file 41598_2023_32446_MOESM3_ESM.pdf]
