# Supplementary material for: The oldest case of paedomorphosis in rove beetles and description of a new genus of Paederinae from Cretaceous amber (Coleoptera: Staphylinidae)
Source: Sci Rep. 2023 Mar 31;13:5317. doi: 10.1038/s41598-023-32446-2 (PMC10066364; doi:10.1038/s41598-023-32446-2)
Supplement: Supplementary file 4 — Supplementary Information 4. [file 41598_2023_32446_MOESM4_ESM.docx]

1. Antennae, form

0 non-geniculate

1 geniculate

2. Antennae, base of antennomere 1

0 concealed to different extent thus not fully visible from above

1 fully exposed and visible from above

3. Head, 'shelf' concealing antennal bases, presence

0 absent

1 present

4. Antennae, antennomere 3, tomentose pubescence, presence

0 absent

1 present

5. Antennae, antennomere 4, tomentose pubescence, presence

0 absent

1 present

6. Antennae, antennomeres 9 and 10, each of them, shape

0 elongated, filiform, thin

1 elongated, egg or funnel shaped

2 wider than long

7. Antennae, antennomere 11, shape

0 radially symmetrical

1 radially asymmetrical

8. Antennae, setation

0 without long setae extending perpendicular to the antennal axis

1 with long setae extending perpendicular to the antennal axis

9. Antennae, distance between bases

0 longer than distance from base of antenna to anterior margin of eye

1 equal to or smaller than distance from base of antenna to anterior margin of eye

10. Eyes, setae between ommatidia, presence

0 absent

1 present

11. Head, Y-shaped longitudinal grooves on dorsal surface between eyes, presence

0 absent

1 present

12. Head, labrum, shape

0 quadrate/rectangular, width less than twice the length

1 transverse, width equal to twice the length or broader

13. Head, labrum, size

0 not expanded, at least half of mandibles visible when closed

1 expanded, almost or completely covering mandibles when closed

14. Head, labrum, development

0 entire

1 notched to different extent

2 dentate

15. Head, labrum, depth of emargination

0 shallow, basally not reaching membrane connecting labrum with frons (frontoclypeus)

1 deep, basally extended through membrane connecting labrum with frons (frontoclypeus)

16. Head, labrum, apical membrane, presence

0 absent

1 present

17. Head, labrum, attachment to frons (frontoclypeus)

0 with distinct membrane between frons and base of labrum

1 without distinct membrane between frons and base of labrum

18. Head, maxillary palpomere 3, shape

0 elongated, regular

1 expanded, more fusiform

2 slightly expanded, vase-like

19. Head, maxillary palpomere 4, shape

0 fusiform

1 conical

2 nipple-like, elongate

3 small, acicular

4 truncate, short

5 securiform

20. Head, maxillary palpomere 4, width

0 narrower than palpomere 3

1 as wide or almost as wide as palpomere 3

2 wider than palpomere 3

21. Head, maxillary palpomeres 3 and 4, length

0 palpomere 3 equal to or shorter than 4

1 palpomere 3 longer than 4

22. Head, maxillary palpomere 4, setation

0 glabrous or at most sparsely setose

1 densely setose

23. Head, labial palpomeres 2 and 3, length

0 palpomere 2 equal to or longer than 3

1 palpomere 2 shorter than 3

24. Head, ligula, development

0 bilobed

1 entire

2 reduced

3 trilobed

25. Head, ligula, dorsal plate, setation

0 absent

1 present

26. Labial palpomere 3, width

0 same as palpomere 2

1 thiner than palpomere 2

2 wider than palpomere 2

27. Head, mandibules, projection

0 strongly projected anteriad

1 bent laterally

28. Head, mandibules, dorso-lateral groove, presence

0 absent

1 present

29. Head, mandibular prostheca, presence

0 present

1 absent

30. Head, mandibles, teeth, presence

0 present

1 absent

31. Head, mandibles, shape

0 stout (at least twice as wide at base as width at apical portion)

1 thin (as wide at base as apical portion or slightly wider)

32. Head, mandibles, left vs right

0 mandibles symmetrical (but size of same teeth may slightly differ on left and right mandible)

1 mandibles asymmetrical (when size, shape or sometimes number of teeth clearly differ on left and right madible)

33. Head, frons (i.e. the area of the head between eyes), punctation

0 more sparse than on the rest of head

1 as on the rest of head

34. Head, dorsal surface, pair(s) of trichobothria, presence

0 absent

1 present

35. Head, integument, presence

0 smooth

1 with microscupture

36. Head, dorsal side, punctation

0 lack of distinctive punctation or surface smooth

1 rugose

37. Head, ventral basal ridge, development

0 underdeveloped or absent

1 fully developed

38. Head, infraorbital ridge, presence

0 absent

1 present

39. Head, postgenal ridge, presence

0 absent

1 present

40. Head, nuchal ridge, presence

0 present

1 absent

41. Head, posterior margin, temples, shape

0 straight

1 rounded

42. Head, ventral, gular sutures, development

0 fully separated, but relatively close

1 partially fused

2 fully fused

3 absent

4 wide apart

43. Head, gular sutures, development at base of gula

0 continue through neck to posterior margin of head (posterior orifice)

1 become indistinct (fade) not reaching posterior margin of head

44. Neck, postoccipital suture vs. gula

0 postoccipital suture do not cross gular sutures, not distinct at base of gula

1 postoccipital suture crosses gular sutures and continues through base of gula

45. Neck, width

0 very narrow, equal or less than 1/5 of head width

1 narrow, equal or less than 1/3 of head width

2 regular, equal or less than 1/2 of head width

3 wide, more than 1/2 of head width

4 very wide, as wide as head

46. Dense hair on head, pronotum and elytra, presence

0 absent

1 present

47. Head vs. pronotum, width

0 head narrower or as wide as pronotum

1 head wider than pronotum

48. Head vs. pronotum, length

0 head shorter or as long as pronotum

1 head longer than pronotum

49. Pronotum, length

0 wider than long or quadrate

1 longer than wide

50. Pronotum, widest at

0 base

1 apex or anterior to its middle

2 middle

3 same width everywhere

51. Pronotum, front angles in dorsal view

0 not producing over anterior margin of pronotum

1 producing over anterior margin of pronotum

52. Pronotum, front angles, shape

0 regular

1 obtuse (apex of pronotum with neck-like projection)

53. Pronotum, midline, punctation

0 less punctation than the rest of pronotum, often appears impunctate

1 as punctated as the rest of pronotum (or not punctated if no punctation in general)

54. Prothorax, antesternal plates, development

0 absent

1 present

2 sclerotised membrane in place of plates

55. Prothorax, additional sclerotisation on anterior margin of prosternum, presence

0 absent

1 present

56. Pronotum, superior marginal line, development

0 not deflexed

1 deflexed

2 absent

57. Pronotum, superior marginal line vs. inferior line

0 not meeting each other

1 meeting or very close to each other

58. Pronotum, postcoxal process of hypomeron, development:

0 well developed and sclerotised

1 translucent, somewhat flexible, or absent

59. Prothorax, front angles of pronotum vs. prosternum in ventral view

0 front angles not produced beyond the meeting point of prosternum and pronotum

1 front angles produced beyond the meeting point of prosternum and pronotum

60. Prosternum, pronotosternal suture, development

0 well developed

1 poorly developed or absent

61. Prosternum, basisternum, surface

0 smooth

1 with punctation or wrinkled

62. Prosternum, basisternum, transversal carina, presence

0 absent

1 present

63. Prosternum, basisternum, longitudinal median carina, presence

0 absent

1 present

64. Prosternum, furcasternum, sharp longitudinal carina, presence

0 present

1 absent

65. Prosternum, furcasternum, transversal carina, presence

0 absent

1 present

66. Prosternum, furcasternum, relative extension basad

0 less extended - its pointy edge does not reach the level of the tip of postcoxal process

1 more extended - its pointy edge reaches the level of the tip of postcoxal process

67. Prosternum, furcasternum, lateral extension

0 not expanded under anterior coxae

1 expanded under anterior coxae

68. Prosternum, furcasternum, length

0 longer than 1/2 of basisternum length

1 shorter or equal to 1/2 of basisternum length

2 longer than basisternum

69. Prosternum, furcasternum, shape

0 triangular

1 rectangular

70. Prothorax, prosternum, prosternal apophysis as clear invagination, presence

0 present

1 absent

71. Mesospiracular peritremes, development

0 distinct

1 reduced

72. Mesothorasic membrane (area under anterior coxae), degree of sclerotisation

0 soft, without strongly sclerotised areas

1 with strongly or fully sclerotised areas embedding spiracles

2 not visible, hidden behind furcasternum

73. Mesosternum, sterno-pleural sutures, shape

0 curved, converging towards each other in their basal (closer to prepecturs) half

1 curved, running parallel to each other in their basal (closer to prepecus) half

2 straight, running transversly along entire extension

74. Mesosternum, sterno-pleural sutures, basal end

0 ending at prepectus

1 ending before (not reaching) prepectus

75. Mesosternum, basisternum, longitudinal carina, presence

0 absent

1 present

76. Mesosternum, furcasternum, longitudinal intercoxal carina, presence

0 present

1 absent

77. Mesosternum, transversal carina between sterno-pleural sutures

0 straight or interrupted in the middle

1 pointed apicad (towards abdomen)

2 pointed or curved basad (towards prothorax)

3 absent

78. Mesosternum, lateral ridges near (apicad from) prepectus, presence and/or number

0 absent

1 present, single

2 present, double

79. Mesosternum, connection to metasternum

0 mesosternum clearly separated from metasternum by a membrane

1 mesosternum separated from metasternum by suture, no membrane

2 mesosternum completely fused to metasternum

80. Mesosternum, ridge below coxal rests, presence

0 present

1 absent

81. Mesothorax, scutellum, scutellar ridge(s), presence

0 absent

1 only one present

2 both anterior and posterior present

82. Mesothorax, elytron, humeral spines or spine-like setae, presence

0 absent

1 present

83. Mesothorax, elytra, epipleuron, marginal ridge, presence

0 present

1 absent

84. Mesothorax, epipleuron, additional ridge (in addition to marginal ridge), presence

0 absent

1 present

85. Mesothorax, elytra, overlap

0 absent

1 present

86. Mesothorax, elytra, elongated elytra bases

0 absent

1 present

87. Protibia, comb-like rows of setae, presence

0 present

1 absent

88. Protibia, comb-like rows of setae, position relative to the long axis of tibia

0 transversally

1 diagonally

2 longitudinally

89. Protibia, comb-like rows of setae, number of fully developed rows

0 2

1 3

2 4

3 more

90. Protibia, comb-like rows of setae, number of setae

0 up to 3

1 many

2 no setae, spines instead

91. Protibia, comb-like rows of setae, position on the tibia

0 closer to tarsus

1 closer to femur or equal distance

92. Protibia, expanded area for comb-like rows of setae, presence

0 absent

1 present

93. Protarsus, basal four tarsomeres, compared to those of meso- and metatarsi, width

0 narrower or equal to meso- and metatarsomeres

1 wider, at most twice as wide as meso- and metatarsomeres

2 more than twice as wide as meso- and metatarsomeres

94. Protarsus, dense pale adhesive setae underneath, presence

0 present

1 absent

95. Protarsus, tarsomere 4

0 bilobed

1 not bilobed

96. Mesotibia, outer edge, thorns, presence

0 present

1 absent

97. Mesotibiae, outer side, long bristles, presence

0 present

1 absent

98. Mesotarsomere 1, length

0 equal to or longer than mesotarsomere 2

1 shorter than mesotarsomere 2

99. Meso- and metatarsomere 4

0 similar to the proceeding one

1 modified, either widened or bilobed, with dense pale of adhesive setae underneath

100. Metacoxa, suture

0 absent

1 present

2 different surface

101. Metatibia, apical ctenidium, presence

0 absent

1 present

102. Metatibia, apical ctenidium, position

0 on both anterior and posterior faces

1 on one side only

103. Metatibia, width

0 same along whole length

1 apically expanded

104. Tarsal formula

0 5-5-5

1 4-4-4

105. Metatarsomere 1, length

0 equal to or longer than metatarsomere 2

1 shorter than metatarsomere 2

106. Metatarsi, tarsomeres 1 and 5, length

0 tarsomere 1 shorter than 5

1 tarsomere 1 equal to or longer than 5

107. Metatarsi, tarsomeres 4 and 5, length

0 tarsomere 4 equal to or shorter than 5

1 tarsomere 4 longer than 5

108. Metatarsi, tarsomere 5 vs tarsomeres 2-4, length

0 tarsomere 5 equal to 2-4 combined

1 tarsomere 5 shorter than 2-4 combined

109. Tarsi, empodial setae, length

0 longer or equal to claws

1 half or less shorter than claws

110. Elytra, row of setae on the edge of the posterior margin, presence

0 absent

1 present

111. Hind wing, venation, MP3 vein, presence

0 present

1 absent

2 apterous

112. Hind wing, venation, veins MP4 and CuA, development

0 completely separated

1 largely or completely fused

2 apterous

113. Abdomen, tergite 1, protergal glands, presence

0 absent

1 present

114. Abdomen, tergites IV-VII, paratergites, presence

0 present

1 absent

115. Abdomen, segment VII, separation

0 tergite and sclerite separated

1 tergite and sclerite fused

116. Intersegmental membrane, pattern of sclerites

0 regular, brick-wall, sclerites hexagonal, rectangular or quadrangular

1 irregular, angular (mostly triangular) sclerites

2 irregular, rounded sclerites

3 small, quadrangular sclerites

4 no pattern

117. Intersegmental membrane, sclerites, degree of sclerotisation

0 weakly sclerotised

1 strongly sclerotised

118. Abdomen, sternite III, keel between metacoxae, presence

0 present

1 absent

119. Abdomen, sternite IV, anteromedian gland, presence

0 absent

1 present

120. Abdomen, tergites IX, shape

0 produced into flat, apically obtuse to sharp, sometimes with spine-like process

1 produced into inflated, apically sharp process

2 produced into inflated, apically obtuse or rounded process

121. Male, aedeagus, paired parameres, presence

0 present

1 highly reduced or absent

2 present but fused into one
