# Supplementary material for: The oldest case of paedomorphosis in rove beetles and description of a new genus of Paederinae from Cretaceous amber (Coleoptera: Staphylinidae)
Source: Sci Rep. 2023 Mar 31;13:5317. doi: 10.1038/s41598-023-32446-2 (PMC10066364; doi:10.1038/s41598-023-32446-2)
Supplement: Supplementary file 5 — Supplementary Information 5. [file 41598_2023_32446_MOESM5_ESM.pdf]

```
#NEXUS
[written Thu Feb 09 11:17:18 CET 2023 by Mesquite  version 3.70 (build 
940)]

BEGIN TAXA;
      TITLE Taxa;
      DIMENSIONS NTAX=52;
      TAXLABELS
            Achenomorphus Astenus 'Astenus_USA' Cylindroxystus 
Dibelonetes Diochus Domene Dysanabatium Echiaster Enallagium Eustilicus 
Haplonazeris Hyperomma Lathrobium Leptobium Lithocharis Lobrathium 
Lordithon Medon 'Medonina_Russia' Neolindus Neosclerus Notobium 
Ochthephilum Oedichirus Orus Oxyporus Paederus Pinophilus Pseudolathra 
Pseudomedon Quedius Ronetus Rugilus Sciocharis Scioporus Scopaeus 
Stilicoderus Stilicopsis Suniotrichus Sunius Tachyporus Tetartopeus 
Thinocharis Thyreocephalus Acanthoglossa Diminudon Luzea Micrillus 
'Midinudon_gen_nov' Ophioomma Scymbalium 
      ;

END;


BEGIN CHARACTERS;
      TITLE  Character_Matrix;
      DIMENSIONS  NCHAR=121;
      FORMAT DATATYPE = STANDARD RESPECTCASE GAP = - MISSING = ? SYMBOLS 
= "  0 1 2 3 4 5";
      CHARSTATELABELS 
            1 'Antennae, form' /  'non-geniculate' geniculate, 
            2 'Antennae, base of antennomere 1' / 
concealed_to_different_extent_thus_not_fully_visible_from_above 
fully_exposed_and_visible_from_above, 
            3 'Head, ''shelf'' concealing antennal bases, presence' / 
absent present, 
            4 'Antennae, antennomere 3, tomentose pubescence, presence' / 
absent present, 
            5 'Antennae, antennomere 4, tomentose pubescence, presence' / 
absent present, 
            6 'Antennae, antennomeres 9 and 10, each of them, shape' / 
'elongated, filiform, thin' 'elongated, egg or funnel shaped' 
wider_than_long, 
            7 'Antennae, antennomere 11, shape' /  radially_symmetrical 
radially_asymmetrical, 
            8 'Antennae, setation' / 
without_long_setae_extending_perpendicular_to_the_antennal_axis 
with_long_setae_extending_perpendicular_to_the_antennal_axis, 
            9 'Antennae, distance between bases' / 
_longer_than_distance_from_base_of_antenna_to_anterior_margin_of_eye 
equal_to_or_smaller_than_distance_from_base_of_antenna_to_anterior_margin
_of_eye, 
            10 'Eyes, setae between ommatidia, presence' /  absent 
present, 
            11 'Head, Y-shaped longitudinal grooves on dorsal surface 
between eyes, presence' /  absent present, 
            12 'Head, labrum, shape' /  'quadrate/rectangular, width less 
than twice the length' 'transverse, width equal to  twice the length or 
broader', 
```

13 'Head, labrum, size' / 'not expanded, at least half of mandibles visible when closed' 'expanded, almost or completely covering mandibles when closed',
14 'Head, labrum, development' / entire notched_to_different_extent dentate,
15 'Head, labrum, depth of emargination' / 'shallow, basally not reaching membrane connecting labrum with frons (frontoclypeus)' 'deep, basally extended through membrane connecting labrum with frons (frontoclypeus)',
16 'Head, labrum, apical membrane, presence' / absent present,
17 'Head, labrum, attachment to frons (frontoclypeus)' / with_distinct_membrane_between_frons_and_base_of_labrum without_distinct_membrane_between_frons_and_base_of_labrum,
18 'Head, maxillary palpomere 3, shape' / 'elongated, regular' 'expanded, more fusiform' 'slightly expanded, vase-like',
19 'Head, maxillary palpomere 4, shape' / fusiform conical 'nipple-like, elongate' 'small, acicular' 'truncate, short' securiform,
20 'Head, maxillary palpomere 4, width' / narrower_than_palpomere_3 as_wide_or_almost_as_wide_as_palpomere_3 wider_than_palpomere_3,
21 'Head, maxillary palpomeres 3 and 4, length' / palpomere_3_equal_to_or_shorter_than_4 palpomere_3_longer_than_4,
22 'Head, maxillary palpomere 4, setation' / glabrous_or_at_most_sparsely_setose densely_setose,
23 'Head, labial palpomeres 2 and 3, length' / palpomere_2_equal_to_or_longer_than_3 palpomere_2_shorter_than_3,
24 'Head, ligula, development' / bilobed entire reduced trilobed,
25 'Head, ligula, dorsal plate, setation' / absent present,
26 'Labial palpomere 3, width' / same_as_palpomere_2 thiner_than_palpomere_2 wider_than_palpomere_2,
27 'Head, mandibules, projection' / strongly_projected_anteriad bent_laterally,
28 'Head, mandibules, dorso-lateral groove, presence' / absent present,
29 'Head, mandibular prostheca, presence' / present absent,
30 'Head, mandibles, teeth, presence' / present absent,
31 'Head, mandibles, shape ' / 'stout (at least twice as wide at base as width at apical portion) ' 'thin (as wide at base as apical portion or slightly wider)',
32 'Head, mandibles, left vs right' / 'mandibles symmetrical (but size of same teeth may slightly differ on left and right mandible)' 'mandibles asymmetrical (when size, shape or sometimes number of teeth clearly differ on left and right madible)',
33 'Head, frons (i.e. the area of the head between eyes), punctation' / more_sparse_than_on_the_rest_of_head as_on_the_rest_of_head,
34 'Head, dorsal surface, pair(s) of trichobothria, presence' / absent present,
35 'Head, integument, presence' / smooth with_microscupture,
36 'Head, dorsal side, punctation' / lack_of_distinctive_punctation_or_surface_smooth rugose,
37 'Head, ventral basal ridge, development' / underdeveloped_or_absent fully_developed,
38 'Head, infraorbital ridge, presence' / absent present,
39 'Head, postgenal ridge, presence' / absent present,

40 'Head, nuchal ridge, presence' / present absent,
41 'Head, posterior margin, temples, shape' / straight rounded,
42 'Head, ventral, gular sutures, development ' / 'fully separated, but relatively close' partially_fused fully_fused absent wide_apart,
43 'Head, gular sutures, development at base of gula' / 'continue through neck to posterior margin of head (posterior orifice)' 'become indistinct (fade) not reaching posterior margin of head',
44 'Neck, postoccipital suture vs. gula' / 'postoccipital suture do not cross gular sutures, not distinct at base of gula' postoccipital_suture_crosses_gular_sutures_and_continues_through_base_of_gula_,
45 'Neck, width' / 'very narrow, equal or less than 1/5 of head width' 'narrow, equal or less than 1/3 of head width' 'regular, equal or less than 1/2 of head width' 'wide, more than 1/2 of head width' 'very wide, as wide as head',
46 'Dense hair on head, pronotum and elytra, presence' / absent present,
47 'Head vs. pronotum, width' / head_narrower_or_as_wide_as_pronotum head_wider_than_pronotum,
48 'Head vs. pronotum, length' / head_shorter_or_as_long_as_pronotum head_longer_than_pronotum,
49 'Pronotum, length' / wider_than_long_or_quadrate longer_than_wide,
50 'Pronotum, widest at' / base apex_or_anterior_to_its_middle middle same_width_everywhere,
51 'Pronotum, front angles in dorsal view' / not_producing_over_anterior_margin_of_pronotum producing_over_anterior_margin_of_pronotum,
52 'Pronotum, front angles, shape' / regular 'obtuse (apex of pronotum with neck-like projection)',
53 'Pronotum, midline, punctation' / 'less punctation than the rest of pronotum, often appears impunctate' 'as punctated as the rest of pronotum (or not punctated if no punctation in general)',
54 'Prothorax, antesternal plates, development' / absent present sclerotised_membrane_in_place_of_plates,
55 'Prothorax, additional sclerotisation on anterior margin of prosternum, presence' / absent present,
56 'Pronotum, superior marginal line, development' / not_deflexed deflexed absent,
57 'Pronotum, superior marginal line vs. inferior line' / not_meeting_each_other meeting_or_very_close_to_each_other,
58 'Pronotum, postcoxal process of hypomeron, development: ' / well_developed_and_sclerotised 'translucent, somewhat flexible, or absent',
59 'Prothorax, front angles of pronotum vs. prosternum in ventral view' / front_angles_not_produced_beyond_the_meeting_point_of_prosternum_and_pronotum front_angles_produced_beyond_the_meeting_point_of_prosternum_and_pronotum,
60 'Prosternum, pronotosternal suture, development' / well_developed poorly_developed_or_absent,
61 'Prosternum, basisternum, surface ' / smooth with_punctation_or_wrinkled,

62 'Prosternum, basisternum, transversal carina, presence' / absent present,
63 'Prosternum, basisternum, longitudinal median carina, presence' / absent present,
64 'Prosternum, furcasternum, sharp longitudinal carina, presence' / present absent,
65 'Prosternum, furcasternum, transversal carina, presence' / absent present,
66 'Prosternum, furcasternum, relative extension basad' / 'less extended - its pointy edge does not reach the level of the tip of postcoxal process' 'more extended - its pointy edge reaches the level of the tip of postcoxal process',
67 'Prosternum, furcasternum, lateral extension' / not_expanded_under_anterior_coxae expanded_under_anterior_coxae,
68 'Prosternum, furcasternum, length' / 'longer than 1/2 of basisternum length' 'shorter or equal to 1/2 of basisternum length' longer_than_basisternum,
69 'Prosternum, furcasternum, shape ' / triangular rectangular,
70 'Prothorax, prosternum, prosternal apophysis as clear invagination, presence' / present absent,
71 'Mesospiracular peritremes, development' / distinct reduced,
72 'Mesothorasic membrane (area under anterior coxae), degree of sclerotisation' / 'soft, without strongly sclerotised areas' with_strongly_or_fully_sclerotised_areas_embedding_spiracles 'not visible, hidden behind furcasternum',
73 'Mesosternum, sterno-pleural sutures, shape' / 'curved, converging towards each other in their basal (closer to prepecturs) half' 'curved, running parallel to each other in their basal (closer to prepecus) half' 'straight, running transversly along entire extension',
74 'Mesosternum, sterno-pleural sutures, basal end' / ending_at_prepectus 'ending before (not reaching) prepectus',
75 'Mesosternum, basisternum, longitudinal carina, presence ' / absent present,
76 'Mesosternum, furcasternum, longitudinal intercoxal carina, presence' / present absent,
77 'Mesosternum, transversal carina between sterno-pleural sutures' / straight_or_interrupted_in_the_middle 'pointed apicad (towards abdomen)' 'pointed or curved basad (towards prothorax)' absent,
78 'Mesosternum, lateral ridges near (apicad from) prepectus, presence and/or number' / absent 'present, single' 'present, double',
79 'Mesosternum, connection to metasternum' / mesosternum_clearly_separated_from_metasternum_by_a_membrane 'mesosternum separated from metasternum by suture, no membrane' mesosternum_completely_fused_to_metasternum,
80 'Mesosternum, ridge below coxal rests, presence ' / present absent,
81 'Mesothorax, scutellum, scutellar ridge(s), presence' / absent only_one_present both_anterior_and_posterior_present,
82 'Mesothorax, elytron, humeral spines or spine-like setae, presence ' / absent present,
83 'Mesothorax, elytra, epipleuron, marginal ridge, presence' / present absent,
84 'Mesothorax, epipleuron, additional ridge (in addition to marginal ridge), presence' / absent present,
85 'Mesothorax, elytra, overlap' / absent present,

86 'Mesothorax, elytra, elongated elytra bases' / absent present,
87 'Protibia, comb-like rows of setae, presence' / present absent,
88 'Protibia, comb-like rows of setae, position relative to the long axis of tibia' / transversally diagonally longitudinally,
89 'Protibia, comb-like rows of setae, number of fully developed rows' / 2 3 4 more,
90 'Protibia, comb-like rows of setae, number of setae' / up_to_3 many 'no setae, spines instead',
91 'Protibia, comb-like rows of setae, position on the tibia ' / closer_to_tarsus closer_to_femur_or_equal_distance,
92 'Protibia, expanded area for comb-like rows of setae, presence' / absent present,
93 'Protarsus, basal four tarsomeres, compared to those of meso- and metatarsi, width' / 'narrower or equal to meso- and metatarsomeres' 'wider, at most twice as wide as meso- and metatarsomeres' 'more than twice as wide as meso- and metatarsomeres',
94 'Protarsus, dense pale adhesive setae underneath, presence' / present absent,
95 'Protarsus, tarsomere 4' / bilobed not_bilobed,
96 'Mesotibia, outer edge, thorns, presence' / present absent,
97 'Mesotibiae, outer side, long bristles, presence' / present_ absent,
98 'Mesotarsomere 1, length' / equal_to_or_longer_than_mesotarsomere_2 shorter_than_mesotarsomere_2,
99 'Meso- and metatarsomere 4' / similar_to_the_proceeding_one 'modified, either widened or bilobed, with dense pale of adhesive setae underneath',
100 'Metacoxa, suture' / absent present different_surface,
101 'Metatibia, apical ctenidium, presence' / absent present,
102 'Metatibia, apical ctenidium, position' / on_both_anterior_and_posterior_faces on_one_side_only,
103 'Metatibia, width' / same_along_whole_length apically_expanded,
104 Tarsal_formula / '5-5-5' '4-4-4',
105 'Metatarsomere 1, length' / equal_to_or_longer_than_metatarsomere_2 shorter_than_metatarsomere_2,
106 'Metatarsi, tarsomeres 1 and 5, length' / tarsomere_1_shorter_than_5 tarsomere_1_equal_to_or_longer_than_5,
107 'Metatarsi, tarsomeres 4 and 5, length' / tarsomere_4_equal_to_or_shorter_than_5 tarsomere_4_longer_than_5,
108 'Metatarsi, tarsomere 5 vs tarsomeres 2-4, length' / 'tarsomere 5 equal to 2-4 combined' 'tarsomere 5 shorter than 2-4 combined',
109 'Tarsi, empodial setae, length' / longer_or_equal_to_claws half_or_less_shorter_than_claws,
110 'Elytra, row of setae on the edge of the posterior margin, presence' / absent present,
111 'Hind wing, venation, MP3 vein, presence' / present absent apterous,
112 'Hind wing, venation, veins MP4 and CuA, development ' / completely_separated largely_or_completely_fused apterous,
113 'Abdomen, tergite 1, protergal glands, presence' / absent present,

```
           114 'Abdomen, tergites IV-VII, paratergites, presence' / 
present absent,
           115 'Abdomen, segment  VII, separation' / 
tergite_and_sclerite_separated tergite_and_sclerite_fused,
           116 'Intersegmental membrane, pattern of sclerites' / 
'regular, brick-wall, sclerites hexagonal, rectangular or quadrangular' 
'irregular, angular (mostly triangular) sclerites' 'irregular, rounded 
sclerites' 'small, quadrangular sclerites' no_pattern,
           117 'Intersegmental membrane, sclerites, degree of 
sclerotisation' /  weakly_sclerotised strongly_sclerotised,
           118 'Abdomen, sternite III, keel between metacoxae, presence' 
/  present absent,
           119 'Abdomen, sternite IV, anteromedian gland, presence' / 
absent present,
           120 'Abdomen, tergites IX, shape' /  'produced into flat, 
apically obtuse to sharp, sometimes with spine-like process' 'produced 
into inflated, apically sharp process' 'produced into inflated, apically 
obtuse or rounded process',
           121 'Male, aedeagus, paired parameres, presence' /  present 
highly_reduced_or_absent present_but_fused_into_one ;
      MATRIX
      Achenomorphus
0011110101010100003010001010000010101000001121000100000110011110110001001
1000200200001020010001110021100010101000000000001
      Astenus
0011111000010200113010000110101010011000121111111011011100111000112111121
1000221100001020010000110121100011100110000000001
      'Astenus_USA'
0011111000010200113010000?1010101001100012111111110110111001110001121112 1
10002211000010200100001101211000111 0?--000000001
      Cylindroxystus
0010110100010100012010000010011000001000041120001200000110001010100001001
1102210200101011010101110021000000000110100000 11
      Dibelonetes
0011111000011200113010010110101010011000121111110101101100011110011211121
1002220000001020010000110121100010100110000000 01
      Diochus
0001100110000100003010001010000000100000110010001000120101100000000111000
0103001111 0001-----0110100011000101 10--000000000
      Domene
0011110100000100021010001010000010010000101110001101100100001000100001001
11012002001010020111011110210101 00000--000000011
      Dysanabatium
0011110100000100021010001011000000101000101120101100000110010000100001001
1101200000001003110100100021000000100110000000 01
      Echiaster
001002010101120011301001011010111001100012110111120110 02-
0011100011211121100120120100102001001111002110001 0100--000000001
      Enallagium
0011110101010200021010000111000110011000001121111100000110011010110001001
100220020000100111010110102100010011100000000000?
      Eustilicus
0011110101011200023010000111100110111000021101101101100100011010110000001
1002100100001020110001110021100010101000000000001
      Haplonazeris
0011110101010200013010011110101110111000120111101101100110011010111010121
0002210200001020010101110011100010100--000000?00
```

```
      Hyperomma
00111101000101000221101000100000000010001000300012000001000110000000001011
1002100200101000010001010001000010100--000000110
      Lathrobium
00111101000001000210100010100000101010001011200013000001000000000100001001
1101200200001002011101101021010100000--000000011
      Leptobium
00100101000101000141110010100000000000001000300012100001000110000000001011
1002200200001003110101010011000010101--000000110
      Lithocharis        00111101010100-
01130100000100001101010000011210001000001000110000000010011000200200000102
00100001110021100010101--000001001
      Lobrathium
0011110100000100021010001010000000101000101120101100000100011000100001001
11012002001010020111011010210101000000000000011
      Lordithon          01000201000000-
010010010101?010010100001100040000000010000110000000001100200130001001001-
----011010000-0001011100000400000
      Medon
0010020101010200013010000110100110001000001121000100000100011000110001001
110220020000102001000111002110001010011000000001
      'Medonina_Russia'
0011110101000100013010001111000100001000001121100100000110011000110001001
100210010000100011000111102110000010111000000001
      Neolindus          00100201000100-
0012010020010011001001000041120001200000100001010100001011100221020010101
10101011000210?000010000010000011
      Neosclerus
0010021101010100013010001110000100101000001110000100000100011010110001001
100220020000102001000111002110001010100000000001
      Notobium
0010110100000100001110001011000000101000001130101100000100001010100001001
110220020010100311010001002100010000100000000000
      Ochthephilum
1011110100010100022110000010000000001000100030001200000100001010000000000
000210020000100101001101001100001010011000000111
      Oedichirus
001110010001010012520100101000000000000011300100011001002-
000100000000000101003100000001003100201100111010010110--011010010
      Orus
0011120101010200013010031111000101001000001121101101000110011000110001001
1002200200001001010011110021100000101--000000001
      Oxyporus
0010020100000111100100021201000110000011100040000100100100000100010011002
10130201100011-----011011000-00100000--000001001
      Paederus
00100101000101000141110011100000000000001000200011010002-
0010001000000001100212120000100101010011011110001010011000000110
      Pinophilus
0011100100010100125201001010000010100001100020000110100000111010000000010
110210000100100311020111011101001011100000010001
      Pseudolathra
0010110100000100011010000010000000101000101120001200000100000010100001001
100120020010101211010110002100000000101000000011
      Pseudomedon
0011120101010100013010000110100110001000001121000100100100011010110001001
100120020000102001010111002110001010111000000001
```

Quedius
0100111100010101000100100010000110101110100040000010100001100001000111002
00030002110001-----101010000-0001011100100201022
Ronetus
00100201010112001130100101101011100110001211011112011002-
00110000112111211003201201001020010011110021100010100--000000001
Rugilus
0010010101011200023010010110100110011000021001100101000100011000110000001
1002101101001020010001110021100010100110000000001
Sciocharis
00100001010112000230100 10?101000100110000011111001000001000110001100010 01
100210010000102??1000111002110000010100000000001
Scioporus
0011110101010100013010001110?001000100000001201101000001000010101102000 01
10022102000010210101011100110000001000000000?01
Scopaeus
00111100010102000130100301100000111010000011011011011002-
00110000100010011002201200001001010011110021100000100--000000001
Stilicoderus
0011110100011200023010010110100110011000021001100101000100011000110000001
1102100100001020110001110021100010100010000000001
Stilicopsis 00111110000110-
0113010010110101110011000121111110101001110011010011211121100222100000102
0010000110121100010100--000000001
Suniotrichus
0011110101010200003010001110100010101000011111110100001100011010110001001
1002200200001020110001110021100010101000000000001
Sunius
0010020101010200013010001110000100001000001120001100000100011000100001001
1002200200001020010001110021100010101--000000001
Tachyporus 00000111000100-
0100110001011010010000001100040000010100001100000000011012001300010010 01-
----011010000-0001011011000401000
Tetartopeus
0011110100000100021010001010000010100000101110001100000100000000100001001
1101200200001002011101101021010100000--000000011
Thinocharis
0010000101011200023010010110100010011000011111100101000100011000110001001
1002100101001020110001110021100010101110000000001
Thyreocephalus
1000120110110100000100111010000100101000021010101100110111110000000101002
001301111101000020000101102110000011101000101000
Acanthoglossa 00111201010100-
0013010000110000100001000001111010100000110011000110001002100210010000102
001010011012110001010111000000001
Diminudon 00111201010000-
0?100100???01?0001011?000?01?20111010001?0011000?1?00????????2?02000010?
???00111100011010000?1???00000?0?
Luzea
0010120101010200013010011110000100101000001121001100000110011010010001001
1002200200001020010001110021100000101110000000001
Micrillus
0011110100000110001110000010000000100000000130001100000110010000100001000
0002110200001003010001010021000010101000000000011
'Midinudon_gen_nov'001??201011000-
0?000100??101?0001010?000001?10111010001?0011000?1?0?????????????000010?
???00111100011010100????00?00?0?

```
      Ophioomma
00111101010101000130100000?1??01?1000100000111101010010010001101O??0?0??01
100???0?000010200101011100211000101??--00000?0?1
      Scymbalium
00101001000001110011100010100001001010001001300011100001000010001000010000
0000100200001003110101010021000010101000000000010

;

END;
BEGIN ASSUMPTIONS;
      TYPESET * UNTITLED   =  unord:  1- 121;

END;

BEGIN NOTES;

      SUT   TAXON = 40 NAME = color INTEGER = 4;

      SU  T = 34 C = 1 N = color I = 7;
      SU  T = 39 C = 1 N = color I = 13;
      SU  T = 52 C = 1 N = color I = 4;

      SU  T = 34 C = 2 N = color I = 7;
      SU  T = 39 C = 2 N = color I = 13;
      SU  T = 52 C = 2 N = color I = 4;

      SU  T = 34 C = 3 N = color I = 7;
      SU  T = 39 C = 3 N = color I = 13;
      SU  T = 52 C = 3 N = color I = 4;

      SU  T = 34 C = 4 N = color I = 7;
      SU  T = 39 C = 4 N = color I = 13;
      SU  T = 52 C = 4 N = color I = 4;

      SU  T = 34 C = 9 N = color I = 7;
      SU  T = 39 C = 9 N = color I = 13;
      SU  T = 52 C = 9 N = color I = 4;

      SU  T = 34 C = 11 N = color I = 7;
      SU  T = 39 C = 11 N = color I = 13;
      SU  T = 52 C = 11 N = color I = 4;

      SU  T = 1 C = 12 N = color I = 6;
      SU  T = 2 C = 12 N = color I = 6;
      SU  T = 4 C = 12 N = color I = 6;
      SU  T = 5 C = 12 N = color I = 6;
      SU  T = 7 C = 12 N = color I = 6;
      SU  T = 9 C = 12 N = color I = 6;
      SU  T = 11 C = 12 N = color I = 6;
      SU  T = 13 C = 12 N = color I = 6;
      SU  T = 14 C = 12 N = color I = 6;
      SU  T = 15 C = 12 N = color I = 6;
      SU  T = 16 C = 12 N = color I = 6;
      SU  T = 19 C = 12 N = color I = 6;
      SU  T = 21 C = 12 N = color I = 6;
      SU  T = 22 C = 12 N = color I = 6;
```

```
SU  T = 24 C = 12 N = color I = 6;
SU  T = 25 C = 12 N = color I = 6;
SU  T = 26 C = 12 N = color I = 6;
SU  T = 28 C = 12 N = color I = 6;
SU  T = 29 C = 12 N = color I = 6;
SU  T = 31 C = 12 N = color I = 6;
SU  T = 32 C = 12 N = color I = 6;
SU  T = 33 C = 12 N = color I = 6;
SU  T = 34 C = 12 N = color I = 6;
SU  T = 37 C = 12 N = color I = 6;
SU  T = 38 C = 12 N = color I = 6;
SU  T = 39 C = 12 N = color I = 6;
SU  T = 40 C = 12 N = color I = 6;
SU  T = 41 C = 12 N = color I = 6;
SU  T = 42 C = 12 N = color I = 6;
SU  T = 43 C = 12 N = color I = 6;
SU  T = 45 C = 12 N = color I = 6;
SU  T = 52 C = 12 N = color I = 4;

SU  C = 13 N = color I = 4;
SU  T = 1 C = 13 N = color I = 7;
SU  T = 2 C = 13 N = color I = 7;
SU  T = 4 C = 13 N = color I = 7;
SU  T = 5 C = 13 N = color I = 7;
SU  T = 7 C = 13 N = color I = 7;
SU  T = 9 C = 13 N = color I = 7;
SU  T = 11 C = 13 N = color I = 7;
SU  T = 13 C = 13 N = color I = 7;
SU  T = 14 C = 13 N = color I = 7;
SU  T = 15 C = 13 N = color I = 7;
SU  T = 16 C = 13 N = color I = 7;
SU  T = 19 C = 13 N = color I = 7;
SU  T = 21 C = 13 N = color I = 7;
SU  T = 22 C = 13 N = color I = 7;
SU  T = 24 C = 13 N = color I = 7;
SU  T = 25 C = 13 N = color I = 7;
SU  T = 26 C = 13 N = color I = 7;
SU  T = 28 C = 13 N = color I = 7;
SU  T = 29 C = 13 N = color I = 7;
SU  T = 31 C = 13 N = color I = 7;
SU  T = 32 C = 13 N = color I = 7;
SU  T = 33 C = 13 N = color I = 7;
SU  T = 34 C = 13 N = color I = 7;
SU  T = 37 C = 13 N = color I = 7;
SU  T = 38 C = 13 N = color I = 7;
SU  T = 39 C = 13 N = color I = 7;
SU  T = 40 C = 13 N = color I = 7;
SU  T = 41 C = 13 N = color I = 7;
SU  T = 42 C = 13 N = color I = 7;
SU  T = 43 C = 13 N = color I = 7;
SU  T = 45 C = 13 N = color I = 7;
SU  T = 52 C = 13 N = color I = 4;

SU  T = 1 C = 14 N = color I = 6;
SU  T = 2 C = 14 N = color I = 6;
SU  T = 4 C = 14 N = color I = 6;
SU  T = 5 C = 14 N = color I = 6;
```

```
SU  T = 7 C = 14 N = color I = 6;
SU  T = 9 C = 14 N = color I = 6;
SU  T = 11 C = 14 N = color I = 6;
SU  T = 13 C = 14 N = color I = 6;
SU  T = 14 C = 14 N = color I = 6;
SU  T = 15 C = 14 N = color I = 6;
SU  T = 16 C = 14 N = color I = 6;
SU  T = 19 C = 14 N = color I = 6;
SU  T = 21 C = 14 N = color I = 6;
SU  T = 22 C = 14 N = color I = 6;
SU  T = 24 C = 14 N = color I = 6;
SU  T = 25 C = 14 N = color I = 6;
SU  T = 26 C = 14 N = color I = 6;
SU  T = 28 C = 14 N = color I = 6;
SU  T = 29 C = 14 N = color I = 6;
SU  T = 31 C = 14 N = color I = 6;
SU  T = 32 C = 14 N = color I = 6;
SU  T = 33 C = 14 N = color I = 6;
SU  T = 34 C = 14 N = color I = 6;
SU  T = 37 C = 14 N = color I = 6;
SU  T = 38 C = 14 N = color I = 6;
SU  T = 39 C = 14 N = color I = 6;
SU  T = 40 C = 14 N = color I = 6;
SU  T = 41 C = 14 N = color I = 6;
SU  T = 42 C = 14 N = color I = 6;
SU  T = 43 C = 14 N = color I = 6;
SU  T = 45 C = 14 N = color I = 6;
SU  T = 52 C = 14 N = color I = 4;

SU  T = 52 C = 18 N = color I = 4;

SU  T = 34 C = 20 N = color I = 7;
SU  T = 39 C = 20 N = color I = 13;
SU  T = 52 C = 20 N = color I = 4;

SU  T = 34 C = 22 N = color I = 7;
SU  T = 39 C = 22 N = color I = 13;
SU  T = 52 C = 22 N = color I = 4;

SU  T = 1 C = 23 N = color I = 17;
SU  T = 2 C = 23 N = color I = 17;
SU  T = 3 C = 23 N = color I = 17;
SU  T = 4 C = 23 N = color I = 17;
SU  T = 5 C = 23 N = color I = 17;
SU  T = 7 C = 23 N = color I = 17;
SU  T = 8 C = 23 N = color I = 17;
SU  T = 9 C = 23 N = color I = 17;
SU  T = 11 C = 23 N = color I = 17;
SU  T = 13 C = 23 N = color I = 17;
SU  T = 14 C = 23 N = color I = 17;
SU  T = 15 C = 23 N = color I = 17;
SU  T = 16 C = 23 N = color I = 17;
SU  T = 18 C = 23 N = color I = 17;
SU  T = 19 C = 23 N = color I = 17;
SU  T = 21 C = 23 N = color I = 17;
SU  T = 22 C = 23 N = color I = 17;
SU  T = 23 C = 23 N = color I = 17;
```

```
SU  T = 24 C = 23 N = color I = 17;
SU  T = 25 C = 23 N = color I = 17;
SU  T = 26 C = 23 N = color I = 17;
SU  T = 28 C = 23 N = color I = 17;
SU  T = 29 C = 23 N = color I = 17;
SU  T = 30 C = 23 N = color I = 17;
SU  T = 31 C = 23 N = color I = 17;
SU  T = 32 C = 23 N = color I = 17;
SU  T = 33 C = 23 N = color I = 17;
SU  T = 34 C = 23 N = color I = 17;
SU  T = 37 C = 23 N = color I = 17;
SU  T = 38 C = 23 N = color I = 17;
SU  T = 39 C = 23 N = color I = 17;
SU  T = 40 C = 23 N = color I = 17;
SU  T = 41 C = 23 N = color I = 17;
SU  T = 42 C = 23 N = color I = 17;
SU  T = 43 C = 23 N = color I = 17;
SU  T = 44 C = 23 N = color I = 17;
SU  T = 45 C = 23 N = color I = 17;
SU  T = 52 C = 23 N = color I = 17;

SU  T = 1 C = 24 N = color I = 6;
SU  T = 2 C = 24 N = color I = 6;
SU  T = 4 C = 24 N = color I = 6;
SU  T = 5 C = 24 N = color I = 6;
SU  T = 7 C = 24 N = color I = 6;
SU  T = 9 C = 24 N = color I = 6;
SU  T = 11 C = 24 N = color I = 6;
SU  T = 13 C = 24 N = color I = 6;
SU  T = 14 C = 24 N = color I = 6;
SU  T = 15 C = 24 N = color I = 6;
SU  T = 16 C = 24 N = color I = 6;
SU  T = 19 C = 24 N = color I = 6;
SU  T = 21 C = 24 N = color I = 6;
SU  T = 22 C = 24 N = color I = 6;
SU  T = 24 C = 24 N = color I = 6;
SU  T = 25 C = 24 N = color I = 6;
SU  T = 26 C = 24 N = color I = 6;
SU  T = 28 C = 24 N = color I = 6;
SU  T = 29 C = 24 N = color I = 6;
SU  T = 31 C = 24 N = color I = 6;
SU  T = 32 C = 24 N = color I = 6;
SU  T = 33 C = 24 N = color I = 6;
SU  T = 34 C = 24 N = color I = 6;
SU  T = 37 C = 24 N = color I = 6;
SU  T = 38 C = 24 N = color I = 6;
SU  T = 39 C = 24 N = color I = 6;
SU  T = 40 C = 24 N = color I = 6;
SU  T = 41 C = 24 N = color I = 6;
SU  T = 42 C = 24 N = color I = 6;
SU  T = 43 C = 24 N = color I = 6;
SU  T = 45 C = 24 N = color I = 6;
SU  T = 52 C = 24 N = color I = 4;

SU  T = 1 C = 25 N = color I = 6;
SU  T = 2 C = 25 N = color I = 6;
SU  T = 4 C = 25 N = color I = 6;
```

```
SU  T = 5 C = 25 N = color I = 6;
SU  T = 7 C = 25 N = color I = 6;
SU  T = 9 C = 25 N = color I = 6;
SU  T = 11 C = 25 N = color I = 6;
SU  T = 13 C = 25 N = color I = 6;
SU  T = 14 C = 25 N = color I = 6;
SU  T = 15 C = 25 N = color I = 6;
SU  T = 16 C = 25 N = color I = 6;
SU  T = 19 C = 25 N = color I = 6;
SU  T = 21 C = 25 N = color I = 6;
SU  T = 22 C = 25 N = color I = 6;
SU  T = 24 C = 25 N = color I = 6;
SU  T = 25 C = 25 N = color I = 6;
SU  T = 26 C = 25 N = color I = 6;
SU  T = 28 C = 25 N = color I = 6;
SU  T = 29 C = 25 N = color I = 6;
SU  T = 31 C = 25 N = color I = 6;
SU  T = 32 C = 25 N = color I = 6;
SU  T = 33 C = 25 N = color I = 6;
SU  T = 34 C = 25 N = color I = 6;
SU  T = 37 C = 25 N = color I = 6;
SU  T = 38 C = 25 N = color I = 6;
SU  T = 39 C = 25 N = color I = 6;
SU  T = 40 C = 25 N = color I = 6;
SU  T = 41 C = 25 N = color I = 6;
SU  T = 42 C = 25 N = color I = 6;
SU  T = 43 C = 25 N = color I = 6;
SU  T = 45 C = 25 N = color I = 6;
SU  T = 52 C = 25 N = color I = 4;

SU  T = 34 C = 27 N = color I = 7;
SU  T = 39 C = 27 N = color I = 13;
SU  T = 52 C = 27 N = color I = 4;

SU  T = 34 C = 28 N = color I = 7;
SU  T = 39 C = 28 N = color I = 13;
SU  T = 52 C = 28 N = color I = 4;

SU  T = 34 C = 29 N = color I = 7;
SU  T = 39 C = 29 N = color I = 13;
SU  T = 52 C = 29 N = color I = 4;

SU  T = 52 C = 30 N = color I = 4;

SU  T = 52 C = 34 N = color I = 4;

SU  T = 52 C = 35 N = color I = 4;

SU  T = 34 C = 36 N = color I = 7;
SU  T = 39 C = 36 N = color I = 13;
SU  T = 52 C = 36 N = color I = 4;

SU  T = 34 C = 38 N = color I = 7;
SU  T = 39 C = 38 N = color I = 13;
SU  T = 52 C = 38 N = color I = 4;

SU  T = 34 C = 39 N = color I = 7;
```

```
SU  T = 39 C = 39 N = color I = 13;
SU  T = 52 C = 39 N = color I = 4;

SU  T = 1 C = 40 N = color I = 5;
SU  T = 2 C = 40 N = color I = 5;
SU  T = 3 C = 40 N = color I = 5;
SU  T = 4 C = 40 N = color I = 5;
SU  T = 5 C = 40 N = color I = 5;
SU  T = 7 C = 40 N = color I = 5;
SU  T = 9 C = 40 N = color I = 5;
SU  T = 11 C = 40 N = color I = 5;
SU  T = 13 C = 40 N = color I = 5;
SU  T = 14 C = 40 N = color I = 5;
SU  T = 15 C = 40 N = color I = 5;
SU  T = 16 C = 40 N = color I = 5;
SU  T = 18 C = 40 N = color I = 5;
SU  T = 19 C = 40 N = color I = 5;
SU  T = 21 C = 40 N = color I = 5;
SU  T = 22 C = 40 N = color I = 5;
SU  T = 24 C = 40 N = color I = 5;
SU  T = 25 C = 40 N = color I = 5;
SU  T = 26 C = 40 N = color I = 5;
SU  T = 28 C = 40 N = color I = 5;
SU  T = 29 C = 40 N = color I = 5;
SU  T = 30 C = 40 N = color I = 5;
SU  T = 31 C = 40 N = color I = 5;
SU  T = 32 C = 40 N = color I = 5;
SU  T = 33 C = 40 N = color I = 5;
SU  T = 34 C = 40 N = color I = 5;
SU  T = 37 C = 40 N = color I = 5;
SU  T = 38 C = 40 N = color I = 5;
SU  T = 39 C = 40 N = color I = 5;
SU  T = 40 C = 40 N = color I = 5;
SU  T = 41 C = 40 N = color I = 5;
SU  T = 42 C = 40 N = color I = 5;
SU  T = 43 C = 40 N = color I = 5;
SU  T = 44 C = 40 N = color I = 5;
SU  T = 45 C = 40 N = color I = 5;
SU  T = 52 C = 40 N = color I = 4;

SU  T = 34 C = 41 N = color I = 7;
SU  T = 39 C = 41 N = color I = 13;
SU  T = 52 C = 41 N = color I = 4;

SU  T = 52 C = 42 N = color I = 4;

SU  T = 34 C = 46 N = color I = 7;
SU  T = 39 C = 46 N = color I = 13;
SU  T = 52 C = 46 N = color I = 4;

SU  T = 34 C = 49 N = color I = 7;
SU  T = 39 C = 49 N = color I = 13;
SU  T = 52 C = 49 N = color I = 4;

SU  T = 34 C = 54 N = color I = 7;
SU  T = 39 C = 54 N = color I = 13;
SU  T = 52 C = 54 N = color I = 4;
```

```
SU  T = 52 C = 55 N = color I = 4;

SU  T = 34 C = 56 N = color I = 7;
SU  T = 39 C = 56 N = color I = 13;
SU  T = 52 C = 56 N = color I = 4;

SU  T = 34 C = 57 N = color I = 7;
SU  T = 39 C = 57 N = color I = 13;
SU  T = 52 C = 57 N = color I = 4;

SU  T = 34 C = 58 N = color I = 7;
SU  T = 39 C = 58 N = color I = 13;
SU  T = 52 C = 58 N = color I = 4;

SU  T = 1 C = 60 N = color I = 6;
SU  T = 2 C = 60 N = color I = 6;
SU  T = 4 C = 60 N = color I = 6;
SU  T = 5 C = 60 N = color I = 6;
SU  T = 7 C = 60 N = color I = 6;
SU  T = 9 C = 60 N = color I = 6;
SU  T = 11 C = 60 N = color I = 6;
SU  T = 13 C = 60 N = color I = 6;
SU  T = 14 C = 60 N = color I = 6;
SU  T = 15 C = 60 N = color I = 6;
SU  T = 16 C = 60 N = color I = 6;
SU  T = 19 C = 60 N = color I = 6;
SU  T = 21 C = 60 N = color I = 6;
SU  T = 22 C = 60 N = color I = 6;
SU  T = 24 C = 60 N = color I = 6;
SU  T = 25 C = 60 N = color I = 6;
SU  T = 26 C = 60 N = color I = 6;
SU  T = 28 C = 60 N = color I = 6;
SU  T = 29 C = 60 N = color I = 6;
SU  T = 31 C = 60 N = color I = 6;
SU  T = 32 C = 60 N = color I = 6;
SU  T = 33 C = 60 N = color I = 6;
SU  T = 34 C = 60 N = color I = 6;
SU  T = 37 C = 60 N = color I = 6;
SU  T = 38 C = 60 N = color I = 6;
SU  T = 39 C = 60 N = color I = 6;
SU  T = 40 C = 60 N = color I = 6;
SU  T = 41 C = 60 N = color I = 6;
SU  T = 42 C = 60 N = color I = 6;
SU  T = 43 C = 60 N = color I = 6;
SU  T = 45 C = 60 N = color I = 6;
SU  T = 52 C = 60 N = color I = 4;

SU  T = 52 C = 61 N = color I = 4;

SU  T = 1 C = 65 N = color I = 17;
SU  T = 2 C = 65 N = color I = 17;
SU  T = 3 C = 65 N = color I = 17;
SU  T = 4 C = 65 N = color I = 17;
SU  T = 5 C = 65 N = color I = 17;
SU  T = 7 C = 65 N = color I = 17;
SU  T = 8 C = 65 N = color I = 17;
```

```
SU  T = 9 C = 65 N = color I = 17;
SU  T = 11 C = 65 N = color I = 17;
SU  T = 13 C = 65 N = color I = 17;
SU  T = 14 C = 65 N = color I = 17;
SU  T = 15 C = 65 N = color I = 17;
SU  T = 16 C = 65 N = color I = 17;
SU  T = 18 C = 65 N = color I = 17;
SU  T = 19 C = 65 N = color I = 17;
SU  T = 21 C = 65 N = color I = 17;
SU  T = 22 C = 65 N = color I = 17;
SU  T = 23 C = 65 N = color I = 17;
SU  T = 24 C = 65 N = color I = 17;
SU  T = 25 C = 65 N = color I = 17;
SU  T = 26 C = 65 N = color I = 17;
SU  T = 28 C = 65 N = color I = 17;
SU  T = 29 C = 65 N = color I = 17;
SU  T = 30 C = 65 N = color I = 17;
SU  T = 31 C = 65 N = color I = 17;
SU  T = 32 C = 65 N = color I = 17;
SU  T = 33 C = 65 N = color I = 17;
SU  T = 34 C = 65 N = color I = 17;
SU  T = 37 C = 65 N = color I = 17;
SU  T = 38 C = 65 N = color I = 17;
SU  T = 39 C = 65 N = color I = 17;
SU  T = 40 C = 65 N = color I = 17;
SU  T = 41 C = 65 N = color I = 17;
SU  T = 42 C = 65 N = color I = 17;
SU  T = 43 C = 65 N = color I = 17;
SU  T = 44 C = 65 N = color I = 17;
SU  T = 45 C = 65 N = color I = 17;
SU  T = 52 C = 65 N = color I = 17;

SU  T = 1 C = 68 N = color I = 17;
SU  T = 2 C = 68 N = color I = 17;
SU  T = 3 C = 68 N = color I = 17;
SU  T = 4 C = 68 N = color I = 17;
SU  T = 5 C = 68 N = color I = 17;
SU  T = 7 C = 68 N = color I = 17;
SU  T = 8 C = 68 N = color I = 17;
SU  T = 9 C = 68 N = color I = 17;
SU  T = 11 C = 68 N = color I = 17;
SU  T = 13 C = 68 N = color I = 17;
SU  T = 14 C = 68 N = color I = 17;
SU  T = 15 C = 68 N = color I = 17;
SU  T = 16 C = 68 N = color I = 17;
SU  T = 18 C = 68 N = color I = 17;
SU  T = 19 C = 68 N = color I = 17;
SU  T = 21 C = 68 N = color I = 17;
SU  T = 22 C = 68 N = color I = 17;
SU  T = 23 C = 68 N = color I = 17;
SU  T = 24 C = 68 N = color I = 17;
SU  T = 25 C = 68 N = color I = 17;
SU  T = 26 C = 68 N = color I = 17;
SU  T = 28 C = 68 N = color I = 17;
SU  T = 29 C = 68 N = color I = 17;
SU  T = 30 C = 68 N = color I = 17;
SU  T = 31 C = 68 N = color I = 17;
```

```
SU  T = 32 C = 68 N = color I = 17;
SU  T = 33 C = 68 N = color I = 17;
SU  T = 34 C = 68 N = color I = 17;
SU  T = 37 C = 68 N = color I = 17;
SU  T = 38 C = 68 N = color I = 17;
SU  T = 39 C = 68 N = color I = 17;
SU  T = 40 C = 68 N = color I = 17;
SU  T = 41 C = 68 N = color I = 17;
SU  T = 42 C = 68 N = color I = 17;
SU  T = 43 C = 68 N = color I = 17;
SU  T = 44 C = 68 N = color I = 17;
SU  T = 45 C = 68 N = color I = 17;
SU  T = 52 C = 68 N = color I = 17;

SU  T = 34 C = 69 N = color I = 7;
SU  T = 39 C = 69 N = color I = 13;
SU  T = 52 C = 69 N = color I = 4;

SU  T = 1 C = 70 N = color I = 6;
SU  T = 2 C = 70 N = color I = 6;
SU  T = 3 C = 70 N = color I = 6;
SU  T = 4 C = 70 N = color I = 6;
SU  T = 5 C = 70 N = color I = 6;
SU  T = 7 C = 70 N = color I = 6;
SU  T = 9 C = 70 N = color I = 6;
SU  T = 11 C = 70 N = color I = 6;
SU  T = 13 C = 70 N = color I = 6;
SU  T = 14 C = 70 N = color I = 6;
SU  T = 15 C = 70 N = color I = 6;
SU  T = 16 C = 70 N = color I = 6;
SU  T = 18 C = 70 N = color I = 6;
SU  T = 19 C = 70 N = color I = 6;
SU  T = 21 C = 70 N = color I = 6;
SU  T = 22 C = 70 N = color I = 6;
SU  T = 24 C = 70 N = color I = 6;
SU  T = 25 C = 70 N = color I = 6;
SU  T = 26 C = 70 N = color I = 6;
SU  T = 28 C = 70 N = color I = 6;
SU  T = 29 C = 70 N = color I = 6;
SU  T = 30 C = 70 N = color I = 6;
SU  T = 31 C = 70 N = color I = 6;
SU  T = 32 C = 70 N = color I = 6;
SU  T = 33 C = 70 N = color I = 6;
SU  T = 34 C = 70 N = color I = 6;
SU  T = 37 C = 70 N = color I = 6;
SU  T = 38 C = 70 N = color I = 6;
SU  T = 39 C = 70 N = color I = 6;
SU  T = 40 C = 70 N = color I = 6;
SU  T = 41 C = 70 N = color I = 6;
SU  T = 42 C = 70 N = color I = 6;
SU  T = 43 C = 70 N = color I = 6;
SU  T = 44 C = 70 N = color I = 6;
SU  T = 45 C = 70 N = color I = 6;
SU  T = 52 C = 70 N = color I = 4;

SU  T = 34 C = 81 N = color I = 7;
SU  T = 39 C = 81 N = color I = 13;
```

```
SU  T = 52 C = 81 N = color I = 4;

SU  T = 1 C = 82 N = color I = 6;
SU  T = 2 C = 82 N = color I = 6;
SU  T = 4 C = 82 N = color I = 6;
SU  T = 5 C = 82 N = color I = 6;
SU  T = 7 C = 82 N = color I = 6;
SU  T = 9 C = 82 N = color I = 6;
SU  T = 11 C = 82 N = color I = 6;
SU  T = 13 C = 82 N = color I = 6;
SU  T = 14 C = 82 N = color I = 6;
SU  T = 15 C = 82 N = color I = 6;
SU  T = 16 C = 82 N = color I = 6;
SU  T = 19 C = 82 N = color I = 6;
SU  T = 21 C = 82 N = color I = 6;
SU  T = 22 C = 82 N = color I = 6;
SU  T = 24 C = 82 N = color I = 6;
SU  T = 25 C = 82 N = color I = 6;
SU  T = 26 C = 82 N = color I = 6;
SU  T = 28 C = 82 N = color I = 6;
SU  T = 29 C = 82 N = color I = 6;
SU  T = 31 C = 82 N = color I = 6;
SU  T = 32 C = 82 N = color I = 6;
SU  T = 33 C = 82 N = color I = 6;
SU  T = 34 C = 82 N = color I = 6;
SU  T = 37 C = 82 N = color I = 6;
SU  T = 38 C = 82 N = color I = 6;
SU  T = 39 C = 82 N = color I = 6;
SU  T = 40 C = 82 N = color I = 6;
SU  T = 41 C = 82 N = color I = 6;
SU  T = 42 C = 82 N = color I = 6;
SU  T = 43 C = 82 N = color I = 6;
SU  T = 45 C = 82 N = color I = 6;
SU  T = 52 C = 82 N = color I = 4;

SU  T = 34 C = 85 N = color I = 7;
SU  T = 39 C = 85 N = color I = 13;
SU  T = 52 C = 85 N = color I = 4;

SU  T = 34 C = 87 N = color I = 7;
SU  T = 39 C = 87 N = color I = 13;
SU  T = 52 C = 87 N = color I = 4;

SU  T = 52 C = 88 N = color I = 4;

SU  T = 1 C = 89 N = color I = 6;
SU  T = 2 C = 89 N = color I = 6;
SU  T = 4 C = 89 N = color I = 6;
SU  T = 5 C = 89 N = color I = 6;
SU  T = 7 C = 89 N = color I = 6;
SU  T = 9 C = 89 N = color I = 6;
SU  T = 11 C = 89 N = color I = 6;
SU  T = 13 C = 89 N = color I = 6;
SU  T = 14 C = 89 N = color I = 6;
SU  T = 15 C = 89 N = color I = 6;
SU  T = 16 C = 89 N = color I = 6;
SU  T = 19 C = 89 N = color I = 6;
```

```
SU  T = 21 C = 89 N = color I = 6;
SU  T = 22 C = 89 N = color I = 6;
SU  T = 24 C = 89 N = color I = 6;
SU  T = 25 C = 89 N = color I = 6;
SU  T = 26 C = 89 N = color I = 6;
SU  T = 28 C = 89 N = color I = 6;
SU  T = 29 C = 89 N = color I = 6;
SU  T = 31 C = 89 N = color I = 6;
SU  T = 32 C = 89 N = color I = 6;
SU  T = 33 C = 89 N = color I = 6;
SU  T = 34 C = 89 N = color I = 6;
SU  T = 37 C = 89 N = color I = 6;
SU  T = 38 C = 89 N = color I = 6;
SU  T = 39 C = 89 N = color I = 6;
SU  T = 40 C = 89 N = color I = 6;
SU  T = 41 C = 89 N = color I = 6;
SU  T = 42 C = 89 N = color I = 6;
SU  T = 43 C = 89 N = color I = 6;
SU  T = 45 C = 89 N = color I = 6;
SU  T = 52 C = 89 N = color I = 4;

SU  T = 1 C = 90 N = color I = 5;
SU  T = 2 C = 90 N = color I = 5;
SU  T = 3 C = 90 N = color I = 5;
SU  T = 4 C = 90 N = color I = 5;
SU  T = 5 C = 90 N = color I = 5;
SU  T = 7 C = 90 N = color I = 5;
SU  T = 8 C = 90 N = color I = 5;
SU  T = 9 C = 90 N = color I = 5;
SU  T = 11 C = 90 N = color I = 5;
SU  T = 13 C = 90 N = color I = 5;
SU  T = 14 C = 90 N = color I = 5;
SU  T = 15 C = 90 N = color I = 5;
SU  T = 16 C = 90 N = color I = 5;
SU  T = 18 C = 90 N = color I = 5;
SU  T = 19 C = 90 N = color I = 5;
SU  T = 21 C = 90 N = color I = 5;
SU  T = 22 C = 90 N = color I = 5;
SU  T = 23 C = 90 N = color I = 5;
SU  T = 24 C = 90 N = color I = 5;
SU  T = 25 C = 90 N = color I = 5;
SU  T = 26 C = 90 N = color I = 5;
SU  T = 28 C = 90 N = color I = 5;
SU  T = 29 C = 90 N = color I = 5;
SU  T = 30 C = 90 N = color I = 5;
SU  T = 31 C = 90 N = color I = 5;
SU  T = 32 C = 90 N = color I = 5;
SU  T = 33 C = 90 N = color I = 5;
SU  T = 34 C = 90 N = color I = 5;
SU  T = 37 C = 90 N = color I = 5;
SU  T = 38 C = 90 N = color I = 5;
SU  T = 39 C = 90 N = color I = 5;
SU  T = 40 C = 90 N = color I = 5;
SU  T = 41 C = 90 N = color I = 5;
SU  T = 42 C = 90 N = color I = 5;
SU  T = 43 C = 90 N = color I = 5;
SU  T = 44 C = 90 N = color I = 5;
```

```
SU  T = 45 C = 90 N = color I = 5;
SU  T = 52 C = 90 N = color I = 5;

SU  T = 52 C = 91 N = color I = 4;

SU  T = 52 C = 92 N = color I = 4;

SU  T = 34 C = 93 N = color I = 7;
SU  T = 39 C = 93 N = color I = 13;
SU  T = 52 C = 93 N = color I = 4;

SU  T = 34 C = 94 N = color I = 7;
SU  T = 39 C = 94 N = color I = 13;
SU  T = 52 C = 94 N = color I = 4;

SU  T = 1 C = 95 N = color I = 17;
SU  T = 2 C = 95 N = color I = 17;
SU  T = 3 C = 95 N = color I = 17;
SU  T = 4 C = 95 N = color I = 17;
SU  T = 5 C = 95 N = color I = 17;
SU  T = 7 C = 95 N = color I = 17;
SU  T = 8 C = 95 N = color I = 17;
SU  T = 9 C = 95 N = color I = 17;
SU  T = 11 C = 95 N = color I = 17;
SU  T = 13 C = 95 N = color I = 17;
SU  T = 14 C = 95 N = color I = 17;
SU  T = 15 C = 95 N = color I = 17;
SU  T = 16 C = 95 N = color I = 17;
SU  T = 18 C = 95 N = color I = 17;
SU  T = 19 C = 95 N = color I = 17;
SU  T = 21 C = 95 N = color I = 17;
SU  T = 22 C = 95 N = color I = 17;
SU  T = 23 C = 95 N = color I = 17;
SU  T = 24 C = 95 N = color I = 17;
SU  T = 25 C = 95 N = color I = 17;
SU  T = 26 C = 95 N = color I = 17;
SU  T = 28 C = 95 N = color I = 17;
SU  T = 29 C = 95 N = color I = 17;
SU  T = 30 C = 95 N = color I = 17;
SU  T = 31 C = 95 N = color I = 17;
SU  T = 32 C = 95 N = color I = 17;
SU  T = 33 C = 95 N = color I = 17;
SU  T = 34 C = 95 N = color I = 17;
SU  T = 37 C = 95 N = color I = 17;
SU  T = 38 C = 95 N = color I = 17;
SU  T = 39 C = 95 N = color I = 17;
SU  T = 40 C = 95 N = color I = 17;
SU  T = 41 C = 95 N = color I = 17;
SU  T = 42 C = 95 N = color I = 17;
SU  T = 43 C = 95 N = color I = 17;
SU  T = 44 C = 95 N = color I = 17;
SU  T = 45 C = 95 N = color I = 17;
SU  T = 52 C = 95 N = color I = 17;

SU  T = 34 C = 98 N = color I = 7;
SU  T = 39 C = 98 N = color I = 13;
SU  T = 52 C = 98 N = color I = 4;
```

```
SU  T = 52 C = 99 N = color I = 4;

SU  T = 1 C = 100 N = color I = 6;
SU  T = 2 C = 100 N = color I = 6;
SU  T = 4 C = 100 N = color I = 6;
SU  T = 5 C = 100 N = color I = 6;
SU  T = 7 C = 100 N = color I = 6;
SU  T = 9 C = 100 N = color I = 6;
SU  T = 11 C = 100 N = color I = 6;
SU  T = 13 C = 100 N = color I = 6;
SU  T = 14 C = 100 N = color I = 6;
SU  T = 15 C = 100 N = color I = 6;
SU  T = 16 C = 100 N = color I = 6;
SU  T = 19 C = 100 N = color I = 6;
SU  T = 21 C = 100 N = color I = 6;
SU  T = 22 C = 100 N = color I = 6;
SU  T = 24 C = 100 N = color I = 6;
SU  T = 25 C = 100 N = color I = 6;
SU  T = 26 C = 100 N = color I = 6;
SU  T = 28 C = 100 N = color I = 6;
SU  T = 29 C = 100 N = color I = 6;
SU  T = 31 C = 100 N = color I = 6;
SU  T = 32 C = 100 N = color I = 6;
SU  T = 33 C = 100 N = color I = 6;
SU  T = 34 C = 100 N = color I = 6;
SU  T = 37 C = 100 N = color I = 6;
SU  T = 38 C = 100 N = color I = 6;
SU  T = 39 C = 100 N = color I = 6;
SU  T = 40 C = 100 N = color I = 6;
SU  T = 41 C = 100 N = color I = 6;
SU  T = 42 C = 100 N = color I = 6;
SU  T = 43 C = 100 N = color I = 6;
SU  T = 45 C = 100 N = color I = 6;
SU  T = 52 C = 100 N = color I = 4;

SU  T = 52 C = 101 N = color I = 4;

SU  C = 102 N = color I = 4;
SU  T = 1 C = 102 N = color I = 5;
SU  T = 2 C = 102 N = color I = 5;
SU  T = 5 C = 102 N = color I = 5;
SU  T = 7 C = 102 N = color I = 5;
SU  T = 13 C = 102 N = color I = 5;
SU  T = 14 C = 102 N = color I = 5;
SU  T = 15 C = 102 N = color I = 5;
SU  T = 19 C = 102 N = color I = 5;
SU  T = 24 C = 102 N = color I = 5;
SU  T = 25 C = 102 N = color I = 5;
SU  T = 29 C = 102 N = color I = 5;
SU  T = 32 C = 102 N = color I = 5;
SU  T = 34 C = 102 N = color I = 7;
SU  T = 37 C = 102 N = color I = 5;
SU  T = 39 C = 102 N = color I = 13;
SU  T = 41 C = 102 N = color I = 5;
SU  T = 43 C = 102 N = color I = 5;
SU  T = 45 C = 102 N = color I = 5;
```

```
SU  T = 52 C = 102 N = color I = 4;

SU  T = 34 C = 105 N = color I = 7;
SU  T = 39 C = 105 N = color I = 13;
SU  T = 52 C = 105 N = color I = 4;

SU  T = 52 C = 109 N = color I = 4;

SU  T = 1 C = 111 N = color I = 6;
SU  T = 2 C = 111 N = color I = 6;
SU  T = 4 C = 111 N = color I = 6;
SU  T = 5 C = 111 N = color I = 6;
SU  T = 7 C = 111 N = color I = 6;
SU  T = 9 C = 111 N = color I = 6;
SU  T = 11 C = 111 N = color I = 6;
SU  T = 13 C = 111 N = color I = 6;
SU  T = 14 C = 111 N = color I = 6;
SU  T = 15 C = 111 N = color I = 6;
SU  T = 16 C = 111 N = color I = 6;
SU  T = 19 C = 111 N = color I = 6;
SU  T = 21 C = 111 N = color I = 6;
SU  T = 22 C = 111 N = color I = 6;
SU  T = 24 C = 111 N = color I = 6;
SU  T = 25 C = 111 N = color I = 6;
SU  T = 26 C = 111 N = color I = 6;
SU  T = 28 C = 111 N = color I = 6;
SU  T = 29 C = 111 N = color I = 6;
SU  T = 31 C = 111 N = color I = 6;
SU  T = 32 C = 111 N = color I = 6;
SU  T = 33 C = 111 N = color I = 6;
SU  T = 34 C = 111 N = color I = 6;
SU  T = 37 C = 111 N = color I = 6;
SU  T = 38 C = 111 N = color I = 6;
SU  T = 39 C = 111 N = color I = 6;
SU  T = 40 C = 111 N = color I = 6;
SU  T = 41 C = 111 N = color I = 6;
SU  T = 42 C = 111 N = color I = 6;
SU  T = 43 C = 111 N = color I = 6;
SU  T = 45 C = 111 N = color I = 6;
SU  T = 52 C = 111 N = color I = 4;

SU  T = 1 C = 112 N = color I = 6;
SU  T = 2 C = 112 N = color I = 6;
SU  T = 4 C = 112 N = color I = 6;
SU  T = 5 C = 112 N = color I = 6;
SU  T = 7 C = 112 N = color I = 6;
SU  T = 9 C = 112 N = color I = 6;
SU  T = 11 C = 112 N = color I = 6;
SU  T = 13 C = 112 N = color I = 6;
SU  T = 14 C = 112 N = color I = 6;
SU  T = 15 C = 112 N = color I = 6;
SU  T = 16 C = 112 N = color I = 6;
SU  T = 19 C = 112 N = color I = 6;
SU  T = 21 C = 112 N = color I = 6;
SU  T = 22 C = 112 N = color I = 6;
SU  T = 24 C = 112 N = color I = 6;
SU  T = 25 C = 112 N = color I = 6;
```

```
      SU  T = 26 C = 112 N = color I = 6;
      SU  T = 28 C = 112 N = color I = 6;
      SU  T = 29 C = 112 N = color I = 6;
      SU  T = 31 C = 112 N = color I = 6;
      SU  T = 32 C = 112 N = color I = 6;
      SU  T = 33 C = 112 N = color I = 6;
      SU  T = 34 C = 112 N = color I = 6;
      SU  T = 37 C = 112 N = color I = 6;
      SU  T = 38 C = 112 N = color I = 6;
      SU  T = 39 C = 112 N = color I = 6;
      SU  T = 40 C = 112 N = color I = 6;
      SU  T = 41 C = 112 N = color I = 6;
      SU  T = 42 C = 112 N = color I = 6;
      SU  T = 43 C = 112 N = color I = 6;
      SU  T = 45 C = 112 N = color I = 6;
      SU  T = 52 C = 112 N = color I = 4;

      SU  T = 34 C = 113 N = color I = 7;
      SU  T = 39 C = 113 N = color I = 13;
      SU  T = 52 C = 113 N = color I = 4;

      SU  T = 34 C = 114 N = color I = 7;
      SU  T = 39 C = 114 N = color I = 13;
      SU  T = 52 C = 114 N = color I = 4;

      SU  T = 52 C = 115 N = color I = 4;

      SU  T = 34 C = 116 N = color I = 7;
      SU  T = 39 C = 116 N = color I = 13;
      SU  T = 52 C = 116 N = color I = 4;

      SU  T = 52 C = 117 N = color I = 4;

      SU  T = 34 C = 118 N = color I = 7;
      SU  T = 39 C = 118 N = color I = 13;
      SU  T = 52 C = 118 N = color I = 4;

      SU  T = 34 C = 119 N = color I = 7;
      SU  T = 39 C = 119 N = color I = 13;
      SU  T = 52 C = 119 N = color I = 4;

END;

BEGIN MESQUITECHARMODELS;
      CharModel my_model (Mk1) =
             rate 1.0 maxState 1;

      ProbModelSet * UNTITLED   =  'Mk1 (est.)':  1- 121;
END;

Begin MESQUITE;
            MESQUITESCRIPTVERSION 2;
            TITLE AUTO;
            tell ProjectCoordinator;
            timeSaved 1675937838413;
            getEmployee #mesquite.minimal.ManageTaxa.ManageTaxa;
            tell It;
```

```
				setID 0 4597810923714763460;
				tell It;
					setSelected  52;
					setDefaultOrder  302 36 292 264 302 101 315 24 43 
96 47 148 25 40 33 49 97 297 302 52 272 46 306 29 21 51 63 265 20 299 46 
333 43 308 60 150 299 48 40 50 329 294 316 298 12 158 186 156 122 197 159 
326;
					attachments ;
				endTell;
			endTell;
			getEmployee 
#mesquite.charMatrices.ManageCharacters.ManageCharacters;
			tell It;
				setID 0 3537645405170558061;
				tell It;
					setDefaultOrder  0 1 3 271 3 8 9 10 29 14 193 37 
137 38 20 21 22 30 25 270 27 5 31 238 30 124 266 203 33 37 39 40 41 231 
71 136 44 252 262 61 207 47 53 54 57 73 62 63 177 64 67 65 67 34 72 37 
140 44 76 78 90 81 82 83 84 85 86 88 208 206 93 94 97 99 100 101 103 104 
106 107 48 204 111 112 117 114 107 140 158 135 149 144 10 15 14 144 145 
185 148 227 188 192 123 120 186 153 154 155 199 122 50 57 210 162 163 157 
173 159 16 169 177;
					attachments ;
				endTell;
				mqVersion 370;
				checksumv 0 3 2704420853 null  getNumChars 121 numChars 
121 getNumTaxa 52 numTaxa 52   short true   bits 63   states 63 
sumSquaresStatesOnly 24534.0 sumSquares 24534.0 longCompressibleToShort 
false usingShortMatrix true   NumFiles 1 NumMatrices 1;
				mqVersion;
			endTell;
			getWindow;
			tell It;
				suppress;
				setResourcesState false false 52;
				setPopoutState 400;
				setExplanationSize 0;
				setAnnotationSize 0;
				setFontIncAnnot 0;
				setFontIncExp 0;
				setSize 958 942;
				setLocation 952 0;
				setFont SanSerif;
				setFontSize 10;
				getToolPalette;
				tell It;
				endTell;
				desuppress;
			endTell;
			getEmployee 
#mesquite.charMatrices.BasicDataWindowCoord.BasicDataWindowCoord;
			tell It;
				showDataWindow #3537645405170558061 
#mesquite.charMatrices.BasicDataWindowMaker.BasicDataWindowMaker;
				tell It;
					getWindow;
					tell It;
```

```
					getTable;
					tell It;
						rowNamesWidth 144;
					endTell;
					setExplanationSize 30;
					setAnnotationSize 60;
					setFontIncAnnot 0;
					setFontIncExp 0;
					setSize 906 830;
					setLocation 952 0;
					setFont SansSerif;
					setFontSize 10;
					getToolPalette;
					tell It;
						setTool
mesquite.charMatrices.BasicDataWindowMaker.BasicDataWindow.ibeam;
					endTell;
					setActive;
					setTool
mesquite.charMatrices.BasicDataWindowMaker.BasicDataWindow.ibeam;
					colorCells
#mesquite.charMatrices.ColorByState.ColorByState;
				tell It;
					setStateLimit 9;
					toggleUniformMaximum on;
				endTell;
					colorRowNames
#mesquite.charMatrices.ColorCells.ColorCells;
				tell It;
					setColor Red;
					removeColor off;
				endTell;
					colorColumnNames
#mesquite.charMatrices.ColorCells.ColorCells;
				tell It;
					setColor Red;
					removeColor off;
				endTell;
					colorText
#mesquite.charMatrices.NoColor.NoColor;
					setBackground White;
					toggleShowNames off;
					toggleShowTaxonNames on;
					toggleTight off;
					toggleThinRows off;
					toggleShowChanges on;
					toggleSeparateLines off;
					toggleShowStates on;
					toggleReduceCellBorders off;
					toggleAutoWCharNames on;
					toggleAutoTaxonNames off;
					toggleShowDefaultCharNames off;
					toggleConstrainCW on;
					toggleBirdsEye off;
					toggleColorOnlyTaxonNames off;
					toggleShowPaleGrid off;
					toggleShowPaleCellColors off;
```

```
					toggleShowPaleExcluded off;
					togglePaleInapplicable on;
					togglePaleMissing off;
					toggleShowBoldCellText off;
					toggleAllowAutosize on;
					toggleColorsPanel off;
					toggleDiagonal on;
					setDiagonalHeight 80;
					toggleLinkedScrolling on;
					toggleScrollLinkedTables off;
					getInfoPanel;
					tell It;
						btspOpen false;
						apOpen false;
						fpOpen true;
					endTell;
					toggleInfoPanel on;
				endTell;
				showWindow;
				getWindow;
				tell It;
					forceAutosize;
				endTell;
				getEmployee
#mesquite.charMatrices.AlterData.AlterData;
				tell It;
					toggleBySubmenus off;
				endTell;
				getEmployee
#mesquite.categ.StateNamesEditor.StateNamesEditor;
				tell It;
					makeWindow;
					tell It;
						getTable;
						tell It;
							rowNamesWidth 318;
						endTell;
						setExplanationSize 30;
						setAnnotationSize 20;
						setFontIncAnnot 0;
						setFontIncExp 0;
						setSize 906 870;
						setLocation 952 0;
						setFont SanSerif;
						setFontSize 10;
						getToolPalette;
						tell It;
							setTool
mesquite.categ.StateNamesEditor.StateNamesWindow.ibeam;
						endTell;
						rowsAreCharacters on;
						toggleConstrainChar on;
						toggleConstrainCharNum 3;
						togglePanel off;
						toggleSummaryPanel off;
					endTell;
					showWindow;
```

```
                    endTell;
                    getEmployee
#mesquite.categ.StateNamesStrip.StateNamesStrip;
                    tell It;
                         showStrip off;
                    endTell;
                    getEmployee
#mesquite.charMatrices.AnnotPanel.AnnotPanel;
                    tell It;
                         togglePanel off;
                    endTell;
                    getEmployee
#mesquite.charMatrices.CharReferenceStrip.CharReferenceStrip;
                    tell It;
                         showStrip off;
                    endTell;
                    getEmployee
#mesquite.charMatrices.QuickKeySelector.QuickKeySelector;
                    tell It;
                         autotabOff;
                    endTell;
                    getEmployee
#mesquite.charMatrices.SelSummaryStrip.SelSummaryStrip;
                    tell It;
                         showStrip off;
                    endTell;
                    getEmployee
#mesquite.categ.SmallStateNamesEditor.SmallStateNamesEditor;
                    tell It;
                         panelOpen true;
                    endTell;
               endTell;
          endTell;
          endTell;
end;
```
