# Supplementary material for: The oldest case of paedomorphosis in rove beetles and description of a new genus of Paederinae from Cretaceous amber (Coleoptera: Staphylinidae)
Source: Sci Rep. 2023 Mar 31;13:5317. doi: 10.1038/s41598-023-32446-2 (PMC10066364; doi:10.1038/s41598-023-32446-2)
Supplement: Supplementary file 6 — Supplementary Information 6. [file 41598_2023_32446_MOESM6_ESM.docx]

### Supplement 4. GenBank accession numbers of all sequences. The novel sequences are marked in red. Missing data are indicated with a dash ‘-’. Taxa used only for morphological analysis are also mentioned in the table. Taxa that were sequenced for the first time are marked with *.

**FMNH** – Field Museum of Natural History, Chicago

**MUSM** – Museo de Historia Natural de la Universidad Nacional Mayor de San Marcos

**NHMD** – Natural History Museum of Denmark, Copenhagen (formerly ‘ZMUC’)

**UTCI** – University of Tennessee at Chattanooga

|  | | **Genbank accession numbers** | | | | | |
| --- | --- | --- | --- | --- | --- | --- | --- |
| **Taxon and placement before present study** | **Collection, Specimen #** | **28S** | **ArgK** | **CADA+CADC** | **COI** | **TP** | **Wg** |
| **Tachyporinae** | | | | | | | |
| *Tachyporus hypnorum* (Fabricius, 1775) | NHMD, NHMD_DZ-7.0 | MN073526 | MN194113 | MN194127 (A) | MN194140 | MN194152 | MN194165 |
| **Mycetoporinae** | | | | | | | |
| *Lordithon* cf. *lunulatus* (Linnaeus, 1760) | NHMD, NHMD_DZ-10.6 | MN165645 | MN251775 | MN256442 (A) | MN264591 | MN284886 | MN328041 |
| **Oxyporinae** | | | | | | | |
| *Oxyporus femoralis* Gravenhorst, 1802 | UTCI, SC-0192 | KT149213 | KT021925 | KT000243 | KT021973 | KT022020 | KT022070 |
| **Staphylininae** | | | | | | | |
| **Staphylinini** | | | | | | | |
| *Quedius molochinus* (Gravenhorst, 1806) | NHMD, QUmol01 | GU377348 | KF178783 | KF178814 | GU377396 | GU377447 | GU377498 |
| **Xantholininae** |  |  |  |  |  |  |  |
| **Diochini** | | | | | | | |
| *Diochus* sp. Erichson, 1839 (Laos) | NHMD, DIOsp01 | GU377325 | KT021922 | KT000260 (C) | GU377370 | GU377420 | GU377471 |
| **Xantholinini** |  |  |  |  |  |  |  |
| *Thyreocephalus annulatus* (Fauvel, 1895) | NHMD, 00046195 | KR559822 | KT021878 | KT000196 | KT021954 | KT021986 | KT022069 |
| **Paederinae** | | | | | | | |
| **Pinophilini** | | | | | | | |
| Proccirina | | | | | | | |
| *Oedichirus* sp. Erichson, 1839 (Australia) | NHMD, zmuc00046231 | MN073519 | KR259669 | MT809527(A) /  KR259788 (C) | KR259767 | KR259736 | KR259704 |
| Pinophilina | | | | | | | |
| *Pinophilus* sp. Gravenhorst, 1802 (Australia) | NHMD, zmuc00046227 | MT801125 | KR259681 | MN194125 (A)  /  KF178807 (C) | GU377362 | GU377412 | GU377463 |
| **Paederini** | | | | | | | |
| Cryptobiina | | | | | | | |
| *Ochthephilum fracticorne* (Paykull, 1800) | NHMD, zmuc00046225 & NHMD_AB-55 | MT801119 | KR259663 | MT809526 (A) /  KR259793 (C) | KR259764 | KR259731 | KR259714 |
| Dicaxina | | | | | | | |
| *Hyperomma bicoloripes* Schomann, 2014 | NHMD, zmuc00046255 & DDM1149 | KJ844900 | KR259684 | MT809522(A)/  KR259786 (C) | KR259773 | KR259734 | KR259700 |
| Dolicaonina | | | | | | | |
| *Leptobium densiventris* (Fauvel, 1875) | NHMD, zmuc00046226 | - | KR259683 | KR259785 (C) | KR259763 | KR259726 | KR259708 |
| Paederina | | | | | | | |
| *Paederus littoralis* Gravenhorst, 1802 | NHMD,  NHMD_DZ-20.5 & DDM1166 | MT801122 | - | KJ845307 (A) / MT809528 (C) | MT792667 | MT809547 | MT809573 |
| **Lathrobiini** | | | | | | | |
| Astenina | | | | | | | |
| *Astenus* sp. Dejean, 1833 (USA) | UTCI, SC-0103 | GU377318 | KF178775 | KF178807 | GU377362 | GU377412 | GU377463 |
| *Astenus pulchellus* (Heer, 1839) | NHMD, zmuc00046222 | MT801099 | KR259662 | KR259774 (C) | KR259751 | KR259718 | KR259689 |
| Cylindroxystina | | | | | | | |
| *Cylindroxystus* *longulus* Bierig, 1943 (CostaRica) | NHMD, NHMD_DZ-5.3 | MT801105 | MT801161 | MT809502 | MT792653 | - | MT809557 |
| *Neolindus* sp. Scheerpeltz, 1933 (Peru) | MUSM,  NHMD_DZ-NEO | MT801117 | - | MT809524 (C) | MT792664 | MT809543 | MT809569 |
| Echiasterina | | | | | | | |
| *Echiaster* sp. Erichson, 1839 (Costa Rica) | NHMD, NHMD_DZ-8.0 | MN073512 | MN158714 | MN194117 (A) | MN194132 | MN194145 | MN194157 |
| *Haplonazeris* sp. Coiffait & Sáiz, 1968 (Chile)* | FMNH  FMNH_AT- | - | OP454304 | - / - | - | - | OP454301 |
| *Ronetus* sp. Blackwelder, 1943 (Mexico) | NHMD,  NHMD_DZ-23.3 | MT801128 | MT801175 | MT809529 (A) | MT792671 | MT809551 | MT809577 |
| Lathrobiina | | | | | | | |
| *Domene* sp. Fauvel, 1873 (Czech Republic) | NHMD, NHMD_DZ-4.1 | MN073511 | MN158713 | MN194116 (A) / MT809520 (C) | MN194131 | MN194144 | MN194156 |
| *Dysanabatium jacobsoni* Bernhauer, 1915 | NHMD, NHMD_AB-1 | MT801108 | MT801163 | MT809503 | MT792655 | MT809537 | MT809560 |
| *Enallagium* sp. Bernhauer, 19l5 (Myanmar) | NHMD, NHMD_AB-23 | MT801110 | MT801165 | MT809504 | MT792657 | MT809539 | MT809562 |
| *Lathrobium brunnipes* (Fabricius, 1792) | NHMD, zmuc00046221 | MN073515 | KR259675 | MT809523 (A) /  KR259779 (C) | KR259757 | KR259722 | KR259709 |
| *Lobrathium candicum* Bordoni, 2009 | NHMD, NHMD_AB-38 | MT801115 | MT801169 | MT809507 | MT792662 | - | MT809567 |
| *Notobium* sp. Solsky, 1864 (Australia) | NHMD, zmuc00046223 | MN073524 | KR259660 | MT809525 (A) /  KR259781 (C) | KR259756 | KR259724 | KR259702 |
| *Tetartopeus* sp. Czwalina, 1888 (Czech Republic) | NHMD,  NHMD_DZ-6.1 | MN073527 | MN194114 | MN194128 (A) / MT809533 (C) | MN194141 | MN194153 | MN194166 |
| Medonina | | | | | | | |
| *Achenomorphus* sp. Motschulsky, 1858 (Costa Rica) | NHMD, NHMD_DZ-9.0 | MN073509 | MT801157 | MN194115 (A) / MT809516 (C) | MN194130 | MN194143 | MN194155 |
| *Lithocharis nigriceps* Kraatz, 1859 | NHMD, NHMD_DZ-4.2 | MT801114 | MT801168 | MT809506 | MT792661 | MT809541 | MT809566 |
| *Medon apicalis* (Kraatz, 1857) | NHMD, zmuc00046233 | MN073517 | KR259674 | KR259775 (C) | KR259753 | KR259719 | KR259690 |
| *Neosclerus* sp. Cameron, 1924 (Myanmar) | NHMD, NHMD_AB-34 | MT801118 | MT801171 | MT809509 | MT792665 | MT809544 | MT809570 |
| *Pseudomedon obscurellus* (Erichson, 1840) | NHMD,  NHMD_DZ-14.5 | MT801127 | MT801174 | MT809512 | MT792670 | MT809550 | MT809576 |
| *Sciocharis* sp. Lynch, 1885 (Mexico)* | NHMD  NHMD_AB- | OP422452 | OP454305 | OP454309 (A)/ OP454311 (C) | OP425398 | OP454307 | OP454302 |
| *Scioporus* sp. Sharp, 1886 (Costa Rica)* | NHMD  NHMD_AT- | OP422453 | OP454306 | OP454310 (A)/ OP454312 (C) | OP425399 | OP454308 | OP454303 |
| *Sunius melanocephalus* (Fabricius, 1792) | NHMD, NHMD_DZ-5.2 | MN073525 | MN194112 | MN194126 (A) / MT809532 (C) | MN194139 | MN194151 | MN194164 |
| *Suniotrichus* sp. Sharp, 1886 (Mexico) | NHMD,  NHMD_DZ-21.3 | MT801130 | MT801177 | MT809514 | MT792673 | MT809552 | MT809579 |
| *Thinocharis* sp. Kraatz, 1859 (Laos) | NHMD, NHMD_AB-4 | MT801132 | MT801179 | MT809515 | MT792675 | MT809554 | MT809581 |
| Scopaeina | | | | | | | |
| *Orus* sp. Casey, 1885 (Mexico) | NHMD, NHMD_DZ-20.6 | MT801120 | - | - | MT792666 | MT809545 | MT809571 |
| *Scopaeus* sp. Erichson, 1839 (Australia) | NHMD, zmuc00046219 & DDM0325 | MN073523 | KR259680 | KJ845232 (A) /  KR259778 (C) | KR259759 | KR259725 | KR259713 |
| Stilicina | | | | | | | |
| *Eustilicus* sp. Sharp, 1886 (Costa Rica) | NHMD, NHMD_DZ-8.4 | MT801111 | MT801166 | MT809505 | MT792658 | MT809540 | MT809563 |
| *Rugilus rufipes* Germar, 1836 | NHMD, zmuc00046228 & NHMD_JK-VII86.Rr | MN073522 | KR259673 | MT809530 (A) / KR259776 (C) | KR259754 | KR259720 | KR259691 |
| *Stilicoderus* sp. Sharp, 1889 (Australia) | NHMD,  NHMD_DZ-24.6 | MT801129 | MT801176 | MT809513 | MT792672 | - | MT809578 |
| Stilicopsina | | | | | | | |
| *Dibelonetes* sp. Sahlberg, 1847 (Australia) | NHMD, zmuc00046229 | MN073510 | KR259672 | MT809518 (A) /  KR259777 (C) | KR259752 | KR259721 | KR259692 |
| *Stilicopsis* sp. Sachse, 1852 (Mexico) | NHMD,  NHMD_DZ-20.3 | MN165651 | MN251780 | MT809531 (A) / MN256445 (C) | MN264597 | MN284890 | MN328046 |
| Lathrobiini insertae sedis | | | | | | | |
| Medonina gen. and sp. indet. (Russia) | NHMD, NHMD_DZ-2.2 | MT801116 | MT801170 | MT809508 | MT792663 | MT809542 | MT809568 |
| *Pseudolathra* sp. Casey, 1905 (Laos) | NHMD, PAE01 | GU377340 | KF178776 | KF178808 (A) | GU377386 | GU377437 | GU377488 |
