# Supplementary material for: The oldest case of paedomorphosis in rove beetles and description of a new genus of Paederinae from Cretaceous amber (Coleoptera: Staphylinidae)
Source: Sci Rep. 2023 Mar 31;13:5317. doi: 10.1038/s41598-023-32446-2 (PMC10066364; doi:10.1038/s41598-023-32446-2)
Supplement: Supplementary file 7 — Supplementary Information 7. [file 41598_2023_32446_MOESM7_ESM.pdf]

>Achenomorphus
GGGAGAAGCCCAGCACTGAATCCCGTGGCCGAACCGGGAAATGTAGTGTTTGGGAGGATCCAATATCCACCGT
GCGACGC
GTCCAAGTCCTTCTTGAACGGGGCCACATACCCATAGAGGGTGCCAGGCCCGGTAGCTGGAGGATCTCTCCTT
AGAGTCG
GGTTGCTTGAGAGTGCAGCCCTAAGTGGGTGGTAAACTCCATCTAAGGCTAAATATGACCACGAGACCGATAG
CGAACAA
GTACCGTGAGGGAAAGTTGAAAAGAACTTTGAAGAGAGAGTTCAATAGTACGTGAAACCGTTCAGGGGTAAAC
CTGAGAA
ACCCGAAAGGTCGAATGGGGAGATTCAGCGTGTCTCGTTTTTGGTTGCGTGACGATGGTGTTCGCACCGGGCT
GCGCCTT
CCGAATCCGTAACCGGTGACGAACTCGTGCACTTCTCCCCTAGTAGGACGTCGCGACCCGTTGGGTGCCGGTC
TAAGGCC
GACGGTGGAGACTTGGGGTCCCGGCCGGCCCGCTCGACGGTAAGACGGAGGCGTGGGGTCGCTACGTTAGCGT
CCGGCCC
GTCACAAGTACGGGCGACTCGGATGTCGGACCTGTGTGCCGACCCCGAGATCGCCGACTGTTGGTGACGGTGT
CCTCGGA
CAGACTACACGCCGGTCGGCGACGCTCTAGCTTTGGGTTTTCAGGACCCGTCTTGAAACACGGACCAAGGAGT
CTAGCAT
GTGCGCGAGTCATTGGGACCGCATCTAAACCTAATGGCAAAATGAAAGTGAAGGCGTGCCGAGGGAGGATGGG
TCGGGGG
GCGTCTCGTTCTCATCGCGAGATGAGGCGCACCCAGAGCGTACACGCGCTTACACCGTATTCGCTGATCTGTT
CGA-CCC
CATCATCGAAGACTACCATGGTGGATTCAAGAAGACCGACAAGCACCCCCCCGCGAACTGGGGTGATGTAAAC
ACCTTCG
CTAATCTCGACCCTGCCGGTGAATACGTCGTATCCACCCGCGTCCGTTGCGGCCGCTCCATGGAGGGCTACCC
CTTCAAC
CCGTGCTTAACCGAGGAGCAGTACAAGGAGATGGAGAGCAAAGTGTCCAGCACTTTGTCCGGCTTGGAAGGCG
AGCTCAA
GGGTACTTTCTACCCCTTGACCGGAATGGACAAGGACACCCAGCAGAAGCTCATCGACGACCACTTCTTGTTC
AAGGAGG
GCGACCGCTTCCTGCAGACCGCCAACGCCTGCCGCTTCTGGCCATCCGGCCGTGGTATCTACCACAACGACAA
CAAGACC
TTCTTGGTTTGGTGCAACGAAGAGGACCATCTCCGCATCATCTCCATGCAGATGGGCGGCGACTTGGGCGAGG
TATACCG
CCGCCTGGTGACCGCCGTCAACGAGATCGAGAAACGTGTACCGTTCTCCCACAATGACAGATTGGGTTTCCTC
ACCTTCT
GCCCAACCAACTTGGGAACTACTGTACGTGCCTCTGTACACATCAAAGTACCTAAGCTCGCCGCCAACAAAGC
CAAACTC
GACGAGGTCGCCGGCAAATACAACTTGCAGGTACGCGGCACTCGCGGTGTTCCTAAAAAAGTGCTAATAATTG
GATCAGG
GGGATTATCAATAGGCCAAGCAGGAGAATTCGATTATTCGGGCTCGCAAGCCATTAAAGCTYTACACGAAGAA
AACATCC
AAACAGTATTGATCAATCCAAACATTGCAACTGTACAAACATCAAAGGGTTTAGCAGACAAAGTTTATTTCTT
ACCTCTA
GTGCCTGAATTTGTAGAACAAGTCATACGAGTGGAACGTCCTGGCGGCGTATTGTTAACGTTCGGCGGACAAA
CAGGGTT
AAATTGTGGTGTGGAACTACAAAAGGCTGGTGTATTCGAAAAATACAATGTTAAAATATTGGGGACACCAATA
CAAGCTA
TTATAGATACAGAAGATAGGAAAATATTTAGTGAAAGGATTGCTATGATTGGTGAAAAAGTAGCTCCCAGCAT
GGCTGCT
TATTCAGTGCAGGAAGCATTGGAAGCAGCTGAATTGCTGGGGTACCCTGTGATGGCTAGAGCAGCGTTTTCTT
TAGGTGG
ATTAGGATCTGGGTTTGCCAACACGGCAGAGGAACTAAAATTACTCGCTCAACAAGCTTTAGCTCATTCTAAT
CAGTTAA
TTATTGATAAGTCTTTGAAAGGATGGAAAGAAGTGGAATATGAAGTTGTCAGAGATGCATATCCGTACCTTAA
GAAAGTT

AATGATGATGAACTGAAAGAACCTACAGATAAAAGAATGTTTGTGCTAGCAGCAGCCTTAAAAAGCGGTTACA
GTGTAGA
CAAATTATATGATTT-
AACAAAAAATAGATCGTTGGTTCTTGCAGAAGATGAAAAATATTGTAGATTACAATACACTTCTC
GAATCCATGCAACAAAATAAAATACAAAATACTGCGTACAATTACAAATTATTATTGAAAGCGAAACAAATTG
GTTTTAG
TGATAAACAAATTGCTGTTGCTATTAAAAGCACTGAGCTTGCAGTTAGAAAACAAAGACAAGACTTTGGTATT
ACACCGT
TTGTTAAACAAATTGATACAGTGGCTGCTGAATGGCCAGCAACAACTAATTATCTGTATTTAACGTACAATGC
AGATAGT
CATGATTTAACTTACAATGAAGAACATATTATGGTGATTGGTTCAGGCGTTTATAGAATTGGTAGCTCTGTTG
AATTTGA
TTGGTGTGCAGTTGGTTGTTTGAGGGAACTTAGAAAATTAAATAAAAAAACAATAATGGTTAATTACAATCCT
GAAACAG
TGAGTACGGATTATGATATGTCAGATAGATTGTATTTTGAGGAAATATCATTTGAAGTTGTAATGGATGAAGT
CTATATT
TTAATTTTACCTGGATTTGGAATAATTTCTCATATTATTTCTCAAGCTAGAGGAAAAAAAGAAACATTTGGAT
CATTAGG
AATAATTTATGCTATAATAGCTATTGGATTATTGGGATTTGTAGTTTGAGCTCATCATATATTTACAGTTGGA
ATAGATG
TAGATACACGAGCTTATTTTACCTCTGCTACTATAATTATTGCGGTTCCTACTGGAATTAAAATTTTTAGATG
ATTAGCT
ACTTTACATGGAACACAAATTAAATTTACTCCTCCAATATTATGATCATTGGGATTTGTTTTTCTATTTACAA
TTGGAGG
ATTAACTGGAGTTATTCTTGCTAATTCTTCTATTGATATTATTCTTCATGATACTTATTATGTTGTTGCCCAT
TTTCATT
ATGTATTATCTATAGGAGCAGTATTTGCTATTATAGCTGGTCTAGTACAATGATTTCCCTTATTTACAGGATT
AGTAATA
AATGAATATTTATTAAAAGTTCAATTTTTTGTTATATTTATTGGAGTAAATATAACATTTTTCCCTCAACATT
TTCTAGG
ACTAGCAGGGATACCTCGACGATATTCTGATTATCCGGATGCATACACTCCATGGAATGTAATTTCTTCAATT
GGAAGAA
TAATTTCAATAATAAGAATTTTTTTATTATTATTTATTATTTGAGAAAGATTTGTTTCTATACGAATAAATAT
CTCAGCT
AAAAATTTTTCAACTTCAATTGAATGATTACAACTATTTCCCCCAGCTGAACATAGATATTCAGAATTACCTA
TATGAAC
A-----------------ATTTGATTTCCTTGGT-
AAGGATTCCATTAGATACTACAATGAAGTACCTGTAGAAAAACGT
GTCTTCAAGAACCTTCAATTATTTATGGATAATAAATCGCCTGGAGATGATCTGTTCGACAGATTGAACACAG
CTGTGAT
GAACAAACATTTAAACGAGTTAATGGAGGGTCTCACTGCCAAGGTGTTTCGTACTTATAACGCTTCTTGGACT
TTACAGC
AACAACTCGATAAGTTGACCAATCCTGATGATTCCATATCTGAAAAAATTTTATCATATAACCGTGCCAATAG
AGCAGTA
GCAATACTTTGTAACCATCAACGTGCTGTACCTAAAGGCCACCAAAAGTCCATGGAGAAACTCAAAGAAAAGA
TCGAAAC
TAAAAAGGACTCCATTAAAGATGCTGAACGACAAGTAAAAGATGCACAGAGAGATGCAAAGCA---
TGGCAGCGTAAAGG
AGAAGCAGATTTATGAAAAGAAAAAGAAAATGTTGGAGAGATTAAGAGAGCAATTGGCTAAATTGGAGATCCA
AGAAACT
GACCGTGACGAAAATAAAACTATTGCCCTAGGTACGTCCAAACTGAATTATTTGGACCCTAGAATTTCTGTAG
CATGGTG
TAAGAAGTTTGGTGTGCCCATTGAAAAAATCTACAAC---------
TGGATGCGGCTGCCCCCGTTCCGCGTGATCGGCG
ACCACCTGAAGGACCGCTTCGACGGGGCGTCGCGCGTCATGCTCAGCAACTCGGCCAGTTCCCGCGG------
------G

AACGC---GAACCGTC---
CCAAACAGGACAAACTGTCCAACAGCATCGCGTCGAACAGCATACACAGCAAGAGGGAGAA
CAGGCCCAGGAAGTACAAGTACGGGTTCCAGTTGAAACCGTACAATCCGGACCAYAAGCCGCCGAGTCCCAAG
GACTTGG
TGTACTTGGAGCCGTTTCCAGGGTTTTGCGAGAAGAACCCGAAGCTGGGGATCCCGGGCACGCCCGGGTGGCT
G-TGCAA
CGACACGTCGATCGGGGTCGACGGGTGCGAT-TGATGTGCTGCGGGAAGGGGCTACAGGACCCAGGAGGTCA-
----

>Astenus
GGGAGAAGCCCAGCACTTAATCCCGTGGCCGAACCGGGAAATGTAGTGTTTGGGAGGGTCCATCAACCACCGT
GCGACGC
GTCCAAGTCCTTCTTGAACGGGGCCACATACCCATAGAGGGTGCCAGGCCCGGTAGCCGGTGGATCTCTCCTC
AGAGTCG
GGTTGCTTGAGAGTGCAGCCCTAAGTGGGTGGTAAACTCCATCTAAGGCTAAATATGACCACGAGACCGATAG
CGAACAA
GTACCGTGAGGGAAAGTTGAAAAGAACTTTGAAGAGAGAGTTCAATAGTACGTGAAACCGTTCAGGGGTAAAC
CTGAGAA
ACCCGAAAGGTCGAATGGGGAGATTCAGCTTGTCTCGTTTCTGGTCGCGTGACGATGGTGCTTGCACCGGACT
GCACCTT
CCGGATCCGTAACCGGCGGCGAACTCGTGCACTTCTCCCCTAGTAGGACGTCGCGACCCGTTGGGTGCCGGTC
TAAGGCC
GACGGTGGAGCCTCGGATTCCCGGCCGGCCCGCTCGACGGTGTGACAGAGACGTGGGGTCGCCATGTTGGCGT
CCGGCCC
GTCACAAGTTCGGGCGGCCCGGATGTCGGACCTGTGTGCCGACCTCGGGCCCGCCGGCTGTTGGTGGCGGTGT
CCTCGGA
CAGACTACACGTCGGTCGGCGACGCTCTAGCTTTGGGTTTTCAGGACCCGTCTTGAAACACGGACCAAGGAGT
CTAGCAT
GTGCGCGAGTCATTGGGACCGCATCTAAACCTAAAGGCGAAATGAAAGTGAAGGCGTGCCGAGGGAGGATGGG
TCGGGGG
GCGTCTCGTTCTCATCGCGAGATGAGGCGCACCCAGAGCGTACACGC---
TACACCGTATTCGCTGATTTGTTCGA-TCC
CATTATTGAAGACTACCATGGTGGATTCAAGAAGACCGACAAGCATCCCCCGAAGAACTGGGGTGATGTAAAC
ACTTTCG
CCAATCTGGATCCAGCTGGTGAGTTTGTTGTGTCAACCCGTGTCCGTTGCGGCCGTTCAATGGAGGGTTACCC
ATTCAAC
CCATGCTTAACCGAAGAGCAATACAAGGAGATGGAAGCTAAAGTCTCGTCTACTYTATCTGGACTCGAAGGTG
AACTCAA
GGGTACTTTCTACCCATTGACTGGAATGGATAAGGATACYCAGCAGAAGCTCATCGACGATCACTTCTTGTTC
AAGGAAG
GTGATCGTTTCCTCCAGGCTGCTAACGCCTGCCGTTACTGGCCATCTGGACGTGGTATCTACCACAACGACAA
CAAAACC
TTCTTGGTCTGGTGCAACGAAGAGGACCATCTTCGCCTGATTTCGATGCAGATGGGAGGTGATCTTGGTGAAG
TCTACCG
TCGCCTTGTGAACGCCGTCAACGACATCGAGAAGCGCGTTCCCTTCTCCCACAATGACAGATTAGGTTTCCTC
ACCTTCT
GCCCAAGCAACTTGGGCACAACTGTACGTGCCTCTGTACACATTAAAGTACCTAAGCTCGCCGCCAACAAGGC
TAAGCTC

CCATACCTCAAAGAAGTT
GATGATGAAGAATTGCAAGAACCTACAGATAAGAGAATGTTTGTTTTAGCTGCCGCTTTAAGAAATGGCTACA
GCGTAGA
TAAACTATACGAATT-
GACAAAGATTGATCGTTGGTTCTTACAAAAAATGAAGAACATTGTAGATTATAAYACCCTTTTA
GAAACTGT---------
TAAATTACAAAATTGCAATAATATTTACAAACTTTTAWTGAAAGCRAAACAAATTGGTTTYAG
TGACAAACAAATTGCTGTTGCTGTTAAAAGTACTGAACTTGCTATTAGAAAACAAAGACAAGATTTTGGTATC
ACTCCTT
ACGTAAAACAAATAGATACTGTAGCTGCTGAATGGCCAGCTACAACAAATTACCTATATATAACTTATAATGC
AGATAGT
CATGATTTAACTTTCACTGATCAACATACAATGGTTATCGGTTCAGGAGTTTATAGGATTGGAAGTTCTGTTG
AGTTTGA
TTGGTGTGCTGTTGGTTGTTTAAGGGAGCTCAGAAAACTGAATAAAAAAACAATTATGGTTAATTACAATCCC
GAAACTG
TTAGTACTGATTATGATATGTCTGATCGTCTATACTTTGAAGAAATTTCATTTGAAGTTGTAATGGATGAAGT
TTATATT
TTAATTTTACCTGGATTTGGTCTTATTTCTCATATTATTAGACAGGCTAGAGGAAAAAAAGAAACATTCGGAT
CATTGGG
AATAATTTATGCAATAATAGCAATTGGATTATTAGGATTTGTAGTTTGAGCTCATCACATATTTACAGTTGGG
ATAGATG
TAGACACTCGGGCTTACTTCACCTCAGCCACAATAATTATTGCTGTGCCTACAGGAATTAAAATTTTCAGATG
ATTGGCT
ACATTGCATGGATCACAAATTAAATATAATCCATCCATATTATGATCTTTAGGATTTGTATTTTTATTTACAG
TAGGAGG
ATTAACTGGAGTAATTTTAGCTAATTCGTCTATTGACATTATTTTACATGATACATATTATGTAGTTGCCCAT
TTCCATT
ATGTTCTTTCCATAGGAGCAGTATTTGCAATTATAGCTGGATTAGTTCAGTGATTTCCATTATTTACAGGATT
AATAATA
AATGAGTATATTTTAAAAATTCAATTTTTAATTATATTTATTGGAGTAAATATAACATTTTTTCCACAACATT
TTTTAGG
ATTAGCCGGAATACCTCGACGTTACTCTGATTATCCGGATGCTTATACCCCATGAAACATAATTTCATCAATT
GGATCAT
TAATTTCTATAATTAGAATTTTTATTTTGTTATTTATTCTGTGAGAAAGATTTGTATCTATGCGAATTAATAT
TGCTAGA
AAAAATTTTTCTACTTCAATTGAATGATTACAATTAACCCCTCCAGCCGAACACAGATACTCTGAATTGCCTA
TAGTAAC
TAAAGATTATGTAGTTGTATTTGATTTCCTTGGT-
AAGGATTCCATTAGATATTACAATGAAGTACCTGTAGAGAAACGT
GTCTTCAAAAACCTTCAGCTGTTCATGGAGAATAAGTCGTCTGGTGATGATTTGTTTGAYAGATTGAACACRG
CTGTAAT
GAATAAACATTTGAATGAATTGATGGAAGGTTTAACAGCTAAAGTGTTTCGTACTTATAACGCGTCTTGGACT
TTACAGC
AACAACTTGATAAATTGACCAATCCAGATGATTCCATATCCGAAAAAATATTATCATACAATCGTGCCAATCG
AGCTGTA
GCTATACTTTGTAACCATCAACGTGCAGTGCCTAAAGGCCATCAGAAATCCATGGAGAAACTCAAGGAAAAGA
TTGAATC

KAAAAGGGAAAACATCAAGGACGCCGAGAGACAAGTTAAAGATGCGCAAAGAGATGCGAAACA---
CGGTAGCGTCAAGG
AGAAGCAGATTTACGAGAAGAAAAAGAAAATGCTGGAGAGACTCCGTGAACAATTGGCCAAATTGGAAATTCA
GGAGACG
GACCGTGACGAAAATAAAACTATTGCGCTCGGTACCTCCAAGCTGAACTATTTGGACCCGAGAATCTCGGTTG
CTTGGTG
TAAGAAGTTTGGTGTGCCCATAGAAAAGATTTATAATAAAACTCAA------
CGCCTGCCACCCTTCAGAGTAATAGGCG
ATCATTTRAAAGACCGCTTCGACGGAGCCTCTAGAGTCATGCTAAGCAACTCGGCGAGTTCCCGAGG------
------A
AACTC---CAACCGGC---
CCAAACAAGACAAACTCTCCAACAACATCGCCTCGAACAGCATCCACAGCAAAAGAGAGAA
CCGACCGAGGAAATACAARTACGGCTTCCAACTCAAACCYTACAATCCCGATCACAAACCTCCGAGCCCYAAA
GACCTTG
TTTACCTCGAACCRTCGCCCGGWTTCTGCGAGAAGAACCCCAAGTTAGGGATACAGGGYACTCACGGTAGGTT
G-TGCAA
CGATACATCGATCGGGGTCGACGGGTGCGATCTGATGTGCTGCGGAAG-
GGGCTACAGGACCCAGGAGGTCATCGTTGTC
GAGAGGTGCAACTGCACG-------------
>Astenus_USA
GGGAGAAGCCCAGCACTTAATCCCGTGGCCGAACCGGGAAATGTAGTGTTTGGGAGGGTCCATCAACCACCGT
GCGACGC
GTCCAAGTCCTTCTTGAACGGGGCCACATACCCATAGAGGGTGCCAGGCCCGGTAGCCGGTGGATCTCTCCTC
AGAGTCG
GGTTGCTTGAGAGTGCAGCCCTAAGTGGGTGGTAAACTCCATCTAAGGCTAAATATGACCACGAGACCGATAG
CGAACAA
GTACCGTGAGGGAAAGTTGAAAAGAACTTTGAAGAGAGAGTTCAATAGTACGTGAAACCGTTCAGGGGTAAAC
CTGAGAA
ACCCGAAAGGTCGAATGGGGAGATTCAGCGTGTCTCGTTTCTGGTCGCGTGACGATGGTGCTTGCACCGGACT
GCACCTT
CCGGATCCGTAACCGGCGGCGAACTCGTGCACTTCTCCCCTAGTAGGACGTCGCGACCCGTTGGGTGCCGGTC
TAAGGCC
GACGGTGGAGCCTTAGATTCCCGACCGGCCCGCTCGACGGTGTGACAGAGACGTGGGGTCGCCATGTTGGCGT
CCGGCCT
GTCACAAGTTCGGGCGGCCCGGATGTCGGACCTGTGTGCCGACCTCGGGCCCGCCGGCTGTTGGTGACTGTGT
CCTCGGA
CAGACTACACGTCGGTCGGCGACGCTCTAGCTTTGGGTTTTCAGGACCCGTCTTGAAACACGGACCAAGGAGT
CTAGCAT
GTGCGCGAGTCATTGGGACCGCATCTAAACCTAAAGGCGAAATGAAAGTGAAGGCGTGCCGAGGGAGGATGGG
TCGGGGG
GCGTCTCGTTCTCATCGCGAGTTGAGGCGCACCCAGAGCGTACACGCTCTTACACCGTATTCGCTGACTTGTT
CGA-CCC
CATCATCGAAGACTACCACGGTGGATTCAAGAAGACCGACAAGCATCCCCCGAAGAACTGGGGTGACGTAAAC
ACTTTTG
CCAATTTGGACCCAGCTGGTGAGTTCATTGTGTCAACCCGCGTCCGTTGCGGCCGTTCAATGGAAGGTTACCC
ATTCAAC
CCGTGCTTGACCGAAGAGCAATACAAGGAGATGGAAGCTAAAGTTTCCAGCACCTTATCAGGGCTTGAAGGTG
AACTTAA
AGGCACCTTCTATCCATTGACTGGAATGGATAAGGATACCCAACAGAAGTTGATTGATGATCACTTCTTGTTC
AAGGAAG
GTGATCGTTTCCTTCAAGCTGCTAATGCTTGCCGCTTCTGGCCGTCTGGACGTGGTATCTACCACAACGACAA
CAAAACC
TTCTTGGTTTGGTGCAACGAAGAAGATCATCTCCGTATTATTTCCATGCAGATGGGCGGTGATCTTGGTGAAG
TCTACCG
TCGTTTGGTGAACGCTGTCAACGACATCGAGAAGCGCATTCCGTTCTCTCATAACGACAGATTAGGTTTCTTG
ACCTTCT

GCCCAAGCAACTTGGGCACAACTGTACGTGCCTCTGTACACATTAAAGTACCTAAGCTCGCCGCCAACAAGGC
TAAGCTG
GATGAAATTGCTGCCAAATACAACTTGCAAGTACGTGGTACCCGT---
GTTCCTAAAAAAGTTTTAATTATTGGATCAGG
GGGATTGTCTATTGGCCAAGCTGGTGAATTTGATTATTCCGGTTCACAAGCAATCAAAGCTTTACATGAAGAC
AATATTC
AAACTGTTTTAATTAATCCTAATATTGCAACAGTTCAAACATCTAAAGGTTTAGCTGATAAAATATACTTTTT
ACCATTA
GTACCTGAATTTGTTGAACAGGTAATTAGGTCAGAACGACCTGGTGGTGTTTTATTAACGTTTGGTGGACAAA
CTGGATT
AAATTGTGGAGTAGAATTACAAAAAGCTGGTGTTTTTGAAAAATATGGTGTTAAAATTTTGGGTACTCCAATA
CAAGCTA
TAATAGATACTGAAGATAGAAAGATATTTAGTGAGAGAATCTCACAGATTGGTGAAAAAGTTGCTCCAAGCAT
GGCCGCA
TATTCAGTGCAAGAAGCTTTGGAAGCGGCAGATTTATTAGGATATCCCGTTATGGCAAGAGCAGCTTTTTCAT
TAGGTGG
TCTAGGATCTGGCTTTGCCAACACAGCTGAAGAATTGAAATTACTTGCTCAACAAGCTTTGGCTCATTCAAAT
CAATTGA
TTATTGATAAGTCTTTGAAAGGATGGAAAGAGGTGGAATATGAAGTAGTAAGAGATGCTTATCCATACCTTAA
AGAAGTT
GATGATGAAGAATTACAAGAGCCTACAGATAAAAGAATGTTTGTTTTAGCTGCTGCTTTAAGAAATGGCTACA
GCGTAGA
TAAACTATATGACTT-
AACCAAAATTGATCGATGGTTCTTACAGAAAATGAAGAACATTGTAGATTATAATACACTTTTA
GAAACAGT---------
TAAATTACAAAATTGTGATAATGTTTACAAACTTTTGTTGAAAGCAAAACAAATCGGTTTCAG
TGATAAACAAATTGCTGTTGCTGTTAAAAGTACAGAACTTGCTATTAGAAAACAAAGACAAGATATTGGTATC
ACTCCAT
ATGTAAAACAGATAGATACTGTAGCTGCTGAATGGCCAGCTACAACAAATTATTTATATATAACTTATAATGC
AGAAAGT
CATGATTTAACATTTACCGATCAACACACAATGGTTATTGGTTCAGGAGTTTATAGGATTGGAAGTTCTGTTG
AGTTTGA
TTGGTGTGCTGTTGGTTGTTTAAGAGAGCTAAGAAAATTGAACAGAAAAACTATAATGGTTAATTACA-----
----

GAAGTTTATATT
TTAATTTTACCTGGTTTTGGAATAATTTCTCACATTATTAGCCAGGCTAGAGGAAAAAAAGAAACATTTGGAG
CCTTAGG
AATAATTTATGCCATAATAGCAATTGGTTTATTAGGGTTTGTAGTTTGAGCCCATCATATATTTACCGTTGGA
ATAGACG
TAGATACTCGAGCCTATTTTACCTCAGCAACTATAATTATTGCAGTTCCTACAGGAATTAAAATTTTTAGGTG
ATTAGCC
ACTCTTCACGGATCACAAATCAAGTATAATCCCCCAATACTATGATCTTTAGGTTTTGTGTTTTTATTTACAA
TCGGAGG
ATTGACTGGAGTTATTTTAGCTAATTCATCTATTGATATTATTTTGCATGATACATACTACGTAGTTGCCCAT
TTTCATT
ATGTTCTATCTATAGGAGCAGTATTTGCTATTATAGCGGGATTAGTTCAATGATTCCCACTATTTACAGGTTT
AATGATA
AATGAATTTTTCTTAAAAATTCAATTTTTTGTTATATTTATTGGAGTCAATATAACGTTCTTCCCTCAACATT
TTTTAGG
ATTAGCAGGAATACCTCGACGATATTCAGACTATCCTGATGCATATACCCCCTGAAACGTTATCTCATCAATT
GGATCGT
TAATTTCAATAATTAGAATTTTTATTCTTTTATTTATTTTATGAGAAAGATTTGTTTCAATACGAATTAATAT
TTCAAGA
AAAAATTTTGCTACATCAATTGAATGATTCCAGCTAACTCCTCCCGCTGAACATAGATAC-------------
----

-AAAGATTATGTAGTTGTATTTGATTTCCTTGGT-
AAGGATTCCATTAGATATTACAATGAAGTACCTGTAGAGAAACGT
GTCTTCAAAAACCTTCAGCTATTCATGGAAAACAAATCACCTGGTGATGATTTGTTCGATAGGTTAAATACAG
CTGTAAT
GAATAAACATTTAAATGAATTGATGGAAGGTTTAACTGCCAAGGTGTTTCGTACTTATAACGCTTCTTGGACA
TTACAGC
AACAACTTGATAAATTGACCAATCCAGATGATTCCATATCCGAAAAAATATTATCATACAATCGTGCCAATAG
AGCTGTA
GCTATACTCTGTAACCATCAACGTGCAGTGCCTAAAGGCCATCAAAAATCCATGGAGAAACTCAAAGAAAAAA
TCGAAAC
GAAAAGAGATAACATTAAGGACGCGGAGAGACAAGTTAAAGATGCACAAAGGGACGCGAAACA---
CGGTAGCGTTAAGG
AGAAGCAGATTTACGAGAAGAAAAAGAAAATGCTGGAGAGACTTCGTGAACAATTAGTTAAATTGGAAATTCA
GGAGACG
GACCGCGACGAAAATAAAACAATTGCGCTCGGTACGTCCAAGCTGAACTATTTGGATCCGAGAATCTCGGTCG
CTTGGTG
TAAGAAGTTTGGTGTCCCAATTGAAAAGATTTATAATAAAACTCAA------
CGCCTACCACCCTTCAGGGTAATAGGTG
ACCATCTAAAAGACCGCTTCGACGGCGCTTCTAGAGTTATGTTGAGCAACTCCGCCAGTTCCCGTGG------
------A
AATTC---CAACCGCC---
CCAAACAAGACAAGCTCTCCAACAACATCGCCAGCAACAGCATCCACAGCAAACGCGAAAA
CCGGCCTAGAAAATACAAGTACGGTTTCCAGCTCAAACCCTACAACCCAGACCACAAACCCCCCAGCCCTAAA
GATCTCG
TTTATTTGGAGCCTTCGCCAGGTTTCTGCGAGAAGAACCCTAAGCTGGGGATTCAGGGTACTCACGGTAGGTT
G-TGCAA
CGATACTTCTATAGGAGTCGACGGGTGCGATTTG---------------------------------------
----

>Cylindroxystus_longulus
GGGAGAAGCCCAGCACTGAATCCCGCGGCCTAGCCGGGAAATGTGGTGTTTGGGAGGATCCGTCATCCACCGT
GCGACGA
GCCCAAGTCCTTCTTGAACGGGGCCACATACCCATAGAGGGTGCCAGGCCCGGTCGCCGGAGGATCTCTCCTT
AGAGTCG
GGTTGCTTGAGAACGCAGCCCCAAGTGGGTGGTAAACTCCATCTAAGGCTAAATATAACCACGAGACCGATAG
CGAACAA
GTACCGTGAGGGAAAGTTGAAAAGAACTTTGAAGAGAGAGTTCAATAGTACGTGAAACCGTTCAGGGGTAAAC
CTGAGAA
ACCCGAAAGGTCGAACGGGGAGATTCAGCGTGTTCAGTCCTCGGTTGCGAGACGGTGGTGCATGCACCGGTCC
GCTCCTT
TCTCGCGCCGCGCTTCCGGCGAAC-
CGTGCACTTCTCCCCTTGTAGGACGTCGCGACCCGTTGTGCGCCGATCTACGGCC
GACGGTGGAGCCTTGGGCTCCCGACCGGCCCGCACGACGGTACGAATAAGACGTGAGGCCGCGAAGTTCGCGT
CCGGCCC
GCCGCAAGCACGCGCGATCCGATCGTCGGACCAGTGTGCCGACCTCGGACTCGCCGGCTGCTAGCGACGGTGC
TCTCGGA
CAGGCTACACGTCGGTCGGCGACGCTCTAGCTTTGGGTTTTCAGGACCCGTCTTGAAACACGGACCAAGGAGT
CTAGCAT
GTGCGCGAGTCATTGGGACCGCATCGAAACCTAAAGGCAAAATGAAAGTGAAGGCGCGCCTAGGGAGGATGGG
TCGGGGG
GCGTCTCGTTCTCATTGCGAGATGAGACGCACCCAGAGCGTACACGC--------------------------
----

ATTGAAGATTACCATGGTGGATTTAAGAAGACAGATAAGCATCCACCTAAGAATTGGGGCGACGTGAACACTT
TTG
CTAACTTGGACCCAGCTGGTGAATTCGTCGTATCAACTCGCGTCAGATGCGGTCGTTCCATGGAAGGATATCC
TTTCAAC

CCCTGTTTAACCGAAGAGCAATACAAGGAAATGGAACAAAAAGTATCAACGACTTTGTCTGGTCTCGAAGGAG
AATTAAA
GGGTACCTTCTATCCGTTGACTGGAATGGATAAGGATACTCAGCAGAAACTGATTGATGATCATTTCTTGTTC
AAGGAAG
GAGATCGTTTCCTCCAAACCGCTAACGCTTGCCGTTACTGGCCAAGCGGACGTGGCATCTACCACAACGACAA
CAAAACG
TTCTTGGTCTGGTGCAACGAAGAGGACCATCTCCGCATTATCTCCATGCAGATGGGCGGTGATCTTGGTGAAG
TCTACCG
TCGCCTTGTAACAGCCGTCAACGAAATCGAAAAGCGCGTACCATTCTCCCACAACGACAGATTAGGTTTCCTC
ACGTTCT
GTCCCACCAACTTGGGCACAACTGTTCGCGCCTCTGTTCACATCAAGGTACCAAAGCTCGCCGCCAACAAAGC
CAAGCTC
GACGAAATCGCCGGAAAGTACAACTTGCAAGTACGTGGTACACGTGGTGTTCCCAAAAAAGTTTTGATTATTG
GGTCAGG
CGGTTTATCGATCGGACAAGCAGGTGAATTTGATTATTCGGGTTCTCAGGCAATTAAAGCATTGAGAGAAGAG
AATATTC
AAACAGTTTTAATTAATCCTAATATAGCAACTGTTCAAACATCTAAAGGTTTATCTGATAAAGTTTATTTTCT
TCCATTA
ACACCTGAGTATGTAGAGCAAGTTATTAAAGTTGAAAGACCTGGAGGTGTTCTATTAACTTTTGGAGGACAAA
CTGGTTT
AAATTGCGGTGTTGAGTTGCAACAAGCTGGAGTCTTTGATAAATATGGCGTAAAAATTTTGGGCACACCAATT
GAGGCGA
TCATCAATACTGAAGATAGAAAAATTTTCAGCGATCGAATTGGAATGATTGGTGAAAGAGTAGCGCCTAGCAT
GGCTGCT
TATACGGTTCAAGAAGCTTTAGATGCGGCAGAGCAATTAGGATATCCTGTAATGGCGCGAGCTGCATTTTCTT
TGGGAGG
ATTAGGATCTGGTTTTGCAGATAATATCGAGGAATTGAAGATATTAGCTCAACAAGCTTTAGCTCATTCAAGT
CAATTGA
TTATAGATAAATCGTTGAAAGGTTGGAAAGAAG-----------------------------
CCTTACATTAAACAAGTT
GATGATGAAGAATTAAAAGAACCTACTGATAAGAGGATGTTTGTTGTTGCTGCTGCGTTAAAACATGGTTATA
GTGTGGA
TAAATTATACGAATT-
AACGAAAATTGATCGATGGTTTTTGCAAAAAATGAAAAATATTACCGATTATATTTCATTTTTA
GAAGCAACAAATCAAAATAAGTTA---------------
ACGCATAATATATTATTGAAAGCTAAACAAATTGGATTTAG
TGATAAACAAATAGCTGTAGCTGTTGAAAGTACTGAGCTTGCAATTAGGAAATTACGACAAGATTTAAATATT
ATTCCAT
TTGTTAAACAAATCGATACAGTTGCTGCTGAATGGCCAGCAACGACTAATTATCTATATTTGACTTATAACGC
CAATAGT
CATGATTTAAACTTTAACGAAGAACATATTATGGTCATTGGATCTGGAGTCTATCGAATTGGTAGTTCAGTTG
AGTTTGA
TTGGGGTGCAGTTGGATGTTTACGAGAGCTTAGAAACTTAAATAAAAAAACGATTATGGTT------------
----

CTCATTCTACCTGGATTTGGGTTAATTTCACATATTATTATACAATCCAGAGGTAAAAAAGAAACTTTCGGAA
CTTTAGG
AATAATTTATGCTATATTAGCTATTGGTCTATTAGGATTCATTGTCTGAGCTCATCATATATTTACAGTAGGT
ATAGATG
TAGACACTCGAGCTTATTTTACCTCAGCAACAATAGTAATTGCTATTCCTACAGGAATCAAAATTTTTAGGTG
ATTAGCT
ACATTCCATGGAACTCAACTAACATTTAATCCCCCAATACTATGAGCTTTAGGGTTTGTTTTCCTATTTACAA
TAGGAGG
ATTAACAGGAGTAATTTTAGCTAATTCATCAATCGATATTATTTTACATGATACCTATTATGTTGTAGCTCAT
TTTCATT

ATGTTTTATCAATAGGGGCAGTCTTTGCAATTATAGCCGGTATTATTCAATGATTCCCTCTATTTACAGGAAC
CTATATA
AATAATTTTTTACTAAAAATTCAATTTTTATTAATATTTATCGGAGTAAATCTTACATTTTTTCCTCAACATT
TTCTAGG
ATTAGCAGGGATACCCCGCCGATATTCAGATTATCCTGATGCCTTTACAACTTGAAACGTAATTTCATCTATT
GGTTCAT
TAATTTCTTTAATTAGAATTTTTTTATTGATATTTATTATTTGAGAAAGATTAACTTCAATTCGAATTATAAT
ATCTCCA
AATAATTTTTCTACATCAATCGAATGACTACAAAAATTTCCACCTTCAGAACATAGTTACTCAGAATTGCCTA
TTATATC
A---

---TGGATGCGGCTGCCTCCGTTCCGCGT-
ATCGGCG
ACCAGCTGAAGGACCGCTTCGACGGCGCCTCCCGGGTGATGCTGAGCAATTCGGCGAGCTCGCGGGGA-----
----AAC
GGCGC---GAACCGGC---
CGAAACAGGACAAGCTCTCCAACAGCATCGCCTCCAACAGCATCCACAGCAAGCGCGAGAA
CAGACCGAGGAAGTACAAGTACGGCTTCCAGCTGAAACCGTACAATCCGGATCATAAACCTCCGAGCGCTAGG
GATCTGG
TGTATCTGGAACCGTCGCCGGGTTTTTGCGAGAAGAACCCGAAATTGGGGATACAGGGGACGCACGGGAGACA
G-TGCAA
CGATACGTCGATCGGCGTCGACGGGTGCGATCTGATGTGCTGCGGAAG-
AGGGTACAGGACGCAGGAGGTCGTCGTCGTC
GAGCGCTGCAACTGCACGTTC----------
>Dibelonetes
GGGAGAAGCCCAGCACTGAATCCCGTGTCCGAGCCGGGAAATGTAGTGTTTGGGAGGGTCCGCCAACCATCGT
ACGACGC
GTCCAAGTCCTTCTTGAACGGGGCCACATACCCATAGAGGGTGCCAGGCCCGGTAGCTGGAGGATCTCTCCTC
AGAGTCG
GGTTGCTTGAGAGCGCAGCCCTAAGTGGGTGGTAAACTCCATCTAAGGCTAAATATGACCACAAGACCGATAG
CGAACAA
GTACCGTGAGGGAAAGTTGAAAAGAACTTTGAAGAGAGAGTTCAATAGTACGTGAAACCGTTCAGGGGTAAAC
CTGAGAA
ACCCGAAAGGTCGAATGGGGAGATTCAGCGTGTCTCGTTTCTGGTCGCGTGACGATGGTGCTTGCATCGGACT
GCGCCTT
CTGGATCCGCAACCGGCGACGAACTCGTGCACTTCTCCCCTAGTAGGACGTCGCGACCCGTTGGGTGCCGGTC
TAAGGTC
GACGGAGGAGCCTCAGAGTCCCGGCCGGCCCGCTCGACGGTAAGACAGAGACGTGGGGTCGCTACGTTAGCGT
CCGGCCC
GCTACAAGTTCGGGCGACTCGGATGTCGGACCTGTGTGCCGACCCCGAGATCGCCGGCTGTTGGTGGCGGTGT
CCTCGGA

CAGACTACACGTCGGTCGGCGACGCTATAGCTTTGGGTTTTCAGGACCCGTCTTGAAACACGGACCAAGGAGT
CTAGCAT
GTGCGCGAGTCATTGGGACCGCATCTAAACCTAAAGGCGAAATGAAAGTGAAGGCGTGCCAAGGGAGGATGGG
TCGGGGG
GCGTCTCGTTCTCATCGCGAGATGAGGCGCACCCA-------------
CTTACACCGTATTCGCTGATTTGTTCGA-TCC
CATCATCGAGGACTACCATGGTGGCTTCAAGAAGACCGACAAGCATCCACCTGCAAACTGGGGTGATGTAAAT
GTCTTCG
CCAATCTGGACCCAGCTGGCGAGTACGTGGTGTCAACCCGCGTCCGTTGCGGCCGCTCCATGGAGGGTTACCC
CTTCAAC
CCTTGCTTAACCGAAGAGCAATACAAAGAGATGGAAGGAAAGGTGTCCACCACCTTGTCTGGTTTGGAAGGTG
AACTCAA
GGGTACCTTCTATCCATTGACCGGAATGGATAAGGCTACTCAACAGAAGCTCATCGATGATCACTTCTTGTTC
AAGGAAG
GTGATCGTTTCCTCCAGACAGCGAACGCTTGCCGTTACTGGCCATCTGGACGTGGTATCTACCACAATGATAA
CAAGACC
TTCTTGGTATGGTGCAACGAAGAAGATCATCTCCGCATCATCTCGATGCAGATGGGTGGTGATCTTGGCGAAG
TCTATCG
TCGTCTTGTGACTGCCGTCAATGAGATCGAGAAGCGCGTTCCATTCTCCCATAATGACAGATTGGGTTTCCTC
ACCTTCT
GCCCAACAAATTTGGGAACAACTGTACGTGCCTCTGTACACATCAAAGTACCAAAGCTCGCCGCCAACAAGGC
CAAGCTT

GTTCCAAGAAAAGTTTTAATAATTGGTTCAGG
CGGTCTATCGATTGGACAAGCTGGAGAATTCGATTATTCCGGTTCGCAGGCGATCAAAGCCTTACAAGAAAAA
AGTATAC
AAACAGTTTTGATCAACCCGAACATTGCAACTGTGCAAACTTCAAAGGGTTTAGCTGATAAAATCTACTTTTT
ACCTTTG
GTTCCCGAGTTTGTTGAGCAAGTTATACGATCAGAAAGACCCGGCGGAGTTTTACTGACTTTTGGAGGGCAGA
CTGGGTT
GAATTGCGGTGTAGAGTTGCAGAAAGCCGGTATATTCGAAAAGTACGGTGTTAAGATACTAGGTACACCTATC
CAAGCTA
TAATAGATACTGAAGACCGAAAGATATTTAGCGAAAGGATTGCTATGATTGGTGAAAAAGTTGCTCCAAGCAT
GGCTGCT
TATTCGGTCCAAGAAGCTTTGGAAGCTGCAGATTTGTTAGGGTATCCAGTAATGGCAAGGGCTGCTTTTTCTT
TAGGAGG
TCTAGGATCTGGCTTTGCAGATACTGCTGAAGAACTCAAATTACTTGCTCAACAAGCTTTAGCGCACTCGAAT
CAGTTGA
TTATCGATAAATCTCTAAAAGGATGGAAGGAAGTCGAGTATGAGGTTGTAAGAGATGCTTATCCTTACCTCAA
AGATGTC
GATGATGACGAACTGAAGGAACCCACTGATAAAAGAATGTTTGTCCTTGCTGCTGCTTTAAGAAGTGGTTACA
GCATTGA
TAAACTCTATGATCT-
GACGAAAATTGATCGTTGGTTCTTGCAGAAAATGAAGAACATCATAGATTACAACACTTTTATA
GAATCTGTCGCTCAAAATAAATTCCAAG---
GCACAAACATTTATAAAGTTTTGTTGAAAGCGAAACAAATCGGTTTCAG
TGATAAACAGATAGCTGTTGCTCTTAAAAGTACTGAACTCGCTATCAGAAAGCAACGGCAAGATTTCGGGATC
ACGCCTT
TTGTGAAACAAATAGATACTGTAGCTGCTGAATGGCCTGCAACTACGAATTACTTGTACCTAACGTACAATGC
TGAAAGT
CATGATTTGACATTCAGTGAGCAACACATAATGGTAATTGGATCAGGTGTTTATAGGATAGGAAGTTCTGTAG
AGTTTGA
TTGGTGCGCTGTTGGATGTTTGAGGGAATTAAGAAAACTAAATAAGAAAACAATAATGGTSAATTACAATCCT
GAAACTG
TGAGCACAGATTACGATATGTCAGATAGATTGTATTTTGAGGAAATATCCTTTGAAGTCGTTATGGATGAAGT
CTACATT

TTAATTTTACCTGGATTTGGATTAATTTCTCACATTATTAGACAAGCTAGAGGTAAAAAGGAAACTTTTGGTT
CATTAGG
AATAGTATATGCTATAATAGCTATTGGACTATTAGGATTCGTAGTTTGAGCTCATCATATATTTACAATTGGA
ATAGATG
TTGATACTCGGGCTTATTTTACTTCAGCCACAATAATTATTGCAGTTCCTACAGGAATTAAAATTTTTAGATG
ATTAGCT
ACTTTACATGGAACTCAAATAAAATTTAATCCTTCAATATTATGAGCTTTAGGATTTGTATTTTTATTTACAA
TCGGAGG
TTTAACTGGAGTTATTTTAGCTAATTCATCAATTGATATTATTTTACATGATACTTATTATGTAGTAGCTCAT
TTTCATT
ATGTTCTTTCTATAGGAGCAGTTTTTGCTATTATAGCTGGATTAATTCAATGATACCCATTATTTACAGGATT
AATAATA
AATGAATATATGTTAAAAATTCAATTTTTTATTATATTTATTGGAGTAAATATAACCTTTTTTCCTCAACATT
TTTTAGG
ATTAGCAGGTATACCTCGACGATATTCTGATTATCCAGATGCTTATACACCGTGAAATATTGTATCTTCTATT
GGATCAT
TAATTTCAATAACTGCAGTATTTTTATTATTATTTATTATTTGAGAAAGTTTTATTTCAATACGAATAAATAT
TTCATCT
AATAATCTATCTTCTTCAGTAGAATGATATCAACTATTTCCACCTGCTGAACATAGATATTCTGAATTACCTG
CTTTAAC
AAAAGAATACGTAGTAGTATTTGATTTCCTCGGT-
AAGGATTCAATTAGGTACTACAATGAAGTACCTGTAGAGAAACGT
GTCTTCAAAAACCTCCAACTGTTTTTGGAAAACAAGGAACCCGGTGATGATTTGTTTGATAGATTAAATACAG
CTGTGAT
GAACAAACATTTAAACGAGTTGATGGAAGGTTTAACTGCCAAGGTGTTTCGTACTTATAACGCGTCTTGGACT
CTACAGC
AGCAACTTGATAAATTGACCAATCCAGATGATTCCATATCTGAAAAAATCTTATCGTACAACCGCGCCAACAG
AGCGGTA
GCTATCCTCTGTAACCATCAACGTGCTGTACCGAAAGGTCACCAGAAATCCATGGAGAAACTMAAAGAAAAAA
TCGAAAC
TAAAAAGGATAACATCAAAGATGCTGAAAGGCAAGTTAAGGATGCGCAGAGGGATGCGAAACA---
CGGAAGCGTTAAAG
AGAAGCAGATCTACGACAAGAAGAAGAAGATGTTGGAGAGGCTAAAGGATCAGCTGGCCAAGTTGGAGATTCA
GGAGACG
GACCGCGATGAAAATAAAACTATTGCCCTCGGCACGTCCAAGCTGAATTATTTGGATCCTAGGATTTCCGTTG
CATGGTG
TAAGAAGTTCGATGTGCCCATTGAAAAAATTTATAACAAAACTCAG------
CGGCTTCCTCCTTTCCGAGTCATCGGCG
AYCACCTGAAGGACCGCTTCGATGGTGCATCYAGRGTGATGCTCAGCAATTCWGCGAGTTCAAGAGG------
------R
AACGC---WAACAGAC---
CSAAACAGGATAAGCTWTCAAACAGCATCGCGTCCAACAGCATACACAGCAAGAGGGARAA
CCGACCTCGCAAGTACAAGTACGGTTTYCAGTTGAAACCTTACAATCCTGATCACAAACCACCAAGTCCTAAG
GATCTGG
TGTACCTGGARCCTTCACCTGGGTTCTGCGAGAAGAACCCAAAAYTRGGGATTCAAGGTACTCATGGTAGATT
G-TGCAA
CGACACGTCGATCGGGGTCGACGGGTGYGACCTCATGTGCTGCGGCAG-
GGGGTACAGGACCCAAGAGGTCATCGTCGTC
GAGAGGTGCAACTGCACG-------------
>Diochus_schaumi
GGGAAGAGCCCAGCACTGAATCCCGCGGACTTGTCGGGAAATGTAGTGTTTGGGAGGGTCCACAATCCGTGGT
GCGGCGC
GTCCAAGTCCTTCTTGAACGGGGCCACTTGCCCATAGAGGGTGCCAGGCCCGGCAGCGGGAGGATCTCTCCTC
GGAGTCG
GGTTGCTTGAGAGTGCAGCCCTAAGTGGGTGGTAAACTCCATCTAAGGCTAAATATGACCACGAGACCGATAG
CGAACAA

GTACCGTGAGGGAAAGTTGAAAAGAACTTTGAAGAGAGAGTTAAATAGTACGTGAAACCGTTCAGGGGTAAAC
CTGAGAA
ACCCGAAAGGTCGAATGGGGAGATTCAGCGCGTATCGATGTCTGTTGAGAGACGGTATCGTTCGCG-
AGAGCCGCTCCAT
CGGTCACCGTCGTC--
CGACGAACGCGTGCACTTCTCCCCTAGTAGGACGTCGCGACCCGTTGGGCGTCGGTCAGAGGTC
CGCACGGTAGACCGCGCGGCCCGGCCGACACGCTCGACGGTACGACAATGGCGCGGGGCCGCGACGTTCGCGT
CTGGCCC
GTCGCAAGTACGAGCGGTTCGGATGTCGGACCTG-
GTGCCGACTCTGGACCCGCTGGCTGCTGGCGACGGTGTCCTTGGA
CAGACCAAGCGCCGGTCAGCGACGCTATAGCGTTGGGTTTTCAGGACCCGTCTTGAAACACGGACCAAGGAGT
CTAGCAT
GTGCGCGAGTCATTGGGACT--
AGCGAAACCTAAAGGCGAAATGAAAGTGAAGGCTTGCCGAGGGAGGATGGGCGGTGGG
GCGTCTCTAGCTCATTGCGAGCTGAGGCGCACCCAGAGCGTACACGC--------------------------
----

CACGGTGGATTCAAGAAGACGGACAAGCACCCACCTGCTAACTGGGGTGATGTAAACACCTTCG
CCAACTTGGACCCTAATGGCGAGTACGTCGTTTCTACTCGCGTCCGTTGCGGTCGCTCCATGGAGGGTTACCC
ATTCAAC
CCCTGCTTGACCGAGGAACAGTACAAAGAAATGGAACAGAAAGTTTCCACCACTCTCTCTGGTCTCGAGGGTG
AATTGAA
GGGTACCTTCTACCCACTTACTGGCATGAGCAAGGAGGTTCAGCAGAAACTCATCGACGACCACTTCCTCTTC
AAAGAAG
GTGATCGCTTCCTCCAGACCGCCAACGCTTGCCGTTACTGGCCATCTGGACGTGGTATCTACCACAACGACAA
CAAGACC
TTCTTGGTCTGGTGCAACGAGGAGGACCATCTCCGCATCATCTCCATGCAGATGGGTGGTGATTTGGGCGAAG
TCTACCG
TCGCCTCGTAACTGCTGTTAACGAGATCGAGAAGCGCGTTCCATTCTCCCACAACGATCGTCTTGGTTTCCTC
ACTTTCT
GCCCAACCAACTTGGGCACCACTGTTCGTGCCTCCGTGCATATTAAGGTTCCCAAACTTGCCGCCAACAAGGC
CAAACTC
GACGAAATCGCTGGTAAGTATAATCTCCAAGTACGTGGTACCCGTGGT-------------------------
----

GAATTAAAAGAACCTACGGATAAACGTATGTTTGTAGTGGCTGCAGCTCTAAGGCACGGTTACACAGTCGA
TCACCTTTACAACCT-
CACTAAAATTGATAGATGGTTCTTGCAAAAAATGAAGAATATAATAGATTACAACTGTTATCTT
GAATCGATTG------ATAATTTA---------------
TCACATGGAGCACTTTTGAAAGCGAAACAAATGGGTTTTAG

TGATAAACAAATAGCAGTTGCCGTTAAAAGCACCGAATTGGCTATTAGAAAACAACGTAAAGATTATGATATT
ATCCCAT
TTGTTAAACAAATTGATACTGTTGCTGCTGAATGGCCAGCGACTACTAACTATCTTTATTTAACATATAACGC
TTTAAAT
CATGATATAAAATTCGTGGAACAACATACAATGGTCATTGGTTCGGGCGTTTATCGTATTGGAAGTTCAGTAG
AATTTGA
TTGGTGTGCTGTTGGTTGCTTACGTGAATTACGTAAACTGAATCGGAAAACTATTATGGTAAACTACAATCCG
GAAACTG
TTAGTACTGACTACGATATGTCGGATCGTCTTTATTTTGAAGAAATTTCATTCGAAGTCGTCATGGATGAAGT
TTATATT
TTAATTCTACCAGGATTTGGAATAATTTCTCATATTATTAGACAAGAAAGAGGAAAAAAAGAAGCTTTTGGAG
CTTTAGG
AATAATTTATGCTATAATAGCAATTGGACTATTAGGATTTATTGTTTGAGCTCACCACATATTTACTGTAGGA
ATAGATG
TAGATACTCGAGCTTATTTTACTTCTGCTACAATAATTATTGCAGTGCCCACAGGTATTAAAATTTTTAGTTG
ACTTGCT
ACATTGCATGGAACTCAAATTAATTACTCTCCTTCAATATTATGAGCTTTAGGATTTGTTTTTTTATTCACTG
TAGGAGG
ATTAACAGGAGTAATTTTAGCTAATTCATCAATTGATATTATTTTACATGATACTTATTATGTAGTAGCCCAT
TTTCATT
ATGTACTTTCTATAGGAGCAGTTTTTGCAATTATAGCAGGATTAATTCAATGATTCCCTTTATTTACTGGTTT
AACTTTA
AATGAAAAATGATTAAAAATTCAATTTCTAACTATATTTATTGGAGTAAATTTAACTTTTTTCCCTCAACATT
TTTTAGG
ATTAGCAGGAATACCCCGACGATATTCAGATTATCCAGATGCTTACACCCCTTGAAATATTTTATCCTCTATT
GGATCAT
TAATTTCAACTGTTAGAATTTTTACTTTATTATTTATTATTTGAGAAAGATTCACCTCAATACGAAAAAGAGT
AACCCCA
TTAAGATTATCTACCTCTATTGAATGATATCAATCAATACCCCCTGCTGAACATTCATATTCTGAATTGCCTA
TA-----

TTTGATCGTTTAAATACTGCAATCAT
GAATAAGCACTTAAACGAATTAATGGAAGGCCTAACAGCTAAGGTTTTCCGTACATACAACGCTTCATTTACC
TTACAGC
AACAGTTGGAAAAGTTGACCAATCCTGAAGATTCCTTGTCTGAAATATTGCTGTCTTACAATCGTGCAAATAG
GGCCGTT
GCAATTCTTTGTAACCATCAACGTGCTGTTCCTAAAGGTCATCAAAAATCGATGGAAAAATTGAAAGAAAAAA
TTGACGC
CAAGCGTGAAGCAATCCGTGATGGAGAGAGACAAGTGAAAGATGCTCAAAAGGATGCTAAGCA---
CGGAAGTGTTAAAG
AGAAGACGGTTTATGAAAAGAAGAAGAAAATGTTGCAAAGATTGAAAGAACAATTAACCAAATTAGAGATTCA
GGAGACC
GATAGAGATGAGAATAAAACAATCGCTTTGGGAACATCAAAGTTAAATTACTTGGATCCTAAGATATCTGTTG
CTTGGTG
TAAAAAATATGATGTT---
CCGTTCCGGGTGATCGGCG
ATAACCTGAAGGACCGTTTCGACGGCGCCTCACGTGTAATGCTCAGCAACACAGCGAACGCACGTAACACAAA
CAGTCCG
CACGC---GAACAGGC---
CGAAGCAGGACAAGCTCAGCAACAGTATCTCCTCGAATAGCATCCACAGTAAGAGGGAGAA
CCGGCCCAAGAAGTACAAGTACGGATTCCAGCTGAAACCGTACAATCCGGACCACAAGCCGCCCAGTCCGAAG
GACTTGG
TGTACTTGGAGCCRTCGCCAGGGTTTTGCGAGAGGAATCCGAAACTAGGTATCCAGGGTACGCACGGGAGGCA
A-TGTAA

CGACACATCGATAGGGGTAGACGGGTGTGATCTGATGTGCTGCGGTAG-GGGATACAGGAC-
CAGGAGGTGGTCGTCGTG
GAGAGATGCAACTGCACGTTCCACTGGTGCT
>Domene
GGGAGAAGCCCAGCACTGAATCCCGTGGCCGAACCGGGAAATGTAGTGTTTGGGAGGGTCCGTCATCCATCGT
ACGACGC
GTCCAAGTCCTTCTTGAACGGGGCCACATACCCATAGAGGGTGCCAGGCCCGATAGCTGGAGGATCTCTCCTC
AGAGTCG
GGTTGCTTGAGAGTGCAGCCCTAAGTGGGTGGTAAACTCCATCTAAGGCTAAATATGACCACGAGACCGATAG
CGAACAA
GTACCGTGAGGGAAAGTTGAAAAGAACTTTGAAGAGAGAGTTCAATAGTACGTGAAACCGTTCAGGGGTAAAC
CTGAGAA
ACCCGAAAGGTCGAATGGGGAGATTCAGCGTGTCTCGTTTTTGGTCGCGTGACGATGGTGCTCGCCACGGGCC
GCGCCTT
CCGAATCCGCAACCTGTTACGAACTCGTGCACTTCTCCCCTAGTAGGACGTCGCGACCCGTTGAGTGTCGGTC
TAAGGCC
GAGGGTGGAGCCTTGAGGTCCCGGCCGGCTCGCTCGACGGTAAGACAGAGACGTGGGGTCGCGAAGTTCGCGT
CCGGCCC
GTTACAAGCTCGGGCGACTCGGACGTCGGACCTGTGTGCCGACCTCGAGCGCGCCGGCTGTTGGTAACGGTGT
CCTCGGA
CAGACTACACGTCGGTCGGCGACGCTTTAGCTTTGGGTTTTCAGGACCCGTCTTGAAACACGGACCAAGGAGT
CTAGCAT
GTGCGCGAGTCATTGGGACCGCATCTAAACCTAAAGGCGAAATGAAAGTGAAGGCGTGCCGAGGGAGGATGGG
TCGGGGG
GCGTCTCGTTCTCATCGCGAGATGAGGCGCACCCAGAGCGTACACGCTCTTACACCGTATTCGCTGATTTGTT
CGA-TCC
CATCATTGAAGACTACCATAGTGGATTCAAGAAGACCGACAAGCATCCTCCCTCAAACTGGGGCGATGTCAAC
ACTTTCG
TCAATCTTGACCCTGCTGGTGAATACGTTGTGTCTACTCGCGTACGTTGCGGCCGCTCTATGGAAGGCTACCC
CTTCAAC
CCCTGCTTAACCGAAGATCAATACAAGGAGATGGAACAGAAGGTTTCGACCACTTTGTCTGGACTCGAGGGCG
AACTTAA
GGGTACCTTCTACCCATTAACTGGAATGGATAAGGATACTCAACAGAAGCTCATCGATGACCATTTCTTGTTC
AAGGAAG
GAGATCGTTTCCTCCAAACTGCTAACGCTTGCCGTTATTGGCCATCTGGACGTGGTATCTACCATAACGACAA
CAAAACA
TTCTTGGTCTGGTGCAACGAAGAGGATCATCTCCGTATCATCTCCATGCAGATGGGTGGTGATCTTGGCGAAG
TCTACCG
TCGTCTTGTCACAGCTGTCAACGAAATGGAGAAGCGCGTACCCTTCTCCCACAATGACAGATTAGGTTTCCTT
ACCTTCT
GCCCAACTAACTTGGGTACAACTGTACGTGCCTCTGTACACATCAAAGTACCTAAGCTCGCTGCCAACAAGGC
TAAGCTC
GATGAAATTGCTGGCAAGTACAACTTGCAAGTACGTGGTACTCGTGGTGTTCCAAGAAAAGTCTTAATTATTG
GTTCCGG
TGGTTTATCAATTGGACAGGCTGGAGAATTTGATTATTCAGGCTCACAAGCAATTAAAGCACTTCAAGAAGTA
AACATTC
AAACTGTTTTAATAAATCCCAACATTGCAACCGTACAAACTTCTAAAGGTTTAGCTGACAAAGTTTATTTTCT
TCCTTTA
GTACCAGAGTATGTTGAACAAGTAATAAGAGTTGAAAGACCAGGCGGTGTTTTACTAACGTTTGGTGGACAAA
CAGGGTT
AAATTGTGGAGTTGAACTTCAAAGGGCTGGAATATTCGAAAAATATGGTGTTAAAATTTTGGGTACACCTATT
CAAGCAA
TTATTGATACAGAAGATAGAAAAATATTTAGTGATAGAATAGCATTAATTGGAGAAAAGGTAGCACCAAGTAT
GGCTGCA
TATTCCGTACAAGAAGCTTTGGAAGCAGCAGAATTGTTAGGATATCCTGTTATGGCAAGAGCAGCTTTTTCAT
TAGGAGG

ATTAGGTTCTGGATTTGCTAATTCCGCAGAAGAATTAAAATCGCTAGCTCAGCAAGCATTGGCACATTCCAAT
CAGTTAA
TTATTGATAAGTCTTTGAAAGGATGGAAAGAAGTTGAATACGAAGTAGTGCGAGATGCACTTCACAACATTAA
ACAAGTT
AACGATGAAGAATTAACGGAACCGACTGATAAGAGAATGTTTGTTGTTGCTGCTGCTTTAAAAAGTGGTTATA
GTATTGA
TAAATTGTACGATTT-
AACAAAAATTGATCGTTGGTTTTTACAAAAAATGAAAAACATTATAGACCATACTACTTTATTA
GAATCAACTGAACAAATTAAATTA---------------
ACAGCTAAAATTTTGTTGAAGGCCAAGAAAATTGGATTTAG
TGATAAGCAGATTGCAGCAGCATGTAAAAGTACTGAGCTTGCAATCAGAAAACAACGTCAAGATTTTAATATT
ACTCCAT
GTGTTAAACAGATAGATACTGTAGCTGCTGAATGGCCAGCTACTACAAATTACTTATATTTAACGTATAATGC
ATCATGT
CATGATATAACATTTATTGATGAGCATATAATGGTTATTGGATCTGGAGTTTATAGAATTGGGAGTTCTGTTG
AATTTGA
TTGGTGTGCTGTTGGATGTTTACGAGAGCTTAGAAAATTAAATAAAAAGACAATAATGGTAAATTATAATCCA
GAAACTG
TGAGCACAGATTATGATATGTCAGATAGATTGTACTTTGAGGAAATTTCATTTGAAGTTGTAATGGATGAAGT
ATACATT
TTAATTTTACCTGGATTTGGATTAATTTCTCACATTATTAGACAAGCTAGAGGAAAAAAAGAAACATTTGGAA
CTCTAGG
AATAGTTTATGCAATAATAGCAATTGGGCTATTAGGATTTATAGTATGAGCTCATCACATATTCACTGTTGGA
ATAGATG
TGGATACTCGAGCTTATTTTACTTCAGCAACCATAGTTATTGCTATCCCTACTGGAATTAAAATTTTTAGCTG
ATTAGCA
ACATTACATGGAACAAAAATTATTTTTAATCCCCCAATAATTTGGGCATTAGGATTCGTATTCTTATTTACAA
TTGGTGG
ACTAACTGGAGTAATTCTAGCAAATTCATCAATTGATATTGTATTACACGACACTTATTATGTTGTAGCACAT
TTCCATT
ACGTACTTTCTATAGGAGCTGTGTTTGCAATTATAGCTGGTTTAGTTCAATGATTTCCATTATTTACTGGACT
AACCTTA
AACAAAAAATGATTAAAAATTCAATTTTTCATCATATTTACTGGAGTAAACTTAACCTTTTTCCCACAACATT
TTCTAGG
AATAGCAGGTATACCTCGACGATACTCAGATTACCCTGATGCCTATACTACCTGAAATGTAATCTCATCAATT
GGATCGT
TAATCTCAATAATAGGGGTTTTTTTATTGCTATTCATTTTATGAGAAAGATTTTCTGCAAAACGAATAATTTT
GTCAGCA
AAAAATTTTGTGACTTCAATTGAATGATATCAACTTTACCCACCAGCAGAACATAGATATAACGAACTCCCAA
TATTAGT
AAAAGACTACGTAGTCGTTTTTGATTTTCTCGGT-
AAAGATTCCATTAGATATTACAATGAAGTACCTGTGGAGAAACGT
GTATTCAAAAACCTCCAGTTGTTCATGGAAAATAAAGCGAAAGCGGACGATTTATTTGATCGCTTAAACACAG
CTGTTAT
GAACAAGCATTTAAACGAGCTCATGGAAGGTTTAACCGCCAAGGTATTTCGTACTTACAATGCTTCTTGGACG
TTGCAAC
AACAACTCGAGAAGCTGACCAATGAAGACGATTCCATATCTGAGAAAATTTTATCATATAACCGTGCCAATAG
GGCTGTC
GCCATTCTGTGTAACCATCAACGTGCTGTTCCTAAAGGCCATCAGAAATCTATGGAGAAGCTAAAGGAGAAAA
TCGATTC
TAAGCGAGATAACATCAAGGATGCAGAGCGGCAGGTTAAGGATGCACAGAAGGACGCTAAGCA---
CGGTAGTGTAAAGG
AGAAGATGGTTTACGACAAGAAAAAGAAGATGTTGGAGAGGCTGCGCGATCAATTAGCGAAATTGGAGATTCA
AGAGACG
GATCGTGACGAGAATAAAACTATTGCGCTTGGTACGTCTAAGTTGAACTATTTGGATCCGAGAATTTCGGTCG
CCTGGTG

TAAGAAGTACGATGTGCCCCTTGAGAAAATTTA-------------
TGGATGCGGCTTCCGCCTTTCAGAGTAATCGGAG
ATCACCTAAAGGACCGTTTCGACGGTGCTTCACGTGTGATGCTCAGCAACTCTGCTAGTTCCAGAAG------
----
--------CAATCGTC---
CCAAGCAGGACAAGCTTAGCAACAACATAGCATCCAACAGCATACATAGCAAGCGCGAGAA
TCGTCCGCGAAAATATAAATATGGCTTCCAACTGAAACCTTACAATCCCGATCATAAGCCTCCCAGTCCTAAA
GACTTGG
TGTATTTGGAGCCGTCGCCTGGTTTCTGCGAGAAGAACCCGAAGCTTGGCATACAGGGTACGCATAGTARGCG
ANTGCAA
CGATACTTCTATAGGTGTCGATGGTTGCGATTTGATGTGTTGCGGAAGNAGGTTACAGGACCCAGGAAGTCAT
TGTTGTG
GAGAGGTGTAACTGCACGTTCCACTGGTGCT
>Dysanabatium
GGGAGGAGCCCAGCACAGAATCCCGTGGCCGAACCGGGAAATGTTGTGTTTGGGAGGGTCCGTCATCCATTGT
GCGACGC
GTCCAAGTCCTTCTTGAACGGGGCCACATACCCATAGAGGGTGCCAGGCCCGATAGCCGGAGGATCTCTCCTC
AGAGTCG
GGTTGCTTGAGAGTGCAGCCCTAAGTGGGTGGTAAACTCCATCTAAGGCTAAATACAACCACGAAACCGATAG
CGAACAA
GTACCGTGAGGGAAAGTTGAAAAGAACTTTGAAGAGAGAGTTCAATAGTACGTGAAACCGTTCAGGGGTAAAC
CTGAGAA
ACCCGAAAGGTCGAATGGGGAGATTCAGCGTGTCACGTTATCGGCCGAGTGACGATGGTGTTCGCACCGGATT
GCACCGA
CCGACTCCTAAACCGGCGGCGAACTCGTGCACTTCTCCCCTAGTAGGACGTCGCGACCCGTTGGGTGCCGGTC
TAAGGCC
AGCGGTGGAGCCTGTGGGTCCCGGCCGGCTCGCCCGACGGTATGACAGAGACGTGTGGTCGCGAAGTTCGCGT
CCGGCCC
GCCACAAGCACGCGCGACTCGAACGCCGGACCTGTGTGCCGACCTCGAGCTCGCCGGCTGTTGGTGGCGGTGT
CCTCGGA
CAGACCACACGTCGGTCGGCGACGCTTTAGCTTTGGGTTTTCAGGACCCGTCTTGAAACACGGACCAAGGAGT
CTAGCAT
GTGCGCGAGTCATTGGGATCATATCTAAACCTAAAGGCGAAATGAAAGTGAAGGCGTGCCTAGGGAGGACGGG
TCGGGGG
GCGTCTCGTTCTCATCGCGAGATGAGGCGCACCTTGAGCGTACACGC--------------------------
----
-
ATCATCGAGGATTACCATGGCGGGTTCAAGAAGACCGACAGCCACCCGCCCAAGAACTGGGGTGACGTGAACA
CCTTCG
CCAACCTCGACCCGGCCGGTGAGTACGTCGTCTCGACCCGCGTCCGTTGCGGCCGCTCCATGGAGGGCTACCC
GTTCAAC
CCGTGCTTGACCGAGGAGCAGTACAAGGAGATGGAGCAGAAGGTCTCGTCCACCCTGTCCGGCATGGAGGGCG
ACCTCAA
GGGCACCTTCTACCCGCTGACCGGCATGGACAAGGATACCCAGCAGAAGCTCATCGACGACCACTTCCTGTTC
AAGGAGG
GCGATCGTTTCCTGCAGGCGGCCAACGCGTGCCGTTTCTGGCCGTCCGGTCGCGGCATCTATCACAACGAGAA
CAAGACC
TTCCTGGTGTGGTGCAACGAGGAGGACCATCTCCGCATCATCTCCATGCAGATGGGCGGCGATCTCGGCGAGG
TATACCG
TCGCCTCGTCAGCGCCGTCAACGAGATCGAGAAGCGCGTGCCGTTCTCGCACAACGACAGGTTGGGTTTCCTC
ACCTTCT
GCCCATCCAACTTGGGCACCACTGTACGTGCCTCTGTACACATCAAAGTACCTAAACTTGCCGCCAACAAAGC
CAAGCTT
GATGAAATCGCTGCCAAATACAACTTGCAAGTACGCGGTACACGCGGTGTTCCGAAAAAAGTTTTAATTATAG
GCTCAGG
TGGTTTGTCAATCGGACAGGCGGGTGAATTTGATTATTCTGGTTCGCAGGCAATCAAAGCATTGCAAGAAGAG
AATATTC

AAACGGTTTTAATCAACCCGAACATTGCGACTGTACAAACATCGAAAGGTTTAGCCGATAAAGTTTATTTTTT
ACCGTTG
GTGCCGGAATATGTGGAGCAAGTAATTAGAGTTGAACGACCTGGCGGTGTGTTATTAACATTTGGCGGCCAAA
CAGGATT
GAATTGCGGAGTGGAACTTCAGAAAGCTGGAATTTTTGAAAAATACGATGTTAAAGTTCTCGGGACACCAATA
CAAGGTA
TTATCGATACTGAAGATCGGAAAATTTTTAGTGAAAGAATTTCAATAATCGGTGAAAAGGTAGCCCCAAGTAT
GGCTGCT
TATTCTGTTCAGGAAGCTTTGGAAGCGGCAGAGATGTTGGGATATCCAGTAATGGCCAGAGCTGCTTTCTCGT
TAGGTGG
ATTGGGATCTGGTTTCGCAAATACAGCAGATGAACTTAAATCATTAGCTCAACAGGCGTTAGCGCATTCAAGT
CAATTAA
TTATCGACAAATCTTTAAGAGGTTGGAAGGAAGTCGAATATGAAGTCGTAAGGGATGCTTATCCGTATCTTAA
AACTGTA
AATGATGAAGAATTAGAAGAACCGACGGATAAACGAATGTTTGTAGTAGCCGCTGCTTTAAGAAATGGTTATT
CCGTGGA
AAAAATATATGAATT-
AACCAAAATTGATCATTGGTTTTTGCAAAAAATGAAAAACATAATAGATTACAACACTTATTTA
GAATCAATTCAACAAAATAAATTA---------------
ACCTATAAAATCTTATTAAAGGCGAAACAAATAGGATTTAG
TGATAAACAGATTGCTGTTGCCGTTAAAAGCACTGAACTCGCTATTAGAAAACAACGGCAGGATTTTAATATC
ATACCTT
TCGTTAAACAAATTGATACAGTTGCTGCAGAGTGGCCTGCGACTACGAATTATTTGTATTTAACGTACAACGC
AGCAAGT
CATGATTTAACTTTCTCCGAAGAACATATTATGGTTATAGGTTCCGGTGTTTATAGAATAGGAAGTTCCGTTG
AATTTGA
TTGGTGTGCTGTCGGTTGTTTACGAGAATTAAGAAAATTAAATAAAAAGACAATAATGGTAAATTACAATCCG
GAAACGG
TTAGTACAGATTATGACATGTCTGACAGATTATATTTCGAAGAAATATCATTTGAAGTTGTAATGGATGAAGT
TTACATT
TTAATTTTACCAGGATTTGGAATAATTTCTCATATTGTAAGACAAAATAGAGGTAAAAAGGAAACATTTGGGA
CATTAGG
AATAATTTATGCTATAATAGCAATTGGTTTATTAGGATTTGTTGTTTGAGCACATCATATATTTACTATTGGT
ATAGATG
TTGATACTCGAGCTTATTTTACATCTGCTACAATAATTATTGCAGTACCAACTGGTATTAAAATTTTTAGATG
ATTAGCT
ACTTTACATGGAACTCAAATTAATTTTAATCCAGCAATATTATGATCATTAGGATTTATTTTTTTATTTACTA
TTGGTGG
ATTAACAGGAGTAATTTTAGCAAATTCATCTATCGATATTATTTTACATGATACTTATTATGTAGTAGCTCAT
TTTCATT
ATGTTCTTTCTATAGGAGCTGTATTTGCAATTATAGCAGGATTAGTTCAATGATTTTCTTTATTTACAGGAAT
TACATTA
AATAGTTATTTATTAAAAATTCAATTTTTTGTTATATTTACAGGTGTAAATTTAACTTTTTTCCCTCAACATT
TTTTAGG
ATTAGCTGGTATACCTCGACGTTATTCTGACTATCCAGATGCCTATACTACTTGAAATGTAGTATCTTCTATT
GGATCTT
TTATTTCTTTAGTAAGAATTTTATTATTACTTTTTATTATTTGAGAAAGATTTACTTCAATTCGTATAGTAAT
TTCAGCC
AAAAATTATTCATCTTCTATTGAATGATATCAATTATACCCTCCTGCTGAACATAGATATTCAGAATTACCTG
CAATATC
T---

TCAATGGAAAAATTAAAGGAGAAGATTGAAGC
TAAAAAGGAATCCATCAAAGACGGTGAGCGTCAAGTTAAAGATGCTCAAAAGGATGCTAAACA---
TGGTAGTGTTAAGG
AGAAACAGATCTATGATAAGAAAAAGAAAATGTTGGAAAGACTAAAGGAACAGTTAGCAAAATTAGAAATTCA
AGAAACT
GACCGTGATGAAAATAAAACAATTGCTCTTGGCACTTCCAAGTTGAATTATCTAGATCCGAGAATCTCTGTGG
CTTGGTG
TAAGAAATATGGAGTACCCATTGA-------------------------
ATGCGCTTGCCTCCATTCAGGGTAATCGGCG
ATCATCTAAAGGACCGCTTCGACGGCGCTTCCCGCGTCATGCTCAGCAATTCGGCGAGCTCCAGGGGC-----
----GGT
AACTC---GAACCGCC---
CCAAGCAGGACAAAATGTCGAACAACATCGCTTCGAACAGTATCCACAGCAAGCGGGAGAA
CAGGCCGCGCAAGTACAAGTACGGCTTCCAGCTGAAACCGTACAACCCCGACCACAAGCCGCCCAGTCCGAAG
GACTTGG
TGTACCTCGAGCCGTCGCCCGGTTTCTGCGAGAAAAATCCGAAGCTCGGCATCCAGGGCACGCACGGGAGGCA
G-TGCAA
CGATACGTCGATAGGTGTAGACGGCTGCGACCTGATGTGCTGCGGAAG-
AGGTTACAGGACCCAGGAAGTCATTGTGGTG
GAGAGGTGCAACTGCACTTTCC---------
>Echiaster
GGGAGAAGCCCAGCACTGAATCCCGTGGTCGAACCGGGAAATGTAGTGTTTGGGAGGGTCCATTATCTATCGT
GCGACGC
GTCCAAGTCCTTCTTGAACGGGGCCACATACCCATAGAGGGTGCCAGGCCCGATAGCTGGAGGATCTCTCCTT
AGAGTCG
GGTTGCTTGAGAGTGCAGCCCTAAGTGGGTGGTAAACTCCATCTAAGGCTAAATATGACCACGAGACCGATAG
CGAACAA
GTACCGTGAGGGAAAGTTGAAAAGAACTTTGAAGAGAGAGTTCAATAGTACGTGAAACCGTTCAGGGGTAAAC
CTGAGAA
ACCCGAAAGGTCGAATGGGGAGATTCAGCGTGTCTCGTTTCTGGTCGCGTGACGAAGGTGCTTGCACCGGCCT
GCGCCTT
CCGGATCCGTAACCGGCGGCGAACTCGTGCACTTCTCCCCTAGTAGGACGTCGCGACCCGTTGGGTGCCGGTC
TAAGGCC
GACGGTGGAGCCTCGGGGTCCCGACCGGCCCGCTCGACGGTAAGACAGAGACGTGGGGTCGCTTCGTAAGCGT
CCGGCCC
GTCACAAGTCCGTGCGCCTCGGACGTCGGACCTGTGTGCCGACCCCGAGCTCGCCGGCTGCTGGTGACGGTGT
CCTCGGA
CAGACTACACGTCGGTCGGCGACGCTTTAGCTTTGGGTTTTCAGGACCCGTCTTGAAACACGGACCAAGGAGT
CTAGCAT
GTGCGCGAGTCATTGGGACCGCATCTAAACCTAAAGGCAAAATGAAAGTGAAGGCGTGCCGAGGGAGGATGGG
TCGGGGG
GCGTCTCGTTCTCATCGCGAGATGAGGCGCACCCAGAGCGTACACGCTCTTACACCGTATTTGCCGACTTGTT
CGA-TCC
CATCATTGAGGATTACCATGGTGGATTCAAGAAGTCCGACAAGCACCCACCTAAGAACTGGGGTGATGTAAAC
ACTTTCG
GCAACCTCGACCCAGCTGGTGAATATGTAGTATCCACCCGTGTACGTTGCGGCCGCTCCATGGAAGGTTACCC
GTTCAAT
CCGTGCTTGACCGAAGAACAATACAAAGAAATGGAAGGTAAAGTGTCCAGCACCTTGTCTGGTCTCGAAGGAG
AACTCAA
GGGTACTTTCTATCCGTTGACCGGTATGGACAAAGCGACCCAACAGAAGCTCATCGATGATCACTTYTTATTC
AAGGAAG
GCGACCGTTTCCTTCAGGCTGCGAACGCCTGCCGCTTCTGGCCATCTGGACGTGGAATCTACCACAACGACAA
CAAAACC

TTCTTGGTCTGGTGCAACGAAGAAGATCATCTCCGCATTATCTCCATGCAGATGGGCGGCGACCTTGGCGAGG
TCTACCG
TCGCCTTGTGACCGCCGTCAACGAAATTGAGAAGCGCGTTCCCTTTTCGCACAATGACAGGTTAGGTTTTCTT
ACCTTCT
GCCCAACCAACTTGGGCACAACTGTACGTGCCTCTGTACACATTAAAGTACCTAAGCTCGCAGCCAATAAGGC
CAAACTT
GACGAAGTCGCTGCCAAATATAACTTGCAAGTACGCGGTACCCGCGGTATTCCTAAAAAAGTTTTAATTATCG
GTTCCGG
AGGTTTGTCTATAGGACAAGCCGGGGAATTCGATTATTCAGGTTCACAAGCAATAAAAGCTTTACAAGAAGAA
AACATAC
AAACAGTCCTGATTAACCCCAATATAGCAACTGTACAAACATCAAAAGGTTTAGCTGATAAAATTTACTTTCT
GCCATTG
GTGCCTGAATTCGTTGAACAAGTGATAAGAGCAGAACGTCCTGGCGGTGTTTTGCTGACATTTGGTGGACAAA
CAGGGTT
GAATTGCGGTGTGGAACTACAAAGAGCAAATGTATTTGAAAAGTAYGGTGTTAAAATTTTGGGTACACCTATA
CAGGCTA
TAATTGATACAGAAGACAGAAAAATTTTTAGTGAAAGAATTGCTTTGATTGGAGAAAAAGTTGCGCCAAGTAT
GGCTGCA
TATTCTGTTCAGGAAGCACTGGAAGCAGCGGAATTGTTAGGATATCCTGTTATGGCAAGAGCTGCGTTTTCTT
TAGGTGG
TTTAGGATCAGGGTTTGCTAATAACGATGAAGAACTGAAAGCACTTGCTCAACAAGCTTTAGCTCATTCGAAT
CAATTAA
TTATTGATAAGTCCTTGAAAGGATGGAAAGAAGTTGAATATGAAGTTGTACGTGATGCTAAT-----------
----

GAAGTTTATATT
TTAATTTTACCCGGGTTTGGTTTAATTTCCCATATTTTTAGACAATCTAGAGGAAAAAAAGAAACTTTTGGTT
CATTAGG
AATAATTTATGCAATAATAGCTATCGGGTTACTAGGGTTTATTGTATGGGCCCATCATATATTTACTGTCGGG
ATAGATG
TTGATACCCGGGCCTACTTTACTTCAGCAACCATAATTATTGCAGTACCTACAGGAATTAAAATTTTTAGTTG
ATTGGCT
ACATTTCATGGGACTCAAATTAAATTTGATCCTCCAATATTATGAGCTTTAGGATTTGTATTTTTATTTACTT
TAGGGGG
ATTAACTGGAGTTATTTTAGCTAATTYTTCTATTGATATTGTACTTCATGATACCTATTATGTTGTAGCTCAT
TTTCATT
ATGTATTATCAATAGGGGCTGTATTTGCAATTATAGGAGGATTAGTTCAATGATTCCCTTTATTTACAGGATT
AGCATTA
AACAATTATTGATTAAAAATTCAATTTTTAATTATATTTATTGGAGTGAATTTAACATTTTTTCCTCAACATT
TTCTTGG
ATTAGCAGGAATACCTCGCCGATATTCAGATTATCCTGATTYTTATATACCTTGAAATACTATTTCCTCTATT
GGATCAA

TTATTTCTATAGTAAGAATTTTTTTTTATTATTTATTATTTGAGAAAGATTCATTACTATGCGAATAAATAT
TATACCA
AAAAATTTTTCTACATCTATTGAATGACTCCAATTAACTCCACCTGCTGAACACAGATWTTCTGAGTTACCAA
TACTAAC
T----------------------------CTTGGT-
AAGGATTCTATTAGATATTATAATGAAGTACCTGTGGAAAAACGT
GTCTTCAAAAACCTTCAATTGTTCATGGAAAACAAATCTCCAGGTGATGATTTATTTGATAGATTGAACACAG
CAGTAAT
GAACAAACATTTAAACGAGTTAATGGAAGGTTTAACTGCCAAGGTATTTCGTACTTATAACGCTTCTTGGACT
CTACAAC
AACAACTAGATAAATTAACCAATCCAAATGATTCCATATCAGAGAAAATATTATCATACAATCGAGCAAATAG
AGCAGTA
GCTATACTTTGTAACCATCAACGTGCAGTACCTAAAGGTCACCAAAAATCCATGGAAAAACTTAAAGAAAAAA
TTGATGC
CAAAAAAGAAGCTATTAGAGATGGTGAACGACAAGTTAAAGATGCACAAAAAGATGCGAAACG---
TGGAAGTGTCAAGG
AGAAACAAATTTATGACAAGAAGAAGAAAATGTTGGACAGACTCAAAGAACAGTTAGCAAAATTGGAAATCCA
GGAGACA
GACCGTGACGAAAATAAAACTATTGCCCTCGGTACTTCCAAGCTGAACTATTTGGATCCCAGAATTTCGGTTG
CTTGGTG
CAAGAAGTTTGATGTGCCCATTGAAAAAATCTA-------------
GGGATGCGCTTGCCACCCTTCCGAGTGATCGGCG
ACCATTTGAAAGACCGCTTCGACGGCGCCTCCAGGGTGATGCTGAGCAATTCGGCGAGTTCTCGCGG------
------G
AACGC---CAACCGGC---
CCAAGCAGGACAAACTCTCCAACAGCATCGCGTCGAACAGCATCCACAGCAAAAGGGAGAA
CCGCCCCCGCAAGTACAAGTACGGCTTCCAATTGAAGCCGTACAATCCGGACCACAAGCCTCCGAGTCCGAAA
GATCTGG
TGTACCTGGAGCCGTCGCCTGGATTCTGCGAGAAGAACCCGAAGCTTGGGATACAGGGCACGCATGGTAGATT
G-TGCAA
CGATACTTCTATAGGTGTGGACGGTTGCGATTTGATGTGCTGCGGTAG-
GGGCTACAGGACGCAGGAAGTCATCGTGGTA
GAAAGGTGCAACTGCACGTTCC---------
>Enallagium
GGGAGAAGCCCAGCACTGAATCCCGTGGCCGAACCGGGAAATGTAGTGTTTGGGAGGGTCCGTTATCCATCGT
GCGACGC
GTCCAAGTCCTTCTTGAACGGGGCCATATTCCCATAGAGGGTGCCAGGCCCGATAGCTGGCGGATCTCTCCTC
AGAGTCG
GGTTGCTTGAGAGTGCAGCCCTAAGTGGGTGGTAAACTCCATCTAAGGCTAAATATGACCACGAGACCGATAG
CGAACAA
GTACCGTGAGGGAAAGTTGAAAAGAACTTTGAAGAGAGAGTTCAATAGTACGTGAAACCGTTCAGGGGTAAAC
CTGAGAA
ACCCGAAAGGTCGAATGGGGAGATTCAGCGTGTCTCGTGTTCGGTCGCGTGACGATGGTGCTTGCACCGGGCT
GCGCCTT
CTGAATCCGTAACCGGCGACGAACTCGTGCACTTCTCCCCTAGTAGGACGTTGCGACCCGTTGGGCGCCGGTC
TACGGCC
GACGGTGGAGCCTTGGGGTCCCGGCCGGCCCGCTCGACGGTAAGACAGAGACGTGGGGTCGCGATGTTCGCGT
CCGGCCC
GTCACAAGTACGGGCGACTCGGACGTCGGACCTGTGTGCCGACCTCGAGCTCGCCGGCTGTTGGTGACGGTGT
CCTCGGA
CAGACTACACGTCGGTCGGCGACGCTTTAGCTTTGGGTTTTCAGGACCCGTCTTGAAACACGGACCAAGGAGT
CTAGCAT
GTGCGCGAGTCATTGGGACCGCATCTAAACCTAAAGGCGAAATGAAAGTGAAGGCGTGCCGAGGGAGGATGGG
TCGGGGG
GCGTCTCGTTCTCATCGCGAGATGAGGCGCACCCAGAGCGTACACGC--------------------------
----

CATGGTGGATTCAAGAAGACCGACAAGCACCCCCCTTCCAACTGGGGAGATGTCAACACTTTCG
TCAACCTTGACCCTGCTGGCGAATACGTTGTATCTACTCGCGTACGTTGCGGCCGCTCCATGGAGGGCTACCC
MTTCAAC
CCCTGCTTAACCGAAGATCAATACAAGGAGATGGAACAGAAGGTTTCCACCACTTTGTCTGGACTCGAGGGTG
AACTTAA
GGGTACCTTCTACCCATTGACCGGAATGGATAAGGACACCCAACAGAAGCTCATCGATGACCATTTCTTGTTC
AAGGAAG
GAGATCGTTTCCTCCAAACTGCTAACGCTTGCCGTTATTGGCCGTCTGGACGTGGTATCTACCATAACGACAA
CAAAACA
TTCTTGGTCTGGTGCAACGAAGAGGATCATCTCCGCATCATCTCCATGCAGATGGGTGGTGATCTTGGTGAAG
TCTACCG
TCGCCTTGTCACAGCCGTCAACGAAATTGAGAAGCGCGTACCCTTCTCTCACAATGACAGATTAGGTTTCCTT
ACCTTCT
GCCCAACTAACTTAGGTACAACTGTACGTGCCTCTGTACACATCAAAGTACCTAAGCTCGCTGCCAACAAGGC
TAAGCTT
GATGAGATTGCTGGAAAGTACAACTTGCAAGTCCGTGGTACTCGTGGTGCTCCTAAAAAAGTTTTAATTATTG
GTTCTGG
TGGTTTATCGATTGGTCAAGCTGGAGAATTTGATTACTCTGGTTCACAAGCTATTAAAGCTTTACACGAAGAA
AATATTC
AAACTGTATTAATAAATCCTAATATCGCCACAGTGCAAACATCAAAAGGTTTAGCTGATAAAATATATTTCTT
ACCGTTA
GTGCCTGAATTCGTTGAACAAGTAATTAGAGTTGAAAGACCCGGTGGTGTTTTATTAACTTTTGGTGGCCAAA
CAGGTTT
GAATTGTGGTGTAGAATTACAAAAAGCTGGTGTTTTTGAAAAATATGGTGTAAAAATTTTGGGTACTCCAATT
CAGGCCA
TAATTGACACTGAAGACCGGAAAGTTTTCAGTGATAGAATAGCACAAATTGGTGAGAAAGTTGCTCCAAGTAT
GGCTGCT
TATTCCGTACAGGAAGCTTTGGAAGCTGCTGAATTATTAGGTTACCCAGTAATGGCAAGAGCTGCATTCTCCT
TAGGTGG
TTTGGGATCTGGATTTGCTGATACTGCCGATGAATTGAAATCACTTGCTCAACAGGCTTTGGCTCATTCTAAT
CAATTAA
TTATAGATAAATCATTGAAAGGTTGGAAGGAAGTTGAATATGAAGTTGTTAGAGATGCGTATCCTTACTTAAA
ACAAGTA
AATGATGAAGAGTTAAAAGAACCCACTGATAAACGGATGTTTGTACTGGCAGCTGCTTTAAGAAATGGTTATA
CTATTGA
TAAACTTTATGATTT-
AACAAAAATAGACCGCTGGTTTTTACAAAAAATGAAAAATATTGTTGATTTCAATACCCATCTA
GAATCAATT---CAAAATAAGTTA---------------
ACGTATAAACACTTATTGAAAGCGAAACAAATTGGTTTCAG
TGATAAACAAATTGCTGTTGCAGTAAAAAGTACTGAACTTGCTGTTAGAAAACACCGTCAAGATTTTGAAATT
ACTCCAT
ATGTTAAGCAAATAGATACTGTAGCAGCTGAATGGCCAGCAACAACAAATTATCTATATTTAACTTATAATGC
TGAAAGT
CATGATATAACTTTTTCCGATGAGCATATTATGGTAATTGGATCAGGTGTATATAGAATTGGAAGTTCAGTGG
AATTCGA
TTGGTGTGCAGTAGGTTGTTTACGAGAACTTAGAAGGTTAAATAAAAAAACAATAATGGTCAATTACAATCCA
GAAACAG
TTAGTACTGACTACGATATGTCTGATAGATTATATTTCGAAGAAATTTCATTTGAAGTTGTAATGGAT-----
----

AAAAAAGGAAACATTGGGGCTTTAGG
AATAATTTATGCAATAATAGCAATTGGCTTGTTAGGATTCGTTGTATGAGCTCATCATATATTTACAGTAGGA
ATAGATG
TTGACACTCGTGCCTATTTTACATCAGCAACTATAATTATTGCTGTTCCAACAGGAATCAAAATTTTTAGATG
ATTAGCT

ACTCTTCATGGAACTCAAATTAAATACACCCCCCCTATATTATGGGCTTTAGGATTTGTATTTCTTTTTACAA
TCGGGGG
ATTGACTGGAGTGATTTTAGCTAACTCATCTATTGATATTATTCTTCATGATACCTATTATGTAGTCGCTCAT
TTTCACT
ATGTTCTATCAATAGGGGCTGTTTTTGCTATTATAGCAGGCCTAGTTCAATGATTCCCCCTATTTACCGGATT
AACTTTA
AATGAATATTTTCTAAAAATTCAATTTTTTATAATATTTATTGGTGTTAATTTAACCTTTTTCCCCCAACATT
TTTTAGG
ATTAGCAGGAATGCCTCGTCGTTATTCTGATTACCCCGATGCCTATACTCCCTGAAATGTTATTTCATCAATT
GGGTCCC
TAATCTCAATAGTTAGAATTTTTTTTTTATTATTTATTATTTGAGAAAGATTTTCCTCAATACGATTAGTTAT
TTCATCT
AAAAACTTTTCAACTTCAATTGAATGATACCAA---

-AAGAATTACGTAGTAGTATTCGATTTCCTTGGT-
AAAGATTCCATTAGATATTACAATGAAGTACCTGTTGAGAAACGA
GTTTTTAAAAACCTCCAATTATTCATGGAAAACAAATCTCCAGGAGACGACTTGTTTGATAGATTAAACACAG
CTGTGAT
GAACAAACATTTAAACGAGTTAATGGAAGGTTTAACCGCCAAGGTGTTTCGTACTTACAATGCGTCTTGGACC
TTACAAC
AACAACTCGATAAACTGACCAATCCAGACGATTCCATATCCGAGAAAATTCTTTCCTACAACCGAGCAAACCG
TGCGGTC
GCCATCCTCTGTAACCATCAACGTGCGGTACCGAAAGGTCATCAAAAATCGATGGAGAAATTAAAGGAAAAAA
TCGAAGC
TAAAAAGGAAAATATACGTGACGGTGAACGGCAAGTTAAGGACGCTCAGAGAGACGCAAAGCA---
CGGTAGCGTTAAGG
AGAAACAGATTTACGAGAAGAAGAAAAAGATGTTGGAGAGACTTAGAGAACAATTAGCTAAATTAGAGATACA
AGAGACG
GACCGCGATGAAAATAAAACTATTGCTCTCGGCACGTCAAAGTTGAATTATTTGGATCCGAGGATTTCGGTTG
CTTGGTG
TAAGAAGTTTGATGTGCCCATTGAAAAGATTTATAATAAAACTCAC---------------------------
----

GCTGGCGAACAGCATCTCATCGAATAGCATCCACAGCAAGCGCGAGAA
CAAGCCGCGCAAATACAAATACGGATTCCAATTAAAACCGTACAATCCCGACCACAAGCCGCCTAGTCCAAAA
GATCTGG
TGTACTTGGAGCCGTCGCCCGGTTTTTGCGAGAGGAATCCAAAATTGGGCATACAGGGCACACACGGCAGACA
A-TGCAA
CGACACTTCCATCGGAGTTGACGGCTGCGATTTGATGTGCTGCGGAAG-
AGGCTACAGGACGCAAGAAGTAGTCGTTATC
GAGAGGTGCAACTGCACCTT-----------
>Eustilicus
GGGAGAAGCCCAGCACTGAATCCCGTGGCCGAACCGGGAAATGTAGTGTTTGGGAGGATCCACTATCCATCGT
GCGACGC
GTCCAAGTCCTTCTTGAACGGGGCCACATACCCATAGAGGGTGCCAGGCCCGATAGCTGGAGGATCTCTCCTC
AGAGTCG
GGTTGCTTGAGAGTGCAGCCCTAAGTGGGTGGTAAACTCCATCTAAGGCTAAATATGACCACGAGACCGATAG
CGAACAA
GTACCGTGAGGGAAAGTTGAAAAGAACTTTGAAGAGAGAGTTCAATAGTACGTGAAACCGTTCAGGGGTAAAC
CTGAGAA
ACCCGAAAGGTCGAATGGGGAGATTCAGCGTGTCTCGTTTCTGGTCGAGTGACGATGGTGCTTGCACCGGGTT
GCGCCGT
CCGGATCCGTATCCGGCGACGAACTCGTGCACTTCTCCCCTAGTAGGACGTCGCGACCCGTTGGGCGCCGGTC
TAAGGCC

GACGGAGGAGCCTTGGGGTCCCGGCCGGCCCGCTCGACGGTAAGACAGAGGCGTGGGGTCGCTACGTTAGCGT
CCGGCCC
GCCACAAGTTCGGGCGACTCGGATGTCGGACCTGTGTGCCGACCTCGAGCTCGCCGGCTGCTGGTGGCGGTGT
CCTCGGA
CAGACTACACGCCGGTCGGCGACGCTCTAGCTTTGGGTTTTCAGGACCCGTCTTGAAACACGGACCAAGGAGT
CTAGCAT
GTGCGCGAGTCATTGGGACCGCATCTAAACCTAAAGGCTAAATGAAAGTGAAGGCGTGCCGAGGGAGGATGGG
TCGGGGG
GCGTCTCGTTCTCATCGCGAGATGAGGCGCACCCAGAGCGTACACGC--------------------------
----

CTTCAAAAAGGGCGACAAGCACCCGCCCAAGAACTGGGGCGACGTGAACACCTTCG
CCAACCTGGACCCCGCCGGCGAGTACGTGGTGTCCACCCGCGTCCGCTGCGGCCGCTCCATGGAGGGCTACCC
CTTCAAC
CCGTGCTTGACCGAGGAGCAGTACAAGGAGATGGAGGCGAAGGTGTCCGGCACCCTGTCCGGCCTCGAGGGCG
AGCTCAA
GGGCACCTTCTACCCGCTGACCGGCATGGACAAGGACACCCAGCAGAAGCTGATCGACGACCACTTCCTGTTC
AAGGAGG
GCGACCGCTTCCTGCAGACCGCCAACGCTTGCCGCTTCTGGCCGTCCGGCCGTGGTATCTACCACAACGACAA
CAAGACC
TTCTTGGTCTGGTGCAACGAAGAGGACCACCTGCGCATCATCTCGATGCAGATGGGTGGCGATCTTGGCGAGG
TCTACCG
TCGCCTCGTGACCGCCGTCAACGAGATCGAGAAGCGCGTCCCGTTCTCCCACAATGACCGATTGGGTTTCCTC
ACCTTCT
GCCCGACCAACCTGGGCACAACTGTACGTGCCTCTGTACACATCAAAGTACCTAAGCTCGCCGCCAACAAGGC
CAAGCTC
GACGAGGTCGCCGGCAAGTACAACTTGCAGGTACGCGGCACTCGCGG-
GTACCTAAAAAGGTTCTAATAATTGGTTCAGG
TGGTTTATCCATTGGACAGGCCGGAGAGTTCGATTATTCCGGTTCACAAGCTATCAAAGCTTTGCAAGAAGAA
AATATAC
AAACAGTACTAATTAATCCAAACATAGCTACTGTACAAACATCAAAGGGTTTAGCTGATAAAGTATATTTCTT
ACCTTTA
GTGCCTGAATTCGTAGAACAAGTAATTAGAGTGGAACGTCCTGGTGGTGTTCTATTAACATTTGGTGGTCAAA
CAGGGTT
AAATTGTGGTGTAGAGTTACAAAAGGCTGGTATATTTGATAAATATGGTGTTAAAATTTTGGGTACACCAATA
GAAGCTA
TAATAGATACTGAAGATAGAAAGATTTTTAGTGAGAGAATATCATTAATTGGTGAAAAAGTTGCTCCAAGTAT
GGCCGCT
TATTCAGTACAAGAAGCTTTGGAAGCGGCCGAGTTATTAGGGTATCCAGTTATGGCAAGAGCTGCATTTTCTT
TAGGTGG
ATTAGGATCCGGATTTGCTAATACAGCTGAAGAACTGAAATTACTTGCTCAACAAGCTTTAGCTCATTCCAAC
CAGTT--

CCTTACCTGAAAGAAGTT
AATGATGATGAATTACAAGAACCTACAGATAAAAGAATGTTCGTTCTAGCAGCAGCTTTAAGAAATGGCTACA
GTGTAGA
TAAGTTATATGATTT-
AACAAAAATTGATCGTTGGTTCCTACAAAAAATGAAGAATATTATAGATTTCAACACTCTCCTT
GAAAAAGTTCAACAAAATAAATTACAGAATTGCTCAAATACATATAAGCTTCTATTAAAAGCAAAACAAATTG
GTTTCAG
TGATAAACAAATTGCTGTTGCTGTTAAAAGTACTGAGCTTGCAATTAGAAAGCAAAGACAAGATTTCGGTATT
ACTCCCT
ATGTTAAACAAATTGATACTGTGGCTGCTGAATGGCCTGCAACTACAAATTATCTATATTTAACGTACAATGC
AGAAAGT
CATGATCTAACTTTCAGTGATCAGCATATAATGGTTATTGGATCAGGAGTTTACAGAATTGGAAGTTCTGTTG
AGTTTGA

TTGGTGTGCTGTGGGGTGTTTGAGGGAGCTTAGAAAATTAAATAAGAAGACAATAATGGTTAATTACAACCCG
GAAACTG
TGAGTACGGATTATGATATGTCAGATAGGTTGTACTTCGAGGAAATATCTTTCGAAGTTGTTATGGAT-----
----

AATTTCTCATATTATTAGTCAATCAAGTGGTAAAAAAGAAACTTTTGGAACTTTAGG
AATAATTTATGCAATAATAGCTATTGGATTATTAGGATTTGTTGTTTGAGCTCATCATATATTTACAGTTGGA
ATAGATG
TTGATACTCGAGCTTATTTCACATCAGCTACAATAATTATTGCTGTTCCAACAGGAATTAAAATTTTTAGTTG
ATTAGCC
ACCCTTCATGGAACCCAAATTAAATTTACTCCACCAATATTATGATCATTAGGATTTGTATTTTTATTTACTA
TTGGAGG
ATTAACAGGAGTAATCTTAGCTAATTCATCAATTGATATTATCCTTCATGATACTTATTACGTAGTAGCCCAT
TTCCATT
ACGTCTTATCAATAGGGGCAGTATTCGCTATTATAGCCGGATTAGTGCAATGATTCCCATTATTTACTGGATT
AACTTTA
AATGAATTTATACTTAAAATTCAATTTTTTATCATATTTATTGGAGTTAATTTAACATTTTTCCCTCAACACT
TTTTAGG
ATTAGCTGGAATACCTCGTCGTTACTCTGATTATCCTGATGCTTATACTCCTTGAAATACTATTTCATCTATT
GGATCTC
TAATTTCTATAATTAGAATTTTCTTTTTATTATTTATTATCTGAGATAGATTTATCTCCATACGAATAAATTT
ATCTG--

-AAAGACTATGTGGTCGTATTTGATTTCCTCGGT-
AAGGATTCTATTAGATATTATAATGAAGTACCTGTGGAAAAGCGT
GTCTTCAAGAATCTCCAATTGTTCATGGAAAATAAGTCGCCTGCAGATGATTTGTTCGATAGATTAAACACAG
CTGTGAT
GAATAAACATTTAAACGAGTTAATGGAGGGCTTAACTGCCAAGGTGTTTCGTACTTACAACGCTTCCTGGACT
TTGCAAC
AGCAGCTCGACAAATTGACCAACCCAGATGAATCCATATCCGAAAAAATTTTATCATACAACCGTGCCAACAG
AGCAGTA
GCTATACTTTGCAACCATCAGCGCGCTGTGCCCAAGGGCCACCAGAAGTCCATGGAGAAACTCAAGGAGAAGA
TCGAGGC
CAAGAGGGACAACATTAAGGACGCCGAGAGGCAGGTTAAGGACGCTCAAAGGGATGCCAAGCA---
CGGCAGCGTCAAGG
AGAAACAGATCTACGAGAAGAAGAAGAAGATGCTGGAGAGACTCAGAGAGCAGTTGGCCAAATTGGAGATCCA
GGAGACT
GACCGCGACGAAAACAAAACCATTGCCCTCGGCACGTCCAAGCTGAACTATTTGGACCCTAGGATCTCGGTCG
CCTGGTG
CAAGAAGTTTGGTGTGCCCATTGAAAAGATTTATAACAAAAC-----
GGATGCGGCGGCCGCCGTTCCGCGTGATCGGCG
ACCACCTGAAGGACCGCTTCGACGGCGCGTCGCGCGTCATGCTCAGCAACTCGGCCAGCTCGCGCGG------
------C
AACGC---GAACCGGC---
CCAAGCAGGACAAGCTGTCGAACAGCATCGCCTCGAACAGCATACACAGCAAGCGGGAGAA
CAGGCCGCGCAAGTACAAGTACGGCTTCCAGCTGAAGCCGTACAACCCGGACCACAAGCCGCCCAGCCCGAAG
GACCTGG
TGTACCTGGAGCCGTCGCCCGGCTTCTGCGAGAAGAACCCGAAACTCGGCATACAGGGCACGCACGGCAGACT
G-TGCAA
CGACACGTCCATCGGCGTCGACGGCTGCGACCTGATGTGCTGCGGCAG-
GGGCTACCGGACGCAGGAGGTGATCGTCGTC
GAGCGGTGCAACTGCACGTT-----------
>Hyperomma_bicoloripes
GGGAAAAGCCCAGCACTGAATCCCGCGGCCGAGCCGGGAAATGTAGTGTTAGGGAGGGTCCGCTATCCGTCGC
GCGGCGC

GTCCAAGTCCTTCTTGAACGGGGCCACTTACCCACAGAGGGTGCCAGGCCCGATCGCGGGAGGATCTCTCCTC
AGAGTCG
GGTTGCTTGAGAGTGCAGCCCTAAGCGGGTGGTAAACTCCATCTAAGGCTAAATATCACCACGAGACCGATAG
CGAACAA
GTACCGTGAGGGAAAGTTGAAAAGAACTTTGAAGAGAGAGTTCAATAGTACGTGAAACCGTTCAGGGGTAAAC
CTGAGAA
ATCCGAAAGGTCGAATGGGGACATTCAGCGCGTCTCGGCTTAGGTCCGGAGACGGCGGCGTTCGCGTTCCGCC
GCTCCGA
CCGAAGCCGCGACCGCTCCCGAACGCGTGCACTTCTCCCCTAGTAGGACGTCGCGACCCGTTGTGTGCAGGTC
ACAGGCC
CGCGAGGGAGCCCGCCGGTCCCGACCTGCTCGCACGACGGTATAACGGTGGCGAAGGGTCGCGAAGTTCGCGT
CCGGCCC
GTCGCAAGCACAGGCGACGGGGACGTCGGACCTATGTGCCGACGCCCCGCACGCCGGCTGTTGGCGACGGTGT
CCTCGGA
CGGACCACACGCCCGTCGGCGACGCTTTAGCTTTGGATTTTCAGGACCCGTCTTGAAACACGGACCAAGGAGT
CTAGCAT
GTGCGCGAGTCATTGGGACCGCATCTAAACCTAAAGGCGAAATGAAAGTGAAGGCGTGCCGAGGGAGGACCGG
TCCGGGG
GCGTCTCGTTCTCATCGCGAGATGAGGCGCACCCAGAGCGTACACGC--------------------------
----
CATTATTGTGGTYTACYTTACTGGATTCTCGAACATAGAMAAGAHTSCAGCCRAGAACTGCAGTGATGTCAAC
GTCTTCA
CCAASCTTGACCCTGCCGGCGAGTACGTCGTGTCCACGCGCGTCCGTTGCGGCCGWTCCATGGAAGGTTACCC
CTTCAAC
CCCTGCTTAACCGAGGACYAATACAAGGAGATGGAACAGAAAGTTTCCAYCACCCTGTCCGGTCTCGAGGGAG
AGCTCAA
GGGCACCTTCTACCCACTGACTGGYATGGACAAGGACACCCCA------------------------------
----

ATTCCGAAAAAGGTGCTGATCATTGGTTCAGG
TGGTTTATCGATTGGACAGGCTGGAGAGTTTGATTATTCTGGATCGCAGGCGATCAAGGCGTTGCAGGAAGAA
AATATCC
AAACTGTTTTAATTAATCCGAATATAGCAACTGTGCAGACTTCCAAAGGTTTGGCTGATAAGGTGTATTTTTT
GCCTTTG
GTGCCTGAATATGTTGAGCAGGTGATTAGAGCTGAACGACCTGGAGGTGTTTTATTGACATTCGGTGGACAAA
CAGGTTT
GAATTGCGGCGTGGAACTGCAAAAGGCTGGGATTTTTGAGAAATACGGTGTGAAGATCCTAGGAACTCCGATC
CAGGCAA
TTATCGATACTGAGGATCGCAAGATTTTCAGTGAAAGAATCGGATTGATTGGGGAAAAGGTTGCTCCAAGCAT
GGCTGCG
TATTCAGTTCAAGAAGCCCTGGATGCAGCTGAACAATTGGGTTATCCAGTGATGGCCAGAGCTGCATTTTCCC
TAGGTGG
CCTTGGATCTGGATTTGCGAATACTGCAGAGGAATTAAAATCGTTGTCGCAGCAGGCTTTGGCCCATTCGAAC
CAGTTGA
TAATCGATAAATCGCTGAAGGGCTGGAAGGAGGTTGAATATGAAGTTGTACGTGACGCTTTTCCGTACATAAA
GGAAGTT
AAYGACGAAGAATTRAAAGAACCGACGGACAAAMGAATGTTTGTGGTAGCGGCTGCCCTGCGGAACGGTTACA
GCGTCGA

TAAATTATACAGCTT-
AACCAAAATCGACCGATGGTTCCTTCAAAAAATGAAGAACATAATCGATTTCAATACTCTACTA
GAATCGATACACCAGCACAAATTA---------------
ACTGGCGAGACTTTGCTCAAAGCCAAACAAATCGGTTTCAG
CGATAAGCAAATTGCGGCTGCAGTTAAAAGCACGGAACTGGCAATCAGAAAGCAGAGACACGATTTTGGAATC
ACACCGT
TTGTMAAACAGATCGATACGGTTGCAGCCGAATGGCCTGCGACCACGAATTATCTGTATTTAACATACAACGC
GTTGAGC
CACGATTTGGAATTTGCCGAAGAGCACACAATGGTCATTGGTTCGGGTGTTTATCGAATTGGCAGYTCRGTAG
AGTTCGA
TTGGTGCGCCGTCGGTTGTCTAMGAGAGTTGAGAAACTTGAATAAAAAGACCATCATGGTCAATTACAACCCC
GAAACGG
TCAGTACAGATTATGATATGTCCGATCGGTTGTACTTCGAAGAGATTTCGTTTGAGGTCGTGATGGAC-
AGGTGTATATT
TTAATTCTTCCAGGATTTGGTATAATTTCCCATGTAATTTGTTATAGAAGAGGTAAACCTGAAACATTTGGTA
CACTAGG
GATAATTTATGCAATATTAGCAATTGGGTTATTAGGATTTATTGTTTGAGCTCACCACATATTTACAATTGGA
ATAGATA
TTGATACTCGTGCTTATTTTACTTCAGCAACAATAGTAATTGCAGTTCCAACTGGAATCAAGGTTTTTAGTTG
AATAGCT
ACAATTTATGGAGGAAATATTAGCTTTAGACCCCCAATAATATGATCCCTAGGTTTTATTTTTCTTTTTACAG
TAGGGGG
GTTAACAGGAGTAATCTTGGCTAATTCATCTATTGATATCATTTTACATGATACTTATTACGTTGTAGCCCAC
TTTCATT
ATGTTTTATCTATAGGAGCTGTATTTGCAATTATAGCAGGATTAGTCCAATGATTTCCCCTATTCGTAGGTCT
AACCTTA
AATGAAAAATACCTAAAAATTCAATTTTTAGTGATATTTATTGGTGTAAATTTCACATTTTTCCCTCAACATT
TCTTAGG
ATTATCTGGTATACCCCGCCGATACTCTGACTATCCAGATGCATATACTACATGAAATGTAGTTTCATCTTCT
GGATCAA
TAGTTTCTTTTATTGGAATCATAACTTTTTTATGAATTATTTGGGAAGCACTAATTTATAAGCGTCAAGTTAT
TTTTATA
CCTGCCCTTCCCACAGCTATTGAATGAATACATTTTTTCCCCCCTGCCGAACATACATATAATGAACTTCCCA
TAATTAG
A---GACTACGTGGTCGTGTTCGATTTCCTCGGC-
AAAGATTCCATTAGGTATTACAACGAGGTGCCGGTCGAGAAGCGC
GTGTTTAAAAATCTGCAGCTGTTCATGGAGAACAAGTCGCCGGGCGACGATCTGTTCGACCGTCTGAACACCG
CCGTGAT
GAACAAACATTTGAACGAGCTCATGGAGGGGCTCACGGCCAAAGTGTTCCGTACTTACAATGCGTCGTTCACT
CTGCAGC
AGCAACTGGACAAATTGACCAACGAGGACGATTCCCTCTCGGAAAAGATACTGTCSTACAACAGGGCCAACAG
GGCGGTG
GCCATCCTGTGCAACCATCAGCGGGCCGTCCCCAAGGGCCACCAGAAGTCGATGGAGAAGCTCAAGGAGAAGA
TCGACGC
CAAAAGGGACGCGATCAAGGACGGGGAGCGGCAGGTGAAGGACGCCCACAGGGACTCGAAGCA---
CGGCAGCGTGAAAG
AGAAGCAGATCTACGACAAGAAGAAGAAGATGCTGGAGAGGCTAAAGGATCAGCTGGCCAAGTTGGAGATCCA
GGAGACG
GACCGCGACGAAAACAAGACCATCGCCCTCGGCACGTCGAAATTGAATTATCTGGACCCTAGGATATCTGTCG
CATGGTG
CAAAAAGTACGACGTCCCGATCGAGAAGATCTACAACAAAACCCAA------
AGATTACCTCCTTTCAGAGCCATTGGCG
ATAATCTCAAAGACCGCTTCGATGGTGCATCGCGGGTCATGCTGACCAACTCCGCCAGTTCGAG---------
------A
AACAG---CAACAGGC---
CGAAACAGGATAAGCTCAGCAACAGCATTGCCTCTAACAGCATCCACAGCAAAAGAGAGAA

CCGTCCCCGCAAGCACAAGTACGGTTTCCAGCTGAAACCGTACAACCCAGACCACAAGCCTCCGAGCCCCAAA
GATCTCG
TGTACCTAGAACCATCACCGGGCTTCTGCGAGAGGAATCCCAAGTTGGGCATCCAGGGTACCCACGGCAGGCA
G-TGTAA
CGATACTTCGATCGGGGTGGACGGTTGTGACCTGATGTGTTGCGGGAG-
AGGGTATCGCACCCAGGAGGTCCTGGTGGTC
GAGCGGTGCAACTG-----------------
>Lathrobium_brunnipes
GGGAGAAGCCCAGCACTGAATCCCGCGGCCGAGCCGGGAAATGTAGTGTTTGGGAGGGTCCGTCATCCATCGT
ACGACGC
GTCCAAGTCCTTCTTGAACGGGGCCACATACCCATAGAGGGTGCCAGGCCCGATAGCCGGAGGATCTCTCCTC
AGAGTCG
GGTTGCTTGAGAGTGCAGCCCTAAGTGGGTGGTAAACTCCATCTAAGGCTAAATATGACCACGAGACCGATAG
CGAACAA
GTACCGTGAGGGAAAGTTGAAAAGAACTTTGAAGAGAGAGTTCAATAGTACGTGAAACCGTTCAGGGGTAAAC
CTGAGAA
ACCCGAAAGGTCGAATGGGGAGATTCAGCGTGTCTCGTTTTTGGTCGCGTGACGTGGGCGTTCGCGCCGGTTC
GCGCCCT
CCGAATCCGCAACCCGCGGCGAACTCGTGCACTTCTCCCCTAGTAGGACGTCGCGACCCGTTGGGCGCCGGTC
TAAGGTC
GGGGGTGGAGCCTCTGGGTCCCGGCCGGCACGCTCGACGGTAGGACAGAGACGTGGGGTCGCGACGTTCGCGT
CCGGCCC
GTCACAAGCTCGGGCGTCTCGGACGTCGGACCTGTGTGCCGACCTCGAGCACGCCGGCTGTTGGTGGCGGTGT
CCTCGGA
CAGACTACACGTCGGTCGGCGACGCTTTAGCTTTGGGTTTTCAGGACCCGTCTTGAAACACGGACCAAGGAGT
CTAGCAT
GTGCGCGAGTCATTGGGACCGCATCTAAACCTAAAGGCGAAATGAAAGTGAAGGCGTGCCGAGGGAGGATGGG
TCGGGGG
GCGTCTCGTTCTCATCGCGAGATGAGGCGCACCCAGAGCGTACACGC-
CTTACACCGTATTCGCTGACTTGTTCGA-CCC
CATCATCGAAGACTACCATGGTGGATTCAAGAAGACCGACAAGCATCCCCCCTCCAACTGGGGTGACGTAAAC
ACTTTCG
TCAACCTTGACCCTGCCGGTGAATACGTCGTGTCAACTCGCGTACGTTGCGGCCGCTCCATGGAAGGTTACCC
CTTCAAC
CCCTGCTTAACCGAAGATCAATACAAGGAGATGGAAGGGAAGGTTTCCACCACTCTCTCCGGACTCGAGGGTG
AACTTAA
GGGTACCTTCTACCCATTGACCGGAATGGACAAGGATACTCAACAGAAATTGATCGATGACCATTTCTTGTTC
AAGGAGG
GAGATCGTTTCCTCCAGACCGCCAACGCTTGCCGTTATTGGCCATCTGGACGTGGTATCTACCACAACGACAA
CAAAACA
TTCTTGGTCTGGTGCAACGAAGAAGATCATCTCCGTATCATCTCCATGCAGATGGGTGGTGATCTTGGTGAGG
TCTACCG
TCGCCTCGTCACGGCCGTCAACGAAATTGAGAAGCGCGTACCATTCTCTCACAATGACAGATTAGGTTTCCTC
ACTTTCT
GCCCAACCAACTTGGGCACAACTGTACGTGCCTCTGTTCACATTAAAGTACCTAAGCTCGCCGCCAACAAGGC
CAAGCTT

GTTCCAAAAAAAGTTTTAATAATCGGGTCTGG
TGGTTTATCAATTGGTCAAGCTGGAGAATTCGACTAYTCAGGTTCCCAAGCAATTAAAGCACTTCAAGAGGAA
AATATTC
AAACGGTCTTAATAAATCCTAACATTGCAACTGTACAAACATCCAAAGGTTTGGCCGATAAAGTTTACTTTCT
TCCGTTA
GTCCCGGAATACGTGGAGCAAGTCATTAGAAGTGAAAGGCCTGATGGTGTATTACTGACATTCGGAGGACAGA
CAGGGTT
AAATTGCGGTGTGGAGCTTCAGAAGGCYGGTGTTTTCGAAAAGTATGGAGTTAAAATTTTGGGTACACCTATT
GAGGCAA

TCATCGATACTGAAGATAGAAAAATATTTAGTGATAGAATAGCTTTAATCGGAGAGAAGGTAGCACCCAGCAT
GGCCGCT
TATTCAGTTCAAGAAGCTTTGGAAGCAGCTGAATTATTAGGATATCCTGTAATGGCAAGAGCTGCCTTTTCCT
TGGGTGG
ATTAGGTTCAGGATTTGCGGACACCGCAGAAGAGTTAAAATCACTTGCACAACAAGCACTTGCACATTCCACT
CAGTTAA
TTATTGATAAGTCTTTGAAAGGTTGGAAAGAAGTTGAGTATGAAGTAGTAAGAGACGCTTTTCACAACATTAA
ACAAGTT
AATGATGAAGARTTAAAAGAACCCACTGATAAAAGRATGTTTGTAGTTGCCGCTGCTTTAAAAAGTGGTTATA
CTGTCGA
TAAATTATATGATTT-
AACAAAAAATTGATCGTTGGTTTTTACAAAAAATGAAGAACATTATTGACCATACTACTTTGCTA
GAGTCGACTGAACAGATTAAATTA---------------
ACAGCWAAAATTTTGTTGAAAGCAAAGCAAATTGGATTTAG
TGATAAGCAAATTGCCGCAGCTTGTAAAAGCACTGAACTTGCAATTAGAAAACAACGCCAAGATTTTAATATY
ACTCCTT
ATGTTAAGCAGATAGATACTGTGGCTGCTGAATGGCCTGCTACTACAAATTACTTATATTTAACGTATAATGC
TGGAAGT
CACGATATAACTTTTACYGAGGAGCACATAATGGTTATTGGATCTGGAGTTTATAGAATTGGAAGTTCTGTTG
AATTTGA
TTGGTGTGCAGTTGGGTGTCTACGTGAGCTTAGAAAATTAAATAAAAAAACAATAATGGTGAATTACAATCCA
GAAACTG
TAAGCACTGATTATGATATGTCGGATAGGTTGTACTTTGAGGAAATCTCATTTGAAGTTGTAATGGATGAAGT
CTACATT
TTAATTTTACCTGGATTTGGAATAATTTCCCATATTATTAGCCAGGCTAGAGGTAAGAAGGAAACTTTTGGAG
CTTTAGG
AATAATTTATGCTATGATAGCAATTGGATTATTAGGGTTTGTTGTTTGAGCTCACCACATATTTACGGTAGGA
ATGGATG
TTGATACCCGAGCTTATTTTACGTCGGCAACAATAATTATTGCAGTTCCAACTGGAATCAAAATTTTTAGGTG
ATTAGCA
ACATTACATGGTACCCAAGTAAGTTTTAATCCCCCAATACTCTGAGCATTAGGATTTGTGTTTTTATTTACAA
TTGGTGG
ATTAACTGGGGTAATTTTAGCTAACTCCTCTATTGACATTATTCTCCACGATACATATTATGTAGTTGCCCAC
TTTCATT
ATGTTTTATCTATGGGTGCAGTATTCGCTATTATAGCAGGATTAGTTCAATGATTCCCCCTTTTTACTGGAGT
AACATTA
AATGATAACCTTTTAAAAATTCAATTTTTTGTAATATTTACTGGAGTAAATTTGACCTTTTTCCCACAACATT
TTTTAGG
ATTAGCTGGAATACCTCGACGATATTCAGATTATCCAGATGCCTACACAACTTGAAATGTTATTTCTTCAATT
GGATCTC
TAATTTCTATAGTTAGAATTTTCCTCCTACTATTTATTATCTGGGAAAGTTTTACTTCAATACGAAAAACACT
ATCCGCA
AAAAATTTTTCTACTTCAATTGAGTGATACCAACTTCATCCTCCTGCTGAACATAGATATAATGAATTACCAA
TATTATC
TAAGGATTATGTTGTTGTTTTCGATTTYCTGGGT-
AAAGATTCCATTAGATATTATAATGAAGTACCKGTGGAGAAACGT
GTCTTCAAGAACCTCCAGTTGTTCATGGAGAACAAAGCGAAAGCCGACGATCTATTCGACAGACTAAACACGG
CCGTGAT
GAACAAGCATTTAAACGAACTCATGGAGGGTTTGACCGCCAAGGTATTTCGTACTTACAACGCCTCTTTTACG
CTGCAAC
AGCAACTCGACAAGCTGACCAACGCCGATGATTCCATATCTGAAAAGATTCTATCGTACAACCGTGCCAATAG
GGCCGTT
GCCATTCTGTGTAACCATCAACGTGCTGTGCCCAAGGGCCATCAAAAAATCCATGGAGAAGTTAAAGGAGAAAA
TCGACGC
CAAAAGGGAGAACATAAGAGACGCAGAGCGGCAGGTTAAAGATGCGCAGAAGGACGCAAAGCG---
TGGAAGCGTCAAGG

AGAAGCAGATCTACGACAAGAAGAAGAAGATGTTGGAGCGGCTTAAGGAACAACTTGCCAAGTTGGAGATCCA
AGAGACG
GATCGCGACGAAAATAAAACCATCGCACTCGGTACGTCCAAGTTGAACTATCTGGACCCGAGAATCTCGGTCG
CCTGGTG
TAAGAAGTTCGATGTGCCCATTGAAAAGATTTATAACAAAACTCAA------
CGCCTTCCACCCTTCCGGGTGATCGGAG
ATCACCTGAAGGACCGCTTCGACGGGGCCTCCCGTGTAATGCTGAGCAACTCCGCTTCCTCCAGGGG------
------C
AACGC---GAATCGTC---
CAAAGCAAGACAAGCTCTCCAACAACATCGCCTCCAACAGCATCCACAGCAAGCGCGAGAA
CCGCCCGCGCAAATACAAGTACGGCTTCCAACTCAAGCCCTACAACCCCGATCATAAACCTCCAAGTCCTAAG
GACCTTG
TATACCTGGAGCCTTCACCCGGTTTCTGCGAGAAGAACCCCAAACTGGGTATACAAGGCACGCACGGCAGACA
G-TGCAA
CGATACTTCCATAGGTGTCGACGGTTGCGATCTGATGTGTTGCGGAAG-
AGGTTACAGAACCCAGGAGGTCATAGTGGTG
GAGAGATGCAACTGCACG-------------
>Lithocharis
GGGAGAAGCCCAGCACTGAATCCCGTGGCCGAACCGGGAAATGTAGTGTTTGGGAGGGTCCGCTATCCACCGT
GCGACGC
GTCCAAGTCCTTCTTGAACGGGGCCACATACCCATAGAGGGTGCCAGGCCCGGTAGCTGGAGGATCTCTCCTT
AGAGTCG
GGTTGCTTGAGAGTGCAGCCCTAAGTGGGTGGTAAACTCCATCTAAGGCTAAATATGACCACGAGACCGATAG
CGAACAA
GTACCGTGAGGGAAAGTTGAAAAGAACTTTGAAGAGAGAGTTCAATAGTACGTGAAACCGTTCAGGGGTAAAC
CTGAGAA
ACCCGAAAGGTCGAATGGGGAGATTCAGCGTGTCTCGTTTTTGGTCGAGTGACGATGGTGTTCGCACTGGGC-
GCGCCTT
CCGAATCCGTAACCGGCAACGAACTCGTGCACTTCTCCCCTAGTAGGACGTCGCGACCCGTTGGGTGCCGGTC
TAAGGCC
GACGGAGGAGACTTGGGGTCCCGGCCGGCCCGCTCGACGGTAAGACAGAGGCGTGGGGTCGCTACGTTAGCGT
CCGGCTC
GTCACAAGTTCGAGTGACTCGGATGTCGGACCTGTGTGCCGATCTCGAGATCGCTGACTGTTGGTGACGGTGT
CCTCGGA
CAGACTACACGCCGGTCGGCGACGCTCTAGCTTTGGGTTTTCAGGACCCGTCTTGAAACACGGACCAAGGAGT
CTAGCAT
GTGCGCGAGTCATTGGGACCGCATCTAAACCTAATGGCAAAATGAAAGTGAAGGCGTGCCGAGGGAGGATGGG
TCGGTGG
GCGTCTCGTTCTCATCACGAGATGAGGCGCACCCAGAGCGTACACGC--------------------------
----

CATGGTGGATTCAAGAAGACCGACAAACACCCTCCCAAGAACTGGGGTGATGTAAACACCTTTG
GTAACCTCGACCCAACCGGTGAATACGTCGTATCCACCCGTGTCCGCTGCGGTCGCTCCATGGAAGGTTATCC
ATTCAAC
CCATGCTTAACCGAAGAGCAATACAAGGAGATGGAAGCTAAAGTCTCAAGCACTTTGTCTGGACTCGAAGGCG
AACTTAA
GGGTACTTTCTACCCATTGACCGGAATGGACAAGGCTACTCAACAGAAGCTCATCGATGACCACTTCTTGTTC
AAGGAAG
GAGATCGTTTCCTCCAGGCTGCCAACGCCTGCCGTTTCTGGCCATCTGGACGTGGTATCTACCACAACGACAA
CAAGACC
TTCTTGGTCTGGTGCAACGAAGAAGATCACCTTCGCATTATTTCCATGCAAATGGGCGGTGATCTTGGTGAAG
TATACCG
TCGCCTTGTAACCGCTGTCAACGAAATCGAGAAGCGTGTACCCTTCTCCCACAATGACAGATTAGGTTTCCTC
ACTTTCT
GCCCAACCAACTTGGGCACAACTGTACGTGCCTCTGTACACATTAAAGTACCTAAGCTCGCCGCCAACAAAGC
CAAACTC

GATGAAGTCGCTGCCAAATACAACTTGCAAGTACGTGGCACCCGCGGTGTTCCTAAAAAAGTATTGATAATTG
GATCAGG
AGGTTTATCTATTGGACAAGCAGGTGAATTTGATTAYTCAGGTTCACAAGCTATAAAAGCATTACAAGAAGAA
AACATCC
AAACAGTATTGATCAATCCAAATATTGCTACTGTACAAACATCRAAGGGTTTGGCTGACAAAATATACTTTTT
ACCTTTA
GTGCCTGAATTTGTAGAACAAGTGATACGAGTAGAACGCCCTGGTGGCGTTTTACTAACATTCGGTGGACAAA
CAGGGTT
AAATTGTGGTGTAGAACTACAAAAAGCAGGTGTTTTTGATAGATACAATGTTAAAATATTGGGAACACCAATA
CAAGCTA
TAATAGATACGGAAGACAGAAAAGTATTTAGCGAAAGAATTGCTTTGATTGGTGAAAAAGTTGCTCCAAGTAT
GGCTGCT
TATTCCGTACAGGAAGCATTGGAAGCAGCTGAGTTAYTAGGATATCCAGTTATGGCTAGAGCAGCTTTTTCTT
TGGGAGG
ATTRGGATCTGGTTTCGCTAATACTGCTGAGGAACTGAAACTACTTGCTCAACAAGCCTTAGCTCATTCAAAT
CAGTTAA
TTATTGATAAGTCGTTAAAAGGATGGAAGGAGGTTGAATATGAAGTTGTTAGAGATGCATATCCCTACATTAA
AGAAGTT
AATGATGATGAATTGAAAGAACCCACAGATAAAAGAATGTTTGTGTTAGCAGCAGCTCTACGTAATGGTTACA
CTGTGGA
CAAATTGTATGATTT-
GACAAAAAATTGATCGATGGTTCTTGCAGAAAATGAAAAATATTATAGATTACAATACGCTTTTG
GAATCCATACAACAAAATAAATTACAGAATAATGCRCACAATTATAAACTTTTGTTGAAAGCTAAACAAATTG
GTTTTAG
TGATAAACAAATTGCTGTTGCTATTAAAAGCACTGAGCTTGCTGTTAGAAAACTGAGGCAAGATTTTGGAATT
ACACCAT
ATGTCAAACAAATTGATACCGTTGCTGCTGAATGGCCTGCTACAACAAATTACTTGTATTTAACGTACAATGC
AGAGAGT
CACGATTTAACATTTAATGAGCAACATATTATGGTTATAGGTTCGGGTGTTTACAGAATTGGTAGTTCTGTTG
AATTTGA
TTGGTGTGCTGTTGGTTGCTTGAGAGAACTTAGGAAATTAAATAAAAAGACAATAATGGTTAATTACAACCCC
GAAACTG
TTAGTACAGATTATGATATGTCGGATAGATTATATTTTGAGGAAATATCCTTTGAAGTTGTAATGGATGAAGT
ATATATT
TTAATTCTTCCTGGATTTGGGATAATTTCTCATATTATTTCTCAAGCCAGAGGAAAAAAGGAAACATTTGGAG
CATTAGG
AATAATTTATGCTATAATAGCAATTGGATTATTAGGATTTGTAGTTTGAGCTCATCATATATTTACAGTAGGA
ATAGATG
TGGATACTCGAGCATATTTTACTTCTGCTACAATAATTATTGCAGTACCTACAGGTATTAAAATTTTCAGATG
ATTAGCA
ACTTTACATGGAACTCAAATTAAATTTACACCTCCTATACTTTGAGCTTTAGGATTTGTATTTTTATTTACTA
TTGGAGG
ATTAACTGGTGTAGTTTTAGCAAATTCTTCTATTGATATTATCCTTCATGATACTTACTATGTAGTAGCTCAT
TTTCATT
ATGTCCTTTCTATAGGAGCAGTATTTGCTATTATAGCTGGTTTAGTCCAATGATTTCCCTTATTTACTGGGTT
AACATTA
AATGAATACTTATTAAAAATTCAATTCTTTGTAATATTTGTTGGAGTGAATCTAACATTTTTCCCTCAACATT
TCTTAGG
ATTAGCTGGAATACCTCGACGATATTCAGATTATCCCGATGCATACACACCTTGAAATGTTGTTTCATCAATT
GGAAGAA
TAATTTCTATATTAAGAATTTTCTTATTATTATTTATTATTTGAGAAAGATTTGTTTCAATACGAATAAATAT
TTCTGCT
AAAAATTTTTCTACTTCAATTGAATGATTCCAGCTATTTCCCCCTGCTGAACATAGATATTCCGAATTACCAA
TATTAAC
AAAAGATTACGTAGTGGTATTTGATTTCCTTGGT-
AAGGATTCCATTAGATATTATAATGAAGTACCTGTAGAAAAACGT

GTCTTCAAGAACCTTCAATTATTCATGGAAAATAAATCACCCGGAGATGATTTGTTTGACAGATTGAACACAG
CTGTGAT
GAACAAACATTTAAACGAATTAATGGAAGGTTTAACTGCCAAGGTGTTTCGTACTTATAACGCTTCGTGGACA
CTACAAC
AGCAACTCGATAAATTGACCAATCCAGATGATTCCATATCCGAAAAAATTTTATCATATAACCGTGCCAATAG
AGCAGTA
GCAATACTTTGTAACCATCAACGTGCTGTCCCTAAAGGCCATCAGAAATCCATGGAAAAACTCAAGGAAAAGA
TCGAAAC
TAAAAAAGACTCTATTAAAGATGCAGAGAGGCAGGTTAAAGATGCACAGAGAGATGCAAAACA---
TGGCAGCGTAAAAG
AGAAGCAGATTTACGAAAAGAAAAAGAAAATGCTGGAGAGACTTCGGGAGCAATTGGCGAAATTGGAAATTCA
AGAAACT
GACCGTGATGAAAACAAAACAATTGCGCTGGGTACCTCCAAGCTGAATTATTTGGACCCCAGAATTTCGGTTG
CATGGTG
TAAGAAGTTTGGTGTGCCCATTGAAAAAATTTATAACAAAACTCAA---------------
TCGTTCCGCGTCATCGGCG
ACCACCTGAAGGACCGCTTCGACGGCGCGTCGCGCGTCATGCTCAGCAACTCGGCCAGCTCCCGCGG------
------G
AACGC---CAACCGCC---
CCAAACAGGACAAGCTCTCCAACAGCATCGCCTCCAACAGCATACACAGCAAGAGGGAGAA
CAGGCCGAGGAAATACAAGTACGGCTTCCAGCTGAAACCCTACAATCCAGACCATAAACCTCCGAGTCCCAAG
GATCTTG
TGTACTTGGAGCCATCGCCTGGTTTCTGCGAGAAGAACCCGAAGCTGGGGATCCAGGGTACCCATGGGAGGTT
G-TGCAA
CGACACGTCGATCGGTGTGGACGGGTGCGATCTGATGTGCTGCGGNAG-
AGGGTACAGGACCCAGGAGGTCATCGTCGTT
GAGAGGTGCAAC-------------------
>Lobrathium_candicum
GGGAGAAGCCCAGCACTGAATCCCGTGGCCGAACCGGGAAATGTAGTGTTTGGGAGGGTCCGTCATCCATCGT
ACGACGC
GTCCAAGTCCTTCTTGAACGGGGCCACATACCCATAGAGGGTGCCAGGCCCGATAGCCGGAGGATCTCTCCTC
AGAGTCG
GGTTGCTTGAGAGTGCAGCCCTAAGTGGGTGGTAAACTCCATCTAAGGCTAAATATGACCACGAGACCGATAG
CGAACAA
GTACCGTGAGGGAAAGTTGAAAAGAACTTTGAAGAGAGAGTTCAATAGTACGTGAAACCGTTCAGGGGTAAAC
CTGAGAA
ACCCGAAAGGTCGAATGGGGAGATTCAGCGTGTCTCGTTTTTGGTCGCGTGACGATGGTGCTTGCACCGGGCC
GCGCCTT
CCGAATCCGTAACCCGCGACGAACTCGTGCACTTCTCCCCTAGTAGGACGTCGCGACCCGTTGGGCGCCGGTC
TAAGGCC
GAGGGTGGAGCCTCGGGGTCCCGGCCGGCACGCTCGACGGTAAGACAGTGGCGTGGGGTCGCGATGTTCGCGT
CCGGCCC
GTCACAAGCTCGGGCGACTCGGACGTCGGACCTGTGTGCCGACCTCGAGCTCGCCGGCTGTTGGTGACGGTGT
CCTCGGA
CAGACTACACGTCGGTCGGCGACGCTTTAGCTTTGGGTTTTCAGGACCCGTCTTGAAACACGGACCAAGGAGT
CTAGCAT
GTGCGCGAGTCATTGGGACCGCATCTAAACCTAAAGGCGAAATGAAAGTGAAGGCGCGCCGAGGGAGGATGGG
TCGGGGG
GCGTCTCGTTCTCATCGCGAGATGAGGCGCACCCAGAGCGTACACGCTCTTACACCGTATTCGCTGATTTGTT
CGACCCC
CATCATTGAAGACTACCATGGTGGATTCAAGAAGACCGACAAGCACCCCCCTTCCAACTGGGGAGATGTCAAC
ACTTTCG
TCAACCTTGACCCTGCTGGCGAATACGTTGTATCTACTCGCGTACGTTGCGGCCGCTCCATGGAGGGCTACCC
ATTCAAC
CCCTGCTTAACCGAAGATCAATACAAGGAGATGGAACAGAAGGTTTCCACCACTTTGTCTGGACTCGAGGGTG
AACTTAA

GGGTACCTTCTACCCATTGACCGGAATGGATAAGGACACCCAACAGAAGCTCATCGATGACCATTTCTTGTTC
AAGGAAG
GAGATCGTTTCCTCCAAACTGCTAACGCTTGCCGTTATTGGCCGTCTGGACGTGGTATCTACCATAACGACAA
CAAAACA
TTCTTGGTCTGGTGCAACGAAGAGGATCATCTCCGCATCATCTCCATGCAGATGGGTGGTGATCTTGGTGAAG
TCTACCG
TCGCCTTGTCACAGCCGTCAACGAAATTGAGAAGCGCGTACCCTTCTCTCACAATGACAGATTAGGTTTCCTT
ACCTTCT
GCCCAACTAACTTAGGTACAACTGTACGTGCCTCTGTACACATCAAAGTACCTAAGCTCGCTGCCAACAAGGC
TAAGCTT
GATGAGATTGCTGGAAAGTACAACTTGCAAGTCCGTGGTACTCGTGGTGTTCCAAAAAAAGTTTTAATAATTG
GTTCCGG
TGGTTTATCAATTGGACAAGCCGGAGAATTTGATTATTCAGGCTCGCAAGCAATTAAAGCACTTCAAGAGGAA
AATATTC
AAACTGTTTTAATAAATCCGAACATTGCAACTGTCCAAACTTCTAAAGGTTTAGCTGATAAGGTTTATTTCCT
TCCGTTA
GTACCAGAGTATGTTGAACAAGTAATTAGAGTTGAAAGACCAAGCGGTGTTTTACTAACTTTCGGCGGACAAA
CAGGGTT
AAATTGTGGCGTGGAGCTTCAAAAGGCTGGAGTTTTCGAAAAATATGGAGTTAAAATTTTGGGTACACCTATT
CAAGCAA
TCATCGATACAGAAGACAGAAAAATATTTAGCGATAGAATAGCATTAATTGGAGAAAAGGTAGCACCGAGTAT
GGCTGCG
TATTCAGTACAAGAAGCTTTGGAAGCGGCAGAATTTTTAGGTTATCCTGTTATGGCAAGAGCTGCTTTTTCTT
TG-----

CATAATATTAAGCAAGTT
AATGATGAAGAATTAAARGAACCAACTGATAAGAGAATGTTTGTTGTTGCTGCTGCTTTAAAAAGTGGTTATA
GTGTTGA
TAAATTATACGATTT-
AACAAAAATTGATCGTTGGTTTTTACAAAAAATGAAAAACATTATAGACCATACTACTTTGCTA
GAGTCGACTGAACAAATCAAACTA---------------
ACAGCTAAAATTTTGTTAAAGGCAAAGCAAATTGGATTTAG
CGACAAGCAGATTGCTGCWGCTTGCAAAAGCACTGAGCTTGCTATTAGAAAACAACGTCAAGATTTTAATATT
ACTCCAT
ATGTTAAGCAAATTGATACCGTAGCTGCTGAATGGCCTGCTACTACAAATTACTTATATTTAACTTATAATGC
GGGAAGT
CATGATTTAACGTTTGATGATGAGCATACGATGGTTATTGGATCTGGAGTTTATAGGATTGGAAGTTCGGTTG
AATTTGA
TTGGTGTGCAGTTGGATGTTTGCGTGAGCTTAGAAAATTAAATAAAAAGACAATAATGGTAAATTACAAT---
----

GAAGTTTACATT
TTAATTTTACCTGGATTCGGAATAATTTCTCATATTATCAGACAAGCAAGAGGAAAAAAGGAAACATTTGGAA
CTTTAGG
AATAATCTATGCTATAATAGCAATTGGTTTACTTGGATTTGTTGTATGAGCTCATCATATATTTACAGTAGGA
ATAGATG
TTGATACACGAGCTTATTTTACATCAGCAACTATAATTATTGCTGTTCCAACAGGAATTAAAATTTTTAGTTG
ATTAGCA
ACTTTTCATGGAACTCAGATTAATTTTAATCCGCCAATGCTTTGAGCTTTAGGATTTGTTTTTTTATTTACTA
TTGGAGG
ATTGACAGGAGTAATTTTAGCTAATTCTTCTATTGATATTATTTTACATGATACATATTATGTAGTAGCTCAT
TTTCATT
ATGTTCTTTCAATAGGAGCTGTATTTGCTATTATAGCAGGATTAGTTCAATGATTTCCTTTATTTACAGGATT
AACTTTA

AACAAAAATATACTTAAAATTCAATTTTTTGTTATATTTACAGGAGTTAATTTAACTTTTTTCCCACAACATT
TTTTAGG
TTTAGCAGGTATACCTCGACGATACTCAGATTATCCTGATGCATATACTACTTGAAATGTAATTTCATCAATT
GGATCTC
TAATTTCAATAATTGGAATTTTTTTACTATTATTTTGTGTTTGAGAAAGATTTGTTTCTATACGAAAAATTTT
ATCTGCA
AAAAATTTTACTACATCAATTGAATGATATCAATCTTATCCTCCTGCTGAACATAGTTATGATGAATTGCCCA
TA-----

TGGATGCGCCTCCCGCCGTTCAGGGTGATCGGCG
ATCACCTGAAGGACCGCTTCGACGGTGCCTCTCGCGTAATGCTCAGCAATTCGGCGAGCTCTAG---------
------G
AATTC---GAACCGTC---
CAAAGCAAGACAAGCTTAGCAACAACATAGCGTCGAATAGCATACACAGCAAAAGGGAAAA
TCGCCCGCGCAAATATAAGTACGGCTTCCAGCTTAAGCCGTACAATCCCGATCATAAGCCTCCGAGCCCCAAG
GACTTGG
TTTACTTGGAGCCGTCGCCGGGTTTCTGCGAGAAGAACCCGAAACTGGGGATACAGGGCACGCACGGAAGGCA
G-TGCAA
CGATACGTCCATAGGCGTCGACGGTTGCGACTTGATGTGCTGCGGAAG-
AGGCTACAGAACCCAGGAGGTCATAGTGGTG
GAGAGGTGCAACTGCACCTTCC---------
>Lordithon
GGGAAGAGCCCAGCACCGAATCCCGCGGCCGCGCCGGGAAATGTGGTGTTTGGGAGGGTCCGCCAGCCACCGT
GCGATGC
GTCCAAGTCCTTCTTGAACGGGGCCACTTACCCATAGAGGGTGCCAGGCCCGGTAGCGGGAGGATCTCTCCTT
AGAGTCG
GGTTGCTTGAGAGTGCAGCTCTAAGTGGGTGGTAAACTCCATCTAAGGCTAAATATGACCACGAGACCGATAG
CGAACAA
GTACCGTGAGGGAAAGTTGAAAAGAACTTTGAAGAGAGAGTTCAATAGTACGTGAAACCGTTCAGGGGTAAAC
CTGAGAA
ACCCGAAAGGTCGAATGGGGAGATTCAGCGTGTCTCGCAGCCGCTTGGGCTGCGACGCACGTTTCGGCGTGCC
TCGCCTC
CACGGCGGCCGCCTCGTCGCGAACGCGTGCACTYYT-CCCTAGTAGGACGTCGCGACCCGTT-
GGCGCCGGCCTGAGGCC
C-
CGGTNGAGCCCGTGTGTCCCAGCCGGCACGCTCGACGGTAACACGATGGCGCGGGGCCGCGACGTTCGCGTCC
GGCCC
GTCGCTGGCGCGGACGGCCTGGCGGTCGGACCTA-
GTGCCGACTCCGGGCCCGCCGCCCGCTGGCGGCGGTGACCTCGGA
CAGACTACACGCCGGTCGGCGACGCTATAGCTTTGGGTTTTCAGGACCCGTCTTGAAACACGGACCAAGGAGT
CTAGCAT

GTGCGCGAGTCATTGGGACTTATGCGAAACCTAAAGGCGAAATGAAAGTGAAGGCGTGCCGTGGGAGCAGGGG
TCGGGGG
GCGTCTCGTTCTCATTGC---

TTCAAGAAGACCGACAGCCACCCCCCGAAGAACTGGGGTGACGTAAGCGTCTTCG
CCAATCTTGATGCCGCCGGTGAATACATCGTGTCCACCCGCGTCCGTTGCGGTCGCTCCATGGAGGGCTACCC
CTTCAAC
CCCTGCTTAACCGAGGAGCAGTACAAGGAGATGGAACAGAAAGTTTCCACCACCCTCTCCGGTTTGGAAGGCG
AGCTTAA
GGGTACCTTCTACCCATTGACTGGCATGGACAAGGACACCCAGCAGAAGTTGATCGATGACCACTTCTTGTTC
AAGGAGG
GAGACCGTTTCCTCCAGACCGCCAACGCTTGCCGTTACTGGCCCAGCGGCCGTGGTATCTACCACAATGACAA
CAAGACC
TTCTTGGTCTGGTGCAACGAGGAGGATCATCTCCGTCTGATCTCCATGCAAATGGGTGGTGATCTTGGAGAGG
TCTACCG
TCGTTTGGTAACCGCCGTCAACGACATCGAGAAGCGCGTGCCATTCTCCCACAATGACAGGCTTGGCTTCCTC
ACTTTCT
GCCCCACCAACTTGGGCACAACTGTCCGCGCGTCCGTGCACATCAAGGTGCCCAAGCTCGCCGCCAACAAGGC
TAAGCTC
GATGAGATCGCTGGCAAGTACAACTTGCAAGTCCGCGGTACCCGTGGT-------------------------
----

CCGTATTTAAAAGAGGTT
AATGATGAAGAACTTACTGAGCCCACCGATAAACGTATGTTTGTAGTAGCCGCAGCGCTCCGCAGTGGATATA
CTGTAGA
TAAACTATACAACCT-
CACCAAAATCGATCGATGGTTTTTGCAGAAAATGAAGCACATTGTGGATTACAACTCGTTTTTG
GAAACCCTTAATGGGCGTAATTA---------------
ACATACGATGTGCTACTCGCTGGTAAAATGATTGGGTTTAG
TGATAAACAAATTGCATCAGCTGTTAAAAGCACTGAATTGGCTGTACGTAAGAAACGCGAGGATTTAAATCTC
AAACCGT
TTGTGAAACAAATTGATACCGTTGCCGCTGAATGGCCTGCAACTACCAATTATTTGTACCTAACATATAATGC
TGAAGAA
CATGATTTGGAATTTAATGACGAACATACAATGGTTATAGGTTCGGGGGTTTATCGTATTGGAAGCTCTGTGG
AATTCGA
TTGGTGCGCTGTGGGATGTCTTAGGGAGCTCAGAAGGTTGAATAGGAAGACGATTATGGTTAATTATAATCCG
GAGACGG
TGAGCACGGATTACGATATGTCGGATCGATTGTATTTTGAAGAAATATCGTTTGAGGTCGTTATGGAT-----
----

ATAATCTCTCATATTATTAGCCAAGAGAGTGGTAAAAAGGAAACTTTTGGGGCACTTGG

GATAATTTATGCTATAATAGCAATTGGTTTATTAGGATTTGTAGTTTGAGCCCATCACATATTTACAGTGGGA
ATAGACG
TTGATACTCGAGCTTACTTTACCTCAGCAACTATAATTATTGCAGTTCCAACTGGGATTAAAATTTTTAGATG
ACTTGCC
ACACTTCACGGAACACAAATTAACTACTCACCCTCTATATTATGGGCATTAGGATTTGTATTTTTATTTACTG
TTGGAGG
TCTAACAGGAGTTATTTTAGCTAATTCATCTATTGATATTATTCTGCATGATACCTACTATGTAGTGGCCCAT
TTTCACT
ACGTACTTTCTATAGGTGCAGTATTTGCTATTATGGCTGGATTAATCCAATGATTCCCCTTATTCACTGGATT
AACAATA
AACGAAAAGTTTTTAAAAATCCAATTTTCAGTGATATTCATTGGAGTAAACCTAACTTTTTTCCCACAACATT
TTTTAGG
ATTAGCAGGTATACCTCGTCGATACTCTGACTACCCTGATGCTTATACATCCTGAAATGTAATTTCATCAATT
GGGTCTT
TAATTTCTTTAACTAGAATTTTCTTACTCCTTTTTATTATTTGAGAAAGATTTATCTCTATACGAAAAAGAAT
TTCATCA
ATAAATTTACCTTCATCAATTGAATGATTACAAACAATACCCCCAGCTGAGCATAGTTATTCTGAATTACCGT
CCCTAAC
A--

ACCGCCGTGAT
GAACAAGCATTTGAATGAACTCATGGAGGGGTTAACTGCGAAAGTATTCCGTACGTATAACGCCTCATTCACT
CTACAGC
AGCAACTAGACAAATTAACCAACCCTGATGATTCCTTATCTGAAAAAATCTTATCCTACAATCGCGCCAACCG
AGCCGTG
GCCATCCTATGTAACCATCAACGGGCCGTGCCGAAAGGCCACCAGAAGTCCATGGAAAAACTCAAAGAGAAAA
TCGACAC
TAAAAGAGAAACCATCAGAGACGCCGAGCGATCGGTTAAAGACGCGCAAAAGGACGCCAAACG---
CGGCAGCGTAAAAG
AGAAGCAAATCTACGACAAGAAGAAGAAAATGCTAGAGCGCCTAAAGGACCAACTAGCCAAACTGGAGATCCA
AGAAACC
GATCGGGACGAAAACAAAACAATCGCGCTGGGCACGTCAAAACTGAACTATCTCGACCCGCGTATTTCGGTGG
CGTGGTG
CAAGAAGTACGATGTGCCCATTGAGAAGATCTATAATAAAACCCAG---------------------------
--CGGTG
ATAACCTAAAGGACCGCTTCGATGGTGCCTCACGAGTGATGCTAAGCAACGCGGGGCACGCACGCAA------
------C
ACAGC---GAGTCGCC---
CGAAAGCCGACAAACTGTCCAACAACATCGCCTCCAACAGCATCCACAGCAAACGCGAAAA
CCGCCCGCGCAAATACAAATACGGCTTCCAACTGAAACCCTACAACCCGGACCACAAGCCGCCCAGCCCAAAA
GACTTGG
TCTACCTAGAACCCTCCCCCGGATTCTGCGAGAAGAACCCCAAGTTGGGCATACAAGGCACACACGGGCGACA
G-TGCAA
CGACACCTCAATCGGTGTCGACGGTTGTGACTTAATGTGTTGTGGTCG-
CGGTTACCGTACGCAGGAGGTGGTTGTGGTG
GAGCGGTGCAACTGCACGTT-----------
>Medon

GAAGCCCAGCACTGAATCCCGTGGCCGAACCGGGAAATGTAGTGTTTGGGAGGGTCCGCTATCCATCGTACGA
CGC
GTCCAAGTCCTTCTTGAACGGGGCCACATACCCATAGAGGGTGCCAGGCCCGATAGCTGGAGGATCTCTCCTC
AGAGTCG
GGTTGCTTGAGAGTGCAGCCCTAAGTGGGTGGTAAACTCCATCTAAGGCTAAATATGACCACGAGACCGATAG
CGAACAA
GTACCGTGAGGGAAAGTTGAAAAGAACTTTGAAGAGAGAGTTCAATAGTACGTGAAACCGTTCAGGGGTAAAC
CTGAGAA

ACCCGAAAGGTCGAATGGGGAGATTCAGCGTGTCTCGTTTTCTGGTCAGTGACGATGGTGTTCGCACCAGGTT
GCGCGGT
CCGGAGCCGTAACCGGCGACGAACTCGTGCACTTCTCCTCTAGTAGGACGTCGCGACCCGTTGGGTGCCGGTC
TAAGGCC
GACGGTGGAGCCTTGGGGTTCCGGCCGGCCCGCTCGACGGTAAGACAGAGGCGTGGGGTCGCTACGTTAGCGT
CCGGCCC
GTCACAAGTTCGKGCGACTCGGATGTCGGACCTGTGTGCCGACCTCGAGCTCGCCGGCTGTTGGTGACGGTGT
CCTCGGA
CAGACTACACGCCGGTCGGCGACGCTCTAGCTTTGGGTTTTCAGGACCCGTCTTGAAACACGGACCAAGGAGT
CTAGCAT
GTGCGCGAGTCATTGGGACCGCATCTAAACCTAAAGGCAAAATGAAAGTGAAGGCGTGCCGAGGGAGGATGGG
TCGGGGG
GCGTCTCGTTCTCATCGCGAGATGAGGCGCACCCAGAGCGTACACGC-
CTTACACCGTATTCGCTGACTTGTTTGA-TCC
CATTATTGAAGATTACCATACTGGATTTAAGAAAACCGATAAGCACCCTCCCAAGAACTGGGGTGATGTAAAC
ACCTTCG
CCAATCTTGATCCTGCAGGTGAATACGTAGTGTCCACTCGCGTCCGTTGTGGACGCTCCATGGAAGGTTATCC
CTTCAAC
CCATGCTTAACTGAAGAGCAATACAAGGAGATGGAAACCAAAGTATCTACCACTTTGTCCGGCCTTGAAGCTG
AACTCAA
GGGTACTTTCTATCCATTGACTGGAATGGATAAAGATACTCAACAGAAGCTCATCGATGATCACTTCTTGTTC
AAGGAAG
GAGATCGTTTTCTCCAGGCTGCTAATGCCTGCCGCTTCTGGCCCTCTGGACGTGGTATTTACCACAACGACAA
CAAGACC
TTCTTGGTATGGTGCAACGAAGAAGATCATCTTCGCATCATCTCCATGCAAATGGGTGGCGATCTTGGGGAAG
TCTACCG
TCGTCTTGTAACTGCTGTTAACGAAATCGAGAAACGTGTCCCCTTCTCTCACAATGACAGATTAGGTTTCCTT
ACTTTCT
GCCCAACAAACTTGGGCACTACTGTACGCGCCTCTGTACACATCAAAGTACCTAAGCTCGCCGCTAACAAAGC
CAAGCTC

CCTTACATCAAAGAAGTA
AATGATGAAGAATTACAAGAACCCACAGATAAAAGAATGTTTGTTCTTGCAGCAGCTTTAAGAAAAGGTTACA
CAGTAGA
TAAACTCTATGATTT-
AACAAAAAATAGATCGTTGGTTCTTACAAAAAATGAAGAACATTATAGATTATAATTCCATTCTC
GAATTAGTTCAACAAAATAAATTACAAAATTGCTCAAATATTTATAAGCTGCTATTGAAAGCAAAACAAATTG
GCTTTAG
TGATAAACAAATTGCTGTTGCTGTTAAAAGTACTGAACTTGCAATTAGAAAACAACGCCAGGACTTAGGGATC
ACACCAT

ATGTTAAACAAATAGATACTGTAGCTGCTGAATGGCCGGCAACTACAAATTATTTATATTTAACATATAATGC
AGAAAGT
CATGATATAACCTTCAATGATCAGCACATGATGGTTATTGGATCAGGTGTTTATAGAATTGGAAGCTCTGTTG
AGTTCGA
TTGGTGTGCTGTAGGTTGTTTGAGGGAGCTTAGGAAATTAAACAAAAAGACAATAATGGTAAATTATAATCCA
GAAACTG
TTAGTACAGATTATGATATGTCAGATCGATTATATTTTGAAGAAATTTCATTTGAAGTTGTTATGGATGAAGT
ATATATT
TTAATTTTACCAGGATTTGGAATAATTTCCCATATTATTAGACAAGCTAGAGGAAAAAAGGAAACTTTTGGTA
GACTAGG
AATAATTTATGCTATAATAGCAATTGGATTATTAGGATTTGTAGTATGAGCACATCATATATTTACAGTGGGT
ATAGATG
TAGATACACGGGCCTATTTTACTTCAGCAACAATAATTATTGCTGTTCCAACAGGAATTAAAATTTTTAGATG
ATTAGCA
ACTTTACACGGCTCTCAAATTAAATTTACTCCACCAATATTATGATCATTAGGATTTGTTTTTTTATTCACAA
TTGGGGG
TTTGACAGGAGTTATTTTAGCTAATTCATCAATTGATATTATCTTACATGATACCTATTATGTCGTAGCTCAT
TTTCATT
ACGTTTTATCAATAGGTGCAGTTTTCGCAATTATGGCTGGATTAGTTCAATGATTCCCACTATTTACTGGATT
AACAATA
AATGAATTTTTATTAAAAATTCAATTTTTTATTATATTTACAGGAGTTAATTTAACGTTTTTCCCCCAACATT
TTCTTGG
ATTAGCGGGAATACCTCGACGATATTCAGATTACCCTGATGCTTACATACCATGAAATGCTATCTCATCAATT
GGGTCTT
TAATCTCAATAACAAGAATTTTTCTTCTATTATTTATTATTTGAGAAAGATTTACTTCCTTACGAATAAACAT
TTCAGGG
AAAAATTTTACAACTTCTATTGAATGATTTCAACAATATCCCCCAGCTGAACATAGCTATTCTGAATTACCAC
TAATAAC
TAAAGATTATGTGGTGGTATTTGATTTCCTCGGT-
AAAGATTCCATTAGATACTACAATGAAGTACCTGTAGAGAAACGT
GTCTTCAAAAACCTTCAATTGTTTATGGAAAACAAGTCGCCAGGAGACGATTTGTTTGATAGATTAAATACAG
CTGTGAT
GAACAAACATTTAAACGAGTTAATGGAAGGTTTAACTGCCAAGGTGTTTCGTACTTATAACGCTTCGTGGACT
CTACAGC
AGCAACTTGATAAATTGACCAATCCCGATGATTCCATATCCGAAAAAATTTTATCATACAACCGTGCCAACAG
AGCGGTT
GCTATACTTTGTAACCATCAGCGTGCCGTACCCAAAGGCCATCAGAAGTCTATGGAAAAATTAAAAGAGAAAA
TCGATAC
AAAAAGGGATAATATCAAGGATGCTGAGAGACAAGTTAAAGATGCACAAAGGGATGCCAAACA---
CGGAAGCGTCAAGG
AGAAGCAGATATACGACAAGAAAAAGAAAGTGCTGGAGAGACTGAGAGAGCAGTTGGCTAAGTTGGAGATTCA
GGAGACC
GACCGTGACGAAAACAAAACTATTGCGTTAGGTACGTCCAAGCTGAACTATTTGGACCCTAGAATCTCGGTCG
CCTGGTG
TAAGAAGTTTGATGTGCCCATTGAAAAAATTTATAACAAAACTCAA------
CGCCTACCACCCTTCAGAGTAATTGGTG
ACCATTTAAAGGACCGCTTCGACGGTGCTTCTAGAGTGATGCTGAGCAACTCGGCGAGCTCCCGAGG------
------A
AACGC---GAACCGTC---
CAAAGCAGGACAAGCTTTCCAACAGCATAGCGTCGAACAGCATCCACAGCAAGAGGGAAAA
CCGGCCGCGCAAATACAAGTACGGCTTCCAGCTGAAACCGTACAATCCGGACCACAAGCCGCCGAGCCCCAAG
GATCTCG
TGTACCTGGAGCCCTCGCCCGGCTTCTGCGAGAAAAACCCGAAGCTCGGCATCCAGGGCACCCACGGTAGACT
G-TGCAA
CGACACCTCGATTGGAGTGGACGGATGCGATTTGATGTGTTGCGGCAG-
GGGGTACAGGACCCAGGAAGTCATCGTGGTC
GAGAGGTGCAACTGCACG-------------

>Medonina_Russia
GGGAGAAGCCCAGCACTGAATCCCGTGGCCGACCGGGAAAATGTAGTGTTTGGGAGGGTCCATCATCCATCGT
GCGACGC
GTCCAAGTCCTTCTTGAACGGGGCCATATTCCCATAGAGGGTGCCAGGCCCGATAGCTGGCGGATCTCTCCTC
AGAGTCG
GGTTGCTTGAGAGTGCAGCCCTAAGTGGGTGGTAAACTCCATCTAAGGCTAAATATGACCACGAGACCGATAG
CGAACAA
GTACCGTGAGGGAAAGTTGAAAAGAACTTTGAAGAGAGAGTTCAATAGTACGTGAAACCGTTCAGGGGTAAAC
CTGAGAA
ACCCGAAAGGTCGAATGGGGAGATTCAGCGTGTCTCGTGTTTGGTCGCGTGACGATGGTGCTCGCACCGGACT
GCGCCTT
CTGAATCCGTAACCGGCGGCGAACTCGTGCACTTCTCCCCTAGTAGGACGTCGCGACCCGTTGGGCGCCGGTC
TACGGCC
GACGGTGGAGCCTTGGGGTCCCGGCCGGCCCGCTCGACGGTAAGACAGAGACGTGGGGTCGCGACGTTCGCGT
CCGGCCC
GTCACAAGTGCGTGCGACTCGGACGTCGGACCTGTGTGCCGACCTCGAGCTCGCCGACTGTTGGTGACGGTGT
CCTCGGA
CAGACTACACGTCGGTCGGCGACGCTTTAGCTTTGGGTTTTCAGGACCCGTCTTGAAACACGGACCAAGGAGT
CTAGCAT
GTGCGCGAGTCATTGGGACCGCATCTAAACCTAAAGGCGAAATGAAAGTGAAGGCGTGCCGAGGGAGGATGGG
TCGGGGG
GCGTCTCGTTCTCATCACGA---------------------------
TCTTACACCGTATTCGCTGATTTGTTCGA-CCC
CATCATCGAAGACTACCATGGTGGTTTCAAGAAGACCGACAAGCACCCCCCTGCAAACTGGGGTGATGTAAAC
ACCTTTG
CTAATCTCGACCCTGCTGGTGAATACGTTGTATCCACCCGCGTTCGTTGCGGTCGTTCAATGGAAGGCTATCC
CTTCAAC
CCTTGCTTAACCGAAGACCAATACAAGGAGATGGAACAGAAGGTTTCATCCACCTTGTCCGGCCTCGAAGGCG
AACTCAA
GGGTACCTTCTACCCATTGACCGGCATGAGCAAGGAGGTTCAACAGAAGCTCATTGATGATCACTTCTTGTTC
AAGGAGG
GCGATCGTTTCCTCCAGGCTGCCAACGCTTGCCGTTACTGGCCAAGCGGACGTGGTATTTACCACAACGACAA
CAAAACC
TTCTTGGTTTGGTGCAACGAAGAGGATCATCTTCGCATCATCTCTATGCAAATGGGTGGAGATCTTGGCGAGG
TATACCG
TCGTCTTGTAACCGCCGTCAACGAAATTGAGAAGCGCGTCCCCTTCTCCCATAACGACAGATTAGGTTTCCTT
ACCTTCT
GCCCAACCAACTTGGGCACAACTGTACGTGCCTCTGTACACATTAAAGTACCTAAGCTCGCCGCCAACAAAGC
CAAGCTT
GATGAAATTGCTGCCAAATACAACTTGCAAGTACGTGGTACCCGT---
GTTCCAAAAAAAAGTTTTAATAATTGGTTCTGG
TGGATTATCAATTGGTCAAGCCGGAGAATTTGATTATTCTGGTTCACAAGCAATTAAAGCTTTACACGAAGAA
AATATTC
AAACTGTTCTGATAAATCCTAACATTGCTACTGTGCAAACATCAAAAGGTTTAGCTGATAAAGTATATTTTTT
ACCTTTA
GTGCCTGAATTTGTAGAACAAGTAATTAGAGTTGAACGACCTGGTGGTGTTTTATTAACTTTTGGTGGGCAAA
CTGGATT
GAATTGTGGTGTTGAATTGCAGAGAGCTGGTATATTTGAAAAATATGGTGTTAAAATTTTGGGAACTCCAATA
GAAGCCA
TAATAGATACTGAAGATCGAAAAGTTTTTAGTGATAGAATATCACTAATTGGCGAGAAGGTAGCACCAAGTAT
GGCTGCT
TATTCAGTTCAAGAAGCTTTAGAGGCTGCTGAATTATTAGGTTATCCGGTAATGGCAAGAGCAGCATTCTCAC
TAGGCGG
TTTGGGATCTGGCTTTGCTAATACATCCGATGAATTGAAATCACTTGCTCAACAAGCATTAGCTCACTCAAAC
CAATTAA
TTATAGATAAATCATTAAAAGGATGGAAAGAAG--

GGTTTTTACAAAAAATGAAAAATATTGTTGATTTTAATACCTATTTA
GAATCAATT---CAAAACAAGTTA---------------
ACGTATAAAGATTTATTAAAAGCAAAACAAATTGGTTTCAG
TGATAAGCAAATTGCTGTAGCTGTTAAAAGCACTGAGCTTGCAATTAGAAAACACCGACAAGATTTTGACATT
ACTCCTT
ATGTGAAACAAATAGATACTGTAGCTGCTGAATGGCCTGCAACAACAAATTATCTATATTTGACTTACAATGC
TGAAAGT
CATGATTTAACTTTCTCAGATGACCATATTATGGTAATTGGTTCGGGTGTATATAGAATTGGAAGTTCTGTGG
AATTCGA
TTGGTGCGCAGTCGGTTGTTTAAGAGAACTTAGAAAGTTAAATAAAAAAACAATAATGGTCAATTATAATCCT
GAAACAG
TTAGTACTGATTACGATATGTCTGACAGATTATATTTCGAAGAAATTTCGTTTGAGGTTGTAATGGAT-----
----

TTCTCATATTATTAGTCAAGCTAGAGGAAAAAAGGAAACTTTTGGAAGATTAGG
AATAATTTATGCCATAATAGCTATTGGTCTTTTAGGTTTTGTAGTGTGAGCTCATCATATATTTACTGTTGGG
ATAGATG
TAGATACCCGAGCCTACTTTACTTCAGCAACTATAATTATTGCTGTTCCTACAGGGATTAAAATTTTTAGATG
GTTAGCA
ACATTACATGGAACTCAAATTAAATTTACTGCCCCTATATTATGAGCACTGGGATTTGTATTTTTATTTACTA
TTGGAGG
ATTAACCGGAGTAATTTTAGCTAACTCATCAATTGATATTATCCTCCATGATACATATTATGTAGTAGCCCAT
TTCCATT
ATGTATTATCAATAGGGGCGGTATTCGCCATTATAGCCGGATTAGTGCAATGATTCCCTTTATTTACAGGATT
AATAATA
AATGAATACCTATTAAAAATTCAATTTTTCATTATATTTATTGGAGTAAATTTAACATTTTTCCCTCAACATT
TTTTAGG
GTTAGCAGGAATGCCACGACGGTATTCTGATTACCCAGATGCCTATACACCATGGAATGTAATTTCTTCAATT
GGCTCAT
TAATCTCTATAATTAGAATTTTTCTATTAGTATTTATTTTCTGAGAAAGATTTTCTTCAATACGAATAATTAT
TTCTCCT
AAAAATCTTTGTACATCAATCGAATGATTTCA---

-AAAGATTACGTAGTAGTATTTGATTTCCTCGGT-
AAAGATTCCATTAGATATTATAATGAAGTACCTGTGGAGAAACGA
GTTTTCAAGAACCTCCAATTATTTATGGAAAATAAAGCTCCAGGAGATGATTTGTTCGATAGATTAAATACAG
CTGTGAT
GAACAAACATTTAAACGAATTAATGGAAGGCTTAACCGCCAAAGTGTTTCGTACTTATAACGCTTCTTGGACC
CTACAGC
AGCAACTCGAAAAACTGACCAATCCAGACGATTCCTTATCCGAAAAAATCCTCTCGTACAACCGTGCCAATCG
AGCGGTT
GCCATTCTCTGTAACCATCAGCGTGCTGTACCCAAAGGCCATCAGAAATCCATGGAAAAACTAAAAGAGAAAA
TCGAAGC
TAAAAAAGAATCCATACGTGACGGCGAAAGGCAAGTGAAGGATGCACAGAGGGATGCGAAGCA---
TGGCAGCGTTAAAG
AGAAACAGATCTACGACAAGAAAAAGAAGATGCTAGAGAGACTAAGAGACCAATTAGCTAAATTAGAGATTCA
GGAGACG
GACCGTGATGAAAATAAAACGATTGCTCTCGGCACGTCGAAGCTGAACTATTTGGATCCTAGGATCTCGGTTG
CCTGGTG
TAAGAAGTTTGATGTGCCCATTGAAAAGATTTATAATAAAACTC-----ATGCGG-
CGCCGCCGTTCCGCGTCATAGGCG
ACCACCTAAAGGACCGCTTCGACGGCGCGTCGCGCGTCATGCTCAGCAACTCGGCGAGCTCGCGGGG------
----GGC

AACGC---GAACCGTC---
CAAAGCAGGACAAACTCTCGAACAGCATCGCCTCGAACAGCATACACAGCAAGCGCGAGAA
CCGGCCGCGGAAGTACAAGTACGGTTTTCAGTTGAAACCGTACAACCCGGACCACAAGCCGCCGAGCCCCAAG
GACCTGG
TGTACCTGGAGCCGAGTCCTGGATTCTGTGAGAAGAATCCGAAGCTGGGGATACAGGGAACGCACGGGAGGCA
G-TGTAA
TGATACATCGATTGGGGTCGATGGTTGCGATTTGATGTGTTGCGGAAA--
GGGTATAGAACGCAGGAGGTTATTGTTGTT
GAAAGGTGT----------------------
>Neolindus
GGGAGAAGCCCAGCACTGAATCCCGCGGCCGGGCCGGGAAATGTAGTGTTTGGGAGGATCCGCCATCCATCGC
ACGACGC
GTCCAAGTCCTTCTTGAACGGGGCCACATACCCGCAGAGGGTGCCAGGCCCGGTAGCCGGAGGATCTCTCCTT
AGAGTCG
GGTTGCTTGAGAGTGCAGCCCTAAGCGGGTGGTAAACTCCATCTAAGGCTAAATATGACCACGAGACCGATAG
CGAACAA
GTACCGTGAGGGAAAGTTGAAAAGAACTTTGAAGAGAGAGTTCAATAGTACGTGAAACCGTTCAGGGGTAAAC
CTGAGAA
ACCCGAAAGGTCGAACGAGGAGATTCAGCGTGTTCCGTTCCGTGGCGAGTGACGGTGGCGTTCGCGCCGTGCC
GCGCCGT
CTAGGTCCGCCGCCGACGACGGACTCGTGCACTTCTCCTCCAGTAGGACGTCGCAACCCGTTGTGTGTCGATC
TACGGCC
GACGGTGGAGCCTTGGCGTCCCGACCGGCTCGCACGACGGTAGCGCGATGACGTGGGGTCGCGACGTTCGCGT
CCGGCCC
GTCACGAGCTCGGGCGACTCGAGCGTCGGACCTGCGTGCCGACCTCGGTCTCGCCGGCTGCCGGTGACGGTGT
CCTCGGA
CCGGCTACACGTCTGTTGGCGACGCTTTAGCTTTGGGTTTTCAGGACCCGTCTTGAAACACGGACCAAGGAGT
CTAGCAT
GTGCGCGAGTCACTGGGAACT-
AACTAAACCCACAGGCGAAATGAAAGTGAAGGCGCGCCGAGGGAGGATGGGTCGGGGG
GCGTCTCGTTCTCATCGCGAGATGAGGCGCACCCAGAGCGTACACGC--------------------------
----

---CCGTA-
TCTAAAAAAGTA
AACGATGAAGAATTAAAAGAGCCAACTGATAAGAGAATGTTTGTCGTTGCTGCGTTAAAATCTGGTTACA
GCGTAGA
AGAATTATATGACTT-
AACGAAAATTGATTCTTGGTTCTTGCAAAAAATGAAGAATATCATTGATTTCATAACATTTCTA
GAAGTGA---ATCAAAATCATTTA-------------
ACACCAAAAATTTTATTACAAGCTAAACAAATGGGATTCAG
TGACAAACAAATTGCTGCCGCTGCTGAAAGTACTGAACTTGCTATAAGGAAACAACGGCAAGATTATAATATA
ACACCTT
TCGTTAAGCAGATTGACACTGTCGCTGCAGAATGGCCTGCAACAACAAATKATTTATATTTGACTTACAATGC
AGGAAGT
CACGATTTGACTTTCGATGAWGAGCATGTAATGGTTATTGGATCTGGAGTTTATCGTATCGGAAGTTCTGTTG
AATTTGA
TTGGTGTGCAGTWGGATGTTTACGTGAGCTTAGAAAATNGAATAAAAAAACAATTATGGTAAATTACAATCCA
GAAACCG
TGAGTACAGATTACGATATGTCAGATAGATTATATTTTGAAGAAATTTCATTTGAAGTTGT-------
GAAGTTTATATT
CTAATTTTACCTGGATTCGGAATAATCTCTCATATTATTATACAAAGAAGAGGAAAAAAAGAAACTTTTGGAA
CCTTAGG
AATAATTTATGCAATAATAGCAATTGGATTATTAGGATTTGTAGTTTGAGCTCATCATATATTTACAGTAGGA
ATGGATG
TAGATACTCGAGCATATTTTACCTCCGCTACTATAATTATTGCTATCCCTACAGGAATCAAAATTTTTAGATG
ATTAGCT
ACTTTTCATGGAACACAAATATCATTTAACCCCCCAATAATTTGAGCTTTAGGATTTGTATTTTTATTTACAG
TAGGAGG
ATTAACTGGAGTTATTTTAGCTAATTCATCTATAGATATTATTCTACATGATACTTACTATGTTGTAGCACAT
TTCCATT
ATGTTTTATCTATAGGAGCAGTATTTGCAATTATAGCAAGTCTAACTCAATGATTTCCTTTATTTACAGGACT
ATCTTTA
AATGAATTTATACTAAAAATTCAATTTACAATTATATTTATTGGAGTTAATTTAACATTTTTCCCTCAACATT
TTCTAGG
ATTAGCAGGAATACCTCGTCGATATTCAGACTACCCTGATTCCTTTACTACTTGAAATGTAATTTCATCAATA
GGCTCAT
TAATTTCATTAATCAGAATTTTTATTATATTATTTATTATTTGAGAAAGATTAACTTCTAAACGATTAATTTT
ATCCTCA
AAAAATTTCTCTACTTCTATTGAATGATTACAATTTTT--------------------------------
----
-AAAGATTATGTCGTGGTTTTCGATTTTCTTGGC-
AAAGATTCCATTAGATATTATAATGAAGTACCTGTAGAAAAACGT
GTCTTCAAAAATCTCCGATTATTCATGGAAAATAAATCGCCAGGCGATGATCTGTTTGACAGATTGAACACAG
CTGTAAT
GAACAAACATTTAAACGAGTTAATGGAGGGATTAACCGCGAAGGTATTTCGTACTTACAACGCATCGTGGACC
TTACTAC
CGAAACATGAGAAATTGACCAATGCWGATGATTCCATATCTGAAAAAATACTGTCGTATAMTCGTGCTWATCG
TGCTGTG
GCAATYTTATGTAMCCATCAACGTGCAGTACCTAAGGGGCWTCARMAATCTATGGAAAAGCTGAAAGAGAAGA
TTGAAGC

GAAAAAAGAAGCAATAAAAGATGGTGAAAGGCAAGTAAAAGATGCGCAACGGGATGCAAAACA---
CGGTAGTGTAAAAG
AAAAGCAAATTTACGATAAAAAGAAGAAACAGTTGGAACGACTTCGCGAACAACTTGCAAAATTAGAAATTCA
AGAGACA
GACAGAGATGAAAACAAGACTATTGCACTTGGCACGTCCAAATTGAATTATTTGGACCCAAGAATCTCTGTAG
CTTGGTG
TAAGAAATACGATGTGCCCCTTGAAAAGATTTATAATAAAACTCAA---
ATGCGCCTTCCTCCATTCAGGGTAATCGGAG
ATCAACTTAAGGATCGCTTCGATGGAGCATCGAGAGTCATGCTGAGTAATTCAGCCAGCTCAAGAGGA-----
----GGT
AACGC---AAATCGTC---
CAAAACAAGACAAACTATCCAACAATATAGCATCGAATAGCATTCATAGCAAACGCGAAAA
TAGACCGCGTAAATATAAATACGGCTTCCAACTTAAACCCTATAACCCAGACCACAAACCACCAAGTGCGAAA
GATTTGG
TATACTTAGAACCATCCCCGGGATTTTGTGAAAAAAATCCRAAGTTAGGAATCCAAGGGACGCATGGCAGGCA
A-TGCAA
CGACACATCGATTGGAGTCGATGGGTGCGATTTGATGTGCTGTGGAAG-
AGGTTATAGGACACAAGAGGTAGTTGTTGTT
GAAAGATGCAACTGCACCTTCC---------
>Neosclerus
GGGAGAAGCCCAGCACTGAATCCCGTGGCCGAACCGGGAAATGTAGTGTTTGGGAGGGTCCGCTATCCATCGT
GCGGCGC
GTCCAAGTCCTTCTTGAACGGGGCCACATACCCATAGAGGGTGCCAGGCCCGATAGCTGGAGGATCTCTCCTC
AGAGTCG
GGTTGCTTGAGAGTGCAGCCCTAAGTGGGTGGTAAACTCCATCTAAGGCTAAATATGACCACGAGACCGATAG
CGAACAA
GTACCGTGAGGGAAAGTTGAAAAGAACTTTGAAGAGAGAGTTCAATAGTACGTGAAACCGTTCAGGGGTAAAC
CTGAGAA
ACCCGAAAGGTCGAATGGGGAGATTCAGCGTGTTTAGTTTCTGGTCGAGTGACGGTGGTGCTAGCACCGGGCT
GCGCCGG
CCGGGACCGCAGCCGGCGACGAACTCGTGCACTTCTCCTCTAGTAGGACGTCGCGACCCGTTGGGCGCCGGTC
TAAGGCC
GACGGAGGAGACTTGGGGTCCCGGCCGGCCCGCTCGACGGTGAGACAGAGGCGTGGGGTCGCTACGTTAGCGT
CCGGCCC
GTCACAAGTTCGGGCGACTCGGATGTCGGACCTGCGTGCCGACCTCGAGATCGCCGGCTGCTGGTGACGGTGT
CCTCGGA
CAGACTACACGCCGGTCGGCGACGCTCTAGCTTTGGGTTTTCAGGACCCGTCTTGAAACACGGACCAAGGAGT
CTAGCAT
GTGCGCGAGTCATTGGGACCGCATCTAAACCTAAAGGCGAAATGAAAGTGAAGGCGTGCCGAGGGAGGACGGG
TCGGGGG
GCGTCTCGTTCTCATCGCGAGATGAGGCGCACCCAGAGCGTACACGC------------------------
TTCGA-TCC
CATTA-
TGAAGATTACCATGGTGGATTCAAAAAGACCGACAAGCACCCCCCAAAGAACTGGGGTGATGTAAACACTTTC
G
GCAACCTTGATCCAGCTGGTGAATACGTAGTCTCCACCCGTGTCCGTTGCGGGCGCTCCATGGAAGGCTACCC
CTTCAAC
CCATGCTTAACCGAAGAACAATACAAGGAGATGGAAGGCAAAGTTTCCAGCACTCTGTCTGGCCTTGAAGCTG
AACTTAA
GGGTACTTTCTATCCCTTGACTGGAATGGACAAAGATACTCAACAGAAGCTCATTGACGATCACTTCTTGTTC
AAAGAAG
GTGATCGTTTCCTCCAAGCTGCCAACGCTTGCCGTTTCTGGCCATCTGGACGTGGTATTTACCACAACGACAA
CAAAACC
TTCTTGGTATGGTGCAACGAAGAAGATCATCTTCGTATCATCTCTATGCAAATGGGTGGTGATCTTGGTGAAG
TCTACCG
TCGCCTTGTAACAGCTGTCAACGAAATCGAGAAGCGTGTTCCCTTCTCCCATAATGACAGATTAGGTTTCTTG
ACCTTCT

GCCCAACCAACTTGGGCACAACTGTACGTGCCTCTGTACACATTAAAGTACCTAAGCTCGCCGCCAACAAGGC
CAAACTT
GATGAGGTCGCTGCCAAATACAACTTGCAAGTACGTGGTACTCGCGGTGTACCTAAAAAAGTCTTAATAATTG
GGTCAGG
TGGATTGTCAATTGGACAAGCTGGAGAATTCGATTATTCAGGTTCACAAGCAATAAAAGCTTTACAAGAAGAA
AATATTC
AAACTGTTCTAATTAATCCAAATATTGCTACAGTACAAACTTCAAAAGGTTTAGCTGATAAAATATACTTTTT
ACCTTTA
GTGCCTGAATTTGTAGAACAAGTAATTAGAGTAGAACGTCCTGGAGGCGTATTGTTAACATTTGGTGGACAAA
CAGGGTT
AAATTGTGGTGTGGAATTACAAAAAGCTGGTGTATTTGAAAAATACAATGTTCAAATTTTGGGCACACCTATA
GAAGCAA
TAATACACACCGAAGATAGAAAGATTTTTAGTGACAGAATAGCATTAATTGGAGAGAAAGTTGCTCCGAGTAT
GGCTGCA
TATTCTGTACAAGAAGCTTTGGATGCAGCAGATTTATTAGGGTATCCAGTTATGGCACGAGCAGCATTTTCTC
TGGGTGG
ATTAGGATCTGGTTTTGMTGATACAGATGAAGAATTGAAATTACTTGCTCAACAAGCTTTAGCTCATTCTAAT
CAGTTAA
TTATTGATAAGTCTTTAAAAGGGTGGAAAGAAGTTGAATATGAAGTTGTT------------
CCTAACATAAAAGAAGTC
AATGATGATGAACTAAAGGAACCTACAGATAAAAGAATGTTTGTTATTGCAGCAGCTTTAAAAAATGGTTATA
GTGTTGA
TAAATTATATGATCT-
AACAAAAAATCGATCGTTGGTTTTTACAAAAGATGAAAAATATTATAGATTACAATTCCCGTTTG
GAATTAGTTCAACAAAATAAATTACAAAATTGCTCAAATATTTACAAGCTGTTATTAAAGGCCAAACAAATTG
GTTTTAG
TGATAAGCAAATTGCTGTTGCTGTTAAAAGTACTGAACTTGCAATTCGAAAGCAAAGACAGGATTTAGGAATT
ACTCCAT
ATGTTAAACAAATTGATACAGTAGCTGCTGAATGGCCTGCAACCACAAATTATTTGTATTTAACGTACAATGC
TGAAAGT
CACGATCTAACTTTTAATGATCAACACATAATGGTTATTGGTTCGGGAGTTTATAGAATTGGGAGTTCTGTTG
AGTTTGA
TTGGTGTGCTGTTGGTTGTTTAAGAGAACTTAGAAAGTTAAATAAAAAGACAATAATGGTCAATTACAATCCA
GAAACTG
TTAGTACAGATTATGATATGTCAGATAGGCTATATTTTGAAGAAATTTCATTTGAAGTTGTTATGGATGAAGT
TTATATT
TTAATCCTCCCTGGATTTGGTATAATTTCCCACATTATTAGACAAGCTAGAGGAAAAAAAGAAGCTTTTGGAG
CTTTAGG
AATAATTTATGCAATAATAGCTATTGGTTTATTAGGATTTGTTGTTTGGGCTCACCATATATTTACTGTTGGA
ATAGACG
TAGATACTCGAGCTTATTTCACCTCAGCAACTATAATTATTGCTGTCCCTACAGGAATTAAAATTTTTAGATG
ATTAGCA
ACTTTACATGGAACACAAATTAAATACACCCCCCCTATATTATGGGCTTTAGGATTTGTTTTTTTATTTACAA
TCGGAGG
ATTAACTGGTGTTATCTTAGCAAATTCATCTATTGATATTATTTTACATGATACATATTATGTAGTAGCTCAT
TTTCATT
ATGTTTTATCAATAGGGGCTGTATTTGCTATTATAGCCGGATTAGTGCAATGATATCCCTTATTTACTGGATT
AACACTA
AATGAATACTTATTAAAAATTCAATTTTTAACTATATTTATTGGAGTAAATTTAACTTTTTTTCCTCAGCATT
TCCTAGG
ATTAGCGGGAATACCTCGACGATATTCCGATTATCCAGACGCCTATACTCCTTGAAATATAGTATCTTCTATA
GGATCAT
TGATTTCAATAAATAGAATTTTTTTACTGTTATTTATTATTTGAGAAAGAATAATTTCTTTACGAATTAATAT
TTCAGCA
AAAAATTTTCCATCATCAATTGAATGAATACAACTGTTTCCTCCAGCAGAACATACATATTCAGAATTACCAA
TTTTAAT

TAAAGATTATCTAGTAGTGTTTGATTTTCTTGGT-
AAGGATTCCATTAGATATTACAATGAAGTACCTGTAGAAAAACGT
GTTTTCAAAAACCTTCAATTGTTTATGGAAAACAAATCTCCTGGTGATGATTTGTTTGATAGATTAAACACAG
CTGTGAT
GAACAAACATTTAAACGAGTTAATGGAAGGTTTAACAGCAAAGGTGTTTCGTACTTATAACGCTTCTTGGACT
CTACAGC
AGCAACTTGATAAATTGACCAATCCAGATGATTCCATATCCGAAAAAATTTTATCATACAATCGTGCTAATAG
AGCAGTA
GCAATACTTTGTAACCATCAACGTGCTGTACCTAAAGGCCATCAGAAATCTATGGAAAAACTCAAAGAAAAAA
TTGGAGC
TAAAAGGGAAACTATCAAAGATGCTGAGAGGCAAGTTAAAGATGCACAAAGAGATGCTAAACA---
TGGAAGTGTTAAGG
AGAAGCAGATCTATGATAAAAAGAAGAAAATGTTGGAAAGACTACGTGAGCAATTGGCCAAGTTGGAAATTCA
AGAAACC
GACCGTGATGAAAATAAAACTATTGCACTTGGTACGTCCAAGCTGAACTATTTGGATCCTAGAATCTCGGTCG
CTTGGTG
TAAGAAGTTTGGTGTGCCCATTGAAAAAATTTATAACAAAACTCAATGGATGCGTTTGCCACCCTTCAGGGTA
ATTGGTG
ATCATTTAAAAGATCGCTTCGATGGTGCTTCTAGAGTAATGTTGAGCAATTCAGCTAGTTCAAGAGG------
------G
AACGC---TAATCGTC---
CAAAACAAGATAAACTTTCGAATAGTATAGCATCGAATAGTATACATAGTAAAAGAGAAAA
CAAGCCGCGCAAGTATAAATATGGTTTTCAACTGAAACCGTACAATCCGGACCATAAACCTCCAAGTCCTAAA
GATTTAG
TGTACCTGGAACCTTCCCCTGGTTTTTGCGAGAAAAACCCAAAGCTCGGCATACAAGGTACTCATGGTAGATT
G-TGCAA
CGATACTTCTATCGGCGTGGATGGATGCGATTTGATGTGTTGCGGTAG-
AGGATACAGAACCCAGGAAGTCATCGTTGTT
GAAAGGTGCAACTGTACGTTCCA--------
>Notobium
GGGAGAAGCCCAGCACTGAATCCCGTGGCCGAATCGGGAAATGTAGTGTTTGGGAGGATCCGTCATCCACCGT
ACGACGC
GTCCAAGTCCTTCTTGAACGGGGCCACATACCCACAGAGGGTGCCAGGCCCGGTAGCCGGAGGATCTCTCCTC
AGAGTCG
GGTTGCTTGAGAGTGCAGCCCTAAGTGGGTGGTAAACTCCATCTAAGGCTAAATATGACCACGAGACCGATAG
CGAACAA
GTACCGTGAGGGAAAGTTGAAAAGAACTTTGAAGAGAGAGTTCAATAGTACGTGAAACCGTTCAGGGGTAAAC
CTGAGAA
GCCCGAAAGGTCGAATGGGGAGATTCAGCGTGTCACGTTTCGGGTCGAGTGACGGTGGTGCGTGCACTGGGCT
GCGCCGT
CCGGATCCGAAACCTGCGGCGAACTCGTGCACTTCTCCCCTAGTAGGACGTCGCGACCCGTTGGGTGCCGGTC
TAAGGCC
AGCGGTGGAGCCTCAAAGTCCCGGCCGGCCCGCTCGACGGTAAGACAGAGACGTGGGGTCGCGATGTTCGCGT
CCGGCCC
GTCACAAGCATGCGCGACTCGGACGCCGGACCTGTGTGCCGACCCCGAGCTCGCCGGCTGTTGGTGGCGGTGT
CCTCGGA
CAGACTACACGTCGGTCGGCGACGCTTTAGCTTTGGGTTTTCAGGACCCGTCTTGAAACACGGACCAAGGAGT
CTAGCAT
GTGCGCGAGTCATTGGGACCGCATCTAAACCTAAAGGCGAAATGAAAGTGAAGGCGCGCCGAGGGAGGATGGG
TCGGGGG
GCGTCTCGTTCTCATCGC------------------------------
CTTACACCGTATTCGCTGATTTGTTCGA-CCC
CATTATTGAGGATTACCATGGTGGTTTCAAGAAGACCGACAGCCATCCCCCAAAGAACTGGGGCGATGTCAAC
ACTTTCG
CCAACCTCGACCCTGCGGGTGAGTACGTTGTGTCAACCCGCGTTCGCTGCGGCCGCTCCATGGAGGGTTACCC
GTTCAAC

CCCTGCTTAACCGAAGATCAATACAAGGAGATGGAACAGAAAGTTTCGTCCACTTTGTCCGGGCTCGAGGGTG
AACTCAA
GGGTACCTTCTACCCGTTGACTGGAATGGACAAGGATACTCAGCAGAAACTCATCGATGATCACTTCTTGTTC
AAGGAGG
GCGATCGTTTCCTCCAGGCTGCTAACGCTTGCCGTTTCTGGCCGTCCGGACGTGGCATCTACCACAACGACAA
CAAGACG
TTCTTGGTCTGGTGCAACGAAGAGGATCACCTCCGTCTTATTTCCATGCAAATGGGTGGGGATCTTGGAGAGG
TTTACCG
CCGTCTCGTTACCGCAGTCAACGACATTGAGAAACGCGTTCCTTTCTCGCATAACGACAGATTAGGTTTCCTC
ACTTTCT
GCCCGACCAATTTGGGTACAACTGTACGTGCCTCGGTACACATTAAAGTACCAAAGCTCGCTGCCAACAAGGC
CAAACTT

GTTCCAAAAAAAGTTTTAATAATTGGTTCAGG
TGGTTTATCAATTGGGCAAGCAGGAGAATTCGATTATTCCGGTTCCCAGGCAATTAAAGCACTACAAGAAGAA
AATATAC
AAACTGTTTTAATCAATCCTAACATTGCAACAGTACAAACCTCTAAAGGTTTAGCTGATAAAGTATATTTCTT
ACCTTTG
GTCCCAGAATACGTAGAAAAAGTAATTAGAGTAGAAAGACCTGGAGGCGTCTTGTTAACGTTTGGAGGTCAGA
CTGGATT
AAATTGTGGTGTAGAACTTCAAAAGGCTGGAGTTTTTGAAAAATATGGTGTTAAAATATTAGGTACCCCTATA
CAAGCGA
TAATAGATACTGAAGATAGAAAAATTTTCAGTGATAGAATTTCATTAATTGGAGAAAAGGTAGCTCCAAGTAT
GGCTGCG
TATTCTGTACAAGAAGCTTTGGAAGCAGCAGAGTTGTTGGGGTATCCTGTTATGGCGAGGGCTGCGTTCTCAT
TAGGGGG
ACTAGGATCAGGATTCGCTAACACAACTGATGAACTTAAGTCACTTGCACAACAAGCACTAGCACATTCCAAT
CAATTAA
TTATTGATAAATCGTTGAGAGGATGGAAGGAAGTTGAGTATGAGGTTGTAAGAGACGCATATCCATATCTCAA
ACAAGTA
AATGATGAGGAATTAAAAGAACCTACAGACAAACGGATGTTTGTTGTCGCTGCTGCATTGAGAAGTGGTTACA
GTGTAGA
CAAATTATATGATTT-
AACAAAAAATTGATCGTTGGTTCTTACAAAAAATGAAAAATATAATTGATTTTAATACCATATTG
GAATCCATTCAGCAAAATAAATTA---------------
ACTGCAAAAGTTTTATTGAAAGCAAAACAAATAGGATTTAG
TGACAAACAAGTCGCTGCAGCGGTTAAAAGTACAGAACTTGCAATACGAAAGCAACGCCAAGATTTTAATCTT
ACTCCTT
ATGTCAAACAAATTGATACTGTGGCTGCTGAGTGGCCTGCCACTACCAATTACCTATATTTAACATATAACGC
AGAAAGT
CATGATTTGAATTTTGCTGAAGAACATATAATGGTTATTGGATCTGGAGTGTACAGAATAGGAAGTTCAGTTG
AATTTGA
TTGGTGTGCTGTAGGATGTTTGAGAGAGCTAAGAAATTTAAATAAAAAAACAATTATGGTAAATTATAATCCA
GAAACTG
TTAGCACGGATTATGATATGTCGGATAGATTGTATTTTGAAGAAATTTCATTTGAAGTAGTTATGGACGAAGT
CTATATC
TTATTTTTACCTGGATTTGGTATAATTTCTCATATTATTAGACAAAGTAGAGGTAAAAAAGAAACATTTGGTT
CTCTTGG
AATAATTTATGCAATAATAGCTATCGGATTATTAGGATTTGTAGTATGAGCTCATCATATATTTACAGTAGGC
ATAGATG
TTGATACTCGAGCTTATTTTACTTCAGCAACAATAATTATTGCTGTTCCTACAGGAATTAAAATTTTTAGATG
ATTAGCT
ACTCTACATGGAACTCAAATTAATTTTAATCCTCCTATTTTATGAGCTTTAGGTTTTGTATTTTTATTTACTA
TTGGGGG
ATTAACTGGTGTAATTTTAGCTAATTCTTCTATTGATATTGTATTACATGATACTTATTATGTAGTTGCTCAT
TTTCATT

ATGTTCTTTCTATAGGAGCTGTATTTGCAATTATAGCAGGTTTAGTTCAATGATTCCCTCTATTCACAGGATT
AACTATA
AATAAAAAATATTTAAAAATTCAATTTTTAGTAATATTTATTGGAGTAAATTTAACTTTTTTCCCTCAACATT
TTCTTGG
TCTAGCAGGAATACCACGACGTTATTCTGACTACCCAGATACTTTTATGCCTTGAAATATAATCTCATCCATT
GGTTCAT
TAATCTCTTTAATTAGAATTTTATATTTTTTATTTATTATTTGAGAAAGATTAGCCTCTATACGACAAATTTT
ATCCTCT
AAAAATTATTCTACTTCAATTGAATGATTCCAACTATTTCCCCCTGCCGAGCATAGTTATTCAGAGCTACCAG
CTCTATC
CAAGGATTACGTTGTAGTGTTTGATTTCCTCGGT-
AAAGATTCCATTAGATATTATAATGAGGTACCTGTAGAAAAACGT
GTCTTCAAAAATCTCCAGTTATTTATGGAAAGTAAGGCGCCTGCTGATGATTTATTCGACCGATTGAACACTA
CTGTGAT
GAATAAACATTTAAATGAATTAATGGAGGGTTTAACCGCCAAGGTATTTCGTACTTACAATGCTTCATTTACG
TTACAAC
AACAGCTCGACAAACTGACCAATGCCGACGATTCCATATCCGAGAAAATCTTATCGTATAACCGTGCCAATCG
AGCTGTG
GCCATCTTGTGTAACCATCAACGTGCTGTGCCGAAGGGCCATCAGAAATCGATGGAGAAATTGAAGGAGAAAA
TCGATGC
CAAGAGAGAAGCTATAAAAGATGGCGAACGGCAAGTTAAGGACGCGCAGAACGACGCGAAACG---
CGGCAGCGTGAGGG
AAAAGCAGATCTATGATAAGAAGAAGAAAATGTTGGAGAGGCTTAGAGACCAGCTTAACAAGTTGGAAATTCA
GGAGACT
GATCGCGATGAAAACAAGACAATCGCGCTTGGCACATCCAAACTAAATTATTTGGATCCGAGGATTTCCGTCG
CCTGGTG
TAAGAAATACGATGTGCCCATTGAAAAAATCTATAATAAAACTCAA------
CGGCTGCCACCGTTCAGGGTCATCGGGG
ACCACCTCAAGGACCGCTTCGACGGCGCCTCCAGGGTGATGCTGAGCAACTCGGCGAGCTCGCG---------
------G
AACGC---GAACCGCC---
CGAAACAGGACAAGCTGTCGAACAGCATAGCGTCGAACAGCATACACAGCAAGCGTGAGAA
CAGGCCGCGCAAGTACAAGTACGGGTTCCAGCTGAAGCCGTACAACCCGGACCACAAGCCGCCGAGCCCGAAG
GACCTGG
TGTACCTGGAGCCATCGCCGGGGTTCTGCGAGAAGAACCCGAAGCTGGGGATACAGGGCACGCACGGCAGGCA
G-TGCAA
CGACACGTCGATTGGTGTGGACGGCTGCGATCTGATGTGCTGCGGGAG-
GGGCTACAGGACCCAGGAGGTCATCGTCGTG
GAGAGGTGCAACTGCACG-------------
>Ochthephilum
GGGAAGAGCCCAGCACCGAATCCCGCGGCCGAGCCGGGAAATGTGGTGTTAGGGAGGGTCCGCTATCCGTCGC
GCGGCGC
GTCCAAGTCCTTCTTGAACGGGGCCACTTACCCAAAGAGGGTGCCAGGCCCGATCGCGGGAGGATCTCTCCTC
AGAGTCG
GGTTGCTTGAGAGTGCAGCCCTAAGTGGGTGGTAAACTCCATCTAAGGCTAAATATAACCACGAGACCGATAG
CGAACAA
GTACCGTGAGGGAAAGTTGAAAAGAACTTTGAAGAGAGAGTTCAATAGTACGTGAAACCGTTCAGGGGTAAAC
CTGAGAA
ATCCGAAAGATCGAATGGGGACATTCAGCGCGTCTCGCCTCAGGACGCGAGACGATGGCGTTCGCGCTCTGTC
GCTCCTT
CCGAAGCCGCGACCGCTGGCGAACGCGTGCACTTCTCCCCTAGTAGGACGTCGCGACCCGTTGGGTGTCGGTC
TAAGGAC
CGCGGTGGAGCCCGCCGGTCCCGACCCGCTCACTCGACGGTAAAACGGTGGCGAAGGGTCGCGAGGTTCGCGC
CCGGCCC
GTCGCAAGCGTTACCGTCCCGGATTTCGGACCTGCGCGCCGATACCGGGCACGGTGGCTGCTGGCGGCGGTGT
CCTCGGG

CCGGCCACACGCCCGTCGGCGACGCTTTAGCTTTGGATTTTCAGGACCCGTCTTGAAACACGGACCAAGGAGT
CTAGCAT
GTGCGCGAGTCATTGGGACTC-
TACTAAACCTAAAGGCGCAATGAAAGTGAAGGCGTGCCGAGGGAGGACCGGTCGGGGG
GCGTCTCATGCTCATCGCGAGCTGAGGCGCACCCAGAGCGTACACGC-
CTTACACCGTATTCGCTGACTTGTTCGA-CCC
CATCATCGAAGACTACCATGGTGGCTTCAAGAAGACCGACAAGCACCCACCGAAGAACTGGGGTGACGGCAGC
GTCTTCT
CCAATTTGGACCCTGCTGGCGAATATGTCGTCTCAACCCGTGTCCGTTGCGGCCGCTCCATGGAGGGATACCC
CTTCAAC
CCATGCTTAACCGAGGAACAGTACAAAGAGATGGAACAGAAGGTTTCCTCCACTTTGTCAAGCCTCGAAGGCG
AACTCAA
GGGTACCTTCTACCCATTGACCGGAATGGACAAGGACACCCAACAGAAGTTGATCGACGATCACTTCTTGTTC
AAGGAGG
GTGACCGTTTCCTCCAGACCGCCAACGCCTGCCGCTTCTGGCCATCCGGACGTGGTATCTACCACAACGACAA
CAAAACA
TTCTTGGTATGGTGCAACGAAGAGGATCACCTCCGCATCATTTCCATGCAGATGGGTGGCGATCTTGGTGAAG
TCTACCG
TCGCCTGGTGACCGCTGTCAACGAAATCGAGAAGCGCGTCCCATTCTCCCACAATGACAGATTAGGTTTCTTG
ACCTTCT
GCCCAACCAACTTGGGCACAACTGTACGTGCCTCTGTACACATCAAAGTACCTAAGCTCGCCGCCAACAAGGC
CAAATTG

GTCCCTAAAAAAGTGCTCATAATTGGTTCTGG
TGGCCTCTCAATTGGACAAGCAGGAGAATTCGATTATTCCGGTTCACAAGCTATTAAAGCACTTCAAGAAGAA
AATATTC
AAACAGTTCTAATAAATCCAAATATTGCAACCATACAAACATCTAAAGGTTTAGCCGATAAAATTTATTTTCT
GCCTTTA
GTTCCCGAATATGTAGAACAAGTAATTAGGGCAGAAAGACCTGGTGGGGTTTTGCTAACCTTTGGTGGGCAAA
CTGGTTT
GAATTGTGGTGTAGAACTTCAAAAAGCGGGAGTATTTAAAAAATATGGAGTTAAAATTTTGGGCACTCCCATA
CAAGCAA
TTATAGATACTGAAGATAGAAAGGTGTTCAGTGAAAAAATTGCAGCAATAGGGGAAAAAGTGGCACCGAGTAT
GGCAGCC
TATTCAGTACAAGAGGCTCTTGATGCAGCCGAACAATTAGGATATCCTGTAATGGCAAGAGCTGCGTTCTCAT
TAGGTGG
TCTTGGTTCTGGTTTTGCAAATACTGCTGATGAATTAAAATCTTTAGCAATGCAAGCCTTGGCACATTCAAAT
CAGTTGA
TTATAGATAAATCATTAAAAGGCTGGAAAGAAGTTGAATATGAAGTAGTAAGAGATGCGTATCCGTACATTAA
AGAAGTC
AATGATGAAGATTTGAAAGAACCTACTGATAAAAGAATGTTTGTTGTTGCTGCAGCATTAAGAGCTGGATACA
GTGTAGA
GAAATTATATAATTT-
AACCAAAATCGATTCTTGGTTCCTACAAAAAATGAAGAATATTATTGATTTCAATACTTTTTTG
GAATCGATGGATCAACATAAATTA---------------
TCATCAGATCTTTTACTTAAAGCAAAACGAATTGGTTTCAG
TGATAAACAAATTGCTGTTGCAGTTAAAAGTACAGAATTAGCAATTAGAAAACAAAGGCTTGATTATGGTATT
ACTCCAT
TTGTAAAACAAATAGATACAGTTGCTGCTGAATGGCCAGCAACYACTAATTATCTTTACCTTACTTACAACGC
TATAGAA
CATGACCTTGATTTCAACCAAGAACATACTATGGTTATAGGTTCTGGAGTTTATCGAATTGGTAGTTCAGTTG
AGTTTGA
TTGGTGTGCTGTAGGCTGCTTAAGAGAACTTAGAAAATTAAATAAGAAAACTATTATGGTAAATTATAATCCT
GAAACTG
TTAGCACTGACTATGATATGTCAGATAGATTGTATTTTGAAGAAATATCGTTTGAAGTAGTTATGGATGAAGT
ATACATT

TTAATTCTCCCTGGCTTTGGAATTATTTCTCATATTATTTGCTACAGAAGAGGAAAATCAGAAACTTTTGGGG
CTTTAGG
AATAATTTATGCAATATTAGCAATTGGGTTGCTAGGATTTATTGTTTGAGCTCATCATATATTTACTGTAGGA
ATAGATG
TTGATACTCGAGCTTATTTTACATCAGCAACAATAGTTATCGCTGTTCCAACAGGAATTAAGGTATTTAGATG
AATAGCA
ACTATTTATGGGGGAAATTTAAACTTTAATCCCCCAATACTATGATCCTTAGGTTTTGTTTTTTTATTTACGG
TCGGTGG
ATTAACAGGAGTAATTTTAGCAAATTCATCAATTGATATTATTTTACATGATACTTATTATGTAGTAGCTCAT
TTTCATT
ACGTTTTATCGATAGGGGCAGTTTTTGCTATTATAGCAGGATTAGTACAATGATTCCCATTATTTATAGGATT
AACCTTA
AATGAAAAATGGCTGAAAATTCAATTCTTAATGATATTTATCGGGGTAAACTTAACATTTTTCCCTCAGCACT
TTTTAGG
ATTAGCAGGTATACCCCGCCGATATTCTGACTACCCTGATGCCTATACAACTTGGAACGTAATTTCTTCAATT
GGGTCTA
TAATTTCTTTTATCGGAATTATATTTTTTTTATGAATTATTTGAGAAAGAATAATTTCTTTACGAAGACCTTT
AGGATCA
ATTACCCCCCCAACAGCTATTGAATGAATACATAAATATCCCCCAACAGAGCATACTTATTCGGAACTTCCAT
TTGTAAC
TAAAGAGTATGTAGTAGTATTTGATTTCCTTGGT-
AAGGATTCCATTAGATATTATAACGAAGTACCTGTCGAAAAGAGA
GTCTTTAAAAATCTGCAATTATTCATGGAGAACAAGGCRCCCGGYGATGACTTGTTCGACAGACTGAACACAT
CTGTGAT
GAATAAACATTTAAACGAGCTAATGGAAGGGCTCACCGCGAAGGTATTTCGTACTTACAACGCCTCATTTACA
YTGCAAC
AACAATTAGACAAACTGACCAACGAGGACGATTCATTATCCGAAAAGATTCTCTCATACAACCGCGCCAACCG
TGCTGTC
GCCATCCTGTGTAACCATCAACGTGCTGTCCCGAAAGGTCACCAGAAATCCATGGAGAAGTTGAAGGAGAAGA
TCCAAGC
CAAAAGAGACTCKATCAAGGAYGGGGAGCGGCAAGTCAAAGATGCCCARAARGACGCCAAGCA---
CGGCAGTGTAAAAG
AAAAACAAATCTACGATAAGAAAAAGAAAATGTTGGAGAGRCTAAAGGAGCARCTGGCGAAATTAGAAATCCA
GGAGACR
GACAGAGAYGAGAACAAAACGATCGCCCTCGGCACTTCGAAGCTGAACTACTTGGATCCCCGAATATCCGTGG
CCTGGTG
TAAAAAGTACAACGTGCCCATTGAAAAGATCTACAACAAAACCCAA------
CGACTTCCACCTTTTAGAGTAATCGGCG
AYAACTTAAAAGACCGCTTTGATGGCGCATCCCGAGTTATGCTAAGTAACTCAGCTAGTAATTC---------
------A
AGAAA---TAACCGGC---
CYAAGCAAGATAAATTAAGCAATAGCATATCCTCAAATAGCATCCATAGTAAAAGAGAAAA
TCGCCCTAGAAAATATAAATATGGTTTCCAACTCAAACCGTATGATCCTGACCATAAACCACCAAGCCCTAAA
GATTTAG
TGTATTTAGAACCATCGCCAGGATTTTGCGAGAAGAACCCTAAATTGGGTATCCAAGGCACTCACGGTAGGCA
A-TGCAA
CGATACTTCAATCGGAGTGGACGGTTGCGATCTTATGTGTTGTGGTAG-
AGGCTACCGCACCCAAGAAGTCATAGTCGTA
GAAAGATGTAACTGCACA-------------
>Oedichirus
GGGATAAGCCCAGCACTGAATCCCGCGGCCGAGCCGGGAAATGTAGTGTTAGGGAGGGTCCGCTATCCATCGT
GCGGCGA
GTTCAAGTCCTCCTTGAACGGGGCCACTCACCCACAGAGGGTGCCAGGCCCGATAGCGGGAGGATCTCTCCTC
AGAGTCG
GGTTGCTTGAGAGTGCAGCCCTAAGTGGGTGGTAAACTCCATCTAAGGCTAAATATAACCACGAGACCGATAG
CGAACAA

GTACCGTGAGGGAAAGTTGAAAAGAACTTTGAAGAGAGAGTTCAATAGTACGTGAAACCGTTCAGGGGTAAAC
CTGAGAA
GCCCGAAAGTTCGAACGGGGAGATTCAGCGCGTCTCGGTTTTGGTCGGGTGACGGTGGCGTTCGCGTCGGGCC
GCACCGA
TCGAAGCCGTTGCCGGCGACGAAGCGTGCCACTTCTACCCTTGTAGGACGTCGCGACCCGTTGGGTGTCGGTC
TAGGTTN
CGCGGTGGAGCCCGTCGGTCCTGGCCGACTCGCTCGACGGTAAAACGATGGCGGTGGGTCGCAAAGTTTGCGT
CCGGCCC
GTTGTCGGCGCGGTTGCCCCGGCTGTCGGACTGTTGTGCCGACACCGGGAGCCGCCGTTGCCGTCAACGGTGT
CCTCGGA
CGGGCCACACGCTTGTCTGCGACGCTTTAGGTTTGGGCTTTCAGGACCCGTCTTGAAACACGGACCAAGGAGT
CTAGCAT
GTGCGCGAGTCATTGGGATTTATTCTAAACCTAAAGGCGAAATGAAAGTGAGGATAATCCTAGGGAGGATGGC
CGTCGGG
GCGTCTCGTTCTCATTACGAGATGAGGCGCACCCAGAGCGTACACGC-
CCTACACCGTCTTCGCCGACTTGTTCGA-TCC
CATCATCGACGACTACCATCAGGGTTTCAAAAAGACCGACAAGCATCCACCAAAGAACTGGGGCGATGTCAGC
ACTTTTG
GCAATCTTGACCCTACCGGCGATTACATCGTGTCGACCCGTGTACGTTGCGGCCGTTCCATGGAAGGCTACCC
CTTCAAC
CCTTGCTTAACCGAGGATCAATACAAAGAGATGGAACAGAAAGTGTCAACTACTCTTTCTGCTCTCGAAGCCG
AGCTTAA
GGGTACCTTCTACCCGTTGACTGGCATGAGCAAGGAGACACAGCAGCAGCTCATCGACGATCATTTCTTGTTC
AAGGAAG
GAGATCGTTTCTTGCAGGCCGCCAACGCTTGCCGTTACTGGCCATCTGGACGTGGTATCTACCACAACGACAA
CAAGACC
TTCTTGGTATGGTGCAACGAAGAGGATCACCTCCGTCTCATTTCTATGCAAATGGGTGGCGATCTTGGCGAGG
TCTACCG
CCGTCTCGTAACAGCTGTCAACGACATCGAAAAACGCATTCCTTTCTCTCACAATGACAGATTAGGTTTCCTC
ACTTTCT
GCCCATCCAACTTGGGCACAACTGTACGTTCCTCTGTACACATCAAAGTACCCAAACTTGCCGCCAACAAGGC
CAAGCTT

ATTCCTCAAAAGGTTCTCATTATAGGTTCCGG
TGGATTATCCATAGGTCAAGCTGGAGAGTTTGATTATTCTGGTTCGCAAGCCATCAAAGCGCTTCAGGAAGAG
AATATCC
AGACGGTTTTGATAAATCCTAACATAGCAACAGTGCAGACTTCAAAAGGTCTAGCGGATAAGGTGTATTTCCT
ACCTTTG
GTGCCGGAATATGTGGAGCAGGTGATTCAGGCTGAAAGACCAGGAGGTGTTTTGTTGACTTTTGGTGGTCAAA
CTGGGTT
AAATTGCGGCGTGCAGTTGCAGAAAGCTGGAGTGTTTGAAAAATACGGTGTAAAAATTTTGGGTACGCCCATT
GAAGCCA
TTATAGATACTGAAGATAGGAAAATCTTCAGCGAAAGGATTGCTTTGATCGGTGAAAAGGTTGCACCTAGTAT
GGCCGCT
TATTCGGTACAGGAAGCGCTTGATGCAGCTGAGCAATTGGGTTATCCTGTGATGGCTAGAGCCGCTTTTTCGT
TGGGTGG
TTTGGGATCTGGTTTTGCAAATAGTTGTGAAGAGCTCAAGTCTTTGGCGCAACAAGCTTTGGCTCATTCCAAT
CAGTTGA
TTATAGATAAGAGTTTGAAGGGCTGGAAGGAAGTGGAATATGAGGTTGTTAGAGATGCGTATCCGTACCTACA
GGAAGTC
AACGATGAGGAGTTGAAGGAACCAACGGATAAACGGATGTTTGTGGTGGCTGCTGCGTTACGTTCTGGATATT
CTGTTGA
CAGGCTTTATGAGCT-
TACAAAAATAGATCGCTGGTTTTTGCAGAAGATGAAGAACATTATAGATTTTAACACGATTTTG
GAATCGATTCATCAACACAAGATG---------------
ACGGCTAAAATTTTGCTCAAAGCCAAACAGATTGGTTTTAG

TGATAAGCAAATTGCTGCTGCTGTGAAGAGTACGGAGTTGGCTATTAGGAAGCAACGTCAGGACTTTAAGATA
ACGCCGT
TTGTGAAGCAGATAGATACAGTGGCTGCTGAATGGCCTGCAACCACAAATTACCTGTATTTGACTTACAACGC
CATCAAC
CACGATTTGGCCTCTTCCGAAGAACACACCATGGTGATTGGTTCTGGTGTGTACCGAATTGGTTCTTCCGTTG
AGTTTGA
TTGGTGCGCTGTTGGTTGCTTGAGGGAGTTGCGTAAACTAAACAAAAAAACCATTATGGTAAACTACAATCCG
GAAACTG
TCAGTACWGACTATGACATGTCGGATCGTTTGTATTTCGAAGAAATATCTTTTGAAGTAGTCATGGATGAAGT
TTATATT
TTAATTTTACCTGGATTTGGAATAATTTCTCATATTATTAGTCAAAGTAGAATAAAAAAAGAAACTTTTGGAA
CGTTAGG
AATAATTTATGCAATAATAGCTATTGGGTTACTAGGATTTGTAGTCTGAGCCCATCATATATTCACAATTGGA
ATAGATG
TAGATACTCGTGCATATTTTACTTCTGCTACAATAATTATTGCTGTTCCAACAGGAATTAAAATTTTTAGGTG
ACTTGCT
ACTCTTCATGGAACTCAAATAAATTTTAACCCTCCTTTATTATGGGCTTTGGGATTTGTTTTTCTTTTCACAA
TTGGTGG
ACTAACAGGTGTAATTTTAGCAAACTCTTCTATCGATATTATTCTTCATGATACTTATTATGTTGTTGCTCAT
TTTCATT
ATGTTTTATCTATAGGAGCAGTTTTTGCTATTATAGCAGGTCTTGTTCAATGATTCCCATTATTTACTGGATT
ATCATTA
AATAATTTTTACTTAAAAATTCAATTTTTTACTATATTTGTAGGAGTTAATTTAACTTTTTTCCCTCAACATT
TTTTAGG
TTTAGCAGGAATACCTCGACGTTATTCTGATTATCCTGATGCTTACACTCCTTGAAATATTCTATCTTCTATT
GGAAGAT
TAATTTCCTTTATTAGAATTATTTTTCTACTATTTGTAATCTGAGAAAGATTCACTTCACGGCGTTTAATTAT
TTCAACT
AAAAATTTTTCAACATCAATTGAATGATACCAATCATACCCACCAGCAGAGCATAGGTATCTAGAACTTCCTA
TACTTTC
TAGGGACTATGTGGTTGTGTTTGATTTCCTTGGT-
AAAGATTCCATTAGATACTACAATGAAGTACCTGTTGAAAAACGT
GTATTTAAGAACTTACAACTATTTATGGAAAATAAAGCACCTGGTGATGATTTATTTGATCGTTTAAATACAG
CTGTGCT
AAACAAACATTTAAATGAACTTATGGAAGGCCTTACAGCCAAGGTGTTTCGTACTTACAATGCATCATTTACT
TTACAGC
AGCAGTTAGATAAATTGACCAATGCTGATGATTCCATATCTGAAAAAATCTTATCATATAATCGAGCTAACCG
AGCTGTA
GCTATTCTGTGTAACCATCAACGTGCAGTGCCTAAAGGCCATCAAAAATCTATGGAAAAATTGAAAGAGAAGA
TTGAAGC
TAAACGAGAAACTATAAAAGATGGGGAGAGGCAAGTAAAGGATGCACAGAAAGATGCCAGACA---
TGGTAGTGTGAAAG
AAAAACAAATATACGATAAGAAGAAAAAGATGTTGGAACGCCTTAAGGAGCAGTTAGCAAAACTCGAAATTCA
GGAGACT
GACCGAGACGAAAACAAAACGATTGCACTTGGTACGTCAAAACTGAATTATTTGGATCCTAGAATTTCCGTCG
CTTGGTG
TAAAAAGTACGGTGTGCCCATTGAAAAAATTTATAACAAAACTCAA------
CGTTTGCCACCGTTCCGTGTCATCGGCG
ACAACCTGAAGGACCGCTTCGATGGCGCCTCTCGCGTTATGCTGAGCAACTCCGCCAACTCCAGGAA------
------C
AGTAA---
AAGTAGACAAAATAAGCAGGACAAACTTTCAAATAACATCGCCTCCAACAGCATCCACAGTAAACGGGAGAA
CAGACCGCGTAAATATAAATACGGCTTTCAACTCAAACCTTACAATCCCGACCACAAACCACCTAGTCCCAAA
GATTTGG
TGTATCTGGAGACATCTCCGGGATTCTGTGAGAAAAACCCGAAACTTGGTATACAAGGTACACACGGAAGGCA
A-TGTAA

CGACACATCGATCGGCGTGGATGGGTGCGATCTTATGTGTTGCGGTCG-
GGGATACCGTACCCAAGAGGTCGTTGTTGTG
GAGAGATGTAATTGCACT-------------
>Orus

CTGGACTGCGCCTT
CTGAATCCGATGCCGGCGGCGAACGCGTGCACTTCTCCCCTAGTAGGACGTCGCGACCCGTTGGGCGCCGGTC
TACGGCC
GACGGTGGAGCCTTGGGGTCCCGGCCGGCCCGCTCGACGGTAAGACAAAGACGTGGGGTCGCGATGTTCGCGT
CCGGCCC
GTCACAAGTGCGAGCGACTCGGACGTCGGACCTGTGTGCCGACCTCGAGCTCGCCGGCTGTTGGTGACGGTGT
CCTCGGA
CAGACTACACGTCGGTCGGCGACGCTTTAGCTTTGGGTTTTCAGGACCCGTCTTGAAACACGGACCAAGGAGT
CTAGCAT
GTGCGCGAGTCGTTGGGACCGCATCTAAACCTAAAGGCGAAATGAAAGTGAAGGCGTGCCGAGGGAGGATGGG
TCGGGGG
GCGTCTCGTTCTCATCGCGAGATAAGGCGCACCCAGAGCGTACACGC-----------------------
----

GATATTAATTTACATGATACATATTATGTNGTAGCTCATTTTCATT
ATGTATTATCAATAGGAGCTGTATTNGCTATTATAGCGGGATTAGTTCAATGATTTCCTCTATTTACAGGACT
TTCAATA
AATGAATATTTATTAAAAATTCAATTTTTATCAATATTTATTGGAGTAAATTTANCCTTTTTTCCTCAACATT
TCTTNGG
ATTAGCTGGAATACCCCGCCGATATTCAGATTACCCTGATATTTATACTCCATGAAATGTAATTTCCTCAATT
GGCTCTC
TTATTTCAATAATAAGAATTTTTTTATTTTTATTTATTTTATGAGAAAGATTTATTTCAATACGAATAATTAT
TTCAGCC
AAAAATTTTAGTTCTTCAATTGAATGATATCAACTATACCCCCCAGCTGAGCA--------------------
----

GATTTATTCGACCGTTTAAACACCGGCGTCAT
GAACAAACACTTAAACGAACTGATGGAAGGATTAACCGCAAAGGTGTTCCGTACTTACAACGCTTCGTGGACC
TTACAGC
AACAATTAGAGAAATTAACCAATCCGGATGATTCCATATCCGAAAAAATACTCTCCTATAACCGTGCGAACCG
CGCCGTC
GCCATTCTCTGTAACCATCAGCGTGCCGTACCGAAAGGCCACCAGAAATCCATGGAGAAGCTCAAGGAGAAAA
TCGAGGC
TAAAAAGGAGTCGATCCGCGACGGCGAGCGACAAGTGAAGGACGCGCAGAGGGACGCCAAGCA---
CGGCAGCGTCAAGG
AGAAACAGATCTACGACAAGAAACGGAAGCAGCTGGCCAAGCTAAAGGAACAACTGGCCAAACTGGAGATACA
GGAGACG
GACCGCGACGAGAACAAAACGATCGCGCTGGGTACGTCGAAGCTGAACTACCTGGACCCGAGGATCTCGGTCG
CCTGGTG

CAAGAAGTTCGATGTGCCCATCGAAAAGATTTACAATAAAACTCAA------
CGTCTGCCGCCGTTCCGCGTGATCGGCG
ACCACCTGAAGGACCGTTTCGACGGGGCCTCCCGCGTCATGATCAGCAATTCGGCGAGCTCGCG---------
------G
AACGC---GASCCGCC---
CGAAACAGGACAAACTATCAAACAGCATCGCCTCCAACAGCATCCACAGCAAGCGGGAGAA
CAGACCGAGGAAGTACAAGTACGGCTTCCAGCTGAAACCGTACAATCCGGAGCACAAGCCGCCGAGTCCGAAG
GACCTGG
TCTACTTGGAGCCGAGTCCCGGTTTCTGCGAGAAGAACCCGAAATTGGGGATCCAGGGGACGCACGGGAGGCA
G-TGCAA
TGATACTTCGATA--

>Oxyporus
GGGAAGAGCCCAGCACTGAATCCCGCGGCCGGGCCGGGAAATGTAGTGTTTGGGAGGATCCGCTATCCGTCGT
GCGGTGC
GTCCAAGTTCTTCTTGAACGGGGCCATTTACCCATAGAGGGTGCNAGGCCCGGCGACGGGAGGATCTCTCCTC
AGAGTCG
GATTGCTTGAGAGTGCAGTCCTAAGTGGGTGGTAAACTCCATCTAAGGCTAAATATAACCACGAGACCGATAG
CGAACAA
GTACCGTGAGGGAAAGTTGAAAAGAACTTTGAAGAGAGAGTTCAATAGTACGTGAAACCGTTCAGGGGTAAAC
CTGAGAA
ACCCGAAAGTTCGACCGGGGAGATTCAGCGTGTCTCGCCAGTACTCGTGCGACGAGGGCGTACGCGCCGTG-
AGCGTCTT
GTGCTCCGCAGTCCCGCCGCGTACGCGTGCACTTCTCCCCCAGTAGGACGTCGCGACCCGTTGGGCGTCGGTC
TGAGGCC
CGCGGTGGAGCCCGCGCGGCCCGACCGACTCGCTCGACGGTATGACTGTGGCGAGGGGCCGCGACGTTCGCGT
CCGACCC
GTCGCAAGCGCGCGCGGTCGATGTGTCGGACCTGTGTGCCGGCCTCGAGCCCGTCGGCTGCTGGCGGCGGTGT
TCTCGGA
CAGACTACACGCCCGTCTGCGACGCTATTGCTTTGGGTTTTCAGGGCCCGTCTTGAAACACGGACCAAGGAGT
CTAGCAT
GTGCGCGAGTCACTGGGACTT-
GTATAAACCCAAAGGCGAAATGAAAGTGAAGGCCCGCCTAGGGAAGATCGGTCGTGGG
GCGTCTCGCGCTCATCGCGAGCTGAGGCGCACCCAGAGCGTACATGCGCTTACACCGTCTTCGCTGATTTGTT
CGA-CCC
AATCATTGAAGATTACCATACTGGATTCAAGAAGACCGACAGCCATCCACCCAAGAACTGGGGTGATGTAAAC
AGCTTCG
CTAACTTGGACCCCACTGGTGAATTCATTGTATCCACCCGTGTGCGTTGCGGACGTTCTTTGGAAGGTTACCC
ATTCAAC
CCATGCTTAACCGAAGAGCAATACAAGGAGATGGAACAGAAAGTTTCGTCCACCTTGTCTGCTTTGGAAGGTG
AACTCAA
GGGAACTTTCTACCCCTTGACTGGAATGGAAAAGGATGTCCAACAGAAACTCATCGATGACCACTTCTTGTTC
AAGGAGG
GTGACCGTTTCTTGCAAACTGCTAACGCTTGCCGTTTCTGGCCATCTGGACGTGGTATCTACCACAACGACAA
CAAGACC
TTCTTGGTCTGGTGCAACGAAGAAGACCATCTTCGCATCATCTCCATGCAAATGGGTGGTGATCTTGGAGAAG
TTTTCCG
TCGTCTTGTAACTGGTGTGAATGACATCGAGAAGCGTATCCCATTCTCCCACAACGACCGATTGGGTTTCTTG
ACCTTCT
GCCCAACCAACTTGGGAACAACTGTCCGCGCCTCCGTGCACATTAAGGTACCCAAACTCGCTGCCAACAAGGC
TAAGCTC
GATGAGGTTGCTGCCAAATACAACTTGCAAGTTCGTGGAACTCGT---
AATCCCAAAAAGGTTTTGATTATCGGTTCAGG
TGGATTAAGTATTGGACAAGCAGGAGAATTTGATTATTCTGGCTCGCAAGCTATCAAAGCTTTACAAGAAGAA
AATATAC

AAACGGTTCTAATCAATCCCAANATTGCAACTGTACAAACCTCTAAAGGTTTAGCCGATAAAATTTATTTCCT
CCCTTTG
GTACCTGAATATGTTGAGCAAGTAATTCGAGTAGAACGTCCAGATGGAGTTCTTCTAACCTTCGGAGGACAAA
CCGGATT
AAATTGTGGTGTCGCATTGGAAAAAGCTCAAATCTTCAAAAAATATGGAGTTAGGATTTTGGGCACACCAATA
CAAGCGA
TTATCGATACAGAAGATAGGAAAATTTTTTCCGAGCGTATTCGNAGTATTGGAGAAAAAGTTGCTCCAAGCAT
GGCTATA
CATTCAGTGCAGGAAGCTTTGGATGCTGCAGAAACTTTAGGTTTTCCTGTAATGGCTCGAGCTGCGTTTTCTT
TAGGAGG
ATTAGGATCTGGGTTCGCTGATGATCCTGAAGAATTNCGCCCGTTAGCCCAACAAGCCCTAGCACACTCAAAT
CAGTTAA
TAATTGATAAGAGTTTGAAAGGTTGGAAGGAAGTTGANTATGAAGTTGTTAGAGATGCTTATCCGTACCTTAA
AGAAGTT
GATGACGAGGAGCTTAAAGCACCCACCGATAAAAGAGTGTTTGTTGTAGCAGCTGCGCTCAAACAAGGGTACA
CTGTTGA
TAAGCTTTACCAACT-
AACCAAAATCGATCGTTGGTTCCTACAGAAAATGAAGAACATCATCACTTACGTTAATCTACTG
GAATCTTTGGACCAATTCAAACTA---------------
ACTCGTGGGGTTTTATTAGAAGCTAAACAAATCGGTTTTAG
TGACAAACAAATTGCTTCTTCTGTTAAAAGCACTGAGCTTGCAGTTCGTAAACAGCGTCAAGATTTACAAGTT
ACTCCAT
ATGTTAAACAAATCGATACAGTTGCAGCTGAATGGCCTGCAACAACAAATTANCTGTATTTAACATACAACGC
AAACAGT
CATGATATAACTTTCGAAGAAGAGCACACGATGGTGATAGGGTCAGGAGTTTATAGAATTGGAAGCTCTGTAG
AATTTGA
TTGGTGTGCTGTTGGATGTTTGCGTGAGTTACGCAAACTTAACCGAAAAACCATTATGGTCAACTACA-----
----

GAAGTATACATT
TTAATTCTTCCTGGATTTGGTTTAATTTCTCATATTATTAGACAAGAAAGAGGAAAAAAGGAAACTTTTGGAG
CATTAGG
AATAATTTATGCAATATCAGCAATTGGTCTATTAGGATTTATTGTATGAGCTCACCATATATTTACTGTTGGA
ATAGACG
TAGACACACGAGCTTACTTTACTTCAGCTACTATAATTATTGCTATTCCTACTGGAATTAAAATTTTTAGATG
ACTAGCT
ACTCTACACGGAACTCAATTAAACTATTCCCCAGCTATACTTTGGGCATTAGGATTTGTATTCTTATTCACTG
TTGGAGG
ATTAACAGGAGTTGTATTAGCAAACTCTTCAATTGATGTAATTTTACATGATACTTATTATGTAGTAGCTCAT
TTTCATT
ATGTTTTATCTATAGGAGCTGTATTTGCTATTATAGGAAGATTAATTCAATGATGACCATTACTAACAGGAGT
TATCTTA
GATGAAAAATTATTAAAAATTCAATTTTTAATGATATTCATTGGAGTAAATTTAACTTTCTTCCCTCAACATT
TCTTAGG
TTTATCAGGAATACCTCGACGATACTCAGATTACCCTGATGCTTATAGTTTATGAAATATAGTATCTTCTATT
GGTTCAT
TAATTTCTCTAGTTAGAGTATTCGTATTCTTATACTTAATTTGAGAAAGATTTGCATCAAGTCGAAAAAGAAT
AACACCT
CTTAATTTACCAACTTCTATCGAATGACTTCAATCTATACCTCCTGCTGAACATAGTTAT-------------
----
-AAGGAATATGTGGTTGTGTTTGACTTCCTCGGT-
AAAGATTCAATTAGGTATTACAATGAAGTTCCGGTAGAGAAGCGC
GTTTTTAAGAACGTGCAACTTTTCATGGAGAATAAGGCGCCTGGTGATGATTTGTTTGATCGACTCAACACTG
GAGTGAT
GAATAAGCATTTGAACGAGTTGATGGAAGGACTGACGGCGAAGGTGTTCCGTACGTACAACGCTTCTATCACA
CTGCAGC

AACAGTTGGACAAACTTACAAATTCGGACGATTCGGTTTCGGAAAAGATTCTTTCATACAACCGTGCTAATCG
AGCTGTC
GCCATTCTGTGTAACCATCAACGTGCTGTGCCGAAGGGGCATGACAAATCGATGGAGAAGCTAAAAGAGAAGA
TTGAAGC
NAAACGAGATACTATCAAAGACGCGGAGCGTGGTGTGAAAGATGCGCAAAAAGACGCGAAACA---
CGGAAGTGTAAAGG
AAAAACAGATCTACGATAAGAAAAAGAAAGCACTCGAAAGACTCAAGGAACAACTTGCAAAGCTCGAGATTCA
AGAGACG
GATAGGGACGAGAATAAGACGATAGCGCTGGGTACGTCTAAGCTGAATTATTTGGATCCTAGAATTTCGGTGG
CGTGGTG
CAAGAAATATGATGTACCGATTGAGAAGATCTACAATAAGACACAG---------
TTGCCTCCNTTCCGCGCGATCGGCG
ATCACCTGAAGGACCGCTTCGACGGTGCGTCGCGTGTGATGCTGAGCAACGCGAGTAACGGTCG---------
------C
AATGC---GAATCGGC---
CCAAAGAGAACAAGCTTTCAAATAGNATCCAGTCGAATAGCATCCACAGCAAGCGCGAGAA
CCGACCCCGTCGCTACAAATACGGATTCCAGCTNAAACCCTACAACCCAGACCACAAGCCACCGAGCCCAAAG
GATTTAG
TGTACCTCGAACCATCTCCAGGTTTTTGCGAGAAGAATCCAAAACTGGGCATTCAAGGCACGCACGGTCGACA
G-TGTAA
CGATACGTCGATCGGGGTGGACGGTTGCGATCTT---------------------------------------
----

>Paederus_littoralis
GGGAAGAGCCCAGCACCGAATCCCGCGGCCGAGCCGGGAAATGTGGTGTTTGGGAGGATCCGCTAACCGTCGT
GCGGCGC
GTCCAAGTCCTTCTTGAACGGGGCCACTTACCCATAGAGGGTGCCAGGCCCGGCAGCGGGTGGATCTCTCCTC
AGAGTCG
GGTTGCTTGAGAGTGCAGCCCTAAGCGGGTGGTAAACTCCATCTAAGGCTAAATATAACCACGAGACCGATAG
CGAACAA
GTACCGTGAGGGAAAGTTGAAAAGAACTTTGAAGAGAGAGTTCAATAGTACGTGAAACCGTTCAGGGGTAAAC
CTGAGAA
ATCCGAAAGGTCGAATGGGGACATTCAGTGCGTTTTCGTTGTGGTCGTGCGACGGTGACGTTCGCGTCCGGCC
GCGCCTT
CCGCATCCGTGACCGAAGGCGAACGCATGCACTTCTCCCCTAGTAGGACGTCGCGACCCGTTGGGTGTCGGTC
TAAGGTC
CGCGACGGAGCCCTCGGGACCCGGCCGGCCCGCTCGACGGTACGAAGGTGGCGTTGGGTCGCGAAGTTCGCGC
CCGGCCC
GTCGCAAGCGCTTGCGTCCCGTCTCTCGGACCTATTTGCCGAAGCGGGGCGCGCAGGCTGATTGCGACGGTGT
CCTCGGG
CGGACCACACGCCCGTCGGCGACGCTTTAGCTTTGGATTTTCAGGACCCGTCTTGAAACACGGACCAAGGAGT
CTAGCAT
GTGCGCGAGTCATTGGTATTT-
AACTAAAACTAAAGGCGCAATGAAAGTGAAGGCGTGCCTAGGGAGGACCGGGCGGGGG
GCGTCTCGTACTCATCGCGAGTTGAGGCGCACCCAGAGCGTACACGC--------------------------
----

GTTCCCAAGAAGGTACTCATAATTGGTTCTGG
AGGACTTTCTATAGGTCAGGCAGGAGAATTTGATTACTCAGGCTCACAAGCAATTAAAGCTTTACAAGAAGAG
AATATTC
AAACGGTACTTATTAATCCGAACATAGCTACAGTGCAGACATCAAAAGGTATGGCTGATAAAGTGTATTTTCT
ACCATTA
GTACCTGAGTACGTAGAACAAGTAATTCGATCTGAAAGACCAGCAGGTGTTCTCTTAACTTTTGGTGGTCAAA
CTGGTTT
AAATTGCGGTGTCGAATTGGAGAAGGCCGGCGTATTTAAAAAATACGGAGTGAAAATACTGGGCACTCCAATT
CAAGCAA
TCATAGAAACTGAAGATAGAAAAATATTTAGCGAACAGATTGCTATAATTGGTGAGAAAGTTGCGCCTAGTAT
GGCAGCT
GAATCAGTACAAGAAGCTTTGGATGCTGCTGAGCAACTAGGATATCCAGTAATGGCACGTGCCGCATTTTCTC
TTGGCGG
TTTAGGATCAGGTTTTGCTGATTGTAAAGAAGAATTGAAGTGTTTAGCTCAACAAGC----------------
----

----CTGTACGACCT-
GACGAAGATCGACCGCTGGTTCATACAAAAGATGAAGAACATCATCGATTTCACGACGAGGCTG
GAGCGAAC----CGACAAAGAATG---------------
ACCCCCGAAATCCTGCTCAAGGCCAAACAAATCGGTTTCAG
CGACAAACAAATTGCTTCAGCCGTTAAGAGCACCGAGTTGGCGATTCGGAAACAGCGGCAGGATTTCGGACTT
ACCCCGT
TCGTCAAGCAAATCGACACGGTCGCCGCGGAATGGCCGGCAACAACAAACTACCTCTACGTAACCTACAATGC
TTTGGAG
CACGACCTTGAATTCTCCGAGGAACACGTCATGGTCATAGGTTCCGGAGTTTACAGGATTGGAAGCTCCGTCG
AGTTCGA
CTGGTGTGCCGTCGGCTGCCTCCGCGAACTGCGGAAGCTCGGCAGGAAAACCGTAATGGTCAACTACAACCCC
GAGACTG
TCAGTACCGACTACGATATGTCTGACCGACTATACTTCGAAGAAATATCCTTTGAAGTTGTCATGGAC-----
----

ACATATTATTTCTTACAGAAGAGGAAAACAAGAAACATTTGGAGCAATAGG
AATAATTTATGCAATATTAGCAATTGGTTTATTAGGATTCATTGTTTGAGCTCACCATATATTTACTGTAGGA
ATAGATA
TCGATACACGAGCTTATTTCACTTCAGCAACCATAGTAATTGCTGTTCCTACAGGAATTAAAGTATTTAGTTG
AATAGCA
ACTATTTATGGAGGAAATTTAAATTTTAGCCCTCCAATAATTTGAAGATTAGGGTTTGTATTTTTATTTACAG
TGGGGGG
ATTAACAGGAGTAATTTTAGCAAATTCATCAATTGATATTGTTTTACATGACACTTATTATGTAGTAGCACAT
TTCCATT
ATGTTTTATCTATAGGGGCAGTATTTGCAATTATAGCAGGATTAGTACAATGATTCCCTATATTCATTGGCTT
AATATTA
AATGAAAAATACTTAAAAATTCAATTTTTAATTATATTTATTGGAGTAAATTTAACATTTTTCCCTCAACATT
TCTTAGG
ATTATCAGGAATACCTCGTCGATACTCCGATTACCCTGATGCATACACTATATGAAATGTAATTTCATCAATT
GGGTCAA

TAATTTCATTTATTGGAATTATATTTTTTTATGAATTATTTGAGAAAGATTCATTTCTATACGGAAAATTAT
CGGAGCT
CCAATTCCTCCTACGGCATTAGAATGGATACATGCA-------------------------------------
----
-AAAGAATTCGTAGTTGTATTCGACTTCCTCGGT-
AAAGATTCCATTAGATATTATAACGAAGTACCTGTAGAGAAACGA
GTTTACAAAAATCTGCAACTATTTATGGAAAACAAGAAGCCCGGTGATGACCTGTTTGATCGATTGAACACGT
CTGTGAT
GAACAAACATCTAAACGAACTAATGGAAGGTCTCACCGCCAAGGTATTTCGTACTTACAATGCATCTTTTACG
CTGCAAC
AACAACTGGACAAGCTAACAAACCCCGATGACTCTTTATCCGAGAAAATCTTGTCGTACAACCGTGCTAATCG
AGCCGTA
GCTATTCTATGTAACCATCAACGTGCCGTTCCGAAAGGTCATCAAAAATCGATGGAAAAATTAAAAGAAAAAA
TCGACGC
CAAAAGGGAAGCTATTAAGGACGGCGAGAGACAGGTTAAGGACGCTCAAAGAGACGCCAAGCA---
CGGCAGCGTTAAGG
AGAAGCAGATATACGACAAGAAGAAGAAGGCGCTGGAACGGCTTAAGGAGCAACTGGTCAAGTTAGAAATTCA
GGAGACG
GACCGCGACGAAAACAAAACCATCGCGCTCGGCACGT------------------------------------
----

GCCGTTCCGTGTGATCGGTG
ATAACCTGAAGGACCGTTTCGACGGCGCGTCTCGGGTGATGTTGAGCAATTCGGCGAGTTCGAG---------
------G
AACAG---CAACAGGC---
CGAAACAGGACAAACTCAGCAACAACATAGCATCGAACAGCATACACAGCAAGCGCGAGAA
CAGACCGCGTAAATACAAATACGGCTTCCAACTGAGGCCCTACAATCCGGACCACAAGCCACCCAGCCCAAAG
GACCTGG
TCTACTTGGAGCCGTCGCCCGGTTTTTGTGAGAAAAATCCCAAATTGGGCATACAAGGCACGCACGGCAGACA
A-TGTAA
CGACACGTCGATAGGAGTCGACGGCTGCGACCTAATGTGCTGCGGGAG-
GGGCTACAGGACGCAAGAAGTCGTCGTCGTC
GAAAGGTGCAACTGCACGTT-----------
>Pinophilus
GGGAAGAGCCCAGCACTGAATCCCGCGGCCGAGCCGGGAAATGTAGTGTTAGGGAGGGTCCGCTATCCATCGC
GCGGTGC
GTCCAAGTTCTCCTTGAACGGGGCCACTCACCCACAGAGGGTGCCAGGCCCGGTAGCGGGAGGATCTCTCCTC
AGAGTCG
GGTTGCTTGAGAGTGCAGCCCTAAGTGGGTGGTAAACTCCATCTAAGGCTAAATATAACCACGAGACCGATAG
CGAACAA
GTACCGTGAGGGAAAGTTGAAAAGAACTTTGAAGAGAGAGTTCAATAGTACGTGAAACCGTTCAGGGGTAAAC
CTGAGAA
GCCCGAAAGGTCGAACGGGGAGATTCAGCGCGTCTCATCCCGTATCGCACGACGGTGGCGTCCGCGTCGGACC
GCGTCGT
ACGGGGCCGTTGCCGGCGACGAACGCGTGCACTTCTCCCCTTGTAGGACGTCGCGACCCGTTGGGTGCCGGTC
TAAGGGC
CGCGGTGGAGCCCGTCGGTCCTGGCCGGCTCGCTCGACGGTAAACCTTTGGCGTTGGGTCGCGACGTTCGCGT
CCGGCCC
CTCGCGAGCGCGGGTGCCCCGGCCGTCGGACTGTTGTGCCGACACCGGGCGCGCCGTTTGCCCGCGTGGGTGT
CCTCGGA
CAGACCACCCGCCAGTCAGCGACGCTTTAGCTTTGGGCTTTCAGGACCCGTCTTGAAACACGGACCAAGGAGT
CTAGCAT
GTGCGCGAGTCATTGGGACCGCATCTAAACCTAAAGGCGAAATGAAAGTAAAGGCGTGCCCAGGGAGGATGGG
CCTGGGG
GCGTCTCGTTCTCATCGCGAGATGAGGCGCACCCAGAGCGTACACGC-
CTTACACCGTATTCGCCGACTTGTTCGA-CCC

CATTATTGAGGACTACCATACTGGCTTCAAGAAGACCGACAAGCATCCCCCTAAGAACTGGGGTGATGTGAAT
ACCTTCG
CCAACTTGGACGCTGCCGGAGAGTACATCGTTTCCACCCGTGTACGTTGCGGCCGCTCCATGGAAGGATACCC
CTTCAAC
CCTTGCTTGACCGAGGACCAATACAAGGAAATGGAACAGAAGGTGTCCAGCACCTTGTCTGGACTTGAGGGCG
AGCTTAA
GGGTACCTTCTACCCATTGACCGGCATGAGCAAGGAAGTGCAGCAGAAACTCATCGATGACCACTTCTTGTTC
AAGGAGG
GCGATCGCTTCTTGCAATCCGCTAACGCCTGCCGTTTCTGGCCCTCCGGACGTGGTATCTACCACAACGACAA
CAAGACC
TTCTTGGTATGGTGCAACGAAGAAGATCATCTCCGCATCATCTCCATGCAGATGGGCGGTGATCTCGGCGAGG
TCTACCG
TCGTCTCGTCACAGCTGTCAACGACATCGAGAAACGCGTTCCCTTCTCCCATAACGACAGATTAGGTTTCCTC
ACCTTCT
GCCCATCTAACTTGGGCACAACTGTACGTGCCTCTGTACACATCAAAGTACCCAAATTGGCCGCCAACAAGGC
CAAGCTC

ATTCCTAATAAAGTCCTTATAATTGGTTCTGG
CGGATTGTCGATTGGTCAAGCCGGGGAATTTGATTATTCAGGTTCGCAGGCCATCAAAGCACTTCAAGAAGAA
AATATTC
AAACCGTTTTGATTAACCCAAACATAGCAACAGTACAAACATCGAAAGGTTTAGCAGATAAAGTTTATTTCTT
GCCTTTG
GTGCCGGAATATGTTGAACAAGTGATTAGAGCTGAAAGACCAGGCGGGGTTTTATTGACGTTTGGTGGACAAA
CAGGTTT
GAACTGCGGAGTAGAACTCCAGAAAGCTGGTGTATTTGAAAAGTACGGTGTTAAAATTTTGGGTACCCCGATA
AAGGCCA
TAATTGATACAGAAGATAGAAAAATGTTTAGCGATAGAATTGCACTGATCGGGGAAAAAGTTGCGCCTAGCAT
GGCGGCT
TATTCTGTGCAAGAAGCTTTGGATGCTGCTGAACAACTTGGTTATCCTGTGATGGCAAGAGCTGCGTTTTCAC
TGGGAGG
GTTGGGATCAGGTTTTGCAAATACCTCTGAAGAATTGAAGAGTTTAGCACAACAGGCTCTGGCACATTCCAAC
CAATTAA
TTATTGACAAGTCTTTGAAAGGATGGAAGGAAGTGGAGTATGAAGTAGTTCGAGATGCTTATCCATACTTGAA
AGAAGTT
AACGATGAGGAGTTGAAAGAACCAACTGATAAACGAATGTGGGTGGTTGCGGCAGCTTTGAGAAATGGTTATT
CTGTTGA
GAAGTTGTATGATCT-
AACGAAAATTGATAAATGGTTTTTACAAAAAATGAAGAATATTGTCGATTTTAATACTTTGCTA
GAATCAATCGACCAGCACACTTTG---------------
TCAGCTAAAACTTTGTTAAAAGCAAAACAAATTGGGTTTAG
TGACAAGCAAATAGCCGCAGCTGTAAAGAGCACGGAACTTGCAATTCGAAAACAACGTCAAGACTTTAAAATT
ACACCGT
ACATTAAACAAATAGATACAGTTGCTGCTGAATGGCCCGCCACTACTAACTATCTTTATTTAACTTACAATGC
CATTAAT
CACGATTTACAATTTACTGAGGAGCACACTATGGTTATTGGATCAGGTGTGTATCGAATTGGTAGCTCAGTAG
AGTTTGA
TTGGTGTGCAGTTGGTTGCCTAAGAGAATTGAGGAAATTAAATAGGAAAACAATTATGGTGAATTACAATCCC
GAAACTG
TTAGTACTGATTATGATATGTCAGATAGATTGTACTTTGAAGAAATATCATTTGAAGTCGTTATGGAT-----
----

TTTCTCATATTATTAGACAAAACAGAGGAAAAAAGGAAACTTTTGGAGCATTAGG
AATAATTTATGCAATACTAGCAATCGGATTATTAGGATTTGTAGTATGAGCTCATCACATATTTACAGTGGGA
ATAGATG
TTGACACTCGAGCTTATTTCACCTCAGCTACTATGATTATTGCAGTTCCGACAGGAATTAAAATTTTGAGTTG
ATTAGCA

ACTTTACATGGAACCCAAATAGTATTCAACCCATCTTTATTATGAGCATTAGGATTCGTATTCTTATTTACAA
TCGGAGG
ATTAACTGGAGTAATTCTAGCAAATTCCTCAATTGATATTATTCTTCACGACACATATTATGTTGTAGCCCAT
TTCCATT
ATGTATTATCAATAGGCGCTGTATTTGCAATCATAAGCGGATTAGTACAATGATTCCCATTATTTACAGGAAT
AACTTTA
AATAATTATTTATTAAAAATTCAATTTTTTATAATATTTATTGGTGTAAATTTAACCTTTTTCCCTCAACATT
TTCTTGG
ACTAGCTGGAATACCTCGACGATACTCAGATTATCCGGATGCCTATACCCCCTGAAATGTTTTATCCTCAATT
GGATCAC
TAATTTCACTTATTAGAATTATATTTTTAYTATTTATTATTTGAGAAAGRTTTACTTCWATACGATTATCATT
ATTTTCC
AAAAATTTTGGTACTTCTATTGAATGATATCAATTATTTCCTCCWGCTGAACATAGATATTCAGAATTACCAT

-AAGGACTACGTTGTTGTGTTTGATTTTCTTGGA-
AAGGATTCCATTAGATATTACAATGAAGTACCTGTTGAAAAACGT
GTCTTTAAGAATTTGCAATTGTTCATGGAAAATAAGTCACCAGGTGATGACTTATTCGATCGTTTAAATACAG
GTGTATT
AAACAAACATTTGAATGAACTTATGGAAGGCCTGACAGCAAAGGTGTTTCGTACTTACAATGCATCATTTACA
TTACAGC
AACAATTAGATAAATTGACCAATACTGATGATTCCATATCTGAAAAAATTTTATCATACAATCGAGCTAATCG
AGCGGTA
GCTATCTTATGTAACCATCAACGTGCAGTGCCTAAGGGCCACCAAAAATCGATGGAGAAATTGAAGGAGAAAA
TTGCAGC
GAAGAAGGAATCGGTATCAGATGGAGAACGACAAGTTAAGGATGCGCAGAAAGAAGCTAGACATAGTGGTAGT
GTAAGAG
ACAAGCAGGTCTATGAGAAAAAGAAGAAAATGTTAGAGCGGCTCAAAGAACAACTGGCAAAACTCGAAATTCA
AGAGACA
GACCGAGATGAAAACAAGACAATAGCTCTTGGTACGTCAAAACTGAATTATTTGGATCCCAGAATTTCCGTCG
CCTGGTG
TAAGAAGTATGGTGTGCCCATTGAAAAAATTTATAACAAAACTCAA------
CGTCTGCCCCCATTCCGTGTCATTGGTG
ACAATCTCAAGGACCGTTTCGACGGCGCATCACGCGTCATGTTAAGCAACTCCGCCAACTCTAGGAA------
------C
AGTAA---AAATCGCC---
CTAAACAAGACAAACTTTCCAACAACATCGCATCCAACAGTATCCACAGCAAACGCGAAAA
CAGACCACGCAAGTACAAATACGGCTTCCAACTCAAACCATACAATCCTGACCACAAACCTCCGAGTCCTAAA
GACCTGG
TCTACCTAGAAACCTCACCCGGCTTTTGCGAGAAGAACCCAAAACTGGGCATACAAGGCACACATAGCAGGCA
A-TGTAA
CGACACCTCTATCGGCGTCGACGGTTGCGATCTCATGTGCTGTGGCAG-
GGGTTACAGAACACAAGAGGTCGTCGTTGTA
GAGAGGTGCAACTGCACG-------------
>Pseudolathra
GGGAGAAGCCCAGCACTGAATCCCGTGGCCCTACCGGGAAATGTAGTGTTTGGGAGGACCCGAT-
TACGCCGTGCGGCGC
GTCCAAGTCCTTCTTGAACGGGGCCACATACCCATAGAGGGTGCCAGGCCCGGCGTCTGGAGGGTCTCTCCTC
AGAGTCG
GGTTGCTTGAGAGTGCAGCCCTAAGTGGGTGGTAAACTCCATCTAAGGCTAAATATGACCACGAGACCGATAG
CGAACAA
GTACCGTGAGGGAAAGTTGAAAAGAACTTTGAAGAGAGAGTTCAATAGTACGTGAAACCGTTCAGGGGTAAAC
CTGAGAA
ACCCGAAAGGTCGAATGGGGAGATTCAGCGTGTCTCGTTT-
TAGGCGCGTGACGTTGGTGCTCGCACCGGACCGCACCGT
AATGACCCGTTGCTTGTGACGAACCCGTGCACTTCTCCCCTAGTAGGACGTCGCGACCCGTTGGGTGCCGGTC
TAAGGCC

GACTGTGGCGCCTTGAGGTCCCGGCCGGCTCGCTCGACGGTAAGACAGAGGCGTGGGGTCGCGATGTTCGCGT
CCGGCCC
GTCACAAGCACAGGCGACTCGGACGTCGGACCTGTGTGCCGACCTCGAGATCGCCGACTGTTGGTGACGGTGT
CCTCGGA
CAGACTACACGTCGGTCGGCGACGCTTTAGCTTTGGGTTTTCAGGACCCGTCTTGAAACACGGACCAAGGAGT
CTAGCAT
GTGCGCGAGTCATTGGGACTCTAACTAAACCTAAAGGCGAAATGAAAGTGAAGGCGTGCCGAGGGAGGATGGG
TCGGGGG
GCGTCTCGTTCTCACCGCGAGATGAGGCGCACCCAGAGCGTACACGCGCTTACACCGTATTCGCTGACTTGTT
CGA-TCC
CATTATTGAAGATTACCATGGTGGATTCAAGAAGACCGATAAGCACCCTCCTAAGAACTGGGGTGATGGCAAT
GTCTTCA
CCAATCTCGACCCTGCTGGTGAATATGTCGTCTCCACCCGTGTCCGTTGCGGTCGCTCCATGGAGGGTTACCC
CTTCAAC
CCTTGCTTAACCGAAGAGCAATACAAGGAGATGGAACAAAAAGTTTCCTCCACTTTGTCTGGTCTCGAAGCTG
AACTCAA
GGGTACTTTCTACCCATTGACTGGAATGGATAAAGATACTCAACAGAAACTCATCGATGATCACTTCTTGTTC
AAAGAGG
GAGATCGTTTCCTCCAGGCCGCCAATGCATGCCGTTTCTGGCCATCTGGACGTGGCATCTACCACAACGACAA
CAAAACA
TTCTTGGTCTGGTGCAACGAAGAGGATCATCTTCGTATCATCTCTATGCAAATGGGTGGAGATCTTGGTGAAG
TTTACCG
CCGTTTGGTCACCGCTGTCAACGAAATCGAGAAACGCGTACCCTTCTCCCATAACGACAGATTAGGTTTCCTC
ACTTTCT
GCCCAACCAACTTGGGCACCACTGTCCGTGCCTCCGTGCACATCAAGGTCCCCAAGCTCGCCGCCAACAAAGC
TAAACTC
GACGAAATCGCCGCCAAGTACAACTTGCAAGTTCGTGGCACCCGT---
GTGCCTAAAAAAGTATTAATAATTGGATCTGG
TGGCCTGTCAATTGGACAAGCTGGAGAATTTGATTATTCTGGATCACAAGCCATCAAAGCTTTAAAAGAAGAA
AACATAA
GAACTGTGTTAATAAACCCTAATATTGCAACGGTGCAAACTTCTAAAGGTTTAGCTGATAAAGTATATTTTCT
ACCATTA
ATTCCTGAATATGTTGAACAAGTAATTCAATCAGAAAGACCGACGGGTGTTTTGTTAACATTTGGAGGTCAAA
CAGGATT
AAATTGTGGTGTAGCTTTGCAAAAAGCAGGTGTATTTGATAAATATGGTGTTAAAATATTAGGTACCCCTATA
AAAGCCA
TAATTGATACAGAAGACAGAAAAATATTTAGTGAGAGGATTGCGTCTATAGGAGAGAAAGTGGCTCCTAGTAT
GGCTGCT
TATTCAGTCCAAGAAGCCTTAGAAGCGGCAGAATTACTTGGATATCCTGTCATGGCAAGAGCTGCATTTTCAT
TGGGAGG
TTTAGGGTCAGGATTTGCTGACAACTCTGAAGAATTGAAATCCCTTGCACAACAAGCTTTAGCTCATTCGAAT
CAGTTAA
TAATTGATAAGTCATTAAAGGGCTGGAANGAAGTTGAATATGAAGTAGTAAGAGATGCTTAT-----------
----

ATCTTCCAGGATTTGGAATAATTTCTCATATTATTAGACAAGGTAGAGGAAAAAGGAAACCTTCGGAGCACT
AGG
TATAATTTATGCTATAATAGCTATTGGATTATTAGGATTTGTAGTATGAGCTCACCATATATTTACTGTTGGA
ATAGATG
TTGATACTCGGGCTTATTTTACTTCAGCCACTATAATCATTGCTGTTCCTACAGGAATTAAGATTTTTAGATG
ATTAGCT
ACTCTTCATGGATCCCAACTAACATTTAACCCACCTATTTTATGATCTTTAGGATTTGTTTTTTTATTTACAA
TTGGAGG
ATTAACTGGAGTAATTTTAGCTAACTCATCAATTGATATTATTTTACACGATACTTACTATGTTGTTGCCCAT
TTTCATT
ATGTATTATCTATAGGAGCAGTATTTGCAATTATAGCAGGATTAATACAATGATTTCCACTATTTACAGGTCT
TTCTATA
AATGAATATTTATTAAAAATTCAATTTTTAATTATATTTATTGGAGTAAACCTAACCTTTTTCCCTCAACATT
TTCTTGG
ATTAGCAGGAATACCCCGACGATACTCAGACTACCCTGACGTTTATACTCCATGAAATGTAATCTCATCAATT
GGTTCTT
TAATTTCTATAATTAGAATTATATTACTATTATACATTCTTTGAGAAAGATTCTCTTCTATACGAATTATCTT
ATCTGCT
AAAAACTTTTCTACTTCAATCGAATGATTTCAATACTATCCTCCTGCTGAACACAGATAT------------
----
-AAAGATTACCTTGTCGTCTTTGATTTCCTTGGT-
AAAGATTCCATTAGATACTATAATGAAGTACCTGTAGAAAAACGT
GTTTTCAAGAATCTCCAGTTGTTCATGGAAAATAAATCACCGGGTGATGATTTATTCGATCGTTTAAACACCG
CTGTGAT
GAACAAACACTTGAACGAGTTAATGGAGGGTCTAACCGCCAAGGTATTTCGTACTTACAATGCTTCTTGGACA
TTGCAAC
AACAACTTGATAAGTTAACAAATGAAGATGATTCGATATCCGAGAAAATTTTATCATACAATCGTGCCAATCG
TGCTGTC
GCCATTCTATGTAACCATCAACGTGCGGTGCCTAAAGGCCATCAGAAATCCATGGAGAAGTTAAAGGAAAAAA
TTGAGAC
TAAGAAAGACGCCATTAGGGACGGGGAGCGTCAAGTGAAAGACGCACAAAAGGATGCGAAACA---
TGGAAGTGTGAGGG
AAAAGCAAATCTACGATAAGAAGAAGAAAATGTTGGAACGCCTCAAAGAACAATTAGCGAAACTGGAAATCCA
AGAGACG
GATCGTGATGAAAACAAAACTATTGCGCTCGGTACGTCAAAGTTGAATTATTTAGATCCGAGGATTTCCGTTG
CATGGTG
TAAGAAGTTCGGTGTACCCATTGAAAAGATCTATAATAAAACTCAA------
CGTTTGCCGCCGTTCCGTGTGATCGGTG
ATCATTTGAAGGATCGCTTCGATGGGGCGTCCCGTGTGATGTTGAGTAATTCAGCGAGCGCACGTGGCGG---
------T
AACGC---GAACAGGC---
CCAAGCAGGATAAGCTGTCGAATAATATCGCGTCGAATAGTATTCATAGTAAAAGGGAGAA
CAGGCCTCGGAAGTATAAGTACGGTTTTCAATTGAAGCCGTACAATCCGGACCATAAGCCTCCGAGTCCTAAG
GATTTGG
TGTACTTGGAGCCGTCGCCTGGTTTNTGCGAGAAGAATCCGAAGCTTGGTATCCAGGGGACGCACGGCAGGCA
G-TGTAA
TGACACGTCGCTGGGGGTGGACGGGTGCGACCTG--------------------------------------
----

>Pseudomedon
GGGAGAAGCCCAGCACTTAATCCCGTGGCCGAACCGGGAAATGTAGTGTTTGGGAGGGTCCATCATCCATCGT
ACGACGC

GTCCAAGTCCTTCTTGAACGGGGCCACATACCCATAGAGGGTGCCAGGCCCGGTAGCTGGTGGATCTCTCCTC
AGAGTCG
GGTTGCTTGAGAGTGCAGCCCTAAGTGGGTGGTAAACTCCATCTAAGGCTAAATATGACCACGAGACCGATAG
CGAACAA
GTACCGTGAGGGAAAGTTGAAAAGAACTTTGAAGAGAGAGTTCAATAGTACGTGAAACCGTTCAGGGGTAAAC
CTGAGAA
ACCCGAAAGGTCGAATGGGGAGATTCAGCGTGTCTCGTTTTCGGTCGCGTGACGAAGGTGCTTGCACCGGGGC
GCGCCTT
CCGGATCCGTAACCTGCGACGAACCCGTGCACTTCTCCCCTAGTAGGACGTCGCGACCCGTTGGGTGCCGGTC
TAAGGCC
GACGGTGGAGCCTTGGGGTCCCGGCCGGCCCGCCCGACGGTAAGACAGAGGCGTGGGGTCGCTACGTTAGCGT
CCGGCCC
GTGACAAGTTCGAGCGACTCGGATGTCGGACCTGCGTGCCGACCCCGAGCTCGCCGGCTGTTGGTTACGGTGT
CCTCGGA
CAGACTACACGCCGGTCGGCGACGCTTTAGCTTTGGGTTTTCAGGACCCGTCTTGAAACACGGACCAAGGAGT
CTAGCAT
GTGCGCGAGTCATTGGGACCGCATCTAAACCTAAAGGCGAAATGAAAGTGAAGGCGTGCCGAGGGAGGATGGG
TCGGGGG
GCGTCTCGTTCTCATCGCGAGATGAGGCGCACCCAGAGCGTACACGCTCTTACACCGTATTCGCTGATTTGTT
CGA-TCC
CATTATTGAAGATTACCATGGTGGATTCAAGAAGACCGACAAGCACCCTCCCGCAAACTGGGGTGATGTCAAC
ACTTTCG
CAAACCTCGACCCAGCTGGCGAGTACGTAGTCTCAACCCGCGTCCGTTGCGGCCGCTCAATGGAAGGCTACCC
ATTCAAC
CCATGCTTAACCGAGGAACAATACAAGGAGATGGAACAGAAGGTCTCCAGCACCTTGTCCGGTCTCGAAGGCG
AACTTAA
GGGTACTTTCTACCCATTGACTGGAATGGATAAGGATACTCAACAGAAGCTCATCGACGATCACTTCTTGTTC
AAGGAAG
GTGACCGTTTCCTCCAGACCGCCAACGCCTGTCGTTTCTGGCCATCTGGACGTGGTATCTACCATAACGACAA
CAAAACA
TTCTTGGTCTGGTGCAACGAAGAGGACCACCTTCGTATCATCTCCATGCAGATGGGTGGTGATCTTGGTGAAG
TCTACCG
TCGTCTCGTCACTGCTGTCAACGAAATCGAGAAGCGCGTCCCCTTCTCCCACAATGACAGATTAGGTTTCCTT
ACTTTCT
GCCCAACCAACTTGGGCACAACTGTACGTGCCTCTGTACACATTAAAGTACCTAAGCTCGCCGCCAACAAAGC
TAAGCTC
GATGAAGTCGCCGGAAAATACAACTTGCAAGTACGTGGTACCCGTGGTGTTCCAAAAAAAGTTTTAATTATTG
GTTCAGG
TGGTTTGTCTATTGGACAAGCCGGTGAATTTGATTACTCTGGTTCACAAGCAATTAAAGCATTGCAAGAAGAA
AATATAC
AAACAGTCCTTATTAACCCTAATATTGCTACAGTTCAAACATCGAAGGGTTTAGCTGATAAAATTTACTTCTT
ACCTTTA
GTGCCTGAATTTGTGGAGCAGGTAATTCGTGTAGAACGTCCTGGCGGTGTYTTGTTAACATTTGGAGGACAAA
CAGGGTT
AAATTGTGGTGTAGAATTACAAAAAGCTGGTATTTTTGAAAAATATGGTGTTAAGATTTTGGGTACACCTATA
CAAGCTA
TAATTGATACGGAAGACAGAAAAGTTTTTAGTGAAAGAATTGCTATGATTGGCGAGAAGGTAGCTCCAAGTAT
GGCCGCT
TATTCAGTACAAGAAGCACTCGAAGCTGCTGAATTATTGGGTTATCCAGTAATGGCAAGAGCCGCCTTTTCTT
TAGGTGG
TTTAGGATCTGGATTTGCAAATACTGCCGAAGAATTGAAATCACTTGCTCAACAAGCTTTAGCTCATTCAAAT
CAGTTAA
TTATTGATAAGTCTTTAAAGGGATGGAAGGAAGTTGAATATGAAGTTGTCAGAGATGCATATCCTTACATTAA
AGAAGTT
AATGACGAAGAACTACAAGAACCGACAGATAAAAGGATGTTTGTACTTGCAGCTGCTTTAAGAAATGGTTATA
GTGTTGA

TAAGTTGTATGATCT-
AACCAAAATTGATCGTTGGTTTTTACAAAAAATGAAAAATATCGTTGATTACAATACTGTACTA
GAATCGATACAACAAAATAAATTG---------------
ACATATAAAGTTTTATTAAAAGCCAAGCAAATTGGTTTTAG
TGATAAACAAATAGCTGTTTGTGTTAAAAGTACTGAACTTGCAATTCGAAAACAACGACAAGATTTTGATATA
ACTCCGT
ACGTTAAACAAATCGATACTGTAGCTGCTGAATGGCCTGCTACCACTAATTATTTGTATTTAACATACAATGC
TGAAACT
CATGACTTAACATATAACGAAAAACATATAATGGTTATAGGATCAGGAGTGTACCGTATTGGAAGTTCTGTTG
AATTTGA
TTGGTGTGCTGTAGGTTGTTTAAGAGAACTTAGAAAATTAAACAAAAAAACAATAATGGTCAATTACAATCCC
GAGACTG
TGAGTACAGACTATGATATGTCAGATAGATTATATTTTGAAGAAATATCGTTCGAAGTTGTTATGGATGAAGT
TTATATT
TTAATTCTTCCAGGATTTGGTATAATTTCCCATATTATTAGCCAAGCTAGGGGAAAAAAAGAAACTTTTGGAA
CCCTAGG
AATAATTTATGCTATAATAGCCATTGGTTTATTGGGTTTTGTTGTATGAGCCCACCATATATTTACTGTAGGT
ATAGATG
TAGATACCCGGGCTTATTTCACATCGGCAACAATAATTATTGCTGTTCCAACAGGAATTAAAATTTTTAGCTG
ATTAGCT
ACACTACACGGAACACAAATAAAATTTAATCCCCCAATATTATGATCTCTAGGATTTGTTTTTTTATTCACTA
TTGGAGG
ATTAACCGGAGTAATTTTAGCTAATTCATCAATTGATATTATCCTTCATGATACTTACTATGTCGTAGCTCAT
TTCCATT
ATGTTCTTTCCATAGGAGCAGTATTTGCTATTATAGCAGGTCTAGTTCAATGATTCCCCTTATTTACAGGATT
AACCTTA
AATGAATACATATTAAAAATTCAATTTTTTATTATATTTATTGGAGTAAATATAACCTTTTTTCCCCAACACT
TCTTAGG
ACTAGCTGGCATGCCCCGTCGTTATTCCGACTATCCAGATGCATATACCCCATGAAATGTAATTTCATCAATT
GGTTCTT
TAATCTCAATAATTAGAATTTTCCTTTTACTATTTATTTTCTGAGAAAGGTTTACTTCTATACGAATTGTTAT
TTCTTCA
AAAAATTATGTCACTTCAATTGAATGATTACAATTACATCCACCAGCAGAACATAGCTATTCTGAATTACCCA
TATTAAC
TAAAGATTATGTAGTAGTATTTGATTTCCTTGGT-
AAGGATTCAATTAGATACTACAATGAGGTACCTGTAGAAAAACGT
GTCTTCAAAAATCTACAATTGTTTATGGAAAATAAATCACCAGGCGATGATTTGTTTGATAGATTAAACACAG
CTGTGAT
GAACAAACATTTAAACGAGCTAATGGAAGGATTAACTGCCAAGGTGTTTCGTACTTATAACGCTTCATGGACC
TTACAAC
AGCAACTAGACAAATTGACCGACCCAAACGATTCCATATCCCAAAAAATTTTATCTTACAACAGAGCAAATCG
AGCTGTC
GCTATACTTTGCAACCATCAACGTGCAGTACCGAAAGGCCACCAAAAATCTATGGAGAAACTTAAGGAAAAAA
TCGAAGC
CAAAAGAGAGTCTATCAGAGATGGCGAGAGACAAGTGAAAGACGCGCAGCGTGACGCAAAGCA---
TGGCAGCGTCAAAG
AAAAACAGATATACGAGAAGAAGAAGAAAATGTTGGAGAAACTCAAAGAGCAACTGACTAAACTGGAGATTCA
GGAGACG
GACCGCGACGAAAACAAAACCATTGCTCTCGGTACGTCGAAACTGAATTATTTGGACCCGAGAATTTCGGTCG
CATGGTG
TAAGAAGTTTGGTGTGCCCATTGAAAAAATTTATAACAAAACTCAA---------------------------
----
-----------------------------TCGCGCGTGATGCTGAGCAACTCGGCGAGCTCGCGCGG------
------C
GGCAA---CGCGAGGC---
CCAAGCAGGATAAGCTCTCGAACAACATCGCCTCGAACAGCATACACAGCAAGAGAGAAAA

CCGGCCGCGCAAATACAAGTACGGGTTCCAATTGAAGCCGTACAATCCGGACCACAAGCCTCCGAGCCCTAAG
GATTTGG
TGTATCTGGAACCSTCGCCCGGTTTCTGCGAGAAGAACCCGAAGCTCGGGATACAGGGCACGCRCGGTAGGTT
A-TGCAA
CGATACTTCGATCGGGGTGGACGGGTGCGATCTGATGTGCTGCGGGAG-
AGGATACAGGACCCAGGAGGTTATCGTTGTC
GAGAGGTGCAACTGC----------------
>Quedius_molochinus
GGGAAGAGCCCAGCACCGAATCCCGCGTCCGTGCCGGGAAATGTGGTGTTAGGGAGGGTCCGCCATCCGTCGC
GCGGTCG
GTCCAAGTCCTTCTTGAACGGGGCCACTTACCCATAGAGGGTGCCAGGCCCGGCAACGGGAGGATCTCTCCTC
AGAGTCG
GGTTGCTTGAGAGTGCAGCCCTAAGTGGGTGGTAAACTCCATCTAAGGCTAAATATAACCACGAGACCGATAG
CGAACAA
GTACCGTGAGGGAAAGTTGAAAAGAACTTTGAAGAGAGAGTTCAATAGTACGTGAAACCGTTCAGGGGTAAAC
CTGAGAA
ACCCGAAATGTCGAATGCAGAGATTCAGCGTGTCTCGCTGGTGGTTGTGTGACGGCGACGAACGCGTC-
GACCGCTCCTT
CCGCCGCCG--
ATTCGCGGCGAACGCGTGCACTTCTCTGCTAGTAGGAAGTCGCGACCCGTTGGGTGCCGGTCTAAGGAC
CGCGGTGGAGCCCGAGTGTCCCGACCGGCTCGCTCGACGGTACGACAGTGGCGCGGGGCCGCGATCTTCGCGT
CCGGCCC
GTCGCAAGTACGGACGGTCTGGATGTCGGACCTA-
GTGCCGACTTCGGAGCCGCCGGCTGCTGGCGACGGTGTCCTCGGA
CAGACTATACGCCGGTCGGCGACGATTTAGCTTTGGGTTTTCAGGACCCGTCTTGAAACACGGACCAAGGAGT
CTAGCAT
GTGCGCGAGTCATTGGGACTT-
AGCGAAACCTAAAGGCGTAATGAAAGTGAAGGCGCGCCTAGGGTGGATGCGCCGGGGG
GCGTCTCGTGCTCATTGCGAGCTGAGGCGCACCCAGAGCGTACACGCGCTTACACCGTATTCGCCGACTTATT
CGA-CCC
CATCATCGAAGATTACCATACAGGCTTCAAGAAGACCGACAAGCATCCACCAAAGAACTGGGGCGATGTCAAC
ACCTTTG
CCAATCTCGACCCTGCTGGCGAGTACGTCGTATCCACCCGCGTCCGTTGCGGTCGCTCCATGGAGGGATACCC
CTTCAAC
CCCTGTTTAACCGAAGACCAATACAAGGAGATGGAACAGAAGGTCTCCACCACCCTCTCTGGACTTGAGGGTG
AACTCAA
GGGTACCTTCTACCCATTGACTGGCATGGGCAAGGACGTCCAACAAAAACTGATTGATGACCATTTCCTCTTC
AAGGAAG
GAGATCGCTTCCTCCAGGCCGCCAACGCTTGCCGCTACTGGCCCAGCGGACGTGGCATCTACCACAACGACAA
CAAGACC
TTCTTGGTCTGGTGCAACGAAGAGGACCATCTCCGTCTTATCTCCATGCAAATGGGTGGCGATCTTGGTGAAG
TTTACCG
TCGTCTCGTAAATGCCGTCAACGATATCGAAAAGCGCGTTCCTTTCTCTCACAATGACAGATTAGGTTTCCTT
ACTTTCT
GCCCAACCAACTTAGGCACAACTGTTCGTGCCTCCGTACACATTAAGGTCCCCAAGCTCGCCGCCAACAAGGC
TAAGCTC
GACGAAATCGCCGGCAAATACAACTTGCAAGTCCGCGGAACCCGT---
GTGCCGAAAAAGGTGTTGATAATCGGTTCGGG
TGGATTGTCGATCGGTCAGGCGGGCGAGTTTGATTATTCGGGGTCTCAGGCGATTAAGGCGTTGCAGGAGGAG
GGGATTC
AGACGGTTTTGATCAATCCGAATATAGCTACGGTGCAGACGTCAAAGGGTTTAGCGGATAAAGTTTACTTTCT
ACCGTTA
GTGCCCGAATACGTGGAGCAGGTGATTCGTGTGGAGAGACCTGGAGGGGTGCTGCTGACGTTCGGCGGACAGA
CTGGATT
GAATTGCGGCGTTGAGTTGGAGAGGGCTGGAGTTTTTAAGAAGTATAATGTTAAGATTCTTGGGACACCGATA
CAAGCGA

TTATAGATACGGAGGATAGGAAGGTGTTTAGTGATAGGATCGGGCAGATTGGGGAGAAGGTCGCGCCTAGCAT
GGCCGCG
TATTCCGTGCAGGAGGCGCTTGAAGCTGCGGAGAAGCTGGGGTACCCCGTGATGGCGAGAGCGGCTTTTTCTT
TGGGGGG
ACTTGGATCGGGCTTCGCGGATACTAAAGAGGAACTCAAGTCGCTCGCGCAACAAGCATTGGCCCATTGGACT
CAGTTGA
TTATCGATAAGTCGTTGAAAGGATGGAAGGAGGTGGAGTACGAGGTGGTGAGGGATGCTTTTTCGTATCTNAA
AGAGGTC
GACGATGAGGATCTNAAGGAACCGACCGACAAGCGGATGTTCGTGGTCGCGGCCGCGTTAAGATCCGGATATT
CCGTGGA
TAAACTTTACGATTT-
AACGAAAATCGATCGTTGGTTTCTGCAAAAGATGAAGAATGTGGTGGATTATAATTCATTCCTG
CAAACCATCGATCAGATCAATCTT---------------
ACGAAAGACAACCTGTTACGCGCGAAGCAAATTGGTTTTAG
TGATAAACAAATTGCCGTCGCTGTTAAGAGCACAGAACTGGCTATCAGGAAGCAAAGGCAGGATTTTAACCTC
ACCCCCT
ACGTGAAGCAAATCGACACAGTCGCCGCGGAATGGCCTGCGACAACCAACTACCTTTACCTAACGTACAACGC
GGTAGCN
AACGATTTGACTTTCGCNGAAGAACATACTATGGTGATAGGTTCTGGCGTATACCGCATCGGAAGCTCCGTAG
AATTCGA
CTGGTGCGCGGTGGGTTGCCTGCGCGAGTTACGAAAACTCGGTAAGAAGACAATAATGGTAAATTACA-----
----

GAAGTCTATATT
TTAATCCTACCTGGATTTGGGATAATCTCCCATATTATTAGGCAGGAAAGAGGAAAAAAAGAAGCCTTTGGAA
CGTTAGG
GATAATTTATGCAATAATAGCAATTGGTTTATTAGGATTTATTGTATGAGCCCATCACATATTCACAGTAGGA
ATAGATG
TTGATACACGAGCTTATTTTACTTCAGCAACAATAATTATTGCTGTTCCAACAGGAATTAAAATTTTTAGGTG
AATGGCT
ACATTACATGGAACTCAAATTAATTACTCCCCCTCAATGATTTGAGCTTTAGGTTTCGTATTTTTATTTACAG
TTGGTGG
TTTAACTGGAGTAGTATTAGCTAATTCATCTATCGATATTATTTTACATGATACCTATTATGTTGTTGCCCAT
TTTCACT
ATGTTTTATCAATAGGGGCAGTATTTGCTATTATAGCAGGATTAATACAATGATTTCCTTTATTCACAGGTTT
AACTTTA
AATGAAAAATTTTTAAAAATTCAATTTTTTTCTATGTTCATTGGAGTAAATTTAACTTTTTTCCCTCAACATT
TTTTAGG
ATTAGCTGGAATACCTCGACGATACTCCGATTACCCGGATATTTATACACCTTGAAATGTAATTTCATCAATC
GGATCTT
TAATTTCAACCATAAGAATTTTTTTACTATTATTTACCATTTGAGAAAGATTTGTATCTACTCGAAAAAGAAT
ATCCCCA
TTAAATTTACCATCCTCAATTGAATGGCTTCAATCTATACCACCTGCTGAGCATAGATAT-------------
----
-AAGGAGTACGTAGTCGTGTTTGATTTCCTCGGT-
AAAGATTCTATTCGGTATTACAATGAGGTGCCCGTCGAGAAGCGC
GTTTTTAAAAATCTCCAATTGTTCATGGAGAACAAGGCTCCGGCTGATGATCTGTTTGATCGGTTGAACACCG
CCGTTAT
GAACAAGCACTTGAACGAGCTGATGGAAGGTTTGACCGCAAAGGTATTCCGTACTTACAATGCCTCGTTTACT
CTGCAAC
AGCAGCTGGATAAGTTAACGAACGAAGATGATTCCTTATCAGAGAAAATTTTATCTTACAACAGAGCGAATCG
AGCTGTT
GCCATACTGTGTAACCATCAACGTGCTGTTCCGAAGGGACATCAGAAGTCGATGGAGAAGCTGAAGGAGAAAA
TTGACAG
CAAACGGGACGCTATCGCTGACGGTGAACGACAAGTCAAGGATGCGCAAAGAGAGGCCAAGCA---
CGGAAGTGTAAAAG

AGAAGGTGAATTTCGATAAAAAGAAGAAAATGTTGCAGAGATTAAAGGAACAGTTGGCTAAATTAGAAATTCA
AGAGACG
GACAGGGATGAGAATAAGACAATTGCTTTGGGCACGTCCAAGCTGAATTATTTGGATCCTAGGATATCTGTGG
CATGGTG
CAAAAAGTTTGATGTGCCGATTGATAAGATCTACAACAAAACTCAA--------
GTTACCACCTTTCCGCGTCATTGGTG
ATAACCTGAAGGACCGTTTCGACGGTGCTTCACGGGTAATGCTTAGTAACACGGCAAACTCGAGGAATAGCAA
CAATGCC
CACAA---CAGCCGCC---
CGAAACAAGATAAGCTCTCGAATAGTATCGCATCCAATAGTATACACAGCAAACGCGAGAA
CAGGCCGCGCAAATACAAGTACGGTTTCCAATTAAAACCATACAATCCTGACCACAAGCCGCCTAGTCCTAAA
GATCTGG
TCTACTTGGAGCCGTCGCCTGGTTTTTGCGAGAGGAATCCCAAATTGGGCATACAGGGCACTCACGGCAGACA
A-TGCAA
CGACACTTCGATTGGAGTTGACGGCTGCGATCTT---------------------------------------
----

>Ronetus
GGGAGAAGCCCAGCACTGAATCCCGTGGCCGAACCGGGAAATGTAGTGTTTGGGAGGGTCCGTTATCCATCGT
GCGGCGC
GTCCAAGTCCTTCTTGAACGGGGCCATATACCCATAGAGGGTGCCAGGCCCGATAGCTGGAGGATCTCTCCTC
AGAGTCG
GGTTGCTTGAGAGTGCAGCCCTAAGTGGGTGGTAAACTCCATCTAAGGCTAAATATGACCACGAGACCGATAG
CGAACAA
GTACCGTGAGGGAAAGTTGAAAAGAACTTTGAAGAGAGAGTTCAATAGTACGTGAAACCGTTCAGGGGTAAAC
CTGAGAA
ACCCGAAAGGTCGAATGGGGAGATTCAGCGTGTCTCGTTTCTGGTTTCGTGACGATGGTGCTTGCACCGGGTA
GCGCATT
CCGGATCCGTAACCGGCGACGAACTCGTGCACTTCTCCCCTAGTAGGACGTCGCGACCCGTTGGGTGTCGGTC
TAAGGCC
GACGGTGGAGCCTTGGGGTCCCGACCGGCCCGCTCGACGGTGAGACAGAGACGTGGGGTCGCTTCGTAAGCGT
CCGGCCC
GTCACAAGTCCGGACGACTCGGACGTCGGACCTGTGCGCCGACCTCGAGCTCGCCGGCTGCTAGTGACGGTGT
CCTCGGA
CAGACTACACGTCGGTCGGCGACGCTTTAGCTTTGGGTTTTCAGGACCCGTCTTGAAACACGGACCAAGGAGT
CTAGCAT
GTGCGCGAGTCATTGGGACCGCATCTAAACCTAAAGGCAAAATGAAAGTAAAGGCGTGCCGAGGGAGGATGGG
TCGGGGG
GCGTCTCGTTCTCATCGCGAGATGAGGCGCACCCAGAGCGTACACGC--------------------------
-GA-TCG
CATTATTGAAGATTACCATGGCGGATTCAAGAAGTCCGACAAGCACCCACCCGCGAACTGGGGCGACGTGAAC
ACCTTCA
CCAACCTGGACCCTGCTGGAGAGTACGTAGTGTCCACCCGCGTCCGTTGCGGCCGTTCCATGGAAGGATACCC
CTTCAAC
CCGTGCTTGACCGAAGAACAATACAAAGAAATGGAAGGCAAAGTGTCCAGCACCTTGTCTGGCCTCGAAGGAG
AACTCAA
GGGCACTTTCTATCCATTAACCGGCATGGACAAGGCAACTCAACAGAAGCTCATAGATGATCACTTCTTGTTC
AAGGAAG
GAGACCGTTTCCTCCAGGCGGCCAACGCCTGCCGCTTCTGGCCATCCGGGCGTGGTATCTACCACAACGACAA
CAAAACC
TTCTTGGTCTGGTGCAACGAAGAGGATCATCTCCGTATCATCTCCATGCAGATGGGCGGCGACCTTGGTGAAG
TATACCG
TCGCCTCGTGACAGCCGTCAACGAAATCGAGAAGCGCGTCCCATTCTCCCACAATGACAGATTAGGTTTCCTC
ACCTTCT
GCCCAACCAATTTGGGCACAACTGTTCGTGCCTCTGTACACATCAAGGTACCAAAGCTCGCAGCCAACAAGGC
CAAGCTC

GACGAAGTCGCTGGCAAATACAACTTGCAAGTACGTGGTACTCGCGGTGTTCCAAAAAAAGTTTTAATCATCG
GTTCAGG
TGGATTGTCCATAGGACAAGCTGGCGAATTCGATTATTCCGGTTCACAAGCGATAAAGGCCTTACAAGAAGAA
AACATAC
AAACAGTCCTGATCAACCCAAACATAGCAACCGTGCAAACGTCTAAAGGTCTAGCTGATAAAATTTATTTTTT
ACCATTG
GTGCCTGAATTTGTCGAACAAGTGATCAGAGCAGAACGTCCTGGAGGGGTTCTGCTTACATTCGGTGGACAAA
CAGGGTT
GAATTGCGGTGTAGAACTTCAGAAAGCGGGTGTATTTGAAAAGTACGGCGTTGAAATTTTGGGCACTCCCATA
CAAGCAA
TTATTGATACTGAAGACAGGAAGATTTTTAGTGAAAGAATTGGTTTCATAGGTGAAAAAGTTGCACCAAGTAT
GGCGGCA
TATTCTGTACAAGAAACATTGGAAGCAGCTGAGTTATTAGGTTATCCTGTAATGGCAAGGGCAGCTTTTTCAC
TAGGCGG
TTTAGGATCTGGGTTCGCTAATAATGATGAAGAACTGAAGACTCTTGCTCAACAAGCGTTAGCT---------
----

------T
TTAATCTTACCAGGATTTGGGATAATTTCCCATATTATTAGACAATCTAGAGGTAAAAAAGAATCTTTTGGTT
CCTTAGG
AATAATTTACGCAATAATAGCTATTGGATTATTAGGATTTGTAGTATGAGCTCATCATATATTCACAGTAGGA
ATAGATG
TAGACACTCGAGCATACTTCACATCAGCAACAATAATTATTGCAGTACCAACAGGAATTAAAATTTTTAGATG
ATTAGCT
ACTTTACATGGCACTAAAATTAAAATTTCCCCCCCTATTCTTTGAGCTATTGGATTTGTATTTTTATTTACAG
TTGGTGG
ATTAACAGGAGTAATTTTAGCTAATTCATCAATCGATATCATCCTCCATGATACTTATTATGTGGTTGCCCAT
TTCCATT
ATGTCCTTTCAATAGGAGCTGTTTTTGCCATTATAGCAGGACTAGTCCAATGATATCCTCTATTTACTGGGTT
ATCTATA
AATAATTTTTACTTGAAAGTTCAGTTTTTTATTATATTTATAGGCGTAAATATAACTTTTTTCCCTCAACATT
TTTTAGG
TTTAGGAGGTATACCTCGGCGATATTCAGATTATCCAGATGCTTACACTACTTGAAACATTATTTCTTCAATT
GGATCAA
TAATTTCAATAATAAGAATTTTTTTCTTAATTTTTATTATTTGAGAAAGTTTTGCGTCTATACGAATTAACAT
TTCCCCT
AAAAACCTTTCTACTTCTATCGAATGAATACAATTAACCCCACCATCTGAACATAGATATA------------
----
-AAAGATTATGTGGTAGTATTTGACTTCCTCGGT-
AAGGATTCTATTAGATACTACAATGAAGTACCTGTGGAAAAACGT

GTCTTCAAAAACCTTCAGTTGTTCATGGAAAACAAATCCCCAGGGGATGATTTATTTGATAGATTAAACACAG
CTGTAAT
GAACAAACATTTAAACGAACTAATGGAAGGTTTAACTGCCAAGGTGTTTCGTACTTACAATGCTTCTTGGACT
CTTCAAC
AGCAACTAGATAAATTGACCAATCCTGATGATTCCATATCAGAAAAAATATTATCATACAATCGAGCCAACAG
AGCGGTA
GCTATTCTATGTAACCATCAACGTGCTGTCCCTAAGGGCCACCAAAAATCCATGGAAAAACTCAAAGAAAAAA
TTGATGC
CAAAAAAGAAGCTATAAAAGATGGCGAGAGACAAGTTAAAGATGCACAAAAAGATGCGAAGCG---
TGGTAGCGTAAAGG
AAAAACAAATTTATGATAAGAAGAAGAAAATGTTGGACAGACTCAAAGAGCAGTTGGCAAAATTGGAAATTCA
GGAGACG
GACCGTGATGAAAACAAAACAATCGCCCTTGGTACTTCCAAGTTGAACTATTTGGATCCCAGAATTTCGGTCG
C------

TGGATGCGCCTGCCACCCTTCCGAGTGATCGGCG
ACCATTTAAAAGATCGATTCGACGGTGCCTCCAGGGTGATGCTGAGCAATTCCGCGAGTTCCCGCGG------
------G
AACGC---CAACAGGC---
CCAAGCAGGACAAACTCTCGAACAGCATCGCGTCCAACAGCATCCACAGCAAAAGAGAAAA
CCGCCCGCGCAAGTATAAGTACGGTTTCCAATTGAAGCCGTACAACCCGGACCACAAACCTCCGAGCCCGAAG
GACCTGG
TGTACCTGGAGCCGTCGCCTGGATTCTGCGAGAAGAACCCCAAGCTCGGCATACAAGGTACGCACGGTAGATT
G-TGCAA
CGACACCTCCATAGGTGTCGACGGCTGCGATTTGATGTGCTGCGGCAG-
GGGATACAGGACGCAGGAAGTCATCGTCGTC
GAAAGGTGCAACTGCACGTT----------
>Rugilus

GCCACATACCCATAGAGGGTGCCAGGCCCGATAGCTGGAGGATCTCTCCTCAGAGTCG
GGTTGCTTGAGAGTGCAGCCCTAAGTGGGTGGTAAACTCCATCTAAGGCTAAATATGACCACGAGACCGATAG
CGAACAA
GTACCGTGAGGGAAAGTTGAAAAGAACTTTGAAGAGAGAGTTCAATAGTACGTGAAACCGTTCAGGGGTAAAC
CTGAGAA
ACCCGAAAGGTCGAATGGGGAGATTCAGCGTGTCTCGTTTCTGGTCGCGTGACGATGGTGCTTGCACCGGGCC
GCGCCCG
CCGGATCCGTATCCGGCGACGAACTCGTGCACTTCTCCCCTAGTAGGACGTCGCGACCCGTTGGGCGCCGGTC
TAAGGCC
GACGGTGGAGCCTTGGGGTCCCGGCCGGCCCGCTCGACGGTAAGACAGAGGCGTGGGGTCGCTACGTTAGCGT
CCGGCCC
GTCACAAGTTCGGGCGACTCGGATGTCGGACCTGCGTGCCGACCCCGAGCTCGCCGGCTGCTGGTGACGGTGT
CCTCGGA
CAGACTACACGCCGGTCGGCGACGCTCTAGCTTTGGGTTTTCAGGACCCGTCTTGAAACACGGACCAAGGAGT
CTAGCAT
GTGCGCGAGTCATTGGGACCGCATCTAAACCTAAAGGCAAAA----------------------------
----

CTTACACCGTGTTCGCTGACTTGTTCGA-TCC
CATTATTGAAGATTACCATACTGGATTCAAAAAGACTGACAAGCACCCACCTAAGAATTGGGGTGATGTAAAT
ACCTTTG
CAAATCTTGATCCCGCTGGTGAATACGTAGTATCCACCCGCGTTCGTTGCGGCCGTTCAATGGAAGGTTATCC
TTTCAAC
CCATGCTTAACTGAAGAGCAATATAAAGAAATGGAAGGCAAAGTCTCAAGCACTTTGTCCGGCCTCGAAGGTG
AACTCAA

GGGTACTTTCTATCCGTTGACTGGAATGGATAAAGATACTCAGCAAAAGCTAATCGATGACCACTTCTTGTTC
AAGGAAG
GTGATCGCTTCCTTCAGGCTGCTAATGCCTGTCGTTTCTGGCCATCCGGCCGTGGTATCTACCACAACGACAA
CAAAACC
TTCTTGGTCTGGTGCAACGAAGAAGATCATCTTCGTATCATCTCCATGCAGATGGGTGGTGATCTTGGTGAAG
TCTATCG
TCGTCTTGTATCTGCTGTTAACGAAATTGAGAAGCGTGTTCCATTCTCCCACAATGACAGATTAGGGTTCCTC
ACTTTCT
GCCCAACCAATTTGGGCACAACTGTACGTGCCTCTGTACACATCAAAGTACCTAAACTTGCTGCCAACAAGGC
CAAGCTC

GTACCTAAAAAAGTTTTAATAATTGGTTCAGG
TGGTTTGTCCATTGGTCAAGCTGGAGAATTTGATTATTCAGGGTCTCAAGCTATAAAAGCTTTGCAAGAAAAC
AATATTC
AAACAGTTTTAATTAACCCAAACATTGCAACTGTACAAACATCGAAGGGTTTAGCTGATAAAGTATACTTTCT
ACCTTTA
GTGCCTGAATTCGTGGAACAAGTAATTAGAGTAGAACGTCCTGGAGGTGTTTTGTTAACATTTGGTGGACAAA
CAGGGTT
AAATTGTGGTGTAGAATTAGAAAAAGCTGGTGTATTTGAGAAATACAATGTTAAAATTTTGGGTACACCAATA
CAAGCAA
TAATAGATACTGAAGATAGAAAGATTTTTAGTGAAAGAATAGCATTGATTGGTGAAAAAGTTGCTCCTAGCAT
GGCTGCT
TATTCAGCACAGGAAGCTTTAGATGCTGCAGATTTGTTAGGTTACCCAGTTATGGCAAGAGCTGCCTTTTCTT
TAGGTGG
ATTAGGGTCTGGGTTTGCTAATACAGCTGAAGAACTGAAATTACTTGCTCAACAAGCTTTAGCTCATTCCACT
CAGTTAA
TTATCGATAAGTCTTTAAAAGGATGGAAAGAAGTTGAATATGAAGTTGTTAGAGATGCATATCCCTACCTCCA
AGAAGTT
AATGATGAAGAACTACAAGAACCTACAGATAAAAGAATGTTTGTTCTAGCAGCAGCTTTAAGAAATGGTTATA
GTGTAGA
TAAATTATATGATTT-
AACAAAAAATTGATCGCTGGTTCTTGCAAAAAATGAAGAATATTATAGATTACAACACTCTTCTT
GAGAAAGTTCAACAAAACAAATTACAAAACTGCTCAAATACATACAAACTTCTATTGAAAGCGAAACAAATTG
GTTTTAG
TGATAAACAAATTGCTGTTGCTGTTAAAAGCACAGAACTTGCAATTAGAAAACAACGACAAGATTTTGGAATT
ACTCCGT
ATGTTAAACAAATTGATACTGTGGCTGCTGAATGGCCTGCGACTACAAATTATCTGTACCTAACATACAATGC
AGAAAGT
CATGATTTACATTTTAGTGATCAACACATAATGGTTATTGGTTCTGGAGTTTATAGAATTGGAAGTTCTGTTG
AGTTTGA
TTGGTGTGCTGTTGGATGTTTGAGGGAGCTTAGGAAATTAAATAAAAAGACAATAATGGTTAATTACAATCCA
GAGACTG
TGAGTACAGATTATGACATGTCAGATCGGTTGTACTTCGAGGAAATTTCATTTGAAGTTGTAATGGATGAAGT
TTACATT
TTAATTCTTCCAGGATTTGGAATAATTTCTCATATTATTAGACAGGCTAGAGGAAAAAAGGAAACATTTGGTT
CATTAGG
AATAATTTATGCTATAATAGCAATTGGATTACTTGGATTCGTTGTATGAGCACATCATATATTTACTGTAGGA
ATAGATG
TAGATACACGAGCTTACTTTACTTCAGCAACAATAATTATTGCTGTTCCTACTGGAATTAAAATTTTCAGATG
ACTTGCT
ACTCTTCATGGAACTCAAGTTAAATATACCCCACCAATATTATGATCCTTAGGATTTGTATTTTTATTTACAA
TTGGCGG
ACTAACAGGTATTATTTTAGCTAATTCCTCAGTTGATATTATTTTACATGATACATATTATGTTGTTGCCCAT
TTTCATT
ATGTACTCTCAATAGGAGCTGTATTTGCTATTATAGCCGGTTTAGTGCAATGATTTCCCCTATTTACTGGATT
AACAATA

AATGAATATTTACTAAAAATTCAATTTTTTTAATATTTACTGGCGTAAATCTAACATTTTTTCCTCAACATT
TTTTAGG
ATTAGCTGGCATGCCTCGTCGATACTCAGATTATCCGGATGCTTATACCCCATGAAATGTAATTTCATCAATC
GGTTCAT
TAATCTCAATAATTTCAATTTTTATTTTATTGTTTATTATCTGAGATAGATTTATTTCTATGCGAATAAATTT
ATCAGCA
AAAAATTTTGCAACTTCAATTGAATGATTTCAACTATTTCCACCAGCTGAACATAGCTACTCAGAGCTACCTC
TGCTAAT
TAAAGATTATGTGGTAGTATTTGATTTCCTTGGT-
AAGGATTCTATTAGATATTATAATGAAGTACCTGTAGAGAAACGT
GTCTTCAAAAATCTCCAATTGTTTATGGAAAACAAGTCGCCAGYTGATGATTTGTTTGATAGATTAAACACAG
CTGTGAT
GAACAAACATTTAAATGAGTTAATGGAAGGCCTAACAGCAAAGGTGTTTCRTACTTATAACGCTTCGTGGACT
TTACAAC
AGCAACTTGAGAAATTGACCAATCCAGATGATTCCATATCCGAAWWWWTTTTATCTTACAACCGTGCCAATAG
AGCAGTA
GCTATACTTTGTAACCATCAACGTGCTGTACCTAAGGRTCATCAAAAATCAATGGAAAAACTAAAGGAKAAGA
TTGATAC
TAAAAGAGACAATATTAAAGATGCCGAGAGGCAGGTCAAAGATGCACAAAGAGATGCAAAGCA---
TGGAAGTGTCAAAG
AGAAACAGATTTATGACAAGAAGAAGAAGATGCTCGAGAGACTCAAGGAGCAACTGGCTAAACTGGAAATTCA
GGAGACT
GACCGTGATGAAAACAAAACTATTGCTCTTGGCACCTCCAAGTTGAACTATTTAGACCCTAGAATTTCGGTCG
CTTGGTG
CAAGAAATTTGATGTGCCCATTGAAAAAATATATAACAAAACTCAA------
CGGCTGCCGCCGTTCCGCGTCATCGGCG
ACCACCTGAAGGACCGCTTCGACGGCGCTTCGCGCGTCATGCTCAGCAACTCTGCCAGCTCCAGGGG------
------G
AACGC---GAACCGCC---
CRAAACAAGACAAGCTGTCGAACAGCATCGCCTCGAACAGCATACACAGCAAAAGGGAGAA
CAGGCCGCGCAAGTACAAGTACGGGTTCCAGCTGAAGCCGTACAACCCGGACCACAAGCCGCCCAGTCCCAAG
GACCTGG
TGTACCTGGAGCCGTCCCCCGGATTCTGCGAGAAGAACCCGAAGCTGGGCATCCAGGGTACCCACGGTAGGCT
G-TGCAA
CGACACCTCCATCGGTGTGGACGGGTGCGATCTGATGTGCTGCGGCAG-
GGGGTACAGGACCCAGGAGGTCATCGTGGTC
GAGAGGTGCAACTGCACG-------------
>Sciocharis
GGGAGAAGCCCAGCACTGAATCCCGTGGCCGAACCGGGAAATGTAGTGTTTGGGAGGGTCCGTTAGCCACCGT
ACGACGC
GTCCAAGTCCTTCTTGAACGGGGCCACATACCCATAGAGGGTGCCAGGCCCGGTAGCTGGCGGATCTCTCCTC
AGAGTCG
GGTTGCTTGAGAGTGCAGCCCTAAGTGGGTGGTAAACTCCATCTAAGGCTAAATATGACCACGAGACCGATAG
CGAACAA
GTACCGTGAGGGAAAGTTGAAAAGAACTTTGAAGAGAGAGTTCAATAGTACGTGAAACCGTTCAGGGGTAAAC
CTGAGAA
ACCCGAAAGGTCGAATGGGGAGATTCAGCGTGTCTCGTGTCTGGTTGCGTGACGGTGGTGCTCGCACCGGAAT
GCGCCTC
CCGGATCCGTAACCGGCGACGAACTCGTGCACTTCTCCCCTAGTAGGACGTCGCGACCCGTTGGGTGCCGGTC
TAAGGCC
GACGGTGGAGCCTTGGGGTCCCGGCCGGCCCGCTCGACGGTAAGACAGAGGCGTGGGGTCGCTACGTTAGCGT
CCGGCCC
GTCACAAGTTCGGGCGACTCGGATGTCGGACCTGTGCGCCGACCCCGAGCTCGCCGGCTGTTGGTGGCGGTGT
CCTCGGA
CAGACTACACGCCGGTCGGCGACGCTTTAGCTTTGGGTTTTCAGGACCCGTCTTGAAACACGGACCAAGGAGT
CTAGCAT

GTGCGCGAGTCATTGGGACCGCATCTAAACCTAAAGGCGAAATGAAAGTGAAGGCGTGCCGAGRGAGGATGGG
TCGGGGG
GCGTCTCGTTCTCATCGCGAGATGAGGCGCACCCAGAGCGTACACGCGCTTACACCGTATTCGCTGATTTGTT
CGA-TCC
CATTATTGAAGACTACCATGGTGGTTTCAAGAAGACCGACAAACACCCCCCTAAGAACTGGGGTGATGTCAAC
ACCTTCG
CTAATCTTGACCCTGCTGGTGAATACGTTGTCTCCACTCGTGTCCGTTGCGGACGTTCAATGGAAGGTTATCC
ATTCAAC
CCATGCTTAACCGAGGAACAATACAAAGAGATGGAAGGTAAAGTTTCCAGCACTTTGTCTGGCCTCGAAGGCG
AACTTAA
GGGTACTTTCTACCCATTGACTGGAATGGATAAAGACACCCAACAGAAACTCATCGATGATCACTTCTTGTTC
AAGGAAG
GTGATCGTTTCCTCCAGGCTGCTAATGCCTGCCGTTTCTGGCCAAGCGGACGTGGTATCTACCACAACGACAA
CAAAACA
TTCTTGGTCTGGTGCAACGAAGAAGATCATCTTCGTCTCATTTCCATGCAGATGGGTGGTGATCTTGGTGAAG
TATATCG
TCGTCTTGTTACTGCTGTCAACGATATTGAGAAGCGCGTTCCTTTCTCCCATAATGACAGATTAGGTTTTCTT
ACTTTCT
GCCCAACCAACTTGGGCACAACTGTACGTGCCTCTGTACACATTAAAGTACCTAAGCTCGCCGCCAACAAAGC
TAAGCTC
GATGAAATTGCTGCCAAATACAACTTGCAAGTACGTGGTACCCGTGGTATTCCTAAAAAAGTTTTGATTATTG
GCTCAGG
TGGTTTATCAATAGGACAAGCTGGAGAATTTGATTATTCTGGATCACAAGCAATAAAAGCTTTACAAGAAGAA
AATATTC
AAACTGTTCTAATCAATCCAAACATAGCCACTGTACAAACATCTAAAGGTTTAGCCGACAAAATTTACTTCCT
ACCTTTA
GTGCCTGAATTTGTAGAGCAAGTTATTAGAGCAGAACGCCCTGGTGGTGTTTTACTAACATTTGGTGGTCAAA
CAGGGTT
AAATTGTGGTGTAGAGTTACAGAGAGCCGGTGTTTTTGAAAAGTATGGTGTTCAAATTCTGGGTACACCTATA
CAAGCCA
TTATTGATACGGAAGACAGAAAAGTTTTCAGTGAAAGAATTGCACAAATTGGTGAAAAAGTTGCTCCAAGTAT
GGCTGCT
TATTCTGTGCAAGAAGCACTGGAAGCAGCAGAATTATTGGGATACCCTGTGATGGCAAGAGCTGCTTTTTCAT
TGGGAGG
GTTAGGTTCTGGGTTTGCAGATACAGCTGAAGAATTAAAATCTCTAGCGCAACAAGCTTTAGCACATTCCAAT
CAATTAA
TTATTGATAAGTCTTTGAAAGGTTGGAAAGAAGTTGAATATGAAGTTGTTAGAGATGCATATCCATATCTAAA
AACAGTA
AATGATGAGGAACTCCAAGAACCAACAGATAAAAGAATGTTTGTTTTGGCTGCTGCTTTAAGAAATGGATATA
GTGTTGA
AAAATTATATGAGTT-
AACAAAAAATTGATCATTGGTTTTTACAGAAAATGAAAAATATTGTGGATTATAATACTCTCTTA
GAATCAATTCAACAACATAAATTG---------------
ACACACAAACTTTTATTAAAAGCGAAACAAATTGGATTTAG
TGACAAACAAATAGCTGTTGCTGTTAAAAGCACCGAACTTGCTATAAGAAAGCAACGACAAGATTTTGAGATA
ACTCCAT
ATGTTAAACAAATTGATACCGTTGCTGCTGAATGGCCTGCAACAACAAATTATCTATATTTAACATATAATGC
TGAAACC
CATGATCTAACATTTTGCGATCAACATATAATAGTTATTGGATCAGGAGTTTATCGAATTGGAAGTTCAGTAG
AGTTTGA
TTGGTGTGCTGTAGGATGCTTAAGAGAACTCCGAAAATTAAACAAAAAAACAATAATGATAAATTACAATCCA
GAAACTG
TTAGTACAGATTACGATATGTCCGACCGATTGTATTTTGAAGAAATTTCATTCGAAGTAGTTATGGATGAAGT
TTATATT
TTAATTTTACCTGGATTTGGGTTAATTTCCCATATTATTAGACAAGCTAGAGGAAAAAAAGAAACTTTTGGAT
CTTTAGG

GATAATTTATGCTATACTTTCAATTGGTTTATTAGGATTTGTTGTTTGAGCCCACCATATATTTACTGTTGGA
ATAGATG
TGGATACCCGGGCTTATTTCACTTCAGCTACTATAATTATTGCGGTTCCAACAGGAATTAAAATTTTTAGATG
ATTAGCT
ACTTTACATGGCACCCAAATTAAATTTACCCCCCCTATGTTATGAGCTTTAGGGTTTGTTTTTTTATTTACAA
TTGGGGG
TTTGACAGGAATTATTCTTGCTAACTCTTCAATTGATATTATTTTACACGATACTTATTATGTTGTCGCCCAT
TTTCATT
ATGTTTTATCTATAGGGGCTGTTTTTGCAATTATAGGAGGATTAGTTCAATGATACTCTTTATTTACTGGTTT
AACTTTA
AATGAACACTTTTTAAAAATTCAATTTTTTATTATATTTATTGGAGTTAATGTAACATTTTTCCCTCAACATT
TTCTTGG
ATTAGCAGGAATACCTCGGCGATATTCAGATTACCCGGATGCTTATACTTCTTGAAATGTTATTTCTTCAATT
GGTAGAT
TAATTTCTATAGTTTCAATTTTTATTTTATTATTTATTATTTGAGAAAGATTCACTTCAATACGAATAGTTAT
TTCAGCT
ACTAATTTTGTCACATCAATTGAATGAATGCAAAAATACCCCCCTGCCGAACATAGATATTCAGAATTACCTA
TATTAAC
AAGAGATTACGTGGTGGTATTTGATTTTCTTGGT-
AAGGATTCCATTAGATATTACAATGAAGTACCTGTGGAAAAACGT
GTCTTCAAAAACCTTCAATTGTTTATGGAAAATAAATCTTCAGGAGATGATTTATTTGATAGGCTGAATACCG
CTGTGAT
GAACAAACATTTAAACGAGTTAATGGAAGGTCTAACTGCCAAGGTGTTTCGTACTTATAACGCTTCCTGGACT
TTACAGC
AGCAACTCGAAAAATTGACCAATCCCGATGATTCCATATCCGAAAAAATTTTATCTTACAACCGAGCTAATAG
AGCTGTA
GCTATACTTTGTAACCATCAACGTGCTGTACCGAAAGGCCACCAGAAATCCATGGAGAAGCTAAAAGAAAAGA
TAGAAGC
CAAAAGGGAGAATATCAAGGATGCCGAGAGACAAGTGAAAGACGCACAAAGGGATGCCAAGCA---
CGGAAGTGTTAAGG
AGTCCCAGATATACGATAAGAAAAGGAAAATGTTGGAGAGGCTAAAGGAACAGTTGGCAAAGTTGGAAATTCA
AGAGACG
GACCGTGACGAAAATAAAACTATTGCCCTCGGCACGTCCAAGTTGAACTATTTAGATCCTAGAATTTCAGTTG
CTTGGTG
TAAGAAGTTTGGTATACCCATCGAAAAGATTTATAACAAAACACAATGGATGCGGTTGCCACCCTTCAGAGTG
ATCGGCG
ATCATCTAAAGGACCGTTTCGATGGTGCCTCCCGAGTGATGCTCAGCAATTCGGCAAGTTCGAG---------
------G
AACCA---AAATCGAC---
CGAAGCAAGATAAACTGTCGAACAATATCGCGTCTAACAGCATTCATAGTAAAAGAGAGAA
CCGCCCGCGAAAATACAAATACGGTTTCCAACTGAAACCTTACAATCCTGATCATAAGCCTCCGAGTCCCAAG
GATTTAG
TATACTTGGAACCGTCACCTGGTTTCTGCGAGAAAAATCCAAAGCTTGGTATACAGGGTACTCACGGTAGATT
G-TGCAA
TGATACGTCTATAGGCGTTGATGGGTGCGACTTAATGTGTTGCGGTAG-
AGGCTACAGGACCCAGGAAGTTATTGTTGTT
GAAAGATGCAACTGCACTTTCC---------
>Scioporus
GGGAGAAGCCCAGCACTGAATCCCGTGGCCGAACCGGGAAATGTAGTGTTTGGGAGGATCCATTATCCATCGT
ACGACGC
GTCCAAGTCCTTCTTGAACGGGGCTAAATACCCATAGAGGGTGCCAGGCCCGGTAGCTGGTGGATCTCTCCTC
AGAGTCG
GGTTGCTTGAGAGTGCAGCCCTAAGTGGGTGGTAAACTCCATCTAAGGCTAAATATGACCACGAGACCGATAG
CGAACAA
GTACCGTGAGGGAAAGTTGAAAAGAACTTTGAAGAGAGAGTTCAATAGTACGTGAAACCGTTCAGGGGTAAAC
CTGAGAA

ACCCGAAAGGTCGAATGGGGAGATTCAGCGAGGCTCGTTTCTGGTTGCGTGACGATTGTGCTTGCACTTGGCT
GCGCCTT
CCGGATCCGCAATCGGCGACGGACTCGTGCACTTCTCCCCTAGTAGGACGTCGCGACCCGTTGGGCGCCGGTC
TAAGGCC
GACGGTGGAGACTTTGGGTCCCGGCCGGCACGCTCGACGGTAAGACAGAGACGTGGGGTCGCTACGTTAGCGT
CCGGCCC
GTCACAAGTTCGGGCGTCTCGGATGTCGGACCTGTGTGCCGACCTCGAGCGCGCCGGCTGTTGGTGGCGGTGT
CCTCGGA
CAGACTACACGTCGGTCGGCGACGCTTTAGCTTTGGGTTTTCAGGACCCGTCTTGAAACACGGACCAAGGAGT
CTAGCAT
GTGCGCGAGTCATTGGGACCGCATCTAAACCTAAAGGCAAAATGAAAGTGAAGGCGTGCCGAGGGAGGATGGG
TCGGGGG
GCGTCTCCTTCTCATCGCGAGATGAGGCGCACCCAGAGCGTACACGC-----------------
TGATTTGTTCGA-TCC
CATCATTGAAGACTACCATGGTGGATTCAAGAAGACCGATAAGCACCCTCCCGCAAACTGGGGTGATGTCAAC
ACCTTCG
CTAACCTCGACCCTGCTGGTGAATACGTAGTCTCCACCCGTGTTCGTTGCGGCCGCTCCATGGAAGGCTATCC
TTTCAAT
CCATGCTTAACCGAAGAACAATACAAGGAAATGGAACAAAAAGTTTCCAGCACTTTGTCCGGCATGGAAGGAG
AACTTAA
GGGTACTTTCTACCCATTGACTGGAATGGATAAGGCTACTCAACAGAAGCTTATTGATGATCATTTCTTATTC
AAGGAAG
GTGATCGTTTCCTTCAAACTGCTAACGCCTGTCGTTTCTGGCCATCTGGACGTGGTATCTACCACAATGATAA
TAAAACC
TTCTTGGTTTGGTGCAACGAAGAGGATCATCTTCGTATCATCTCTATGCAGATGGGTGGTGATCTTGGTGAAG
TCTACCG
TCGTCTTGTAAGCGCTGTCAACGAAATTGAAAAACGCGTACCATTCTCTCATAATGACAGATTAGGTTTCCTT
ACTTTCT
GCCCGACTAACTTGGGCACAACAGTACGTGCCTCTGTACACATTAAAGTACCTAAGCTCGCTGCCAATAAGGC
TAAACTC
G--
GTACCTAAAAAAGTACTAATAATTGGTTCAGG
AGGTTTATCTATAGGACAAGCTGGTGAATTTGATTATTCTGGCTCACAGGCGATAAAAGCGTTGCAAGAARAA
AACATTC
AAACGGTTTTAATCAATCCCAACATTGCTACTGTACAAACATCGAAAGGCTTGGCTGATAAAGTTTACTTTTT
ACCATTA
GTGCCTGAATTTGKTGAAGAAGTAATTAGAGTAGAACGCCCTGGAGGTGTTCTACTAACATTTGGTGGACAAA
CAGGGTT
AAATTGSGGSGTAGAGTTACAAAAAGCTGGTATTTTTGAAAAATATGGTGTCAAAATCCTAGGTACACCTATA
GAAGCCA
TAATTGATACAGAAGACAGGAAAATTTTTAGTGATAGAATTTCTTTAATTGGTGAAAAGGTCGCTCCAAGTAT
GGCTGCG
TATTCAGTACAAGAAGCACTTGAAGCTGCAGAATTGTTAGGCTACCCAGTAATGGCAAGAGCTGCATTTTCTT
TAGGTGG
TTTAGGGTCTGGTTTTGCTAATACCGATGAGGAACTGAAATCACTTGCTCAACAAGCTTTAGCTCACTCAAAT
CAATTGA
TTATTGATAAATCTTTGAAAGGATGGAAAGAAGTTGAGTATGAAGTTGTACGGGATGCATATCCATATTTAAA
AGATGTT
GATGATGAAGAATTGAAAGAACCTACAGATAAAAGAATGTTTGTACTGGCTGCTGCT----
GGAGTCSCTACMCAATCGA
TAAATTATATGATTTNAACTAAAATCGATCGTTGGTTCTTACAAAAAATGAAGAAGATTGTAGACTATAACAC
ATATTTG
GAATCAATTCAACAGAATAAATTA---------------
ACGTTTAAAATGTTACTAAAAGCGAAACAAATTGGTTTTAG
CGATAAACAAATTGCTGTAGCTGTTAAAAGCACTGAGCTTGCTATTAGAAAGCAACGCCAGGATTTCAATATA
ACTCCAT

ATGTTAAACAGATTGATACAGTTGCTGCTGAATGGCCGGCTACCACAAATTACCTCTATCTAACGTACAATGC
AGAAAGC
CATGATATATCTTTTAATGACCAACATATAATAGTTATTGGTTCCGGTGTTTACAGAATTGGAAGTTCAGTTG
AATTCGA
TTGGTGTGCTGTTGGGTGCCTAAGGGAACTTCGAAAATTAAATAAAAAAACTATAATGATTAATTATAATCCT
GAGACTG
TTAGTACAGACTATGATATGTCAGATAGACTATATTTTGAAGAAATATCGTTTGAAGTAGTTATGGAT-----
----
----TTTTACCTGGATT--
GGATAATTTCTCATATTATTAGTCAATCTAGAGGAAAAAAAGAAACATTTGKAAYTTTARG
AATAATCTATGCTATAATAGCTATTGGATTATTAGGATTTGTTGTTTGAGCTCATCATATATTTACAGTMGGA
ATARATG
TTGATACACGAGCTTATTTAAAAGTCCAACAAMYATTATTGCTGTTCCTCCMKGAATTAAAATTTT------
----

TCTTTGATTTTCTTGGTAAAGGATTCCATTAGATACTACAATGAAGTACCTGTAGAAAAACGT
GTCTTCAAAAATCTTCAATTATTCATGGAAAATAAATCGCCAGGAGATGATTTGTTTGATAGATTAAATACAG
CTGTGAT
GAACAAACATTTAAATGAACTAATGGAAGGTTTAACTGCCAAGGTGTTTCGTACTTACAATGCTTCTTGGACT
CTACAAC
AACAACTCGATAAATTGACCAATCCAGATGATTCCATATCCGAAAAAATATT---------------------
----

------G
ACCATTTAAAAGACCGCTTTGACGGTGCATCGAGGGTAATGTTAAGCAACTCAGCTAGTTCCCGTGG------
------A
AATGC---AAACCGCC---
CTAAACAAGACAAACTATCAAACAACATCGCTTCAAACAGCATACACAGTAAAAGAGAAAA
CCGCCCTCGCAAATACAAATATGGGTTCCAATTAAAACCATATAATCCAGACCACAAACCTCCAAGTCCTAAA
GATTTAG
TATACTTGGAGCCATCACCTGGTTTCTGCGAGAAGAACCCGAAGCTTGGGATCCAAGGTACACATGGTAGGTT
A-TGTAA
TGATACTTCAATTGGTATTGATGGATGCGATTTGATGTGCTGCGGAAG-
GGGATACAGGACCCAGGAAGTTATTGTAGTC
GAA----------------------------

>Scopaeus

AAGCCCAGCACTGAATCCCGTGGCCGAACCGGGAAATGTAGTGTTTGGGAGGGTCCGTTATCCATCGTGCGAC
GC
GTCCAAGTCCTTCTTGAACGGGGCCATATACCCATAGAGGGTGCCAGGCCCGATAGCTGGTGGATCCCTCCTC
AGAGTCG
GGTTGCTTGAGAGTGCAGCCCTAAGTGGGTGGTAAACTCCATCTAAGGCTAAATATGACCACGAGACCGATAG
CGAACAA
GTACCGTGAGGGAAAGTTGAAAAGAACTTTGAAGAGAGAGTTCAATAGTACGTGAAACCGTTCAGGGGTAAAC
CTGAGAA
ACCCGAAAGTTCGAATGGGGAGATTCAGCGTGTATCGTGTTTGGTCGCGTGACGATGGTGCTTGCACCTGGCT
GCGCCTT
CTGAGCCCAATTCTTACTGCGAGCGCGTGCACTTCTCCCCTAGTAGGACGTCGCGACCCGTTGGGCGCCGGTC
TACGGCC
GATGGAGGAGACTTGAGGTCCCGGCCGGCCCGCTCGACGGTAGGACAGAGACGTGGGGTCGCGACGTTCGCGT
CCGGCCC
GTCACAAGTATGGGCGACCTGGACGCCGGACCTATGTGCCGGCCCCGGGCCCGACTGCTGCTGGTGGCGGTGT
CCTCGGA
CAGACTGCACGTCGGTCGGCGACGCTTTAGCTTTGGGTTTTCAGGACCCGTCTTGAAACACGGACCAAGGAGT
CTAGCAT
GTGCGCGAGTCATTGGGACTTGA--
TAAACCTAAAGGCGAAATGAAAGTGAAGACCTGTCGAGGGAGGATGGGTCGGGGG
GCGTCTCATTCTCATCACGAG---------------------------
CTTACACAACATTCGCCGACTTGTTCGA-CCC
CATCATCGAAGACTACCATGGTGGTTTCAAGAAGACCGACAAACACCCACCTGCTAACTGGGGTGATGTCAGC
ACTTTCG
GCAATTTGGACCCAGCTGGTGAATACGTCGTCTCCACCCGTGTCCGTTGCGGCCGTTCAATGGAAGGCTATCC
ATTCAAC
CCATGTTTAACTGAAGACCAATACAAGGAGATGGAACAAAAAGTTTCAGCCACTTTGTCTGGTTTGGAAGGTG
AATTGAA
GGGTACTTTCTACCCATTGACTGGAATGAGCAAGGATGTTCAACAGAAACTCATCGACGATCATTTCTTGTTC
AAGGAAG
GTGATCGCTTCTTGCAAACTGCTAACGCTTGCCGTTACTGGCCAAGCGGACGTGGTATCTACCACAACGACAA
CAAGACT
TTCTTGGTCTGGTGCAACGAAGAAGATCACCTTCGTATCATCTCCATGCAAATGGGTGGTGATTTAGGTGAAG
TATACCG
TCGCCTTGTCACCGCTGTCAACGAAATCGAGAAGCGCGTCCCATTCTCTCATAACGACAGATTAGGTTTCCTT
ACCTTCT
GCCCAACCAACTTGGGTACAACTGTCCGTGCCTCTGTACACATCAAGGTGCCCAAACTCGCCGCTAACAAGGC
TAAACTC

GTTCCTAAAAAAGTATTAATCATTGGTTCAGG
TGGCTTATCAATCGGTCAAGCTGGAGAGTTTGATTATTCTGGATCTCAAGCTATTAAAGCTTTACAAGAAGAG
AACATAC
AAACAGTGTTGATCAATCCCAATATAGCTACTGTACAAACATCTAAAGGACTTGCTGATAAAGTATATTTTTT
ACCGTTA
GTGCCCGAATTTGTTGAACAAGTAATTAGAGTTGAACGTCCAGGAGGGGTTTTATTAACCTTTGGGGGTCAAA
CAGGTTT
AAATTGTGGAGTTGAACTTCAAAAAGCTGGTATTTTTGAAAAATATGGTGTTAAAATATTGGGTACTCCCATA
CAAGCTA
TCATAGACACGGAAGATCGTAAAGTTTTTAGCGACAGAATATCGTTAATTGGTGAGAAAGTAGCACCAAGTAT
GGCTGCT
TATTCTGTGCAAGAAGCTTTAGAAGCAGCCGAGTTGTTAGGTTACCCTGTTATGGCTAGGGCAGCTTTCTCGT
TGGGTGG
TTTAGGATCTGGTTTTGCTACACAGCTNNATGAACTAAAATCACTTGCTCAACAAGC-----------------
----

CCTTATCTTAAAGAAGTA
AATGATGAAGAATTACAAGAACCAACAGACAAACGTATGTTTGTACTTGCTGCTGCTTTAAGAAATGGTTATT
CTGTGGA
TAAATTATACAATTT-
AACAAAAAATTGATCGTTGGTTCTTGCAAAAAATGAAAAACATTGTTGATTTTAATACGTACTTG
GAAACCGTT---CAGAACAAACTT--------------
ACTTTTAAAGATTTGTTGAAAGCTAAGCAAATTGGTTTTAG
CGATAAACAAATTGCCGTTGCTGTGAAAAGTACCGAACTAGCAGTTAGAAAACGTAGAAAAGATTTCGATATA
ACACCAT
ATGTAAAACAAATAGACACTGTTGCTGCTGAATGGCCTGCTACTACCAATTATTTATATTTAACTTACAATGC
TGAAAGT
CATGATTTAACCTTTAACGATGAACATATTATGGTTATTGGATCTGGTGTTTATAGAATTGGAAGTTCTGTGG
AGTTTGA
TTGGTGTGCTGTTGGTTGTTTGCGAGAACTTAGAAAACTAAATAAAAAAACTATTATGGTCAACTATAATCCC
GAAACTG
TTAGTACAGATTATGATATGTCTGATAGACTATACTTTGAAGAAATATCATTTGAAGTCGTAATGGATGAAGT
ATACATT
TTAATCCTCCCAGGGTTTGGAATAATTTCCCACATTATTAGACAAGCTAGAGGCAAAAAAGAAACTTTTGGAT
GTCTGGG
AATAATTTATGCAATAATAGCAATCGGATTATTGGGATTTATTGTATGAGCTCACCATATATTTACAGTTGGC
ATAGATG
TTGACACTCGGGCCTATTTTACATCAGCTACTATAATCATTGCTGTACCTACAGGTATCAAAATTTTTAGATG
GTTAGCA
ACCCTTCATGGAAGACAAATTAATTTTAATCCTCCTATATTATGATCCCTAGGGTTTGTCTTTTTATTCACTA
TTGGGGG
GTTGACAGGAGTTATCTTAGCCAATTCATCAATTGATATTATTCTTCATGATACTTACTACGTAGTAGCCCAC
TTTCACT
ATGTACTTTCAATAGGAGCTGTATTTGCAATCATAGCTGGATTAGTCCAATGATTCCCTTTATTCACTGGATT
AACCATA
AATGAGTACCTTTTAAAGATTCAATTTTTCATTATATTTATTGGGGTAAATCTAACATTTTTCCCCCAACATT
TTTTAGG
ACTAGCAGGTATACCTCGACGATACTCTGACTACCCAGATGTTTATACTCCGTGAAATGTAATTTCATCAATC
GGCTCCT
TAGTCTCTATAGTTAGAATTTTTTTATTAATTTTTATCATTTGAGAAAGATTCTCATCTCTTCGATTAGTGGT
ATCCTCA
AAAAACTTCTGCACATCCATTGAATGATTCCAATTTTATCCCCCCTCTGAACACTCTTATTCTGAATTACCAA
TAATTAT
TAAAGACTATGTTGTTGTATTTGATTTCCTCGGA-
AAGGATTCCATTAGATATTACAATGAAGTACCTGTAGAGAAACGT
GTTTTCAAAAACCTTCAATTGTTCATGGAAAACAAATCTCCAGGTGATGATTTATTCGATCGATTAAACACAG
CTGTGAT
GAACAAACACTTAAATGAATTGATGGAAGGGTTAACGGCCAAAGTGTTCCGTACTTATAACGCCTCCTGGACT
TTACAGC
AACAACTTGAAAAACTCACAAATCCCGATGATTCCATATCCGAAAAAATCTTATCATACAACCGAGCCAATCG
TGCCGTT
GCTATTTTATGTAACCATCAACGTGCAGTCCCTAAAGGTCACCAAAAATCCATGGAAAAACTTAAAGAAAAAA
TCGATGC
CAAACGAGACGCTATACGTGATGCCGAACGTATGGTTAAAGATGCCCAGAGAGATGCTAAACA---
TGGAAGTGTGAAAG
AAAAACAAATCTACGATAAAAAGAAGAAAATGTTGGAAAGATTGCGTGAACAACTCGCTAAATTAGAGATACA
AGAAACT
GATCGCGATGAAAATAAAACTATTGCTCTTGGTACGTCCAAGTTGAATTATTTAGATCCTAGGATCTCTGTCG
CTTGGTG
TAAGAAGTTCGGTGTGCCCATCGAAAAGATTTACAATAAAACTCAA------
CGTCTACCACCGTTCCGTGTCATCGGCG

ATCACCTGAAGGACCGTTTCGACGGCGCATCCCGGGTGATGCTTTCRAATTCCGCCAGTTCCAACCG------
------C
AACGC---CAATCGTC---
CGAAACAAGATAAACTATCAAACAGCATAGCTTCCAACAGCATCCACAGCAAACGCGAAAA
TCGACCGAGAAAATATAAATACGGTTTYCAATTAAAACCGTATAATCCTGATCATAAACCGCCGAGTCCTAAA
GATTTAG
TTTACTTGGAACCGAGTCCCGGTTTTTGTGAGAAAAATCCCAAGTTGGGGATACAGGGTACGCATGGTAGACA
G-TGTAA
TGATACTTCTATTGGAGTCGACGGGTGCGATTTGATGTGTTGCGGGAG-
AGGTTACAGAACACAGGAGGTGGTTGTTGTT
GAACGGTGCAATTGCACG-------------
>Stilicoderus
GGGAGAAGCCCAGCACTGAATCCCGTGGCCGAATCGGGAAATGTAGTGTTTGGGAGGGTCCGTTAACCATCGT
GCGACGC
GTCCAAGTCCTTCTTGAACGGGGCCACATACCCATAGAGGGTGCCAGGCCCGATAGCTGGTGGATCTCTCCTC
AGAGTCG
GGTTGCTTGAGAGTGCAGCCCTAAGTGGGTGGTAAACTCCATCTAAGGCTAAATATGACCACGAGACCGATAG
CGAACAA
GTACCGTGAGGGAAAGTTGAAAAGAACTTTGAAGAGAGAGTTCAATAGTACGTGAAACCGTTCAGGGGTAAAC
CTGAGAA
ACCCGAAAGGTCGAATGGGGAGATTCAGCGTGTCTCGTTTCTGGTCGCGTGACGATGGTGCTTGCACCGGGCT
GCGCCTT
CCGGATCCGTATCCGGCGACGAACTCGTGCACTTCTCCCCTAGTAGGACGTCGCGACCCGTTGGGCGCCGGTC
TAAGGCC
GACGGTGGAGCCTTGGGGTCCCGGCCGGCCCGCTCGACGGTAAGACAGAGGCGTGGGGTCGCTACGTTAGCGT
CCGGCCC
GTCACAAGTTCGGGCGACTCGGATGTCGGACCTGTGTGCCGACCTCGAGCTCGCCGGCTGTTGGTGACGGTGT
CCTCGGA
CAGACTACACGCCGGTCGGCGACGCTCTAGCTTTGGGTTTTCAGGACCCGTCTTGAAACACGGACCAAGGAGT
CTAGCAT
GTGCGCGAGTCATTGGGACCGCATCTAAACCTAAAGGCTAAATGAAAGTGAAGGCGTGCCGAGGGAGGATGGG
TCGGGGG
GCGTCTCGTTCTCATCGCGAGTTGAGGCGCACCCAGAGCGTACACGCGCTTACACCGTATTCGCTGATTTGTT
CGA-TCC
CATCATTGAAGACTACCATACCGGCTTCAAGAAGAGCGACAAGCACCCGCCCAAGAATTGGGGAGACGTAAAT
ACTTTCG
CCAATCTTGATCCTGCAGGTGAATATGTGGTATCCACCCGTGTTCGTTGCGGTCGCTCCATGGAAGGCTACCC
CTTCAAC
CCGTGCTTAACCGAAGAGCAATACAAGGAGATGGAAGGCAAAGTCTCCGGCACTTTGTCCGGTCTCGAAGCCG
AACTCAA
GGGTACATTCTACCCGTTGACCGGAATGGACAAGGATACTCAACAGAAGCTCATCGACGATCACTTCTTGTTC
AAGGAAG
GTGACCGTTTCCTCCAGGCTGCCAACGCCTGTCGCTTCTGGCCATCCGGACGTGGTATCTACCACAACGACAA
CAAGACC
TTCTTGGTTTGGTGCAACGAGGAGGATCATCTTCGCATCATCTCGATGCAGATGGGCGGTGATCTTGGCGAAG
TCTATCG
CCGCCTCGTTACCGCCGTTAACGAAATCGAGAAGCGTGTACCGTTCTCGCATAATGACAGATTAGGTTTCCTC
ACTTTCT
GCCCAACAAACTTGGGTACAACTGTACGTGCCTCTGTACACATCAAAGTACCTAAACTCGCCGCCAACAAAGC
CAAGCTG
GATGAAGTCGCTGCCAAATACAACTTACAAGTACGTGGTACCCGC---
GTACCTAAAAAAGTTTTAATAATCGGATCAGG
AGGGTTGTCCATCGGACAAGCTGGAGAGTTTGATTATTCTGGCTCACAAGCTATCAAAGCTTTGCAAGAAGAA
AACATTC
AAACAGTGCTAATTAATCCAAACATTGCAACTGTACAAACATCTAAAGGTTTAGCAGATAAAGTTTATTTTTT
GCCTTTA

GTGCCTGAATTTGTAGAACAAGTGATTCGAGTGGAGCGTCCTGGAGGTGTTTTATTAACATTCGGCGGACAAA
CTGGGTT
AAATTGCGGTGTGGAATTACAAAAAGCTGGTATCTTTGATAAATACGGCGTTAAAATTTTGGGTACACCAATA
GAAGCCA
TTATAGATACTGAAGACAGAAAGATTTTTAGCGAAAGAATAGCATTAATTGGTGAAAAAGTTGCTCCAAGTAT
GGCTGCT
TATTCAGTACAAGAAGCTTTAGAAGCTGCCGAGTTATTAGGGTATCCAGTTATGGCAAGAGCTGCTTTCTCTT
TAGGCGG
ATTAGGGTCAGGGTTTGCTAATACAGCTGAAGAACTGAAATTACTTGCTCAACAAGCTTTAGCTCATTCCACT
CAGTTAA
TTATTGATAAGTCTTTAAAAGGGTGGAAAGAAGTTGAATATGAAGTTGTTAGAGATGCATATCCTTATCTAAA
GGATGTT
AATGATGATGAATTACAAGAACCTACAGATAAAAGAATGTTCGTCTTAGCAGCGGCTTTAAGAAATGGTTATA
GTGTAGA
TAAATTATATGATTT-
AACAAAAAATTGATCGTTGGTTCTTACAAAAAATGAAGAAAATCGTAGATTTTAACACTATTCTC
GAAAACATTCAACAAAATAAATTACAAAATTGCTCAAATACGTATAGGCTTTTATTAAAAGCGAAACAAATTG
GATTTAG
TGATAAACAAATTGCTGTTGCTATTAAAAGCACTGAACTTGCAGTTCGAAAGCAACGACAGGATTTCGGCATC
ACTCCAT
ACGTGAAACAAATTGATACTGTCGCTGCTGAATGGCCAGCAACTACAAATTATCTATATCTAACATACAATGC
CGAAGAT
CACGATTTAACTTTCTCAGAACAACACATAATAGTTATAGGATCTGGAGTTTATAGAATTGGAAGTTCTGTGG
AGTTTGA
TTGGTGTGCTGTAGGGTGTTTGAGAGAATTAAGGAAATTAAATAAAAAGACAATAATGGTTAATTATAATCCT
GAAACTG
TTAGTACAGATTATGATATGTCGGACAGGTTGTACTTCGAGGAAATTTCATTTGAAGTTGTAATGGAT-----
----
CTAATTTTACCAGGATTCGGATTAATTTCTCATATTATTAGTCAATCTAGAGGAAAAAAAGAAACTTTTGGGG
CCTTAGG
AATAATTTATGCTATAATAGCAATTGGCCTCCTAGGATTTGTTGTATGAGCACATCACATATTTACTGTAGGT
ATAGATG
TAGATACACGAGCTTATTTTACTTCTGCTACTATAATTATTGCTGTTCCTACAGGAATTAAAATTTTTAGATG
ATTAGCT
ACTTTACATGGAACACAAATTAAATTTTCTCCTCCAATATTATGAGCCTTAGGATTTGTATTTTTATTCACAA
TTGGAGG
ACTTACAGGAGTAATTTTAGCTAATTCTTCTATTGATATTATTCTTCATGATACTTATTATGTAGTAGCTCAT
TTCCATT
ATGTTCTTTCTATAGGAGCTGTTTTTGCTATTATAGCAGGATTAGTCCAATGATTCCCCCTATTTACTGGATT
AACTTTA
AATGAATATCTTCTAAAAATTCAATTTATTATTATATTTACAGGAGTTAATTTAACATTTTTCCCTCAACATT
TTCTAGG
TCTAGCTGGTATACCTCGTCGTTATTCCGATTACCCTGATGCTTATATACCATGAAATGTAATTTCTTCTATT
GGATCTC
TTATTTCGATAATTAGAATTTTCATTCTATTATTTATTATCTGAGAAAGATTCTCTTCATTTCGAATAAATAT
TTCAGCA
AAAAATTTTTCCACGTCAATTGAATGATTCCAACTTTATCCTCCAGCTGAACAT-------------------
----

TGGATGCGGCTGCCCCCGTTCCGCGTGATCGGCG
ACCACCTGAAGGACCGGTTCGACGGGGCGTCGCGCGTCATGCTCAGCAACTCGGCGAGCTCCAGGGG------
------T
AGCGC---GAACCGCC---
CCAAACAGGACAAGCTGTCGAACAGCATCGCCTCGAACAGCATACACAGCAAGCGGGAGAA
CAGGCCGCGCAAGTACAAGTACGGTTTCCAGCTGAAGCCGTACAATCCGGACCACAAGCCGCCCAGTCCCAAG
GACCTGG
TGTACCTGGAACCGTCGCCGGGGTTCTGCGAGAAGAACCCCAAGYTGGGCATACAGGGCACGCACGGCAGGCT
G-TGCAA
CGACACATCGATCGGGGTGGACGGGTGCGACCTGATGTGCTGCGGCAG-
GGGGTACAGGACCCAGGAGGTCGTCGTCGTC
GAGAGGTGCAACTGCACGTTCCACTGGTGCT
>Stilicopsis
GGGAGAAGCCCAGCACTGAATCCCGTGGCCGAACCGGGAAATGTAGTGTTTGGGAGGGTCCGCTATCCATTGT
ACGGTGC
GTCCAAGTCCTTCTTGAACGGGGCCACATACCCATAGAGGGTGCCAGGCCCGATAGCTGGAGGATCTCTCCTC
AGAGTCG
GGTTGCTTGAGAGTGCAGCCCTAAGTGGGTGGTAAACTCCATCTAAGGCTAAATATGACCACGAGACCGATAG
CGAACAA
GTACCGTGAGGGAAAGTTGAAAAGAACTTTGAAGAGAGAGTTCAATAGTACGTGAAACCGTTCAGGGGTAAAC
CTGAGAA
ACCCGAAAGGTCGAATGGGGAGATTCAGCGTGTCTCGTTTCTGGTCGCGTGACGATGATGCTTGCATCGGACT
GCGCCTT
CCGGATCCGTAACCGGCGACGAACTCGTGCACTTCTCCCCTAGTAGGACGTCGCGACCCGTTGGGTGCCGGTC
TAAGGCC
GATGGTGGAGCCTTAAAG-
CCCGACCGGCCCGCTCGACGGTAAGACAGAGACGTGGGGTCGCTACGTTAGCGTCCGGCCC
GTTACAAGTTCGGGCGACTCGGATGTCGGACGTGTGTGCCGACCCCGAGCTCGCCGGCTGTTGGTGACGGTGT
CCTCGGA
CAGACTACACGTCGGTCGGCGACGCTATAGCTTTGGGTTTTCAGGACCCGTCTTGAAACACGGACCAAGGAGT
CTAGCAT
GTGCGCGAGTCATTGGGACCGCATCTAAACCTAAAGGCGAAATGAAAGTGAAGGCGTGCCGAGGGAGGATGGG
TCGAGGG
GCGTCTCGTTCTCATCGCGAGATGAGGCGCACCCAGAGCGTACACGCTCTTACACCGTATTCGCTGATCTGTT
CGA-TCC
CATCATCGAGGATTACCATGGTGGATTCAAGAAGACCGACAAGCATCCACCAGCAAACTGGGGTGATGTAAAT
ACTTTCG
CTAACTTGGACCCGGCTGGTGAATACGTCGTCTCCACTCGTGTCCGTTGCGGACGCTCAATGGAAGGATACCC
GTTCAAT
CCTTGCTTAACCGAAGAGCAATACAAAGAAATGGAAGGAAAAGTATCAACCACCTTGTCTGGTCTTGAAGGTG
AACTCAA
GGGTACTTTCTATCCTTTGACTGGAATGGACAAAACTACTCAACAGAAGTTGATTGATGATCACTTCTTGTTC
AAGGAAG
GAGATCGTTTCCTTCAAACCGCGAATGCTTGTCGTTACTGGCCTTCCGGCCGTGGTATTTACCACAATGATAA
CAAGACC
TTCTTGGTATGGTGTAACGAAGAAGATCATCTTCGCATTATTTCGATGCAGATGGGTGGTGATCTTGGCGAAG
TGTACCG

TCGTCTTGTAACAGCTGTTAATGAGATCGAAAAGCGCGTTCCATTCTCTCATAACGATAGATTAGGATTCCTC
ACCTTCT
GCCCTACCAACTTGGGT--

GTACCYAGAAAAGTGTTAATCATTGGTTCAGG
AGGTCTTTCTATCGGACAAGCTGGTGAATTCGATTATTCCGGTTCGCAAGCGATCAAAGCTTTACAAGAAGAA
AACATCC
AAACGGTTTTAATTAATCCAAACATCGCAACTGTACAAACATCGAAAGGTTTATCCGATAAAATATATTTTCT
TCCTCTC
GTGCCCGAGTTTGTCGAGCAAGTTATTCGAGCAGAACGCCCCGGTGGTGTTTTGTTAACATTCGGAGGACAAA
CTGGGTT
AAATTGCGGTGTAGAATTACAAAAAGCTGGAATTTTTGATAAATACGATGTTAAAATACTCGGTACACCTATA
CAAGCTA
TAATCGATACGGAAGACAGGAAAATATTTAGCGAAAGAATTGCTTCTATAGGTGAAAAAGTTGCTCCGAGTAT
GGCAGCT
TATTCTGTGCAAGAAGCATTAGAAGCTGCGGAATTATTAGGATATCCTGTGATGGCTAGGGCGGCATTTTCTT
TAGGAGG
TTTAGGATCTGGTTTTGCAAAYAGTAGCGATGAACTCAAATTACTTGCTCAACAAGCTTTAGCTCACTCAAAT
CAATTAA
TAATAGATAAATCGTTGAAAGGATGGAAAGAAGTTGAGTATGAGGTTGTAAGAGATGATTAT-----------
----GTT
AATGATGAAGAATTACAAGAACCTACTGATAAAAGAATGTTTGTGCTTGCTGCTGCTTTAAGAAACGGTTATA
GTGTTGA
TAAACTTTATGATTT-
AACCAAAATAGATCGTTGGTTTTTACAGAAAATGAAGAACATCATTGATTATAACAATGTTCTT
GAATCGTATCCTCAGAATAAATTTCAAG---
GCACTAATATATATATAAAGTTTTACTAAAAGCTAAACAAATCGGGTTTAG
CGATAAACAAATAGCTGTAGCTATTAAAAGTACAGAATTAGCTGTCAGAAAACAAAGACAAGATTTTGGTATT
ATTCCAT
ATGTTAAACAAATAGATACTGTAGCTGCTGAATGGCCTGCGACAACGAATTATTTGTATTTAACGTATAATGC
AAATAGC
CATGATATAACTTTTAGTGATCAACACATAATGGTTATTGGATCAGGAGTTTATAGAATCGGAAGTTCTGTTG
AGTTTGA
TTGGTGTGCTGTTGGATGTTTGAGGGAATTAAGGAAATTAAA---------------------------------
----

TTCTCATATTATTAGCCAGTCTAGAGGTAAAAATGAAACTTTTGGTACTTTAGG
AATAATTTATGCAATAATAGCTATTGGATTATTGGGATTTGTAGTTTGAGCTCATCATATATTTACTGTTGGA
ATAGATG
TTGATACTCGGGCTTATTTTACTTCTGCCACTATAATTATTGCAATTCCTACAGGAATTAAAATTTTTAGATG
ATTAGCA
ACTTTACATGGTACTCAAATAAAATTTACTCCTTCAATGTTATGGGCTTTAGGATTTGTTTTTTTATTTACTA
TTGGTGG
ATTAACAGGAGTAATTTTAGCTAATTCTTCTATTGATATTATTTTACATGATACTTATTATGTAGTTGCTCAT
TTTCATT
ATGTATTATCGATAGGTGCAGTTTTTGCTATTATAGCAGGATTTGTTCAATGATTTCCATTATTTACGGGTTT
AACAATA
AATGAAACTATACTAAAAATTCAATTTTTTATTATATTTATAGGAGTAAATTTAACTTTTTTCCCTCAACATT
TTTTAGG
ATTAGCAGGTATACCTCGACGTTATTCTGATTATCCTGATATTTATACCCCATGAAATGTAATTTCTTCAATT
GGAAGAT
TAATTTCTATAGTTAGAATTTTTTTATTACTATTTATTGTATGAGATAGATTTACAAGAATACGTATAAATAT
TTCATCT

TTAAACTTTAGAACATCCTTAGAATGATATCAATTATTTCCACCCGCTGAACATAG-----------------
----
-AAGGAATATGTAGTAGTATTTGATTTTCTTGGT-
AAGGATTCGATCAGATATTATAATGAAGTACCTGTCGAAAAACGT
GTCTTCAAAAATCTCCAATTGTTTTTGGAAAACAAAGAGCCAGGAGACGATTTATTTGATAGATTAAATACAG
CTGTGAT
GAACAAACATTTAAACGAACTAATGGAAGGCTTAACCGCTAAGGTATTTCGTACTTATAACGCTTCTTGGACC
CTACAAC
AACAACTTGACAAATTGACCAACGCAGACGATTCCATATCTGAAAAAATACTTTCATATAACCGAGCCAATAG
GGCAGTA
GCTATACTCTGTAACCATCAACGTGCTGTACCAAAAGGTCACCAAAAATCAATGGAAAAACTCAAAGAAAAAA
TCGACAC
TAAAAAAGATAATATCAAAGATGCCGAGAGGCAAGTTAAGGATGCACAGAGAGATGCGAAACA---
CGGAAGTGTTAAAG
AGAAACAGATCTATGAAAAGAAAAAGAAAATGTTGGAGAGGCTAAGAGAGCAGCTAGCTAAATTGGAAATTCA
GGAGACC
GACAGAGATGAAAATAAAACAATTGCACTCGGTACGTCAAAGTTGAACTATTTAGATCCGAGAATTTCGGTCG
CTTGGTG
TAAGAAATTTGGTGTGCCGATTGAAAAAATTTATAACAAAACTCAA---------------------------
----GAG
ATCACTTAAAGGACCGTTTCGATGGTGCATCAAGGGTGATGTTGAGTAATTCGGCGAGTTCTAGGGG------
------A
AACGC---GAATCGTC---
CAAAACAAGATAAATTATCAAACAGTATAGCGTCTAATAGTATCCATAGTAAGAGAGAAAA
TCGGCCGAGAAAATATAAATACGGTTTTCAATTGAAACCCTATAATCCGGATCATAAACCTCCGAGCCCTAAA
GATTTGG
TATATTTAGAACCATCGCCAGGTTTCTGCGAGAAGAATCCTAAACTAGGTATTCAAGGTACTCACGGTAGATT
G-TGCAA
T---

>Suniotrichus
GGGAAAAGCCCAGCACTGAATCCCGTGTCCGAACCGGGAAATGTAGTGTTTGGGAGGGTCCGCTATCCATCGT
ACGACGC
GTCCAAGTCCTTCTTGAACGGGGCCACATACCCATAGAGGGTGCCAGGCCCGATAGCTGGAGGATCTCTCCTC
AGAGTCG
GGTTGCTTGAGAGTGCAGCCCTAAGTGGGTGGTAAACTCCATCTAAGGCTAAATATGACCACGAGACCGATAG
CGAACAA
GTACCGTGAGGGAAAGTTGAAAAGAACTTTGAAGAGAGAGTTCAATAGTACGTGAAACCGTTCAGGGGTAAAC
CTGAGAA
ACCCGAAAGGTCGAATGGGGAGATTCAGCGTGTCTCGTGTTCTGGTCTGTGACGATGGTGTTCGCACCGGGCT
GCGCCTT
CCGGATCCGAAACCGGCGATGAACTCGTGCACTTCTCCCCTAGTAGGACGTCGCGACCCGTTGGGTGCCGGTC
TAAGGCC
GACGGTGGAGCCTTGAAGTCCCGGCCGGCCCGCTCGACGGTAAGACAGAGGCGTGGGGTCGCTACGTTAGCGT
CCGGCCC
GTCACAAGTTCGTTCGACTCGGATGTTGGACCTGTGTGCCGACCTCGAGCTCGCCGGCTGTTGGTGACGGTGT
CCTCGGA
CAGGCTACACGCCGGTCGGCGACGCTCTAGCTTTGGGTTTTCAGGACCCGTCTTGAAACACGGACCAAGGAGT
CTAGCAT
GTGCGCGAGTCATTGGGACCGCATCTAAACCTAAAGGCGAAATGAAAGTGAAGGCGTGCCGAGGGAGGATGGG
TCGGGGG
GCGTCTCGTTCTCATCGCGAGATGAGGCGCACCCTAGGCGTACACGC--------------------------
----

TACCATGGTGGATTCAAGAAGACCGACAAGCACCCCCCTAAGAACTGGGGTGACGTAAACGTCTTCG

CCAATCTCGACCCTGCCGGTGAATACGTTGTATCCACCCGCGTCCGCTGCGGCCGCTCCATGGAGGGTTACCC
ATTCAAC
CCCTGCTTAACCGAAGAGCAATACAAGGAGATGGAGTCGAAAGTGTCCAGCACCTTGTCCGGTCTCGAAGGCG
AACTCAA
GGGTACTTTCTACCCGTTGACCGGCATGGATAAGGATACTCAGCAGAAGCTCATCGACGACCACTTCTTGTTC
AAGGAGG
GCGATCGCTTCCTCCAGGCTGCCAACGCCTGCCGCTTCTGGCCGTCTGGACGTGGCATCTACCACAACGACAA
CAAAACA
TTCTTGGTCTGGTGCAACGAGGAGGACCATCTCCGTCTCATCTCCATGCAAATGGGTGGCGATCTTGGCGAAG
TCTACCG
TCGTCTCGTGAACGCCGTCAACGACATCGAAAAGCGCGTTCCCTTCTCTCATAACGACAGATTAGGTTTCCTC
ACTTTCT
GCCCATCCAACTTGGGCACAACTGTACGTGCCTCTGTACACATCAAAGTACCAAAGCTCGCATCCAACAAGGC
CAAGCTC
GACGAGGTCGCTGCTAAATACAACTTGCAAGTACGTGGCACCCGCGGTGTTCCTACAAAAGTTTTAATTATTG
GATCAGG
GGGATTATCAATTGGACAAGCCGGTGAATTCGATTACTCCGGTTCGCAAGCTATCAAAGCGTTACAAGAAGAA
AATATTC
AAACGGTGCTTATTAACCCCAACATTGCAACGGTACAAACATCGAAAGGTTTAGCTGATAAAATCTATTTTTT
ACCTTTA
GTTCCTGAATTTGTGGAACAAGTAATACGTGTTGAACGTCCCGGAGGTGTTTTATTAACATTTGGAGGTCAGA
CGGGTTT
AAATTGCGGTGTCGAATTACAAAAGGCTGGTATTTTCGAAAAATACGGTGTTAAAATTTTGGGCACACCTATA
GAAGCTA
TAATAGATACTGAAGACAGAAAGATTTTTAGCGAAAGAATTGCTATGATTGGTGAAAAAGTTGCTCCAAGTAT
GGCTGCT
CATTCTGTGCAAGAAGCTTTGGAAGCTGCTCATCTATTAGGATATCCGGTTATGGCGAGAGCTGCTTTTTCTT
TAGGAGG
TTTAGGTTCTGGTTTTGCAAATACAGCTGAAGAATTGAAATTGCTTGCACAACAAGCTTTAGCACATTCGAAT
CAATTAA
TTATTGATAAGTCTTTAAGAGGATGGAAGGAAGTTGAATACGAAGTTGTACGTGATGCTTTTCCATACATTAA
GGAAGTT
AATGATGATGAACTAGAAGAACCTACAGATAAACGAATGTTTGTTCTTGCAGCAGCTTTAAGAAAAGGTTACA
GTGTTGA
TAAACTTTACGACTT-
AACTAAGATAGATCGATGGTTCCTACAAAAAATGAAGAATATTATAGATTACAACACTCTTTTG
GAGTCTGCTCAACAATCTAAACAACAAATTTCGGCGAACACTTATAAACTGTTGCTAAAAGCGAAGCAAATCG
GTTTCAG
TGACAAACAAATTGCTGTCGCTGTTAAAAGCACTGAACTGGCAGTTAGAAAGCAACGACAAGATTTTGGTATC
ATTCCAT
ATGTTAAACAAATAGATACTGTAGCAGCTGAATGGCCAGCCACTACCAATTATCTATACTTAACATACAATGC
CGACAGC
CATGACTTAACATTTACCGACCAACATACTATGGTTATCGGTTCAGGAGTTTACAGAATTGGTAGTTCAGTTG
AGTTCGA
TTGGTGTGCTGTGGGATGTTTAAGAGAGCTTAGAAAACTTAATAAGAAAACAATAATGGTTAATTACAATCCG
GAAACTG
TGAGTACAGATTATGATATGTCAGACCGGCTGTACTTTGAA--------------------------------
----

CTCATATTATTTCTCAAACAAGAGGAAAAAAGGAAACTTTTGGGACTTTAGG
AATAATTTATGCAATAATAGCAATTGGGCTATTAGGGTTTATTGTATGAGCACATCACATATTTACTGTAGGA
ATAGATG
TGGATACTCGAGCATATTTTACTTCAGCAACTATAATTATTGCCGTACCTACTGGAATCAAAATTTTTAGATG
ATTAGCT
ACATTACATGGAACTCAAATTAACTTTTCTCCTTCAATACTTTGATCTTTAGGGTTTGTTTTTCTATTTACAA
TTGGGGG

ATTAACAGGAGTAATTTTAGCTAATTCATCTATTGATATTATTCTTCATGATACTTACTATGTAGTAGCACAT
TTCCATT
ATGTTTTATCTATAGGAGCAGTATTCGCAATTATAGCAGGATTAATTCAATGGTTCCCTTTATTAACAGGCCT
TACCATA
AACGATTATTTACTAAAAATTCAATTTTTTTCTATATTTATTGGTGTTAATTTAACCTTTTTTCCTCAGCATT
TTCTTGG
TTTAGCAGGTATACCCCGTCGATACTCAGACTACCCTGATGCTTACACCCCTTGAAATATTGTGTCTTCAATT
GGGTCTG
TAATTTCTTTAATTTCAATTTTTTTATTTATATTTATTATATGAGAAAGGTTTACTTCAATGCGAATTAATTT
ATCAAGA
TTAAATTTAAATTCATCAATTGAATGAATACAACTTTATCCCCCTTCAGAACATAGATACTCAGAATTACCTA
TTTTATC
GAAGGATTATGTGGTAGTATTCGACTTTCTCGGT-
AAGGATTCCATTAGATACTATAATGAAGTACCTGTAGAAAAACGT
GTCTTCAAAAACCTCCAATTATTTATGGAAAACAAATCGCCGGGAGACGATTTGTTTGATAGATTAAACACAG
CTGTGAT
GAACAAACATTTAAACGAGTTAATGGAAGGTTTAACTGCCAAGGTGTTTCGTACTTATAACGCTTCTTGGACC
TTACAAC
AGCAACTTGATAAATTGACCAATCCCGATGATTCCATATCAGAGAAAATTTTATCGTATAATCGTGCCAACAG
AGCCGTG
GCTATACTATGTAACCATCAACGTGCAGTACCAAAAGGTCATCAGAAATCGATGGAGAAGCTCAAAGAAAAAA
TAGAAAC
TAAACGGGATACTATCAAGGATGCCGAAAGGCAAGTCAAAGATGCACAAAGAGATGCAAAACA---
CGGAAGTGTTAAGG
AGAAACAAATTTATGACAAAAAGAAGAAAACGCTAGAAAGACTAAGAGAACAGTTGGCAAAATTGGAAATTCA
AGAGACG
GATCGTGATGAAAACAAGACAATTGCCCTTGGTACGTCCAAGTTGAATTATTTGGACCCTAGAATTTCCGTTG
CATGGTG
CAAGAAGTTTGGTGTACCCATTGAAAAAATTTATAACAAAACTCAATGGATGCGACTGCCGCCCTTCAGGATA
ATCGGTG
ATCACCTGAAGGATCGCTTCGATGGCGCTTCCAGAGTGATGCTGAGCAACTCGGCCAGTTCGAGGGG------
------A
AACGC---GAACCGTC---
CGAAGCAGGACAAACTGTCGAACAACATCGCTTCGAACAGCATCCACAGCAAACGCGAGAA
CCGTCCTAGGAAATACAAGTACGGTTTCCAGTTGAAACCCTACAATCCCGACCATAAGCCTCCGAGTCCCAAA
GATCTGG
TGTACTTGGAACCGTCGCCTGGGTTCTGCGAGAAGAACCCGAAGCTGGGTATTCAGGGTACTCATGGTAGACT
G-TGTAA
CGATACGTCGATTGGTGTTGATGGATGCGATTTGATGTGCTGCGGTAG-
GGGTTACAGGACCCAGGAGGTGATCGTTGTT
GAAAGGTGTAATTGCACTTT-----------
>Sunius

AGGGTGCCAGGCCCGATCGCCGGAGGATCTCTCCTCAGAGTCG
GGTTGCTTGAGAGTGCAGCCCTAAGTGGGTGGTAAACTCCATCTAAGGCTAAATATGACCACGAGACCGATAG
CGAACAA
GTACCGTGAGGGAAAGTTGAAAAGAACTTTGAAGAGAGAGTTCAATAGTACGTGAAACCGTTCAGGGGTAAAC
CTGAGAA
ACCCGAAAGGTCGAATGAGGAGATTCAGCGTGTCTCGTCTGCGGTCGCGTGACGATGGTGCTTGCACCGGACC
GCGCCTG
CCGTAGACGTAGCCGGCGACGAACTCGTGCACTTCTCCTCTAGTAGGACGTCGCGACCCGTTGGGCGCCGGTC
TAAGGCC
GACGGAGGAGCCTTGGGGTCCCGGCCGGCCCGCTCGACGGTACGACAGAGGCGTGGGGTCGCTACGTTAGCGT
CCGGCCC

GTCACAAGTTCGGGCGACTCGGACGTCGGACCTGTGTGCCGACCCCGAGCTCGCCGGCTGATGGTGGCGGTGT
CCTCGGA
CAGACTACACGCCGGTCGGCGACGCTCTAGCTTTGGGTTTTCAGGACCCGTCTTGAAACACGGACCAAGGAGT
CTAGCAT
GTACGCGAGTCATTTGGACCGCATCTAAACCAAAAGGCAAAATGAAAGTGAAGGCGTGCCGAGGGAGGATGGG
GCGGGGG
GCGTCTCGTTCTCATCGCGAGATGAGGCGCACCCA------------
GCTTACACCGTATTCGCTGACTTGTTCGA-TCC
CATTATTGAAGATTACCATGGTGGATTCAAGAAGACCGATAAGCACCCTCCCAAGAACTGGGGAGATGTAAGC
ACCTTCG
GCAATCTTGACCCAGCTGGTGAATATGTAGTCTCCACCCGCGTCCGTTGCGGCCGCTCCATGGAAGGCTATCC
ATTCAAC
CCATGCTTAACCGAAGAACAATACAAGGAAATGGAAGGCAAAGTCTCTACCACTTTGTCCGGCCTTGAAGCCG
AACTCAA
GGGTACTTTCTATCCTTTAACTGGAATGGACAAAGATACTCAACAAAAGCTCATCGATGATCACTTCTTGTTC
AAGGAAG
GAGATCGTTTCCTCCAGGCTGCAAACGCTTGCCGTTTTTGGCCATCTGGACGTGGTATTTACCATAACGACAA
CAAAACC
TTCTTGGTATGGTGCAACGAAGAAGATCACCTTCGTATCATCTCTATGCAAATGGGTGGTGATCTTGGCGAGG
TCTACCG
TCGCCTTGTAACCGCTGTCAACGAAATCGAGAAGCGTGTTCCCTTCTCCCACAATGACAGATTAGGTTTCTTG
ACCTTCT
GCCCAACCAACTTGGGCACTACTGTACGTGCCTCTGTACACATTAAAGTACCTAAGCTCGCCGCCAACAAGGC
CAAACTT
GATGAAGTTGCTGCCAAATACAACTTGCAAGTACGTGGTACTCGCGGTGTTCCTAAAAAAGTGTTAATAATTG
GGTCWGG
GGGATTATCCATTGGACAAGCAGGAGAATTCGATTATTCCGGATCACAAGCAATAAAAGCTTTACAAGAAGAA
AACATCC
AAACTGTTCTAATTAATCCAAATATCGCAACAGTACAAACCTCGAAAGGTTTAGCTGATAAAATATACTTTTT
GCCATTA
GTGCCTGAATTTGTGGAACAAGTAATTAGAGTAGAACGTCCTGGAGGCGTATTGTTAACATTTGGTGGACAAA
CAGGGTT
AAATTGTGGAGTAGAATTACAAAAAGCTGGAGTATTCGAAAAATACAATGTTCAAATTTTGGGTACCCCAATA
GAAGCTA
TAATAGATACTGAAGATAGAAAGATTTTCAGTGACAGAATTGCACTAATTGGAGAAAAAGTTGCTCCTAGTAT
GGCTGCA
TATTCTGTACAGGAAGCTTTGGAAGCTGCAGATTTATTAGGGTACCCAGTTATGGCTCGAGCTGCATTTTCTT
TRGGTGG
TTTGGGGTCTGGCTTTGCTAATACTGCAGAAGAACTGAAATTACTTGCTCAACAAGCTTTAGCTCATTCGAAT
CAGTTGA
TTATTGATAAGTCTTTAAAAGGTTGGAAGGAAGTTGAATATGAAGTTGTCAAAGATGCATATCCTTACATAAA
AGAAGTT
AATGATGATGAATTAAAGGAACCTACAGATAAAAGAATGTTTGTTCTTGCAGCAGCTTTAAGAAATGGTTACA
GTGTTGA
TAAATTGTATGATTT-
AACAAAAAATTGATCGTTGGTTCTTACAAAAAATGAAAAACATTATAGATTACAATTCACTCTTG
GAATTAGTCCAACAAAATAAATTACAAAGTTGCTCAAATATTTACAAGTTGCTATTGAAAGCGAAACAAATTG
GTTTTAG
TGATAAACAAATTGCTGTCGCTGTTAAAAGTACTGAACTTGCAATCCGAAAACAACGACAGGATTTAGGGATT
ACACCGT
TTGTTAAACAAATTGATACTGTAGCTGCTGAATGGCCTGCAACTACAAATTATTTATATTTAACGTACAACGC
TGAAAGC
CACGATATAACTTTTAATGATCAACACATAATGGTTATAGGTTCTGGTGTTTATAGAATTGGAAGCTCTGTCG
AGTTTGA
TTGGTGTGCTGTAGGTTGTTTGAGAGAACTTAGGAAATTAAATAAAAAGACAATAATGGTCAATTACAATCCA
GAAACTG

TTAGTACAGATTATGATATGTCCGATAGGTTGTATTTTGAGGAAATTTCTTTTGAAGTTGTTATGGAT-----
----

CTCATATTATTAGACAAGCTAGAGGTAAAAAGGAAACATTTGGGGCCTTAGG
AATAATTTATGCAATAATAGCAATTGGACTATTAGGATTTGTTGTTTGAGCTCATCATATATTTACAGTAGGA
ATAGATG
TGGATACACGAGCTTATTTTACTTCAGCAACTATAATTATTGCAGTTCCTACTGGAATCAAAATTTTTAGTTG
ATTAGCC
ACCCTCCATGGTACACAAATTAAATTTAATCCTTCAATATTATGATCTCTTGGATTTGTGTTTCTTTTTACAA
TTGGAGG
ATTAACAGGAGTAATTTTAGCTAACTCATCAATTGATATTATTTTACATGATACATATTATGTAGTTGCCCAT
TTTCATT
ATGTATTATCAATAGGTGCAGTTTTTGCTATTATAGCAGGATTAATACAATGATTTCCTTTATTAACTGGTTT
AGTAATA
AATGAGTATCTTTTAAAAATTCAATTTTTTATTATATTTATTGGAGTTAATTTAACATTTTTCCCTCAACATT
TTTTAGG
ATTAGCAGGAATACCTCGACGATACTCTGATTATCCAGATGCTTATACCCCATGAAATATTATTTCTTCTATT
GGATCAT
TAGTTTCCATAATAAGAATTTTTTTACTATTATTTATTATATGAGAAAGATTATCTTCAATGCGAATAAATAT
CTCTGCT
TTAAATTTTTCTTCATCAATTGAATGATTCCAATTATACCCACCAGCAGAACATAGCTATATTGAACTTCCAA
T------
-AAAGATTATGTGGTAGTGTTTGATTTCCTTGGT-
AAGGATTCCATTAGATATTATAACGAAGTACCTGTAGAGAAACGT
GTCTTCAAAAACCTTCAATTGTTTATGGAAAACAAATCCCCCGGTGATGATTTGTTTGATAGATTAAATACTG
CTGTGAT
GAACAAACATTTGAATGAGTTAATGGAAGGTTTAACTGCAAAGGTGTTTCGTACTTATAACGCTTCGTGGACA
CTACAAC
AGCAACTCGATAAATTGACCAATCCAGATGATTCCATATCCGAAAAAATTTTATCATACAATCGTGCCAATAG
AGCAGTA
GCAATACTTTGTAACCATCAACGTGCTGTACCTAAAGGCCATCAAAAATCCATGGAAAAATTGAAAGAGAAAA
TTGATAC
TAAAAGAGATACTATTAAAGACGCTGAGAGGCAAGTTAAAGATGCACAAAAAGATGCCAAGCA---
TGGGAGTGTTAAGG
AGAAGCAGATCTATGAGAAAAAGAAGAAAATGTTGGAGAGACTACGTGAGCAATTGGCTAAGTTGGAGATTCA
AGAGACC
GACCGCGATGAAAATAAAACTATTGCCCTTGGCACGTCCAAGCTGAACTATTTGGATCCTAGAATCTCGGTCG
CTTGGTG
TAAGAAGTTTGATGTGCCCATTGAAAAAATTTATAACAAAACTCAA---
ATGCGTTTGCCACCCTTCAGAGTAATCGGTG
ACCATTTGAAAGATCGTTTCGACGGCGCTTCTAGAGTGATGTTGAGCAATTCGGCTAGTTCAAGAGG------
------G
AATGC---TAATCGTC---
CAAAACAAGATAAACTTTCGAATAGTATAGCATCGAATAGTATACATAGTAAAAGAGAAAA
TAGGCCGAGGAAGTATAAATACGGTTTCCAATTGAAACCTTACAATCCCGACCACAAACCTCCGAGTCCTAAA
GATTTGG
TGTATCTGGAACCGTCGCCGGGTTTCTGCGAGAAAAACCCGAAGCTCGGGATACAAGGTACTCACGGTAGATT
G-TGTAA
TGATACTTCTATTGGTGTGGATGGATGCGATTTGATGTGTTGCGGTAG-
AGGATACAGGACCCAGGAAGTTATCGTCGTC
GAAAGGTGCAACTGCACGTT-----------
>Tachyporus

GCGAAGTGTAGTGTTTGGGAGGATCTATTAACCGTCGCGGGGCGA
GTCCAAGTCCTTCTTGAACGGGGCCACTTACCCATAGAGGGTGCCAGGCCCGACGTTGGAGAGGTCTCTCCCT
AGAGTCA

GGTTGCTTGAGAGTGCAGCCCTAAGCAGGTGGTAAACTCCATCTAAGGCAAAATAACACCACGAGACCGATAG
CAAACAA
GTACCGTGAGGGAAAGTTGAAAAGAACTTTGAAGAGAGAGTTCAAAAGTACGTGAAACCGTTCAGGGGTAAAC
CTGAGAA
ACCCGAGAGTCTGAAACGGGAGATTCATCGCGTCTYTRCGACAAACGCATGGTGACAGCGGCCCCGGCCAATC
GCACCGC
AGCTAGTCGTG--
CGGCGAAGAATGTGTGCACTTCTCCCAAAGTAGAACGTCGTGAGCCGTTGAGCTCTGGTCTACGGCC
CGATGGGGAGCCC-CGGATCCCGGCCA--
CCGCCCGACGGCATAACAAAGGCACGGAGCCGCGAAGCACGCGTCCGGCCC
GTCACAAGCGCGCTCGGCCCGGAATTCGGACCTA-
GTGCCGTTTCTGGAGCTGACGGCTGCTGGTGGCGGCGCTCTCGGA
CCGTCTACATGCCTCTCCACGACGCTTTAGCTAAGGGTTCTCAGGACCCGTCTTGAAACACGGACCAAGGAGT
CTAGCAT
GTACGCGAGTCATTGGGAAT--
TATTAAACCCATTGGCGAAATGAATGTGAAGACGCTTTTAGGGAGGATGGGAATCGGG
GCGTCTCGTTCTCATTGCGAGTTGAGGCGCACCTAGAGCGTACACGCGCTTACACCGTCTTCGCTGAACTTTT
CGA-CCC
TATCATTGAAGACTACCATACCGGTTTCAAGAAGACTGACAAGCACCCACCTAAGAACTGGGGTGATGTATCC
ACCTTTG
GAAACGTTGACCCCACCGGCGAATACGTCGTGTCCACACGCGTCCGTTGCGGTCGCTCCATGGAAGGATACCC
GTTCAAC
CCATGCTTAACCGAARAACAGTACAAAGAAATGGAACAGAAAGTGTCCACCACCCTCTCCGCCCTCGAGGGAG
AACTTAA
GGGTACCTTCTACCCATTGACTGGAATGAGCAAGGACGTTCAACAGAAACTCATCGACGACCACTTCTTGTTC
AAGGAAG
GTGACCGTTTCCTTCAAACCGCCAACGCTTGCCGTTACTGGCCATCCGGACGTGGCATCTACCACAACGATAA
CAAAACC
TTCTTGGTCTGGTGCAACGAAGAAGATYATCTTCGTCTGATTTCCATGCAAATGGGTGGTGATCTTGGCGAAG
TATACCG
TCGTCTCGTAACAGCCGTCAACGACATTGAGAAGCGTGTGCCATTCTCCCACAACGACAGATTAGGTTTCCTC
ACCTTCT
GCCCAACCAACTTGGGAACCACTGTACGTGCCTCCGTACACATCAAAGTACCTAAACTCGCCGCCAACAAGGC
TAAGCTC
GATGAAATTGCCGGCAAGTACAACTTGCAAGTCCGCGGAACCCGCGGTGTTCCTAAGAAAGTTCTTATTATTG
GGTCAGG
AGGTTTATCWATAGGTCAAGCTGGAGAATTCGATTATTCAGGATCCCAAGCGATTAAAGCATTGCAGGAAGAG
AATATTC
AAACGGTTTTGATAAATCCTAACATAGCAACAGTCCAAACATCTAAGGGCCTGGCCGATAAAGTCTACTTCTT
GCCTTTG
GTGCCGGAATATGTTGAACAAGTTATTTGTGCTGAAAGGCCAGGTGGTGTTTTACTAACATTTGGTGGGCAAA
CTGGGTT
AAACTGTGGGGTAGCTTTACAAAAGGCTGGAGTTTTTGAAAAGTATGGAGTGAAGATATTGGGCACTCCAATA
CAGGCCA
TAATTGATACCGAAGACCGGCAAGTATTTAGTGAGAAAATAGCTAGTATTGGTGAAAAAGTGGCACCCAGCAT
GGCTGCC
TATTCTGTACAAGAAGCACTGGATGCGGCTGAGCAGTTGGGGTATCCAGTAATGGCAAGAGCAGCATTTTCAT
TGGGTGG
TTTAGGATCTGGTTTTGCAAATAACAGTGATGAATTAAAGTCATTATCACAACAAGCACTGGCTCATTCAACT
CAATTGA
TAATCGATAAATCATTGAAAGGGTGGAAAGAAGTTGAAT-----------------------------------
----

GAAGTATATATT
TTAATTTTACCTGGATTTGGAATAATTTCCCATATTATTAGACAAGAAAGAGGAAAAAAAGAAACTTTCGGAG
CTTTAGG
AATAATTTATGCTATAATAGCAATTGGAATTTTAGGATTTGTTGTATGAGCTCACCATATATTTACAATTGGT
ATAGATG
TAGATACCCGAGCTTACTTTACCTCAGCAACTATAATTATTGCTGTCCCTACAGGAATTAAAATTTTCAGATG
ATTAGCT
ACACTTCATGGAACACAAATCAATTATTCCCCTTCCATATTATGAGCCCTAGGATTTGTATTTCTTTTTACAG
TTGGAGG
TTTAACTGGAGTAATTTTAGCTAATTCCTCAATTGATATTATTTTACATGATACTTATTATGTAGTAGCCCAT
TTTCATT
ATGTTTTATCTATAGGAGCAGTATTTGCTATTATAGCTGGTATTACACATTGATTTTCTTTATTTACAGGATT
AACTTTA
AATGAAAAATTTCTAAAAATTCAATTTTTAACAATATTCATTGGAGTCAATTTAACATTTTTTCCTCAACATT
TTTTAGG
ATTAGCTGGAATACCTCGACGATATTCAGATTTTCCTGATGCTTATTTAACTTGAAATATAATTTCCTCAATT
GGATCTT
TAATTTCAATAATTAGAATTTTTTTTCTTTTATTTATTATTTGAGAAAGATTTATCTCATCACGAAAAAGAAT
TTATCCA
TTAAATTTAATTTCATCAATTGAATGATTTCAATCACTTCCTCCCGCTGAACATAGTTATTCTGAACTTCCTT
TAATCAC
T--------------CGTCTTCGATTTTCTTGGT-
AAGGATTCTATTAGATATTATAATGAAGTACCTGTTGAGAAGCGT
GTTTTTAAAAATTTACAACTATTCATGGAGAATAAGGCTCCGGGTGATGATTTGTTTGATCGGTTAAACACTG
CCGTTAT
GAATAAGCATTTAAACGAACTCATGGAAGGCCTTACGGCTAAGGTTTTCCGTACCTACAATGCCTCATTTACA
TTACAAC
AGCAACTCGATAAATTAACCAACGAAGATGATTCTCTCTCTGAAAAAATTCTCTCCTACAATCGCGCGAATCG
TGCGGTC
GCAATCCTCTGTAACCATCAACGTGCAGTTCCAAAAGGACACCAAAAATCGATGGAAAAGCTCAAAGAAAAGA
TTGATTC
CAAAAGGGAGAACATTCATGATGCTGAGAGACAGGTTAAAGATGCTGAAAAGGCAGCTAAACG---
GGGTAGTGTCAAGG
AAAAGCAGATCTATGACAAGAAAAAGAAACAGTTGCAGCGGCTCAAAGAGCAGCTGGCCAAGCTAGAGATTCA
AGAAACG
GACCGTGATGAAAACAAAACTATCGCATTGGGCACGTCAAAATTGAACTATTTAGATCCCAGGATTTCGGTGG
CCTGGTG
CAAAAAG--
TGGATGCGGTTGCCGCCGTTCCGCGTGATCGGCG
ACAACCTGAAGGACCGCTTCGACGGCGCCTCCCGCGTCATGGTCAGCAACGCCGGCAACGCGCGCAACGC---
---CAAC
AACGCGCACAACCGCC---
CCAAACAGGACAAGCTCTCCAACAGCATCGCCTCCAACAGCATCCACAGCAAGCGCGAGAA
CAAACCGCGCAAATACAAGTACGGCTTCCAACTAAAACCGTACAATCCCGATCACAAACCGCCGAGCGCGAAG
GATTTGG

TGTATTTGGAACCTTCGCCTGGTTTCTGCGAGAGGAATCCGAAGTTGGGGATTCAGGGGACGCACGGGAGGCA
G-TGTAA
TGAGACGTCGATAGGGGTGGATGGGTGCGACCTCATGTGCTGCGGGAG-
GGGTTACCGCACGCAGGAGGTCGTCGTCGTC
GAGCGGTGCAACTGCACCTTCC---------
>Tetartopeus
GGGAGAAGCCCAGCACTGAATCCCGTGGCCGAACCGGGAAATGTAGTGTTTGGGAGGGTCCGTCATCCATCGT
GCGACGC
GTCCAAGTCCTTCTTGAACGGGGCCACATACCCATAGAGGGTGCCAGGCCCGATAGCTGGAGGACCTCTCCTC
AGAGTCG
GGTTGCTTGAGAGTGCAGCCCTAAGTGGGTGGTAAACTCCATCTAAGGCTAAATATGACCACGAGACCGATAG
CGAACAA
GTACCGTGAGGGAAAGTTGAAAAGAACTTTGAAGAGAGAGTTCAATAGTACGTGAAACCGTTCAGGGGTAAAC
CTGAGAA
ACCCGAAAGGTCGAATGGGGAGATTCAGCGTGTCTCGTCTTTGGTGGCGTGACGATGGTGCGTGCACCGGGAC
GCGCCCT
CCGAAGCCGCAACCCGCGGCGAACTCGTGCACTTCTCCCCTAGTAGGACGTCGCGACCCGTTGGGCGCCGGTC
TAAGGCC
GAGGGTGGAGCCTTGGGGTCCCGGCCGGCCCGCTCGACGGTAAGACAGAGGCGTGGGGTCGCGAAGTTCGCGT
CCGGCCC
GTCACAAGCGCGGGCGACTCGGACGTCGGACCTGTGTGCCGACCTCGAGCTCGCCGGCTGTTGGTGACGGTGT
CCTCGGA
CAGACTACACGCCGGTCGGCGACGCTTTAGCTTTGGGTTTTCAGGACCCGTCTTGAAACACGGACCAAGGAGT
CTAGCAT
GTGCGCGAGTCATTGGGACCGCATCTAAACCTAAAGGCGAAATGAAAGTGAAGGCGTGCCGAGGGAGGATGGG
TCGGGGG
GCGTCTCGTTCTCATCGCGAGATGAGGCGCACCCAGAGCGTACACGCTCTTACACCACTTTCGCTGATTTGTT
CGA-CCC
AATCATTGAAGACTATCATGGTGGATTCAAGAAGACCGACAAGCATCCCCCATCAAACTGGGGTGATGTTAAC
ACTTTCG
TCAACCTTGACCCTGCTGGTGAATACGTTGTATCCACCCGTGTACGTTGCGGCCGTTCAATGGAAGGTTATCC
CTTCAAC
CCTTGCTTAACCGAGGATCAATACAAGGAGATGGAACAAAAAGTTTCAACCACTTTGTCTGGACTTGAAGGCG
AACTTAA
GGGTACCTTCTACCCATTGACTGGAATGGATAAGGATACTCAACAAAAATTGATTGATGACCATTTCTTGTTC
AAGGAAG
GTGATCGTTTCCTTCAAACCGCTAACGCTTGCCGTTATTGGCCATCTGGACGTGGTATCTACCACAACGATAA
CAAAACA
TTCTTGGTCTGGTGCAACGAAGAGGATCATCTCCGTATCATCTCCATGCAAATGGGTGGTGATCTTGGTGAAG
TATACCG
TCGCCTTGTAACGGCCGTCAACGAAATTGAGAAGCGCATCCCATTCTCTCACAATGACAGATTAGGTTTCCTT
ACCTTCT
GCCCAACCAACTTGGGTACAACTGTACGTGCCTCTGTACACATCAAAGTACCTAAGCTCGCTGCCAACAAGGC
CAAGCTT
GATGAAATCGCTGGCAAGTACAACTTGCAAGTACGTGGTACCCGTGGTGTTCCAAAAAAAGTTTTAATAATTG
GATCCGG
TGGATTATCGATCGGTCAAGCTGGAGAATTCGATTATTCAGGTTCACAAGCAATTAAAGCYCTMCAAGAAGAA
AATATAC
AAACAGTTTTAATTAATCCAAACATTGCAACGGTACAAACTTCCAAAGGCTTGGCCGATAAAGTTTATTTCCT
ACCTTTA
GTACCTGAATATGTAGAGCAAGTTATTCGGGTTGAAAGACCTGGAGGTGTATTATTAACATTTGGCGGACAAA
CAGGGTT
AAATTGTGGTGTTGCCCTTCAAAAAGCTGGAGTTTTYGAAAAATACGGTGTTAAAATTTTGGGCACACCTATT
CAAGCAA
TTATCGATACMGAAGATCGAAAAATTTTTAGTGATAGAATAGCATTAATTGGAGAAAAAGTTGCACCAAGTAT
GGCTGCA

TATTCTGTACAAGAAGCTTTAGAAGCTGCCGAGTTATTAGGATACCCTGTAATGGCAAGAGCAGCATTTTCAT
TAGGAGG
TTTAGGTTCTGGATTTGCAAATACCTCGGAGGAGTTAAAATCTTTAGCGCAACAAGCACTTGCACATTCCAAT
CAATTAA
TTATTGATAAGTCTTTGAAAGGGTGGAAGGAAGTTGAATATGAAGTTGTAAGGGATGCATATCACGATATCAA
GCAAGTT
AACGATGAAGAATTAAAGGAACCTACAGATAAGAGAATGTTTGTAGTTGCAGCTGCATTAAAAAGTGGTTATA
GCGTTGA
TAAATTATATGATTT-
AACGAAAATTGATCGATGGTTTTTGCAAAAAATGAAAAATATTATTGATCTTACGACTTTACTA
GAATCGACAGAACAAATAAAATTA---------------
ACAGCTAATATTTTATTAAAAGCAAAGCAAATTGGATTTAG
TGATAAACAGATTGCTGCTGCTTGTAAAAGTACGGAACTTGCTATAAGAAAACAACGTCAAGATTTTAATATT
ACTCCGT
ATGTTAAGCAGATTGATACTGTCGCTGCTGAATGGCCGGCTACTACGAATTATCTATACTTAACTTATAATGC
TGGAAGT
CATGATTTAACCTTTGCTGAAGAGCATACAATGGTTATAGGTTCAGGAGTTTATAGAATTGGTAGTTCTGTTG
AATTTGA
TTGGTGTGCAGTTGGATGTTTACGTGAGTTAAGAAAATTAAATAAAAAAACAATCATGGTAAATTACAATCCG
GAGACTG
TAAGTACTGACTACGATATGTCAGATAGGTTGTACTTTGAAGAAATCTCATTTGAAGTTGTTATGGATGAAGT
TTATATT
TTAATTTTACCAGGATTTGGAATAATTTCTCATATCATTAGGCAAGCCAGAGGAAAAAAGGAAACTTTTGGAA
CTCTAGG
TATAATTTATGCAATAATAGCAATTGGATTACTAGGATTTGTAGTATGAGCCCATCATATATTTACTGTGGGT
ATAGATG
TAGATACCCGAGCTTATTTTACATCAGCAACAATGATTATTGCTGTACCAACTGGAATTAAAATTTTCAGATG
ATTAGCC
ACTTTACATGGAATCCAACTAAACTTTAACCCGCCTACTCTATGAGCTCTAGGATTTGTATTCTTATTTACTA
TTGGAGG
ATTAACTGGAGTTATCCTAGCTAATTCTTCTATTGATATTATTTTACATGATACTTATTATGTAGTAGCTCAT
TTTCATT
ATGTTCTGTCAATAGGAGCTGTATTTGCAATTATGGCAGGATTAGTTCAATGATTCCCCTTATTTACAGGACT
TAGACTA
AATGAAAAATTATTGAAAATTCAATTTTTTTCAATATTCATTGGAGTAAATTTAACCTTTTTTCCTCAGCATT
TTCTTGG
ATTAGCCGGGATACCTCGACGATACTCTGATTATCCTGATGCCTATACAACCTGAAATGTAATTTCATCAATT
GGATCTT
TAATTTCAACTATAAGTATTTTACTTTTATTATTTATTATTTGAGAAAGGTTTTCCTCATCTCGTATAATTTT
ATCAACT
AAAAATTTTTCTACTTCAATTGAATGATACCAATCCTATCCTCCCTCTGAACACAGATATAATGAACTACCTA
TGCTATC
AAAGGATTACGTTGTGGTCTTTGATTTCCTCGGT-
AAGGATTCSATTAGATATTACAATGAAGTACCTGTGGAGAAACGT
GTTTTCAAAAACCTCCAATTGTTCATGGAAAACAAGGCGCCCGGCGATGATTTATTCGATAGGCTTAACACGG
CTGTTAT
GAATAAGCATTTAAACGAGCTAATGGAAGGTTTAACCGCCAAGGTATTTCGTACTTATAACGCCTCTTGGACT
TTACAAC
AACAACTCGAGAAACTCACAAATGAAGACGATTCCATATCCGAGAAGATCTTATCGTATAACCGTGCCAATAG
GGCGGTG
GCTATCCTCTGTAACCATCAACGTGCCGTACCTAAAGGCCATCAGAAATCTATGGAGAAATTAAAGGAAAAAA
TTGAAAC
TAAAAAGGAGAATATTAGAGATGCAGAACGGCAGGTAAAAGATGCGCAGAAGGACGCAAAGCA---
TGGAAGCGTTAGGG
AGAAGCAGATCTACGATAAGAAGAAGAAAATGTTGGAGCGGCTTAGGGAGCAACTGGCGAAGCTCGAGATACA
GGAGACG

GACCGCGACGAGAATAAGACAATTGCTCTCGGTACGTCCAAGCTGAATTATTTGGACCCGAGGATCTCGGTTG
CTTGGTG
TAAGAAATTCGGTGTGCCCATCGAAAAA---------------------
ATGCGGCTTCCCCCGTTTCGCGTGATCGGTG
ACCACCTGAAGGACCGCTTCGACGGTGCCTCGCGTGTCATGCTCAGCAACTCAGCGAGTTCCCGAGG------
------C
AACGC---CAATCGTC---
CCAAACAGGATAAGCTCAGCAACAACATAGCTTCCAACAGTATACACAGTAAAAGGGAGAA
TCGCCCAAGGAAATACAAGTATGGCTTCCAGCTTAAGCCGTATAATCCTGACCATAAACCACCGAGCCCTAAA
GATTTGG
TGTATCTAGAGCCATCACCTGGTTTCTGCGAGAAGAATCCGAAATTAGGGATACAAGGCACGCATGGAAGACA
G-TGCAA
CGATACTTCAATAGGTGTTGATGGTTGCGATTTAATGTGTTGTGGAAG-AGGTTACAGGACCCAGG-------
----

>Thinocharis
GGGAGAAGCCCAGCACTGAATCCCGTGGCCGAACCGGGAAATGTAGTGTTTGGGAGGGTCCGCCATCCATCGT
CCGACGC
GTCCAAGTCCTTCTTGAACGGGGCCACATACCCACAGAGGGTGCCAGRCCCGATAGCCGGAGGATCTCTCCTC
AGAGTCG
GGTTGCTTGAGAGTGCAGCCCTAAGTGGGTGGTAAACTCCATCTAAGGCTAAATATGACCACGAGACCGATAG
CGAACAA
GTACCKTGAGGGAAAGTTGAAAAGAACTTTGAAGAGAGAGTTCAATAGTACGTGAAACCGTTCAGGGGTAAAC
CTGAGAA
ACCCGAAAGGTCGAATGGGGAGATTCAGCGTGTCTCGTTTCTGGTCGCGTGACGGTGGTGCTCGCACCGTGCC
GCGCCTT
CCGGATCCGTAACCGGAGACGAACTCGTGCACTTCTCCCCTAGTAGGACGTCGCGACCCGTTGGGTGCCGGTC
TAAGGCA
GGCGGTGGAGCCCCGGGG-
CCCGGCCGGCCCGCTCGACGGTAAGACAGAGGCGTGGGGTCGCTACGTTAGCGTCCGGCCC
GTCACAAGTTCGGGCGCCTCGGATGTCGGACTTTTGTGCCGACCCCGAGCTCGCCGGCTGCTGGTGGCGGTGT
CCTCGGA
CAGACTACACGCCGGTCGGCGACGCTCTAGCTTTGGGTTTTCAGGACCCGTCTTGAAACACGGACCAAGGAGT
CTAGCAT
GTGCGCGAGTCATTGGGACCCGTACTAAACCTAAAGGCGAAATGAAAGTGAAGGCGTGCCGAGGGAGGATGGG
TCGGGGG
GCGTCTCGTTCTCAACGCGAGGTGAGGCGCACCCAGAGCGTACACGC--------------
CGCTGATTTGTTCGA-TCC
CATCATTGAAGATTACCATACTGGCTTCAAGAAGAGCGATAAGCATCCCCCGAAGAACTGGGGTGATGTAAAC
ACCTTCG
CCAATCTCGACCCAGCCGGTGAATATGTAGTCTCCACCCGCGTCCGTTGCGGCCGCTCCATGGAAGGCTACCC
CTTCAAC
CCGTGCTTAACCGAAGAGCAATACAAGGAGATGGAGACCAAAGTCGCCGGTACTCTGTCCGGCATGGAAGGAG
AACTCAA
GGGTACATTCTACCCRTTGACCGGCATGGACAAGGCTACCCAGCAGAAACTCATCGACGACCACTTCCTGTTC
AAGGAGG
GTGACCGTTTCCTCCAGGCCGCGAATGCTTGCCGCTTCTGGCCATCCGGACGTGGTATCTACCACAACGACAA
CAAAACC
TTCTTGGTCTGGTGCAACGAAGAGGACCATCTCCGCATCATCTCCATGCAGATGGGCGGCGATCTTGGCGAAG
TCTACCG
TCGCCTCGTGACCGCCGTCAACGAAATCGAGAAGCGCGTCCCGTTCTCCCACAATGACAGATTAGGTTTCCTC
ACCTTCT
GCCCAACCAACTTGGGCACTACTGTACGTGCCTCTGTACACATCAAAGTACCTAAGCTCGCGGCCAACAAGGC
CAAGCTC
GATGAGGTCGCCGGCAAGTACAACTTGCAAGTACGTGGTACCCGTGGC-------------------------
----

GCTGGGGAGTTCGACTATTCTGGTTCGCAAGCAATTAAAGCGTTACAAGAAGAAAATATAC
AAACAGTACTAATTAATCCAAACATAGCTACTGTACAAACATCAAAAGGTTTAGCAGATAAAATTTACTTTTT
ACCATTA
GTGCCTGAATTTGTGGAACAAGTGATTAGAGTAGAACGTCCTGGCGGTGTTTTGTTAACATTTGGCGGACAAA
CAGGGTT
AAATTGTGGAGTGGAATTACAAAGAGCTGGTGTATTCGATAAATACGGTGTAAAAATTTTGGGTACACCTATT
CAAGCGA
TTATAGATACAGAGGATAGAAAAATTTTTAGTGAAAGAATATCATTAATCGGAGAAAAAGTTGCTCCTAGTAT
GGCAGCA
TATTCAGTACAAGAAGCACTTGAAGCAGCTGATTTACTAGGCTATCCTGTCATGGCAAGAGCTGCTTTTTCTT
TGGGTGG
TTTAGGATCCGGTTTTGCGAATAACGATGAAGAACTTAAATTACTATCTCAACAAGCTTTAGCACATTCC---
----

CCATACTTAAAAGAAGTA
GACGATGACGAATTGAAAGAACCTACTGATAAACGAATGTTTGTAATTGCGGCCGCTCTAAGAAATGGTTACA
GCGTAGA
TAAGTTGTATGATTT-
AACAAAAAATCGATCGCTGGTTCTTACAAAAAATGAAAAACATTATAGATTACAACACTCTTTTG
GAATCAGTCC------
ATAAGTTACAGAACTGTTCGAATACCTATAAACTTTTGTTGAAAGCGAAACAAATCGGTTTCAG
TGATAAACAAATTGCTGTCGCAGTTAAAAGTACTGAACTTGCTATCAGAAAACAACGACAAGATTATGGTATA
ACTCCAT
ATGTTAAACAAATTGATACTGTTGCCGCTGAATGGCCGGCTACTACAAATTATTTGTATTTAACGTACAATGC
AGAAAGT
CATGATTTAACTTTCAGTGATCAACACATAATGGTTATAGGATCTGGCGTTTACAGAATTGGAAGTTCTGTTG
AGTTTGA
TTGGTGTGCTGTTGGATGCTTAAGAGAACTTAGAAAGTTAAACAAAAAAACTATAATGGTCAATTATAATCCA
GAAACTG
TTAGTACAGATTATGATATGTCAGATAGATTATACTTTGAAGAAATTTCATTTGAAGTTGTTATGGAT-----
----

TATTATTAGACAAGCAAGAGGAAAAAAAGAAACATTTGGTTCTTTAGG
AATAATTTATGCTATAATAGCAATTGGATTATTAGGATTTGTTGTATGAGCTCACCATATATTTACAGTAGGA
ATAGATG
TTGATACTCGAGCTTATTTTACTTCTGCAACTATAATCATTGCAGTTCCTACTGGAATTAAAATTTTTAGATG
ATTAGCC
ACTCTTCATGGAACACAAATTAAATTTAATCCTCCAATACTTTGATCATTAGGGTTTGTTTTCTTATTCACAA
TTGGAGG
ACTTACTGGTGTAATTCTAGCAAATTCATCAATTGATATTATTTTACATGACACTTATTATGTTGTTGCACAT
TTTCATT
ATGTTCTATCCATAGGGGCGGTATTTGCAATTATAGCTGGATTAGTTCAATGATTCCCTCTTTTTACTGGATT
AATAATA
AATGAATATTTATTAAAAATTCAATTTTTCATTATATTTATTGGAGTAAATTTAACTTTTTTCCCTCAGCATT
TTCTTGG
ACTAGCGGGTATACCTCGACGTTATTCTGATTATCCAGATGCTTACACTCCATGAAATATAATTTCATCAATT
GGCTCAT
TAATTTCAATAATTAGAATTTTTATTTTATTATTTATTTTATGAGAAAGATTTACATCTATTCGAATAAATAT
TTCATCA
AAAAATTTTTCCACATCAATTGAATGATTAC--

-AAAGATTATGTAGTAGTATTTGATTTCCTCGGT-
AAAGATTCCATTAGATATTACAATGAGGTACCTGTGGAAAAACGT
GTCTTTAAAAACCTTCAGTTGTTTATGGAAAACAAATCACCAGGCGATGATTTATTTGATAGATTAAACACAG
CTGTGAT

GAACAAACATTTAAATGAGTTAATGGAAGGTTTAACTGCCAAGGTGTTTCGTACTTACAATGCTTCTTGGACT
TTGCAAC
AACAACTCGACAAATTGACCAATCCAGATGATTCCATATCTGAGAAAATTTTATCATACAACCGTGCGAACAG
AGCGGTA
GCTATACTCTGTAACCATCAACGTGCTGTACCTAAAGGCCATCAGAAGTCCATGGAGAAACTTAAAGAGAAAA
TCGACGC
TAAACGGGACACAATTAAAGACGCCGAGAGACAAGTTAAAGACGCCCAGAAAGATGCTAAACA---
CGGAAGCGTCAAAG
AGAAGCAGATCTACGAGAAGAAGAAGAAGATGCTGGAGAGGATGAGGGAGCAACTGGCTAAATTGGAGATTCA
GGAAACG
GACCGCGACGAGAACAAGACCATCGCCCTTGGCACGTCCAAGCTGAACTATTTGGACCCTAGAATCTCGGTCG
CCTGGTG
CAAGAAGTTCGAGGTGCCCATTGAA---------------------
TGGATGCGGCTGCCGCCGTTCCGCGTGATCGGCG
ACCACCTGAAGGACCGCTTCGACGGCGCCTCGCGCGTCATGCTCAGCAACTCGGCGAGCTCGCGCGG------
------C
AACGC---GAACCGGC---
CGAAGCAGGACAAGCTCTCGAACAGCATCGCCTCGAACAGCATCCACAGCAAGCGCGAGAA
CAGGCCGCGCAAGTACWAGTATTGGTTCCAGCTGAAGCCGTACAACCCGGACCACAAGCCGCCGAGTCCCAGG
GACCTTG
TGTWCCTGGAGCCGTCGCCCGGTTTCTGTTAGAAGAACCCGAAGCTCGGCATACAGGGCACGCACGGGAGGCT
G-TGCAA
CGAGACTTCGATCGGCGTCGACGGGTGCGACCTGATGTGCTGCGGCAG-
GGGGTACAGGAGCCAGGAGGTGATCGTCGTC
GAGCGGTGCAACTGCACTTTCCA--------
>Thyreocephalus_annulatus
GGGAAAAGCCCAGCACCGAATCCCGCGGTCGTGCCGGGAAATGTGGTGTTAGGGAGGAT-
CACTGTCCGTCGTGCGGCGC
GTCCAAGTCCACCTTGAACGGGGCCACTTACCCATAGAGGGTGCCAGGCCCGGCAACGGGAGGATCTCTCCTC
AGAGTCG
GGTTGCTTGAGAGTGCAGCCCTAAGTGGGTGGTAAACTCCATCTAAGGCTAAATATAACCACGAGACCGATAG
CGAACAA
GTACCGTGAGGGAAAGTTGAAAAGAACTTTGAAGAGAGAGTTCAACAGTACGTGAAACCGTTCAGGGGTAAAC
CTGAGAA
ACTCGAAAGATCGAATGGGGAGATTCAGCGCGTCTCGGTGGCGGTGATGTGACGGTGACGTTCGCGTTGG-
CCGCCTCGC
C-GTTACCGCAGTT--
TGACGAACGTGTGCACTTCTCCCCTAGTAGAAGGTCGTGACCCGTTGGGTGCCCGTCTACGGCC
CGCGGTGGAGACCGTGCGTCCTGGCCGGCCCGCTCGACGGTATGAAGTTGGCGAGGGGCCGCGATTTTCGCGT
CCGGCAC
GCGACAAGCACGGACGATCTGTCTGTCGGACCTG-
GTGCCGACGGCGGATCCGTCGGCTGCTGGTTGTGTTGTCCTCGGA
CAGACCATACGCCTGTCAGCGACGCCTTTGCATTGGGTTCTCAGGACCCGTCTTGAAACACGGACCAAGGAGT
CTAGCAT
GTGCGCGAGTCATTGGGACT--
AGCGAAACCTAAAGGCGAAATGAAAGCAAAGGCGTGCCGAGGGAGGATGCGGAGTGGG
GCGTCTCGAGCTCATCGCGAGCTGAGGCGCACCTAGAGCGTACACGC--------------------------
----

------G
GCAACCTCGACCCCACTGGCGAGTTCGTCGTGTCTACTCGTGTCCGTTGCGGCCGCTCCATGGAGGGATATCC
CTTCAAC
CCTTGCCTCACTGAGGAGCAGTACAAGGAGATGGAACAGAAGGTTTCCGGCACTCTGTCCGGTCTCGAGGCAG
AACTCAA
GGGTACCTTCTACCCCCTCACCGGCATGAGCAAGGAAGTCCAGCAGAAGTTGATCGACGATCACTTCTTGTTC
AAGGAGG

GTGACCGTTTCCTCCAGGCCGCCAACGCTTGCCGTTTCTGGCCCAGCGGCCGTGGCATCTACCACAACGATAA
CAAGACC
TTCTTGGTCTGGTGCAACGAGGAGGATCACCTCCGCATCATTTCCATGCGGATGGGCGGCGACTTGGGTGAAG
TTTACCG
CCGTCTGGTCACAGCCGTAAACGAGATCGAGAAGCGCGTCCCCTTCAGCCACAACGACAGGCTCGGCTTCCTC
ACCTTCT
GCCCGACCAACTTGGGCACCACCGTTCGTGCCTCCGTGCACATCAAGGTCCCCAAGCTCGCCGCCAACAA---
----

GTTCCTAGAAAAGTGTTAATCATTGGTTCTGG
TGGACTTTCCATTGGTCAAGCAGGAGAATTCGATTACTCAGGGTCACAGGCAATAAAAGCTTTACAAGAGGAA
CACATTC
AAACCGTCCTCATTAATCCAAACATTGCTACTGTACAAACATCGAAAGGTTTGGCAGATAAAGTATACTTTTT
ACCTCTC
GTTCCCGAATACGTGGAAGAAGTGATTAGATCTGAACGTCCAGGAGGAGTACTTCTCACATTTGGTGGCCAAA
CAGGTTT
AAACTGTGGGGTGGAATTAGAAAAAGCTGGTGTATTTGCCAAATACAATGTGAAGATCTTAGGAACACCAATT
CAAGCTA
TCATAGACACGGAAGATAGGAAAGTTTTTAGTGAAAGAATTTCTTCAATTGATGAAAAGGTAGCTCCCAGTTT
GGCAGCT
TATTCGGTTCAAGAAGCTTTGGATGCTGCTGAAAAATTAGGGTATCCGGTTATGGCACGTGCAGCTTTTTCAC
TAGGAGG
CTTAGGTTCCGGTTTTGCTGACACTGCTGATGAATTGAAATCTTTAGCCCAACAAGCCTTGGCACATTCAACT
CAATTAA
TCATTGATAAATCACTAAAGGGTTG--------------------------------------
CCGTATATAAAAAATGTA
AACGATGAAGAATTGAAAGAACCCACAGACAAAAGGATGTTTGTTGTGGCTGCAGCACTCAAGCACGGATATA
CTGTAGA
CCAATTATATAATTT-
AACTAAAATTGATAGATGGTTCCTGCAAAAAATGAAAAACATCATTGACTACTTCTCATATTTA
GAATCGATTGATT------GCATA---------------
TCAAAAGGGGTTCTTTTAAAAGCGAAACAACTTGGCTTCAG
TGATAAACAAATCGCAGCTGCTGTTAAAAGCACAGAATTGGCTATTAGGAAGCAAAGGAATGATTTAAACATC
ACACCAT
ATGTAAAACAAATTGATACTGTAGCTGCTGAATGGCCAGCTACAACTAACTACCTGTATTTAACATACAACGC
CAATTCC
CACGATCTTACTTTCAATGATGCGCATACTATGGTTATCGGATCTGGCGTTTACAGAATTGGTAGTTCTGTGG
AGTTTGA
TTGGTGCGCCGTTGGTTGTTTAAGAGAATTAAGGAAGTTGAATAAAAAGACCATAATGGTTAACTACA-----
----

TTACCTGGATTTGGAATAATTTCTCATATTATTAGACAAGAAAGAGGAAAAAAGGAAGCATTCGGAACTTTAG
G
AATAATTTATGCAATAATAGCAATTGGTCTTTTAGGTTTTATTGTTTGAGCTCATCACATATTTACAGTTGGA
ATAGATG
TTGATACTCGAGCTTACTTTACTTCAGCAACTATAATTATTGCTGTACCAACAGGAATTAAAATTTTCAGATG
ATTAGCC
ACTCTACATGGAACACAAATTAATTATTCTCCCTCTATGCTTTGAGCTTTAGGGTTTGTATTTTTATTTACTG
TTGGAGG
ACTGACAGGAGTAATTCTAGCTAATTCATCAATTGATATTATTTTACATGATACCTACTATGTAGTAGCTCAT
TTTCATT
ATGTGCTATCTATAGGAGCAGTATTTGCTATTATAGCAGGATTAGTACATTGATTTTCTCTATTTACTGGTTT
AACTTTA
AACGAAAAATTCCTAAAAATTCAATTTTTAACTATATTTATTGGAGTAAATTTAACCTTTTTTCCCCAACATT
TCTTAGG

ATTAGCTGGTATACCTCGACGATACTCTGATTATCCTGACGCTTACACTCCTTGAAATGTTCTTTCTTCTATT
GGTTCAT
TAATTTCATTAAGAAGAGTTTTCTTCCTTCTATTTATTATTTGAGAAGGATTTGTTGCAAAACGAAAAAGAAT
TTCGGCT
TTAAATTTACCTTCCTCAATTGAATGACTTCAAGAAATACCTCCTGCTGAGCATAGCTAT-------------
----
-AAGGATTATGTTGTCGTCTTCGACTTCCTAGGT-
AAAGATTCCATAAGGTACTACAATGAGGTGCCTGTGGAAAAGAGA
GTATTCAAGAACCTCCAATTGTTCATGGAGAACAAATCGCCGGGTGATGATCTGTTTGATAGACTGAACACTG
GCGTGAT
GAACAAGCATTTAAACGAGCTCATGGAAGGGCTTACAGCGAAAGTGTTCCGTACGTATAATGCCTCATTCACA
CTGCAGC
AGCAATTAGACAAGCTTACAAACCCCGATGATTCCCTCTCCGAGAAGATCTTGGCGTACAACCGGGCGAATCG
CGCTGTT
GCCATCCTCTGTAACCATCAGCGCGCCGTTCCCAAGGGCCACCAGAAGTCAATGGAGAAGCTAAAGGAGAAGA
TCGACTC
AAAACGAGACGCAATATCAGATGCTGAGCGCCAAGTTTCGGATGCAAAACGCGATGCCAAGCA---
CGGCAGCGTCAAAG
AAAAGGTTGTTTACGAGAAGAAGAAGAAAATGCTGGAGAGACTGCGTGAGCAGCTTAGCAAGCTCGAGATCCA
AGAGACA
GACAGGGACGAGAACAAAACAATAGCGCTGGGCACGTCCAAGCTCAATTATCTGGACCCCCGTATCTCGGTGG
CCTGGTG
CAAGAAGTACGATGTGCCCATCGAGAAAATCTACAACAAAACCCAG---------
TTACCACCATTCAGGGTCATCGGTG
ACAACCTCAAAGATCGTTTCGATGGCGCCACTAGGGTGATGCTCAGCAACACTGCAAACTCCCGCAACCTAAA
CGGTCCA
AACTC---CAATAGAC---
CTAAACAAGACAAACTATCAAAYGCCATATCCTCCAATAGCATCCACAGTAAACGCGAGAA
CAAACCCCGAAAATACAAATACGGCTTCCAGCTCAAACCTTACAATCCTGACCATAAACCTCCAAGCCCCAAA
GACTTGG
TGTATCTGGAACCATCGCCAGGCTTCTGCGAACGCAACCCCAAGCTAGGCATCCAAGGCACCAATGGTAGACA
G-TGCGA
TGACACTTCAGTGGGAGTAGATGGCTGCGATCTG---------------------------------------
----

>Haplonazeris

AAGCACCCTCCTTCTAACTGGGGTGATGTAAACACCTTCK
TTAATCTTGATCCAGCTGGTGAATACGTASTCTCCACCCGCGTTCGTTGTGGTCGCTCCATGGAAGGCTATCC
CTTCAAC
CCCTGCTTAACTGAARAACAATACAAARAAATGGAAGGTAAAGTTTCCAGCACTTTATCTGGTCTCRAAGGTG
AACTCAA
GGGTACTTTCTACCCATTGACTGGAATGGATAAARACACCCAACARAASCTCATCRATGATCACTTCTTGTTC
AAGGAAG
GTGATCGTTTCCTTCARGCTGCCAAC?????????????????????????????????TACCACAACGACAA
CAAAACA
TTYTTGGTYTGGKGCAACGAAGAGGATCATYTTCGCATTATYTYTATGCAAATGGGKGGAGATYTTGGTGAAG
TYTATYG
TCGCCTTGTAACCGCTGTCAACGAAATGGAGAAGCGCGTTYCCTTYTCCCACAATGACAGATTAGGTTTCCTC
ACTTTYT
GCCCATCCAACTTGGGCACAACTGTACGTGCCTCTGTACACATTAAAGTACCTAAGCTCGCTGCCAACAAGGC
CAAGCTC
GATGAAATCGCTGCC--

TGGATGCGCCTTCCGCCCTTCAGAGTWATCGGCG
ACCATCTAAAAGACCGCTTCGATGGCGCTTCCAGAGTCATGCTCAGCAATTCGGCGAGTTCCCGAGG------
------A
AACGC---CAACCGTC---
CGAAGCAAGACAAACTTTCGAACAGCATAGCTTCGAATAGCATTCACAGCAAAAGGGAGAA
CCGACCGCGGAAATACAAATACGGTTTTCAATTGAAACCTTACAATCCGGACCATAAACCTCCGAGTCCTAAG
GATTTAG
TGTACTTGGAACCGTCGCCTGGTTTCTGCGAGAAGAACCCGAAGCTTGGTATACAGGGTACTCATGGTAGGTT
G-TGCAA
CGATACTTCCATTGGTGTTGATGGATGCGATTTGATGTGCTGCGGTAG-
GGGCTACAGGACACAGGAAGTCATCGTTGTT
GAGAGGTGCAACTGCACGTTCCAYTGGTGCT
>Leptobium

CATATACCGTTTTCGCGGACTTGTTCGA-CCC
CATCATTGAAGACTACCACACTGGATTCAAAAAGACCGACAAACACCCCCCCAAGAACTGGGGTGATGTGAAC
GTGTTTA
CCAACCTGGACCCTGCTGGTGAGTACGTCGTTTCGACCCGAGTCCGCTGTGGCCGTTCTATGGAAGGATACCC
CTTCAAC
CCATGCTTAACCGAGGAGCAGTACAAAGAGATGGAGCAGAAGGTATCCACCACCTTGTCCGGACTTGAGGGCG
AGCTCAA
AGGAACATTCTACCCTTTAACTGGCATGGATAAAGATACTCAACAAAAACTCATCGATGACCACTTCTTGTTC
AAGGAGG
GTGATCGTTTCCTTCAAGCCGCAAATGCTTGCCGTTTCTGGCCCAGCGGACGTGGTATCTACCACAACGACAA
CAAGACC
TTCTTGGTCTGGTGCAACGAAGAGGACCATCTTCGTATCATCTCCATGCAGATGGGCGGTGATCTTGGTGAAG
TATACCG
TCGCCTCGTTACCGCTGTTAACGACATTGAGAAGCGTGTCCCCTTCTCCCACAATGATAGGCTGGGTTTCTTA
ACTTTTT
GCCCAACCAACTTGGGTACAACTGTACGCGCCTCTGTACATATTAAGGTCCCCAAACTAGCCGCCAACAAGGC
CAAGCTT

CCATATTTAAAAGAAGTT
AATGATGAAGAATTGAAAGAACCAACAGATAAAAGGATGTTTGTATTAGCAGCAGCTTTAAAGAATGGTTATA
GTGTAGA
TAAATTATACAACTT-
AACCAAAATTGACCGTTGGTTTATTCAAAAAATGAAGAATATAATTGATTTCAATAACAAATTA
GAATCTATAGATCAACACAAATTA--------------
TCAGCTGAAATTTTACTTAAAGCTAAACAAATTGGCTTCAG
TGATAAGCAAGTAGCAACAGCTGTCAAAAGTACAGAATTAGCAATTAGAAAACAGCGACATGATTTTGGGTTA
ATTCCAT
TTGTTAAACAGATTGATACTGTTGCTGCAGAATGGCCAGCTACTACAAATTACCTTTATATAACCTACAATGC
ATCAAAT

CATGATTTAGAATTCACAGAAGAACATATTATGGTCATAGGTTCGGGTGTGTACAGAATTGGTAGCTCGGTTG
AATTTGA
TTGGTGTGCTGTTGGTTGTTTGAGAGAATTGAGAAAATTGAATAAGAAAACTATAATGGTTAATTATAACCCT
GAAACTG
TTAGTACAGATTATGATATGTCAGATAGATTATACTTTGAGGAAATATCTTTTGAAGTTGTTATGGATGAAGT
TTATATT
TTAATTTTACCTGGATTGGAAATAATTTCTCATATTATTTCATATAGAAGAGGTAAATCCGAAACTTTTGGAT
CATTAGG
AATAATTTATGCAATATTAGCAATTGGCCTATTAGGATTTATTGTTTGAGCCCATCATATATTTACTGTAGGA
ATAGATG
TTGATACTCGAGCCTACTTTACATCTGCTACTATAGTAATTGCTGTTCCTACCGGAATCAAAGTATTTAGATG
AATAGCA
ACAATTTATGGGGGAAACTTAAACTTTAACCCTCCTATATTATGATCTTTAGGTTTTGTATTCTTATTTACAG
TTGGTGG
ACTAACAGGTGTTATTTTAGCTAATTCATCAATTGATATTATTCTTCATGATACATATTATGTAGTTGCTCAT
TTTCATT
ATGTTTTATCTATAGGAGCTGTATTTGCTATTATAGCAGGACTTGTTCAATGATTTCCATTATTTGTAGGATA
TACCTTA
AATGAAAAATACTTAAAAATTCAATTTTTAATTATATTTATTGGAGTAAATTTAACATTTTTCCCTCAACATT
TTTTAGG
TCTTGCAGGAATACCTCGACGATACTCAGATTATCCAGATGCCTATACAACTTGAAATGTAATTTCTTCAATT
GGTTCAA
TAGTATCATTTATTGGAATTATGTTCTTTTTATGAATTATTTGAGAAAGATTTGTATCAATACGAAAAATCCT
AGGATCA
CCTATTCCACCAACAGCTATTGAATGAATACACTCATACCCTCCCTCAGAACATACATATTCTGAATTACCTT
ATATTAC
A---------GTTGTTGTGTTTGATTTCCTCGGC-
AAAGATTCCATTAGATATTATAACGAAGTACCTGTCGAGAAACRC
GTCTTTAAAAATCTGCAGCTCTTTATGGAGAATAAAAAACCCGGTGATGACCTATTTGATCGGTTAAACACAG
CTGTTAT
GAACAAACATTTAAACGAACTTATGGAAGGTCTAACAGCGAAGGTATTTCGTACTTACAATGCTTCTTTTACT
TTGCAAC
AGCAATTAGACAAATTAACCAATCCWGATGACTCTTTGTCGGAGAAAATTTTATCATATAACCGTGCTAATCG
AGCCGTA
GCTATCCTTTGTAACCATCAGCGTGCCGTTCCCAAGGGTCACCAGAAATCAATGGAGAAGCTCAAAGAGAAAA
TTGACGC
TAAGAGGGAAGCCATTAAAGATGGAGAAAGGCAAGTGAAGGATGCCCAACGAGATGCTAAACA---
TGGCAGCGTCAAGG
AAAAGCAGATCTATGATAAGAAGAAAAAACAGCTGGAGAGATTCAAAGAGCAGCTTGCAAAACTAGAAATACA
AGAGACG
GACCGTGACGAGAACAAAACTATTGCACTTGGTACGTCAAAACTGAATTATTTGGATCCTAGGATATCGGTTG
CTTGGTG
TAAAAAATTCGACGTGCCCATTGAGAAAATTTATAACAAAACTCAA---------------------------
------G
GGAACCTGAAGGACCGATTCGACGGTGCATCGCGAGTCATGCTCAGCAATTCGGCAAGCTCGAGGAA------
----
--------CAACAGGC---
CCAAACAGGATAAACTCAGTAACAGCATAGCGTCGAACAGCATACACAGCAAACGAGAGAA
CAGACCGCGCAAATACAAATACGGTTTCCAACTGAAGCCCTACAATCCGGACCATAAGCCGCCCAGCCCGAAG
GACCTGG
TCTACCTGGAGCCTTCGCCCGGTTTTTGCGAGAAAAATCCCAAATTGGGCATACAAGGCACGCACAGCAGACA
A-TGCAA
CGACACGTCGATAGGAGTCGATGGTTGTGACCTCATGTGCTGCGGGAG-
GGGCTACAGGACGCAAGAAGTCGTCGTC---
