# Supplementary material for: The oldest case of paedomorphosis in rove beetles and description of a new genus of Paederinae from Cretaceous amber (Coleoptera: Staphylinidae)
Source: Sci Rep. 2023 Mar 31;13:5317. doi: 10.1038/s41598-023-32446-2 (PMC10066364; doi:10.1038/s41598-023-32446-2)
Supplement: Supplementary file 8 — Supplementary Information 8. [file 41598_2023_32446_MOESM8_ESM.pdf]

```
#NEXUS

BEGIN DATA;
      DIMENSIONS  NTAX=52 NCHAR=4952;
      FORMAT DATATYPE = MIXED(DNA:1-4831, Standard:4832-4952) GAP = -
MISSING = ? interleave=yes;
      MATRIX

      Achenomorphus
GGGAGAAGCCCAGCACTGAATCCCGTGGCCGAACCGGGAAATGTAGTGTTTGGGAGGATCCAATATCCACCGT
GCGACGCGTCCAAGTCCTTCTTGAACGGGGCCACATACCCATAGAGGGTGCCAGGCCCGGTAGCTGGAGGATC
TCTCCTTAGAGTCGGGTTGCTTGAGAGTGCAGCCCTAAGTGGGTGGTAAACTCCATCTAAGGCTAAATATGAC
CACGAGACCGATAGCGAACAAGTACCGTGAGGGAAAGTTGAAAAGAACTTTGAAGAGAGAGTTCAATAGTACG
TGAAACCGTTCAGGGGTAAACCTGAGAAACCCGAAAGGTCGAATGGGGAGATTCAGCGTGTCTCGTTTTTGGT
TGCGTGACGATGGTGTTCGCACCGGGCTGCGCCTTCCGAATCCGTAACCGGTGACGAACTCGTGCACTTCTCC
CCTAGTAGGACGTCGCGACCCGTTGGGTGCCGGTCTAAGGCCGACGGTGGAGACTTGGGGTCCCGGCCGGCCC
GCTCGACGGTAAGACGGAGGCGTGGGGTCGCTACGTTAGCGTCCGGCCCGTCACAAGTACGGGCGACTCGGAT
GTCGGACCTGTGTGCCGACCCCGAGATCGCCGACTGTTGGTGACGGTGTCCTCGGACAGACTACACGCCGGTC
GGCGACGCTCTAGCTTTGGGTTTTCAGGACCCGTCTTGAAACACGGACCAAGGAGTCTAGCATGTGCGCGAGT
CATTGGGACCGCATCTAAACCTAATGGCAAAATGAAAGTGAAGGCGTGCCGAGGGAGGATGGGTCGGGGGGCG
TCTCGTTCTCATCGCGAGATGAGGCGCACCCAGAGCGTACACGCGCTTACACCGTATTCGCTGATCTGTTCGA
?CCCCATCATCGAAGACTACCATGGTGGATTCAAGAAGACCGACAAGCACCCCCCCGCGAACTGGGGTGATGT
AAACACCTTCGCTAATCTCGACCCTGCCGGTGAATACGTCGTATCCACCCGCGTCCGTTGCGGCCGCTCCATG
GAGGGCTACCCCTTCAACCCGTGCTTAACCGAGGAGCAGTACAAGGAGATGGAGAGCAAAGTGTCCAGCACTT
TGTCCGGCTTGGAAGGCGAGCTCAAGGGTACTTTCTACCCCTTGACCGGAATGGACAAGGACACCCAGCAGAA
GCTCATCGACGACCACTTCTTGTTCAAGGAGGGCGACCGCTTCCTGCAGACCGCCAACGCCTGCCGCTTCTGG
CCATCCGGCCGTGGTATCTACCACAACGACAACAAGACCTTCTTGGTTTGGTGCAACGAAGAGGACCATCTCC
GCATCATCTCCATGCAGATGGGCGGCGACTTGGGCGAGGTATACCGCCGCCTGGTGACCGCCGTCAACGAGAT
CGAGAAACGTGTACCGTTCTCCCACAATGACAGATTGGGTTTCCTCACCTTCTGCCCAACCAACTTGGGAACT
ACTGTACGTGCCTCTGTACACATCAAAGTACCTAAGCTCGCCGCCAACAAAGCCAAACTCGACGAGGTCGCCG
GCAAATACAACTTGCAGGTACGCGGCACTCGCGGTGTTCCTAAAAAAGTGCTAATAATTGGATCAGGGGGATT
ATCAATAGGCCAAGCAGGAGAATTCGATTATTCGGGCTCGCAAGCCATTAAAGCTYTACACGAAGAAAACATC
CAAACAGTATTGATCAATCCAAACATTGCAACTGTACAAACATCAAAGGGTTTAGCAGACAAAGTTTATTTCT
TACCTCTAGTGCCTGAATTTGTAGAACAAGTCATACGAGTGGAACGTCCTGGCGGCGTATTGTTAACGTTCGG
CGGACAAACAGGGTTAAATTGTGGTGTGGAACTACAAAAGGCTGGTGTATTCGAAAAATACAATGTTAAAATA
TTGGGGACACCAATACAAGCTATTATAGATACAGAAGATAGGAAAATATTTAGTGAAAGGATTGCTATGATTG
GTGAAAAAGTAGCTCCCAGCATGGCTGCTTATTCAGTGCAGGAAGCATTGGAAGCAGCTGAATTGCTGGGGTA
CCCTGTGATGGCTAGAGCAGCGTTTTCTTTAGGTGGATTAGGATCTGGGTTTGCCAACACGGCAGAGGAACTA
AAATTACTCGCTCAACAAGCTTTAGCTCATTCTAATCAGTTAATTATTGATAAGTCTTTGAAAGGATGGAAAG
AAGTGGAATATGAAGTTGTCAGAGATGCATATCCGTACCTTAAGAAAGTTAATGATGATGAACTGAAAGAACC
TACAGATAAAAGAATGTTTGTGCTAGCAGCAGCCTTAAAAAGCGGTTACAGTGTAGACAAATTATATGATTT?
AACAAAAATAGATCGTTGGTTCTTGCAGAAGATGAAAAATATTGTAGATTACAATACACTTCTCGAATCCATG
CAACAAAATAAAATACAAAATACTGCGTACAATTACAAATTATTATTGAAAGCGAAACAAATTGGTTTTAGTG
ATAAACAAATTGCTGTTGCTATTAAAAGCACTGAGCTTGCAGTTAGAAAACAAAGACAAGACTTTGGTATTAC
ACCGTTTGTTAAACAAATTGATACAGTGGCTGCTGAATGGCCAGCAACAACTAATTATCTGTATTTAACGTAC
AATGCAGATAGTCATGATTTAACTTACAATGAAGAACATATTATGGTGATTGGTTCAGGCGTTTATAGAATTG
GTAGCTCTGTTGAATTTGATTGGTGTGCAGTTGGTTGTTTGAGGGAACTTAGAAAATTAAATAAAAAAACAAT
AATGGTTAATTACAATCCTGAAACAGTGAGTACGGATTATGATATGTCAGATAGATTGTATTTTGAGGAAATA
TCATTTGAAGTTGTAATGGATGAAGTCTATATTTTAATTTTACCTGGATTTGGAATAATTTCTCATATTATTT
CTCAAGCTAGAGGAAAAAAAGAAACATTTGGATCATTAGGAATAATTTATGCTATAATAGCTATTGGATTATT
GGGATTTGTAGTTTGAGCTCATCATATATTTACAGTTGGAATAGATGTAGATACACGAGCTTATTTTACCTCT
GCTACTATAATTATTGCGGTTCCTACTGGAATTAAAATTTTTAGATGATTAGCTACTTTACATGGAACACAAA
TTAAATTTACTCCTCCAATATTATGATCATTGGGATTTGTTTTTCTATTTACAATTGGAGGATTAACTGGAGT
TATTCTTGCTAATTCTTCTATTGATATTATTCTTCATGATACTTATTATGTTGTTGCCCATTTTCATTATGTA
TTATCTATAGGAGCAGTATTTGCTATTATAGCTGGTCTAGTACAATGATTTCCCTTATTTACAGGATTAGTAA
TAAATGAATATTTATTAAAAGTTCAATTTTTTGTTATATTTATTGGAGTAAATATAACATTTTTCCCTCAACA
TTTTCTAGGACTAGCAGGATACCTCGACGATATTCTGATTATCCGGATGCATACACTCCATGGAATGTAATT
```

TCTTCAATTGGAAGAATAATTTCAATAATAAGAATTTTTTTATTATTATTTATTATTTGAGAAAGATTTGTTT
CTATACGAATAAATATCTCAGCTAAAAATTTTTCAACTTCAATTGAATGATTACAACTATTTCCCCCAGCTGA
ACATAGATATTCAGAATTACCTATATGAACA?????????????????ATTTGATTTCCTTGGT?AAGGATTC
CATTAGATACTACAATGAAGTACCTGTAGAAAAACGTGTCTTCAAGAACCTTCAATTATTTATGGATAATAAA
TCGCCTGGAGATGATCTGTTCGACAGATTGAACACAGCTGTGATGAACAAACATTTAAACGAGTTAATGGAGG
GTCTCACTGCCAAGGTGTTTCGTACTTATAACGCTTCTTGGACTTTACAGCAACAACTCGATAAGTTGACCAA
TCCTGATGATTCCATATCTGAAAAAATTTTATCATATAACCGTGCCAATAGAGCAGTAGCAATACTTTGTAAC
CATCAACGTGCTGTACCTAAAGGCCACCAAAAGTCCATGGAGAAACTCAAAGAAAAGATCGAAACTAAAAAGG
ACTCCATTAAAGATGCTGAACGACAAGTAAAAGATGCACAGAGAGATGCAAAGCA???TGGCAGCGTAAAGGA
GAAGCAGATTTATGAAAAGAAAAAGAAAATGTTGGAGAGATTAAGAGAGCAATTGGCTAAATTGGAGATCCAA
GAAACTGACCGTGACGAAAATAAAACTATTGCCCTAGGTACGTCCAAACTGAATTATTTGGACCCTAGAATTT
CTGTAGCATGGTGTAAGAAGTTTGGTGTGCCCATTGAAAAAATCTACAAC?????????TGGATGCGGCTGCC
CCCGTTCCGCGTGATCGGCGACCACCTGAAGGACCGCTTCGACGGGGCGTCGCGCGTCATGCTCAGCAACTCG
GCCAGTTCCCGCGG????????????GAACGC???GAACCGTC???CCAAACAGGACAAACTGTCCAACAGCA
TCGCGTCGAACAGCATACACAGCAAGAGGGAGAACAGGCCCAGGAAGTACAAGTACGGGTTCCAGTTGAAACC
GTACAATCCGGACCAYAAGCCGCCGAGTCCCAAGGACTTGGTGTACTTGGAGCCGTTTCCAGGGTTTTGCGAG
AAGAACCCGAAGCTGGGGATCCCGGGCACGCCCGGGTGGCTG?TGCAACGACACGTCGATCGGGGTCGACGGG
TGCGAT?TGATGTGCTGCGGGAAGGGGCTACAGGACCCAGGAGGTCA??????????????????????????
?????????????

Astenus

GGGAGAAGCCCAGCACTTAATCCCGTGGCCGAACCGGGAAATGTAGTGTTTGGGAGGGTCCATCAACCACCGT
GCGACGCGTCCAAGTCCTTCTTGAACGGGGCCACATACCCATAGAGGGTGCCAGGCCCGGTAGCCGGTGGATC
TCTCCTCAGAGTCGGGTTGCTTGAGAGTGCAGCCCTAAGTGGGTGGTAAACTCCATCTAAGGCTAAATATGAC
CACGAGACCGATAGCGAACAAGTACCGTGAGGGAAAGTTGAAAAGAACTTTGAAGAGAGAGTTCAATAGTACG
TGAAACCGTTCAGGGGTAAACCTGAGAAACCCGAAAGGTCGAATGGGGAGATTCAGCTTGTCTCGTTTCTGGT
CGCGTGACGATGGTGCTTGCACCGGACTGCACCTTCCGGATCCGTAACCGGCGGCGAACTCGTGCACTTCTCC
CCTAGTAGGACGTCGCGACCCGTTGGGTGCCGGTCTAAGGCCGACGGTGGAGCCTCGGATTCCCGGCCGGCCC
GCTCGACGGTGTGACAGAGACGTGGGGTCGCCATGTTGGCGTCCGGCCCGTCACAAGTTCGGGCGGCCCGGAT
GTCGGACCTGTGTGCCGACCTCGGGCCCGCCGGCTGTTGGTGGCGGTGTCCTCGGACAGACTACACGTCGGTC
GGCGACGCTCTAGCTTTGGGTTTTCAGGACCCGTCTTGAAACACGGACCAAGGAGTCTAGCATGTGCGCGAGT
CATTGGGACCGCATCTAAACCTAAAGGCGAAATGAAAGTGAAGGCGTGCCGAGGGAGGATGGGTCGGGGGGCG
TCTCGTTCTCATCGCGAGATGAGGCGCACCCAGAGCGTACACGC???TACACCGTATTCGCTGATTTGTTCGA
?TCCCATTATTGAAGACTACCATGGTGGATTCAAGAAGACCGACAAGCATCCCCCGAAGAACTGGGGTGATGT
AAACACTTTCGCCAATCTGGATCCAGCTGGTGAGTTTGTTGTGTCAACCCGTGTCCGTTGCGGCCGTTCAATG
GAGGGTTACCCATTCAACCCATGCTTAACCGAAGAGCAATACAAGGAGATGGAAGCTAAAGTCTCGTCTACTY
TATCTGGACTCGAAGGTGAACTCAAGGGTACTTTCTACCCATTGACTGGAATGGATAAGGATACYCAGCAGAA
GCTCATCGACGATCACTTCTTGTTCAAGGAAGGTGATCGTTTCCTCCAGGCTGCTAACGCCTGCCGTTACTGG
CCATCTGGACGTGGTATCTACCACAACGACAACAAAACCTTCTTGGTCTGGTGCAACGAAGAGGACCATCTTC
GCCTGATTTCGATGCAGATGGGAGGTGATCTTGGTGAAGTCTACCGTCGCCTTGTGAACGCCGTCAACGACAT
CGAGAAGCGCGTTCCCTTCTCCCACAATGACAGATTAGGTTTCCTCACCTTCTGCCCAAGCAACTTGGGCACA
ACTGTACGTGCCTCTGTACACATTAAAGTACCTAAGCTCGCCGCCAACAAGGCTAAGCTC?????????????
???
???
???
???
???
???
???
???
???
????????????????????????????????CCATACCTCAAAGAAGTTGATGATGAAGAATTGCAAGAACC
TACAGATAAGAGAATGTTTGTTTTAGCTGCCGCTTTAAGAAATGGCTACAGCGTAGATAAACTATACGAATT?
GACAAAGATTGATCGTTGGTTCTTACAAAAAATGAAGAACATTGTAGATTATAAYACCCTTTTAGAAACTGT?
????????TAAATTACAAAATTGCAATAATATTTACAAACTTTTAWTGAAAGCRAAACAAATTGGTTTYAGTG
ACAAACAAATTGCTGTTGCTGTTAAAAGTACTGAACTTGCTATTAGAAAACAAAGACAAGATTTTGGTATCAC
TCCTTACGTAAAACAAATAGATACTGTAGCTGCTGAATGGCCAGCTACAACAAATTACCTATATATAACTTAT
AATGCAGATAGTCATGATTTAACTTTCACTGATCAACATACAATGGTTATCGGTTCAGGAGTTTATAGGATTG

GAAGTTCTGTTGAGTTTGATTGGTGTGCTGTTGGTTGTTTAAGGGAGCTCAGAAAACTGAATAAAAAAACAAT
TATGGTTAATTACAATCCCGAAACTGTTAGTACTGATTATGATATGTCTGATCGTCTATACTTTGAAGAAATT
TCATTTGAAGTTGTAATGGATGAAGTTTATATTTAATTTTACCTGGATTTGGTCTTATTTCTCATATTATTA
GACAGGCTAGAGGAAAAAAAGAAACATTCGGATCATTGGGAATAATTTATGCAATAATAGCAATTGGATTATT
AGGATTTGTAGTTTGAGCTCATCACATATTTACAGTTGGGATAGATGTAGACACTCGGGCTTACTTCACCTCA
GCCACAATAATTATTGCTGTGCCTACAGGAATTAAAATTTTCAGATGATTGGCTACATTGCATGGATCACAAA
TTAAATATAATCCATCCATATTATGATCTTTAGGATTTGTATTTTTATTTACAGTAGGAGGATTAACTGGAGT
AATTTTAGCTAATTCGTCTATTGACATTATTTACATGATACATATTATGTAGTTGCCCATTTCCATTATGTT
CTTTCCATAGGAGCAGTATTTGCAATTATAGCTGGATTAGTTCAGTGATTTCCATTATTTACAGGATTAATAA
TAAATGAGTATATTTTAAAAATTCAATTTTTAATTATATTTATTGGAGTAAATATAACATTTTTTCCACAACA
TTTTTTAGGATTAGCCGGAATACCTCGACGTTACTCTGATTATCCGGATGCTTATACCCCATGAAACATAATT
TCATCAATTGGATCATTAATTTCTATAATTAGAATTTTTATTTTGTTATTTATTCTGTGAGAAAGATTTGTAT
CTATGCGAATTAATATTGCTAGAAAAAATTTTTCTACTTCAATTGAATGATTACAATTAACCCCTCCAGCCGA
ACACAGATACTCTGAATTGCCTATAGTAACTAAAGATTATGTAGTTGTATTTGATTTCCTTGGT?AAGGATTC
CATTAGATATTACAATGAAGTACCTGTAGAGAAACGTGTCTTCAAAAACCTTCAGCTGTTCATGGAGAATAAG
TCGTCTGGTGATGATTTGTTTGAYAGATTGAACACRGCTGTAATGAATAAACATTTGAATGAATTGATGGAAG
GTTTAACAGCTAAAGTGTTTCGTACTTATAACGCGTCTTGGACTTTACAGCAACAACTTGATAAATTGACCAA
TCCAGATGATTCCATATCCGAAAAAATATTATCATACAATCGTGCCAATCGAGCTGTAGCTATACTTTGTAAC
CATCAACGTGCAGTGCCTAAAGGCCATCAGAAATCCATGGAGAAACTCAAGGAAAAGATTGAATCKAAAAGGG
AAAACATCAAGGACGCCGAGAGACAAGTTAAAGATGCGCAAAGAGATGCGAAACA???CGGTAGCGTCAAGGA
GAAGCAGATTTACGAGAAGAAAAAGAAAATGCTGGAGAGACTCCGTGAACAATTGGCCAAATTGGAAATTCAG
GAGACGGACCGTGACGAAAATAAAACTATTGCGCTCGGTACCTCCAAGCTGAACTATTTGGACCCGAGAATCT
CGGTTGCTTGGTGTAAGAAGTTTGGTGTGCCCATAGAAAAGATTTATAATAAAACTCAA??????CGCCTGCC
ACCCTTCAGAGTAATAGGCGATCATTTRAAAGACCGCTTCGACGGAGCCTCTAGAGTCATGCTAAGCAACTCG
GCGAGTTCCCGAGG????????????AAACTC???CAACCGGC???CCAAACAAGACAAACTCTCCAACAACA
TCGCCTCGAACAGCATCCACAGCAAAAGAGAGAACCGACCGAGGAAATACAARTACGGCTTCCAACTCAAACC
YTACAATCCCGATCACAAACCTCCGAGCCCYAAAGACCTTGTTTACCTCGAACCRTCGCCCGGWTTCTGCGAG
AAGAACCCCAAGTTAGGGATACAGGGYACTCACGGTAGGTTG?TGCAACGATACATCGATCGGGGTCGACGGG
TGCGATCTGATGTGCTGCGGAAG?GGGCTACAGGACCCAGGAGGTCATCGTTGTCGAGAGGTGCAACTGCACG
?????????????

Astenus_USA

GGGAGAAGCCCAGCACTTAATCCCGTGGCCGAACCGGGAAATGTAGTGTTTGGGAGGGTCCATCAACCACCGT
GCGACGCGTCCAAGTCCTTCTTGAACGGGGCCACATACCCATAGAGGGTGCCAGGCCCGGTAGCCGGTGGATC
TCTCCTCAGAGTCGGGTTGCTTGAGAGTGCAGCCCTAAGTGGGTGGTAAACTCCATCTAAGGCTAAATATGAC
CACGAGACCGATAGCGAACAAGTACCGTGAGGGAAAGTTGAAAAGAACTTTGAAGAGAGAGTTCAATAGTACG
TGAAACCGTTCAGGGGTAAACCTGAGAAACCCGAAAGGTCGAATGGGGAGATTCAGCGTGTCTCGTTTCTGGT
CGCGTGACGATGGTGCTTGCACCGGACTGCACCTTCCGGATCCGTAACCGGCGGCGAACTCGTGCACTTCTCC
CCTAGTAGGACGTCGCGACCCGTTGGGTGCCGGTCTAAGGCCGACGGTGGAGCCTTAGATTCCCGACCGGCCC
GCTCGACGGTGTGACAGAGACGTGGGGTCGCCATGTTGGCGTCCGGCCTGTCACAAGTTCGGGCGGCCCGGAT
GTCGGACCTGTGTGCCGACCTCGGGCCCGCCGGCTGTTGGTGACTGTGTCCTCGGACAGACTACACGTCGGTC
GGCGACGCTCTAGCTTTGGGTTTTCAGGACCCGTCTTGAAACACGGACCAAGGAGTCTAGCATGTGCGCGAGT
CATTGGGACCGCATCTAAACCTAAAGGCGAAATGAAAGTGAAGGCGTGCCGAGGGAGGATGGGTCGGGGGGCG
TCTCGTTCTCATCGCGAGTTGAGGCGCACCCAGAGCGTACACGCTCTTACACCGTATTCGCTGACTTGTTCGA
?CCCCATCATCGAAGACTACCACGGTGGATTCAAGAAGACCGACAAGCATCCCCCGAAGAACTGGGGTGACGT
AAACACTTTTGCCAATTTGGACCCAGCTGGTGAGTTCATTGTGTCAACCCGCGTCCGTTGCGGCCGTTCAATG
GAAGGTTACCCATTCAACCCGTGCTTGACCGAAGAGCAATACAAGGAGATGGAAGCTAAAGTTTCCAGCACCT
TATCAGGGCTTGAAGGTGAACTTAAAGGCACCTTCTATCCATTGACTGGAATGGATAAGGATACCCAACAGAA
GTTGATTGATGATCACTTCTTGTTCAAGGAAGGTGATCGTTTCCTTCAAGCTGCTAATGCTTGCCGCTTCTGG
CCGTCTGGACGTGGTATCTACCACAACGACAACAAAACCTTCTTGGTTTGGTGCAACGAAGAAGATCATCTCC
GTATTATTTCCATGCAGATGGGCGGTGATCTTGGTGAAGTCTACCGTCGTTTGGTGAACGCTGTCAACGACAT
CGAGAAGCGCATTCCGTTCTCTCATAACGACAGATTAGGTTTCTTGACCTTCTGCCCAAGCAACTTGGGCACA
ACTGTACGTGCCTCTGTACACATTAAAGTACCTAAGCTCGCCGCCAACAAGGCTAAGCTGGATGAAATTGCTG
CCAAATACAACTTGCAAGTACGTGGTACCCGT???GTTCCTAAAAAAGTTTTAATTATTGGATCAGGGGGATT
GTCTATTGGCCAAGCTGGTGAATTTGATTATTCCGGTTCACAAGCAATCAAAGCTTTACATGAAGACAATATT
CAAACTGTTTTAATTAATCCTAATATTGCAACAGTTCAAACATCTAAAGGTTTAGCTGATAAAATATACTTTT
TACCATTAGTACCTGAATTTGTTGAACAGGTAATTAGGTCAGAACGACCTGGTGGTGTTTTATTAACGTTTGG
TGGACAAACTGGATTAAATTGTGGAGTAGAATTACAAAAAGCTGGTGTTTTTGAAAAATATGGTGTTAAAATT

TTGGGTACTCCAATACAAGCTATAATAGATACTGAAGATAGAAAGATATTTAGTGAGAGAATCTCACAGATTG
GTGAAAAAGTTGCTCCAAGCATGGCCGCATATTCAGTGCAAGAAGCTTTGGAAGCGGCAGATTTATTAGGATA
TCCCGTTATGGCAAGAGCAGCTTTTTCATTAGGTGGTCTAGGATCTGGCTTTGCCAACACAGCTGAAGAATTG
AAATTACTTGCTCAACAAGCTTTGGCTCATTCAAATCAATTGATTATTGATAAGTCTTTGAAAGGATGGAAAG
AGGTGGAATATGAAGTAGTAAGAGATGCTTATCCATACCTTAAAGAAGTTGATGATGAAGAATTACAAGAGCC
TACAGATAAAAGAATGTTTGTTTTAGCTGCTGCTTTAAGAAATGGCTACAGCGTAGATAAACTATATGACTT?
AACCAAAATTGATCGATGGTTCTTACAGAAAATGAAGAACATTGTAGATTATAATACACTTTTAGAAACAGT?
????????TAAATTACAAAATTGTGATAATGTTTACAAACTTTTGTTGAAAGCAAAACAAATCGGTTTCAGTG
ATAAACAAATTGCTGTTGCTGTTAAAAGTACAGAACTTGCTATTAGAAAACAAAGACAAGATATTGGTATCAC
TCCATATGTAAAACAGATAGATACTGTAGCTGCTGAATGGCCAGCTACAACAAATTATTTATATATAACTTAT
AATGCAGAAAGTCATGATTTAACATTTACCGATCAACACACAATGGTTATTGGTTCAGGAGTTTATAGGATTG
GAAGTTCTGTTGAGTTTGATTGGTGTGCTGTTGGTTGTTTAAGAGAGCTAAGAAAATTGAACAGAAAAACTAT
AATGGTTAATTACA???
?????????????????????GAAGTTTATATTTAATTTTACCTGGTTTTGGAATAATTTCTCACATTATTA
GCCAGGCTAGAGGAAAAAAAGAAACATTTGGAGCCTTAGGAATAATTTATGCCATAATAGCAATTGGTTTATT
AGGGTTTGTAGTTTGAGCCCATCATATATTTACCGTTGGAATAGACGTAGATACTCGAGCCTATTTTACCTCA
GCAACTATAATTATTGCAGTTCCTACAGGAATTAAAATTTTTAGGTGATTAGCCACTCTTCACGGATCACAAA
TCAAGTATAATCCCCCAATACTATGATCTTTAGGTTTTGTGTTTTTATTTACAATCGGAGGATTGACTGGAGT
TATTTTAGCTAATTCATCTATTGATATTATTTTGCATGATACATACTACGTAGTTGCCCATTTTCATTATGTT
CTATCTATAGGAGCAGTATTTGCTATTATAGCGGGATTAGTTCAATGATTCCCACTATTTACAGGTTTAATGA
TAAATGAATTTTTCTTAAAAATTCAATTTTTTGTTATATTTATTGGAGTCAATATAACGTTCTTCCCTCAACA
TTTTTTAGGATTAGCAGGAATACCTCGACGATATTCAGACTATCCTGATGCATATACCCCCTGAAACGTTATC
TCATCAATTGGATCGTTAATTTCAATAATTAGAATTTTTATTCTTTTATTTATTTTATGAGAAAGATTTGTTT
CAATACGAATTAATATTTCAAGAAAAAATTTTGCTACATCAATTGAATGATTCCAGCTAACTCCTCCCGCTGA
ACATAGATAC?????????????????????AAAGATTATGTAGTTGTATTTGATTTCCTTGGT?AAGGATTC
CATTAGATATTACAATGAAGTACCTGTAGAGAAACGTGTCTTCAAAAACCTTCAGCTATTCATGGAAAACAAA
TCACCTGGTGATGATTTGTTCGATAGGTTAAATACAGCTGTAATGAATAAACATTTAAATGAATTGATGGAAG
GTTTAACTGCCAAGGTGTTTCGTACTTATAACGCTTCTTGGACATTACAGCAACAACTTGATAAATTGACCAA
TCCAGATGATTCCATATCCGAAAAAATATTATCATACAATCGTGCCAATAGAGCTGTAGCTATACTCTGTAAC
CATCAACGTGCAGTGCCTAAAGGCCATCAAAAATCCATGGAGAAACTCAAAGAAAAAATCGAAACGAAAAGAG
ATAACATTAAGGACGCGGAGAGACAAGTTAAAGATGCACAAAGGGACGCGAAACA???CGGTAGCGTTAAGGA
GAAGCAGATTTACGAGAAGAAAAAGAAAATGCTGGAGAGACTTCGTGAACAATTAGTTAAATTGGAAATTCAG
GAGACGGACCGCGACGAAAATAAAACAATTGCGCTCGGTACGTCCAAGCTGAACTATTTGGATCCGAGAATCT
CGGTCGCTTGGTGTAAGAAGTTTGGTGTCCCAATTGAAAAGATTTATAATAAAACTCAA??????CGCCTACC
ACCCTTCAGGGTAATAGGTGACCATCTAAAAGACCGCTTCGACGGCGCTTCTAGAGTTATGTTGAGCAACTCC
GCCAGTTCCCGTGG????????????AAATTC???CAACCGCC???CCAAACAAGACAAGCTCTCCAACAACA
TCGCCAGCAACAGCATCCACAGCAAACGCGAAAACCGGCCTAGAAAATACAAGTACGGTTTCCAGCTCAAACC
CTACAACCCAGACCACAAACCCCCCAGCCCTAAAGATCTCGTTTATTTGGAGCCTTCGCCAGGTTTCTGCGAG
AAGAACCCTAAGCTGGGGATTCAGGGTACTCACGGTAGGTTG?TGCAACGATACTTCTATAGGAGTCGACGGG
TGCGATTTG??
?????????????

Cylindroxystus

GGGAGAAGCCCAGCACTGAATCCCGCGGCCTAGCCGGGAAATGTGGTGTTTGGGAGGATCCGTCATCCACCGT
GCGACGAGCCCAAGTCCTTCTTGAACGGGGCCACATACCCATAGAGGGTGCCAGGCCCGGTCGCCGGAGGATC
TCTCCTTAGAGTCGGGTTGCTTGAGAACGCAGCCCCAAGTGGGTGGTAAACTCCATCTAAGGCTAAATATAAC
CACGAGACCGATAGCGAACAAGTACCGTGAGGGAAAGTTGAAAAGAACTTTGAAGAGAGAGTTCAATAGTACG
TGAAACCGTTCAGGGGTAAACCTGAGAAACCCGAAAGGTCGAACGGGGAGATTCAGCGTGTTCAGTCCTCGGT
TGCGAGACGGTGGTGCATGCACCGGTCCGCTCCTTTCTCGCGCCGCGCTTCCGGCGAAC?CGTGCACTTCTCC
CCTTGTAGGACGTCGCGACCCGTTGTGCGCCGATCTACGGCCGACGGTGGAGCCTTGGGCTCCCGACCGGCCC
GCACGACGGTACGAATAAGACGTGAGGCCGCGAAGTTCGCGTCCGGCCCGCCGCAAGCACGCGCGATCCGATC
GTCGGACCAGTGTGCCGACCTCGGACTCGCCGGCTGCTAGCGACGGTGCTCTCGGACAGGCTACACGTCGGTC
GGCGACGCTCTAGCTTTGGGTTTTCAGGACCCGTCTTGAAACACGGACCAAGGAGTCTAGCATGTGCGCGAGT
CATTGGGACCGCATCGAAACCTAAAGGCAAAATGAAAGTGAAGGCGCGCCTAGGGAGGATGGGTCGGGGGGCG
TCTCGTTCTCATTGCGAGATGAGACGCACCCAGAGCGTACACGC?????????????????????????????
????????ATTGAAGATTACCATGGTGGATTTAAGAAGACAGATAAGCATCCACCTAAGAATTGGGGCGACGT
GAACACTTTTGCTAACTTGGACCCAGCTGGTGAATTCGTCGTATCAACTCGCGTCAGATGCGGTCGTTCCATG
GAAGGATATCCTTTCAACCCCTGTTTAACCGAAGAGCAATACAAGGAAATGGAACAAAAAGTATCAACGACTT

TGTCTGGTCTCGAAGGAGAATTAAAGGGTACCTTCTATCCGTTGACTGGAATGGATAAGGATACTCAGCAGAA
ACTGATTGATGATCATTTCTTGTTCAAGGAAGGAGATCGTTTCCTCCAAACCGCTAACGCTTGCCGTTACTGG
CCAAGCGGACGTGGCATCTACCACAACGACAACAAAACGTTCTTGGTCTGGTGCAACGAAGAGGACCATCTCC
GCATTATCTCCATGCAGATGGGCGGTGATCTTGGTGAAGTCTACCGTCGCCTTGTAACAGCCGTCAACGAAAT
CGAAAAGCGCGTACCATTCTCCCACAACGACAGATTAGGTTTCCTCACGTTCTGTCCCACCAACTTGGGCACA
ACTGTTCGCGCCTCTGTTCACATCAAGGTACCAAAGCTCGCCGCCAACAAAGCCAAGCTCGACGAAATCGCCG
GAAAGTACAACTTGCAAGTACGTGGTACACGTGGTGTTCCCAAAAAAGTTTTGATTATTGGGTCAGGCGGTTT
ATCGATCGGACAAGCAGGTGAATTTGATTATTCGGGTTCTCAGGCAATTAAAGCATTGAGAGAAGAGAATATT
CAAACAGTTTTAATTAATCCTAATATAGCAACTGTTCAAACATCTAAAGGTTTATCTGATAAAGTTTATTTTC
TTCCATTAACACCTGAGTATGTAGAGCAAGTTATTAAAGTTGAAAGACCTGGAGGTGTTCTATTAACTTTTGG
AGGACAAACTGGTTTAAATTGCGGTGTTGAGTTGCAACAAGCTGGAGTCTTTGATAAATATGGCGTAAAAATT
TTGGGCACACCAATTGAGGCGATCATCAATACTGAAGATAGAAAAATTTTCAGCGATCGAATTGGAATGATTG
GTGAAAGAGTAGCGCCTAGCATGGCTGCTTATACGGTTCAAGAAGCTTTAGATGCGGCAGAGCAATTAGGATA
TCCTGTAATGGCGCGAGCTGCATTTTCTTTGGGAGGATTAGGATCTGGTTTTGCAGATAATATCGAGGAATTG
AAGATATTAGCTCAACAAGCTTTAGCTCATTCAAGTCAATTGATTATAGATAAATCGTTGAAAGGTTGGAAAG
AAG?????????????????????????????CCTTACATTAAACAAGTTGATGATGAAGAATTAAAAGAACC
TACTGATAAGAGGATGTTTGTTGTTGCTGCTGCGTTAAAACATGGTTATAGTGTGGATAAATTATACGAATT?
AACGAAAATTGATCGATGGTTTTTGCAAAAAATGAAAAATATTACCGATTATATTTCATTTTTAGAAGCAACA
AATCAAAATAAGTTA???????????????ACGCATAATATATTATTGAAAGCTAAACAAATTGGATTTAGTG
ATAAACAAATAGCTGTAGCTGTTGAAAGTACTGAGCTTGCAATTAGGAAATTACGACAAGATTTAAATATTAT
TCCATTTGTTAAACAAATCGATACAGTTGCTGCTGAATGGCCAGCAACGACTAATTATCTATATTTGACTTAT
AACGCCAATAGTCATGATTTAAACTTTAACGAAGAACATATTATGGTCATTGGATCTGGAGTCTATCGAATTG
GTAGTTCAGTTGAGTTTGATTGGGGTGCAGTTGGATGTTTACGAGAGCTTAGAAACTTAAATAAAAAAACGAT
TATGGTT??
?????????????????????????????????CTCATTCTACCTGGATTTGGGTTAATTTCACATATTATTA
TACAATCCAGAGGTAAAAAAGAAACTTTCGGAACTTTAGGAATAATTTATGCTATATTAGCTATTGGTCTATT
AGGATTCATTGTCTGAGCTCATCATATATTTACAGTAGGTATAGATGTAGACACTCGAGCTTATTTTACCTCA
GCAACAATAGTAATTGCTATTCCTACAGGAATCAAAATTTTTAGGTGATTAGCTACATTCCATGGAACTCAAC
TAACATTTAATCCCCCAATACTATGAGCTTTAGGGTTTGTTTTCCTATTTACAATAGGAGGATTAACAGGAGT
AATTTTAGCTAATTCATCAATCGATATTATTTTACATGATACCTATTATGTTGTAGCTCATTTTCATTATGTT
TTATCAATAGGGGCAGTCTTTGCAATTATAGCCGGTATTATTCAATGATTCCCTCTATTTACAGGAACCTATA
TAAATAATTTTTTACTAAAAATTCAATTTTTATTAATATTTATCGGAGTAAATCTTACATTTTTTCCTCAACA
TTTTCTAGGATTAGCAGGGATACCCCGCCGATATTCAGATTATCCTGATGCCTTTACAACTTGAAACGTAATT
TCATCTATTGGTTCATTAATTTCTTTAATTAGAATTTTTTTATTGATATTTATTATTTGAGAAAGATTAACTT
CAATTCGAATTATAATATCTCCAAATAATTTTTCTACATCAATCGAATGACTACAAAAATTTCCACCTTCAGA
ACATAGTTACTCAGAATTGCCTATTATATCA??
???
???
???
???
???
???
???
???
???TGGATGCGGCTGCC
TCCGTTCCGCGT?ATCGGCGACCAGCTGAAGGACCGCTTCGACGGCGCCTCCCGGGTGATGCTGAGCAATTCG
GCGAGCTCGCGGGA??????????AACGGCGC???GAACCGGC???CGAAACAGGACAAGCTCTCCAACAGCA
TCGCCTCCAACAGCATCCACAGCAAGCGCGAGAACAGACCGAGGAAGTACAAGTACGGCTTCCAGCTGAAACC
GTACAATCCGGATCATAAACCTCCGAGCGCTAGGGATCTGGTGTATCTGGAACCGTCGCCGGGTTTTTGCGAG
AAGAACCCGAAATTGGGGATACAGGGGACGCACGGGAGACAG?TGCAACGATACGTCGATCGGCGTCGACGGG
TGCGATCTGATGTGCTGCGGAAG?AGGGTACAGGACGCAGGAGGTCGTCGTCGTCGAGCGCTGCAACTGCACG
TTC??????????

Dibelonetes

GGGAGAAGCCCAGCACTGAATCCCGTGTCCGAGCCGGGAAATGTAGTGTTTGGGAGGGTCCGCCAACCATCGT
ACGACGCGTCCAAGTCCTTCTTGAACGGGGCCACATACCCATAGAGGGTGCCAGGCCCGGTAGCTGGAGGATC
TCTCCTCAGAGTCGGGTTGCTTGAGAGCGCAGCCCTAAGTGGGTGGTAAACTCCATCTAAGGCTAAATATGAC
CACAAGACCGATAGCGAACAAGTACCGTGAGGGAAAGTTGAAAAGAACTTTGAAGAGAGAGTTCAATAGTACG

TGAAACCGTTCAGGGGTAAACCTGAGAAACCCGAAAGGTCGAATGGGGAGATTCAGCGTGTCTCGTTTCTGGT
CGCGTGACGATGGTGCTTGCATCGGACTGCGCCTTCTGGATCCGCAACCGGCGACGAACTCGTGCACTTCTCC
CCTAGTAGGACGTCGCGACCCGTTGGGTGCCGGTCTAAGGTCGACGGAGGAGCCTCAGAGTCCCGGCCGGCCC
GCTCGACGGTAAGACAGAGACGTGGGGTCGCTACGTTAGCGTCCGGCCCGCTACAAGTTCGGGCGACTCGGAT
GTCGGACCTGTGTGCCGACCCCGAGATCGCCGGCTGTTGGTGGCGGTGTCCTCGGACAGACTACACGTCGGTC
GGCGACGCTATAGCTTTGGGTTTTCAGGACCCGTCTTGAAACACGGACCAAGGAGTCTAGCATGTGCGCGAGT
CATTGGGACCGCATCTAAACCTAAAGGCGAAATGAAAGTGAAGGCGTGCCAAGGGAGGATGGGTCGGGGGGCG
TCTCGTTCTCATCGCGAGATGAGGCGCACCCA?????????????CTTACACCGTATTCGCTGATTTGTTCGA
?TCCCATCATCGAGGACTACCATGGTGGCTTCAAGAAGACCGACAAGCATCCACCTGCAAACTGGGGTGATGT
AAATGTCTTCGCCAATCTGGACCCAGCTGGCGAGTACGTGGTGTCAACCCGCGTCCGTTGCGGCCGCTCCATG
GAGGGTTACCCCTTCAACCCTTGCTTAACCGAAGAGCAATACAAAGAGATGGAAGGAAAGGTGTCCACCACCT
TGTCTGGTTTGGAAGGTGAACTCAAGGGTACCTTCTATCCATTGACCGGAATGGATAAGGCTACTCAACAGAA
GCTCATCGATGATCACTTCTTGTTCAAGGAAGGTGATCGTTTCCTCCAGACAGCGAACGCTTGCCGTTACTGG
CCATCTGGACGTGGTATCTACCACAATGATAACAAGACCTTCTTGGTATGGTGCAACGAAGAAGATCATCTCC
GCATCATCTCGATGCAGATGGGTGGTGATCTTGGCGAAGTCTATCGTCGTCTTGTGACTGCCGTCAATGAGAT
CGAGAAGCGCGTTCCATTCTCCCATAATGACAGATTGGGTTTCCTCACCTTCTGCCCAACAAATTTGGGAACA
ACTGTACGTGCCTCTGTACACATCAAAGTACCAAGCTCGCCGCCAACAAGGCCAAGCTT??????????????
???????????????????????????????????GTTCCAAGAAAAGTTTTAATAATTGGTTCAGGCGGTCT
ATCGATTGGACAAGCTGGAGAATTCGATTATTCCGGTTCGCAGGCGATCAAAGCCTTACAAGAAAAAAGTATA
CAAACAGTTTTGATCAACCCGAACATTGCAACTGTGCAAACTTCAAAGGGTTTAGCTGATAAAATCTACTTTT
TACCTTTGGTTCCCGAGTTTGTTGAGCAAGTTATACGATCAGAAAGACCCGGCGGAGTTTTACTGACTTTTGG
AGGGCAGACTGGGTTGAATTGCGGTGTAGAGTTGCAGAAAGCCGGTATATTCGAAAAGTACGGTGTTAAGATA
CTAGGTACACCTATCCAAGCTATAATAGATACTGAAGACCGAAAGATATTTAGCGAAAGGATTGCTATGATTG
GTGAAAAAGTTGCTCCAAGCATGGCTGCTTATTCGGTCCAAGAAGCTTTGGAAGCTGCAGATTTGTTAGGGTA
TCCAGTAATGGCAAGGGCTGCTTTTTCTTTAGGAGGTCTAGGATCTGGCTTTGCAGATACTGCTGAAGAACTC
AAATTACTTGCTCAACAAGCTTTAGCGCACTCGAATCAGTTGATTATCGATAAATCTCTAAAAGGATGGAAGG
AAGTCGAGTATGAGGTTGTAAGAGATGCTTATCCTTACCTCAAAGATGTCGATGATGACGAACTGAAGGAACC
CACTGATAAAAGAATGTTTGTCCTTGCTGCTGCTTTAAGAAGTGGTTACAGCATTGATAAACTCTATGATCT?
GACGAAAATTGATCGTTGGTTCTTGCAGAAAATGAAGAACATCATAGATTACAACACTTTTATAGAATCTGTC
GCTCAAAATAAATTCCAAG???GCACAAACATTTATAAAGTTTTGTTGAAAGCGAAACAAATCGGTTTCAGTG
ATAAACAGATAGCTGTTGCTCTTAAAAGTACTGAACTCGCTATCAGAAAGCAACGGCAAGATTTCGGGATCAC
GCCTTTTGTGAAACAAATAGATACTGTAGCTGCTGAATGGCCTGCAACTACGAATTACTTGTACCTAACGTAC
AATGCTGAAAGTCATGATTTGACATTCAGTGAGCAACACATAATGGTAATTGGATCAGGTGTTTATAGGATAG
GAAGTTCTGTAGAGTTTGATTGGTGCGCTGTTGGATGTTTGAGGGAATTAAGAAAACTAAATAAGAAAACAAT
AATGGTSAATTACAATCCTGAAACTGTGAGCACAGATTACGATATGTCAGATAGATTGTATTTTGAGGAAATA
TCCTTTGAAGTCGTTATGGATGAAGTCTACATTTTAATTTTACCTGGATTTGGATTAATTTCTCACATTATTA
GACAAGCTAGAGGTAAAAAGGAAACTTTTGGTTCATTAGGAATAGTATATGCTATAATAGCTATTGGACTATT
AGGATTCGTAGTTTGAGCTCATCATATATTTACAATTGGAATAGATGTTGATACTCGGGCTTATTTTACTTCA
GCCACAATAATTATTGCAGTTCCTACAGGAATTAAAATTTTTAGATGATTAGCTACTTTACATGGAACTCAAA
TAAAATTTAATCCTTCAATATTATGAGCTTTAGGATTTGTATTTTTATTTACAATCGGAGGTTTAACTGGAGT
TATTTTAGCTAATTCATCAATTGATATTATTTTACATGATACTTATTATGTAGTAGCTCATTTTCATTATGTT
CTTTCTATAGGAGCAGTTTTTGCTATTATAGCTGGATTAATTCAATGATACCCATTATTTACAGGATTAATAA
TAAATGAATATATGTTAAAAATTCAATTTTTTATTATATTTATTGGAGTAAATATAACCTTTTTTCCTCAACA
TTTTTTAGGATTAGCAGGTATACCTCGACGATATTCTGATTATCCAGATGCTTATACACCGTGAAATATTGTA
TCTTCTATTGGATCATTAATTTCAATAACTGCAGTATTTTTATTATTATTTATTATTTGAGAAAGTTTTATTT
CAATACGAATAAATATTTCATCTAATAATCTATCTTCTTCAGTAGAATGATATCAACTATTTCCACCTGCTGA
ACATAGATATTCTGAATTACCTGCTTTAACAAAAGAATACGTAGTAGTATTTGATTTCCTCGGT?AAGGATTC
AATTAGGTACTACAATGAAGTACCTGTAGAGAAACGTGTCTTCAAAAACCTCCAACTGTTTTTGGAAAACAAG
GAACCCGGTGATGATTTGTTTGATAGATTAAATACAGCTGTGATGAACAAACATTTAAACGAGTTGATGGAAG
GTTTAACTGCCAAGGTGTTTCGTACTTATAACGCGTCTTGGACTCTACAGCAGCAACTTGATAAATTGACCAA
TCCAGATGATTCCATATCTGAAAAAATCTTATCGTACAACCGCGCCAACAGAGCGGTAGCTATCCTCTGTAAC
CATCAACGTGCTGTACCGAAAGGTCACCAGAAATCCATGGAGAAACTMAAAGAAAAAATCGAAACTAAAAAGG
ATAACATCAAAGATGCTGAAAGGCAAGTTAAGGATGCGCAGAGGGATGCGAAACA???CGGAAGCGTTAAAGA
GAAGCAGATCTACGACAAGAAGAAGAAGATGTTGGAGAGGCTAAAGGATCAGCTGGCCAAGTTGGAGATTCAG
GAGACGGACCGCGATGAAAATAAAACTATTGCCCTCGGCACGTCCAAGCTGAATTATTTGGATCCTAGGATTT
CCGTTGCATGGTGTAAGAAGTTCGATGTGCCCATTGAAAAAATTTATAACAAAACTCAG??????CGGCTTCC
TCCTTTCCGAGTCATCGGCGAYCACCTGAAGGACCGCTTCGATGGTGCATCYAGRGTGATGCTCAGCAATTCW

GCGAGTTCAAGAGG????????????RAACGC???WAACAGAC???CSAAACAGGATAAGCTWTCAAACAGCA
TCGCGTCCAACAGCATACACAGCAAGAGGGARAACCGACCTCGCAAGTACAAGTACGGTTTYCAGTTGAAACC
TTACAATCCTGATCACAAACCACCAAGTCCTAAGGATCTGGTGTACCTGGARCCTTCACCTGGGTTCTGCGAG
AAGAACCCAAAAYTRGGGATTCAAGGTACTCATGGTAGATTG?TGCAACGACACGTCGATCGGGGTCGACGGG
TGYGACCTCATGTGCTGCGGCAG?GGGGTACAGGACCCAAGAGGTCATCGTCGTCGAGAGGTGCAACTGCACG
?????????????

Diochus

GGGAAGAGCCCAGCACTGAATCCCGCGGACTTGTCGGGAAATGTAGTGTTTGGGAGGGTCCACAATCCGTGGT
GCGGCGCGTCCAAGTCCTTCTTGAACGGGGCCACTTGCCCATAGAGGGTGCCAGGCCCGGCAGCGGGAGGATC
TCTCCTCGGAGTCGGGTTGCTTGAGAGTGCAGCCCTAAGTGGGTGGTAAACTCCATCTAAGGCTAAATATGAC
CACGAGACCGATAGCGAACAAGTACCGTGAGGGAAAGTTGAAAAGAACTTTGAAGAGAGAGTTAAATAGTACG
TGAAACCGTTCAGGGGTAAACCTGAGAAACCCGAAAGGTCGAATGGGGAGATTCAGCGCGTATCGATGTCTGT
TGAGAGACGGTATCGTTCGCG?AGAGCCGCTCCATCGGTCACCGTCGTC??CGACGAACGCGTGCACTTCTCC
CCTAGTAGGACGTCGCGACCCGTTGGGCGTCGGTCAGAGGTCCGCACGGTAGACCGCGCGGCCCGGCCGACAC
GCTCGACGGTACGACAATGGCGCGGGGCCGCGACGTTCGCGTCTGGCCCGTCGCAAGTACGAGCGGTTCGGAT
GTCGGACCTG?GTGCCGACTCTGGACCCGCTGGCTGCTGGCGACGGTGTCCTTGGACAGACCAAGCGCCGGTC
AGCGACGCTATAGCGTTGGGTTTTCAGGACCCGTCTTGAAACACGGACCAAGGAGTCTAGCATGTGCGCGAGT
CATTGGGACT??AGCGAAACCTAAAGGCGAAATGAAAGTGAAGGCTTGCCGAGGGAGGATGGGCGGTGGGGCG
TCTCTAGCTCATTGCGAGCTGAGGCGCACCCAGAGCGTACACGC?????????????????????????????
????????????????????CACGGTGGATTCAAGAAGACGGACAAGCACCCACCTGCTAACTGGGGTGATGT
AAACACCTTCGCCAACTTGGACCCTAATGGCGAGTACGTCGTTTCTACTCGCGTCCGTTGCGGTCGCTCCATG
GAGGGTTACCCATTCAACCCCTGCTTGACCGAGGAACAGTACAAAGAAATGGAACAGAAAGTTTCCACCACTC
TCTCTGGTCTCGAGGGTGAATTGAAGGGTACCTTCTACCCACTTACTGGCATGAGCAAGGAGGTTCAGCAGAA
ACTCATCGACGACCACTTCCTCTTCAAAGAAGGTGATCGCTTCCTCCAGACCGCCAACGCTTGCCGTTACTGG
CCATCTGGACGTGGTATCTACCACAACGACAACAAGACCTTCTTGGTCTGGTGCAACGAGGAGGACCATCTCC
GCATCATCTCCATGCAGATGGGTGGTGATTTGGGCGAAGTCTACCGTCGCCTCGTAACTGCTGTTAACGAGAT
CGAGAAGCGCGTTCCATTCTCCCACAACGATCGTCTTGGTTTCCTCACTTTCTGCCCAACCAACTTGGGCACC
ACTGTTCGTGCCTCCGTGCATATTAAGGTTCCCAAACTTGCCGCCAACAAGGCCAAACTCGACGAAATCGCTG
GTAAGTATAATCTCCAAGTACGTGGTACCCGTGGT??????????????????????????????????????
???
???
???
???
???
???
???
???
???GAATTAAAAGAACC
TACGGATAAACGTATGTTTGTAGTGGCTGCAGCTCTAAGGCACGGTTACACAGTCGATCACCTTTACAACCT?
CACTAAAATTGATAGATGGTTCTTGCAAAAAATGAAGAATATAATAGATTACAACTGTTATCTTGAATCGATT
G??????ATAATTTA????????????????TCACATGGAGCACTTTTGAAAGCGAAACAAATGGGTTTTAGTG
ATAAACAAATAGCAGTTGCCGTTAAAAGCACCGAATTGGCTATTAGAAAACAACGTAAAGATTATGATATTAT
CCCATTTGTTAAACAAATTGATACTGTTGCTGCTGAATGGCCAGCGACTACTAACTATCTTTATTTAACATAT
AACGCTTTAAATCATGATATAAAATTCGTGGAACAACATACAATGGTCATTGGTTCGGGCGTTTATCGTATTG
GAAGTTCAGTAGAATTTGATTGGTGTGCTGTTGGTTGCTTACGTGAATTACGTAAACTGAATCGGAAAACTAT
TATGGTAAACTACAATCCGGAAACTGTTAGTACTGACTACGATATGTCGGATCGTCTTTATTTTGAAGAAATT
TCATTCGAAGTCGTCATGGATGAAGTTTATATTTTAATTCTACCAGGATTTGGAATAATTTCTCATATTATTA
GACAAGAAAGAGGAAAAAAAGAAGCTTTTGGAGCTTTAGGAATAATTTATGCTATAATAGCAATTGGACTATT
AGGATTTATTGTTTGAGCTCACCACATATTTACTGTAGGAATAGATGTAGATACTCGAGCTTATTTTACTTCT
GCTACAATAATTATTGCAGTGCCCACAGGTATTAAAATTTTTAGTTGACTTGCTACATTGCATGGAACTCAAA
TTAATTACTCTCCTTCAATATTATGAGCTTTAGGATTTGTTTTTTTATTCACTGTAGGAGGATTAACAGGAGT
AATTTTAGCTAATTCATCAATTGATATTATTTTACATGATACTTATTATGTAGTAGCCCATTTTCATTATGTA
CTTTCTATAGGAGCAGTTTTTGCAATTATAGCAGGATTAATTCAATGATTCCCTTTATTTACTGGTTTAACTT
TAAATGAAAAATGATTAAAAATTCAATTTCTAACTATATTTATTGGAGTAAATTTAACTTTTTTCCCTCAACA
TTTTTTAGGATTAGCAGGAATACCCCGACGATATTCAGATTATCCAGATGCTTACACCCCTTGAAATATTTTA
TCCTCTATTGGATCATTAATTTCAACTGTTAGAATTTTTACTTTATTATTTATTATTTGAGAAAGATTCACCT
CAATACGAAAAAGAGTAACCCCATTAAGATTATCTACCTCTATTGAATGATATCAATCAATACCCCCTGCTGA

ACATTCATATTCTGAATTGCCTATA???
??
?????????????????TTTGATCGTTTAAATACTGCAATCATGAATAAGCACTTAAACGAATTAATGGAAG
GCCTAACAGCTAAGGTTTTCCGTACATACAACGCTTCATTTACCTTACAGCAACAGTTGGAAAAGTTGACCAA
TCCTGAAGATTCCTTGTCTGAAATATTGCTGTCTTACAATCGTGCAAATAGGGCCGTTGCAATTCTTTGTAAC
CATCAACGTGCTGTTCCTAAAGGTCATCAAAAATCGATGGAAAAATTGAAAGAAAAAAATTGACGCCAAGCGTG
AAGCAATCCGTGATGGAGAGAGACAAGTGAAAGATGCTCAAAAGGATGCTAAGCA???CGGAAGTGTTAAAGA
GAAGACGGTTTATGAAAAGAAGAAGAAAATGTTGCAAAGATTGAAAGAACAATTAACCAAATTAGAGATTCAG
GAGACCGATAGAGATGAGAATAAAACAATCGCTTTGGGAACATCAAAGTTAAATTACTTGGATCCTAAGATAT
CTGTTGCTTGGTGTAAAAAATATGATGTT???
?CCGTTCCGGGTGATCGGCGATAACCTGAAGGACCGTTTCGACGGCGCCTCACGTGTAATGCTCAGCAACACA
GCGAACGCACGTAACACAAACAGTCCGCACGC???GAACAGGC???CGAAGCAGGACAAGCTCAGCAACAGTA
TCTCCTCGAATAGCATCCACAGTAAGAGGGAGAACCGGCCCAAGAAGTACAAGTACGGATTCCAGCTGAAACC
GTACAATCCGGACCACAAGCCGCCCAGTCCGAAGGACTTGGTGTACTTGGAGCCRTCGCCAGGGTTTTGCGAG
AGGAATCCGAAACTAGGTATCCAGGGTACGCACGGGAGGCAA?TGTAACGACACATCGATAGGGGTAGACGGG
TGTGATCTGATGTGCTGCGGTAG?GGGATACAGGAC?CAGGAGGTGGTCGTCGTGGAGAGATGCAACTGCACG
TTCCACTGGTGCT

Domene

GGGAGAAGCCCAGCACTGAATCCCGTGGCCGAACCGGGAAATGTAGTGTTTGGGAGGGTCCGTCATCCATCGT
ACGACGCGTCCAAGTCCTTCTTGAACGGGGCCACATACCCATAGAGGGTGCCAGGCCCGATAGCTGGAGGATC
TCTCCTCAGAGTCGGGTTGCTTGAGAGTGCAGCCCTAAGTGGGTGGTAAACTCCATCTAAGGCTAAATATGAC
CACGAGACCGATAGCGAACAAGTACCGTGAGGGAAAGTTGAAAAGAACTTTGAAGAGAGAGTTCAATAGTACG
TGAAACCGTTCAGGGGTAAACCTGAGAAACCCGAAAGGTCGAATGGGGAGATTCAGCGTGTCTCGTTTTTGGT
CGCGTGACGATGGTGCTCGCCACGGGCCGCGCCTTCCGAATCCGCAACCTGTTACGAACTCGTGCACTTCTCC
CCTAGTAGGACGTCGCGACCCGTTGAGTGTCGGTCTAAGGCCGAGGGTGGAGCCTTGAGGTCCCGGCCGGCTC
GCTCGACGGTAAGACAGAGACGTGGGGTCGCGAAGTTCGCGTCCGGCCCGTTACAAGCTCGGGCGACTCGGAC
GTCGGACCTGTGTGCCGACCTCGAGCGCGCCGGCTGTTGGTAACGGTGTCCTCGGACAGACTACACGTCGGTC
GGCGACGCTTTAGCTTTGGGTTTTCAGGACCCGTCTTGAAACACGGACCAAGGAGTCTAGCATGTGCGCGAGT
CATTGGGACCGCATCTAAACCTAAAGGCGAAATGAAAGTGAAGGCGTGCCGAGGGAGGATGGGTCGGGGGGCG
TCTCGTTCTCATCGCGAGATGAGGCGCACCCAGAGCGTACACGCTCTTACACCGTATTCGCTGATTTGTTCGA
?TCCCATCATTGAAGACTACCATAGTGGATTCAAGAAGACCGACAAGCATCCTCCCTCAAACTGGGGCGATGT
CAACACTTTCGTCAATCTTGACCCTGCTGGTGAATACGTTGTGTCTACTCGCGTACGTTGCGGCCGCTCTATG
GAAGGCTACCCCTTCAACCCCTGCTTAACCGAAGATCAATACAAGGAGATGGAACAGAAGGTTTCGACCACTT
TGTCTGGACTCGAGGGCGAACTTAAGGGTACCTTCTACCCATTAACTGGAATGGATAAGGATACTCAACAGAA
GCTCATCGATGACCATTTCTTGTTCAAGGAAGGAGATCGTTTCCTCCAAACTGCTAACGCTTGCCGTTATTGG
CCATCTGGACGTGGTATCTACCATAACGACAACAAAACATTCTTGGTCTGGTGCAACGAAGAGGATCATCTCC
GTATCATCTCCATGCAGATGGGTGGTGATCTTGGCGAAGTCTACCGTCGTCTTGTCACAGCTGTCAACGAAAT
GGAGAAGCGCGTACCCTTCTCCCACAATGACAGATTAGGTTTCCTTACCTTCTGCCCAACTAACTTGGGTACA
ACTGTACGTGCCTCTGTACACATCAAAGTACCTAAGCTCGCTGCCAACAAGGCTAAGCTCGATGAAATTGCTG
GCAAGTACAACTTGCAAGTACGTGGTACTCGTGGTGTTCCAAGAAAAGTCTTAATTATTGGTTCCGGTGGTTT
ATCAATTGGACAGGCTGGAGAATTTGATTATTCAGGCTCACAAGCAATTAAAGCACTTCAAGAAGTAAACATT
CAAACTGTTTTAATAAATCCCAACATTGCAACCGTACAAACTTCTAAAGGTTTAGCTGACAAAGTTTATTTTC
TTCCTTTAGTACCAGAGTATGTTGAACAAGTAATAAGAGTTGAAAGACCAGGCGGTGTTTTACTAACGTTTGG
TGGACAAACAGGGTTAAATTGTGGAGTTGAACTTCAAAGGGCTGGAATATTCGAAAAATATGGTGTTAAAATT
TTGGGTACACCTATTCAAGCAATTATTGATACAGAAGATAGAAAAATATTTAGTGATAGAATAGCATTAATTG
GAGAAAAGGTAGCACCAAGTATGGCTGCATATTCCGTACAAGAAGCTTTGGAAGCAGCAGAATTGTTAGGATA
TCCTGTTATGGCAAGAGCAGCTTTTTCATTAGGAGGATTAGGTTCTGGATTTGCTAATTCCGCAGAAGAATTA
AAATCGCTAGCTCAGCAAGCATTGGCACATTCCAATCAGTTAATTATTGATAAGTCTTTGAAAGGATGGAAAG
AAGTTGAATACGAAGTAGTGCGAGATGCACTTCACAACATTAAACAAGTTAACGATGAAGAATTAACGGAACC
GACTGATAAGAGAATGTTTGTTGTTGCTGCTGCTTTAAAAAGTGGTTATAGTATTGATAAATTGTACGATTT?
AACAAAAATTGATCGTTGGTTTTTACAAAAAATGAAAAACATTATAGACCATACTACTTTATTAGAATCAACT
GAACAAATTAAATTA???????????????ACAGCTAAAATTTTGTTGAAGGCCAAGAAAATTGGATTTAGTG
ATAAGCAGATTGCAGCAGCATGTAAAAGTACTGAGCTTGCAATCAGAAAACAACGTCAAGATTTTAATATTAC
TCCATGTGTTAAACAGATAGATACTGTAGCTGCTGAATGGCCAGCTACTACAAATTACTTATATTTAACGTAT
AATGCATCATGTCATGATATAACATTTATTGATGAGCATATAATGGTTATTGGATCTGGAGTTTATAGAATTG
GGAGTTCTGTTGAATTTGATTGGTGTGCTGTTGGATGTTTACGAGAGCTTAGAAAATTAAATAAAAAGACAAT
AATGGTAAATTATAATCCAGAAACTGTGAGCACAGATTATGATATGTCAGATAGATTGTACTTTGAGGAAATT

TCATTTGAAGTTGTAATGGATGAAGTATACATTTTAATTTTACCTGGATTTGGATTAATTTCTCACATTATTA
GACAAGCTAGAGGAAAAAAAGAAACATTTGGAACTCTAGGAATAGTTTATGCAATAATAGCAATTGGGCTATT
AGGATTTATAGTATGAGCTCATCACATATTCACTGTTGGAATAGATGTGGATACTCGAGCTTATTTTACTTCA
GCAACCATAGTTATTGCTATCCCTACTGGAATTAAAATTTTTAGCTGATTAGCAACATTACATGGAACAAAAA
TTATTTTTAATCCCCCAATAATTTGGGCATTAGGATTCGTATTCTTATTTACAATTGGTGGACTAACTGGAGT
AATTCTAGCAAATTCATCAATTGATATTGTATTACACGACACTTATTATGTTGTAGCACATTTCCATTACGTA
CTTTCTATAGGAGCTGTGTTTGCAATTATAGCTGGTTTAGTTCAATGATTTCCATTATTTACTGGACTAACCT
TAAACAAAAAATGATTAAAAATTCAATTTTTCATCATATTTACTGGAGTAAACTTAACCTTTTTCCCACAACA
TTTTCTAGGAATAGCAGGTATACCTCGACGATACTCAGATTACCCTGATGCCTATACTACCTGAAATGTAATC
TCATCAATTGGATCGTTAATCTCAATAATAGGGGTTTTTTTATTGCTATTCATTTTATGAGAAAGATTTTCTG
CAAAACGAATAATTTTGTCAGCAAAAAATTTTGTGACTTCAATTGAATGATATCAACTTTACCCACCAGCAGA
ACATAGATATAACGAACTCCCAATATTAGTAAAAGACTACGTAGTCGTTTTTGATTTTCTCGGT?AAAGATTC
CATTAGATATTACAATGAAGTACCTGTGGAGAAACGTGTATTCAAAAACCTCCAGTTGTTCATGGAAAATAAA
GCGAAAGCGGACGATTTATTTGATCGCTTAAACACAGCTGTTATGAACAAGCATTTAAACGAGCTCATGGAAG
GTTTAACCGCCAAGGTATTTCGTACTTACAATGCTTCTTGGACGTTGCAACAACAACTCGAGAAGCTGACCAA
TGAAGACGATTCCATATCTGAGAAAATTTTATCATATAACCGTGCCAATAGGGCTGTCGCCATTCTGTGTAAC
CATCAACGTGCTGTTCCTAAAGGCCATCAGAAATCTATGGAGAAGCTAAAGGAGAAAATCGATTCTAAGCGAG
ATAACATCAAGGATGCAGAGCGGCAGGTTAAGGATGCACAGAAGGACGCTAAGCA???CGGTAGTGTAAAGGA
GAAGATGGTTTACGACAAGAAAAAGAAGATGTTGGAGAGGCTGCGCGATCAATTAGCGAAATTGGAGATTCAA
GAGACGGATCGTGACGAGAATAAAACTATTGCGCTTGGTACGTCTAAGTTGAACTATTTGGATCCGAGAATTT
CGGTCGCCTGGTGTAAGAAGTACGATGTGCCCCTTGAGAAAATTTA?????????????TGGATGCGGCTTCC
GCCTTTCAGAGTAATCGGAGATCACCTAAAGGACCGTTTCGACGGTGCTTCACGTGTGATGCTCAGCAACTCT
GCTAGTTCCAGAAG?????????????????????CAATCGTC???CCAAGCAGGACAAGCTTAGCAACAACA
TAGCATCCAACAGCATACATAGCAAGCGCGAGAATCGTCCGCGAAAATATAAATATGGCTTCCAACTGAAACC
TTACAATCCCGATCATAAGCCTCCCAGTCCTAAAGACTTGGTGTATTTGGAGCCGTCGCCTGGTTTCTGCGAG
AAGAACCCGAAGCTTGGCATACAGGGTACGCATAGTARGCGA?TGCAACGATACTTCTATAGGTGTCGATGGT
TGCGATTTGATGTGTTGCGGAAG?AGGTTACAGGACCCAGGAAGTCATTGTTGTGGAGAGGTGTAACTGCACG
TTCCACTGGTGCT

Dysanabatium

GGGAGGAGCCCAGCACAGAATCCCGTGGCCGAACCGGGAAATGTTGTGTTTGGGAGGGTCCGTCATCCATTGT
GCGACGCGTCCAAGTCCTTCTTGAACGGGGCCACATACCCATAGAGGGTGCCAGGCCCGATAGCCGGAGGATC
TCTCCTCAGAGTCGGGTTGCTTGAGAGTGCAGCCCTAAGTGGGTGGTAAACTCCATCTAAGGCTAAATACAAC
CACGAAACCGATAGCGAACAAGTACCGTGAGGGAAAGTTGAAAAGAACTTTGAAGAGAGAGTTCAATAGTACG
TGAAACCGTTCAGGGGTAAACCTGAGAAACCCGAAAGGTCGAATGGGGAGATTCAGCGTGTCACGTTATCGGC
CGAGTGACGATGGTGTTCGCACCGGATTGCACCGACCGACTCCTAAACCGGCGGCGAACTCGTGCACTTCTCC
CCTAGTAGGACGTCGCGACCCGTTGGGTGCCGGTCTAAGGCCAGCGGTGGAGCCTGTGGGTCCCGGCCGGCTC
GCCCGACGGTATGACAGAGACGTGTGGTCGCGAAGTTCGCGTCCGGCCCGCCACAAGCACGCGCGACTCGAAC
GCCGGACCTGTGTGCCGACCTCGAGCTCGCCGGCTGTTGGTGGCGGTGTCCTCGGACAGACCACACGTCGGTC
GGCGACGCTTTAGCTTTGGGTTTTCAGGACCCGTCTTGAAACACGGACCAAGGAGTCTAGCATGTGCGCGAGT
CATTGGGATCATATCTAAACCTAAAGGCGAAATGAAAGTGAAGGCGTGCCTAGGGAGGACGGTCGGGGGGCG
TCTCGTTCTCATCGCGAGATGAGGCGCACCTTGAGCGTACACGC??????????????????????????????
?????ATCATCGAGGATTACCATGGCGGGTTCAAGAAGACCGACAGCCACCCGCCCAAGAACTGGGGTGACGT
GAACACCTTCGCCAACCTCGACCCGGCCGGTGAGTACGTCGTCTCGACCCGCGTCCGTTGCGGCCGCTCCATG
GAGGGCTACCCGTTCAACCCGTGCTTGACCGAGGAGCAGTACAAGGAGATGGAGCAGAAGGTCTCGTCCACCC
TGTCCGGCATGGAGGGCGACCTCAAGGGCACCTTCTACCCGCTGACCGGCATGGACAAGGATACCCAGCAGAA
GCTCATCGACGACCACTTCCTGTTCAAGGAGGGCGATCGTTTCCTGCAGGCGGCCAACGCGTGCCGTTTCTGG
CCGTCCGGTCGCGGCATCTATCACAACGAGAACAAGACCTTCCTGGTGTGGTGCAACGAGGAGGACCATCTCC
GCATCATCTCCATGCAGATGGGCGGCGATCTCGGCGAGGTATACCGTCGCCTCGTCAGCGCCGTCAACGAGAT
CGAGAAGCGCGTGCCGTTCTCGCACAACGACAGGTTGGGTTTCCTCACCTTCTGCCCATCCAACTTGGGCACC
ACTGTACGTGCCTCTGTACACATCAAAGTACCTAAACTTGCCGCCAACAAAGCCAAGCTTGATGAAATCGCTG
CCAAATACAACTTGCAAGTACGCGGTACACGCGGTGTTCCGAAAAAAGTTTAATTATAGGCTCAGGTGGTTT
GTCAATCGGACAGGCGGGTGAATTTGATTATTCTGGTTCGCAGGCAATCAAAGCATTGCAAGAAGAGAATATT
CAAACGGTTTTAATCAACCCGAACATTGCGACTGTACAAACATCGAAAGGTTTAGCCGATAAAGTTTATTTTT
TACCGTTGGTGCCGGAATATGTGGAGCAAGTAATTAGAGTTGAACGACCTGGCGGTGTGTTATTAACATTTGG
CGGCCAAACAGGATTGAATTGCGGAGTGGAACTTCAGAAAGCTGGAATTTTTGAAAAATACGATGTTAAAGTT
CTCGGGACACCAATACAAGGTATTATCGATACTGAAGATCGGAAAATTTTTAGTGAAAGAATTTCAATAATCG
GTGAAAAGGTAGCCCCAAGTATGGCTGCTTATTCTGTTCAGGAAGCTTTGGAAGCGGCAGAGATGTTGGGATA

TCCAGTAATGGCCAGAGCTGCTTTCTCGTTAGGTGGATTGGGATCTGGTTTCGCAAATACAGCAGATGAACTT
AAATCATTAGCTCAACAGGCGTTAGCGCATTCAAGTCAATTAATTATCGACAAATCTTTAAGAGGTTGGAAGG
AAGTCGAATATGAAGTCGTAAGGGATGCTTATCCGTATCTTAAAACTGTAAATGATGAAGAATTAGAAGAACC
GACGGATAAACGAATGTTTGTAGTAGCCGCTGCTTTAAGAAATGGTTATTCCGTGGAAAAAATATATGAATT?
AACCAAAATTGATCATTGGTTTTTGCAAAAAATGAAAAACATAATAGATTACAACACTTATTTAGAATCAATT
CAACAAAATAAATTA???????????????ACCTATAAAATCTTATTAAAGGCGAAACAAATAGGATTTAGTG
ATAAACAGATTGCTGTTGCCGTTAAAAGCACTGAACTCGCTATTAGAAAACAACGGCAGGATTTTAATATCAT
ACCTTTCGTTAAACAAATTGATACAGTTGCTGCAGAGTGGCCTGCGACTACGAATTATTTGTATTTAACGTAC
AACGCAGCAAGTCATGATTTAACTTTCTCCGAAGAACATATTATGGTTATAGGTTCCGGTGTTTATAGAATAG
GAAGTTCCGTTGAATTTGATTGGTGTGCTGTCGGTTGTTTACGAGAATTAAGAAAATTAAATAAAAAGACAAT
AATGGTAAATTACAATCCGGAAACGGTTAGTACAGATTATGACATGTCTGACAGATTATATTTCGAAGAAATA
TCATTTGAAGTTGTAATGGATGAAGTTTACATTTTAATTTTACCAGGATTTGGAATAATTTCTCATATTGTAA
GACAAAATAGAGGTAAAAAGGAAACATTTGGGACATTAGGAATAATTTATGCTATAATAGCAATTGGTTTATT
AGGATTTGTTGTTTGAGCACATCATATATTTACTATTGGTATAGATGTTGATACTCGAGCTTATTTTACATCT
GCTACAATAATTATTGCAGTACCAACTGGTATTAAAATTTTTAGATGATTAGCTACTTTACATGGAACTCAAA
TTAATTTTAATCCAGCAATATTATGATCATTAGGATTTATTTTTTTATTTACTATTGGTGGATTAACAGGAGT
AATTTTAGCAAATTCATCTATCGATATTATTTTACATGATACTTATTATGTAGTAGCTCATTTTCATTATGTT
CTTTCTATAGGAGCTGTATTTGCAATTATAGCAGGATTAGTTCAATGATTTTCTTTATTTACAGGAATTACAT
TAAATAGTTATTTATTAAAAATTCAATTTTTTGTTATATTTACAGGTGTAAATTTAACTTTTTTCCCTCAACA
TTTTTTAGGATTAGCTGGTATACCTCGACGTTATTCTGACTATCCAGATGCCTATACTACTTGAAATGTAGTA
TCTTCTATTGGATCTTTTATTTCTTTAGTAAGAATTTTATTATTACTTTTTATTATTTGAGAAAGATTTACTT
CAATTCGTATAGTAATTTCAGCCAAAAATTATTCATCTTCTATTGAATGATATCAATTATACCCTCCTGCTGA
ACATAGATATTCAGAATTACCTGCAATATCT???
???
???
???
???
?????????????????????????????????TCAATGGAAAAATTAAAGGAGAAGATTGAAGCTAAAAAGG
AATCCATCAAAGACGGTGAGCGTCAAGTTAAAGATGCTCAAAAGGATGCTAAACA???TGGTAGTGTTAAGGA
GAAACAGATCTATGATAAGAAAAAGAAAATGTTGGAAAGACTAAAGGAACAGTTAGCAAAATTAGAAATTCAA
GAAACTGACCGTGATGAAAATAAAACAATTGCTCTTGGCACTTCCAAGTTGAATTATCTAGATCCGAGAATCT
CTGTGGCTTGGTGTAAGAAATATGGAGTACCCATTGA?????????????????????????ATGCGCTTGCC
TCCATTCAGGGTAATCGGCGATCATCTAAAGGACCGCTTCGACGGCGCTTCCCGCGTCATGCTCAGCAATTCG
GCGAGCTCCAGGGGC?????????GGTAACTC???GAACCGCC???CCAAGCAGGACAAAATGTCGAACAACA
TCGCTTCGAACAGTATCCACAGCAAGCGGGAGAACAGGCCGCGCAAGTACAAGTACGGCTTCCAGCTGAAACC
GTACAACCCCGACCACAAGCCGCCCAGTCCGAAGGACTTGGTGTACCTCGAGCCGTCGCCCGGTTTCTGCGAG
AAAAATCCGAAGCTCGGCATCCAGGGCACGCACGGGAGGCAG?TGCAACGATACGTCGATAGGTGTAGACGGC
TGCGACCTGATGTGCTGCGGAAG?AGGTTACAGGACCCAGGAAGTCATTGTGGTGGAGAGGTGCAACTGCACT
TTCC?????????

Echiaster

GGGAGAAGCCCAGCACTGAATCCCGTGGTCGAACCGGGAAATGTAGTGTTTGGGAGGGTCCATTATCTATCGT
GCGACGCGTCCAAGTCCTTCTTGAACGGGGCCACATACCCATAGAGGGTGCCAGGCCCGATAGCTGGAGGATC
TCTCCTTAGAGTCGGGTTGCTTGAGAGTGCAGCCCTAAGTGGGTGGTAAACTCCATCTAAGGCTAAATATGAC
CACGAGACCGATAGCGAACAAGTACCGTGAGGGAAAGTTGAAAAGAACTTTGAAGAGAGAGTTCAATAGTACG
TGAAACCGTTCAGGGGTAAACCTGAGAAACCCGAAAGGTCGAATGGGGAGATTCAGCGTGTCTCGTTTCTGGT
CGCGTGACGAAGGTGCTTGCACCGGCCTGCGCCTTCCGGATCCGTAACCGGCGGCGAACTCGTGCACTTCTCC
CCTAGTAGGACGTCGCGACCCGTTGGGTGCCGGTCTAAGGCCGACGGTGGAGCCTCGGGGTCCCGACCGGCCC
GCTCGACGGTAAGACAGAGACGTGGGGTCGCTTCGTAAGCGTCCGGCCCGTCACAAGTCCGTGCGCCTCGGAC
GTCGGACCTGTGTGCCGACCCCGAGCTCGCCGGCTGCTGGTGACGGTGTCCTCGGACAGACTACACGTCGGTC
GGCGACGCTTTAGCTTTGGGTTTTCAGGACCCGTCTTGAAACACGGACCAAGGAGTCTAGCATGTGCGCGAGT
CATTGGGACCGCATCTAAACCTAAAGGCAAAATGAAAGTGAAGGCGTGCCGAGGGAGGATGGGTCGGGGGGCG
TCTCGTTCTCATCGCGAGATGAGGCGCACCCAGAGCGTACACGCTCTTACACCGTATTTGCCGACTTGTTCGA
?TCCCATCATTGAGGATTACCATGGTGGATTCAAGAAGTCCGACAAGCACCCACCTAAGAACTGGGGTGATGT
AAACACTTTCGGCAACCTCGACCCAGCTGGTGAATATGTAGTATCCACCCGTGTACGTTGCGGCCGCTCCATG
GAAGGTTACCCGTTCAATCCGTGCTTGACCGAAGAACAATACAAAGAAATGGAAGGTAAAGTGTCCAGCACCT
TGTCTGGTCTCGAAGGAGAACTCAAGGGTACTTTCTATCCGTTGACCGGTATGGACAAAGCGACCCAACAGAA
GCTCATCGATGATCACTTYTTATTCAAGGAAGGCGACCGTTTCCTTCAGGCTGCGAACGCCTGCCGCTTCTGG

CCATCTGGACGTGGAATCTACCACAACGACAACAAAACCTTCTTGGTCTGGTGCAACGAAGAAGATCATCTCC
GCATTATCTCCATGCAGATGGGCGGCGACCTTGGCGAGGTCTACCGTCGCCTTGTGACCGCCGTCAACGAAAT
TGAGAAGCGCGTTCCCTTTTCGCACAATGACAGGTTAGGTTTTCTTACCTTCTGCCCAACCAACTTGGGCACA
ACTGTACGTGCCTCTGTACACATTAAAGTACCTAAGCTCGCAGCCAATAAGGCCAAACTTGACGAAGTCGCTG
CCAAATATAACTTGCAAGTACGCGGTACCCGCGGTATTCCTAAAAAAGTTTTAATTATCGGTTCCGGAGGTTT
GTCTATAGGACAAGCCGGGGAATTCGATTATTCAGGTTCACAAGCAATAAAAGCTTTACAAGAAGAAAACATA
CAAACAGTCCTGATTAACCCCAATATAGCAACTGTACAAACATCAAAAGGTTTAGCTGATAAAATTTACTTTC
TGCCATTGGTGCCTGAATTCGTTGAACAAGTGATAAGAGCAGAACGTCCTGGCGGTGTTTTGCTGACATTTGG
TGGACAAACAGGGTTGAATTGCGGTGTGGAACTACAAAGAGCAAATGTATTTGAAAAGTAYGGTGTTAAAATT
TTGGGTACACCTATACAGGCTATAATTGATACAGAAGACAGAAAAATTTTTAGTGAAAGAATTGCTTTGATTG
GAGAAAAAGTTGCGCCAAGTATGGCTGCATATTCTGTTCAGGAAGCACTGGAAGCAGCGGAATTGTTAGGATA
TCCTGTTATGGCAAGAGCTGCGTTTTCTTTAGGTGGTTTAGGATCAGGGTTTGCTAATAACGATGAAGAACTG
AAAGCACTTGCTCAACAAGCTTTAGCTCATTCGAATCAATTAATTATTGATAAGTCCTTGAAAGGATGGAAAG
AAGTTGAATATGAAGTTGTACGTGATGCTAAT???
???
???
???
???
???
???
???
???
??????????????????????GAAGTTTATATTTAATTTACCCGGGTTTGGTTTAATTTCCCATATTTTTA
GACAATCTAGAGGAAAAAAAGAAACTTTTGGTTCATTAGGAATAATTTATGCAATAATAGCTATCGGGTTACT
AGGGTTTATTGTATGGGCCCATCATATATTTACTGTCGGGATAGATGTTGATACCCGGGCCTACTTTACTTCA
GCAACCATAATTATTGCAGTACCTACAGGAATTAAAATTTTTAGTTGATTGGCTACATTTCATGGGACTCAAA
TTAAATTTGATCCTCCAATATTATGAGCTTTAGGATTTGTATTTTTATTTACTTTAGGGGGATTAACTGGAGT
TATTTTAGCTAATTYTTCTATTGATATTGTACTTCATGATACCTATTATGTTGTAGCTCATTTTCATTATGTA
TTATCAATAGGGGCTGTATTTGCAATTATAGGAGGATTAGTTCAATGATTCCCTTTATTTACAGGATTAGCAT
TAAACAATTATTGATTAAAAATTCAATTTTTAATTATATTTATTGGAGTGAATTTAACATTTTTTCCTCAACA
TTTTCTTGGATTAGCAGGAATACCTCGCCGATATTCAGATTATCCTGATTYTTATATACCTTGAAATACTATT
TCCTCTATTGGATCAATTATTTCTATAGTAAGAATTTTTTTTTTATTATTTATTATTTGAGAAAGATTCATTA
CTATGCGAATAAATATTATACCAAAAAATTTTTCTACATCTATTGAATGACTCCAATTAACTCCACCTGCTGA
ACACAGATWTTCTGAGTTACCAATACTAACT???????????????????????????CTTGGT?AAGGATTC
TATTAGATATTATAATGAAGTACCTGTGGAAAAACGTGTCTTCAAAAACCTTCAATTGTTCATGGAAAACAAA
TCTCCAGGTGATGATTTATTTGATAGATTGAACACAGCAGTAATGAACAAACATTTAAACGAGTTAATGGAAG
GTTTAACTGCCAAGGTATTTCGTACTTATAACGCTTCTTGGACTCTACAACAACAACTAGATAAATTAACCAA
TCCAAATGATTCCATATCAGAGAAAATATTATCATACAATCGAGCAAATAGAGCAGTAGCTATACTTTGTAAC
CATCAACGTGCAGTACCTAAAGGTCACCAAAAATCCATGGAAAAACTTAAAGAAAAAATTGATGCCAAAAAAG
AAGCTATTAGAGATGGTGAACGACAAGTTAAAGATGCACAAAAAGATGCGAAACG???TGGAAGTGTCAAGGA
GAAACAAATTTATGACAAGAAGAAGAAAATGTTGGACAGACTCAAAGAACAGTTAGCAAAATTGGAAATCCAG
GAGACAGACCGTGACGAAAATAAAACTATTGCCCTCGGTACTTCCAAGCTGAACTATTTGGATCCCAGAATTT
CGGTTGCTTGGTGCAAGAAGTTTGATGTGCCCATTGAAAAAATCTA?????????????GGGATGCGCTTGCC
ACCCTTCCGAGTGATCGGCGACCATTTGAAAGACCGCTTCGACGGCGCCTCCAGGGTGATGCTGAGCAATTCG
GCGAGTTCTCGCGG????????????GAACGC???CAACCGGC???CCAAGCAGGACAAACTCTCCAACAGCA
TCGCGTCGAACAGCATCCACAGCAAAAGGGAGAACCGCCCCCGCAAGTACAAGTACGGCTTCCAATTGAAGCC
GTACAATCCGGACCACAAGCCTCCGAGTCCGAAAGATCTGGTGTACCTGGAGCCGTCGCCTGGATTCTGCGAG
AAGAACCCGAAGCTTGGGATACAGGGCACGCATGGTAGATTG?TGCAACGATACTTCTATAGGTGTGGACGGT
TGCGATTTGATGTGCTGCGGTAG?GGGCTACAGGACGCAGGAAGTCATCGTGGTAGAAAGGTGCAACTGCACG
TTCC?????????

Enallagium

GGGAGAAGCCCAGCACTGAATCCCGTGGCCGAACCGGGAAATGTAGTGTTTGGGAGGGTCCGTTATCCATCGT
GCGACGCGTCCAAGTCCTTCTTGAACGGGGCCATATTCCCATAGAGGGTGCCAGGCCCGATAGCTGGCGGATC
TCTCCTCAGAGTCGGGTTGCTTGAGAGTGCAGCCCTAAGTGGGTGGTAAACTCCATCTAAGGCTAAATATGAC
CACGAGACCGATAGCGAACAAGTACCGTGAGGGAAAGTTGAAAAGAACTTTGAAGAGAGAGTTCAATAGTACG
TGAAACCGTTCAGGGGTAAACCTGAGAAACCCGAAAGGTCGAATGGGGAGATTCAGCGTGTCTCGTGTTCGGT
CGCGTGACGATGGTGCTTGCACCGGGCTGCGCCTTCTGAATCCGTAACCGGCGACGAACTCGTGCACTTCTCC

CCTAGTAGGACGTTGCGACCCGTTGGGCGCCGGTCTACGGCCGACGGTGGAGCCTTGGGGTCCCGGCCGGCCC
GCTCGACGGTAAGACAGAGACGTGGGGTCGCGATGTTCGCGTCCGGCCCGTCACAAGTACGGGCGACTCGGAC
GTCGGACCTGTGTGCCGACCTCGAGCTCGCCGGCTGTTGGTGACGGTGTCCTCGGACAGACTACACGTCGGTC
GGCGACGCTTTAGCTTTGGGTTTTCAGGACCCGTCTTGAAACACGGACCAAGGAGTCTAGCATGTGCGCGAGT
CATTGGGACCGCATCTAAACCTAAAGGCGAAATGAAAGTGAAGGCGTGCCGAGGGAGGATGGGTCGGGGGGCG
TCTCGTTCTCATCGCGAGATGAGGCGCACCCAGAGCGTACACGC?????????????????????????????
????????????????????CATGGTGGATTCAAGAAGACCGACAAGCACCCCCCTTCCAACTGGGGAGATGT
CAACACTTTCGTCAACCTTGACCCTGCTGGCGAATACGTTGTATCTACTCGCGTACGTTGCGGCCGCTCCATG
GAGGGCTACCCMTTCAACCCCTGCTTAACCGAAGATCAATACAAGGAGATGGAACAGAAGGTTTCCACCACTT
TGTCTGGACTCGAGGGTGAACTTAAGGGTACCTTCTACCCATTGACCGGAATGGATAAGGACACCCAACAGAA
GCTCATCGATGACCATTTCTTGTTCAAGGAAGGAGATCGTTTCCTCCAAACTGCTAACGCTTGCCGTTATTGG
CCGTCTGGACGTGGTATCTACCATAACGACAACAAAACATTCTTGGTCTGGTGCAACGAAGAGGATCATCTCC
GCATCATCTCCATGCAGATGGGTGGTGATCTTGGTGAAGTCTACCGTCGCCTTGTCACAGCCGTCAACGAAAT
TGAGAAGCGCGTACCCTTCTCTCACAATGACAGATTAGGTTTCCTTACCTTCTGCCCAACTAACTTAGGTACA
ACTGTACGTGCCTCTGTACACATCAAAGTACCTAAGCTCGCTGCCAACAAGGCTAAGCTTGATGAGATTGCTG
GAAAGTACAACTTGCAAGTCCGTGGTACTCGTGGTGCTCCTAAAAAAGTTTAATTATTGGTTCTGGTGGTTT
ATCGATTGGTCAAGCTGGAGAATTTGATTACTCTGGTTCACAAGCTATTAAAGCTTTACACGAAGAAAATATT
CAAACTGTATTAATAAATCCTAATATCGCCACAGTGCAAACATCAAAAGGTTTAGCTGATAAAATATATTTCT
TACCGTTAGTGCCTGAATTCGTTGAACAAGTAATTAGAGTTGAAAGACCCGGTGGTGTTTTATTAACTTTTGG
TGGCCAAACAGGTTTGAATTGTGGTGTAGAATTACAAAAAGCTGGTGTTTTTGAAAAATATGGTGTAAAAATT
TTGGGTACTCCAATTCAGGCCATAATTGACACTGAAGACCGGAAAGTTTTCAGTGATAGAATAGCACAAATTG
GTGAGAAAGTTGCTCCAAGTATGGCTGCTTATTCCGTACAGGAAGCTTTGGAAGCTGCTGAATTATTAGGTTA
CCCAGTAATGGCAAGAGCTGCATTCTCCTTAGGTGGTTTGGGATCTGGATTTGCTGATACTGCCGATGAATTG
AAATCACTTGCTCAACAGGCTTTGGCTCATTCTAATCAATTAATTATAGATAAATCATTGAAAGGTTGGAAGG
AAGTTGAATATGAAGTTGTTAGAGATGCGTATCCTTACTTAAAACAAGTAAATGATGAAGAGTTAAAAGAACC
CACTGATAAACGGATGTTTGTACTGGCAGCTGCTTTAAGAAATGGTTATACTATTGATAAACTTTATGATTT?
AACAAAAATAGACCGCTGGTTTTTACAAAAAATGAAAAATATTGTTGATTTCAATACCCATCTAGAATCAATT
???CAAAATAAGTTA???????????????ACGTATAAACACTTATTGAAAGCGAAACAAATTGGTTTCAGTG
ATAAACAAATTGCTGTTGCAGTAAAAAGTACTGAACTTGCTGTTAGAAAACACCGTCAAGATTTTGAAATTAC
TCCATATGTTAAGCAAATAGATACTGTAGCAGCTGAATGGCCAGCAACAACAAATTATCTATATTTAACTTAT
AATGCTGAAAGTCATGATATAACTTTTTCCGATGAGCATATTATGGTAATTGGATCAGGTGTATATAGAATTG
GAAGTTCAGTGGAATTCGATTGGTGTGCAGTAGGTTGTTTACGAGAACTTAGAAGGTTAAATAAAAAAACAAT
AATGGTCAATTACAATCCAGAAACAGTTAGTACTGACTACGATATGTCTGATAGATTATATTTCGAAGAAATT
TCATTTGAAGTTGTAATGGAT??
??????????????AAAAAAGGAAACATTGGGGCTTTAGGAATAATTTATGCAATAATAGCAATTGGCTTGTT
AGGATTCGTTGTATGAGCTCATCATATATTTACAGTAGGAATAGATGTTGACACTCGTGCCTATTTTACATCA
GCAACTATAATTATTGCTGTTCCAACAGGAATCAAAATTTTTAGATGATTAGCTACTCTTCATGGAACTCAAA
TTAAATACACCCCCCCTATATTATGGGCTTTAGGATTTGTATTTCTTTTTACAATCGGGGGATTGACTGGAGT
GATTTTAGCTAACTCATCTATTGATATTATTCTTCATGATACCTATTATGTAGTCGCTCATTTTCACTATGTT
CTATCAATAGGGGCTGTTTTTGCTATTATAGCAGGCCTAGTTCAATGATTCCCCCTATTTACCGGATTAACTT
TAAATGAATATTTTCTAAAAATTCAATTTTTTATAATATTTATTGGTGTTAATTTAACCTTTTTCCCCCAACA
TTTTTTAGGATTAGCAGGAATGCCTCGTCGTTATTCTGATTACCCCGATGCCTATACTCCCTGAAATGTTATT
TCATCAATTGGGTCCCTAATCTCAATAGTTAGAATTTTTTTTTATTATTTATTATTTGAGAAAGATTTTCCT
CAATACGATTAGTTATTTCATCTAAAAACTTTTCAACTTCAATTGAATGATACCAA?????????????????
???????????????????????????????AAGAATTACGTAGTAGTATTCGATTTCCTTGGT?AAAGATTC
CATTAGATATTACAATGAAGTACCTGTTGAGAAACGAGTTTTTAAAAACCTCCAATTATTCATGGAAAACAAA
TCTCCAGGAGACGACTTGTTTGATAGATTAAACACAGCTGTGATGAACAAACATTTAAACGAGTTAATGGAAG
GTTTAACCGCCAAGGTGTTTCGTACTTACAATGCGTCTTGGACCTTACAACAACAACTCGATAAACTGACCAA
TCCAGACGATTCCATATCCGAGAAAATTCTTTCCTACAACCGAGCAAACCGTGCGGTCGCCATCCTCTGTAAC
CATCAACGTGCGGTACCGAAAGGTCATCAAAAATCGATGGAGAAATTAAAGGAAAAAATCGAAGCTAAAAAGG
AAAATATACGTGACGGTGAACGGCAAGTTAAGGACGCTCAGAGAGACGCAAAGCA???CGGTAGCGTTAAGGA
GAAACAGATTTACGAGAAGAAGAAAAAGATGTTGGAGAGACTTAGAGAACAATTAGCTAAATTAGAGATACAA
GAGACGGACCGCGATGAAAATAAAACTATTGCTCTCGGCACGTCAAAGTTGAATTATTTGGATCCGAGGATTT
CGGTTGCTTGGTGTAAGAAGTTTGATGTGCCCATTGAAAAGATTTATAATAAAACTCAC??????????????
???
???GCTGGCGAACAGCA
TCTCATCGAATAGCATCCACAGCAAGCGCGAGAACAAGCCGCGCAAATACAAATACGGATTCCAATTAAAACC

GTACAATCCCGACCACAAGCCGCCTAGTCCAAAAGATCTGGTGTACTTGGAGCCGTCGCCCGGTTTTTGCGAG
AGGAATCCAAAATTGGGCATACAGGGCACACACGGCAGACAA?TGCAACGACACTTCCATCGGAGTTGACGGC
TGCGATTTGATGTGCTGCGGAAG?AGGCTACAGGACGCAAGAAGTAGTCGTTATCGAGAGGTGCAACTGCACC
TT???????????

Eustilicus

GGGAGAAGCCCAGCACTGAATCCCGTGGCCGAACCGGGAAATGTAGTGTTTGGGAGGATCCACTATCCATCGT
GCGACGCGTCCAAGTCCTTCTTGAACGGGGCCACATACCCATAGAGGGTGCCAGGCCCGATAGCTGGAGGATC
TCTCCTCAGAGTCGGGTTGCTTGAGAGTGCAGCCCTAAGTGGGTGGTAAACTCCATCTAAGGCTAAATATGAC
CACGAGACCGATAGCGAACAAGTACCGTGAGGGAAAGTTGAAAAGAACTTTGAAGAGAGAGTTCAATAGTACG
TGAAACCGTTCAGGGGTAAACCTGAGAAACCCGAAAGGTCGAATGGGGAGATTCAGCGTGTCTCGTTTCTGGT
CGAGTGACGATGGTGCTTGCACCGGGTTGCGCCGTCCGGATCCGTATCCGGCGACGAACTCGTGCACTTCTCC
CCTAGTAGGACGTCGCGACCCGTTGGGCGCCGGTCTAAGGCCGACGGAGGAGCCTTGGGGTCCCGGCCGGCCC
GCTCGACGGTAAGACAGAGGCGTGGGGTCGCTACGTTAGCGTCCGGCCCGCCACAAGTTCGGGCGACTCGGAT
GTCGGACCTGTGTGCCGACCTCGAGCTCGCCGGCTGCTGGTGGCGGTGTCCTCGGACAGACTACACGCCGGTC
GGCGACGCTCTAGCTTTGGGTTTTCAGGACCCGTCTTGAAACACGGACCAAGGAGTCTAGCATGTGCGCGAGT
CATTGGGACCGCATCTAAACCTAAAGGCTAAATGAAAGTGAAGGCGTGCCGAGGGAGGATGGGTCGGGGGGCG
TCTCGTTCTCATCGCGAGATGAGGCGCACCCAGAGCGTACACGC?????????????????????????????
?????????????????????????????CTTCAAAAAGGGCGACAAGCACCCGCCCAAGAACTGGGGCGACGT
GAACACCTTCGCCAACCTGGACCCCGCCGGCGAGTACGTGGTGTCCACCCGCGTCCGCTGCGGCCGCTCCATG
GAGGGCTACCCCTTCAACCCGTGCTTGACCGAGGAGCAGTACAAGGAGATGGAGGCGAAGGTGTCCGGCACCC
TGTCCGGCCTCGAGGGCGAGCTCAAGGGCACCTTCTACCCGCTGACCGGCATGGACAAGGACACCCAGCAGAA
GCTGATCGACGACCACTTCCTGTTCAAGGAGGGCGACCGCTTCCTGCAGACCGCCAACGCTTGCCGCTTCTGG
CCGTCCGGCCGTGGTATCTACCACAACGACAACAAGACCTTCTTGGTCTGGTGCAACGAAGAGGACCACCTGC
GCATCATCTCGATGCAGATGGGTGGCGATCTTGGCGAGGTCTACCGTCGCCTCGTGACCGCCGTCAACGAGAT
CGAGAAGCGCGTCCCGTTCTCCCACAATGACCGATTGGGTTTCCTCACCTTCTGCCCGACCAACCTGGGCACA
ACTGTACGTGCCTCTGTACACATCAAAGTACCTAAGCTCGCCGCCAACAAGGCCAAGCTCGACGAGGTCGCCG
GCAAGTACAACTTGCAGGTACGCGGCACTCGCGG?GTACCTAAAAAGGTTCTAATAATTGGTTCAGGTGGTTT
ATCCATTGGACAGGCCGGAGAGTTCGATTATTCCGGTTCACAAGCTATCAAAGCTTTGCAAGAAGAAAATATA
CAAACAGTACTAATTAATCCAAACATAGCTACTGTACAAACATCAAAGGGTTTAGCTGATAAAGTATATTTCT
TACCTTTAGTGCCTGAATTCGTAGAACAAGTAATTAGAGTGGAACGTCCTGGTGGTGTTCTATTAACATTTGG
TGGTCAAACAGGGTTAAATTGTGGTGTAGAGTTACAAAAGGCTGGTATATTTGATAAATATGGTGTTAAAATT
TTGGGTACACCAATAGAAGCTATAATAGATACTGAAGATAGAAAGATTTTTAGTGAGAGAATATCATTAATTG
GTGAAAAAGTTGCTCCAAGTATGGCCGCTTATTCAGTACAAGAAGCTTTGGAAGCGGCCGAGTTATTAGGGTA
TCCAGTTATGGCAAGAGCTGCATTTTCTTTAGGTGGATTAGGATCCGGATTTGCTAATACAGCTGAAGAACTG
AAATTACTTGCTCAACAAGCTTTAGCTCATTCCAACCAGTT?????????????????????????????????
?????????????????????????????????CCTTACCTGAAAGAAGTTAATGATGATGAATTACAAGAACC
TACAGATAAAAGAATGTTCGTTCTAGCAGCAGCTTTAAGAAATGGCTACAGTGTAGATAAGTTATATGATTT?
AACAAAAATTGATCGTTGGTTCCTACAAAAAATGAAGAATATTATAGATTTCAACACTCTCCTTGAAAAAGTT
CAACAAAATAAATTACAGAATTGCTCAAATACATATAAGCTTCTATTAAAAGCAAAACAAATTGGTTTCAGTG
ATAAACAAATTGCTGTTGCTGTTAAAAGTACTGAGCTTGCAATTAGAAAGCAAAGACAAGATTTCGGTATTAC
TCCCTATGTTAAACAAATTGATACTGTGGCTGCTGAATGGCCTGCAACTACAAATTATCTATATTTAACGTAC
AATGCAGAAAGTCATGATCTAACTTTCAGTGATCAGCATATAATGGTTATTGGATCAGGAGTTTACAGAATTG
GAAGTTCTGTTGAGTTTGATTGGTGTGCTGTGGGGTGTTTGAGGGAGCTTAGAAAATTAAATAAGAAGACAAT
AATGGTTAATTACAACCCGGAAACTGTGAGTACGGATTATGATATGTCAGATAGGTTGTACTTCGAGGAAATA
TCTTTCGAAGTTGTTATGGAT???????????????????????????????????AATTTCTCATATTATTA
GTCAATCAAGTGGTAAAAAAGAAACTTTTGGAACTTTAGGAATAATTTATGCAATAATAGCTATTGGATTATT
AGGATTTGTTGTTTGAGCTCATCATATATTTACAGTTGGAATAGATGTTGATACTCGAGCTTATTTCACATCA
GCTACAATAATTATTGCTGTTCCAACAGGAATTAAAATTTTTAGTTGATTAGCCACCCTTCATGGAACCCAAA
TTAAATTTACTCCACCAATATTATGATCATTAGGATTTGTATTTTTATTTACTATTGGAGGATTAACAGGAGT
AATCTTAGCTAATTCATCAATTGATATTATCCTTCATGATACTTATTACGTAGTAGCCCATTTCCATTACGTC
TTATCAATAGGGGCAGTATTCGCTATTATAGCCGGATTAGTGCAATGATTCCCATTATTTACTGGATTAACTT
TAAATGAATTTATACTTAAAATTCAATTTTTTATCATATTTATTGGAGTTAATTTAACATTTTTCCCTCAACA
CTTTTTAGGATTAGCTGGAATACCTCGTCGTTACTCTGATTATCCTGATGCTTATACTCCTTGAAATACTATT
TCATCTATTGGATCTCTAATTTCTATAATTAGAATTTTCTTTTTATTATTTATTATCTGAGATAGATTTATCT
CCATACGAATAAATTTATCTG??
????????????????????????????????AAAGACTATGTGGTCGTATTTGATTTCCTCGGT?AAGGATTC
TATTAGATATTATAATGAAGTACCTGTGGAAAAGCGTGTCTTCAAGAATCTCCAATTGTTCATGGAAAATAAG

TCGCCTGCAGATGATTTGTTCGATAGATTAAACACAGCTGTGATGAATAAACATTTAAACGAGTTAATGGAGG
GCTTAACTGCCAAGGTGTTTCGTACTTACAACGCTTCCTGGACTTTGCAACAGCAGCTCGACAAATTGACCAA
CCCAGATGAATCCATATCCGAAAAAATTTTATCATACAACCGTGCCAACAGAGCAGTAGCTATACTTTGCAAC
CATCAGCGCGCTGTGCCCAAGGGCCACCAGAAGTCCATGGAGAAACTCAAGGAGAAGATCGAGGCCAAGAGGG
ACAACATTAAGGACGCCGAGAGGCAGGTTAAGGACGCTCAAAGGGATGCCAAGCA???CGGCAGCGTCAAGGA
GAAACAGATCTACGAGAAGAAGAAGAAGATGCTGGAGAGACTCAGAGAGCAGTTGGCCAAATTGGAGATCCAG
GAGACTGACCGCGACGAAAACAAAACCATTGCCCTCGGCACGTCCAAGCTGAACTATTTGGACCCTAGGATCT
CGGTCGCCTGGTGCAAGAAGTTTGGTGTGCCCATTGAAAAGATTTATAACAAAAC?????GGATGCGGCGGCC
GCCGTTCCGCGTGATCGGCGACCACCTGAAGGACCGCTTCGACGGCGCGTCGCGCGTCATGCTCAGCAACTCG
GCCAGCTCGCGCGG????????????CAACGC???GAACCGGC???CCAAGCAGGACAAGCTGTCGAACAGCA
TCGCCTCGAACAGCATACACAGCAAGCGGGAGAACAGGCCGCGCAAGTACAAGTACGGCTTCCAGCTGAAGCC
GTACAACCCGGACCACAAGCCGCCCAGCCCGAAGGACCTGGTGTACCTGGAGCCGTCGCCCGGCTTCTGCGAG
AAGAACCCGAAACTCGGCATACAGGGCACGCACGGCAGACTG?TGCAACGACACGTCCATCGGCGTCGACGGC
TGCGACCTGATGTGCTGCGGCAG?GGGCTACCGGACGCAGGAGGTGATCGTCGTCGAGCGGTGCAACTGCACG
TT???????????

Haplonazeris

???
???
???
???
???
???
???
???
???
???
???
???
??AAGCACCCTCCTTCTAACTGGGGTGATGT
AAACACCTTCKTTAATCTTGATCCAGCTGGTGAATACGTASTCTCCACCCGCGTTCGTTGTGGTCGCTCCATG
GAAGGCTATCCCTTCAACCCCTGCTTAACTGAARAACAATACAAARAAATGGAAGGTAAAGTTTCCAGCACTT
TATCTGGTCTCRAAGGTGAACTCAAGGGTACTTTCTACCCATTGACTGGAATGGATAAARACACCCAACARAA
SCTCATCRATGATCACTTCTTGTTCAAGGAAGGTGATCGTTTCCTTCARGCTGCCAAC???????????????
??????????????????TACCACAACGACAACAAAACATTYTTGGTYTGGKGCAACGAAGAGGATCATYTTC
GCATTATYTYTATGCAAATGGGKGGAGATYTTGGTGAAGTYTATYGTCGCCTTGTAACCGCTGTCAACGAAAT
GGAGAAGCGCGTTYCCTTYTCCCACAATGACAGATTAGGTTTCCTCACTTTYTGCCCATCCAACTTGGGCACA
ACTGTACGTGCCTCTGTACACATTAAAGTACCTAAGCTCGCTGCCAACAAGGCCAAGCTCGATGAAATCGCTG
CC???
???
???
???
???
???
???
???
???
???
???
???
???
???
???
???
???
???
???

???
???
???
???
???
???
???
???
???
???
???
???
???
???
???
???
???
???
???TGGATGCGCCTTCC
GCCCTTCAGAGTWATCGGCGACCATCTAAAAGACCGCTTCGATGGCGCTTCCAGAGTCATGCTCAGCAATTCG
GCGAGTTCCCGAGG????????????AAACGC???CAACCGTC???CGAAGCAAGACAAACTTTCGAACAGCA
TAGCTTCGAATAGCATTCACAGCAAAAGGGAGAACCGACCGCGGAAATACAAATACGGTTTTCAATTGAAACC
TTACAATCCGGACCATAAACCTCCGAGTCCTAAGGATTTAGTGTACTTGGAACCGTCGCCTGGTTTCTGCGAG
AAGAACCCGAAGCTTGGTATACAGGGTACTCATGGTAGGTTG?TGCAACGATACTTCCATTGGTGTTGATGGA
TGCGATTTGATGTGCTGCGGTAG?GGGCTACAGGACACAGGAAGTCATCGTTGTTGAGAGGTGCAACTGCACG
TTCCAYTGGTGCT

Hyperomma

GGGAAAAGCCCAGCACTGAATCCCGCGGCCGAGCCGGGAAATGTAGTGTTAGGGAGGGTCCGCTATCCGTCGC
GCGGCGCGTCCAAGTCCTTCTTGAACGGGGCCACTTACCCACAGAGGGTGCCAGGCCCGATCGCGGGAGGATC
TCTCCTCAGAGTCGGGTTGCTTGAGAGTGCAGCCCTAAGCGGGTGGTAAACTCCATCTAAGGCTAAATATCAC
CACGAGACCGATAGCGAACAAGTACCGTGAGGGAAAGTTGAAAAGAACTTTGAAGAGAGAGTTCAATAGTACG
TGAAACCGTTCAGGGGTAAACCTGAGAAATCCGAAAGGTCGAATGGGGACATTCAGCGCGTCTCGGCTTAGGT
CCGGAGACGGCGGCGTTCGCGTTCCGCCGCTCCGACCGAAGCCGCGACCGCTCCCGAACGCGTGCACTTCTCC
CCTAGTAGGACGTCGCGACCCGTTGTGTGCAGGTCACAGGCCCGCGAGGGAGCCCGCCGGTCCCGACCTGCTC
GCACGACGGTATAACGGTGGCGAAGGGTCGCGAAGTTCGCGTCCGGCCCGTCGCAAGCACAGGCGACGGGGAC
GTCGGACCTATGTGCCGACGCCCCGCACGCCGGCTGTTGGCGACGGTGTCCTCGGACGGACCACACGCCCGTC
GGCGACGCTTTAGCTTTGGATTTTCAGGACCCGTCTTGAAACACGGACCAAGGAGTCTAGCATGTGCGCGAGT
CATTGGGACCGCATCTAAACCTAAAGGCGAAATGAAAGTGAAGGCGTGCCGAGGGAGGACCGGTCCGGGGGCG
TCTCGTTCTCATCGCGAGATGAGGCGCACCCAGAGCGTACACGC?????????????????????????????
????CATTATTGTGGTYTACYTTACTGGATTCTCGAACATAGAMAAGAHTSCAGCCRAGAACTGCAGTGATGT
CAACGTCTTCACCAASCTTGACCCTGCCGGCGAGTACGTCGTGTCCACGCGCGTCCGTTGCGGCCGWTCCATG
GAAGGTTACCCCTTCAACCCCTGCTTAACCGAGGACYAATACAAGGAGATGGAACAGAAAGTTTCCAYCACCC
TGTCCGGTCTCGAGGGAGAGCTCAAGGGCACCTTCTACCCACTGACTGGYATGGACAAGGACACCCCA?????
???
???
???
???
???
???????????????????????????????????ATTCCGAAAAAGGTGCTGATCATTGGTTCAGGTGGTTT
ATCGATTGGACAGGCTGGAGAGTTTGATTATTCTGGATCGCAGGCGATCAAGGCGTTGCAGGAAGAAAATATC
CAAACTGTTTTAATTAATCCGAATATAGCAACTGTGCAGACTTCCAAAGGTTTGGCTGATAAGGTGTATTTTT
TGCCTTTGGTGCCTGAATATGTTGAGCAGGTGATTAGAGCTGAACGACCTGGAGGTGTTTTATTGACATTCGG
TGGACAAACAGGTTTGAATTGCGGCGTGGAACTGCAAAAGGCTGGGATTTTTGAGAAATACGGTGTGAAGATC
CTAGGAACTCCGATCCAGGCAATTATCGATACTGAGGATCGCAAGATTTTCAGTGAAAGAATCGGATTGATTG
GGGAAAAGGTTGCTCCAAGCATGGCTGCGTATTCAGTTCAAGAAGCCCTGGATGCAGCTGAACAATTGGGTTA
TCCAGTGATGGCCAGAGCTGCATTTTCCCTAGGTGGCCTTGGATCTGGATTTGCGAATACTGCAGAGGAATTA
AATCGTTGTCGCAGCAGGCTTTGGCCCATTCGAACCAGTTGATAATCGATAAATCGCTGAAGGGCTGGAAGG

AGGTTGAATATGAAGTTGTACGTGACGCTTTTCCGTACATAAAGGAAGTTAAYGACGAAGAATTRAAAGAACC
GACGGACAAAMGAATGTTTGTGGTAGCGGCTGCCCTGCGGAACGGTTACAGCGTCGATAAATTATACAGCTT?
AACCAAAATCGACCGATGGTTCCTTCAAAAAATGAAGAACATAATCGATTTCAATACTCTACTAGAATCGATA
CACCAGCACAAATTA???????????????ACTGGCGAGACTTTGCTCAAAGCCAAACAAATCGGTTTCAGCG
ATAAGCAAATTGCGGCTGCAGTTAAAAGCACGGAACTGGCAATCAGAAAGCAGAGACACGATTTTGGAATCAC
ACCGTTTGTMAAACAGATCGATACGGTTGCAGCCGAATGGCCTGCGACCACGAATTATCTGTATTTAACATAC
AACGCGTTGAGCCACGATTTGGAATTTGCCGAAGAGCACACAATGGTCATTGGTTCGGGTGTTTATCGAATTG
GCAGYTCRGTAGAGTTCGATTGGTGCGCCGTCGGTTGTCTAMGAGAGTTGAGAAACTTGAATAAAAAGACCAT
CATGGTCAATTACAACCCCGAAACGGTCAGTACAGATTATGATATGTCCGATCGGTTGTACTTCGAAGAGATT
TCGTTTGAGGTCGTGATGGAC?AGGTGTATATTTTAATTCTTCCAGGATTTGGTATAATTTCCCATGTAATTT
GTTATAGAAGAGGTAAACCTGAAACATTTGGTACACTAGGGATAATTTATGCAATATTAGCAATTGGGTTATT
AGGATTTATTGTTTGAGCTCACCACATATTTACAATTGGAATAGATATTGATACTCGTGCTTATTTTACTTCA
GCAACAATAGTAATTGCAGTTCCAACTGGAATCAAGGTTTTTAGTTGAATAGCTACAATTTATGGAGGAAATA
TTAGCTTTAGACCCCCAATAATATGATCCCTAGGTTTTATTTTTCTTTTTACAGTAGGGGGGTTAACAGGAGT
AATCTTGGCTAATTCATCTATTGATATCATTTTACATGATACTTATTACGTTGTAGCCCACTTTCATTATGTT
TTATCTATAGGAGCTGTATTTGCAATTATAGCAGGATTAGTCCAATGATTTCCCCTATTCGTAGGTCTAACCT
TAAATGAAAAATACCTAAAAATTCAATTTTTAGTGATATTTATTGGTGTAAATTTCACATTTTTCCCTCAACA
TTTCTTAGGATTATCTGGTATACCCCGCCGATACTCTGACTATCCAGATGCATATACTACATGAAATGTAGTT
TCATCTTCTGGATCAATAGTTTCTTTTATTGGAATCATAACTTTTTTATGAATTATTTGGGAAGCACTAATTT
ATAAGCGTCAAGTTATTTTTATACCTGCCCTTCCCACAGCTATTGAATGAATACATTTTTTCCCCCCTGCCGA
ACATACATATAATGAACTTCCCATAATTAGA???GACTACGTGGTCGTGTTCGATTTCCTCGGC?AAAGATTC
CATTAGGTATTACAACGAGGTGCCGGTCGAGAAGCGCGTGTTTAAAAATCTGCAGCTGTTCATGGAGAACAAG
TCGCCGGGCGACGATCTGTTCGACCGTCTGAACACCGCCGTGATGAACAAACATTTGAACGAGCTCATGGAGG
GGCTCACGGCCAAAGTGTTCCGTACTTACAATGCGTCGTTCACTCTGCAGCAGCAACTGGACAAATTGACCAA
CGAGGACGATTCCCTCTCGGAAAAGATACTGTCSTACAACAGGGCCAACAGGGCGGTGGCCATCCTGTGCAAC
CATCAGCGGGCCGTCCCCAAGGGCCACCAGAAGTCGATGGAGAAGCTCAAGGAGAAGATCGACGCCAAAAGGG
ACGCGATCAAGGACGGGGAGCGGCAGGTGAAGGACGCCCACAGGGACTCGAAGCA???CGGCAGCGTGAAAGA
GAAGCAGATCTACGACAAGAAGAAGAAGATGCTGGAGAGGCTAAAGGATCAGCTGGCCAAGTTGGAGATCCAG
GAGACGGACCGCGACGAAAACAAGACCATCGCCCTCGGCACGTCGAAATTGAATTATCTGGACCCTAGGATAT
CTGTCGCATGGTGCAAAAAGTACGACGTCCCGATCGAGAAGATCTACAACAAAACCCAA??????AGATTACC
TCCTTTCAGAGCCATTGGCGATAATCTCAAAGACCGCTTCGATGGTGCATCGCGGGTCATGCTGACCAACTCC
GCCAGTTCGAG???????????????AAACAG???CAACAGGC???CGAAACAGGATAAGCTCAGCAACAGCA
TTGCCTCTAACAGCATCCACAGCAAAAGAGAGAACCGTCCCCGCAAGCACAAGTACGGTTTCCAGCTGAAACC
GTACAACCCAGACCACAAGCCTCCGAGCCCCAAAGATCTCGTGTACCTAGAACCATCACCGGGCTTCTGCGAG
AGGAATCCCAAGTTGGGCATCCAGGGTACCCACGGCAGGCAG?TGTAACGATACTTCGATCGGGGTGGACGGT
TGTGACCTGATGTGTTGCGGGAG?AGGGTATCGCACCCAGGAGGTCCTGGTGGTCGAGCGGTGCAACTG????
?????????????

Lathrobium

GGGAGAAGCCCAGCACTGAATCCCGCGGCCGAGCCGGGAAATGTAGTGTTTGGGAGGGTCCGTCATCCATCGT
ACGACGCGTCCAAGTCCTTCTTGAACGGGGCCACATACCCATAGAGGGTGCCAGGCCCGATAGCCGGAGGATC
TCTCCTCAGAGTCGGGTTGCTTGAGAGTGCAGCCCTAAGTGGGTGGTAAACTCCATCTAAGGCTAAATATGAC
CACGAGACCGATAGCGAACAAGTACCGTGAGGGAAAGTTGAAAAGAACTTTGAAGAGAGAGTTCAATAGTACG
TGAAACCGTTCAGGGGTAAACCTGAGAAACCCGAAAGGTCGAATGGGGAGATTCAGCGTGTCTCGTTTTTGGT
CGCGTGACGTGGGCGTTCGCGCCGGTTCGCGCCCTCCGAATCCGCAACCCGCGGCGAACTCGTGCACTTCTCC
CCTAGTAGGACGTCGCGACCCGTTGGGCGCCGGTCTAAGGTCGGGGGTGGAGCCTCTGGGTCCCGGCCGGCAC
GCTCGACGGTAGGACAGAGACGTGGGGTCGCGACGTTCGCGTCCGGCCCGTCACAAGCTCGGGCGTCTCGGAC
GTCGGACCTGTGTGCCGACCTCGAGCACGCCGGCTGTTGGTGGCGGTGTCCTCGGACAGACTACACGTCGGTC
GGCGACGCTTTAGCTTTGGGTTTTCAGGACCCGTCTTGAAACACGGACCAAGGAGTCTAGCATGTGCGCGAGT
CATTGGGACCGCATCTAAACCTAAAGGCGAAATGAAAGTGAAGGCGTGCCGAGGGAGGATGGGTCGGGGGGCG
TCTCGTTCTCATCGCGAGATGAGGCGCACCCAGAGCGTACACGC?CTTACACCGTATTCGCTGACTTGTTCGA
?CCCCATCATCGAAGACTACCATGGTGGATTCAAGAAGACCGACAAGCATCCCCCCTCCAACTGGGGTGACGT
AAACACTTTCGTCAACCTTGACCCTGCCGGTGAATACGTCGTGTCAACTCGCGTACGTTGCGGCCGCTCCATG
GAAGGTTACCCCTTCAACCCCTGCTTAACCGAAGATCAATACAAGGAGATGGAAGGGAAGGTTTCCACCACTC
TCTCCGGACTCGAGGGTGAACTTAAGGGTACCTTCTACCCATTGACCGGAATGGACAAGGATACTCAACAGAA
ATTGATCGATGACCATTTCTTGTTCAAGGAGGGAGATCGTTTCCTCCAGACCGCCAACGCTTGCCGTTATTGG
CCATCTGGACGTGGTATCTACCACAACGACAACAAAACATTCTTGGTCTGGTGCAACGAAGAAGATCATCTCC
GTATCATCTCCATGCAGATGGGTGGTGATCTTGGTGAGGTCTACCGTCGCCTCGTCACGGCCGTCAACGAAAT

TGAGAAGCGCGTACCATTCTCTCACAATGACAGATTAGGTTTCCTCACTTTCTGCCCAACCAACTTGGGCACA
ACTGTACGTGCCTCTGTTCACATTAAAGTACCTAAGCTCGCCGCCAACAAGGCCAAGCTT?????????????
??????????????????????????????????GTTCCAAAAAAAGTTTTAATAATCGGGTCTGGTGGTTT
ATCAATTGGTCAAGCTGGAGAATTCGACTAYTCAGGTTCCCAAGCAATTAAAGCACTTCAAGAGGAAAATATT
CAAACGGTCTTAATAAATCCTAACATTGCAACTGTACAAACATCCAAAGGTTTGGCCGATAAAGTTTACTTTC
TTCCGTTAGTCCCGGAATACGTGGAGCAAGTCATTAGAAGTGAAAGGCCTGATGGTGTATTACTGACATTCGG
AGGACAGACAGGGTTAAATTGCGGTGTGGAGCTTCAGAAGGCYGGTGTTTTCGAAAAGTATGGAGTTAAAATT
TTGGGTACACCTATTGAGGCAATCATCGATACTGAAGATAGAAAAATATTTAGTGATAGAATAGCTTTAATCG
GAGAGAAGGTAGCACCCAGCATGGCCGCTTATTCAGTTCAAGAAGCTTTGGAAGCAGCTGAATTATTAGGATA
TCCTGTAATGGCAAGAGCTGCCTTTTCCTTGGGTGGATTAGGTTCAGGATTTGCGGACACCGCAGAAGAGTTA
AAATCACTTGCACAACAAGCACTTGCACATTCCACTCAGTTAATTATTGATAAGTCTTTGAAAGGTTGGAAAG
AAGTTGAGTATGAAGTAGTAAGAGACGCTTTTCACAACATTAAACAAGTTAATGATGAAGARTTAAAAGAACC
CACTGATAAAAGRATGTTTGTAGTTGCCGCTGCTTTAAAAAGTGGTTATACTGTCGATAAATTATATGATTT?
AACAAAAATTGATCGTTGGTTTTTACAAAAAATGAAGAACATTATTGACCATACTACTTTGCTAGAGTCGACT
GAACAGATTAAATTA???????????????ACAGCWAAAATTTTGTTGAAAGCAAAGCAAATTGGATTTAGTG
ATAAGCAAATTGCCGCAGCTTGTAAAAGCACTGAACTTGCAATTAGAAAACAACGCCAAGATTTTAATATYAC
TCCTTATGTTAAGCAGATAGATACTGTGGCTGCTGAATGGCCTGCTACTACAAATTACTTATATTTAACGTAT
AATGCTGGAAGTCACGATATAACTTTTACYGAGGAGCACATAATGGTTATTGGATCTGGAGTTTATAGAATTG
GAAGTTCTGTTGAATTTGATTGGTGTGCAGTTGGGTGTCTACGTGAGCTTAGAAAATTAAATAAAAAAACAAT
AATGGTGAATTACAATCCAGAAACTGTAAGCACTGATTATGATATGTCGGATAGGTTGTACTTTGAGGAAATC
TCATTTGAAGTTGTAATGGATGAAGTCTACATTTTAATTTTACCTGGATTTGGAATAATTTCCCATATTATTA
GCCAGGCTAGAGGTAAGAAGGAAACTTTTGGAGCTTTAGGAATAATTTATGCTATGATAGCAATTGGATTATT
AGGGTTTGTTGTTTGAGCTCACCACATATTTACGGTAGGAATGGATGTTGATACCCGAGCTTATTTTACGTCG
GCAACAATAATTATTGCAGTTCCAACTGGAATCAAAATTTTTAGGTGATTAGCAACATTACATGGTACCCAAG
TAAGTTTTAATCCCCCAATACTCTGAGCATTAGGATTTGTGTTTTTATTTACAATTGGTGGATTAACTGGGGT
AATTTTAGCTAACTCCTCTATTGACATTATTCTCCACGATACATATTATGTAGTTGCCCACTTTCATTATGTT
TTATCTATGGGTGCAGTATTCGCTATTATAGCAGGATTAGTTCAATGATTCCCCCTTTTTACTGGAGTAACAT
TAAATGATAACCTTTTAAAAATTCAATTTTTTGTAATATTTACTGGAGTAAATTTGACCTTTTTCCCACAACA
TTTTTTAGGATTAGCTGGAATACCTCGACGATATTCAGATTATCCAGATGCCTACACAACTTGAAATGTTATT
TCTTCAATTGGATCTCTAATTTCTATAGTTAGAATTTTCCTCCTACTATTTATTATCTGGGAAAGTTTTACTT
CAATACGAAAAACACTATCCGCAAAAAATTTTTCTACTTCAATTGAGTGATACCAACTTCATCCTCCTGCTGA
ACATAGATATAATGAATTACCAATATTATCTAAGGATTATGTTGTTGTTTTCGATTTYCTGGGT?AAAGATTC
CATTAGATATTATAATGAAGTACCKGTGGAGAAACGTGTCTTCAAGAACCTCCAGTTGTTCATGGAGAACAAA
GCGAAAGCCGACGATCTATTCGACAGACTAAACACGGCCGTGATGAACAAGCATTTAAACGAACTCATGGAGG
GTTTGACCGCCAAGGTATTTCGTACTTACAACGCCTCTTTTACGCTGCAACAGCAACTCGACAAGCTGACCAA
CGCCGATGATTCCATATCTGAAAAGATTCTATCGTACAACCGTGCCAATAGGGCCGTTGCCATTCTGTGTAAC
CATCAACGTGCTGTGCCCAAGGGCCATCAAAAATCCATGGAGAAGTTAAAGGAGAAAATCGACGCCAAAAGGG
AGAACATAAGAGACGCAGAGCGGCAGGTTAAAGATGCGCAGAAGGACGCAAAGCG???TGGAAGCGTCAAGGA
GAAGCAGATCTACGACAAGAAGAAGAAGATGTTGGAGCGGCTTAAGGAACAACTTGCCAAGTTGGAGATCCAA
GAGACGGATCGCGACGAAAATAAAACCATCGCACTCGGTACGTCCAAGTTGAACTATCTGGACCCGAGAATCT
CGGTCGCCTGGTGTAAGAAGTTCGATGTGCCCATTGAAAAGATTTATAACAAAACTCAA??????CGCCTTCC
ACCCTTCCGGGTGATCGGAGATCACCTGAAGGACCGCTTCGACGGGGCCTCCCGTGTAATGCTGAGCAACTCC
GCTTCCTCCAGGGG????????????CAACGC???GAATCGTC???CAAAGCAAGACAAGCTCTCCAACAACA
TCGCCTCCAACAGCATCCACAGCAAGCGCGAGAACCGCCCGCGCAAATACAAGTACGGCTTCCAACTCAAGCC
CTACAACCCCGATCATAAACCTCCAAGTCCTAAGGACCTTGTATACCTGGAGCCTTCACCCGGTTTCTGCGAG
AAGAACCCCAAACTGGGTATACAAGGCACGCACGGCAGACAG?TGCAACGATACTTCCATAGGTGTCGACGGT
TGCGATCTGATGTGTTGCGGAAG?AGGTTACAGAACCCAGGAGGTCATAGTGGTGGAGAGATGCAACTGCACG
?????????????

Leptobium

???
???
???
???
???
???
???
???

???
???
???
???CATATACCGTTTTCGCGGACTTGTTCGA
?CCCCATCATTGAAGACTACCACACTGGATTCAAAAAGACCGACAAACACCCCCCCAAGAACTGGGGTGATGT
GAACGTGTTTACCAACCTGGACCCTGCTGGTGAGTACGTCGTTTCGACCCGAGTCCGCTGTGGCCGTTCTATG
GAAGGATACCCCTTCAACCCATGCTTAACCGAGGAGCAGTACAAAGAGATGGAGCAGAAGGTATCCACCACCT
TGTCCGGACTTGAGGGCGAGCTCAAAGGAACATTCTACCCTTTAACTGGCATGGATAAAGATACTCAACAAAA
ACTCATCGATGACCACTTCTTGTTCAAGGAGGGTGATCGTTTCCTTCAAGCCGCAAATGCTTGCCGTTTCTGG
CCCAGCGGACGTGGTATCTACCACAACGACAACAAGACCTTCTTGGTCTGGTGCAACGAAGAGGACCATCTTC
GTATCATCTCCATGCAGATGGGCGGTGATCTTGGTGAAGTATACCGTCGCCTCGTTACCGCTGTTAACGACAT
TGAGAAGCGTGTCCCCTTCTCCCACAATGATAGGCTGGGTTTCTTAACTTTTTGCCCAACCAACTTGGGTACA
ACTGTACGCGCCTCTGTACATATTAAGGTCCCCAAACTAGCCGCCAACAAGGCCAAGCTT?????????????
???
???
???
???
???
???
???
???
???
????????????????????????????????CCATATTTAAAAGAAGTTAATGATGAAGAATTGAAAGAACC
AACAGATAAAAGGATGTTTGTATTAGCAGCAGCTTTAAAGAATGGTTATAGTGTAGATAAATTATACAACTT?
AACCAAAATTGACCGTTGGTTTATTCAAAAAATGAAGAATATAATTGATTTCAATAACAAATTAGAATCTATA
GATCAACACAAATTA????????????????TCAGCTGAAATTTACTTAAAGCTAAACAAATTGGCTTCAGTG
ATAAGCAAGTAGCAACAGCTGTCAAAAGTACAGAATTAGCAATTAGAAAACAGCGACATGATTTTGGGTTAAT
TCCATTTGTTAAACAGATTGATACTGTTGCTGCAGAATGGCCAGCTACTACAAATTACCTTTATATAACCTAC
AATGCATCAAATCATGATTTAGAATTCACAGAAGAACATATTATGGTCATAGGTTCGGGTGTGTACAGAATTG
GTAGCTCGGTTGAATTTGATTGGTGTGCTGTTGGTTGTTTGAGAGAATTGAGAAAATTGAATAAGAAAACTAT
AATGGTTAATTATAACCCTGAAACTGTTAGTACAGATTATGATATGTCAGATAGATTATACTTTGAGGAAATA
TCTTTTGAAGTTGTTATGGATGAAGTTTATATTTTAATTTTACCTGGATTGGAAATAATTTCTCATATTATTT
CATATAGAAGAGGTAAATCCGAAACTTTTGGATCATTAGGAATAATTTATGCAATATTAGCAATTGGCCTATT
AGGATTTATTGTTTGAGCCCATCATATATTTACTGTAGGAATAGATGTTGATACTCGAGCCTACTTTACATCT
GCTACTATAGTAATTGCTGTTCCTACCGGAATCAAAGTATTTAGATGAATAGCAACAATTTATGGGGGAAACT
TAAACTTTAACCCTCCTATATTATGATCTTTAGGTTTTGTATTCTTATTTACAGTTGGTGGACTAACAGGTGT
TATTTTAGCTAATTCATCAATTGATATTATTCTTCATGATACATATTATGTAGTTGCTCATTTTCATTATGTT
TTATCTATAGGAGCTGTATTTGCTATTATAGCAGGACTTGTTCAATGATTTCCATTATTTGTAGGATATACCT
TAAATGAAAAATACTTAAAAATTCAATTTTTAATTATATTTATTGGAGTAAATTTAACATTTTTCCCTCAACA
TTTTTTAGGTCTTGCAGGAATACCTCGACGATACTCAGATTATCCAGATGCCTATACAACTTGAAATGTAATT
TCTTCAATTGGTTCAATAGTATCATTTATTGGAATTATGTTCTTTTTATGAATTATTTGAGAAAGATTTGTAT
CAATACGAAAAATCCTAGGATCACCTATTCCACCAACAGCTATTGAATGAATACACTCATACCCTCCCTCAGA
ACATACATATTCTGAATTACCTTATATTACA?????????GTTGTTGTGTTTGATTTCCTCGGC?AAAGATTC
CATTAGATATTATAACGAAGTACCTGTCGAGAAACRCGTCTTTAAAAATCTGCAGCTCTTTATGGAGAATAAA
AAACCCGGTGATGACCTATTTGATCGGTTAAACACAGCTGTTATGAACAAACATTTAAACGAACTTATGGAAG
GTCTAACAGCGAAGGTATTTCGTACTTACAATGCTTCTTTTACTTTGCAACAGCAATTAGACAAATTAACCAA
TCCWGATGACTCTTTGTCGGAGAAAATTTTATCATATAACCGTGCTAATCGAGCCGTAGCTATCCTTTGTAAC
CATCAGCGTGCCGTTCCCAAGGGTCACCAGAAATCAATGGAGAAGCTCAAAGAGAAAATTGACGCTAAGAGGG
AAGCCATTAAAGATGGAGAAAGGCAAGTGAAGGATGCCCAACGAGATGCTAAACA???TGGCAGCGTCAAGGA
AAAGCAGATCTATGATAAGAAGAAAAAACAGCTGGAGAGATTCAAAGAGCAGCTTGCAAAACTAGAAATACAA
GAGACGGACCGTGACGAGAACAAAACTATTGCACTTGGTACGTCAAAACTGAATTATTTGGATCCTAGGATAT
CGGTTGCTTGGTGTAAAAAATTCGACGTGCCCATTGAGAAAATTTATAACAAAACTCAA??????????????
???????????????????GGGAACCTGAAGGACCGATTCGACGGTGCATCGCGAGTCATGCTCAGCAATTCG
GCAAGCTCGAGGAA?????????????????????CAACAGGC???CCAAACAGGATAAACTCAGTAACAGCA
TAGCGTCGAACAGCATACACAGCAAACGAGAGAACAGACCGCGCAAATACAAATACGGTTTCCAACTGAAGCC
CTACAATCCGGACCATAAGCCGCCCAGCCCGAAGGACCTGGTCTACCTGGAGCCTTCGCCCGGTTTTTGCGAG
AAAAATCCCAAATTGGGCATACAAGGCACGCACAGCAGACAA?TGCAACGACACGTCGATAGGAGTCGATGGT

TGTGACCTCATGTGCTGCGGGAG?GGGCTACAGGACGCAAGAAGTCGTCGTC????????????????????
?????????????

Lithocharis

GGGAGAAGCCCAGCACTGAATCCCGTGGCCGAACCGGGAAATGTAGTGTTTGGGAGGGTCCGCTATCCACCGT
GCGACGCGTCCAAGTCCTTCTTGAACGGGGCCACATACCCATAGAGGGTGCCAGGCCCGGTAGCTGGAGGATC
TCTCCTTAGAGTCGGGTTGCTTGAGAGTGCAGCCCTAAGTGGGTGGTAAACTCCATCTAAGGCTAAATATGAC
CACGAGACCGATAGCGAACAAGTACCGTGAGGGAAAGTTGAAAAGAACTTTGAAGAGAGAGTTCAATAGTACG
TGAAACCGTTCAGGGGTAAACCTGAGAAACCCGAAAGGTCGAATGGGGAGATTCAGCGTGTCTCGTTTTTGGT
CGAGTGACGATGGTGTTCGCACTGGGC?GCGCCTTCCGAATCCGTAACCGGCAACGAACTCGTGCACTTCTCC
CCTAGTAGGACGTCGCGACCCGTTGGGTGCCGGTCTAAGGCCGACGGAGGAGACTTGGGGTCCCGGCCGGCCC
GCTCGACGGTAAGACAGAGGCGTGGGGTCGCTACGTTAGCGTCCGGCTCGTCACAAGTTCGAGTGACTCGGAT
GTCGGACCTGTGTGCCGATCTCGAGATCGCTGACTGTTGGTGACGGTGTCCTCGGACAGACTACACGCCGGTC
GGCGACGCTCTAGCTTTGGGTTTTCAGGACCCGTCTTGAAACACGGACCAAGGAGTCTAGCATGTGCGCGAGT
CATTGGGACCGCATCTAAACCTAATGGCAAAATGAAAGTGAAGGCGTGCCGAGGGAGGATGGGTCGGTGGGCG
TCTCGTTCTCATCACGAGATGAGGCGCACCCAGAGCGTACACGC?????????????????????????????
???????????????????CATGGTGGATTCAAGAAGACCGACAAACACCCTCCCAAGAACTGGGGTGATGT
AAACACCTTTGGTAACCTCGACCCAACCGGTGAATACGTCGTATCCACCCGTGTCCGCTGCGGTCGCTCCATG
GAAGGTTATCCATTCAACCCATGCTTAACCGAAGAGCAATACAAGGAGATGGAAGCTAAAGTCTCAAGCACTT
TGTCTGGACTCGAAGGCGAACTTAAGGGTACTTTCTACCCATTGACCGGAATGGACAAGGCTACTCAACAGAA
GCTCATCGATGACCACTTCTTGTTCAAGGAAGGAGATCGTTTCCTCCAGGCTGCCAACGCCTGCCGTTTCTGG
CCATCTGGACGTGGTATCTACCACAACGACAACAAGACCTTCTTGGTCTGGTGCAACGAAGAAGATCACCTTC
GCATTATTTCCATGCAAATGGGCGGTGATCTTGGTGAAGTATACCGTCGCCTTGTAACCGCTGTCAACGAAAT
CGAGAAGCGTGTACCCTTCTCCCACAATGACAGATTAGGTTTCCTCACTTTCTGCCCAACCAACTTGGGCACA
ACTGTACGTGCCTCTGTACACATTAAAGTACCTAAGCTCGCCGCCAACAAAGCCAAACTCGATGAAGTCGCTG
CCAAATACAACTTGCAAGTACGTGGCACCCGCGGTGTTCCTAAAAAAGTATTGATAATTGGATCAGGAGGTTT
ATCTATTGGACAAGCAGGTGAATTTGATTAYTCAGGTTCACAAGCTATAAAAGCATTACAAGAAGAAAACATC
CAAACAGTATTGATCAATCCAAATATTGCTACTGTACAAACATCRAAGGGTTTGGCTGACAAAATATACTTTT
TACCTTTAGTGCCTGAATTTGTAGAACAAGTGATACGAGTAGAACGCCCTGGTGGCGTTTTACTAACATTCGG
TGGACAAACAGGGTTAAATTGTGGTGTAGAACTACAAAAAGCAGGTGTTTTTGATAGATACAATGTTAAAATA
TTGGGAACACCAATACAAGCTATAATAGATACGGAAGACAGAAAAGTATTTAGCGAAAGAATTGCTTTGATTG
GTGAAAAAGTTGCTCCAAGTATGGCTGCTTATTCCGTACAGGAAGCATTGGAAGCAGCTGAGTTAYTAGGATA
TCCAGTTATGGCTAGAGCAGCTTTTTCTTTGGGAGGATTRGGATCTGGTTTCGCTAATACTGCTGAGGAACTG
AAACTACTTGCTCAACAAGCCTTAGCTCATTCAAATCAGTTAATTATTGATAAGTCGTTAAAAGGATGGAAGG
AGGTTGAATATGAAGTTGTTAGAGATGCATATCCCTACATTAAAGAAGTTAATGATGATGAATTGAAAGAACC
CACAGATAAAAGAATGTTTGTGTTAGCAGCAGCTCTACGTAATGGTTACACTGTGGACAAATTGTATGATTT?
GACAAAAATTGATCGATGGTTCTTGCAGAAAATGAAAAATATTATAGATTACAATACGCTTTTGGAATCCATA
CAACAAAATAAATTACAGAATAATGCRCACAATTATAAACTTTTGTTGAAAGCTAAACAAATTGGTTTAGTG
ATAAACAAATTGCTGTTGCTATTAAAAGCACTGAGCTTGCTGTTAGAAAACTGAGGCAAGATTTTGGAATTAC
ACCATATGTCAAACAAATTGATACCGTTGCTGCTGAATGGCCTGCTACAACAAATTACTTGTATTTAACGTAC
AATGCAGAGAGTCACGATTTAACATTTAATGAGCAACATATTATGGTTATAGGTTCGGGTGTTTACAGAATTG
GTAGTTCTGTTGAATTTGATTGGTGTGCTGTTGGTTGCTTGAGAGAACTTAGGAAATTAAATAAAAAGACAAT
AATGGTTAATTACAACCCCGAAACTGTTAGTACAGATTATGATATGTCGGATAGATTATATTTTGAGGAAATA
TCCTTTGAAGTTGTAATGGATGAAGTATATATTTTAATTCTTCCTGGATTTGGGATAATTTCTCATATTATTT
CTCAAGCCAGAGGAAAAAAGGAAACATTTGGAGCATTAGGAATAATTTATGCTATAATAGCAATTGGATTATT
AGGATTTGTAGTTTGAGCTCATCATATATTTACAGTAGGAATAGATGTGGATACTCGAGCATATTTTACTTCT
GCTACAATAATTATTGCAGTACCTACAGGTATTAAAATTTTCAGATGATTAGCAACTTTACATGGAACTCAAA
TTAAATTTACACCTCCTATACTTTGAGCTTTAGGATTTGTATTTTTATTTACTATTGGAGGATTAACTGGTGT
AGTTTTAGCAAATTCTTCTATTGATATTATCCTTCATGATACTTACTATGTAGTAGCTCATTTTCATTATGTC
CTTTCTATAGGAGCAGTATTTGCTATTATAGCTGGTTTAGTCCAATGATTTCCCTTATTTACTGGGTTAACAT
TAAATGAATACTTATTAAAAATTCAATTCTTTGTAATATTTGTTGGAGTGAATCTAACATTTTTCCCTCAACA
TTTCTTAGGATTAGCTGGAATACCTCGACGATATTCAGATTATCCCGATGCATACACACCTTGAAATGTTGTT
TCATCAATTGGAAGAATAATTTCTATATTAAGAATTTTCTTATTATTATTTATTATTTGAGAAAGATTTGTTT
CAATACGAATAAATATTTCTGCTAAAAATTTTTCTACTTCAATTGAATGATTCCAGCTATTTCCCCCTGCTGA
ACATAGATATTCCGAATTACCAATATTAACAAAAGATTACGTAGTGGTATTTGATTTCCTTGGT?AAGGATTC
CATTAGATATTATAATGAAGTACCTGTAGAAAAACGTGTCTTCAAGAACCTTCAATTATTCATGGAAAATAAA
TCACCCGGAGATGATTTGTTTGACAGATTGAACACAGCTGTGATGAACAAACATTTAAACGAATTAATGGAAG
GTTTAACTGCCAAGGTGTTTCGTACTTATAACGCTTCGTGGACACTACAACAGCAACTCGATAAATTGACCAA

TCCAGATGATTCCATATCCGAAAAAATTTTATCATATAACCGTGCCAATAGAGCAGTAGCAATACTTTGTAAC
CATCAACGTGCTGTCCCTAAAGGCCATCAGAAATCCATGGAAAAACTCAAGGAAAAGATCGAAACTAAAAAAG
ACTCTATTAAAGATGCAGAGAGGCAGGTTAAAGATGCACAGAGAGATGCAAAACA???TGGCAGCGTAAAAGA
GAAGCAGATTTACGAAAAGAAAAAGAAAATGCTGGAGAGACTTCGGGAGCAATTGGCGAAATTGGAAATTCAA
GAAACTGACCGTGATGAAAACAAAACAATTGCGCTGGGTACCTCCAAGCTGAATTATTTGGACCCCAGAATTT
CGGTTGCATGGTGTAAGAAGTTTGGTGTGCCCATTGAAAAAATTTATAACAAAACTCAA?????????????
?TCGTTCCGCGTCATCGGCGACCACCTGAAGGACCGCTTCGACGGCGCGTCGCGCGTCATGCTCAGCAACTCG
GCCAGCTCCCGCGG????????????GAACGC???CAACCGCC???CCAAACAGGACAAGCTCTCCAACAGCA
TCGCCTCCAACAGCATACACAGCAAGAGGGAGAACAGGCCGAGGAAATACAAGTACGGCTTCCAGCTGAAACC
CTACAATCCAGACCATAAACCTCCGAGTCCCAAGGATCTTGTGTACTTGGAGCCATCGCCTGGTTTCTGCGAG
AAGAACCCGAAGCTGGGGATCCAGGGTACCCATGGGAGGTTG?TGCAACGACACGTCGATCGGTGTGGACGGG
TGCGATCTGATGTGCTGCGG?AG?AGGGTACAGGACCCAGGAGGTCATCGTCGTTGAGAGGTGCAAC??????
?????????????

Lobrathium

GGGAGAAGCCCAGCACTGAATCCCGTGGCCGAACCGGGAAATGTAGTGTTTGGGAGGGTCCGTCATCCATCGT
ACGACGCGTCCAAGTCCTTCTTGAACGGGGCCACATACCCATAGAGGGTGCCAGGCCCGATAGCCGGAGGATC
TCTCCTCAGAGTCGGGTTGCTTGAGAGTGCAGCCCTAAGTGGGTGGTAAACTCCATCTAAGGCTAAATATGAC
CACGAGACCGATAGCGAACAAGTACCGTGAGGGAAAGTTGAAAAGAACTTTGAAGAGAGAGTTCAATAGTACG
TGAAACCGTTCAGGGGTAAACCTGAGAAACCCGAAAGGTCGAATGGGGAGATTCAGCGTGTCTCGTTTTTGGT
CGCGTGACGATGGTGCTTGCACCGGGCCGCGCCTTCCGAATCCGTAACCCGCGACGAACTCGTGCACTTCTCC
CCTAGTAGGACGTCGCGACCCGTTGGGCGCCGGTCTAAGGCCGAGGGTGGAGCCTCGGGGTCCCGGCCGGCAC
GCTCGACGGTAAGACAGTGGCGTGGGGTCGCGATGTTCGCGTCCGGCCCGTCACAAGCTCGGGCGACTCGGAC
GTCGGACCTGTGTGCCGACCTCGAGCTCGCCGGCTGTTGGTGACGGTGTCCTCGGACAGACTACACGTCGGTC
GGCGACGCTTTAGCTTTGGGTTTTCAGGACCCGTCTTGAAACACGGACCAAGGAGTCTAGCATGTGCGCGAGT
CATTGGGACCGCATCTAAACCTAAAGGCGAAATGAAAGTGAAGGCGCGCCGAGGGAGGATGGGTCGGGGGGCG
TCTCGTTCTCATCGCGAGATGAGGCGCACCCAGAGCGTACACGCTCTTACACCGTATTCGCTGATTTGTTCGA
CCCCCATCATTGAAGACTACCATGGTGGATTCAAGAAGACCGACAAGCACCCCCCTTCCAACTGGGGAGATGT
CAACACTTTCGTCAACCTTGACCCTGCTGGCGAATACGTTGTATCTACTCGCGTACGTTGCGGCCGCTCCATG
GAGGGCTACCCATTCAACCCCCTGCTTAACCGAAGATCAATACAAGGAGATGGAACAGAAGGTTTCCACCACTT
TGTCTGGACTCGAGGGTGAACTTAAGGGTACCTTCTACCCATTGACCGGAATGGATAAGGACACCCAACAGAA
GCTCATCGATGACCATTTCTTGTTCAAGGAAGGAGATCGTTTCCTCCAAACTGCTAACGCTTGCCGTTATTGG
CCGTCTGGACGTGGTATCTACCATAACGACAACAAAACATTCTTGGTCTGGTGCAACGAAGAGGATCATCTCC
GCATCATCTCCATGCAGATGGGTGGTGATCTTGGTGAAGTCTACCGTCGCCTTGTCACAGCCGTCAACGAAAT
TGAGAAGCGCGTACCCTTCTCTCACAATGACAGATTAGGTTTCCTTACCTTCTGCCCAACTAACTTAGGTACA
ACTGTACGTGCCTCTGTACACATCAAAGTACCTAAGCTCGCTGCCAACAAGGCTAAGCTTGATGAGATTGCTG
GAAAGTACAACTTGCAAGTCCGTGGTACTCGTGGTGTTCCAAAAAAAGTTTTAATAATTGGTTCCGGTGGTTT
ATCAATTGGACAAGCCGGAGAATTTGATTATTCAGGCTCGCAAGCAATTAAAGCACTTCAAGAGGAAAATATT
CAAACTGTTTTAATAAATCCGAACATTGCAACTGTCCAAACTTCTAAAGGTTTAGCTGATAAGGTTTATTTCC
TTCCGTTAGTACCAGAGTATGTTGAACAAGTAATTAGAGTTGAAAGACCAAGCGGTGTTTTACTAACTTTCGG
CGGACAAACAGGGTTAAATTGTGGCGTGGAGCTTCAAAAGGCTGGAGTTTTCGAAAAATATGGAGTTAAAATT
TTGGGTACACCTATTCAAGCAATCATCGATACAGAAGACAGAAAAATATTTAGCGATAGAATAGCATTAATTG
GAGAAAAGGTAGCACCGAGTATGGCTGCGTATTCAGTACAAGAAGCTTTGGAAGCGGCAGAATTTTTAGGTTA
TCCTGTTATGGCAAGAGCTGCTTTTTCTTTG???
???
???????????????????????????????CATAATATTAAGCAAGTTAATGATGAAGAATTAAARGAACC
AACTGATAAGAGAATGTTTGTTGTTGCTGCTGCTTTAAAAAGTGGTTATAGTGTTGATAAATTATACGATTT?
AACAAAAAATTGATCGTTGGTTTTTACAAAAAATGAAAAACATTATAGACCATACTACTTTGCTAGAGTCGACT
GAACAAATCAAACTA???????????????ACAGCTAAAATTTTGTTAAAGGCAAAGCAAATTGGATTTAGCG
ACAAGCAGATTGCTGCWGCTTGCAAAAGCACTGAGCTTGCTATTAGAAAACAACGTCAAGATTTTAATATTAC
TCCATATGTTAAGCAAATTGATACCGTAGCTGCTGAATGGCCTGCTACTACAAATTACTTATATTTAACTTAT
AATGCGGGAAGTCATGATTTAACGTTTGATGATGAGCATACGATGGTTATTGGATCTGGAGTTTATAGGATTG
GAAGTTCGGTTGAATTTGATTGGTGTGCAGTTGGATGTTTGCGTGAGCTTAGAAAATTAAATAAAAAGACAAT
AATGGTAAATTACAAT???
?????????????????????GAAGTTTACATTTTAATTTTACCTGGATTCGGAATAATTTCTCATATTATCA
GACAAGCAAGAGGAAAAAAGGAAACATTTGGAACTTTAGGAATAATCTATGCTATAATAGCAATTGGTTTACT
TGGATTTGTTGTATGAGCTCATCATATATTTACAGTAGGAATAGATGTTGATACACGAGCTTATTTTACATCA
GCAACTATAATTATTGCTGTTCCAACAGGAATTAAAATTTTTAGTTGATTAGCAACTTTTCATGGAACTCAGA

TTAATTTAATCCGCCAATGCTTTGAGCTTTAGGATTTGTTTTTTATTTACTATTGGAGGATTGACAGGAGT
AATTTTAGCTAATTCTTCTATTGATATTATTTACATGATACATATTATGTAGTAGCTCATTTTCATTATGTT
CTTTCAATAGGAGCTGTATTTGCTATTATAGCAGGATTAGTTCAATGATTTCCTTTATTTACAGGATTAACTT
TAAACAAAAATATACTTAAAATTCAATTTTTTGTTATATTTACAGGAGTTAATTTAACTTTTTTCCCACAACA
TTTTTTAGGTTTAGCAGGTATACCTCGACGATACTCAGATTATCCTGATGCATATACTACTTGAAATGTAATT
TCATCAATTGGATCTCTAATTTCAATAATTGGAATTTTTTTACTATTATTTTGTGTTTGAGAAAGATTTGTTT
CTATACGAAAAATTTTATCTGCAAAAAATTTTACTACATCAATTGAATGATATCAATCTTATCCTCCTGCTGA
ACATAGTTATGATGAATTGCCCATA???
??
??
??
??
??
??
??
??
???TGGATGCGCCTCCC
GCCGTTCAGGGTGATCGGCGATCACCTGAAGGACCGCTTCGACGGTGCCTCTCGCGTAATGCTCAGCAATTCG
GCGAGCTCTAG???????????????GAATTC???GAACCGTC???CAAAGCAAGACAAGCTTAGCAACAACA
TAGCGTCGAATAGCATACACAGCAAAAGGGAAAATCGCCCGCGCAAATATAAGTACGGCTTCCAGCTTAAGCC
GTACAATCCCGATCATAAGCCTCCGAGCCCCAAGGACTTGGTTTACTTGGAGCCGTCGCCGGGTTTCTGCGAG
AAGAACCCGAAACTGGGGATACAGGGCACGCACGGAAGGCAG?TGCAACGATACGTCCATAGGCGTCGACGGT
TGCGACTTGATGTGCTGCGGAAG?AGGCTACAGAACCCAGGAGGTCATAGTGGTGGAGAGGTGCAACTGCACC
TTCC?????????

Lordithon

GGGAAGAGCCCAGCACCGAATCCCGCGGCCGCGCCGGGAAATGTGGTGTTTGGGAGGGTCCGCCAGCCACCGT
GCGATGCGTCCAAGTCCTTCTTGAACGGGGCCACTTACCCATAGAGGGTGCCAGGCCCGGTAGCGGGAGGATC
TCTCCTTAGAGTCGGGTTGCTTGAGAGTGCAGCTCTAAGTGGGTGGTAAACTCCATCTAAGGCTAAATATGAC
CACGAGACCGATAGCGAACAAGTACCGTGAGGGAAAGTTGAAAAGAACTTTGAAGAGAGAGTTCAATAGTACG
TGAAACCGTTCAGGGGTAAACCTGAGAAACCCGAAAGGTCGAATGGGGAGATTCAGCGTGTCTCGCAGCCGCT
TGGGCTGCGACGCACGTTTCGGCGTGCCTCGCCTCCACGGCGGCCGCCTCGTCGCGAACGCGTGCACTYYT?C
CCTAGTAGGACGTCGCGACCCGTT?GGCGCCGGCCTGAGGCCC?CGGT?GAGCCCGTGTGTCCCAGCCGGCAC
GCTCGACGGTAACACGATGGCGCGGGGCCGCGACGTTCGCGTCCGGCCCGTCGCTGGCGCGGACGGCCTGGCG
GTCGGACCTA?GTGCCGACTCCGGGCCCGCCGCCCGCTGGCGGCGGTGACCTCGGACAGACTACACGCCGGTC
GGCGACGCTATAGCTTTGGGTTTTCAGGACCCGTCTTGAAACACGGACCAAGGAGTCTAGCATGTGCGCGAGT
CATTGGGACTTATGCGAAACCTAAAGGCGAAATGAAAGTGAAGGCGTGCCGTGGGAGCAGGGGTCGGGGGGCG
TCTCGTTCTCATTGC???
?????????????????????????????TTCAAGAAGACCGACAGCCACCCCCCGAAGAACTGGGGTGACGT
AAGCGTCTTCGCCAATCTTGATGCCGCCGGTGAATACATCGTGTCCACCCGCGTCCGTTGCGGTCGCTCCATG
GAGGGCTACCCCTTCAACCCCTGCTTAACCGAGGAGCAGTACAAGGAGATGGAACAGAAAGTTTCCACCACCC
TCTCCGGTTTGGAAGGCGAGCTTAAGGGTACCTTCTACCCATTGACTGGCATGGACAAGGACACCCAGCAGAA
GTTGATCGATGACCACTTCTTGTTCAAGGAGGGAGACCGTTTCCTCCAGACCGCCAACGCTTGCCGTTACTGG
CCCAGCGGCCGTGGTATCTACCACAATGACAACAAGACCTTCTTGGTCTGGTGCAACGAGGAGGATCATCTCC
GTCTGATCTCCATGCAAATGGGTGGTGATCTTGGAGAGGTCTACCGTCGTTTGGTAACCGCCGTCAACGACAT
CGAGAAGCGCGTGCCATTCTCCCACAATGACAGGCTTGGCTTCCTCACTTTCTGCCCCACCAACTTGGGCACA
ACTGTCCGCGCGTCCGTGCACATCAAGGTGCCCAAGCTCGCCGCCAACAAGGCTAAGCTCGATGAGATCGCTG
GCAAGTACAACTTGCAAGTCCGCGGTACCCGTGGT?????????????????????????????????????
??
??
??
??
??
??
??
??
????????????????????????????????CCGTATTTAAAAGAGGTTAATGATGAAGAACTTACTGAGCC
CACCGATAAACGTATGTTTGTAGTAGCCGCAGCGCTCCGCAGTGGATATACTGTAGATAAACTATACAACCT?

CACCAAAATCGATCGATGGTTTTTGCAGAAAATGAAGCACATTGTGGATTACAACTCGTTTTTGGAAACCCTT
AATGGGCGTAATTTA???????????????ACATACGATGTGCTACTCGCTGGTAAAATGATTGGGTTTAGTG
ATAAACAAATTGCATCAGCTGTTAAAAGCACTGAATTGGCTGTACGTAAGAAACGCGAGGATTTAAATCTCAA
ACCGTTTGTGAAACAAATTGATACCGTTGCCGCTGAATGGCCTGCAACTACCAATTATTTGTACCTAACATAT
AATGCTGAAGAACATGATTTGGAATTTAATGACGAACATACAATGGTTATAGGTTCGGGGGTTTATCGTATTG
GAAGCTCTGTGGAATTCGATTGGTGCGCTGTGGGATGTCTTAGGGAGCTCAGAAGGTTGAATAGGAAGACGAT
TATGGTTAATTATAATCCGGAGACGGTGAGCACGGATTACGATATGTCGGATCGATTGTATTTTGAAGAAATA
TCGTTTGAGGTCGTTATGGAT?????????????????????????????????ATAATCTCTCATATTATTA
GCCAAGAGAGTGGTAAAAAGGAAACTTTTGGGGCACTTGGGATAATTTATGCTATAATAGCAATTGGTTTATT
AGGATTTGTAGTTTGAGCCCATCACATATTTACAGTGGGAATAGACGTTGATACTCGAGCTTACTTTACCTCA
GCAACTATAATTATTGCAGTTCCAACTGGGATTAAAATTTTTAGATGACTTGCCACACTTCACGGAACACAAA
TTAACTACTCACCCTCTATATTATGGGCATTAGGATTTGTATTTTTATTTACTGTTGGAGGTCTAACAGGAGT
TATTTTAGCTAATTCATCTATTGATATTATTCTGCATGATACCTACTATGTAGTGGCCCATTTTCACTACGTA
CTTTCTATAGGTGCAGTATTTGCTATTATGGCTGGATTAATCCAATGATTCCCCTTATTCACTGGATTAACAA
TAAACGAAAAGTTTTTAAAAATCCAATTTTCAGTGATATTCATTGGAGTAAACCTAACTTTTTTCCCACAACA
TTTTTTAGGATTAGCAGGTATACCTCGTCGATACTCTGACTACCCTGATGCTTATACATCCTGAAATGTAATT
TCATCAATTGGGTCTTTAATTTCTTTAACTAGAATTTTCTTACTCCTTTTTATTATTTGAGAAAGATTTATCT
CTATACGAAAAAGAATTTCATCAATAAATTTACCTTCATCAATTGAATGATTACAAACAATACCCCCAGCTGA
GCATAGTTATTCTGAATTACCGTCCCTAACA??
???
?????????????????????????????????ACCGCCGTGATGAACAAGCATTTGAATGAACTCATGGAGG
GGTTAACTGCGAAAGTATTCCGTACGTATAACGCCTCATTCACTCTACAGCAGCAACTAGACAAATTAACCAA
CCCTGATGATTCCTTATCTGAAAAAATCTTATCCTACAATCGCGCCAACCGAGCCGTGGCCATCCTATGTAAC
CATCAACGGGCCGTGCCGAAAGGCCACCAGAAGTCCATGGAAAAACTCAAAGAGAAAATCGACACTAAAAGAG
AAACCATCAGAGACGCCGAGCGATCGGTTAAAGACGCGCAAAAGGACGCCAAACG???CGGCAGCGTAAAAGA
GAAGCAAATCTACGACAAGAAGAAGAAAATGCTAGAGCGCCTAAAGGACCAACTAGCCAAACTGGAGATCCAA
GAAACCGATCGGGACGAAAACAAAACAATCGCGCTGGGCACGTCAAAACTGAACTATCTCGACCCGCGTATTT
CGGTGGCGTGGTGCAAGAAGTACGATGTGCCCATTGAGAAGATCTATAATAAAACCCAG?????????????
???????????????CGGTGATAACCTAAAGGACCGCTTCGATGGTGCCTCACGAGTGATGCTAAGCAACGCG
GGGCACGCACGCAA????????????CACAGC???GAGTCGCC???CGAAAGCCGACAAACTGTCCAACAACA
TCGCCTCCAACAGCATCCACAGCAAACGCGAAAACCGCCCGCGCAAATACAAATACGGCTTCCAACTGAAACC
CTACAACCCGGACCACAAGCCGCCCAGCCCAAAAGACTTGGTCTACCTAGAACCCTCCCCCGGATTCTGCGAG
AAGAACCCCAAGTTGGGCATACAAGGCACACACGGGCGACAG?TGCAACGACACCTCAATCGGTGTCGACGGT
TGTGACTTAATGTGTTGTGGTCG?CGGTTACCGTACGCAGGAGGTGGTTGTGGTGGAGCGGTGCAACTGCACG
TT???????????

Medon

????GAAGCCCAGCACTGAATCCCGTGGCCGAACCGGGAAATGTAGTGTTTGGGAGGGTCCGCTATCCATCGT
ACGACGCGTCCAAGTCCTTCTTGAACGGGGCCACATACCCATAGAGGGTGCCAGGCCCGATAGCTGGAGGATC
TCTCCTCAGAGTCGGGTTGCTTGAGAGTGCAGCCCTAAGTGGGTGGTAAACTCCATCTAAGGCTAAATATGAC
CACGAGACCGATAGCGAACAAGTACCGTGAGGGAAAGTTGAAAAGAACTTTGAAGAGAGAGTTCAATAGTACG
TGAAACCGTTCAGGGGTAAACCTGAGAAACCCGAAAGGTCGAATGGGGAGATTCAGCGTGTCTCGTTTTCTGG
TCAGTGACGATGGTGTTCGCACCAGGTTGCGCGGTCCGGAGCCGTAACCGGCGACGAACTCGTGCACTTCTCC
TCTAGTAGGACGTCGCGACCCGTTGGGTGCCGGTCTAAGGCCGACGGTGGAGCCTTGGGGTTCCGGCCGGCCC
GCTCGACGGTAAGACAGAGGCGTGGGGTCGCTACGTTAGCGTCCGGCCCGTCACAAGTTCGKGCGACTCGGAT
GTCGGACCTGTGTGCCGACCTCGAGCTCGCCGGCTGTTGGTGACGGTGTCCTCGGACAGACTACACGCCGGTC
GGCGACGCTCTAGCTTTGGGTTTTCAGGACCCGTCTTGAAACACGGACCAAGGAGTCTAGCATGTGCGCGAGT
CATTGGGACCGCATCTAAACCTAAAGGCAAAATGAAAGTGAAGGCGTGCCGAGGGAGGATGGGTCGGGGGGCG
TCTCGTTCTCATCGCGAGATGAGGCGCACCCAGAGCGTACACGC?CTTACACCGTATTCGCTGACTTGTTTGA
?TCCCATTATTGAAGATTACCATACTGGATTTAAGAAAACCGATAAGCACCCTCCCAAGAACTGGGGTGATGT
AAACACCTTCGCCAATCTTGATCCTGCAGGTGAATACGTAGTGTCCACTCGCGTCCGTTGTGGACGCTCCATG
GAAGGTTATCCCTTCAACCCATGCTTAACTGAAGAGCAATACAAGGAGATGGAAACCAAAGTATCTACCACTT
TGTCCGGCCTTGAAGCTGAACTCAAGGGTACTTTCTATCCATTGACTGGAATGGATAAAGATACTCAACAGAA
GCTCATCGATGATCACTTCTTGTTCAAGGAAGGAGATCGTTTTCTCCAGGCTGCTAATGCCTGCCGCTTCTGG
CCCTCTGGACGTGGTATTTACCACAACGACAACAAGACCTTCTTGGTATGGTGCAACGAAGAAGATCATCTTC
GCATCATCTCCATGCAAATGGGTGGCGATCTTGGGGAAGTCTACCGTCGTCTTGTAACTGCTGTTAACGAAAT
CGAGAAACGTGTCCCCTTCTCTCACAATGACAGATTAGGTTTCCTTACTTTCTGCCCAACAAACTTGGGCACT
ACTGTACGCGCCTCTGTACACATCAAAGTACCTAAGCTCGCCGCTAACAAAGCCAAGCTC?????????????

??
??
??
??
??
??
??
??
??
?????????????????????????????????CCTTACATCAAAGAAGTAAATGATGAAGAATTACAAGAACC
CACAGATAAAAGAATGTTTGTTCTTGCAGCAGCTTTAAGAAAAGGTTACACAGTAGATAAACTCTATGATTT?
AACAAAAATAGATCGTTGGTTCTTACAAAAAATGAAGAACATTATAGATTATAATTCCATTCTCGAATTAGTT
CAACAAAATAAATTACAAAATTGCTCAAATATTTATAAGCTGCTATTGAAAGCAAAACAAATTGGCTTTAGTG
ATAAACAAATTGCTGTTGCTGTTAAAAGTACTGAACTTGCAATTAGAAAACAACGCCAGGACTTAGGGATCAC
ACCATATGTTAAACAAATAGATACTGTAGCTGCTGAATGGCCGGCAACTACAAATTATTTATATTTAACATAT
AATGCAGAAAGTCATGATATAACCTTCAATGATCAGCACATGATGGTTATTGGATCAGGTGTTTATAGAATTG
GAAGCTCTGTTGAGTTCGATTGGTGTGCTGTAGGTTGTTTGAGGGAGCTTAGGAAATTAAACAAAAAGACAAT
AATGGTAAATTATAATCCAGAAACTGTTAGTACAGATTATGATATGTCAGATCGATTATATTTTGAAGAAATT
TCATTTGAAGTTGTTATGGATGAAGTATATATTTTAATTTTACCAGGATTTGGAATAATTTCCCATATTATTA
GACAAGCTAGAGGAAAAAAGGAAACTTTTGGTAGACTAGGAATAATTTATGCTATAATAGCAATTGGATTATT
AGGATTTGTAGTATGAGCACATCATATATTTACAGTGGGTATAGATGTAGATACACGGGCCTATTTTACTTCA
GCAACAATAATTATTGCTGTTCCAACAGGAATTAAAATTTTTAGATGATTAGCAACTTTACACGGCTCTCAAA
TTAAATTTACTCCACCAATATTATGATCATTAGGATTTGTTTTTTTATTCACAATTGGGGGTTTGACAGGAGT
TATTTTAGCTAATTCATCAATTGATATTATCTTACATGATACCTATTATGTCGTAGCTCATTTTCATTACGTT
TTATCAATAGGTGCAGTTTTCGCAATTATGGCTGGATTAGTTCAATGATTCCCACTATTTACTGGATTAACAA
TAAATGAATTTTTATTAAAAATTCAATTTTTTATTATATTTACAGGAGTTAATTTAACGTTTTTCCCCCAACA
TTTTCTTGGATTAGCGGGAATACCTCGACGATATTCAGATTACCCTGATGCTTACATACCATGAAATGCTATC
TCATCAATTGGGTCTTTAATCTCAATAACAAGAATTTTTCTTCTATTATTTATTATTTGAGAAAGATTTACTT
CCTTACGAATAAACATTTCAGGGAAAAAATTTTACAACTTCTATTGAATGATTTCAACAATATCCCCCAGCTGA
ACATAGCTATTCTGAATTACCACTAATAACTAAAGATTATGTGGTGGTATTTGATTTCCTCGGT?AAAGATTC
CATTAGATACTACAATGAAGTACCTGTAGAGAAACGTGTCTTCAAAAACCTTCAATTGTTTATGGAAAACAAG
TCGCCAGGAGACGATTTGTTTGATAGATTAAATACAGCTGTGATGAACAAACATTTAAACGAGTTAATGGAAG
GTTTAACTGCCAAGGTGTTTCGTACTTATAACGCTTCGTGGACTCTACAGCAGCAACTTGATAAATTGACCAA
TCCCGATGATTCCATATCCGAAAAAATTTTATCATACAACCGTGCCAACAGAGCGGTTGCTATACTTTGTAAC
CATCAGCGTGCCGTACCCAAAGGCCATCAGAAGTCTATGGAAAAATTAAAAGAGAAAATCGATACAAAAAGGG
ATAATATCAAGGATGCTGAGAGACAAGTTAAAGATGCACAAAGGGATGCCAAACA???CGGAAGCGTCAAGGA
GAAGCAGATATACGACAAGAAAAAGAAAGTGCTGGAGAGACTGAGAGAGCAGTTGGCTAAGTTGGAGATTCAG
GAGACCGACCGTGACGAAAACAAAACTATTGCGTTAGGTACGTCCAAGCTGAACTATTTGGACCCTAGAATCT
CGGTCGCCTGGTGTAAGAAGTTTGATGTGCCCATTGAAAAAATTTATAACAAAACTCAA??????CGCCTACC
ACCCTTCAGAGTAATTGGTGACCATTTAAAGGACCGCTTCGACGGTGCTTCTAGAGTGATGCTGAGCAACTCG
GCGAGCTCCCGAGG????????????AAACGC???GAACCGTC???CAAAGCAGGACAAGCTTTCCAACAGCA
TAGCGTCGAACAGCATCCACAGCAAGAGGGAAAACCGGCCGCGCAAATACAAGTACGGCTTCCAGCTGAAACC
GTACAATCCGGACCACAAGCCGCCGAGCCCCAAGGATCTCGTGTACCTGGAGCCCTCGCCCGGCTTCTGCGAG
AAAAACCCGAAGCTCGGCATCCAGGGCACCCACGGTAGACTG?TGCAACGACACCTCGATTGGAGTGGACGGA
TGCGATTTGATGTGTTGCGGCAG?GGGGTACAGGACCCAGGAAGTCATCGTGGTCGAGAGGTGCAACTGCACG
?????????????

Medonina_Russia

GGGAGAAGCCCAGCACTGAATCCCGTGGCCGACCGGGAAAATGTAGTGTTTGGGAGGGTCCATCATCCATCGT
GCGACGCGTCCAAGTCCTTCTTGAACGGGGCCATATTCCCATAGAGGGTGCCAGGCCCGATAGCTGGCGGATC
TCTCCTCAGAGTCGGGTTGCTTGAGAGTGCAGCCCTAAGTGGGTGGTAAACTCCATCTAAGGCTAAATATGAC
CACGAGACCGATAGCGAACAAGTACCGTGAGGGAAAGTTGAAAAGAACTTTGAAGAGAGAGTTCAATAGTACG
TGAAACCGTTCAGGGGTAAACCTGAGAAACCCGAAAGGTCGAATGGGGAGATTCAGCGTGTCTCGTGTTTGGT
CGCGTGACGATGGTGCTCGCACCGGACTGCGCCTTCTGAATCCGTAACCGGCGGCGAACTCGTGCACTTCTCC
CCTAGTAGGACGTCGCGACCCGTTGGGCGCCGGTCTACGGCCGACGGTGGAGCCTTGGGGTCCCGGCCGGCCC
GCTCGACGGTAAGACAGAGACGTGGGGTCGCGACGTTCGCGTCCGGCCCGTCACAAGTGCGTGCGACTCGGAC
GTCGGACCTGTGTGCCGACCTCGAGCTCGCCGACTGTTGGTGACGGTGTCCTCGGACAGACTACACGTCGGTC
GGCGACGCTTTAGCTTTGGGTTTTCAGGACCCGTCTTGAAACACGGACCAAGGAGTCTAGCATGTGCGCGAGT

CATTGGGACCGCATCTAAACCTAAAGGCGAAATGAAAGTGAAGGCGTGCCGAGGGAGGATGGGTCGGGGGGCG
TCTCGTTCTCATCACGA???????????????????????????TCTTACACCGTATTCGCTGATTTGTTCGA
?CCCCATCATCGAAGACTACCATGGTGGTTTCAAGAAGACCGACAAGCACCCCCCTGCAAACTGGGGTGATGT
AAACACCTTTGCTAATCTCGACCCTGCTGGTGAATACGTTGTATCCACCCGCGTTCGTTGCGGTCGTTCAATG
GAAGGCTATCCCTTCAACCCTTGCTTAACCGAAGACCAATACAAGGAGATGGAACAGAAGGTTTCATCCACCT
TGTCCGGCCTCGAAGGCGAACTCAAGGGTACCTTCTACCCATTGACCGGCATGAGCAAGGAGGTTCAACAGAA
GCTCATTGATGATCACTTCTTGTTCAAGGAGGGCGATCGTTTCCTCCAGGCTGCCAACGCTTGCCGTTACTGG
CCAAGCGGACGTGGTATTTACCACAACGACAACAAAACCTTCTTGGTTTGGTGCAACGAAGAGGATCATCTTC
GCATCATCTCTATGCAAATGGGTGGAGATCTTGGCGAGGTATACCGTCGTCTTGTAACCGCCGTCAACGAAAT
TGAGAAGCGCGTCCCCTTCTCCCATAACGACAGATTAGGTTTCCTTACCTTCTGCCCAACCAACTTGGGCACA
ACTGTACGTGCCTCTGTACACATTAAAGTACCTAAGCTCGCCGCCAACAAAGCCAAGCTTGATGAAATTGCTG
CCAAATACAACTTGCAAGTACGTGGTACCCGT???GTTCCAAAAAAAGTTTTAATAATTGGTTCTGGTGGATT
ATCAATTGGTCAAGCCGGAGAATTTGATTATTCTGGTTCACAAGCAATTAAAGCTTTACACGAAGAAAATATT
CAAACTGTTCTGATAAATCCTAACATTGCTACTGTGCAAACATCAAAAGGTTTAGCTGATAAAGTATATTTTT
TACCTTTAGTGCCTGAATTTGTAGAACAAGTAATTAGAGTTGAACGACCTGGTGGTGTTTTATTAACTTTTGG
TGGGCAAACTGGATTGAATTGTGGTGTTGAATTGCAGAGAGCTGGTATATTTGAAAAATATGGTGTTAAAATT
TTGGGAACTCCAATAGAAGCCATAATAGATACTGAAGATCGAAAAGTTTTTAGTGATAGAATATCACTAATTG
GCGAGAAGGTAGCACCAAGTATGGCTGCTTATTCAGTTCAAGAAGCTTTAGAGGCTGCTGAATTATTAGGTTA
TCCGGTAATGGCAAGAGCAGCATTCTCACTAGGCGGTTTGGGATCTGGCTTTGCTAATACATCCGATGAATTG
AAATCACTTGCTCAACAAGCATTAGCTCACTCAAACCAATTAATTATAGATAAATCATTAAAAGGATGGAAAG
AAG??
???
?????????????????GGTTTTTACAAAAAATGAAAAATATTGTTGATTTTAATACCTATTTAGAATCAATT
???CAAAACAAGTTA???????????????ACGTATAAAGATTTATTAAAAGCAAAACAAATTGGTTTCAGTG
ATAAGCAAATTGCTGTAGCTGTTAAAAGCACTGAGCTTGCAATTAGAAAACACCGACAAGATTTTGACATTAC
TCCTTATGTGAAACAAATAGATACTGTAGCTGCTGAATGGCCTGCAACAACAAATTATCTATATTTGACTTAC
AATGCTGAAAGTCATGATTTAACTTTCTCAGATGACCATATTATGGTAATTGGTTCGGGTGTATATAGAATTG
GAAGTTCTGTGGAATTCGATTGGTGCGCAGTCGGTTGTTTAAGAGAACTTAGAAAGTTAAATAAAAAAACAAT
AATGGTCAATTATAATCCTGAAACAGTTAGTACTGATTACGATATGTCTGACAGATTATATTTCGAAGAAATT
TCGTTTGAGGTTGTAATGGAT??????????????????????????????????????TTCTCATATTATTA
GTCAAGCTAGAGGAAAAAAGGAAACTTTTGGAAGATTAGGAATAATTTATGCCATAATAGCTATTGGTCTTTT
AGGTTTTGTAGTGTGAGCTCATCATATATTTACTGTTGGGATAGATGTAGATACCCGAGCCTACTTTACTTCA
GCAACTATAATTATTGCTGTTCCTACAGGGATTAAAATTTTTAGATGGTTAGCAACATTACATGGAACTCAAA
TTAAATTTACTGCCCCTATATTATGAGCACTGGGATTTGTATTTTTATTTACTATTGGAGGATTAACCGGAGT
AATTTTAGCTAACTCATCAATTGATATTATCCTCCATGATACATATTATGTAGTAGCCCATTTCCATTATGTA
TTATCAATAGGGGCGGTATTCGCCATTATAGCCGGATTAGTGCAATGATTCCCTTTATTTACAGGATTAATAA
TAAATGAATACCTATTAAAAATTCAATTTTTCATTATATTTATTGGAGTAAATTTAACATTTTTCCCTCAACA
TTTTTTAGGGTTAGCAGGAATGCCACGACGGTATTCTGATTACCCAGATGCCTATACACCATGGAATGTAATT
TCTTCAATTGGCTCATTAATCTCTATAATTAGAATTTTTCTATTAGTATTTATTTTCTGAGAAAGATTTTCTT
CAATACGAATAATTATTTCTCCTAAAAATCTTTGTACATCAATCGAATGATTTCA??????????????????
???????????????????????????????AAAGATTACGTAGTAGTATTTGATTTCCTCGGT?AAAGATTC
CATTAGATATTATAATGAAGTACCTGTGGAGAAACGAGTTTTCAAGAACCTCCAATTATTTATGGAAAATAAA
GCTCCAGGAGATGATTTGTTCGATAGATTAAATACAGCTGTGATGAACAAACATTTAAACGAATTAATGGAAG
GCTTAACCGCCAAAGTGTTTCGTACTTATAACGCTTCTTGGACCCTACAGCAGCAACTCGAAAAACTGACCAA
TCCAGACGATTCCTTATCCGAAAAAATCCTCTCGTACAACCGTGCCAATCGAGCGGTTGCCATTCTCTGTAAC
CATCAGCGTGCTGTACCCAAAGGCCATCAGAAATCCATGGAAAAACTAAAAGAGAAAATCGAAGCTAAAAAAG
AATCCATACGTGACGGCGAAAGGCAAGTGAAGGATGCACAGAGGGATGCGAAGCA???TGGCAGCGTTAAAGA
GAAACAGATCTACGACAAGAAAAAGAAGATGCTAGAGAGACTAAGAGACCAATTAGCTAAATTAGAGATTCAG
GAGACGGACCGTGATGAAAATAAAACGATTGCTCTCGGCACGTCGAAGCTGAACTATTTGGATCCTAGGATCT
CGGTTGCCTGGTGTAAGAAGTTTGATGTGCCCATTGAAAAGATTTATAATAAAACTC?????ATGCGG?CGCC
GCCGTTCCGCGTCATAGGCGACCACCTAAAGGACCGCTTCGACGGCGCGTCGCGCGTCATGCTCAGCAACTCG
GCGAGCTCGCGGGG??????????GGCAACGC???GAACCGTC???CAAAGCAGGACAAACTCTCGAACAGCA
TCGCCTCGAACAGCATACACAGCAAGCGCGAGAACCGGCCGCGGAAGTACAAGTACGGTTTTCAGTTGAAACC
GTACAACCCGGACCACAAGCCGCCGAGCCCCAAGGACCTGGTGTACCTGGAGCCGAGTCCTGGATTCTGTGAG
AAGAATCCGAAGCTGGGGATACAGGGAACGCACGGGAGGCAG?TGTAATGATACATCGATTGGGGTCGATGGT
TGCGATTTGATGTGTTGCGGAAA??GGGTATAGAACGCAGGAGGTTATTGTTGTTGAAAGGTGT?????????
?????????????

Neolindus

GGGAGAAGCCCAGCACTGAATCCCGCGGCCGGGCCGGGAAATGTAGTGTTTGGGAGGATCCGCCATCCATCGC
ACGACGCGTCCAAGTCCTTCTTGAACGGGGCCACATACCCGCAGAGGGTGCCAGGCCCGGTAGCCGGAGGATC
TCTCCTTAGAGTCGGGTTGCTTGAGAGTGCAGCCCTAAGCGGGTGGTAAACTCCATCTAAGGCTAAATATGAC
CACGAGACCGATAGCGAACAAGTACCGTGAGGGAAAGTTGAAAAGAACTTTGAAGAGAGAGTTCAATAGTACG
TGAAACCGTTCAGGGGTAAACCTGAGAAACCCGAAAGGTCGAACGAGGAGATTCAGCGTGTTCCGTTCCGTGG
CGAGTGACGGTGGCGTTCGCGCCGTGCCGCGCCGTCTAGGTCCGCCGCCGACGACGGACTCGTGCACTTCTCC
TCCAGTAGGACGTCGCAACCCGTTGTGTGTCGATCTACGGCCGACGGTGGAGCCTTGGCGTCCCGACCGGCTC
GCACGACGGTAGCGCGATGACGTGGGGTCGCGACGTTCGCGTCCGGCCCGTCACGAGCTCGGGCGACTCGAGC
GTCGGACCTGCGTGCCGACCTCGGTCTCGCCGGCTGCCGGTGACGGTGTCCTCGGACCGGCTACACGTCTGTT
GGCGACGCTTTAGCTTTGGGTTTTCAGGACCCGTCTTGAAACACGGACCAAGGAGTCTAGCATGTGCGCGAGT
CACTGGGAACT?AACTAAACCCACAGGCGAAATGAAAGTGAAGGCGCGCCGAGGGAGGATGGGTCGGGGGGCG
TCTCGTTCTCATCGCGAGATGAGGCGCACCCAGAGCGTACACGC?????????????????????????????
???
???
???
???
???
???
???
???
???
???
???
???
???
???
???
???
???
???
????????????????????????????????CCGTA?TCTAAAAAAGTAAACGATGAAGAATTAAAAGAGCC
AACTGATAAGAGAATGTTTGTCGTTGCTGCTGCGTTAAAATCTGGTTACAGCGTAGAAGAATTATATGACTT?
AACGAAAATTGATTCTTGGTTCTTGCAAAAAATGAAGAATATCATTGATTTCATAACATTTCTAGAAGTGA??
?ATCAAAATCATTA????????????????ACACCAAAAATTTTATTACAAGCTAAACAAATGGATTCAGTG
ACAAACAAATTGCTGCCGCTGCTGAAAGTACTGAACTTGCTATAAGGAAACAACGGCAAGATTATAATATAAC
ACCTTTCGTTAAGCAGATTGACACTGTCGCTGCAGAATGGCCTGCAACAACAAATKATTTATATTTGACTTAC
AATGCAGGAAGTCACGATTTGACTTTCGATGAWGAGCATGTAATGGTTATTGGATCTGGAGTTTATCGTATCG
GAAGTTCTGTTGAATTTGATTGGTGTGCAGTWGGATGTTTACGTGAGCTTAGAAAAT?GAATAAAAAAACAAT
TATGGTAAATTACAATCCAGAAACCGTGAGTACAGATTACGATATGTCAGATAGATTATATTTGAAGAAATT
TCATTTGAAGTTGT???????GAAGTTTATATTCTAATTTACCTGGATTCGGAATAATCTCTCATATTATTA
TACAAAGAAGAGGAAAAAAAGAAACTTTTGGAACCTTAGGAATAATTTATGCAATAATAGCAATTGGATTATT
AGGATTTGTAGTTTGAGCTCATCATATATTTACAGTAGGAATGGATGTAGATACTCGAGCATATTTTACCTCC
GCTACTATAATTATTGCTATCCCTACAGGAATCAAAATTTTTAGATGATTAGCTACTTTTCATGGAACACAAA
TATCATTTAACCCCCCAATAATTTGAGCTTTAGGATTTGTATTTTTATTTACAGTAGGAGGATTAACTGGAGT
TATTTTAGCTAATTCATCTATAGATATTATTCTACATGATACTTACTATGTTGTAGCACATTTCCATTATGTT
TTATCTATAGGAGCAGTATTTGCAATTATAGCAAGTCTAACTCAATGATTTCCTTTATTTACAGGACTATCTT
TAAATGAATTTATACTAAAAATTCAATTTACAATTATATTTATTGGAGTTAATTTAACATTTTTCCCTCAACA
TTTTCTAGGATTAGCAGGAATACCTCGTCGATATTCAGACTACCCTGATTCCTTTACTACTTGAAATGTAATT
TCATCAATAGGCTCATTAATTTCATTAATCAGAATTTTTATTATATTATTTATTATTTGAGAAAGATTAACTT
CTAAACGATTAATTTTATCCTCAAAAAATTTCTCTACTTCTATTGAATGATTACAATTTTT????????????
???????????????????????????????AAAGATTATGTCGTGGTTTTCGATTTTCTTGGC?AAAGATTC
CATTAGATATTATAATGAAGTACCTGTAGAAAAACGTGTCTTCAAAAATCTCCGATTATTCATGGAAAATAAA
TCGCCAGGCGATGATCTGTTTGACAGATTGAACACAGCTGTAATGAACAAACATTTAAACGAGTTAATGGAGG
GATTAACCGCGAAGGTATTTCGTACTTACAACGCATCGTGGACCTTACTACCGAAACATGAGAAATTGACCAA
TGCWGATGATTCCATATCTGAAAAAATACTGTCGTATAMTCGTGCTWATCGTGCTGTGGCAATYTTATGTAMC
CATCAACGTGCAGTACCTAAGGGGCWTCARMAATCTATGGAAAAGCTGAAAGAGAAGATTGAAGCGAAAAAAG

AAGCAATAAAAGATGGTGAAAGGCAAGTAAAAGATGCGCAACGGGATGCAAAACA???CGGTAGTGTAAAAGA
AAAGCAAATTTACGATAAAAAGAAGAAACAGTTGGAACGACTTCGCGAACAACTTGCAAAATTAGAAATTCAA
GAGACAGACAGAGATGAAAACAAGACTATTGCACTTGGCACGTCCAAATTGAATTATTTGGACCCAAGAATCT
CTGTAGCTTGGTGTAAGAAATACGATGTGCCCCTTGAAAAGATTTATAATAAAACTCAA???ATGCGCCTTCC
TCCATTCAGGGTAATCGGAGATCAACTTAAGGATCGCTTCGATGGAGCATCGAGAGTCATGCTGAGTAATTCA
GCCAGCTCAAGAGGA?????????GGTAACGC???AAATCGTC???CAAAACAAGACAAACTATCCAACAATA
TAGCATCGAATAGCATTCATAGCAAACGCGAAAATAGACCGCGTAAATATAAATACGGCTTCCAACTTAAACC
CTATAACCCAGACCACAAACCACCAAGTGCGAAAGATTTGGTATACTTAGAACCATCCCCGGGATTTTGTGAA
AAAAATCCRAAGTTAGGAATCCAAGGGACGCATGGCAGGCAA?TGCAACGACACATCGATTGGAGTCGATGGG
TGCGATTTGATGTGCTGTGGAAG?AGGTTATAGGACACAAGAGGTAGTTGTTGTTGAAAGATGCAACTGCACC
TTCC?????????

Neosclerus

GGGAGAAGCCCAGCACTGAATCCCGTGGCCGAACCGGGAAATGTAGTGTTTGGGAGGGTCCGCTATCCATCGT
GCGGCGCGTCCAAGTCCTTCTTGAACGGGGCCACATACCCATAGAGGGTGCCAGGCCCGATAGCTGGAGGATC
TCTCCTCAGAGTCGGGTTGCTTGAGAGTGCAGCCCTAAGTGGGTGGTAAACTCCATCTAAGGCTAAATATGAC
CACGAGACCGATAGCGAACAAGTACCGTGAGGGAAAGTTGAAAAGAACTTTGAAGAGAGAGTTCAATAGTACG
TGAAACCGTTCAGGGGTAAACCTGAGAAACCCGAAAGGTCGAATGGGGAGATTCAGCGTGTTTAGTTTCTGGT
CGAGTGACGGTGGTGCTAGCACCGGGCTGCGCCGGCCGGGACCGCAGCCGGCGACGAACTCGTGCACTTCTCC
TCTAGTAGGACGTCGCGACCCGTTGGGCGCCGGTCTAAGGCCGACGGAGGAGACTTGGGGTCCCGGCCGGCCC
GCTCGACGGTGAGACAGAGGCGTGGGGTCGCTACGTTAGCGTCCGGCCCGTCACAAGTTCGGGCGACTCGGAT
GTCGGACCTGCGTGCCGACCTCGAGATCGCCGGCTGCTGGTGACGGTGTCCTCGGACAGACTACACGCCGGTC
GGCGACGCTCTAGCTTTGGGTTTTCAGGACCCGTCTTGAAACACGGACCAAGGAGTCTAGCATGTGCGCGAGT
CATTGGGACCGCATCTAAACCTAAAGGCGAAATGAAAGTGAAGGCGTGCCGAGGGAGGACGGGTCGGGGGGCG
TCTCGTTCTCATCGCGAGATGAGGCGCACCCAGAGCGTACACGC?????????????????????????TTCGA
?TCCCATTA?TGAAGATTACCATGGTGGATTCAAAAAGACCGACAAGCACCCCCCAAAGAACTGGGGTGATGT
AAACACTTTCGGCAACCTTGATCCAGCTGGTGAATACGTAGTCTCCACCCGTGTCCGTTGCGGGCGCTCCATG
GAAGGCTACCCCTTCAACCCATGCTTAACCGAAGAACAATACAAGGAGATGGAAGGCAAAGTTTCCAGCACTC
TGTCTGGCCTTGAAGCTGAACTTAAGGGTACTTTCTATCCCTTGACTGGAATGGACAAAGATACTCAACAGAA
GCTCATTGACGATCACTTCTTGTTCAAAGAAGGTGATCGTTTCCTCCAAGCTGCCAACGCTTGCCGTTTCTGG
CCATCTGGACGTGGTATTTACCACAACGACAACAAAACCTTCTTGGTATGGTGCAACGAAGAAGATCATCTTC
GTATCATCTCTATGCAAATGGGTGGTGATCTTGGTGAAGTCTACCGTCGCCTTGTAACAGCTGTCAACGAAAT
CGAGAAGCGTGTTCCCTTCTCCCATAATGACAGATTAGGTTTCTTGACCTTCTGCCCAACCAACTTGGGCACA
ACTGTACGTGCCTCTGTACACATTAAAGTACCTAAGCTCGCCGCCAACAAGGCCAAACTTGATGAGGTCGCTG
CCAAATACAACTTGCAAGTACGTGGTACTCGCGGTGTACCTAAAAAAGTCTTAATAATTGGGTCAGGTGGATT
GTCAATTGGACAAGCTGGAGAATTCGATTATTCAGGTTCACAAGCAATAAAAGCTTTACAAGAAGAAAATATT
CAAACTGTTCTAATTAATCCAAATATTGCTACAGTACAAACTTCAAAAGGTTTAGCTGATAAAATATACTTTT
TACCTTTAGTGCCTGAATTTGTAGAACAAGTAATTAGAGTAGAACGTCCTGGAGGCGTATTGTTAACATTTGG
TGGACAAACAGGGTTAAATTGTGGTGTGGAATTACAAAAAGCTGGTGTATTTGAAAAATACAATGTTCAAATT
TTGGGCACACCTATAGAAGCAATAATACACACCGAAGATAGAAAGATTTTTAGTGACAGAATAGCATTAATTG
GAGAGAAAGTTGCTCCGAGTATGGCTGCATATTCTGTACAAGAAGCTTTGGATGCAGCAGATTTATTAGGGTA
TCCAGTTATGGCACGAGCAGCATTTTCTCTGGGTGGATTAGGATCTGGTTTTGMTGATACAGATGAAGAATTG
AAATTACTTGCTCAACAAGCTTTAGCTCATTCTAATCAGTTAATTATTGATAAGTCTTTAAAAGGGTGGAAAG
AAGTTGAATATGAAGTTGTT????????????CCTAACATAAAAGAAGTCAATGATGATGAACTAAAGGAACC
TACAGATAAAAGAATGTTTGTTATTGCAGCAGCTTTAAAAAATGGTTATAGTGTTGATAAATTATATGATCT?
AACAAAAATCGATCGTTGGTTTTTACAAAAGATGAAAAATATTATAGATTACAATTCCCGTTTGGAATTAGTT
CAACAAAATAAATTACAAAATTGCTCAAATATTTACAAGCTGTTATTAAAGGCCAAACAAATTGGTTTTAGTG
ATAAGCAAATTGCTGTTGCTGTTAAAAGTACTGAACTTGCAATTCGAAAGCAAAGACAGGATTTAGGAATTAC
TCCATATGTTAAACAAATTGATACAGTAGCTGCTGAATGGCCTGCAACCACAAATTATTTGTATTTAACGTAC
AATGCTGAAAGTCACGATCTAACTTTTAATGATCAACACATAATGGTTATTGGTTCGGGAGTTTATAGAATTG
GGAGTTCTGTTGAGTTTGATTGGTGTGCTGTTGGTTGTTTAAGAGAACTTAGAAAGTTAAATAAAAAGACAAT
AATGGTCAATTACAATCCAGAAACTGTTAGTACAGATTATGATATGTCAGATAGGCTATATTTTGAAGAAATT
TCATTTGAAGTTGTTATGGATGAAGTTTATATTTTAATCCTCCCTGGATTTGGTATAATTTCCCACATTATTA
GACAAGCTAGAGGAAAAAAAGAAGCTTTTGGAGCTTTAGGAATAATTTATGCAATAATAGCTATTGGTTTATT
AGGATTTGTTGTTTGGGCTCACCATATATTTACTGTTGGAATAGACGTAGATACTCGAGCTTATTTCACCTCA
GCAACTATAATTATTGCTGTCCCTACAGGAATTAAAATTTTTAGATGATTAGCAACTTTACATGGAACACAAA
TTAAATACACCCCCCCTATATTATGGGCTTTAGGATTTGTTTTTTTATTTACAATCGGAGGATTAACTGGTGT
TATCTTAGCAAATTCATCTATTGATATTATTTTACATGATACATATTATGTAGTAGCTCATTTTCATTATGTT

TTATCAATAGGGGCTGTATTTGCTATTATAGCCGGATTAGTGCAATGATATCCCTTATTTACTGGATTAACAC
TAAATGAATACTTATTAAAAATTCAATTTTTAACTATATTTATTGGAGTAAATTTAACTTTTTTTCCTCAGCA
TTTCCTAGGATTAGCGGGAATACCTCGACGATATTCCGATTATCCAGACGCCTATACTCCTTGAAATATAGTA
TCTTCTATAGGATCATTGATTTCAATAAATAGAATTTTTTTACTGTTATTTATTATTTGAGAAAGAATAATTT
CTTTACGAATTAATATTTCAGCAAAAAATTTTCCATCATCAATTGAATGAATACAACTGTTTCCTCCAGCAGA
ACATACATATTCAGAATTACCAATTTTAATTAAAGATTATCTAGTAGTGTTTGATTTTCTTGGT?AAGGATTC
CATTAGATATTACAATGAAGTACCTGTAGAAAAACGTGTTTTCAAAAACCTTCAATTGTTTATGGAAAACAAA
TCTCCTGGTGATGATTTGTTTGATAGATTAAACACAGCTGTGATGAACAAACATTTAAACGAGTTAATGGAAG
GTTTAACAGCAAAGGTGTTTCGTACTTATAACGCTTCTTGGACTCTACAGCAGCAACTTGATAAATTGACCAA
TCCAGATGATTCCATATCCGAAAAAATTTTATCATACAATCGTGCTAATAGAGCAGTAGCAATACTTTGTAAC
CATCAACGTGCTGTACCTAAAGGCCATCAGAAATCTATGGAAAAACTCAAAGAAAAAATTGGAGCTAAAAGGG
AAACTATCAAAGATGCTGAGAGGCAAGTTAAAGATGCACAAAGAGATGCTAAACA???TGGAAGTGTTAAGGA
GAAGCAGATCTATGATAAAAAGAAGAAAATGTTGGAAAGACTACGTGAGCAATTGGCCAAGTTGGAAATTCAA
GAAACCGACCGTGATGAAAATAAAACTATTGCACTTGGTACGTCCAAGCTGAACTATTTGGATCCTAGAATCT
CGGTCGCTTGGTGTAAGAAGTTTGGTGTGCCCATTGAAAAAATTTATAACAAAACTCAATGGATGCGTTTGCC
ACCCTTCAGGGTAATTGGTGATCATTTAAAAGATCGCTTCGATGGTGCTTCTAGAGTAATGTTGAGCAATTCA
GCTAGTTCAAGAGG????????????GAACGC???TAATCGTC???CAAAACAAGATAAACTTTCGAATAGTA
TAGCATCGAATAGTATACATAGTAAAAGAGAAAACAAGCCGCGCAAGTATAAATATGGTTTTCAACTGAAACC
GTACAATCCGGACCATAAACCTCCAAGTCCTAAAGATTTAGTGTACCTGGAACCTTCCCCTGGTTTTTGCGAG
AAAAACCCAAAGCTCGGCATACAAGGTACTCATGGTAGATTG?TGCAACGATACTTCTATCGGCGTGGATGGA
TGCGATTTGATGTGTTGCGGTAG?AGGATACAGAACCCAGGAAGTCATCGTTGTTGAAAGGTGCAACTGTACG
TTCCA????????

Notobium

GGGAGAAGCCCAGCACTGAATCCCGTGGCCGAATCGGGAAATGTAGTGTTTGGGAGGATCCGTCATCCACCGT
ACGACGCGTCCAAGTCCTTCTTGAACGGGGCCACATACCCACAGAGGGTGCCAGGCCCGGTAGCCGGAGGATC
TCTCCTCAGAGTCGGGTTGCTTGAGAGTGCAGCCCTAAGTGGGTGGTAAACTCCATCTAAGGCTAAATATGAC
CACGAGACCGATAGCGAACAAGTACCGTGAGGGAAAGTTGAAAAGAACTTTGAAGAGAGAGTTCAATAGTACG
TGAAACCGTTCAGGGGTAAACCTGAGAAGCCCGAAAGGTCGAATGGGGAGATTCAGCGTGTCACGTTTCGGGT
CGAGTGACGGTGGTGCGTGCACTGGGCTGCGCCGTCCGGATCCGAAACCTGCGGCGAACTCGTGCACTTCTCC
CCTAGTAGGACGTCGCGACCCGTTGGGTGCCGGTCTAAGGCCAGCGGTGGAGCCTCAAAGTCCCGGCCGGCCC
GCTCGACGGTAAGACAGAGACGTGGGGTCGCGATGTTCGCGTCCGGCCCGTCACAAGCATGCGCGACTCGGAC
GCCGGACCTGTGTGCCGACCCCGAGCTCGCCGGCTGTTGGTGGCGGTGTCCTCGGACAGACTACACGTCGGTC
GGCGACGCTTTAGCTTTGGGTTTTCAGGACCCGTCTTGAAACACGGACCAAGGAGTCTAGCATGTGCGCGAGT
CATTGGGACCGCATCTAAACCTAAAGGCGAAATGAAAGTGAAGGCGCGCCGAGGGAGGATGGGTCGGGGGGCG
TCTCGTTCTCATCGC???????????????????????????????CTTACACCGTATTCGCTGATTTGTTCGA
?CCCCATTATTGAGGATTACCATGGTGGTTTCAAGAAGACCGACAGCCATCCCCCAAAGAACTGGGGCGATGT
CAACACTTTCGCCAACCTCGACCCTGCGGGTGAGTACGTTGTGTCAACCCGCGTTCGCTGCGGCCGCTCCATG
GAGGGTTACCCGTTCAACCCCTGCTTAACCGAAGATCAATACAAGGAGATGGAACAGAAAGTTTCGTCCACTT
TGTCCGGGCTCGAGGGTGAACTCAAGGGTACCTTCTACCCGTTGACTGGAATGGACAAGGATACTCAGCAGAA
ACTCATCGATGATCACTTCTTGTTCAAGGAGGGCGATCGTTTCCTCCAGGCTGCTAACGCTTGCCGTTTCTGG
CCGTCCGGACGTGGCATCTACCACAACGACAACAAGACGTTCTTGGTCTGGTGCAACGAAGAGGATCACCTCC
GTCTTATTTCCATGCAAATGGGTGGGGATCTTGGAGAGGTTTACCGCCGTCTCGTTACCGCAGTCAACGACAT
TGAGAAACGCGTTCCTTTCTCGCATAACGACAGATTAGGTTTCCTCACTTTCTGCCCGACCAATTTGGGTACA
ACTGTACGTGCCTCGGTACACATTAAAGTACCAAAGCTCGCTGCCAACAAGGCCAAACTT?????????????
???????????????????????????????????GTTCCAAAAAAAGTTTAATAATTGGTTCAGGTGGTTT
ATCAATTGGGCAAGCAGGAGAATTCGATTATTCCGGTTCCCAGGCAATTAAAGCACTACAAGAAGAAAATATA
CAAACTGTTTTAATCAATCCTAACATTGCAACAGTACAAACCTCTAAAGGTTTAGCTGATAAAGTATATTTCT
TACCTTTGGTCCCAGAATACGTAGAAAAAGTAATTAGAGTAGAAAGACCTGGAGGCGTCTTGTTAACGTTTGG
AGGTCAGACTGGATTAAATTGTGGTGTAGAACTTCAAAAGGCTGGAGTTTTTGAAAAATATGGTGTTAAAATA
TTAGGTACCCCTATACAAGCGATAATAGATACTGAAGATAGAAAAATTTTCAGTGATAGAATTTCATTAATTG
GAGAAAAGGTAGCTCCAAGTATGGCTGCGTATTCTGTACAAGAAGCTTTGGAAGCAGCAGAGTTGTTGGGGTA
TCCTGTTATGGCGAGGGCTGCGTTCTCATTAGGGGGACTAGGATCAGGATTCGCTAACACAACTGATGAACTT
AAGTCACTTGCACAACAAGCACTAGCACATTCCAATCAATTAATTATTGATAAATCGTTGAGAGGATGGAAGG
AAGTTGAGTATGAGGTTGTAAGAGACGCATATCCATATCTCAAACAAGTAAATGATGAGGAATTAAAAGAACC
TACAGACAAACGGATGTTTGTTGTCGCTGCTGCATTGAGAAGTGGTTACAGTGTAGACAAATTATATGATTT?
AACAAAAATTGATCGTTGGTTCTTACAAAAAATGAAAAATATAATTGATTTTAATACCATATTGGAATCCATT
CAGCAAAATAAATTA???????????????ACTGCAAAAGTTTATTGAAAGCAAAACAAATAGGATTTAGTG

ACAAACAAGTCGCTGCAGCGGTTAAAAGTACAGAACTTGCAATACGAAAGCAACGCCAAGATTTTAATCTTAC
TCCTTATGTCAAACAAATTGATACTGTGGCTGCTGAGTGGCCTGCCACTACCAATTACCTATATTTAACATAT
AACGCAGAAAGTCATGATTTGAATTTTGCTGAAGAACATATAATGGTTATTGGATCTGGAGTGTACAGAATAG
GAAGTTCAGTTGAATTTGATTGGTGTGCTGTAGGATGTTTGAGAGAGCTAAGAAATTTAAATAAAAAAACAAT
TATGGTAAATTATAATCCAGAAACTGTTAGCACGGATTATGATATGTCGGATAGATTGTATTTTGAAGAAATT
TCATTTGAAGTAGTTATGGACGAAGTCTATATCTTATTTTTACCTGGATTTGGTATAATTTCTCATATTATTA
GACAAAGTAGAGGTAAAAAAGAAACATTTGGTTCTCTTGGAATAATTTATGCAATAATAGCTATCGGATTATT
AGGATTTGTAGTATGAGCTCATCATATATTTACAGTAGGCATAGATGTTGATACTCGAGCTTATTTTACTTCA
GCAACAATAATTATTGCTGTTCCTACAGGAATTAAAATTTTTAGATGATTAGCTACTCTACATGGAACTCAAA
TTAATTTTAATCCTCCTATTTTATGAGCTTTAGGTTTTGTATTTTTATTTACTATTGGGGGATTAACTGGTGT
AATTTTAGCTAATTCTTCTATTGATATTGTATTACATGATACTTATTATGTAGTTGCTCATTTTCATTATGTT
CTTTCTATAGGAGCTGTATTTGCAATTATAGCAGGTTTAGTTCAATGATTCCCTCTATTCACAGGATTAACTA
TAAATAAAAAATATTTAAAAATTCAATTTTTAGTAATATTTATTGGAGTAAATTTAACTTTTTTCCCTCAACA
TTTTCTTGGTCTAGCAGGAATACCACGACGTTATTCTGACTACCCAGATACTTTTATGCCTTGAAATATAATC
TCATCCATTGGTTCATTAATCTCTTTAATTAGAATTTTATATTTTTTATTTATTATTTGAGAAAGATTAGCCT
CTATACGACAAATTTTATCCTCTAAAAATTATTCTACTTCAATTGAATGATTCCAACTATTTCCCCCTGCCGA
GCATAGTTATTCAGAGCTACCAGCTCTATCCAAGGATTACGTTGTAGTGTTTGATTTCCTCGGT?AAAGATTC
CATTAGATATTATAATGAGGTACCTGTAGAAAAACGTGTCTTCAAAAATCTCCAGTTATTTATGGAAAGTAAG
GCGCCTGCTGATGATTTATTCGACCGATTGAACACTACTGTGATGAATAAACATTTAAATGAATTAATGGAGG
GTTTAACCGCCAAGGTATTTCGTACTTACAATGCTTCATTTACGTTACAACAACAGCTCGACAAACTGACCAA
TGCCGACGATTCCATATCCGAGAAAATCTTATCGTATAACCGTGCCAATCGAGCTGTGGCCATCTTGTGTAAC
CATCAACGTGCTGTGCCGAAGGGCCATCAGAAATCGATGGAGAAATTGAAGGAGAAAATCGATGCCAAGAGAG
AAGCTATAAAAGATGGCGAACGGCAAGTTAAGGACGCGCAGAACGACGCGAAACG???CGGCAGCGTGAGGGA
AAAGCAGATCTATGATAAGAAGAAGAAAATGTTGGAGAGGCTTAGAGACCAGCTTAACAAGTTGGAAATTCAG
GAGACTGATCGCGATGAAAACAAGACAATCGCGCTTGGCACATCCAAACTAAATTATTTGGATCCGAGGATTT
CCGTCGCCTGGTGTAAGAAATACGATGTGCCCATTGAAAAAATCTATAATAAAACTCAA??????CGGCTGCC
ACCGTTCAGGGTCATCGGGGACCACCTCAAGGACCGCTTCGACGGCGCCTCCAGGGTGATGCTGAGCAACTCG
GCGAGCTCGCG??????????????GAACGC???GAACCGCC???CGAAACAGGACAAGCTGTCGAACAGCA
TAGCGTCGAACAGCATACACAGCAAGCGTGAGAACAGGCCGCGCAAGTACAAGTACGGGTTCCAGCTGAAGCC
GTACAACCCGGACCACAAGCCGCCGAGCCCGAAGGACCTGGTGTACCTGGAGCCATCGCCGGGGTTCTGCGAG
AAGAACCCGAAGCTGGGGATACAGGGCACGCACGGCAGGCAG?TGCAACGACACGTCGATTGGTGTGGACGGC
TGCGATCTGATGTGCTGCGGGAG?GGGCTACAGGACCCAGGAGGTCATCGTCGTGGAGAGGTGCAACTGCACG
?????????????

Ochthephilum

GGGAAGAGCCCAGCACCGAATCCCGCGGCCGAGCCGGGAAATGTGGTGTTAGGGAGGGTCCGCTATCCGTCGC
GCGGCGCGTCCAAGTCCTTCTTGAACGGGGCCACTTACCCAAAGAGGGTGCCAGGCCCGATCGCGGGAGGATC
TCTCCTCAGAGTCGGGTTGCTTGAGAGTGCAGCCCTAAGTGGGTGGTAAACTCCATCTAAGGCTAAATATAAC
CACGAGACCGATAGCGAACAAGTACCGTGAGGGAAAGTTGAAAAGAACTTTGAAGAGAGAGTTCAATAGTACG
TGAAACCGTTCAGGGGTAAACCTGAGAAATCCGAAAGATCGAATGGGGACATTCAGCGCGTCTCGCCTCAGGA
CGCGAGACGATGGCGTTCGCGCTCTGTCGCTCCTTCCGAAGCCGCGACCGCTGGCGAACGCGTGCACTTCTCC
CCTAGTAGGACGTCGCGACCCGTTGGGTGTCGGTCTAAGGACCGCGGTGGAGCCCGCCGGTCCCGACCCGCTC
ACTCGACGGTAAAACGGTGGCGAAGGGTCGCGAGGTTCGCGCCCGGCCCGTCGCAAGCGTTACCGTCCCGGAT
TTCGGACCTGCGCGCCGATACCGGGCACGGTGGCTGCTGGCGGCGGTGTCCTCGGGCCGGCCACACGCCCGTC
GGCGACGCTTTAGCTTTGGATTTTCAGGACCCGTCTTGAAACACGGACCAAGGAGTCTAGCATGTGCGCGAGT
CATTGGGACTC?TACTAAACCTAAAGGCGCAATGAAAGTGAAGGCGTGCCGAGGGAGGACCGGTCGGGGGGCG
TCTCATGCTCATCGCGAGCTGAGGCGCACCCAGAGCGTACACGC?CTTACACCGTATTCGCTGACTTGTTCGA
?CCCCATCATCGAAGACTACCATGGTGGCTTCAAGAAGACCGACAAGCACCCACCGAAGAACTGGGGTGACGG
CAGCGTCTTCTCCAATTTGGACCCTGCTGGCGAATATGTCGTCTCAACCCGTGTCCGTTGCGGCCGCTCCATG
GAGGGATACCCCTTCAACCCATGCTTAACCGAGGAACAGTACAAAGAGATGGAACAGAAGGTTTCCTCCACTT
TGTCAAGCCTCGAAGGCGAACTCAAGGGTACCTTCTACCCATTGACCGGAATGGACAAGGACACCCAACAGAA
GTTGATCGACGATCACTTCTTGTTCAAGGAGGGTGACCGTTTCCTCCAGACCGCCAACGCCTGCCGCTTCTGG
CCATCCGGACGTGGTATCTACCACAACGACAACAAAACATTCTTGGTATGGTGCAACGAAGAGGATCACCTCC
GCATCATTTCCATGCAGATGGGTGGCGATCTTGGTGAAGTCTACCGTCGCCTGGTGACCGCTGTCAACGAAAT
CGAGAAGCGCGTCCCATTCTCCCACAATGACAGATTAGGTTTCTTGACCTTCTGCCCAACCAACTTGGGCACA
ACTGTACGTGCCTCTGTACACATCAAAGTACCTAAGCTCGCCGCCAACAAGGCCAAATTG?????????????
???????????????????????????????????GTCCCTAAAAAAGTGCTCATAATTGGTTCTGGTGGCCT
CTCAATTGGACAAGCAGGAGAATTCGATTATTCCGGTTCACAAGCTATTAAAGCACTTCAAGAAGAAAATATT

CAAACAGTTCTAATAAATCCAAATATTGCAACCATACAAACATCTAAAGGTTTAGCCGATAAAATTTATTTTC
TGCCTTTAGTTCCCGAATATGTAGAACAAGTAATTAGGGCAGAAAGACCTGGTGGGGTTTTGCTAACCTTTGG
TGGGCAAACTGGTTTGAATTGTGGTGTAGAACTTCAAAAAGCGGGAGTATTTAAAAAATATGGAGTTAAAATT
TTGGGCACTCCCATACAAGCAATTATAGATACTGAAGATAGAAAGGTGTTCAGTGAAAAAATTGCAGCAATAG
GGGAAAAAGTGGCACCGAGTATGGCAGCCTATTCAGTACAAGAGGCTCTTGATGCAGCCGAACAATTAGGATA
TCCTGTAATGGCAAGAGCTGCGTTCTCATTAGGTGGTCTTGGTTCTGGTTTTGCAAATACTGCTGATGAATTA
AAATCTTTAGCAATGCAAGCCTTGGCACATTCAAATCAGTTGATTATAGATAAATCATTAAAAGGCTGGAAAG
AAGTTGAATATGAAGTAGTAAGAGATGCGTATCCGTACATTAAAGAAGTCAATGATGAAGATTTGAAAGAACC
TACTGATAAAAGAATGTTTGTTGTTGCTGCAGCATTAAGAGCTGGATACAGTGTAGAGAAATTATATAATTT?
AACCAAAATCGATTCTTGGTTCCTACAAAAAATGAAGAATATTATTGATTTCAATACTTTTTTGGAATCGATG
GATCAACATAAATTA???????????????TCATCAGATCTTTTACTTAAAGCAAAACGAATTGGTTTCAGTG
ATAAACAAATTGCTGTTGCAGTTAAAAGTACAGAATTAGCAATTAGAAAACAAAGGCTTGATTATGGTATTAC
TCCATTTGTAAAACAAATAGATACAGTTGCTGCTGAATGGCCAGCAACYACTAATTATCTTTACCTTACTTAC
AACGCTATAGAACATGACCTTGATTTCAACCAAGAACATACTATGGTTATAGGTTCTGGAGTTTATCGAATTG
GTAGTTCAGTTGAGTTTGATTGGTGTGCTGTAGGCTGCTTAAGAGAACTTAGAAAATTAAATAAGAAAACTAT
TATGGTAAATTATAATCCTGAAACTGTTAGCACTGACTATGATATGTCAGATAGATTGTATTTTGAAGAAATA
TCGTTTGAAGTAGTTATGGATGAAGTATACATTTTAATTCTCCCTGGCTTTGGAATTATTTCTCATATTATTT
GCTACAGAAGAGGAAAATCAGAAACTTTTGGGGCTTTAGGAATAATTTATGCAATATTAGCAATTGGGTTGCT
AGGATTTATTGTTTGAGCTCATCATATATTTACTGTAGGAATAGATGTTGATACTCGAGCTTATTTTACATCA
GCAACAATAGTTATCGCTGTTCCAACAGGAATTAAGGTATTTAGATGAATAGCAACTATTTATGGGGGAAATT
TAAACTTTAATCCCCCAATACTATGATCCTTAGGTTTTGTTTTTTTATTTACGGTCGGTGGATTAACAGGAGT
AATTTTAGCAAATTCATCAATTGATATTATTTTACATGATACTTATTATGTAGTAGCTCATTTTCATTACGTT
TTATCGATAGGGGCAGTTTTTGCTATTATAGCAGGATTAGTACAATGATTCCCATTATTTATAGGATTAACCT
TAAATGAAAAATGGCTGAAAATTCAATTCTTAATGATATTTATCGGGGTAAACTTAACATTTTTCCCTCAGCA
CTTTTTAGGATTAGCAGGTATACCCCGCCGATATTCTGACTACCCTGATGCCTATACAACTTGGAACGTAATT
TCTTCAATTGGGTCTATAATTTCTTTTATCGGAATTATATTTTTTTTATGAATTATTTGAGAAAGAATAATTT
CTTTACGAAGACCTTTAGGATCAATTACCCCCCCAACAGCTATTGAATGAATACATAAATATCCCCCAACAGA
GCATACTTATTCGGAACTTCCATTTGTAACTAAAGAGTATGTAGTAGTATTTGATTTCCTTGGT?AAGGATTC
CATTAGATATTATAACGAAGTACCTGTCGAAAAGAGAGTCTTTAAAAATCTGCAATTATTCATGGAGAACAAG
GCRCCCGGYGATGACTTGTTCGACAGACTGAACACATCTGTGATGAATAAACATTTAAACGAGCTAATGGAAG
GGCTCACCGCGAAGGTATTTCGTACTTACAACGCCTCATTTACAYTGCAACAACAATTAGACAAACTGACCAA
CGAGGACGATTCATTATCCGAAAAGATTCTCTCATACAACCGCGCCAACCGTGCTGTCGCCATCCTGTGTAAC
CATCAACGTGCTGTCCCGAAAGGTCACCAGAAATCCATGGAGAAGTTGAAGGAGAAGATCCAAGCCAAAAGAG
ACTCKATCAAGGAYGGGGAGCGGCAAGTCAAAGATGCCCARAARGACGCCAAGCA???CGGCAGTGTAAAAGA
AAAACAAATCTACGATAAGAAAAAGAAAATGTTGGAGAGRCTAAAGGAGCARCTGGCGAAATTAGAAATCCAG
GAGACRGACAGAGAYGAGAACAAAACGATCGCCCTCGGCACTTCGAAGCTGAACTACTTGGATCCCCGAATAT
CCGTGGCCTGGTGTAAAAAGTACAACGTGCCCATTGAAAAGATCTACAACAAAACCCAA??????CGACTTCC
ACCTTTTAGAGTAATCGGCGAYAACTTAAAAGACCGCTTTGATGGCGCATCCCGAGTTATGCTAAGTAACTCA
GCTAGTAATTC???????????????AAGAAA???TAACCGGC???CYAAGCAAGATAAATTAAGCAATAGCA
TATCCTCAAATAGCATCCATAGTAAAAGAGAAAATCGCCCTAGAAAATATAAATATGGTTTCCAACTCAAACC
GTATGATCCTGACCATAAACCACCAAGCCCTAAAGATTTAGTGTATTTAGAACCATCGCCAGGATTTTGCGAG
AAGAACCCTAAATTGGGTATCCAAGGCACTCACGGTAGGCAA?TGCAACGATACTTCAATCGGAGTGGACGGT
TGCGATCTTATGTGTTGTGGTAG?AGGCTACCGCACCCAAGAAGTCATAGTCGTAGAAAGATGTAACTGCACA
?????????????

Oedichirus

GGGATAAGCCCAGCACTGAATCCCGCGGCCGAGCCGGGAAATGTAGTGTTAGGGAGGGTCCGCTATCCATCGT
GCGGCGAGTTCAAGTCCTCCTTGAACGGGGCCACTCACCCACAGAGGGTGCCAGGCCCGATAGCGGGAGGATC
TCTCCTCAGAGTCGGGTTGCTTGAGAGTGCAGCCCTAAGTGGGTGGTAAACTCCATCTAAGGCTAAATATAAC
CACGAGACCGATAGCGAACAAGTACCGTGAGGGAAAGTTGAAAAGAACTTTGAAGAGAGAGTTCAATAGTACG
TGAAACCGTTCAGGGGTAAACCTGAGAAGCCCGAAAGTTCGAACGGGGAGATTCAGCGCGTCTCGGTTTTGGT
CGGGTGACGGTGGCGTTCGCGTCGGGCCGCACCGATCGAAGCCGTTGCCGGCGACGAAGCGTGCCACTTCTAC
CCTTGTAGGACGTCGCGACCCGTTGGGTGTCGGTCTAGGTT?CGCGGTGGAGCCCGTCGGTCCTGGCCGACTC
GCTCGACGGTAAAACGATGGCGGTGGGTCGCAAAGTTTGCGTCCGGCCCGTTGTCGGCGCGGTTGCCCCGGCT
GTCGGACTGTTGTGCCGACACCGGGAGCCGCCGTTGCCGTCAACGGTGTCCTCGGACGGGCCACACGCTTGTC
TGCGACGCTTTAGGTTTGGGCTTTCAGGACCCGTCTTGAAACACGGACCAAGGAGTCTAGCATGTGCGCGAGT
CATTGGGATTTATTCTAAACCTAAAGGCGAAATGAAAGTGAGGATAATCCTAGGGAGGATGGCCGTCGGGGCG
TCTCGTTCTCATTACGAGATGAGGCGCACCCAGAGCGTACACGC?CCTACACCGTCTTCGCCGACTTGTTCGA

?TCCCATCATCGACGACTACCATCAGGGTTTCAAAAAGACCGACAAGCATCCACCAAAGAACTGGGGCGATGT
CAGCACTTTTGGCAATCTTGACCCTACCGGCGATTACATCGTGTCGACCCGTGTACGTTGCGGCCGTTCCATG
GAAGGCTACCCCTTCAACCCTTGCTTAACCGAGGATCAATACAAAGAGATGGAACAGAAAGTGTCAACTACTC
TTTCTGCTCTCGAAGCCGAGCTTAAGGGTACCTTCTACCCGTTGACTGGCATGAGCAAGGAGACACAGCAGCA
GCTCATCGACGATCATTTCTTGTTCAAGGAAGGAGATCGTTTCTTGCAGGCCGCCAACGCTTGCCGTTACTGG
CCATCTGGACGTGGTATCTACCACAACGACAACAAGACCTTCTTGGTATGGTGCAACGAAGAGGATCACCTCC
GTCTCATTTCTATGCAAATGGGTGGCGATCTTGGCGAGGTCTACCGCCGTCTCGTAACAGCTGTCAACGACAT
CGAAAAACGCATTCCTTTCTCTCACAATGACAGATTAGGTTTCCTCACTTTCTGCCCATCCAACTTGGGCACA
ACTGTACGTTCCTCTGTACACATCAAAGTACCCAAACTTGCCGCCAACAAGGCCAAGCTT?????????????
???????????????????????????????????ATTCCTCAAAAGGTTCTCATTATAGGTTCCGGTGGATT
ATCCATAGGTCAAGCTGGAGAGTTTGATTATTCTGGTTCGCAAGCCATCAAAGCGCTTCAGGAAGAGAATATC
CAGACGGTTTTGATAAATCCTAACATAGCAACAGTGCAGACTTCAAAAGGTCTAGCGGATAAGGTGTATTTCC
TACCTTTGGTGCCGGAATATGTGGAGCAGGTGATTCAGGCTGAAAGACCAGGAGGTGTTTTGTTGACTTTTGG
TGGTCAAACTGGGTTAAATTGCGGCGTGCAGTTGCAGAAAGCTGGAGTGTTTGAAAAATACGGTGTAAAAATT
TTGGGTACGCCCATTGAAGCCATTATAGATACTGAAGATAGGAAAATCTTCAGCGAAAGGATTGCTTTGATCG
GTGAAAAGGTTGCACCTAGTATGGCCGCTTATTCGGTACAGGAAGCGCTTGATGCAGCTGAGCAATTGGGTTA
TCCTGTGATGGCTAGAGCCGCTTTTTCGTTGGGTGGTTTGGGATCTGGTTTTGCAAATAGTTGTGAAGAGCTC
AAGTCTTTGGCGCAACAAGCTTTGGCTCATTCCAATCAGTTGATTATAGATAAGAGTTTGAAGGGCTGGAAGG
AAGTGGAATATGAGGTTGTTAGAGATGCGTATCCGTACCTACAGGAAGTCAACGATGAGGAGTTGAAGGAACC
AACGGATAAACGGATGTTTGTGGTGGCTGCTGCGTTACGTTCTGGATATTCTGTTGACAGGCTTTATGAGCT?
TACAAAAATAGATCGCTGGTTTTTGCAGAAGATGAAGAACATTATAGATTTTAACACGATTTTGGAATCGATT
CATCAACACAAGATG???????????????ACGGCTAAAATTTTGCTCAAAGCCAAACAGATTGGTTTTAGTG
ATAAGCAAATTGCTGCTGCTGTGAAGAGTACGGAGTTGGCTATTAGGAAGCAACGTCAGGACTTTAAGATAAC
GCCGTTTGTGAAGCAGATAGATACAGTGGCTGCTGAATGGCCTGCAACCACAAATTACCTGTATTTGACTTAC
AACGCCATCAACCACGATTTGGCCTCTTCCGAAGAACACACCATGGTGATTGGTTCTGGTGTGTACCGAATTG
GTTCTTCCGTTGAGTTTGATTGGTGCGCTGTTGGTTGCTTGAGGGAGTTGCGTAAACTAAACAAAAAAACCAT
TATGGTAAACTACAATCCGGAAACTGTCAGTACWGACTATGACATGTCGGATCGTTTGTATTTCGAAGAAATA
TCTTTTGAAGTAGTCATGGATGAAGTTTATATTTTAATTTTACCTGGATTTGGAATAATTTCTCATATTATTA
GTCAAAGTAGAATAAAAAAAGAAACTTTTGGAACGTTAGGAATAATTTATGCAATAATAGCTATTGGGTTACT
AGGATTTGTAGTCTGAGCCCATCATATATTCACAATTGGAATAGATGTAGATACTCGTGCATATTTTACTTCT
GCTACAATAATTATTGCTGTTCCAACAGGAATTAAAATTTTTAGGTGACTTGCTACTCTTCATGGAACTCAAA
TAAATTTTAACCCTCCTTTATTATGGGCTTTGGGATTTGTTTTTCTTTTCACAATTGGTGGACTAACAGGTGT
AATTTTAGCAAACTCTTCTATCGATATTATTCTTCATGATACTTATTATGTTGTTGCTCATTTTCATTATGTT
TTATCTATAGGAGCAGTTTTTGCTATTATAGCAGGTCTTGTTCAATGATTCCCATTATTTACTGGATTATCAT
TAAATAATTTTTACTTAAAAATTCAATTTTTTACTATATTTGTAGGAGTTAATTTAACTTTTTTCCCTCAACA
TTTTTTAGGTTTAGCAGGAATACCTCGACGTTATTCTGATTATCCTGATGCTTACACTCCTTGAAATATTCTA
TCTTCTATTGGAAGATTAATTTCCTTTATTAGAATTATTTTTCTACTATTTGTAATCTGAGAAAGATTCACTT
CACGGCGTTTAATTATTTCAACTAAAAATTTTTCAACATCAATTGAATGATACCAATCATACCCACCAGCAGA
GCATAGGTATCTAGAACTTCCTATACTTTCTAGGGACTATGTGGTTGTGTTTGATTTCCTTGGT?AAAGATTC
CATTAGATACTACAATGAAGTACCTGTTGAAAAACGTGTATTTAAGAACTTACAACTATTTATGGAAAATAAA
GCACCTGGTGATGATTTATTTGATCGTTTAAATACAGCTGTGCTAAACAAACATTTAAATGAACTTATGGAAG
GCCTTACAGCCAAGGTGTTTCGTACTTACAATGCATCATTTACTTTACAGCAGCAGTTAGATAAATTGACCAA
TGCTGATGATTCCATATCTGAAAAAATCTTATCATATAATCGAGCTAACCGAGCTGTAGCTATTCTGTGTAAC
CATCAACGTGCAGTGCCTAAAGGCCATCAAAAATCTATGGAAAAATTGAAAGAGAAGATTGAAGCTAAACGAG
AAACTATAAAAGATGGGGAGAGGCAAGTAAAGGATGCACAGAAAGATGCCAGACA???TGGTAGTGTGAAAGA
AAAACAAATATACGATAAGAAGAAAAAGATGTTGGAACGCCTTAAGGAGCAGTTAGCAAAACTCGAAATTCAG
GAGACTGACCGAGACGAAAACAAAACGATTGCACTTGGTACGTCAAAACTGAATTATTTGGATCCTAGAATTT
CCGTCGCTTGGTGTAAAAAGTACGGTGTGCCCATTGAAAAAATTTATAACAAAACTCAA??????CGTTTGCC
ACCGTTCCGTGTCATCGGCGACAACCTGAAGGACCGCTTCGATGGCGCCTCTCGCGTTATGCTGAGCAACTCC
GCCAACTCCAGGAA????????????CAGTAA???AAGTAGACAAAATAAGCAGGACAAACTTTCAAATAACA
TCGCCTCCAACAGCATCCACAGTAAACGGGAGAACAGACCGCGTAAATATAAATACGGCTTTCAACTCAAACC
TTACAATCCCGACCACAAACCACCTAGTCCCAAAGATTTGGTGTATCTGGAGACATCTCCGGGATTCTGTGAG
AAAAACCCGAAACTTGGTATACAAGGTACACACGGAAGGCAA?TGTAACGACACATCGATCGGCGTGGATGGG
TGCGATCTTATGTGTTGCGGTCG?GGGATACCGTACCCAAGAGGTCGTTGTTGTGGAGAGATGTAATTGCACT
?????????????

Orus

???

???
???
???
???
?????????????????????CTGGACTGCGCCTTCTGAATCCGATGCCGGCGGCGAACGCGTGCACTTCTCC
CCTAGTAGGACGTCGCGACCCGTTGGGCGCCGGTCTACGGCCGACGGTGGAGCCTTGGGGTCCCGGCCGGCCC
GCTCGACGGTAAGACAAAGACGTGGGGTCGCGATGTTCGCGTCCGGCCCGTCACAAGTGCGAGCGACTCGGAC
GTCGGACCTGTGTGCCGACCTCGAGCTCGCCGGCTGTTGGTGACGGTGTCCTCGGACAGACTACACGTCGGTC
GGCGACGCTTTAGCTTTGGGTTTTCAGGACCCGTCTTGAAACACGGACCAAGGAGTCTAGCATGTGCGCGAGT
CGTTGGGACCGCATCTAAACCTAAAGGCGAAATGAAAGTGAAGGCGTGCCGAGGGAGGATGGGTCGGGGGGCG
TCTCGTTCTCATCGCGAGATAAGGCGCACCCAGAGCGTACACGC?????????????????????????????
???
???
???
???
???
???
???
???
???
???
???
???
???
???
???
???
???
???
???
???
???
???
???
???
???
???
???
???
???
???
???
???
??????????????????????GATATTAATTTACATGATACATATTATGT?GTAGCTCATTTTCATTATGTA
TTATCAATAGGAGCTGTATT?GCTATTATAGCGGGATTAGTTCAATGATTTCCTCTATTTACAGGACTTTCAA
TAAATGAATATTTATTAAAAATTCAATTTTTATCAATATTTATTGGAGTAAATTTA?CCTTTTTTCCTCAACA
TTTCTT?GGATTAGCTGGAATACCCCGCCGATATTCAGATTACCCTGATATTTATACTCCATGAAATGTAATT
TCCTCAATTGGCTCTCTTATTTCAATAATAAGAATTTTTTTATTTTTATTTATTTTATGAGAAAGATTTATTT
CAATACGAATAATTATTTCAGCCAAAAATTTTAGTTCTTCAATTGAATGATATCAACTATACCCCCCAGCTGA
GCA??
???
????????????GATTTATTCGACCGTTTAAACACCGGCGTCATGAACAAACACTTAAACGAACTGATGGAAG
GATTAACCGCAAAGGTGTTCCGTACTTACAACGCTTCGTGGACCTTACAGCAACAATTAGAGAAATTAACCAA
TCCGGATGATTCCATATCCGAAAAAATACTCTCCTATAACCGTGCGAACCGCGCCGTCGCCATTCTCTGTAAC
CATCAGCGTGCCGTACCGAAAGGCCACCAGAAATCCATGGAGAAGCTCAAGGAGAAAATCGAGGCTAAAAAGG
AGTCGATCCGCGACGGCGAGCGACAAGTGAAGGACGCGCAGAGGGACGCCAAGCA???CGGCAGCGTCAAGGA
GAAACAGATCTACGACAAGAAACGGAAGCAGCTGGCCAAGCTAAAGGAACAACTGGCCAAACTGGAGATACAG

GAGACGGACCGCGACGAGAACAAAACGATCGCGCTGGGTACGTCGAAGCTGAACTACCTGGACCCGAGGATCT
CGGTCGCCTGGTGCAAGAAGTTCGATGTGCCCATCGAAAAGATTTACAATAAAACTCAA??????CGTCTGCC
GCCGTTCCGCGTGATCGGCGACCACCTGAAGGACCGTTTCGACGGGGCCTCCCGCGTCATGATCAGCAATTCG
GCGAGCTCGCG???????????????GAACGC???GASCCGCC???CGAAACAGGACAAACTATCAAACAGCA
TCGCCTCCAACAGCATCCACAGCAAGCGGGAGAACAGACCGAGGAAGTACAAGTACGGCTTCCAGCTGAAACC
GTACAATCCGGAGCACAAGCCGCCGAGTCCGAAGGACCTGGTCTACTTGGAGCCGAGTCCCGGTTTCTGCGAG
AAGAACCCGAAATTGGGGATCCAGGGGACGCACGGGAGGCAG?TGCAATGATACTTCGATA????????????
???
?????????????

Oxyporus

GGGAAGAGCCCAGCACTGAATCCCGCGGCCGGGCCGGGAAATGTAGTGTTTGGGAGGATCCGCTATCCGTCGT
GCGGTGCGTCCAAGTTCTTCTTGAACGGGGCCATTTACCCATAGAGGGTGC?AGGCCCGGCGACGGGAGGATC
TCTCCTCAGAGTCGGATTGCTTGAGAGTGCAGTCCTAAGTGGGTGGTAAACTCCATCTAAGGCTAAATATAAC
CACGAGACCGATAGCGAACAAGTACCGTGAGGGAAAGTTGAAAAGAACTTTGAAGAGAGAGTTCAATAGTACG
TGAAACCGTTCAGGGGTAAACCTGAGAAACCCGAAAGTTCGACCGGGGAGATTCAGCGTGTCTCGCCAGTACT
CGTGCGACGAGGGCGTACGCGCCGTG?AGCGTCTTGTGCTCCGCAGTCCCGCCGCGTACGCGTGCACTTCTCC
CCCAGTAGGACGTCGCGACCCGTTGGGCGTCGGTCTGAGGCCCGCGGTGGAGCCCGCGCGGCCCGACCGACTC
GCTCGACGGTATGACTGTGGCGAGGGGCCGCGACGTTCGCGTCCGACCCGTCGCAAGCGCGCGCGGTCGATGT
GTCGGACCTGTGTGCCGGCCTCGAGCCCGTCGGCTGCTGGCGGCGGTGTTCTCGGACAGACTACACGCCCGTC
TGCGACGCTATTGCTTTGGGTTTTCAGGGCCCGTCTTGAAACACGGACCAAGGAGTCTAGCATGTGCGCGAGT
CACTGGGACTT?GTATAAACCCAAAGGCGAAATGAAAGTGAAGGCCCGCCTAGGGAAGATCGGTCGTGGGGCG
TCTCGCGCTCATCGCGAGCTGAGGCGCACCCAGAGCGTACATGCGCTTACACCGTCTTCGCTGATTTGTTCGA
?CCCAATCATTGAAGATTACCATACTGGATTCAAGAAGACCGACAGCCATCCACCCAAGAACTGGGGTGATGT
AAACAGCTTCGCTAACTTGGACCCCACTGGTGAATTCATTGTATCCACCCGTGTGCGTTGCGGACGTTCTTTG
GAAGGTTACCCATTCAACCCATGCTTAACCGAAGAGCAATACAAGGAGATGGAACAGAAAGTTTCGTCCACCT
TGTCTGCTTTGGAAGGTGAACTCAAGGGAACTTTCTACCCCTTGACTGGAATGGAAAAGGATGTCCAACAGAA
ACTCATCGATGACCACTTCTTGTTCAAGGAGGGTGACCGTTTCTTGCAAACTGCTAACGCTTGCCGTTTCTGG
CCATCTGGACGTGGTATCTACCACAACGACAACAAGACCTTCTTGGTCTGGTGCAACGAAGAAGACCATCTTC
GCATCATCTCCATGCAAATGGGTGGTGATCTTGGAGAAGTTTTCCGTCGTCTTGTAACTGGTGTGAATGACAT
CGAGAAGCGTATCCCATTCTCCCACAACGACCGATTGGGTTTCTTGACCTTCTGCCCAACCAACTTGGGAACA
ACTGTCCGCGCCTCCGTGCACATTAAGGTACCCAAACTCGCTGCCAACAAGGCTAAGCTCGATGAGGTTGCTG
CCAAATACAACTTGCAAGTTCGTGGAACTCGT???AATCCCAAAAAGGTTTTGATTATCGGTTCAGGTGGATT
AAGTATTGGACAAGCAGGAGAATTTGATTATTCTGGCTCGCAAGCTATCAAAGCTTTACAAGAAGAAAATATA
CAAACGGTTCTAATCAATCCCAA?ATTGCAACTGTACAAACCTCTAAAGGTTTAGCCGATAAAATTTATTTCC
TCCCTTTGGTACCTGAATATGTTGAGCAAGTAATTCGAGTAGAACGTCCAGATGGAGTTCTTCTAACCTTCGG
AGGACAAACCGGATTAAATTGTGGTGTCGCATTGGAAAAAGCTCAAATCTTCAAAAAATATGGAGTTAGGATT
TTGGGCACACCAATACAAGCGATTATCGATACAGAAGATAGGAAAATTTTTTCCGAGCGTATTCG?AGTATTG
GAGAAAAAGTTGCTCCAAGCATGGCTATACATTCAGTGCAGGAAGCTTTGGATGCTGCAGAAACTTTAGGTTT
TCCTGTAATGGCTCGAGCTGCGTTTTCTTTAGGAGGATTAGGATCTGGGTTCGCTGATGATCCTGAAGAATT?
CGCCCGTTAGCCCAACAAGCCCTAGCACACTCAAATCAGTTAATAATTGATAAGAGTTTGAAAGGTTGGAAGG
AAGTTGA?TATGAAGTTGTTAGAGATGCTTATCCGTACCTTAAAGAAGTTGATGACGAGGAGCTTAAAGCACC
CACCGATAAAAGAGTGTTTGTTGTAGCAGCTGCGCTCAAACAAGGGTACACTGTTGATAAGCTTTACCAACT?
AACCAAAATCGATCGTTGGTTCCTACAGAAAATGAAGAACATCATCACTTACGTTAATCTACTGGAATCTTTG
GACCAATTCAAACTA????????????????ACTCGTGGGGTTTATTAGAAGCTAAACAAATCGGTTTTAGTG
ACAAACAAATTGCTTCTTCTGTTAAAAGCACTGAGCTTGCAGTTCGTAAACAGCGTCAAGATTTACAAGTTAC
TCCATATGTTAAACAAATCGATACAGTTGCAGCTGAATGGCCTGCAACAACAAATTA?CTGTATTTAACATAC
AACGCAAACAGTCATGATATAACTTTCGAAGAAGAGCACACGATGGTGATAGGGTCAGGAGTTTATAGAATTG
GAAGCTCTGTAGAATTTGATTGGTGTGCTGTTGGATGTTTGCGTGAGTTACGCAAACTTAACCGAAAAACCAT
TATGGTCAACTACA???
?????????????????????GAAGTATACATTTTAATTCTTCCTGGATTTGGTTTAATTTCTCATATTATTA
GACAAGAAAGAGGAAAAAAGGAAACTTTTGGAGCATTAGGAATAATTTATGCAATATCAGCAATTGGTCTATT
AGGATTTATTGTATGAGCTCACCATATATTTACTGTTGGAATAGACGTAGACACACGAGCTTACTTTACTTCA
GCTACTATAATTATTGCTATTCCTACTGGAATTAAAATTTTTAGATGACTAGCTACTCTACACGGAACTCAAT
TAAACTATTCCCCAGCTATACTTTGGGCATTAGGATTTGTATTCTTATTCACTGTTGGAGGATTAACAGGAGT
TGTATTAGCAAACTCTTCAATTGATGTAATTTTACATGATACTTATTATGTAGTAGCTCATTTTCATTATGTT
TTATCTATAGGAGCTGTATTTGCTATTATAGGAAGATTAATTCAATGATGACCATTACTAACAGGAGTTATCT
TAGATGAAAAATTATTAAAAATTCAATTTTTAATGATATTCATTGGAGTAAATTTAACTTTCTTCCCTCAACA

TTTCTTAGGTTTATCAGGAATACCTCGACGATACTCAGATTACCCTGATGCTTATAGTTTATGAAATATAGTA
TCTTCTATTGGTTCATTAATTTCTCTAGTTAGAGTATTCGTATTCTTATACTTAATTTGAGAAAGATTTGCAT
CAAGTCGAAAAAGAATAACACCTCTTAATTTACCAACTTCTATCGAATGACTTCAATCTATACCTCCTGCTGA
ACATAGTTAT?????????????????????AAGGAATATGTGGTTGTGTTTGACTTCCTCGGT?AAAGATTC
AATTAGGTATTACAATGAAGTTCCGGTAGAGAAGCGCGTTTTTAAGAACGTGCAACTTTTCATGGAGAATAAG
GCGCCTGGTGATGATTTGTTTGATCGACTCAACACTGGAGTGATGAATAAGCATTTGAACGAGTTGATGGAAG
GACTGACGGCGAAGGTGTTCCGTACGTACAACGCTTCTATCACACTGCAGCAACAGTTGGACAAACTTACAAA
TTCGGACGATTCGGTTTCGGAAAAGATTCTTTCATACAACCGTGCTAATCGAGCTGTCGCCATTCTGTGTAAC
CATCAACGTGCTGTGCCGAAGGGGCATGACAAATCGATGGAGAAGCTAAAAGAGAAGATTGAAGC?AAACGAG
ATACTATCAAAGACGCGGAGCGTGGTGTGAAAGATGCGCAAAAAGACGCGAAACA???CGGAAGTGTAAAGGA
AAAACAGATCTACGATAAGAAAAAGAAAGCACTCGAAAGACTCAAGGAACAACTTGCAAAGCTCGAGATTCAA
GAGACGGATAGGGACGAGAATAAGACGATAGCGCTGGGTACGTCTAAGCTGAATTATTTGGATCCTAGAATTT
CGGTGGCGTGGTGCAAGAAATATGATGTACCGATTGAGAAGATCTACAATAAGACACAG?????????TTGCC
TCC?TTCCGCGCGATCGGCGATCACCTGAAGGACCGCTTCGACGGTGCGTCGCGTGTGATGCTGAGCAACGCG
AGTAACGGTCG???????????????CAATGC???GAATCGGC???CCAAAGAGAACAAGCTTTCAAATAG?A
TCCAGTCGAATAGCATCCACAGCAAGCGCGAGAACCGACCCCGTCGCTACAAATACGGATTCCAGCT?AAACC
CTACAACCCAGACCACAAGCCACCGAGCCCAAAGGATTTAGTGTACCTCGAACCATCTCCAGGTTTTTGCGAG
AAGAATCCAAAACTGGGCATTCAAGGCACGCACGGTCGACAG?TGTAACGATACGTCGATCGGGGTGGACGGT
TGCGATCTT???
?????????????

Paederus

GGGAAGAGCCCAGCACCGAATCCCGCGGCCGAGCCGGGAAATGTGGTGTTTGGGAGGATCCGCTAACCGTCGT
GCGGCGCGTCCAAGTCCTTCTTGAACGGGGCCACTTACCCATAGAGGGTGCCAGGCCCGGCAGCGGGTGGATC
TCTCCTCAGAGTCGGGTTGCTTGAGAGTGCAGCCCTAAGCGGGTGGTAAACTCCATCTAAGGCTAAATATAAC
CACGAGACCGATAGCGAACAAGTACCGTGAGGGAAAGTTGAAAAGAACTTTGAAGAGAGAGTTCAATAGTACG
TGAAACCGTTCAGGGGTAAACCTGAGAAATCCGAAAGGTCGAATGGGGACATTCAGTGCGTTTTCGTTGTGGT
CGTGCGACGGTGACGTTCGCGTCCGGCCGCGCCTTCCGCATCCGTGACCGAAGGCGAACGCATGCACTTCTCC
CCTAGTAGGACGTCGCGACCCGTTGGGTGTCGGTCTAAGGTCCGCGACGGAGCCCTCGGGACCCGGCCGGCCC
GCTCGACGGTACGAAGGTGGCGTTGGGTCGCGAAGTTCGCGCCCGGCCCGTCGCAAGCGCTTGCGTCCCGTCT
CTCGGACCTATTTGCCGAAGCGGGGCGCGCAGGCTGATTGCGACGGTGTCCTCGGGCGGACCACACGCCCGTC
GGCGACGCTTTAGCTTTGGATTTTCAGGACCCGTCTTGAAACACGGACCAAGGAGTCTAGCATGTGCGCGAGT
CATTGGTATTT?AACTAAAACTAAAGGCGCAATGAAAGTGAAGGCGTGCCTAGGGAGGACCGGGCGGGGGGCG
TCTCGTACTCATCGCGAGTTGAGGCGCACCCAGAGCGTACACGC?????????????????????????????
???
???
???
???
???
???
???
???
???
???????????????????????????????????GTTCCCAAGAAGGTACTCATAATTGGTTCTGGAGGACT
TTCTATAGGTCAGGCAGGAGAATTTGATTACTCAGGCTCACAAGCAATTAAAGCTTTACAAGAAGAGAATATT
CAAACGGTACTTATTAATCCGAACATAGCTACAGTGCAGACATCAAAAGGTATGGCTGATAAAGTGTATTTTC
TACCATTAGTACCTGAGTACGTAGAACAAGTAATTCGATCTGAAAGACCAGCAGGTGTTCTCTTAACTTTTGG
TGGTCAAACTGGTTTAAATTGCGGTGTCGAATTGGAGAAGGCCGGCGTATTTAAAAAATACGGAGTGAAAATA
CTGGGCACTCCAATTCAAGCAATCATAGAAACTGAAGATAGAAAAATATTTAGCGAACAGATTGCTATAATTG
GTGAGAAAGTTGCGCCTAGTATGGCAGCTGAATCAGTACAAGAAGCTTTGGATGCTGCTGAGCAACTAGGATA
TCCAGTAATGGCACGTGCCGCATTTTCTCTTGGCGGTTTAGGATCAGGTTTTGCTGATTGTAAAGAAGAATTG
AAGTGTTTAGCTCAACAAGC???
???
???CTGTACGACCT?
GACGAAGATCGACCGCTGGTTCATACAAAAGATGAAGAACATCATCGATTTCACGACGAGGCTGGAGCGAAC?
???CGACAAAGAATG???????????????ACCCCCGAAATCCTGCTCAAGGCCAAACAAATCGGTTTCAGCG
ACAAACAAATTGCTTCAGCCGTTAAGAGCACCGAGTTGGCGATTCGGAAACAGCGGCAGGATTTCGGACTTAC
CCCGTTCGTCAAGCAAATCGACACGGTCGCCGCGGAATGGCCGGCAACAACAAACTACCTCTACGTAACCTAC

AATGCTTTGGAGCACGACCTTGAATTCTCCGAGGAACACGTCATGGTCATAGGTTCCGGAGTTTACAGGATTG
GAAGCTCCGTCGAGTTCGACTGGTGTGCCGTCGGCTGCCTCCGCGAACTGCGGAAGCTCGGCAGGAAAACCGT
AATGGTCAACTACAACCCCGAGACTGTCAGTACCGACTACGATATGTCTGACCGACTATACTTCGAAGAAATA
TCCTTTGAAGTTGTCATGGAC???????????????????????????????????????ACATATTATTT
CTTACAGAAGAGGAAAACAAGAAACATTTGGAGCAATAGGAATAATTTATGCAATATTAGCAATTGGTTTATT
AGGATTCATTGTTTGAGCTCACCATATATTTACTGTAGGAATAGATATCGATACACGAGCTTATTTCACTTCA
GCAACCATAGTAATTGCTGTTCCTACAGGAATTAAAGTATTTAGTTGAATAGCAACTATTTATGGAGGAAATT
TAAATTTTAGCCCTCCAATAATTTGAAGATTAGGGTTTGTATTTTTATTTACAGTGGGGGGATTAACAGGAGT
AATTTTAGCAAATTCATCAATTGATATTGTTTTACATGACACTTATTATGTAGTAGCACATTTCCATTATGTT
TTATCTATAGGGGCAGTATTTGCAATTATAGCAGGATTAGTACAATGATTCCCTATATTCATTGGCTTAATAT
TAAATGAAAAATACTTAAAAATTCAATTTTTAATTATATTTATTGGAGTAAATTTAACATTTTTCCCTCAACA
TTTCTTAGGATTATCAGGAATACCTCGTCGATACTCCGATTACCCTGATGCATACACTATATGAAATGTAATT
TCATCAATTGGGTCAATAATTTCATTTATTGGAATTATATTTTTTTTATGAATTATTTGAGAAAGATTCATTT
CTATACGGAAAATTATCGGAGCTCCAATTCCTCCTACGGCATTAGAATGGATACATGCA?????????????
???????????????????????????????AAAGAATTCGTAGTTGTATTCGACTTCCTCGGT?AAAGATTC
CATTAGATATTATAACGAAGTACCTGTAGAGAAACGAGTTTACAAAAATCTGCAACTATTTATGGAAAACAAG
AAGCCCGGTGATGACCTGTTTGATCGATTGAACACGTCTGTGATGAACAAACATCTAAACGAACTAATGGAAG
GTCTCACCGCCAAGGTATTTCGTACTTACAATGCATCTTTTACGCTGCAACAACAACTGGACAAGCTAACAAA
CCCCGATGACTCTTTATCCGAGAAAATCTTGTCGTACAACCGTGCTAATCGAGCCGTAGCTATTCTATGTAAC
CATCAACGTGCCGTTCCGAAAGGTCATCAAAAATCGATGGAAAAATTAAAAGAAAAAATCGACGCCAAAAGGG
AAGCTATTAAGGACGGCGAGAGACAGGTTAAGGACGCTCAAAGAGACGCCAAGCA???CGGCAGCGTTAAGGA
GAAGCAGATATACGACAAGAAGAAGAAGGCGCTGGAACGGCTTAAGGAGCAACTGGTCAAGTTAGAAATTCAG
GAGACGGACCGCGACGAAAACAAAACCATCGCGCTCGGCACGT??????????????????????????????
???
GCCGTTCCGTGTGATCGGTGATAACCTGAAGGACCGTTTCGACGGCGCGTCTCGGGTGATGTTGAGCAATTCG
GCGAGTTCGAG???????????????GAACAG???CAACAGGC???CGAAACAGGACAAACTCAGCAACAACA
TAGCATCGAACAGCATACACAGCAAGCGCGAGAACAGACCGCGTAAATACAAATACGGCTTCCAACTGAGGCC
CTACAATCCGGACCACAAGCCACCCAGCCCAAAGGACCTGGTCTACTTGGAGCCGTCGCCCGGTTTTTGTGAG
AAAAATCCCAAATTGGGCATACAAGGCACGCACGGCAGACAA?TGTAACGACACGTCGATAGGAGTCGACGGC
TGCGACCTAATGTGCTGCGGGAG?GGGCTACAGGACGCAAGAAGTCGTCGTCGTCGAAAGGTGCAACTGCACG
TT???????????

Pinophilus

GGGAAGAGCCCAGCACTGAATCCCGCGGCCGAGCCGGGAAATGTAGTGTTAGGGAGGGTCCGCTATCCATCGC
GCGGTGCGTCCAAGTTCTCCTTGAACGGGGCCACTCACCCACAGAGGGTGCCAGGCCCGGTAGCGGGAGGATC
TCTCCTCAGAGTCGGGTTGCTTGAGAGTGCAGCCCTAAGTGGGTGGTAAACTCCATCTAAGGCTAAATATAAC
CACGAGACCGATAGCGAACAAGTACCGTGAGGGAAAGTTGAAAAGAACTTTGAAGAGAGAGTTCAATAGTACG
TGAAACCGTTCAGGGGTAAACCTGAGAAGCCCGAAAGGTCGAACGGGGAGATTCAGCGCGTCTCATCCCGTAT
CGCACGACGGTGGCGTCCGCGTCGGACCGCGTCGTACGGGGCCGTTGCCGGCGACGAACGCGTGCACTTCTCC
CCTTGTAGGACGTCGCGACCCGTTGGGTGCCGGTCTAAGGGCCGCGGTGGAGCCCGTCGGTCCTGGCCGGCTC
GCTCGACGGTAAACCTTTGGCGTTGGGTCGCGACGTTCGCGTCCGGCCCCTCGCGAGCGCGGGTGCCCCGGCC
GTCGGACTGTTGTGCCGACACCGGGCGCGCCGTTTGCCCGCGTGGGTGTCCTCGGACAGACCACCCGCCAGTC
AGCGACGCTTTAGCTTTGGGCTTTCAGGACCCGTCTTGAAACACGGACCAAGGAGTCTAGCATGTGCGCGAGT
CATTGGGACCGCATCTAAACCTAAAGGCGAAATGAAAGTAAAGGCGTGCCCAGGGAGGATGGCCTGGGGGCG
TCTCGTTCTCATCGCGAGATGAGGCGCACCCAGAGCGTACACGC?CTTACACCGTATTCGCCGACTTGTTCGA
?CCCCATTATTGAGGACTACCATACTGGCTTCAAGAAGACCGACAAGCATCCCCCTAAGAACTGGGGTGATGT
GAATACCTTCGCCAACTTGGACGCTGCCGGAGAGTACATCGTTTCCACCCGTGTACGTTGCGGCCGCTCCATG
GAAGGATACCCCTTCAACCCTTGCTTGACCGAGGACCAATACAAGGAAATGGAACAGAAGGTGTCCAGCACCT
TGTCTGGACTTGAGGGCGAGCTTAAGGGTACCTTCTACCCATTGACCGGCATGAGCAAGGAAGTGCAGCAGAA
ACTCATCGATGACCACTTCTTGTTCAAGGAGGGCGATCGCTTCTTGCAATCCGCTAACGCCTGCCGTTTCTGG
CCCTCCGGACGTGGTATCTACCACAACGACAACAAGACCTTCTTGGTATGGTGCAACGAAGAAGATCATCTCC
GCATCATCTCCATGCAGATGGGCGGTGATCTCGGCGAGGTCTACCGTCGTCTCGTCACAGCTGTCAACGACAT
CGAGAAACGCGTTCCCTTCTCCCATAACGACAGATTAGGTTTCCTCACCTTCTGCCCATCTAACTTGGGCACA
ACTGTACGTGCCTCTGTACACATCAAAGTACCCAAATTGGCCGCCAACAAGGCCAAGCTC?????????????
??????????????????????????????????ATTCCTAATAAAGTCCTTATAATTGGTTCTGGCGGATT
GTCGATTGGTCAAGCCGGGGAATTTGATTATTCAGGTTCGCAGGCCATCAAAGCACTTCAAGAAGAAAATATT
CAAACCGTTTTGATTAACCCAAACATAGCAACAGTACAAACATCGAAAGGTTTAGCAGATAAAGTTTATTTCT
TGCCTTTGGTGCCGGAATATGTTGAACAAGTGATTAGAGCTGAAAGACCAGGCGGGGTTTATTGACGTTTGG

TGGACAAACAGGTTTGAACTGCGGAGTAGAACTCCAGAAAGCTGGTGTATTTGAAAAGTACGGTGTTAAAATT
TTGGGTACCCCGATAAAGGCCATAATTGATACAGAAGATAGAAAAATGTTTAGCGATAGAATTGCACTGATCG
GGGAAAAAGTTGCGCCTAGCATGGCGGCTTATTCTGTGCAAGAAGCTTTGGATGCTGCTGAACAACTTGGTTA
TCCTGTGATGGCAAGAGCTGCGTTTTCACTGGGAGGGTTGGGATCAGGTTTTGCAAATACCTCTGAAGAATTG
AAGAGTTTAGCACAACAGGCTCTGGCACATTCCAACCAATTAATTATTGACAAGTCTTTGAAAGGATGGAAGG
AAGTGGAGTATGAAGTAGTTCGAGATGCTTATCCATACTTGAAAGAAGTTAACGATGAGGAGTTGAAAGAACC
AACTGATAAACGAATGTGGGTGGTTGCGGCAGCTTTGAGAAATGGTTATTCTGTTGAGAAGTTGTATGATCT?
AACGAAAATTGATAAATGGTTTTTACAAAAAATGAAGAATATTGTCGATTTTAATACTTTGCTAGAATCAATC
GACCAGCACACTTTG???????????????TCAGCTAAAACTTTGTTAAAAGCAAAACAAATTGGGTTTAGTG
ACAAGCAAATAGCCGCAGCTGTAAAGAGCACGGAACTTGCAATTCGAAAACAACGTCAAGACTTTAAAATTAC
ACCGTACATTAAACAAATAGATACAGTTGCTGCTGAATGGCCCGCCACTACTAACTATCTTTATTTAACTTAC
AATGCCATTAATCACGATTTACAATTTACTGAGGAGCACACTATGGTTATTGGATCAGGTGTGTATCGAATTG
GTAGCTCAGTAGAGTTTGATTGGTGTGCAGTTGGTTGCCTAAGAGAATTGAGGAAATTAAATAGGAAAACAAT
TATGGTGAATTACAATCCCGAAACTGTTAGTACTGATTATGATATGTCAGATAGATTGTACTTTGAAGAAATA
TCATTTGAAGTCGTTATGGAT?????????????????????????????????????TTTCTCATATTATTA
GACAAAACAGAGGAAAAAAGGAAACTTTTGGAGCATTAGGAATAATTTATGCAATACTAGCAATCGGATTATT
AGGATTTGTAGTATGAGCTCATCACATATTTACAGTGGGAATAGATGTTGACACTCGAGCTTATTTCACCTCA
GCTACTATGATTATTGCAGTTCCGACAGGAATTAAAATTTTGAGTTGATTAGCAACTTTACATGGAACCCAAA
TAGTATTCAACCCATCTTTATTATGAGCATTAGGATTCGTATTCTTATTTACAATCGGAGGATTAACTGGAGT
AATTCTAGCAAATTCCTCAATTGATATTATTCTTCACGACACATATTATGTTGTAGCCCATTTCCATTATGTA
TTATCAATAGGCGCTGTATTTGCAATCATAAGCGGATTAGTACAATGATTCCCATTATTTACAGGAATAACTT
TAAATAATTATTTATTAAAAATTCAATTTTTTATAATATTTATTGGTGTAAATTTAACCTTTTTCCCTCAACA
TTTTCTTGGACTAGCTGGAATACCTCGACGATACTCAGATTATCCGGATGCCTATACCCCCTGAAATGTTTTA
TCCTCAATTGGATCACTAATTTCACTTATTAGAATTATATTTTTAYTATTTATTATTTGAGAAAGRTTTACTT
CWATACGATTATCATTATTTTCCAAAAATTTTGGTACTTCTATTGAATGATATCAATTATTTCCTCCWGCTGA
ACATAGATATTCAGAATTACCAT????????AAGGACTACGTTGTTGTGTTTGATTTTCTTGGA?AAGGATTC
CATTAGATATTACAATGAAGTACCTGTTGAAAAACGTGTCTTTAAGAATTTGCAATTGTTCATGGAAAATAAG
TCACCAGGTGATGACTTATTCGATCGTTTAAATACAGGTGTATTAAACAAACATTTGAATGAACTTATGGAAG
GCCTGACAGCAAAGGTGTTTCGTACTTACAATGCATCATTTACATTACAGCAACAATTAGATAAATTGACCAA
TACTGATGATTCCATATCTGAAAAAATTTTATCATACAATCGAGCTAATCGAGCGGTAGCTATCTTATGTAAC
CATCAACGTGCAGTGCCTAAGGGCCACCAAAAATCGATGGAGAAATTGAAGGAGAAAATTGCAGCGAAGAAGG
AATCGGTATCAGATGGAGAACGACAAGTTAAGGATGCGCAGAAAGAAGCTAGACATAGTGGTAGTGTAAGAGA
CAAGCAGGTCTATGAGAAAAAGAAGAAAATGTTAGAGCGGCTCAAAGAACAACTGGCAAAACTCGAAATTCAA
GAGACAGACCGAGATGAAAACAAGACAATAGCTCTTGGTACGTCAAAACTGAATTATTTGGATCCCAGAATTT
CCGTCGCCTGGTGTAAGAAGTATGGTGTGCCCATTGAAAAAATTTATAACAAAACTCAA??????CGTCTGCC
CCCATTCCGTGTCATTGGTGACAATCTCAAGGACCGTTTCGACGGCGCATCACGCGTCATGTTAAGCAACTCC
GCCAACTCTAGGAA????????????CAGTAA???AAATCGCC???CTAAACAAGACAAACTTTCCAACAACA
TCGCATCCAACAGTATCCACAGCAAACGCGAAAACAGACCACGCAAGTACAAATACGGCTTCCAACTCAAACC
ATACAATCCTGACCACAAACCTCCGAGTCCTAAAGACCTGGTCTACCTAGAAACCTCACCCGGCTTTTGCGAG
AAGAACCCAAAACTGGGCATACAAGGCACACATAGCAGGCAA?TGTAACGACACCTCTATCGGCGTCGACGGT
TGCGATCTCATGTGCTGTGGCAG?GGGTTACAGAACACAAGAGGTCGTCGTTGTAGAGAGGTGCAACTGCACG
?????????????

Pseudolathra

GGGAGAAGCCCAGCACTGAATCCCGTGGCCCTACCGGGAAATGTAGTGTTTGGGAGGACCCGAT?TACGCCGT
GCGGCGCGTCCAAGTCCTTCTTGAACGGGGCCACATACCCATAGAGGGTGCCAGGCCCGGCGTCTGGAGGGTC
TCTCCTCAGAGTCGGGTTGCTTGAGAGTGCAGCCCTAAGTGGGTGGTAAACTCCATCTAAGGCTAAATATGAC
CACGAGACCGATAGCGAACAAGTACCGTGAGGGAAAGTTGAAAAGAACTTTGAAGAGAGAGTTCAATAGTACG
TGAAACCGTTCAGGGGTAAACCTGAGAAACCCGAAAGGTCGAATGGGGAGATTCAGCGTGTCTCGTTT?TAGG
CGCGTGACGTTGGTGCTCGCACCGGACCGCACCGTAATGACCCGTTGCTTGTGACGAACCCGTGCACTTCTCC
CCTAGTAGGACGTCGCGACCCGTTGGGTGCCGGTCTAAGGCCGACTGTGGCGCCTTGAGGTCCCGGCCGGCTC
GCTCGACGGTAAGACAGAGGCGTGGGGTCGCGATGTTCGCGTCCGGCCCGTCACAAGCACAGGCGACTCGGAC
GTCGGACCTGTGTGCCGACCTCGAGATCGCCGACTGTTGGTGACGGTGTCCTCGGACAGACTACACGTCGGTC
GGCGACGCTTTAGCTTTGGGTTTTCAGGACCCGTCTTGAAACACGGACCAAGGAGTCTAGCATGTGCGCGAGT
CATTGGGACTCTAACTAAACCTAAAGGCGAAATGAAAGTGAAGGCGTGCCGAGGGAGGATGGGTCGGGGGGCG
TCTCGTTCTCACCGCGAGATGAGGCGCACCCAGAGCGTACACGCGCTTACACCGTATTCGCTGACTTGTTCGA
?TCCCATTATTGAAGATTACCATGGTGGATTCAAGAAGACCGATAAGCACCCTCCTAAGAACTGGGGTGATGG
CAATGTCTTCACCAATCTCGACCCTGCTGGTGAATATGTCGTCTCCACCCGTGTCCGTTGCGGTCGCTCCATG

GAGGGTTACCCCTTCAACCCTTGCTTAACCGAAGAGCAATACAAGGAGATGGAACAAAAAGTTTCCTCCACTT
TGTCTGGTCTCGAAGCTGAACTCAAGGGTACTTTCTACCCATTGACTGGAATGGATAAAGATACTCAACAGAA
ACTCATCGATGATCACTTCTTGTTCAAAGAGGGAGATCGTTTCCTCCAGGCCGCCAATGCATGCCGTTTCTGG
CCATCTGGACGTGGCATCTACCACAACGACAACAAAACATTCTTGGTCTGGTGCAACGAAGAGGATCATCTTC
GTATCATCTCTATGCAAATGGGTGGAGATCTTGGTGAAGTTTACCGCCGTTTGGTCACCGCTGTCAACGAAAT
CGAGAAACGCGTACCCTTCTCCCATAACGACAGATTAGGTTTCCTCACTTTCTGCCCAACCAACTTGGGCACC
ACTGTCCGTGCCTCCGTGCACATCAAGGTCCCCAAGCTCGCCGCCAACAAAGCTAAACTCGACGAAATCGCCG
CCAAGTACAACTTGCAAGTTCGTGGCACCCGT???GTGCCTAAAAAAGTATTAATAATTGGATCTGGTGGCCT
GTCAATTGGACAAGCTGGAGAATTTGATTATTCTGGATCACAAGCCATCAAAGCTTTAAAAGAAGAAAACATA
AGAACTGTGTTAATAAACCCTAATATTGCAACGGTGCAAACTTCTAAAGGTTTAGCTGATAAAGTATATTTTC
TACCATTAATTCCTGAATATGTTGAACAAGTAATTCAATCAGAAAGACCGACGGGTGTTTTGTTAACATTTGG
AGGTCAAACAGGATTAAATTGTGGTGTAGCTTTGCAAAAAGCAGGTGTATTTGATAAATATGGTGTTAAAATA
TTAGGTACCCCTATAAAAGCCATAATTGATACAGAAGACAGAAAAATATTTAGTGAGAGGATTGCGTCTATAG
GAGAGAAAGTGGCTCCTAGTATGGCTGCTTATTCAGTCCAAGAAGCCTTAGAAGCGGCAGAATTACTTGGATA
TCCTGTCATGGCAAGAGCTGCATTTTCATTGGGAGGTTTAGGGTCAGGATTTGCTGACAACTCTGAAGAATTG
AAATCCCTTGCACAACAAGCTTTAGCTCATTCGAATCAGTTAATAATTGATAAGTCATTAAAGGGCTGGAA?G
AAGTTGAATATGAAGTAGTAAGAGATGCTTAT???
???
???
???
???
???
???
???
???
?????????????????????????????????????ATCTTCCAGGATTTGGAATAATTTCTCATATTATTA
GACAAGGTAGAGGAAAAAAGGAAACCTTCGGAGCACTAGGTATAATTTATGCTATAATAGCTATTGGATTATT
AGGATTTGTAGTATGAGCTCACCATATATTTACTGTTGGAATAGATGTTGATACTCGGGCTTATTTTACTTCA
GCCACTATAATCATTGCTGTTCCTACAGGAATTAAGATTTTTAGATGATTAGCTACTCTTCATGGATCCCAAC
TAACATTTAACCCACCTATTTTATGATCTTTAGGATTTGTTTTTTTATTTACAATTGGAGGATTAACTGGAGT
AATTTTAGCTAACTCATCAATTGATATTATTTTACACGATACTTACTATGTTGTTGCCCATTTTCATTATGTA
TTATCTATAGGAGCAGTATTTGCAATTATAGCAGGATTAATACAATGATTTCCACTATTTACAGGTCTTTCTA
TAAATGAATATTTATTAAAAATTCAATTTTTAATTATATTTATTGGAGTAAACCTAACCTTTTTCCCTCAACA
TTTTCTTGGATTAGCAGGAATACCCCGACGATACTCAGACTACCCTGACGTTTATACTCCATGAAATGTAATC
TCATCAATTGGTTCTTTAATTTCTATAATTAGAATTATATTACTATTATACATTCTTTGAGAAAGATTCTCTT
CTATACGAATTATCTTATCTGCTAAAAACTTTTCTACTTCAATCGAATGATTTCAATACTATCCTCCTGCTGA
ACACAGATAT?????????????????????AAAGATTACCTTGTCGTCTTTGATTTCCTTGGT?AAAGATTC
CATTAGATACTATAATGAAGTACCTGTAGAAAAACGTGTTTTCAAGAATCTCCAGTTGTTCATGGAAAATAAA
TCACCGGGTGATGATTTATTCGATCGTTTAAACACCGCTGTGATGAACAAACACTTGAACGAGTTAATGGAGG
GTCTAACCGCCAAGGTATTTCGTACTTACAATGCTTCTTGGACATTGCAACAACAACTTGATAAGTTAACAAA
TGAAGATGATTCGATATCCGAGAAAATTTTATCATACAATCGTGCCAATCGTGCTGTCGCCATTCTATGTAAC
CATCAACGTGCGGTGCCTAAAGGCCATCAGAAATCCATGGAGAAGTTAAAGGAAAAAATTGAGACTAAGAAAG
ACGCCATTAGGGACGGGGAGCGTCAAGTGAAAGACGCACAAAAGGATGCGAAACA???TGGAAGTGTGAGGGA
AAAGCAAATCTACGATAAGAAGAAGAAAATGTTGGAACGCCTCAAAGAACAATTAGCGAAACTGGAAATCCAA
GAGACGGATCGTGATGAAAACAAAACTATTGCGCTCGGTACGTCAAAGTTGAATTATTTAGATCCGAGGATTT
CCGTTGCATGGTGTAAGAAGTTCGGTGTACCCATTGAAAAGATCTATAATAAAACTCAA??????CGTTTGCC
GCCGTTCCGTGTGATCGGTGATCATTTGAAGGATCGCTTCGATGGGGCGTCCCGTGTGATGTTGAGTAATTCA
GCGAGCGCACGTGGCGG?????????TAACGC???GAACAGGC???CCAAGCAGGATAAGCTGTCGAATAATA
TCGCGTCGAATAGTATTCATAGTAAAAGGGAGAACAGGCCTCGGAAGTATAAGTACGGTTTTCAATTGAAGCC
GTACAATCCGGACCATAAGCCTCCGAGTCCTAAGGATTTGGTGTACTTGGAGCCGTCGCCTGGTTT?TGCGAG
AAGAATCCGAAGCTTGGTATCCAGGGGACGCACGGCAGGCAG?TGTAATGACACGTCGCTGGGGGTGGACGGG
TGCGACCTG??
?????????????

Pseudomedon

GGGAGAAGCCCAGCACTTAATCCCGTGGCCGAACCGGGAAATGTAGTGTTTGGGAGGGTCCATCATCCATCGT
ACGACGCGTCCAAGTCCTTCTTGAACGGGGCCACATACCCATAGAGGGTGCCAGGCCCGGTAGCTGGTGGATC
TCTCCTCAGAGTCGGGTTGCTTGAGAGTGCAGCCCTAAGTGGGTGGTAAACTCCATCTAAGGCTAAATATGAC

CACGAGACCGATAGCGAACAAGTACCGTGAGGGAAAGTTGAAAAGAACTTTGAAGAGAGAGTTCAATAGTACG
TGAAACCGTTCAGGGGTAAACCTGAGAAACCCGAAAGGTCGAATGGGGAGATTCAGCGTGTCTCGTTTTCGGT
CGCGTGACGAAGGTGCTTGCACCGGGGCGCGCCTTCCGGATCCGTAACCTGCGACGAACCCGTGCACTTCTCC
CCTAGTAGGACGTCGCGACCCGTTGGGTGCCGGTCTAAGGCCGACGGTGGAGCCTTGGGGTCCCGGCCGGCCC
GCCCGACGGTAAGACAGAGGCGTGGGGTCGCTACGTTAGCGTCCGGCCCGTGACAAGTTCGAGCGACTCGGAT
GTCGGACCTGCGTGCCGACCCCGAGCTCGCCGGCTGTTGGTTACGGTGTCCTCGGACAGACTACACGCCGGTC
GGCGACGCTTTAGCTTTGGGTTTTCAGGACCCGTCTTGAAACACGGACCAAGGAGTCTAGCATGTGCGCGAGT
CATTGGGACCGCATCTAAACCTAAAGGCGAAATGAAAGTGAAGGCGTGCCGAGGGAGGATGGGTCGGGGGGCG
TCTCGTTCTCATCGCGAGATGAGGCGCACCCAGAGCGTACACGCTCTTACACCGTATTCGCTGATTTGTTCGA
?TCCCATTATTGAAGATTACCATGGTGGATTCAAGAAGACCGACAAGCACCCTCCCGCAAACTGGGGTGATGT
CAACACTTTCGCAAACCTCGACCCAGCTGGCGAGTACGTAGTCTCAACCCGCGTCCGTTGCGGCCGCTCAATG
GAAGGCTACCCATTCAACCCATGCTTAACCGAGGAACAATACAAGGAGATGGAACAGAAGGTCTCCAGCACCT
TGTCCGGTCTCGAAGGCGAACTTAAGGGTACTTTCTACCCATTGACTGGAATGGATAAGGATACTCAACAGAA
GCTCATCGACGATCACTTCTTGTTCAAGGAAGGTGACCGTTTCCTCCAGACCGCCAACGCCTGTCGTTTCTGG
CCATCTGGACGTGGTATCTACCATAACGACAACAAAACATTCTTGGTCTGGTGCAACGAAGAGGACCACCTTC
GTATCATCTCCATGCAGATGGGTGGTGATCTTGGTGAAGTCTACCGTCGTCTCGTCACTGCTGTCAACGAAAT
CGAGAAGCGCGTCCCCTTCTCCCACAATGACAGATTAGGTTTCCTTACTTTCTGCCCAACCAACTTGGGCACA
ACTGTACGTGCCTCTGTACACATTAAAGTACCTAAGCTCGCCGCCAACAAAGCTAAGCTCGATGAAGTCGCCG
GAAAATACAACTTGCAAGTACGTGGTACCCGTGGTGTTCCAAAAAAAGTTTTAATTATTGGTTCAGGTGGTTT
GTCTATTGGACAAGCCGGTGAATTTGATTACTCTGGTTCACAAGCAATTAAAGCATTGCAAGAAGAAAATATA
CAAACAGTCCTTATTAACCCTAATATTGCTACAGTTCAAACATCGAAGGGTTTAGCTGATAAAATTTACTTCT
TACCTTTAGTGCCTGAATTTGTGGAGCAGGTAATTCGTGTAGAACGTCCTGGCGGTGTYTTGTTAACATTTGG
AGGACAAACAGGGTTAAATTGTGGTGTAGAATTACAAAAAGCTGGTATTTTTGAAAAATATGGTGTTAAGATT
TTGGGTACACCTATACAAGCTATAATTGATACGGAAGACAGAAAAGTTTTTAGTGAAAGAATTGCTATGATTG
GCGAGAAGGTAGCTCCAAGTATGGCCGCTTATTCAGTACAAGAAGCACTCGAAGCTGCTGAATTATTGGGTTA
TCCAGTAATGGCAAGAGCCGCCTTTTCTTTAGGTGGTTTAGGATCTGGATTTGCAAATACTGCCGAAGAATTG
AAATCACTTGCTCAACAAGCTTTAGCTCATTCAAATCAGTTAATTATTGATAAGTCTTTAAAGGGATGGAAGG
AAGTTGAATATGAAGTTGTCAGAGATGCATATCCTTACATTAAAGAAGTTAATGACGAAGAACTACAAGAACC
GACAGATAAAAGGATGTTTGTACTTGCAGCTGCTTTAAGAAATGGTTATAGTGTTGATAAGTTGTATGATCT?
AACCAAAATTGATCGTTGGTTTTTACAAAAAATGAAAAATATCGTTGATTACAATACTGTACTAGAATCGATA
CAACAAAATAAATTG???????????????ACATATAAAGTTTATTAAAAGCCAAGCAAATTGGTTTAGTG
ATAAACAAATAGCTGTTTGTGTTAAAAGTACTGAACTTGCAATTCGAAAACAACGACAAGATTTTGATATAAC
TCCGTACGTTAAACAAATCGATACTGTAGCTGCTGAATGGCCTGCTACCACTAATTATTTGTATTTAACATAC
AATGCTGAAACTCATGACTTAACATATAACGAAAAACATATAATGGTTATAGGATCAGGAGTGTACCGTATTG
GAAGTTCTGTTGAATTTGATTGGTGTGCTGTAGGTTGTTTAAGAGAACTTAGAAAATTAAACAAAAAAACAAT
AATGGTCAATTACAATCCCGAGACTGTGAGTACAGACTATGATATGTCAGATAGATTATATTTTGAAGAAATA
TCGTTCGAAGTTGTTATGGATGAAGTTTATATTTTAATTCTTCCAGGATTTGGTATAATTTCCCATATTATTA
GCCAAGCTAGGGGAAAAAAAGAAACTTTTGGAACCCTAGGAATAATTTATGCTATAATAGCCATTGGTTTATT
GGGTTTTGTTGTATGAGCCCACCATATATTTACTGTAGGTATAGATGTAGATACCCGGGCTTATTTCACATCG
GCAACAATAATTATTGCTGTTCCAACAGGAATTAAAATTTTTAGCTGATTAGCTACACTACACGGAACACAAA
TAAAATTTAATCCCCCAATATTATGATCTCTAGGATTTGTTTTTTTATTCACTATTGGAGGATTAACCGGAGT
AATTTTAGCTAATTCATCAATTGATATTATCCTTCATGATACTTACTATGTCGTAGCTCATTTCCATTATGTT
CTTTCCATAGGAGCAGTATTTGCTATTATAGCAGGTCTAGTTCAATGATTCCCCTTATTTACAGGATTAACCT
TAAATGAATACATATTAAAAATTCAATTTTTTATTATATTTATTGGAGTAAATATAACCTTTTTTCCCCAACA
CTTCTTAGGACTAGCTGGCATGCCCCGTCGTTATTCCGACTATCCAGATGCATATACCCCATGAAATGTAATT
TCATCAATTGGTTCTTTAATCTCAATAATTAGAATTTTCCTTTTACTATTTATTTTCTGAGAAAGGTTTACTT
CTATACGAATTGTTATTTCTTCAAAAAATTATGTCACTTCAATTGAATGATTACAATTACATCCACCAGCAGA
ACATAGCTATTCTGAATTACCCATATTAACTAAAGATTATGTAGTAGTATTTGATTTCCTTGGT?AAGGATTC
AATTAGATACTACAATGAGGTACCTGTAGAAAAACGTGTCTTCAAAAATCTACAATTGTTTATGGAAAATAAA
TCACCAGGCGATGATTTGTTTGATAGATTAAACACAGCTGTGATGAACAAACATTTAAACGAGCTAATGGAAG
GATTAACTGCCAAGGTGTTTCGTACTTATAACGCTTCATGGACCTTACAACAGCAACTAGACAAATTGACCGA
CCCAAACGATTCCATATCCCAAAAAATTTTATCTTACAACAGAGCAAATCGAGCTGTCGCTATACTTTGCAAC
CATCAACGTGCAGTACCGAAAGGCCACCAAAAATCTATGGAGAAACTTAAGGAAAAAATCGAAGCCAAAAGAG
AGTCTATCAGAGATGGCGAGAGACAAGTGAAAGACGCGCAGCGTGACGCAAAGCA???TGGCAGCGTCAAAGA
AAAACAGATATACGAGAAGAAGAAGAAAATGTTGGAGAAACTCAAAGAGCAACTGACTAAACTGGAGATTCAG
GAGACGGACCGCGACGAAAACAAAACCATTGCTCTCGGTACGTCGAAACTGAATTATTTGGACCCGAGAATTT
CGGTCGCATGGTGTAAGAAGTTTGGTGTGCCCATTGAAAAAATTTATAACAAAACTCAA??????????????

???TCGCGCGTGATGCTGAGCAACTCG
GCGAGCTCGCGCGG????????????CGGCAA???CGCGAGGC???CCAAGCAGGATAAGCTCTCGAACAACA
TCGCCTCGAACAGCATACACAGCAAGAGAGAAAACCGGCCGCGCAAATACAAGTACGGGTTCCAATTGAAGCC
GTACAATCCGGACCACAAGCCTCCGAGCCCTAAGGATTTGGTGTATCTGGAACCSTCGCCCGGTTTCTGCGAG
AAGAACCCGAAGCTCGGGATACAGGGCACGCRCGGTAGGTTA?TGCAACGATACTTCGATCGGGGTGGACGGG
TGCGATCTGATGTGCTGCGGGAG?AGGATACAGGACCCAGGAGGTTATCGTTGTCGAGAGGTGCAACTGC???
?????????????

Quedius

GGGAAGAGCCCAGCACCGAATCCCGCGTCCGTGCCGGGAAATGTGGTGTTAGGGAGGGTCCGCCATCCGTCGC
GCGGTCGGTCCAAGTCCTTCTTGAACGGGGCCACTTACCCATAGAGGGTGCCAGGCCCGGCAACGGGAGGATC
TCTCCTCAGAGTCGGGTTGCTTGAGAGTGCAGCCCTAAGTGGGTGGTAAACTCCATCTAAGGCTAAATATAAC
CACGAGACCGATAGCGAACAAGTACCGTGAGGGAAAGTTGAAAAGAACTTTGAAGAGAGAGTTCAATAGTACG
TGAAACCGTTCAGGGGTAAACCTGAGAAACCCGAAATGTCGAATGCAGAGATTCAGCGTGTCTCGCTGGTGGT
TGTGTGACGGCGACGAACGCGTC?GACCGCTCCTTCCGCCGCCG??ATTCGCGGCGAACGCGTGCACTTCTCT
GCTAGTAGGAAGTCGCGACCCGTTGGGTGCCGGTCTAAGGACCGCGGTGGAGCCCGAGTGTCCCGACCGGCTC
GCTCGACGGTACGACAGTGGCGCGGGGCCGCGATCTTCGCGTCCGGCCCGTCGCAAGTACGGACGGTCTGGAT
GTCGGACCTA?GTGCCGACTTCGGAGCCGCCGGCTGCTGGCGACGGTGTCCTCGGACAGACTATACGCCGGTC
GGCGACGATTTAGCTTTGGGTTTTCAGGACCCGTCTTGAAACACGGACCAAGGAGTCTAGCATGTGCGCGAGT
CATTGGGACTT?AGCGAAACCTAAAGGCGTAATGAAAGTGAAGGCGCGCCTAGGGTGGATGCGCCGGGGGGCG
TCTCGTGCTCATTGCGAGCTGAGGCGCACCCAGAGCGTACACGCGCTTACACCGTATTCGCCGACTTATTCGA
?CCCCATCATCGAAGATTACCATACAGGCTTCAAGAAGACCGACAAGCATCCACCAAAGAACTGGGGCGATGT
CAACACCTTTGCCAATCTCGACCCTGCTGGCGAGTACGTCGTATCCACCCGCGTCCGTTGCGGTCGCTCCATG
GAGGGATACCCCTTCAACCCCTGTTTAACCGAAGACCAATACAAGGAGATGGAACAGAAGGTCTCCACCACCC
TCTCTGGACTTGAGGGTGAACTCAAGGGTACCTTCTACCCATTGACTGGCATGGGCAAGGACGTCCAACAAAA
ACTGATTGATGACCATTTCCTCTTCAAGGAAGGAGATCGCTTCCTCCAGGCCGCCAACGCTTGCCGCTACTGG
CCCAGCGGACGTGGCATCTACCACAACGACAACAAGACCTTCTTGGTCTGGTGCAACGAAGAGGACCATCTCC
GTCTTATCTCCATGCAAATGGGTGGCGATCTTGGTGAAGTTTACCGTCGTCTCGTAAATGCCGTCAACGATAT
CGAAAAGCGCGTTCCTTTCTCTCACAATGACAGATTAGGTTTCCTTACTTTCTGCCCAACCAACTTAGGCACA
ACTGTTCGTGCCTCCGTACACATTAAGGTCCCCAAGCTCGCCGCCAACAAGGCTAAGCTCGACGAAATCGCCG
GCAAATACAACTTGCAAGTCCGCGGAACCCGT???GTGCCGAAAAAGGTGTTGATAATCGGTTCGGGTGGATT
GTCGATCGGTCAGGCGGGCGAGTTTGATTATTCGGGGTCTCAGGCGATTAAGGCGTTGCAGGAGGAGGGGATT
CAGACGGTTTTGATCAATCCGAATATAGCTACGGTGCAGACGTCAAAGGGTTTAGCGGATAAAGTTTACTTTC
TACCGTTAGTGCCCGAATACGTGGAGCAGGTGATTCGTGTGGAGAGACCTGGAGGGGTGCTGCTGACGTTCGG
CGGACAGACTGGATTGAATTGCGGCGTTGAGTTGGAGAGGGCTGGAGTTTTTAAGAAGTATAATGTTAAGATT
CTTGGGACACCGATACAAGCGATTATAGATACGGAGGATAGGAAGGTGTTTAGTGATAGGATCGGGCAGATTG
GGGAGAAGGTCGCGCCTAGCATGGCCGCGTATTCCGTGCAGGAGGCGCTTGAAGCTGCGGAGAAGCTGGGGTA
CCCCGTGATGGCGAGAGCGGCTTTTTCTTTGGGGGGACTTGGATCGGGCTTCGCGGATACTAAAGAGGAACTC
AAGTCGCTCGCGCAACAAGCATTGGCCCATTGGACTCAGTTGATTATCGATAAGTCGTTGAAAGGATGGAAGG
AGGTGGAGTACGAGGTGGTGAGGGATGCTTTTTCGTATCT?AAAGAGGTCGACGATGAGGATCT?AAGGAACC
GACCGACAAGCGGATGTTCGTGGTCGCGGCCGCGTTAAGATCCGGATATTCCGTGGATAAACTTTACGATTT?
AACGAAAATCGATCGTTGGTTTCTGCAAAAGATGAAGAATGTGGTGGATTATAATTCATTCCTGCAAACCATC
GATCAGATCAATCTT???????????????ACGAAAGACAACCTGTTACGCGCGAAGCAAATTGGTTTTAGTG
ATAAACAAATTGCCGTCGCTGTTAAGAGCACAGAACTGGCTATCAGGAAGCAAAGGCAGGATTTTAACCTCAC
CCCCTACGTGAAGCAAATCGACACAGTCGCCGCGGAATGGCCTGCGACAACCAACTACCTTTACCTAACGTAC
AACGCGGTAGC?AACGATTTGACTTTCGC?GAAGAACATACTATGGTGATAGGTTCTGGCGTATACCGCATCG
GAAGCTCCGTAGAATTCGACTGGTGCGCGGTGGGTTGCCTGCGCGAGTTACGAAAACTCGGTAAGAAGACAAT
AATGGTAAATTACA???
?????????????????????GAAGTCTATATTTTAATCCTACCTGGATTTGGGATAATCTCCCATATTATTA
GGCAGGAAAGAGGAAAAAAAGAAGCCTTTGGAACGTTAGGGATAATTTATGCAATAATAGCAATTGGTTTATT
AGGATTTATTGTATGAGCCCATCACATATTCACAGTAGGAATAGATGTTGATACACGAGCTTATTTTACTTCA
GCAACAATAATTATTGCTGTTCCAACAGGAATTAAAATTTTTAGGTGAATGGCTACATTACATGGAACTCAAA
TTAATTACTCCCCCTCAATGATTTGAGCTTTAGGTTTCGTATTTTTATTTACAGTTGGTGGTTTAACTGGAGT
AGTATTAGCTAATTCATCTATCGATATTATTTTACATGATACCTATTATGTTGTTGCCCATTTTCACTATGTT
TTATCAATAGGGGCAGTATTTGCTATTATAGCAGGATTAATACAATGATTTCCTTTATTCACAGGTTTAACTT
TAAATGAAAAATTTTTAAAAATTCAATTTTTTTCTATGTTCATTGGAGTAAATTTAACTTTTTTCCCTCAACA
TTTTTTAGGATTAGCTGGAATACCTCGACGATACTCCGATTACCCGGATATTTATACACCTTGAAATGTAATT
TCATCAATCGGATCTTTAATTTCAACCATAAGAATTTTTTTACTATTATTTACCATTTGAGAAAGATTTGTAT

CTACTCGAAAAAGAATATCCCCATTAAATTTACCATCCTCAATTGAATGGCTTCAATCTATACCACCTGCTGA
GCATAGATAT?????????????????????AAGGAGTACGTAGTCGTGTTTGATTTCCTCGGT?AAAGATTC
TATTCGGTATTACAATGAGGTGCCCGTCGAGAAGCGCGTTTTTAAAAATCTCCAATTGTTCATGGAGAACAAG
GCTCCGGCTGATGATCTGTTTGATCGGTTGAACACCGCCGTTATGAACAAGCACTTGAACGAGCTGATGGAAG
GTTTGACCGCAAAGGTATTCCGTACTTACAATGCCTCGTTTACTCTGCAACAGCAGCTGGATAAGTTAACGAA
CGAAGATGATTCCTTATCAGAGAAAATTTTATCTTACAACAGAGCGAATCGAGCTGTTGCCATACTGTGTAAC
CATCAACGTGCTGTTCCGAAGGGACATCAGAAGTCGATGGAGAAGCTGAAGGAGAAAATTGACAGCAAACGGG
ACGCTATCGCTGACGGTGAACGACAAGTCAAGGATGCGCAAAGAGAGGCCAAGCA???CGGAAGTGTAAAAGA
GAAGGTGAATTTCGATAAAAAGAAGAAAATGTTGCAGAGATTAAAGGAACAGTTGGCTAAATTAGAAATTCAA
GAGACGGACAGGGATGAGAATAAGACAATTGCTTTGGGCACGTCCAAGCTGAATTATTTGGATCCTAGGATAT
CTGTGGCATGGTGCAAAAAGTTTGATGTGCCGATTGATAAGATCTACAACAAAACTCAA????????GTTACC
ACCTTTCCGCGTCATTGGTGATAACCTGAAGGACCGTTTCGACGGTGCTTCACGGGTAATGCTTAGTAACACG
GCAAACTCGAGGAATAGCAACAATGCCCACAA???CAGCCGCC???CGAAACAAGATAAGCTCTCGAATAGTA
TCGCATCCAATAGTATACACAGCAAACGCGAGAACAGGCCGCGCAAATACAAGTACGGTTTCCAATTAAAACC
ATACAATCCTGACCACAAGCCGCCTAGTCCTAAAGATCTGGTCTACTTGGAGCCGTCGCCTGGTTTTTGCGAG
AGGAATCCCAAATTGGGCATACAGGGCACTCACGGCAGACAA?TGCAACGACACTTCGATTGGAGTTGACGGC
TGCGATCTT??
?????????????

Ronetus

GGGAGAAGCCCAGCACTGAATCCCGTGGCCGAACCGGGAAATGTAGTGTTTGGGAGGGTCCGTTATCCATCGT
GCGGCGCGTCCAAGTCCTTCTTGAACGGGGCCATATACCCATAGAGGGTGCCAGGCCCGATAGCTGGAGGATC
TCTCCTCAGAGTCGGGTTGCTTGAGAGTGCAGCCCTAAGTGGGTGGTAAACTCCATCTAAGGCTAAATATGAC
CACGAGACCGATAGCGAACAAGTACCGTGAGGGAAAGTTGAAAAGAACTTTGAAGAGAGAGTTCAATAGTACG
TGAAACCGTTCAGGGGTAAACCTGAGAAACCCGAAAGGTCGAATGGGGAGATTCAGCGTGTCTCGTTTCTGGT
TTCGTGACGATGGTGCTTGCACCGGGTAGCGCATTCCGGATCCGTAACCGGCGACGAACTCGTGCACTTCTCC
CCTAGTAGGACGTCGCGACCCGTTGGGTGTCGGTCTAAGGCCGACGGTGGAGCCTTGGGGTCCCGACCGGCCC
GCTCGACGGTGAGACAGAGACGTGGGGTCGCTTCGTAAGCGTCCGGCCCGTCACAAGTCCGGACGACTCGGAC
GTCGGACCTGTGCGCCGACCTCGAGCTCGCCGGCTGCTAGTGACGGTGTCCTCGGACAGACTACACGTCGGTC
GGCGACGCTTTAGCTTTGGGTTTTCAGGACCCGTCTTGAAACACGGACCAAGGAGTCTAGCATGTGCGCGAGT
CATTGGGACCGCATCTAAACCTAAAGGCAAAATGAAAGTAAAGGCGTGCCGAGGGAGGATGGGTCGGGGGGCG
TCTCGTTCTCATCGCGAGATGAGGCGCACCCAGAGCGTACACGC???????????????????????????GA
?TCGCATTATTGAAGATTACCATGGCGGATTCAAGAAGTCCGACAAGCACCCACCCGCGAACTGGGGCGACGT
GAACACCTTCACCAACCTGGACCCTGCTGGAGAGTACGTAGTGTCCACCCGCGTCCGTTGCGGCCGTTCCATG
GAAGGATACCCCTTCAACCCGTGCTTGACCGAAGAACAATACAAAGAAATGGAAGGCAAAGTGTCCAGCACCT
TGTCTGGCCTCGAAGGAGAACTCAAGGGCACTTTCTATCCATTAACCGGCATGGACAAGGCAACTCAACAGAA
GCTCATAGATGATCACTTCTTGTTCAAGGAAGGAGACCGTTTCCTCCAGGCGGCCAACGCCTGCCGCTTCTGG
CCATCCGGGCGTGGTATCTACCACAACGACAACAAAACCTTCTTGGTCTGGTGCAACGAAGAGGATCATCTCC
GTATCATCTCCATGCAGATGGGCGGCGACCTTGGTGAAGTATACCGTCGCCTCGTGACAGCCGTCAACGAAAT
CGAGAAGCGCGTCCCATTCTCCCACAATGACAGATTAGGTTTCCTCACCTTCTGCCCAACCAATTTGGGCACA
ACTGTTCGTGCCTCTGTACACATCAAGGTACCAAAGCTCGCAGCCAACAAGGCCAAGCTCGACGAAGTCGCTG
GCAAATACAACTTGCAAGTACGTGGTACTCGCGGTGTTCCAAAAAAAGTTTTAATCATCGGTTCAGGTGGATT
GTCCATAGGACAAGCTGGCGAATTCGATTATTCCGGTTCACAAGCGATAAAGGCCTTACAAGAAGAAAACATA
CAAACAGTCCTGATCAACCCAAACATAGCAACCGTGCAAACGTCTAAAGGTCTAGCTGATAAAATTTATTTTT
TACCATTGGTGCCTGAATTTGTCGAACAAGTGATCAGAGCAGAACGTCCTGGAGGGGTTCTGCTTACATTCGG
TGGACAAACAGGGTTGAATTGCGGTGTAGAACTTCAGAAAGCGGGTGTATTTGAAAAGTACGGCGTTGAAATT
TTGGGCACTCCCATACAAGCAATTATTGATACTGAAGACAGGAAGATTTTTAGTGAAAGAATTGGTTTCATAG
GTGAAAAAGTTGCACCAAGTATGGCGGCATATTCTGTACAAGAAACATTGGAAGCAGCTGAGTTATTAGGTTA
TCCTGTAATGGCAAGGGCAGCTTTTTCACTAGGCGGTTTAGGATCTGGGTTCGCTAATAATGATGAAGAACTG
AAGACTCTTGCTCAACAAGCGTTAGCT??
???
???
???
???
???
???
???
???

??
????????????????????????????????TTTAATCTTACCAGGATTTGGGATAATTTCCCATATTATTA
GACAATCTAGAGGTAAAAAAGAATCTTTTGGTTCCTTAGGAATAATTTACGCAATAATAGCTATTGGATTATT
AGGATTTGTAGTATGAGCTCATCATATATTCACAGTAGGAATAGATGTAGACACTCGAGCATACTTCACATCA
GCAACAATAATTATTGCAGTACCAACAGGAATTAAAATTTTTAGATGATTAGCTACTTTACATGGCACTAAAA
TTAAAATTTCCCCCCCTATTCTTTGAGCTATTGGATTTGTATTTTTATTTACAGTTGGTGGATTAACAGGAGT
AATTTTAGCTAATTCATCAATCGATATCATCCTCCATGATACTTATTATGTGGTTGCCCATTTCCATTATGTC
CTTTCAATAGGAGCTGTTTTTGCCATTATAGCAGGACTAGTCCAATGATATCCTCTATTTACTGGGTTATCTA
TAAATAATTTTTACTTGAAAGTTCAGTTTTTTATTATATTTATAGGCGTAAATATAACTTTTTTCCCTCAACA
TTTTTTAGGTTTAGGAGGTATACCTCGGCGATATTCAGATTATCCAGATGCTTACACTACTTGAAACATTATT
TCTTCAATTGGATCAATAATTTCAATAATAAGAATTTTTTTCTTAATTTTTATTATTTGAGAAAGTTTTGCGT
CTATACGAATTAACATTTCCCCTAAAAACCTTTCTACTTCTATCGAATGAATACAATTAACCCCACCATCTGA
ACATAGATATA????????????????????AAAGATTATGTGGTAGTATTTGACTTCCTCGGT?AAGGATTC
TATTAGATACTACAATGAAGTACCTGTGGAAAAACGTGTCTTCAAAAACCTTCAGTTGTTCATGGAAAACAAA
TCCCCAGGGGATGATTTATTTGATAGATTAAACACAGCTGTAATGAACAAACATTTAAACGAACTAATGGAAG
GTTTAACTGCCAAGGTGTTTCGTACTTACAATGCTTCTTGGACTCTTCAACAGCAACTAGATAAATTGACCAA
TCCTGATGATTCCATATCAGAAAAAAATATTATCATACAATCGAGCCAACAGAGCGGTAGCTATTCTATGTAAC
CATCAACGTGCTGTCCCTAAGGGCCACCAAAAATCCATGGAAAAACTCAAAGAAAAAATTGATGCCAAAAAAG
AAGCTATAAAAGATGGCGAGAGACAAGTTAAAGATGCACAAAAAGATGCGAAGCG???TGGTAGCGTAAAGGA
AAAACAAATTTATGATAAGAAGAAGAAAATGTTGGACAGACTCAAAGAGCAGTTGGCAAAATTGGAAATTCAG
GAGACGGACCGTGATGAAAACAAAACAATCGCCCTTGGTACTTCCAAGTTGAACTATTTGGATCCCAGAATTT
CGGTCGC??TGGATGCGCCTGCC
ACCCTTCCGAGTGATCGGCGACCATTTAAAAGATCGATTCGACGGTGCCTCCAGGGTGATGCTGAGCAATTCC
GCGAGTTCCCGCGG?????????????GAACGC???CAACAGGC???CCAAGCAGGACAAACTCTCGAACAGCA
TCGCGTCCAACAGCATCCACAGCAAAAGAGAAAACCGCCCGCGCAAGTATAAGTACGGTTTCCAATTGAAGCC
GTACAACCCGGACCACAAACCTCCGAGCCCGAAGGACCTGGTGTACCTGGAGCCGTCGCCTGGATTCTGCGAG
AAGAACCCCAAGCTCGGCATACAAGGTACGCACGGTAGATTG?TGCAACGACACCTCCATAGGTGTCGACGGC
TGCGATTTGATGTGCTGCGGCAG?GGGATACAGGACGCAGGAAGTCATCGTCGTCGAAAGGTGCAACTGCACG
TT???????????

Rugilus

??
?????????????????????????????GCCACATACCCATAGAGGGTGCCAGGCCCGATAGCTGGAGGATC
TCTCCTCAGAGTCGGGTTGCTTGAGAGTGCAGCCCTAAGTGGGTGGTAAACTCCATCTAAGGCTAAATATGAC
CACGAGACCGATAGCGAACAAGTACCGTGAGGGAAAGTTGAAAAGAACTTTGAAGAGAGAGTTCAATAGTACG
TGAAACCGTTCAGGGGTAAACCTGAGAAACCCGAAAGGTCGAATGGGGAGATTCAGCGTGTCTCGTTTCTGGT
CGCGTGACGATGGTGCTTGCACCGGGCCGCGCCCGCCGGATCCGTATCCGGCGACGAACTCGTGCACTTCTCC
CCTAGTAGGACGTCGCGACCCGTTGGGCGCCGGTCTAAGGCCGACGGTGGAGCCTTGGGGTCCCGGCCGGCCC
GCTCGACGGTAAGACAGAGGCGTGGGGTCGCTACGTTAGCGTCCGGCCCGTCACAAGTTCGGGCGACTCGGAT
GTCGGACCTGCGTGCCGACCCCGAGCTCGCCGGCTGCTGGTGACGGTGTCCTCGGACAGACTACACGCCGGTC
GGCGACGCTCTAGCTTTGGGTTTTCAGGACCCGTCTTGAAACACGGACCAAGGAGTCTAGCATGTGCGCGAGT
CATTGGGACCGCATCTAAACCTAAAGGCAAAA??
???CTTACACCGTGTTCGCTGACTTGTTCGA
?TCCCATTATTGAAGATTACCATACTGGATTCAAAAAGACTGACAAGCACCCACCTAAGAATTGGGGTGATGT
AAATACCTTTGCAAATCTTGATCCCGCTGGTGAATACGTAGTATCCACCCGCGTTCGTTGCGGCCGTTCAATG
GAAGGTTATCCTTTCAACCCATGCTTAACTGAAGAGCAATATAAAGAAATGGAAGGCAAAGTCTCAAGCACTT
TGTCCGGCCTCGAAGGTGAACTCAAGGGTACTTTCTATCCGTTGACTGGAATGGATAAAGATACTCAGCAAAA
GCTAATCGATGACCACTTCTTGTTCAAGGAAGGTGATCGCTTCCTTCAGGCTGCTAATGCCTGTCGTTTCTGG
CCATCCGGCCGTGGTATCTACCACAACGACAACAAAACCTTCTTGGTCTGGTGCAACGAAGAAGATCATCTTC
GTATCATCTCCATGCAGATGGGTGGTGATCTTGGTGAAGTCTATCGTCGTCTTGTATCTGCTGTTAACGAAAT
TGAGAAGCGTGTTCCATTCTCCCACAATGACAGATTAGGGTTCCTCACTTTCTGCCCAACCAATTTGGGCACA
ACTGTACGTGCCTCTGTACACATCAAAGTACCTAAACTTGCTGCCAACAAGGCCAAGCTC?????????????
????????????????????????????????????GTACCTAAAAAAGTTTAATAATTGGTTCAGGTGGTTT
GTCCATTGGTCAAGCTGGAGAATTTGATTATTCAGGGTCTCAAGCTATAAAAGCTTTGCAAGAAAACAATATT
CAAACAGTTTTAATTAACCCAAACATTGCAACTGTACAAACATCGAAGGGTTTAGCTGATAAAGTATACTTTC
TACCTTTAGTGCCTGAATTCGTGGAACAAGTAATTAGAGTAGAACGTCCTGGAGGTGTTTTGTTAACATTTGG
TGGACAAACAGGGTTAAATTGTGGTGTAGAATTAGAAAAAGCTGGTGTATTTGAGAAATACAATGTTAAAATT
TTGGGTACACCAATACAAGCAATAATAGATACTGAAGATAGAAAGATTTTTAGTGAAAGAATAGCATTGATTG

GTGAAAAAGTTGCTCCTAGCATGGCTGCTTATTCAGCACAGGAAGCTTTAGATGCTGCAGATTTGTTAGGTTA
CCCAGTTATGGCAAGAGCTGCCTTTTCTTTAGGTGGATTAGGGTCTGGGTTTGCTAATACAGCTGAAGAACTG
AAATTACTTGCTCAACAAGCTTTAGCTCATTCCACTCAGTTAATTATCGATAAGTCTTTAAAAGGATGGAAAG
AAGTTGAATATGAAGTTGTTAGAGATGCATATCCCTACCTCCAAGAAGTTAATGATGAAGAACTACAAGAACC
TACAGATAAAAGAATGTTTGTTCTAGCAGCAGCTTTAAGAAATGGTTATAGTGTAGATAAATTATATGATTT?
AACAAAAAATTGATCGCTGGTTCTTGCAAAAAATGAAGAATATTATAGATTACAACACTCTTCTTGAGAAAGTT
CAACAAAACAAATTACAAAACTGCTCAAATACATACAAACTTCTATTGAAAGCGAAACAAATTGGTTTTAGTG
ATAAACAAATTGCTGTTGCTGTTAAAAGCACAGAACTTGCAATTAGAAAACAACGACAAGATTTTGGAATTAC
TCCGTATGTTAAACAAATTGATACTGTGGCTGCTGAATGGCCTGCGACTACAAATTATCTGTACCTAACATAC
AATGCAGAAAGTCATGATTTACATTTTAGTGATCAACACATAATGGTTATTGGTTCTGGAGTTTATAGAATTG
GAAGTTCTGTTGAGTTTGATTGGTGTGCTGTTGGATGTTTGAGGGAGCTTAGGAAATTAAATAAAAAGACAAT
AATGGTTAATTACAATCCAGAGACTGTGAGTACAGATTATGACATGTCAGATCGGTTGTACTTCGAGGAAATT
TCATTTGAAGTTGTAATGGATGAAGTTTACATTTTAATTCTTCCAGGATTTGGAATAATTTCTCATATTATTA
GACAGGCTAGAGGAAAAAAGGAAACATTTGGTTCATTAGGAATAATTTATGCTATAATAGCAATTGGATTACT
TGGATTCGTTGTATGAGCACATCATATATTTACTGTAGGAATAGATGTAGATACACGAGCTTACTTTACTTCA
GCAACAATAATTATTGCTGTTCCTACTGGAATTAAAATTTTCAGATGACTTGCTACTCTTCATGGAACTCAAG
TTAAATATACCCCACCAATATTATGATCCTTAGGATTTGTATTTTTATTTACAATTGGCGGACTAACAGGTAT
TATTTTAGCTAATTCCTCAGTTGATATTATTTTACATGATACATATTATGTTGTTGCCCATTTTCATTATGTA
CTCTCAATAGGAGCTGTATTTGCTATTATAGCCGGTTTAGTGCAATGATTTCCCCTATTTACTGGATTAACAA
TAAATGAATATTTACTAAAAATTCAATTTTTTTAATATTTACTGGCGTAAATCTAACATTTTTTCCTCAACA
TTTTTTAGGATTAGCTGGCATGCCTCGTCGATACTCAGATTATCCGGATGCTTATACCCCATGAAATGTAATT
TCATCAATCGGTTCATTAATCTCAATAATTTCAATTTTTATTTTATTGTTTATTATCTGAGATAGATTTATTT
CTATGCGAATAAATTTATCAGCAAAAAATTTTGCAACTTCAATTGAATGATTTCAACTATTTCCACCAGCTGA
ACATAGCTACTCAGAGCTACCTCTGCTAATTAAAGATTATGTGGTAGTATTTGATTTCCTTGGT?AAGGATTC
TATTAGATATTATAATGAAGTACCTGTAGAGAAACGTGTCTTCAAAAATCTCCAATTGTTTATGGAAAACAAG
TCGCCAGYTGATGATTTGTTTGATAGATTAAACACAGCTGTGATGAACAAACATTTAAATGAGTTAATGGAAG
GCCTAACAGCAAAGGTGTTTCRTACTTATAACGCTTCGTGGACTTTACAACAGCAACTTGAGAAATTGACCAA
TCCAGATGATTCCATATCCGAAWWWWTTTTATCTTACAACCGTGCCAATAGAGCAGTAGCTATACTTTGTAAC
CATCAACGTGCTGTACCTAAGGRTCATCAAAAATCAATGGAAAAACTAAAGGAKAAGATTGATACTAAAAGAG
ACAATATTAAAGATGCCGAGAGGCAGGTCAAAGATGCACAAAGAGATGCAAAGCA???TGGAAGTGTCAAAGA
GAAACAGATTTATGACAAGAAGAAGAAGATGCTCGAGAGACTCAAGGAGCAACTGGCTAAACTGGAAATTCAG
GAGACTGACCGTGATGAAAACAAAACTATTGCTCTTGGCACCTCCAAGTTGAACTATTTAGACCCTAGAATTT
CGGTCGCTTGGTGCAAGAAATTTGATGTGCCCATTGAAAAAATATATAACAAAACTCAA??????CGGCTGCC
GCCGTTCCGCGTCATCGGCGACCACCTGAAGGACCGCTTCGACGGCGCTTCGCGCGTCATGCTCAGCAACTCT
GCCAGCTCCAGGGG????????????GAACGC???GAACCGCC???CRAAACAAGACAAGCTGTCGAACAGCA
TCGCCTCGAACAGCATACACAGCAAAAGGGAGAACAGGCCGCGCAAGTACAAGTACGGGTTCCAGCTGAAGCC
GTACAACCCGGACCACAAGCCGCCCAGTCCCAAGGACCTGGTGTACCTGGAGCCGTCCCCCGGATTCTGCGAG
AAGAACCCGAAGCTGGGCATCCAGGGTACCCACGGTAGGCTG?TGCAACGACACCTCCATCGGTGTGGACGGG
TGCGATCTGATGTGCTGCGGCAG?GGGGTACAGGACCCAGGAGGTCATCGTGGTCGAGAGGTGCAACTGCACG
?????????????

Sciocharis

GGGAGAAGCCCAGCACTGAATCCCGTGGCCGAACCGGGAAATGTAGTGTTTGGGAGGGTCCGTTAGCCACCGT
ACGACGCGTCCAAGTCCTTCTTGAACGGGGCCACATACCCATAGAGGGTGCCAGGCCCGGTAGCTGGCGGATC
TCTCCTCAGAGTCGGGTTGCTTGAGAGTGCAGCCCTAAGTGGGTGGTAAACTCCATCTAAGGCTAAATATGAC
CACGAGACCGATAGCGAACAAGTACCGTGAGGGAAAGTTGAAAAGAACTTTGAAGAGAGAGTTCAATAGTACG
TGAAACCGTTCAGGGGTAAACCTGAGAAACCCGAAAGGTCGAATGGGGAGATTCAGCGTGTCTCGTGTCTGGT
TGCGTGACGGTGGTGCTCGCACCGGAATGCGCCTCCCGGATCCGTAACCGGCGACGAACTCGTGCACTTCTCC
CCTAGTAGGACGTCGCGACCCGTTGGGTGCCGGTCTAAGGCCGACGGTGGAGCCTTGGGGTCCCGGCCGGCCC
GCTCGACGGTAAGACAGAGGCGTGGGGTCGCTACGTTAGCGTCCGGCCCGTCACAAGTTCGGGCGACTCGGAT
GTCGGACCTGTGCGCCGACCCCGAGCTCGCCGGCTGTTGGTGGCGGTGTCCTCGGACAGACTACACGCCGGTC
GGCGACGCTTTAGCTTTGGGTTTTCAGGACCCGTCTTGAAACACGGACCAAGGAGTCTAGCATGTGCGCGAGT
CATTGGGACCGCATCTAAACCTAAAGGCGAAATGAAAGTGAAGGCGTGCCGAGRGAGGATGGGTCGGGGGGCG
TCTCGTTCTCATCGCGAGATGAGGCGCACCCAGAGCGTACACGCGCTTACACCGTATTCGCTGATTTGTTCGA
?TCCCATTATTGAAGACTACCATGGTGGTTTCAAGAAGACCGACAAACACCCCCCTAAGAACTGGGGTGATGT
CAACACCTTCGCTAATCTTGACCCTGCTGGTGAATACGTTGTCTCCACTCGTGTCCGTTGCGGACGTTCAATG
GAAGGTTATCCATTCAACCCATGCTTAACCGAGGAACAATACAAAGAGATGGAAGGTAAAGTTTCCAGCACTT
TGTCTGGCCTCGAAGGCGAACTTAAGGGTACTTTCTACCCATTGACTGGAATGGATAAAGACACCCAACAGAA

ACTCATCGATGATCACTTCTTGTTCAAGGAAGGTGATCGTTTCCTCCAGGCTGCTAATGCCTGCCGTTTCTGG
CCAAGCGGACGTGGTATCTACCACAACGACAACAAAACATTCTTGGTCTGGTGCAACGAAGAAGATCATCTTC
GTCTCATTTCCATGCAGATGGGTGGTGATCTTGGTGAAGTATATCGTCGTCTTGTTACTGCTGTCAACGATAT
TGAGAAGCGCGTTCCTTTCTCCCATAATGACAGATTAGGTTTCTTACTTTCTGCCCAACCAACTTGGGCACA
ACTGTACGTGCCTCTGTACACATTAAAGTACCTAAGCTCGCCGCCAACAAAGCTAAGCTCGATGAAATTGCTG
CCAAATACAACTTGCAAGTACGTGGTACCCGTGGTATTCCTAAAAAAGTTTTGATTATTGGCTCAGGTGGTTT
ATCAATAGGACAAGCTGGAGAATTTGATTATTCTGGATCACAAGCAATAAAAGCTTTACAAGAAGAAAATATT
CAAACTGTTCTAATCAATCCAAACATAGCCACTGTACAAACATCTAAAGGTTTAGCCGACAAAATTTACTTCC
TACCTTTAGTGCCTGAATTTGTAGAGCAAGTTATTAGAGCAGAACGCCCTGGTGGTGTTTTACTAACATTTGG
TGGTCAAACAGGGTTAAATTGTGGTGTAGAGTTACAGAGAGCCGGTGTTTTTGAAAAGTATGGTGTTCAAATT
CTGGGTACACCTATACAAGCCATTATTGATACGGAAGACAGAAAAGTTTTCAGTGAAAGAATTGCACAAATTG
GTGAAAAAGTTGCTCCAAGTATGGCTGCTTATTCTGTGCAAGAAGCACTGGAAGCAGCAGAATTATTGGGATA
CCCTGTGATGGCAAGAGCTGCTTTTTCATTGGGAGGGTTAGGTTCTGGGTTTGCAGATACAGCTGAAGAATTA
AAATCTCTAGCGCAACAAGCTTTAGCACATTCCAATCAATTAATTATTGATAAGTCTTTGAAAGGTTGGAAAG
AAGTTGAATATGAAGTTGTTAGAGATGCATATCCATATCTAAAAACAGTAAATGATGAGGAACTCCAAGAACC
AACAGATAAAAGAATGTTTGTTTTGGCTGCTGCTTTAAGAAATGGATATAGTGTTGAAAAATTATATGAGTT?
AACAAAAATTGATCATTGGTTTTTACAGAAAATGAAAAATATTGTGGATTATAATACTCTCTTAGAATCAATT
CAACAACATAAATTG???????????????ACACACAAACTTTTATTAAAAGCGAAACAAATTGGATTTAGTG
ACAAACAAATAGCTGTTGCTGTTAAAAGCACCGAACTTGCTATAAGAAAGCAACGACAAGATTTTGAGATAAC
TCCATATGTTAAACAAATTGATACCGTTGCTGCTGAATGGCCTGCAACAACAAATTATCTATATTTAACATAT
AATGCTGAAACCCATGATCTAACATTTTGCGATCAACATATAATAGTTATTGGATCAGGAGTTTATCGAATTG
GAAGTTCAGTAGAGTTTGATTGGTGTGCTGTAGGATGCTTAAGAGAACTCCGAAAATTAAACAAAAAAACAAT
AATGATAAATTACAATCCAGAAACTGTTAGTACAGATTACGATATGTCCGACCGATTGTATTTTGAAGAAATT
TCATTCGAAGTAGTTATGGATGAAGTTTATATTTTAATTTTACCTGGATTTGGGTTAATTTCCCATATTATTA
GACAAGCTAGAGGAAAAAAAGAAACTTTTGGATCTTTAGGGATAATTTATGCTATACTTTCAATTGGTTTATT
AGGATTTGTTGTTTGAGCCCACCATATATTTACTGTTGGAATAGATGTGGATACCCGGGCTTATTTCACTTCA
GCTACTATAATTATTGCGGTTCCAACAGGAATTAAAATTTTTAGATGATTAGCTACTTTACATGGCACCCAAA
TTAAATTTACCCCCCCTATGTTATGAGCTTTAGGGTTTGTTTTTTTATTTACAATTGGGGGTTTGACAGGAAT
TATTCTTGCTAACTCTTCAATTGATATTATTTTACACGATACTTATTATGTTGTCGCCCATTTTCATTATGTT
TTATCTATAGGGGCTGTTTTTGCAATTATAGGAGGATTAGTTCAATGATACTCTTTATTTACTGGTTTAACTT
TAAATGAACACTTTTTAAAAATTCAATTTTTTATTATATTTATTGGAGTTAATGTAACATTTTTCCCTCAACA
TTTTCTTGGATTAGCAGGAATACCTCGGCGATATTCAGATTACCCGGATGCTTATACTTCTTGAAATGTTATT
TCTTCAATTGGTAGATTAATTTCTATAGTTTCAATTTTTATTTTATTATTTATTATTTGAGAAAGATTCACTT
CAATACGAATAGTTATTTCAGCTACTAATTTTGTCACATCAATTGAATGAATGCAAAAATACCCCCCTGCCGA
ACATAGATATTCAGAATTACCTATATTAACAAGAGATTACGTGGTGGTATTTGATTTTCTTGGT?AAGGATTC
CATTAGATATTACAATGAAGTACCTGTGGAAAAACGTGTCTTCAAAAACCTTCAATTGTTTATGGAAAATAAA
TCTTCAGGAGATGATTTATTTGATAGGCTGAATACCGCTGTGATGAACAAACATTTAAACGAGTTAATGGAAG
GTCTAACTGCCAAGGTGTTTCGTACTTATAACGCTTCCTGGACTTTACAGCAGCAACTCGAAAAATTGACCAA
TCCCGATGATTCCATATCCGAAAAAATTTTATCTTACAACCGAGCTAATAGAGCTGTAGCTATACTTTGTAAC
CATCAACGTGCTGTACCGAAAGGCCACCAGAAATCCATGGAGAAGCTAAAAGAAAAGATAGAAGCCAAAAGGG
AGAATATCAAGGATGCCGAGAGACAAGTGAAAGACGCACAAAGGGATGCCAAGCA???CGGAAGTGTTAAGGA
GTCCCAGATATACGATAAGAAAAGGAAAATGTTGGAGAGGCTAAAGGAACAGTTGGCAAAGTTGGAAATTCAA
GAGACGGACCGTGACGAAAATAAAACTATTGCCCTCGGCACGTCCAAGTTGAACTATTTAGATCCTAGAATTT
CAGTTGCTTGGTGTAAGAAGTTTGGTATACCCATCGAAAAGATTTATAACAAAACACAATGGATGCGGTTGCC
ACCCTTCAGAGTGATCGGCGATCATCTAAAGGACCGTTTCGATGGTGCCTCCCGAGTGATGCTCAGCAATTCG
GCAAGTTCGAG???????????????GAACCA???AAATCGAC???CGAAGCAAGATAAACTGTCGAACAATA
TCGCGTCTAACAGCATTCATAGTAAAAGAGAGAACCGCCCGCGAAAATACAAATACGGTTTCCAACTGAAACC
TTACAATCCTGATCATAAGCCTCCGAGTCCCAAGGATTTAGTATACTTGGAACCGTCACCTGGTTTCTGCGAG
AAAAATCCAAAGCTTGGTATACAGGGTACTCACGGTAGATTG?TGCAATGATACGTCTATAGGCGTTGATGGG
TGCGACTTAATGTGTTGCGGTAG?AGGCTACAGGACCCAGGAAGTTATTGTTGTTGAAAGATGCAACTGCACT
TTCC?????????

Scioporus

GGGAGAAGCCCAGCACTGAATCCCGTGGCCGAACCGGGAAATGTAGTGTTTGGGAGGATCCATTATCCATCGT
ACGACGCGTCCAAGTCCTTCTTGAACGGGGCTAAATACCCATAGAGGGTGCCAGGCCCGGTAGCTGGTGGATC
TCTCCTCAGAGTCGGGTTGCTTGAGAGTGCAGCCCTAAGTGGGTGGTAAACTCCATCTAAGGCTAAATATGAC
CACGAGACCGATAGCGAACAAGTACCGTGAGGGAAAGTTGAAAAGAACTTTGAAGAGAGAGTTCAATAGTACG
TGAAACCGTTCAGGGGTAAACCTGAGAAACCCGAAAGGTCGAATGGGGAGATTCAGCGAGGCTCGTTTCTGGT

TGCGTGACGATTGTGCTTGCACTTGGCTGCGCCTTCCGGATCCGCAATCGGCGACGGACTCGTGCACTTCTCC
CCTAGTAGGACGTCGCGACCCGTTGGGCGCCGGTCTAAGGCCGACGGTGGAGACTTTGGGTCCCGGCCGGCAC
GCTCGACGGTAAGACAGAGACGTGGGGTCGCTACGTTAGCGTCCGGCCCGTCACAAGTTCGGGCGTCTCGGAT
GTCGGACCTGTGTGCCGACCTCGAGCGCGCCGGCTGTTGGTGGCGGTGTCCTCGGACAGACTACACGTCGGTC
GGCGACGCTTTAGCTTTGGGTTTTCAGGACCCGTCTTGAAACACGGACCAAGGAGTCTAGCATGTGCGCGAGT
CATTGGGACCGCATCTAAACCTAAAGGCAAAATGAAAGTGAAGGCGTGCCGAGGGAGGATGGGTCGGGGGGCG
TCTCCTTCTCATCGCGAGATGAGGCGCACCCAGAGCGTACACGC?????????????????TGATTTGTTCGA
?TCCCATCATTGAAGACTACCATGGTGGATTCAAGAAGACCGATAAGCACCCTCCCGCAAACTGGGGTGATGT
CAACACCTTCGCTAACCTCGACCCTGCTGGTGAATACGTAGTCTCCACCCGTGTTCGTTGCGGCCGCTCCATG
GAAGGCTATCCTTTCAATCCATGCTTAACCGAAGAACAATACAAGGAAATGGAACAAAAAGTTTCCAGCACTT
TGTCCGGCATGGAAGGAGAACTTAAGGGTACTTTCTACCCATTGACTGGAATGGATAAGGCTACTCAACAGAA
GCTTATTGATGATCATTTCTTATTCAAGGAAGGTGATCGTTTCCTTCAAACTGCTAACGCCTGTCGTTTCTGG
CCATCTGGACGTGGTATCTACCACAATGATAATAAAACCTTCTTGGTTTGGTGCAACGAAGAGGATCATCTTC
GTATCATCTCTATGCAGATGGGTGGTGATCTTGGTGAAGTCTACCGTCGTCTTGTAAGCGCTGTCAACGAAAT
TGAAAAACGCGTACCATTCTCTCATAATGACAGATTAGGTTTCCTTACTTTCTGCCCGACTAACTTGGGCACA
ACAGTACGTGCCTCTGTACACATTAAAGTACCTAAGCTCGCTGCCAATAAGGCTAAACTCG????????????
???????????????????????????????????GTACCTAAAAAAGTACTAATAATTGGTTCAGGAGGTTT
ATCTATAGGACAAGCTGGTGAATTTGATTATTCTGGCTCACAGGCGATAAAAGCGTTGCAAGAARAAAACATT
CAAACGGTTTTAATCAATCCCAACATTGCTACTGTACAAACATCGAAAGGCTTGGCTGATAAAGTTTACTTTT
TACCATTAGTGCCTGAATTTGKTGAAGAAGTAATTAGAGTAGAACGCCCTGGAGGTGTTCTACTAACATTTGG
TGGACAAACAGGGTTAAATTGSGGSGTAGAGTTACAAAAAGCTGGTATTTTTGAAAAATATGGTGTCAAAATC
CTAGGTACACCTATAGAAGCCATAATTGATACAGAAGACAGGAAAATTTTTAGTGATAGAATTTCTTTAATTG
GTGAAAAGGTCGCTCCAAGTATGGCTGCGTATTCAGTACAAGAAGCACTTGAAGCTGCAGAATTGTTAGGCTA
CCCAGTAATGGCAAGAGCTGCATTTTCTTTAGGTGGTTTAGGGTCTGGTTTTGCTAATACCGATGAGGAACTG
AAATCACTTGCTCAACAAGCTTTAGCTCACTCAAATCAATTGATTATTGATAAATCTTTGAAAGGATGGAAAG
AAGTTGAGTATGAAGTTGTACGGGATGCATATCCATATTTAAAAGATGTTGATGATGAAGAATTGAAAGAACC
TACAGATAAAAGAATGTTTGTACTGGCTGCTGCT????GGAGTCSCTACMCAATCGATAAATTATATGATTT?
AACTAAAATCGATCGTTGGTTCTTACAAAAAATGAAGAAGATTGTAGACTATAACACATATTTGGAATCAATT
CAACAGAATAAATTA????????????????ACGTTTAAAATGTTACTAAAAGCGAAACAAATTGGTTTTAGCG
ATAAACAAATTGCTGTAGCTGTTAAAAGCACTGAGCTTGCTATTAGAAAGCAACGCCAGGATTTCAATATAAC
TCCATATGTTAAACAGATTGATACAGTTGCTGCTGAATGGCCGGCTACCACAAATTACCTCTATCTAACGTAC
AATGCAGAAAGCCATGATATATCTTTTAATGACCAACATATAATAGTTATTGGTTCCGGTGTTTACAGAATTG
GAAGTTCAGTTGAATTCGATTGGTGTGCTGTTGGGTGCCTAAGGGAACTTCGAAAATTAAATAAAAAAACTAT
AATGATTAATTATAATCCTGAGACTGTTAGTACAGACTATGATATGTCAGATAGACTATATTTTGAAGAAATA
TCGTTTGAAGTAGTTATGGAT????????????????TTTTACCTGGATT??GGATAATTTCTCATATTATTA
GTCAATCTAGAGGAAAAAAAGAAACATTTGKAAYTTTARGAATAATCTATGCTATAATAGCTATTGGATTATT
AGGATTTGTTGTTTGAGCTCATCATATATTTACAGTMGGAATARATGTTGATACACGAGCTTATTTTAAAAGT
CCAACAAMYATTATTGCTGTTCCTCCMKGAATTAAAATTTT????????????????????????????????
???
???
???
???
???
???
???
???TCTTTGATTTCTTGGTAAGGATTC
CATTAGATACTACAATGAAGTACCTGTAGAAAAACGTGTCTTCAAAAATCTTCAATTATTCATGGAAAATAAA
TCGCCAGGAGATGATTTGTTTGATAGATTAAATACAGCTGTGATGAACAAACATTTAAATGAACTAATGGAAG
GTTTAACTGCCAAGGTGTTTCGTACTTACAATGCTTCTTGGACTCTACAACAACAACTCGATAAATTGACCAA
TCCAGATGATTCCATATCCGAAAAAATATT???
???
???
???
???
???
???????????????????GACCATTTAAAAGACCGCTTTGACGGTGCATCGAGGGTAATGTTAAGCAACTCA
GCTAGTTCCCGTGG????????????AAATGC???AAACCGCC???CTAAACAAGACAAACTATCAAACAACA

TCGCTTCAAACAGCATACACAGTAAAAGAGAAAACCGCCCTCGCAAATACAAATATGGGTTCCAATTAAAACC
ATATAATCCAGACCACAAACCTCCAAGTCCTAAAGATTTAGTATACTTGGAGCCATCACCTGGTTTCTGCGAG
AAGAACCCGAAGCTTGGGATCCAAGGTACACATGGTAGGTTA?TGTAATGATACTTCAATTGGTATTGATGGA
TGCGATTTGATGTGCTGCGGAAG?GGGATACAGGACCCAGGAAGTTATTGTAGTCGAA???????????????
?????????????

Scopaeus

?????AAGCCCAGCACTGAATCCCGTGGCCGAACCGGGAAATGTAGTGTTTGGGAGGGTCCGTTATCCATCGT
GCGACGCGTCCAAGTCCTTCTTGAACGGGGCCATATACCCATAGAGGGTGCCAGGCCCGATAGCTGGTGGATC
CCTCCTCAGAGTCGGGTTGCTTGAGAGTGCAGCCCTAAGTGGGTGGTAAACTCCATCTAAGGCTAAATATGAC
CACGAGACCGATAGCGAACAAGTACCGTGAGGGAAAGTTGAAAAGAACTTTGAAGAGAGAGTTCAATAGTACG
TGAAACCGTTCAGGGGTAAACCTGAGAAACCCGAAAGTTCGAATGGGGAGATTCAGCGTGTATCGTGTTTGGT
CGCGTGACGATGGTGCTTGCACCTGGCTGCGCCTTCTGAGCCCAATTCTTACTGCGAGCGCGTGCACTTCTCC
CCTAGTAGGACGTCGCGACCCGTTGGGCGCCGGTCTACGGCCGATGGAGGAGACTTGAGGTCCCGGCCGGCCC
GCTCGACGGTAGGACAGAGACGTGGGGTCGCGACGTTCGCGTCCGGCCCGTCACAAGTATGGGCGACCTGGAC
GCCGGACCTATGTGCCGGCCCCGGGCCCGACTGCTGCTGGTGGCGGTGTCCTCGGACAGACTGCACGTCGGTC
GGCGACGCTTTAGCTTTGGGTTTTCAGGACCCGTCTTGAAACACGGACCAAGGAGTCTAGCATGTGCGCGAGT
CATTGGGACTTGA??TAAACCTAAAGGCGAAATGAAAGTGAAGACCTGTCGAGGGAGGATGGGTCGGGGGGCG
TCTCATTCTCATCACGAG????????????????????????????CTTACACAACATTCGCCGACTTGTTCGA
?CCCCATCATCGAAGACTACCATGGTGGTTTCAAGAAGACCGACAAACACCCACCTGCTAACTGGGGTGATGT
CAGCACTTTCGGCAATTTGGACCCAGCTGGTGAATACGTCGTCTCCACCCGTGTCCGTTGCGGCCGTTCAATG
GAAGGCTATCCATTCAACCCATGTTTAACTGAAGACCAATACAAGGAGATGGAACAAAAAGTTTCAGCCACTT
TGTCTGGTTTGGAAGGTGAATTGAAGGGTACTTTCTACCCATTGACTGGAATGAGCAAGGATGTTCAACAGAA
ACTCATCGACGATCATTTCTTGTTCAAGGAAGGTGATCGCTTCTTGCAAACTGCTAACGCTTGCCGTTACTGG
CCAAGCGGACGTGGTATCTACCACAACGACAACAAGACTTTCTTGGTCTGGTGCAACGAAGAAGATCACCTTC
GTATCATCTCCATGCAAATGGGTGGTGATTTAGGTGAAGTATACCGTCGCCTTGTCACCGCTGTCAACGAAAT
CGAGAAGCGCGTCCCATTCTCTCATAACGACAGATTAGGTTTCCTTACCTTCTGCCCAACCAACTTGGGTACA
ACTGTCCGTGCCTCTGTACACATCAAGGTGCCCAAACTCGCCGCTAACAAGGCTAAACTC?????????????
???????????????????????????????????GTTCCTAAAAAAGTATTAATCATTGGTTCAGGTGGCTT
ATCAATCGGTCAAGCTGGAGAGTTTGATTATTCTGGATCTCAAGCTATTAAAGCTTTACAAGAAGAGAACATA
CAAACAGTGTTGATCAATCCCAATATAGCTACTGTACAAACATCTAAAGGACTTGCTGATAAAGTATATTTTT
TACCGTTAGTGCCCGAATTTGTTGAACAAGTAATTAGAGTTGAACGTCCAGGAGGGGTTTATTAACCTTTGG
GGGTCAAACAGGTTTAAATTGTGGAGTTGAACTTCAAAAAGCTGGTATTTTTGAAAAATATGGTGTTAAAATA
TTGGGTACTCCCATACAAGCTATCATAGACACGGAAGATCGTAAAGTTTTTAGCGACAGAATATCGTTAATTG
GTGAGAAAGTAGCACCAAGTATGGCTGCTTATTCTGTGCAAGAAGCTTTAGAAGCAGCCGAGTTGTTAGGTTA
CCCTGTTATGGCTAGGGCAGCTTTCTCGTTGGGTGGTTTAGGATCTGGTTTTGCTACACAGCT??ATGAACTA
AAATCACTTGCTCAACAAGC???
????????????????????????????????CCTTATCTTAAAGAAGTAAATGATGAAGAATTACAAGAACC
AACAGACAAACGTATGTTTGTACTTGCTGCTGCTTTAAGAAATGGTTATTCTGTGGATAAATTATACAATTT?
AACAAAAATTGATCGTTGGTTCTTGCAAAAAATGAAAAACATTGTTGATTTTAATACGTACTTGGAAACCGTT
???CAGAACAAACTT???????????????ACTTTTAAAGATTTGTTGAAAGCTAAGCAAATTGGTTTTAGCG
ATAAACAAATTGCCGTTGCTGTGAAAAGTACCGAACTAGCAGTTAGAAAACGTAGAAAAGATTTCGATATAAC
ACCATATGTAAAACAAATAGACACTGTTGCTGCTGAATGGCCTGCTACTACCAATTATTTATATTTAACTTAC
AATGCTGAAAGTCATGATTTAACCTTTAACGATGAACATATTATGGTTATTGGATCTGGTGTTTATAGAATTG
GAAGTTCTGTGGAGTTTGATTGGTGTGCTGTTGGTTGTTTGCGAGAACTTAGAAAACTAAATAAAAAAACTAT
TATGGTCAACTATAATCCCGAAACTGTTAGTACAGATTATGATATGTCTGATAGACTATACTTTGAAGAAATA
TCATTTGAAGTCGTAATGGATGAAGTATACATTTTAATCCTCCCAGGGTTTGGAATAATTTCCCACATTATTA
GACAAGCTAGAGGCAAAAAAGAAACTTTTGGATGTCTGGGAATAATTTATGCAATAATAGCAATCGGATTATT
GGGATTTATTGTATGAGCTCACCATATATTTACAGTTGGCATAGATGTTGACACTCGGGCCTATTTTACATCA
GCTACTATAATCATTGCTGTACCTACAGGTATCAAAATTTTTAGATGGTTAGCAACCCTTCATGGAAGACAAA
TTAATTTTAATCCTCCTATATTATGATCCCTAGGGTTTGTCTTTTTATTCACTATTGGGGGGTTGACAGGAGT
TATCTTAGCCAATTCATCAATTGATATTATTCTTCATGATACTTACTACGTAGTAGCCCACTTTCACTATGTA
CTTTCAATAGGAGCTGTATTTGCAATCATAGCTGGATTAGTCCAATGATTCCCTTTATTCACTGGATTAACCA
TAAATGAGTACCTTTTAAAGATTCAATTTTTCATTATATTTATTGGGGTAAATCTAACATTTTTCCCCCAACA
TTTTTTAGGACTAGCAGGTATACCTCGACGATACTCTGACTACCCAGATGTTTATACTCCGTGAAATGTAATT
TCATCAATCGGCTCCTTAGTCTCTATAGTTAGAATTTTTTTATTAATTTTTATCATTTGAGAAAGATTCTCAT
CTCTTCGATTAGTGGTATCCTCAAAAAACTTCTGCACATCCATTGAATGATTCCAATTTTATCCCCCCTCTGA
ACACTCTTATTCTGAATTACCAATAATTATTAAAGACTATGTTGTTGTATTTGATTTCCTCGGA?AAGGATTC

CATTAGATATTACAATGAAGTACCTGTAGAGAAACGTGTTTTCAAAAACCTTCAATTGTTCATGGAAAACAAA
TCTCCAGGTGATGATTTATTCGATCGATTAAACACAGCTGTGATGAACAAACACTTAAATGAATTGATGGAAG
GGTTAACGGCCAAAGTGTTCCGTACTTATAACGCCTCCTGGACTTTACAGCAACAACTTGAAAAACTCACAAA
TCCCGATGATTCCATATCCGAAAAAATCTTATCATACAACCGAGCCAATCGTGCCGTTGCTATTTTATGTAAC
CATCAACGTGCAGTCCCTAAAGGTCACCAAAAATCCATGGAAAAACTTAAAGAAAAAATCGATGCCAAACGAG
ACGCTATACGTGATGCCGAACGTATGGTTAAAGATGCCCAGAGAGATGCTAAACA???TGGAAGTGTGAAAGA
AAAACAAATCTACGATAAAAAGAAGAAAATGTTGGAAAGATTGCGTGAACAACTCGCTAAATTAGAGATACAA
GAAACTGATCGCGATGAAAATAAAACTATTGCTCTTGGTACGTCCAAGTTGAATTATTTAGATCCTAGGATCT
CTGTCGCTTGGTGTAAGAAGTTCGGTGTGCCCATCGAAAAGATTTACAATAAAACTCAA??????CGTCTACC
ACCGTTCCGTGTCATCGGCGATCACCTGAAGGACCGTTTCGACGGCGCATCCCGGGTGATGCTTTCRAATTCC
GCCAGTTCCAACCG????????????CAACGC???CAATCGTC???CGAAACAAGATAAACTATCAAACAGCA
TAGCTTCCAACAGCATCCACAGCAAACGCGAAAATCGACCGAGAAAATATAAATACGGTTTYCAATTAAAACC
GTATAATCCTGATCATAAACCGCCGAGTCCTAAAGATTTAGTTTACTTGGAACCGAGTCCCGGTTTTTGTGAG
AAAAATCCCAAGTTGGGGATACAGGGTACGCATGGTAGACAG?TGTAATGATACTTCTATTGGAGTCGACGGG
TGCGATTTGATGTGTTGCGGGAG?AGGTTACAGAACACAGGAGGTGGTTGTTGTTGAACGGTGCAATTGCACG
?????????????

Stilicoderus

GGGAGAAGCCCAGCACTGAATCCCGTGGCCGAATCGGGAAATGTAGTGTTTGGGAGGGTCCGTTAACCATCGT
GCGACGCGTCCAAGTCCTTCTTGAACGGGGCCACATACCCATAGAGGGTGCCAGGCCCGATAGCTGGTGGATC
TCTCCTCAGAGTCGGGTTGCTTGAGAGTGCAGCCCTAAGTGGGTGGTAAACTCCATCTAAGGCTAAATATGAC
CACGAGACCGATAGCGAACAAGTACCGTGAGGGAAAGTTGAAAAGAACTTTGAAGAGAGAGTTCAATAGTACG
TGAAACCGTTCAGGGGTAAACCTGAGAAACCCGAAAGGTCGAATGGGGAGATTCAGCGTGTCTCGTTTCTGGT
CGCGTGACGATGGTGCTTGCACCGGGCTGCGCCTTCCGGATCCGTATCCGGCGACGAACTCGTGCACTTCTCC
CCTAGTAGGACGTCGCGACCCGTTGGGCGCCGGTCTAAGGCCGACGGTGGAGCCTTGGGGTCCCGGCCGGCCC
GCTCGACGGTAAGACAGAGGCGTGGGGTCGCTACGTTAGCGTCCGGCCCGTCACAAGTTCGGGCGACTCGGAT
GTCGGACCTGTGTGCCGACCTCGAGCTCGCCGGCTGTTGGTGACGGTGTCCTCGGACAGACTACACGCCGGTC
GGCGACGCTCTAGCTTTGGGTTTTCAGGACCCGTCTTGAAACACGGACCAAGGAGTCTAGCATGTGCGCGAGT
CATTGGGACCGCATCTAAACCTAAAGGCTAAATGAAAGTGAAGGCGTGCCGAGGGAGGATGGGTCGGGGGGCG
TCTCGTTCTCATCGCGAGTTGAGGCGCACCCAGAGCGTACACGCGCTTACACCGTATTCGCTGATTTGTTCGA
?TCCCATCATTGAAGACTACCATACCGGCTTCAAGAAGAGCGACAAGCACCCGCCCAAGAATTGGGGAGACGT
AAATACTTTCGCCAATCTTGATCCTGCAGGTGAATATGTGGTATCCACCCGTGTTCGTTGCGGTCGCTCCATG
GAAGGCTACCCCTTCAACCCGTGCTTAACCGAAGAGCAATACAAGGAGATGGAAGGCAAAGTCTCCGGCACTT
TGTCCGGTCTCGAAGCCGAACTCAAGGGTACATTCTACCCGTTGACCGGAATGGACAAGGATACTCAACAGAA
GCTCATCGACGATCACTTCTTGTTCAAGGAAGGTGACCGTTTCCTCCAGGCTGCCAACGCCTGTCGCTTCTGG
CCATCCGGACGTGGTATCTACCACAACGACAACAAGACCTTCTTGGTTTGGTGCAACGAGGAGGATCATCTTC
GCATCATCTCGATGCAGATGGGCGGTGATCTTGGCGAAGTCTATCGCCGCCTCGTTACCGCCGTTAACGAAAT
CGAGAAGCGTGTACCGTTCTCGCATAATGACAGATTAGGTTTCCTCACTTTCTGCCCAACAAACTTGGGTACA
ACTGTACGTGCCTCTGTACACATCAAAGTACCTAAACTCGCCGCCAACAAAGCCAAGCTGGATGAAGTCGCTG
CCAAATACAACTTACAAGTACGTGGTACCCGC???GTACCTAAAAAAGTTTTAATAATCGGATCAGGAGGGTT
GTCCATCGGACAAGCTGGAGAGTTTGATTATTCTGGCTCACAAGCTATCAAAGCTTTGCAAGAAGAAAACATT
CAAACAGTGCTAATTAATCCAAACATTGCAACTGTACAAACATCTAAAGGTTTAGCAGATAAAGTTTATTTTT
TGCCTTTAGTGCCTGAATTTGTAGAACAAGTGATTCGAGTGGAGCGTCCTGGAGGTGTTTTATTAACATTCGG
CGGACAAACTGGGTTAAATTGCGGTGTGGAATTACAAAAAGCTGGTATCTTTGATAAATACGGCGTTAAAATT
TTGGGTACACCAATAGAAGCCATTATAGATACTGAAGACAGAAAGATTTTTAGCGAAAGAATAGCATTAATTG
GTGAAAAAGTTGCTCCAAGTATGGCTGCTTATTCAGTACAAGAAGCTTTAGAAGCTGCCGAGTTATTAGGGTA
TCCAGTTATGGCAAGAGCTGCTTTCTCTTTAGGCGGATTAGGGTCAGGGTTTGCTAATACAGCTGAAGAACTG
AAATTACTTGCTCAACAAGCTTTAGCTCATTCCACTCAGTTAATTATTGATAAGTCTTTAAAAGGGTGGAAAG
AAGTTGAATATGAAGTTGTTAGAGATGCATATCCTTATCTAAAGGATGTTAATGATGATGAATTACAAGAACC
TACAGATAAAAGAATGTTCGTCTTAGCAGCGGCTTTAAGAAATGGTTATAGTGTAGATAAATTATATGATTT?
AACAAAAATTGATCGTTGGTTCTTACAAAAAATGAAGAAAATCGTAGATTTTAACACTATTCTCGAAAACATT
CAACAAAATAAATTACAAAATTGCTCAAATACGTATAGGCTTTTATTAAAAGCGAAACAAATTGGATTTAGTG
ATAAACAAATTGCTGTTGCTATTAAAAGCACTGAACTTGCAGTTCGAAAGCAACGACAGGATTTCGGCATCAC
TCCATACGTGAAACAAATTGATACTGTCGCTGCTGAATGGCCAGCAACTACAAATTATCTATATCTAACATAC
AATGCCGAAGATCACGATTTAACTTTCTCAGAACAACACATAATAGTTATAGGATCTGGAGTTTATAGAATTG
GAAGTTCTGTGGAGTTTGATTGGTGTGCTGTAGGGTGTTTGAGAGAATTAAGGAAATTAAATAAAAAGACAAT
AATGGTTAATTATAATCCTGAAACTGTTAGTACAGATTATGATATGTCGGACAGGTTGTACTTCGAGGAAATT
TCATTTGAAGTTGTAATGGAT????????????CTAATTTTACCAGGATTCGGATTAATTTCTCATATTATTA

GTCAATCTAGAGGAAAAAAAGAAACTTTTGGGGCCTTAGGAATAATTTATGCTATAATAGCAATTGGCCTCCT
AGGATTTGTTGTATGAGCACATCACATATTTACTGTAGGTATAGATGTAGATACACGAGCTTATTTTACTTCT
GCTACTATAATTATTGCTGTTCCTACAGGAATTAAAATTTTTAGATGATTAGCTACTTTACATGGAACACAAA
TTAAATTTTCTCCTCCAATATTATGAGCCTTAGGATTTGTATTTTTATTCACAATTGGAGGACTTACAGGAGT
AATTTTAGCTAATTCTTCTATTGATATTATTCTTCATGATACTTATTATGTAGTAGCTCATTTCCATTATGTT
CTTTCTATAGGAGCTGTTTTTGCTATTATAGCAGGATTAGTCCAATGATTCCCCCTATTTACTGGATTAACTT
TAAATGAATATCTTCTAAAAATTCAATTTATTATTATATTTACAGGAGTTAATTTAACATTTTTCCCTCAACA
TTTTCTAGGTCTAGCTGGTATACCTCGTCGTTATTCCGATTACCCTGATGCTTATATACCATGAAATGTAATT
TCTTCTATTGGATCTCTTATTTCGATAATTAGAATTTTCATTCTATTATTTATTATCTGAGAAAGATTCTCTT
CATTTCGAATAAATATTTCAGCAAAAAATTTTTCCACGTCAATTGAATGATTCCAACTTTATCCTCCAGCTGA
ACAT???
???
???
???
???
???
???
???
???
???TGGATGCGGCTGCC
CCCGTTCCGCGTGATCGGCGACCACCTGAAGGACCGGTTCGACGGGGCGTCGCGCGTCATGCTCAGCAACTCG
GCGAGCTCCAGGGG????????????TAGCGC???GAACCGCC???CCAAACAGGACAAGCTGTCGAACAGCA
TCGCCTCGAACAGCATACACAGCAAGCGGGAGAACAGGCCGCGCAAGTACAAGTACGGTTTCCAGCTGAAGCC
GTACAATCCGGACCACAAGCCGCCCAGTCCCAAGGACCTGGTGTACCTGGAACCGTCGCCGGGGTTCTGCGAG
AAGAACCCCAAGYTGGGCATACAGGGCACGCACGGCAGGCTG?TGCAACGACACATCGATCGGGGTGGACGGG
TGCGACCTGATGTGCTGCGGCAG?GGGGTACAGGACCCAGGAGGTCGTCGTCGTCGAGAGGTGCAACTGCACG
TTCCACTGGTGCT

Stilicopsis

GGGAGAAGCCCAGCACTGAATCCCGTGGCCGAACCGGGAAATGTAGTGTTTGGGAGGGTCCGCTATCCATTGT
ACGGTGCGTCCAAGTCCTTCTTGAACGGGGCCACATACCCATAGAGGGTGCCAGGCCCGATAGCTGGAGGATC
TCTCCTCAGAGTCGGGTTGCTTGAGAGTGCAGCCCTAAGTGGGTGGTAAACTCCATCTAAGGCTAAATATGAC
CACGAGACCGATAGCGAACAAGTACCGTGAGGGAAAGTTGAAAAGAACTTTGAAGAGAGAGTTCAATAGTACG
TGAAACCGTTCAGGGGTAAACCTGAGAAACCCGAAAGGTCGAATGGGGAGATTCAGCGTGTCTCGTTTCTGGT
CGCGTGACGATGATGCTTGCATCGGACTGCGCCTTCCGGATCCGTAACCGGCGACGAACTCGTGCACTTCTCC
CCTAGTAGGACGTCGCGACCCGTTGGGTGCCGGTCTAAGGCCGATGGTGGAGCCTTAAAG?CCCGACCGGCCC
GCTCGACGGTAAGACAGAGACGTGGGGTCGCTACGTTAGCGTCCGGCCCGTTACAAGTTCGGGCGACTCGGAT
GTCGGACGTGTGTGCCGACCCCGAGCTCGCCGGCTGTTGGTGACGGTGTCCTCGGACAGACTACACGTCGGTC
GGCGACGCTATAGCTTTGGGTTTTCAGGACCCGTCTTGAAACACGGACCAAGGAGTCTAGCATGTGCGCGAGT
CATTGGGACCGCATCTAAACCTAAAGGCGAAATGAAAGTGAAGGCGTGCCGAGGGAGGATGGGTCGAGGGGCG
TCTCGTTCTCATCGCGAGATGAGGCGCACCCAGAGCGTACACGCTCTTACACCGTATTCGCTGATCTGTTCGA
?TCCCATCATCGAGGATTACCATGGTGGATTCAAGAAGACCGACAAGCATCCACCAGCAAACTGGGGTGATGT
AAATACTTTCGCTAACTTGGACCCGGCTGGTGAATACGTCGTCTCCACTCGTGTCCGTTGCGGACGCTCAATG
GAAGGATACCCGTTCAATCCTTGCTTAACCGAAGAGCAATACAAAGAAATGGAAGGAAAAGTATCAACCACCT
TGTCTGGTCTTGAAGGTGAACTCAAGGGTACTTTCTATCCTTTGACTGGAATGGACAAAACTACTCAACAGAA
GTTGATTGATGATCACTTCTTGTTCAAGGAAGGAGATCGTTTCCTTCAAACCGCGAATGCTTGTCGTTACTGG
CCTTCCGGCCGTGGTATTTACCACAATGATAACAAGACCTTCTTGGTATGGTGTAACGAAGAAGATCATCTTC
GCATTATTTCGATGCAGATGGGTGGTGATCTTGGCGAAGTGTACCGTCGTCTTGTAACAGCTGTTAATGAGAT
CGAAAAGCGCGTTCCATTCTCTCATAACGATAGATTAGGATTCCTCACCTTCTGCCCTACCAACTTGGGT???
???
???????????????????????????????????GTACCYAGAAAAGTGTTAATCATTGGTTCAGGAGGTCT
TTCTATCGGACAAGCTGGTGAATTCGATTATTCCGGTTCGCAAGCGATCAAAGCTTTACAAGAAGAAAACATC
CAAACGGTTTTAATTAATCCAAACATCGCAACTGTACAAACATCGAAAGGTTTATCCGATAAAATATATTTTC
TTCCTCTCGTGCCCGAGTTTGTCGAGCAAGTTATTCGAGCAGAACGCCCCGGTGGTGTTTTGTTAACATTCGG
AGGACAAACTGGGTTAAATTGCGGTGTAGAATTACAAAAAGCTGGAATTTTTGATAAATACGATGTTAAAATA
CTCGGTACACCTATACAAGCTATAATCGATACGGAAGACAGGAAAATATTTAGCGAAAGAATTGCTTCTATAG
GTGAAAAAGTTGCTCCGAGTATGGCAGCTTATTCTGTGCAAGAAGCATTAGAAGCTGCGGAATTATTAGGATA
TCCTGTGATGGCTAGGGCGGCATTTTCTTTAGGAGGTTTAGGATCTGGTTTTGCAAAYAGTAGCGATGAACTC

AAATTACTTGCTCAACAAGCTTTAGCTCACTCAAATCAATTAATAATAGATAAATCGTTGAAAGGATGGAAAG
AAGTTGAGTATGAGGTTGTAAGAGATGATTAT???????????????GTTAATGATGAAGAATTACAAGAACC
TACTGATAAAAGAATGTTTGTGCTTGCTGCTGCTTTAAGAAACGGTTATAGTGTTGATAAACTTTATGATTT?
AACCAAAATAGATCGTTGGTTTTTACAGAAAATGAAGAACATCATTGATTATAACAATGTTCTTGAATCGTAT
CCTCAGAATAAATTTCAAG???GCACTAATATATATAAAGTTTACTAAAAGCTAAACAAATCGGGTTTAGCG
ATAAACAAATAGCTGTAGCTATTAAAAGTACAGAATTAGCTGTCAGAAAACAAAGACAAGATTTTGGTATTAT
TCCATATGTTAAACAAATAGATACTGTAGCTGCTGAATGGCCTGCGACAACGAATTATTTGTATTTAACGTAT
AATGCAAATAGCCATGATATAACTTTTAGTGATCAACACATAATGGTTATTGGATCAGGAGTTTATAGAATCG
GAAGTTCTGTTGAGTTTGATTGGTGTGCTGTTGGATGTTTGAGGGAATTAAGGAAATTAAA???????????
???
??TTCTCATATTATTA
GCCAGTCTAGAGGTAAAAATGAAACTTTTGGTACTTTAGGAATAATTTATGCAATAATAGCTATTGGATTATT
GGGATTTGTAGTTTGAGCTCATCATATATTTACTGTTGGAATAGATGTTGATACTCGGGCTTATTTTACTTCT
GCCACTATAATTATTGCAATTCCTACAGGAATTAAAATTTTTAGATGATTAGCAACTTTACATGGTACTCAAA
TAAAATTTACTCCTTCAATGTTATGGGCTTTAGGATTTGTTTTTTTATTTACTATTGGTGGATTAACAGGAGT
AATTTTAGCTAATTCTTCTATTGATATTATTTTACATGATACTTATTATGTAGTTGCTCATTTTCATTATGTA
TTATCGATAGGTGCAGTTTTTGCTATTATAGCAGGATTTGTTCAATGATTTCCATTATTTACGGGTTTAACAA
TAAATGAAACTATACTAAAAATTCAATTTTTTATTATATTTATAGGAGTAAATTTAACTTTTTTCCCTCAACA
TTTTTTAGGATTAGCAGGTATACCTCGACGTTATTCTGATTATCCTGATATTTATACCCCATGAAATGTAATT
TCTTCAATTGGAAGATTAATTTCTATAGTTAGAATTTTTTTATTACTATTTATTGTATGAGATAGATTTACAA
GAATACGTATAAATATTTCATCTTTAAACTTTAGAACATCCTTAGAATGATATCAATTATTTCCACCCGCTGA
ACATAG?????????????????????????AAGGAATATGTAGTAGTATTTGATTTTCTTGGT?AAGGATTC
GATCAGATATTATAATGAAGTACCTGTCGAAAAACGTGTCTTCAAAAATCTCCAATTGTTTTTGGAAAACAAA
GAGCCAGGAGACGATTTATTTGATAGATTAAATACAGCTGTGATGAACAAACATTTAAACGAACTAATGGAAG
GCTTAACCGCTAAGGTATTTCGTACTTATAACGCTTCTTGGACCCTACAACAACAACTTGACAAATTGACCAA
CGCAGACGATTCCATATCTGAAAAAATACTTTCATATAACCGAGCCAATAGGGCAGTAGCTATACTCTGTAAC
CATCAACGTGCTGTACCAAAAGGTCACCAAAAATCAATGGAAAAACTCAAAGAAAAAATCGACACTAAAAAAG
ATAATATCAAAGATGCCGAGAGGCAAGTTAAGGATGCACAGAGAGATGCGAAACA???CGGAAGTGTTAAAGA
GAAACAGATCTATGAAAAGAAAAAGAAAATGTTGGAGAGGCTAAGAGAGCAGCTAGCTAAATTGGAAATTCAG
GAGACCGACAGAGATGAAAATAAAACAATTGCACTCGGTACGTCAAAGTTGAACTATTTAGATCCGAGAATTT
CGGTCGCTTGGTGTAAGAAATTTGGTGTGCCGATTGAAAAAATTTATAACAAAACTCAA?????????????
?????????????????GAGATCACTTAAAGGACCGTTTCGATGGTGCATCAAGGGTGATGTTGAGTAATTCG
GCGAGTTCTAGGGG????????????AAACGC???GAATCGTC???CAAAACAAGATAAATTATCAAACAGTA
TAGCGTCTAATAGTATCCATAGTAAGAGAGAAAATCGGCCGAGAAAATATAAATACGGTTTTCAATTGAAACC
CTATAATCCGGATCATAAACCTCCGAGCCCTAAAGATTTGGTATATTTAGAACCATCGCCAGGTTTCTGCGAG
AAGAATCCTAAACTAGGTATTCAAGGTACTCACGGTAGATTG?TGCAAT????????????????????????
???
?????????????

Suniotrichus

GGGAAAAGCCCAGCACTGAATCCCGTGTCCGAACCGGGAAATGTAGTGTTTGGGAGGGTCCGCTATCCATCGT
ACGACGCGTCCAAGTCCTTCTTGAACGGGGCCACATACCCATAGAGGGTGCCAGGCCCGATAGCTGGAGGATC
TCTCCTCAGAGTCGGGTTGCTTGAGAGTGCAGCCCTAAGTGGGTGGTAAACTCCATCTAAGGCTAAATATGAC
CACGAGACCGATAGCGAACAAGTACCGTGAGGGAAAGTTGAAAAGAACTTTGAAGAGAGAGTTCAATAGTACG
TGAAACCGTTCAGGGGTAAACCTGAGAAACCCGAAAGGTCGAATGGGGAGATTCAGCGTGTCTCGTGTTCTGG
TCTGTGACGATGGTGTTCGCACCGGGCTGCGCCTTCCGGATCCGAAACCGGCGATGAACTCGTGCACTTCTCC
CCTAGTAGGACGTCGCGACCCGTTGGGTGCCGGTCTAAGGCCGACGGTGGAGCCTTGAAGTCCCGGCCGGCCC
GCTCGACGGTAAGACAGAGGCGTGGGGTCGCTACGTTAGCGTCCGGCCCGTCACAAGTTCGTTCGACTCGGAT
GTTGGACCTGTGTGCCGACCTCGAGCTCGCCGGCTGTTGGTGACGGTGTCCTCGGACAGGCTACACGCCGGTC
GGCGACGCTCTAGCTTTGGGTTTTCAGGACCCGTCTTGAAACACGGACCAAGGAGTCTAGCATGTGCGCGAGT
CATTGGGACCGCATCTAAACCTAAAGGCGAAATGAAAGTGAAGGCGTGCCGAGGGAGGATGGGTCGGGGGGCG
TCTCGTTCTCATCGCGAGATGAGGCGCACCCTAGGCGTACACGC????????????????????????????
?????????????????TACCATGGTGGATTCAAGAAGACCGACAAGCACCCCCCTAAGAACTGGGGTGACGT
AAACGTCTTCGCCAATCTCGACCCTGCCGGTGAATACGTTGTATCCACCCGCGTCCGCTGCGGCCGCTCCATG
GAGGGTTACCCATTCAACCCCTGCTTAACCGAAGAGCAATACAAGGAGATGGAGTCGAAAGTGTCCAGCACCT
TGTCCGGTCTCGAAGGCGAACTCAAGGGTACTTTCTACCCGTTGACCGGCATGGATAAGGATACTCAGCAGAA
GCTCATCGACGACCACTTCTTGTTCAAGGAGGGCGATCGCTTCCTCCAGGCTGCCAACGCCTGCCGCTTCTGG
CCGTCTGGACGTGGCATCTACCACAACGACAACAAAACATTCTTGGTCTGGTGCAACGAGGAGGACCATCTCC

GTCTCATCTCCATGCAAATGGGTGGCGATCTTGGCGAAGTCTACCGTCGTCTCGTGAACGCCGTCAACGACAT
CGAAAAGCGCGTTCCCTTCTCTCATAACGACAGATTAGGTTTCCTCACTTTCTGCCCATCCAACTTGGGCACA
ACTGTACGTGCCTCTGTACACATCAAAGTACCAAAGCTCGCATCCAACAAGGCCAAGCTCGACGAGGTCGCTG
CTAAATACAACTTGCAAGTACGTGGCACCCGCGGTGTTCCTACAAAAGTTTTAATTATTGGATCAGGGGGATT
ATCAATTGGACAAGCCGGTGAATTCGATTACTCCGGTTCGCAAGCTATCAAAGCGTTACAAGAAGAAAATATT
CAAACGGTGCTTATTAACCCCAACATTGCAACGGTACAAACATCGAAAGGTTTAGCTGATAAAATCTATTTTT
TACCTTTAGTTCCTGAATTTGTGGAACAAGTAATACGTGTTGAACGTCCCGGAGGTGTTTTATTAACATTTGG
AGGTCAGACGGGTTTAAATTGCGGTGTCGAATTACAAAAGGCTGGTATTTTCGAAAAATACGGTGTTAAAATT
TTGGGCACACCTATAGAAGCTATAATAGATACTGAAGACAGAAAGATTTTTAGCGAAAGAATTGCTATGATTG
GTGAAAAAGTTGCTCCAAGTATGGCTGCTCATTCTGTGCAAGAAGCTTTGGAAGCTGCTCATCTATTAGGATA
TCCGGTTATGGCGAGAGCTGCTTTTTCTTTAGGAGGTTTAGGTTCTGGTTTTGCAAATACAGCTGAAGAATTG
AAATTGCTTGCACAACAAGCTTTAGCACATTCGAATCAATTAATTATTGATAAGTCTTTAAGAGGATGGAAGG
AAGTTGAATACGAAGTTGTACGTGATGCTTTTCCATACATTAAGGAAGTTAATGATGATGAACTAGAAGAACC
TACAGATAAACGAATGTTTGTTCTTGCAGCAGCTTTAAGAAAAGGTTACAGTGTTGATAAACTTTACGACTT?
AACTAAGATAGATCGATGGTTCCTACAAAAAATGAAGAATATTATAGATTACAACACTCTTTTGGAGTCTGCT
CAACAATCTAAACAACAAATTTCGGCGAACACTTATAAACTGTTGCTAAAAGCGAAGCAAATCGGTTTCAGTG
ACAAACAAATTGCTGTCGCTGTTAAAAGCACTGAACTGGCAGTTAGAAAGCAACGACAAGATTTTGGTATCAT
TCCATATGTTAAACAAATAGATACTGTAGCAGCTGAATGGCCAGCCACTACCAATTATCTATACTTAACATAC
AATGCCGACAGCCATGACTTAACATTTACCGACCAACATACTATGGTTATCGGTTCAGGAGTTTACAGAATTG
GTAGTTCAGTTGAGTTCGATTGGTGTGCTGTGGGATGTTTAAGAGAGCTTAGAAAACTTAATAAGAAAACAAT
AATGGTTAATTACAATCCGGAAACTGTGAGTACAGATTATGATATGTCAGACCGGCTGTACTTTGAA??????
???CTCATATTATTT
CTCAAACAAGAGGAAAAAAGGAAACTTTTGGGACTTTAGGAATAATTTATGCAATAATAGCAATTGGGCTATT
AGGGTTTATTGTATGAGCACATCACATATTTACTGTAGGAATAGATGTGGATACTCGAGCATATTTTACTTCA
GCAACTATAATTATTGCCGTACCTACTGGAATCAAAATTTTTAGATGATTAGCTACATTACATGGAACTCAAA
TTAACTTTTCTCCTTCAATACTTTGATCTTTAGGGTTTGTTTTTCTATTTACAATTGGGGGATTAACAGGAGT
AATTTTAGCTAATTCATCTATTGATATTATTCTTCATGATACTTACTATGTAGTAGCACATTTCCATTATGTT
TTATCTATAGGAGCAGTATTCGCAATTATAGCAGGATTAATTCAATGGTTCCCTTTATTAACAGGCCTTACCA
TAAACGATTATTTACTAAAAATTCAATTTTTTTCTATATTTATTGGTGTTAATTTAACCTTTTTTCCTCAGCA
TTTTCTTGGTTTAGCAGGTATACCCCGTCGATACTCAGACTACCCTGATGCTTACACCCCTTGAAATATTGTG
TCTTCAATTGGGTCTGTAATTTCTTTAATTTCAATTTTTTTATTTATATTTATTATATGAGAAAGGTTTACTT
CAATGCGAATTAATTTATCAAGATTAAATTTAAATTCATCAATTGAATGAATACAACTTTATCCCCCTTCAGA
ACATAGATACTCAGAATTACCTATTTTATCGAAGGATTATGTGGTAGTATTCGACTTTCTCGGT?AAGGATTC
CATTAGATACTATAATGAAGTACCTGTAGAAAAACGTGTCTTCAAAAACCTCCAATTATTTATGGAAAACAAA
TCGCCGGGAGACGATTTGTTTGATAGATTAAACACAGCTGTGATGAACAAACATTTAAACGAGTTAATGGAAG
GTTTAACTGCCAAGGTGTTTCGTACTTATAACGCTTCTTGGACCTTACAACAGCAACTTGATAAATTGACCAA
TCCCGATGATTCCATATCAGAGAAAATTTTATCGTATAATCGTGCCAACAGAGCCGTGGCTATACTATGTAAC
CATCAACGTGCAGTACCAAAAGGTCATCAGAAATCGATGGAGAAGCTCAAAGAAAAAATAGAAACTAAACGGG
ATACTATCAAGGATGCCGAAAGGCAAGTCAAAGATGCACAAAGAGATGCAAAACA???CGGAAGTGTTAAGGA
GAAACAAATTTATGACAAAAAGAAGAAAACGCTAGAAAGACTAAGAGAACAGTTGGCAAAATTGGAAATTCAA
GAGACGGATCGTGATGAAAACAAGACAATTGCCCTTGGTACGTCCAAGTTGAATTATTTGGACCCTAGAATTT
CCGTTGCATGGTGCAAGAAGTTTGGTGTACCCATTGAAAAAATTTATAACAAAACTCAATGGATGCGACTGCC
GCCCTTCAGGATAATCGGTGATCACCTGAAGGATCGCTTCGATGGCGCTTCCAGAGTGATGCTGAGCAACTCG
GCCAGTTCGAGGGG????????????AAACGC???GAACCGTC???CGAAGCAGGACAAACTGTCGAACAACA
TCGCTTCGAACAGCATCCACAGCAAACGCGAGAACCGTCCTAGGAAATACAAGTACGGTTTCCAGTTGAAACC
CTACAATCCCGACCATAAGCCTCCGAGTCCCAAAGATCTGGTGTACTTGGAACCGTCGCCTGGGTTCTGCGAG
AAGAACCCGAAGCTGGGTATTCAGGGTACTCATGGTAGACTG?TGTAACGATACGTCGATTGGTGTTGATGGA
TGCGATTTGATGTGCTGCGGTAG?GGGTTACAGGACCCAGGAGGTGATCGTTGTTGAAAGGTGTAATTGCACT
TT???????????

Sunius

???
??AGGGTGCCAGGCCCGATCGCCGGAGGATC
TCTCCTCAGAGTCGGGTTGCTTGAGAGTGCAGCCCTAAGTGGGTGGTAAACTCCATCTAAGGCTAAATATGAC
CACGAGACCGATAGCGAACAAGTACCGTGAGGGAAAGTTGAAAAGAACTTTGAAGAGAGAGTTCAATAGTACG
TGAAACCGTTCAGGGGTAAACCTGAGAAACCCGAAAGGTCGAATGAGGAGATTCAGCGTGTCTCGTCTGCGGT
CGCGTGACGATGGTGCTTGCACCGGACCGCGCCTGCCGTAGACGTAGCCGGCGACGAACTCGTGCACTTCTCC
TCTAGTAGGACGTCGCGACCCGTTGGGCGCCGGTCTAAGGCCGACGGAGGAGCCTTGGGGTCCCGGCCGGCCC

GCTCGACGGTACGACAGAGGCGTGGGGTCGCTACGTTAGCGTCCGGCCCGTCACAAGTTCGGGCGACTCGGAC
GTCGGACCTGTGTGCCGACCCCGAGCTCGCCGGCTGATGGTGGCGGTGTCCTCGGACAGACTACACGCCGGTC
GGCGACGCTCTAGCTTTGGGTTTTCAGGACCCGTCTTGAAACACGGACCAAGGAGTCTAGCATGTACGCGAGT
CATTTGGACCGCATCTAAACCAAAAGGCAAAATGAAAGTGAAGGCGTGCCGAGGGAGGATGGGGCGGGGGGCG
TCTCGTTCTCATCGCGAGATGAGGCGCACCCA????????????GCTTACACCGTATTCGCTGACTTGTTCGA
?TCCCATTATTGAAGATTACCATGGTGGATTCAAGAAGACCGATAAGCACCCTCCCAAGAACTGGGGAGATGT
AAGCACCTTCGGCAATCTTGACCCAGCTGGTGAATATGTAGTCTCCACCCGCGTCCGTTGCGGCCGCTCCATG
GAAGGCTATCCATTCAACCCATGCTTAACCGAAGAACAATACAAGGAAATGGAAGGCAAAGTCTCTACCACTT
TGTCCGGCCTTGAAGCCGAACTCAAGGGTACTTTCTATCCTTTAACTGGAATGGACAAAGATACTCAACAAAA
GCTCATCGATGATCACTTCTTGTTCAAGGAAGGAGATCGTTTCCTCCAGGCTGCAAACGCTTGCCGTTTTTGG
CCATCTGGACGTGGTATTTACCATAACGACAACAAAACCTTCTTGGTATGGTGCAACGAAGAAGATCACCTTC
GTATCATCTCTATGCAAATGGGTGGTGATCTTGGCGAGGTCTACCGTCGCCTTGTAACCGCTGTCAACGAAAT
CGAGAAGCGTGTTCCCTTCTCCCACAATGACAGATTAGGTTTCTTGACCTTCTGCCCAACCAACTTGGGCACT
ACTGTACGTGCCTCTGTACACATTAAAGTACCTAAGCTCGCCGCCAACAAGGCCAAACTTGATGAAGTTGCTG
CCAAATACAACTTGCAAGTACGTGGTACTCGCGGTGTTCCTAAAAAAGTGTTAATAATTGGGTCWGGGGGATT
ATCCATTGGACAAGCAGGAGAATTCGATTATTCCGGATCACAAGCAATAAAAGCTTTACAAGAAGAAAACATC
CAAACTGTTCTAATTAATCCAAATATCGCAACAGTACAAACCTCGAAAGGTTTAGCTGATAAAATATACTTTT
TGCCATTAGTGCCTGAATTTGTGGAACAAGTAATTAGAGTAGAACGTCCTGGAGGCGTATTGTTAACATTTGG
TGGACAAACAGGGTTAAATTGTGGAGTAGAATTACAAAAAGCTGGAGTATTCGAAAAATACAATGTTCAAATT
TTGGGTACCCCAATAGAAGCTATAATAGATACTGAAGATAGAAAGATTTTCAGTGACAGAATTGCACTAATTG
GAGAAAAAGTTGCTCCTAGTATGGCTGCATATTCTGTACAGGAAGCTTTGGAAGCTGCAGATTTATTAGGGTA
CCCAGTTATGGCTCGAGCTGCATTTTCTTTRGGTGGTTTGGGGTCTGGCTTTGCTAATACTGCAGAAGAACTG
AAATTACTTGCTCAACAAGCTTTAGCTCATTCGAATCAGTTGATTATTGATAAGTCTTTAAAAGGTTGGAAGG
AAGTTGAATATGAAGTTGTCAAAGATGCATATCCTTACATAAAAGAAGTTAATGATGATGAATTAAAGGAACC
TACAGATAAAAGAATGTTTGTTCTTGCAGCAGCTTTAAGAAATGGTTACAGTGTTGATAAATTGTATGATTT?
AACAAAAATTGATCGTTGGTTCTTACAAAAAATGAAAAACATTATAGATTACAATTCACTCTTGGAATTAGTC
CAACAAAATAAATTACAAAGTTGCTCAAATATTTACAAGTTGCTATTGAAAGCGAAACAAATTGGTTTTAGTG
ATAAACAAATTGCTGTCGCTGTTAAAAGTACTGAACTTGCAATCCGAAAACAACGACAGGATTTAGGGATTAC
ACCGTTTGTTAAACAAATTGATACTGTAGCTGCTGAATGGCCTGCAACTACAAATTATTTATATTTAACGTAC
AACGCTGAAAGCCACGATATAACTTTTAATGATCAACACATAATGGTTATAGGTTCTGGTGTTTATAGAATTG
GAAGCTCTGTCGAGTTTGATTGGTGTGCTGTAGGTTGTTTGAGAGAACTTAGGAAATTAAATAAAAAGACAAT
AATGGTCAATTACAATCCAGAAACTGTTAGTACAGATTATGATATGTCCGATAGGTTGTATTTTGAGGAAATT
TCTTTTGAAGTTGTTATGGAT???CTCATATTATTA
GACAAGCTAGAGGTAAAAAGGAAACATTTGGGGCCTTAGGAATAATTTATGCAATAATAGCAATTGGACTATT
AGGATTTGTTGTTTGAGCTCATCATATATTTACAGTAGGAATAGATGTGGATACACGAGCTTATTTTACTTCA
GCAACTATAATTATTGCAGTTCCTACTGGAATCAAAATTTTTAGTTGATTAGCCACCCTCCATGGTACACAAA
TTAAATTTAATCCTTCAATATTATGATCTCTTGGATTTGTGTTTCTTTTTACAATTGGAGGATTAACAGGAGT
AATTTTAGCTAACTCATCAATTGATATTATTTTACATGATACATATTATGTAGTTGCCCATTTTCATTATGTA
TTATCAATAGGTGCAGTTTTTGCTATTATAGCAGGATTAATACAATGATTTCCTTTATTAACTGGTTTAGTAA
TAAATGAGTATCTTTTAAAAATTCAATTTTTTATTATATTTATTGGAGTTAATTTAACATTTTTCCCTCAACA
TTTTTTAGGATTAGCAGGAATACCTCGACGATACTCTGATTATCCAGATGCTTATACCCCATGAAATATTATT
TCTTCTATTGGATCATTAGTTTCCATAATAAGAATTTTTTTACTATTATTTATTATATGAGAAAGATTATCTT
CAATGCGAATAAATATCTCTGCTTTAAATTTTTCTTCATCAATTGAATGATTCCAATTATACCCACCAGCAGA
ACATAGCTATATTGAACTTCCAAT???????AAAGATTATGTGGTAGTGTTTGATTTCCTTGGT?AAGGATTC
CATTAGATATTATAACGAAGTACCTGTAGAGAAACGTGTCTTCAAAAACCTTCAATTGTTTATGGAAAACAAA
TCCCCCGGTGATGATTTGTTTGATAGATTAAATACTGCTGTGATGAACAAACATTTGAATGAGTTAATGGAAG
GTTTAACTGCAAAGGTGTTTCGTACTTATAACGCTTCGTGGACACTACAACAGCAACTCGATAAATTGACCAA
TCCAGATGATTCCATATCCGAAAAAATTTTATCATACAATCGTGCCAATAGAGCAGTAGCAATACTTTGTAAC
CATCAACGTGCTGTACCTAAAGGCCATCAAAAATCCATGGAAAAATTGAAAGAGAAAATTGATACTAAAAGAG
ATACTATTAAAGACGCTGAGAGGCAAGTTAAAGATGCACAAAAAGATGCCAAGCA???TGGGAGTGTTAAGGA
GAAGCAGATCTATGAGAAAAAGAAGAAAATGTTGGAGAGACTACGTGAGCAATTGGCTAAGTTGGAGATTCAA
GAGACCGACCGCGATGAAAATAAAACTATTGCCCTTGGCACGTCCAAGCTGAACTATTTGGATCCTAGAATCT
CGGTCGCTTGGTGTAAGAAGTTTGATGTGCCCATTGAAAAAATTTATAACAAAACTCAA???ATGCGTTTGCC
ACCCTTCAGAGTAATCGGTGACCATTTGAAAGATCGTTTCGACGGCGCTTCTAGAGTGATGTTGAGCAATTCG
GCTAGTTCAAGAGG????????????GAATGC???TAATCGTC???CAAAACAAGATAAACTTTCGAATAGTA
TAGCATCGAATAGTATACATAGTAAAAGAGAAAATAGGCCGAGGAAGTATAAATACGGTTTCCAATTGAAACC
TTACAATCCCGACCACAAACCTCCGAGTCCTAAAGATTTGGTGTATCTGGAACCGTCGCCGGGTTTCTGCGAG

AAAAACCCGAAGCTCGGGATACAAGGTACTCACGGTAGATTG?TGTAATGATACTTCTATTGGTGTGGATGGA
TGCGATTTGATGTGTTGCGGTAG?AGGATACAGGACCCAGGAAGTTATCGTCGTCGAAAGGTGCAACTGCACG
TT???????????

Tachyporus

????????????????????????????????????GCGAAGTGTAGTGTTTGGGAGGATCTATTAACCGTCGC
GGGGCGAGTCCAAGTCCTTCTTGAACGGGGCCACTTACCCATAGAGGGTGCCAGGCCCGACGTTGGAGAGGTC
TCTCCCTAGAGTCAGGTTGCTTGAGAGTGCAGCCCTAAGCAGGTGGTAAACTCCATCTAAGGCAAAATAACAC
CACGAGACCGATAGCAAACAAGTACCGTGAGGGAAAGTTGAAAAGAACTTTGAAGAGAGAGTTCAAAAGTACG
TGAAACCGTTCAGGGGTAAACCTGAGAAACCCGAGAGTCTGAAACGGGAGATTCATCGCGTCTYTRCGACAAA
CGCATGGTGACAGCGGCCCCGGCCAATCGCACCGCAGCTAGTCGTG??CGGCGAAGAATGTGTGCACTTCTCC
CAAAGTAGAACGTCGTGAGCCGTTGAGCTCTGGTCTACGGCCCGATGGGGAGCCC?CGGATCCCGGCCA??CC
GCCCGACGGCATAACAAAGGCACGGAGCCGCGAAGCACGCGTCCGGCCCGTCACAAGCGCGCTCGGCCCGGAA
TTCGGACCTA?GTGCCGTTTCTGGAGCTGACGGCTGCTGGTGGCGGCGCTCTCGGACCGTCTACATGCCTCTC
CACGACGCTTTAGCTAAGGGTTCTCAGGACCCGTCTTGAAACACGGACCAAGGAGTCTAGCATGTACGCGAGT
CATTGGGAAT??TATTAAACCCATTGGCGAAATGAATGTGAAGACGCTTTTAGGGAGGATGGGAATCGGGGCG
TCTCGTTCTCATTGCGAGTTGAGGCGCACCTAGAGCGTACACGCGCTTACACCGTCTTCGCTGAACTTTTCGA
?CCCTATCATTGAAGACTACCATACCGGTTTCAAGAAGACTGACAAGCACCCACCTAAGAACTGGGGTGATGT
ATCCACCTTTGGAAACGTTGACCCCACCGGCGAATACGTCGTGTCCACACGCGTCCGTTGCGGTCGCTCCATG
GAAGGATACCCGTTCAACCCATGCTTAACCGAARAACAGTACAAAGAAATGGAACAGAAAGTGTCCACCACCC
TCTCCGCCCTCGAGGGAGAACTTAAGGGTACCTTCTACCCATTGACTGGAATGAGCAAGGACGTTCAACAGAA
ACTCATCGACGACCACTTCTTGTTCAAGGAAGGTGACCGTTTCCTTCAAACCGCCAACGCTTGCCGTTACTGG
CCATCCGGACGTGGCATCTACCACAACGATAACAAAACCTTCTTGGTCTGGTGCAACGAAGAAGATYATCTTC
GTCTGATTTCCATGCAAATGGGTGGTGATCTTGGCGAAGTATACCGTCGTCTCGTAACAGCCGTCAACGACAT
TGAGAAGCGTGTGCCATTCTCCCACAACGACAGATTAGGTTTCCTCACCTTCTGCCCAACCAACTTGGGAACC
ACTGTACGTGCCTCCGTACACATCAAAGTACCTAAACTCGCCGCCAACAAGGCTAAGCTCGATGAAATTGCCG
GCAAGTACAACTTGCAAGTCCGCGGAACCCGCGGTGTTCCTAAGAAAGTTCTTATTATTGGGTCAGGAGGTTT
ATCWATAGGTCAAGCTGGAGAATTCGATTATTCAGGATCCCAAGCGATTAAAGCATTGCAGGAAGAGAATATT
CAAACGGTTTTGATAAATCCTAACATAGCAACAGTCCAAACATCTAAGGGCCTGGCCGATAAAGTCTACTTCT
TGCCTTTGGTGCCGGAATATGTTGAACAAGTTATTTGTGCTGAAAGGCCAGGTGGTGTTTTACTAACATTTGG
TGGGCAAACTGGGTTAAACTGTGGGGTAGCTTTACAAAAGGCTGGAGTTTTTGAAAAGTATGGAGTGAAGATA
TTGGGCACTCCAATACAGGCCATAATTGATACCGAAGACCGGCAAGTATTTAGTGAGAAAATAGCTAGTATTG
GTGAAAAAGTGGCACCCAGCATGGCTGCCTATTCTGTACAAGAAGCACTGGATGCGGCTGAGCAGTTGGGGTA
TCCAGTAATGGCAAGAGCAGCATTTTCATTGGGTGGTTTAGGATCTGGTTTTGCAAATAACAGTGATGAATTA
AAGTCATTATCACAACAAGCACTGGCTCATTCAACTCAATTGATAATCGATAAATCATTGAAAGGGTGGAAAG
AAGTTGAAT??
???
???
???
???
???
???
???
???
?????????????????????GAAGTATATATTTTAATTTTACCTGGATTTGGAATAATTTCCCATATTATTA
GACAAGAAAGAGGAAAAAAAGAAACTTTCGGAGCTTTAGGAATAATTTATGCTATAATAGCAATTGGAATTTT
AGGATTTGTTGTATGAGCTCACCATATATTTACAATTGGTATAGATGTAGATACCCGAGCTTACTTTACCTCA
GCAACTATAATTATTGCTGTCCCTACAGGAATTAAAATTTTCAGATGATTAGCTACACTTCATGGAACACAAA
TCAATTATTCCCCTTCCATATTATGAGCCCTAGGATTTGTATTTCTTTTTACAGTTGGAGGTTTAACTGGAGT
AATTTTAGCTAATTCCTCAATTGATATTATTTTACATGATACTTATTATGTAGTAGCCCATTTTCATTATGTT
TTATCTATAGGAGCAGTATTTGCTATTATAGCTGGTATTACACATTGATTTTCTTTATTTACAGGATTAACTT
TAAATGAAAAATTTCTAAAAATTCAATTTTTAACAATATTCATTGGAGTCAATTTAACATTTTTTCCTCAACA
TTTTTTAGGATTAGCTGGAATACCTCGACGATATTCAGATTTTCCTGATGCTTATTTAACTTGAAATATAATT
TCCTCAATTGGATCTTTAATTTCAATAATTAGAATTTTTTTTCTTTTATTTATTATTTGAGAAAGATTTATCT
CATCACGAAAAAGAATTTATCCATTAAATTTAATTTCATCAATTGAATGATTTCAATCACTTCCTCCCGCTGA
ACATAGTTATTCTGAACTTCCTTTAATCACT??????????????CGTCTTCGATTTTCTTGGT?AAGGATTC
TATTAGATATTATAATGAAGTACCTGTTGAGAAGCGTGTTTTTAAAAATTTACAACTATTCATGGAGAATAAG
GCTCCGGGTGATGATTTGTTTGATCGGTTAAACACTGCCGTTATGAATAAGCATTTAAACGAACTCATGGAAG

GCCTTACGGCTAAGGTTTTCCGTACCTACAATGCCTCATTTACATTACAACAGCAACTCGATAAATTAACCAA
CGAAGATGATTCTCTCTCTGAAAAAATTCTCTCCTACAATCGCGCGAATCGTGCGGTCGCAATCCTCTGTAAC
CATCAACGTGCAGTTCCAAAAGGACACCAAAAATCGATGGAAAAGCTCAAAGAAAAGATTGATTCCAAAAGGG
AGAACATTCATGATGCTGAGAGACAGGTTAAAGATGCTGAAAAGGCAGCTAAACG???GGGTAGTGTCAAGGA
AAAGCAGATCTATGACAAGAAAAAGAAACAGTTGCAGCGGCTCAAAGAGCAGCTGGCCAAGCTAGAGATTCAA
GAAACGGACCGTGATGAAAACAAAACTATCGCATTGGGCACGTCAAAATTGAACTATTTAGATCCCAGGATTT
CGGTGGCCTGGTGCAAAAAG???TGGATGCGGTTGCC
GCCGTTCCGCGTGATCGGCGACAACCTGAAGGACCGCTTCGACGGCGCCTCCCGCGTCATGGTCAGCAACGCC
GGCAACGCGCGCAACGC??????CAACAACGCGCACAACCGCC???CCAAACAGGACAAGCTCTCCAACAGCA
TCGCCTCCAACAGCATCCACAGCAAGCGCGAGAACAAACCGCGCAAATACAAGTACGGCTTCCAACTAAAACC
GTACAATCCCGATCACAAACCGCCGAGCGCGAAGGATTTGGTGTATTTGGAACCTTCGCCTGGTTTCTGCGAG
AGGAATCCGAAGTTGGGGATTCAGGGGACGCACGGGAGGCAG?TGTAATGAGACGTCGATAGGGGTGGATGGG
TGCGACCTCATGTGCTGCGGGAG?GGGTTACCGCACGCAGGAGGTCGTCGTCGTCGAGCGGTGCAACTGCACC
TTCC?????????

Tetartopeus

GGGAGAAGCCCAGCACTGAATCCCGTGGCCGAACCGGGAAATGTAGTGTTTGGGAGGGTCCGTCATCCATCGT
GCGACGCGTCCAAGTCCTTCTTGAACGGGGCCACATACCCATAGAGGGTGCCAGGCCCGATAGCTGGAGGACC
TCTCCTCAGAGTCGGGTTGCTTGAGAGTGCAGCCCTAAGTGGGTGGTAAACTCCATCTAAGGCTAAATATGAC
CACGAGACCGATAGCGAACAAGTACCGTGAGGGAAAGTTGAAAAGAACTTTGAAGAGAGAGTTCAATAGTACG
TGAAACCGTTCAGGGGTAAACCTGAGAAACCCGAAAGGTCGAATGGGGAGATTCAGCGTGTCTCGTCTTTGGT
GGCGTGACGATGGTGCGTGCACCGGGACGCGCCCTCCGAAGCCGCAACCCGCGGCGAACTCGTGCACTTCTCC
CCTAGTAGGACGTCGCGACCCGTTGGGCGCCGGTCTAAGGCCGAGGGTGGAGCCTTGGGGTCCCGGCCGGCCC
GCTCGACGGTAAGACAGAGGCGTGGGGTCGCGAAGTTCGCGTCCGGCCCGTCACAAGCGCGGGCGACTCGGAC
GTCGGACCTGTGTGCCGACCTCGAGCTCGCCGGCTGTTGGTGACGGTGTCCTCGGACAGACTACACGCCGGTC
GGCGACGCTTTAGCTTTGGGTTTTCAGGACCCGTCTTGAAACACGGACCAAGGAGTCTAGCATGTGCGCGAGT
CATTGGGACCGCATCTAAACCTAAAGGCGAAATGAAAGTGAAGGCGTGCCGAGGGAGGATGGGTCGGGGGGCG
TCTCGTTCTCATCGCGAGATGAGGCGCACCCAGAGCGTACACGCTCTTACACCACTTTCGCTGATTTGTTCGA
?CCCAATCATTGAAGACTATCATGGTGGATTCAAGAAGACCGACAAGCATCCCCCATCAAACTGGGGTGATGT
TAACACTTTCGTCAACCTTGACCCTGCTGGTGAATACGTTGTATCCACCCGTGTACGTTGCGGCCGTTCAATG
GAAGGTTATCCCTTCAACCCTTGCTTAACCGAGGATCAATACAAGGAGATGGAACAAAAAGTTTCAACCACTT
TGTCTGGACTTGAAGGCGAACTTAAGGGTACCTTCTACCCATTGACTGGAATGGATAAGGATACTCAACAAAA
ATTGATTGATGACCATTTCTTGTTCAAGGAAGGTGATCGTTTCCTTCAAACCGCTAACGCTTGCCGTTATTGG
CCATCTGGACGTGGTATCTACCACAACGATAACAAAACATTCTTGGTCTGGTGCAACGAAGAGGATCATCTCC
GTATCATCTCCATGCAAATGGGTGGTGATCTTGGTGAAGTATACCGTCGCCTTGTAACGGCCGTCAACGAAAT
TGAGAAGCGCATCCCATTCTCTCACAATGACAGATTAGGTTTCCTTACCTTCTGCCCAACCAACTTGGGTACA
ACTGTACGTGCCTCTGTACACATCAAAGTACCTAAGCTCGCTGCCAACAAGGCCAAGCTTGATGAAATCGCTG
GCAAGTACAACTTGCAAGTACGTGGTACCCGTGGTGTTCCAAAAAAAGTTTTAATAATTGGATCCGGTGGATT
ATCGATCGGTCAAGCTGGAGAATTCGATTATTCAGGTTCACAAGCAATTAAAGCYCTMCAAGAAGAAAATATA
CAAACAGTTTTAATTAATCCAAACATTGCAACGGTACAAACTTCCAAAGGCTTGGCCGATAAAGTTTATTTCC
TACCTTTAGTACCTGAATATGTAGAGCAAGTTATTCGGGTTGAAAGACCTGGAGGTGTATTATTAACATTTGG
CGGACAAACAGGGTTAAATTGTGGTGTTGCCCTTCAAAAAGCTGGAGTTTTYGAAAAATACGGTGTTAAAATT
TTGGGCACACCTATTCAAGCAATTATCGATACMGAAGATCGAAAAATTTTTAGTGATAGAATAGCATTAATTG
GAGAAAAAGTTGCACCAAGTATGGCTGCATATTCTGTACAAGAAGCTTTAGAAGCTGCCGAGTTATTAGGATA
CCCTGTAATGGCAAGAGCAGCATTTTCATTAGGAGGTTTAGGTTCTGGATTTGCAAATACCTCGGAGGAGTTA
AAATCTTTAGCGCAACAAGCACTTGCACATTCCAATCAATTAATTATTGATAAGTCTTTGAAAGGGTGGAAGG
AAGTTGAATATGAAGTTGTAAGGGATGCATATCACGATATCAAGCAAGTTAACGATGAAGAATTAAAGGAACC
TACAGATAAGAGAATGTTTGTAGTTGCAGCTGCATTAAAAAGTGGTTATAGCGTTGATAAATTATATGATTT?
AACGAAAATTGATCGATGGTTTTTGCAAAAAATGAAAAATATTATTGATCTTACGACTTTACTAGAATCGACA
GAACAAATAAAATTA???????????????ACAGCTAATATTTTATTAAAAGCAAAGCAAATTGGATTTAGTG
ATAAACAGATTGCTGCTGCTTGTAAAAGTACGGAACTTGCTATAAGAAAACAACGTCAAGATTTTAATATTAC
TCCGTATGTTAAGCAGATTGATACTGTCGCTGCTGAATGGCCGGCTACTACGAATTATCTATACTTAACTTAT
AATGCTGGAAGTCATGATTTAACCTTTGCTGAAGAGCATACAATGGTTATAGGTTCAGGAGTTTATAGAATTG
GTAGTTCTGTTGAATTTGATTGGTGTGCAGTTGGATGTTTACGTGAGTTAAGAAAATTAAATAAAAAAACAAT
CATGGTAAATTACAATCCGGAGACTGTAAGTACTGACTACGATATGTCAGATAGGTTGTACTTTGAAGAAATC
TCATTTGAAGTTGTTATGGATGAAGTTTATATTTTAATTTTACCAGGATTTGGAATAATTTCTCATATCATTA
GGCAAGCCAGAGGAAAAAAGGAAACTTTTGGAACTCTAGGTATAATTTATGCAATAATAGCAATTGGATTACT
AGGATTTGTAGTATGAGCCCATCATATATTTACTGTGGGTATAGATGTAGATACCCGAGCTTATTTTACATCA

GCAACAATGATTATTGCTGTACCAACTGGAATTAAAATTTTCAGATGATTAGCCACTTTACATGGAATCCAAC
TAAACTTTAACCCGCCTACTCTATGAGCTCTAGGATTTGTATTCTTATTTACTATTGGAGGATTAACTGGAGT
TATCCTAGCTAATTCTTCTATTGATATTATTTTACATGATACTTATTATGTAGTAGCTCATTTTCATTATGTT
CTGTCAATAGGAGCTGTATTTGCAATTATGGCAGGATTAGTTCAATGATTCCCCTTATTTACAGGACTTAGAC
TAAATGAAAAATTATTGAAAATTCAATTTTTTTCAATATTCATTGGAGTAAATTTAACCTTTTTTCCTCAGCA
TTTTCTTGGATTAGCCGGGATACCTCGACGATACTCTGATTATCCTGATGCCTATACAACCTGAAATGTAATT
TCATCAATTGGATCTTTAATTTCAACTATAAGTATTTTACTTTTATTATTTATTATTTGAGAAAGGTTTTCCT
CATCTCGTATAATTTTATCAACTAAAAATTTTTCTACTTCAATTGAATGATACCAATCCTATCCTCCCTCTGA
ACACAGATATAATGAACTACCTATGCTATCAAAGGATTACGTTGTGGTCTTTGATTTCCTCGGT?AAGGATTC
SATTAGATATTACAATGAAGTACCTGTGGAGAAACGTGTTTTCAAAAACCTCCAATTGTTCATGGAAAACAAG
GCGCCCGGCGATGATTTATTCGATAGGCTTAACACGGCTGTTATGAATAAGCATTTAAACGAGCTAATGGAAG
GTTTAACCGCCAAGGTATTTCGTACTTATAACGCCTCTTGGACTTTACAACAACAACTCGAGAAACTCACAAA
TGAAGACGATTCCATATCCGAGAAGATCTTATCGTATAACCGTGCCAATAGGGCGGTGGCTATCCTCTGTAAC
CATCAACGTGCCGTACCTAAAGGCCATCAGAAATCTATGGAGAAATTAAAGGAAAAAATTGAAACTAAAAAGG
AGAATATTAGAGATGCAGAACGGCAGGTAAAAGATGCGCAGAAGGACGCAAAGCA???TGGAAGCGTTAGGGA
GAAGCAGATCTACGATAAGAAGAAGAAAATGTTGGAGCGGCTTAGGGAGCAACTGGCGAAGCTCGAGATACAG
GAGACGGACCGCGACGAGAATAAGACAATTGCTCTCGGTACGTCCAAGCTGAATTATTTGGACCCGAGGATCT
CGGTTGCTTGGTGTAAGAAATTCGGTGTGCCCATCGAAAAA?????????????????????ATGCGGCTTCC
CCCGTTTCGCGTGATCGGTGACCACCTGAAGGACCGCTTCGACGGTGCCTCGCGTGTCATGCTCAGCAACTCA
GCGAGTTCCCGAGG????????????CAACGC???CAATCGTC???CCAAACAGGATAAGCTCAGCAACAACA
TAGCTTCCAACAGTATACACAGTAAAAGGGAGAATCGCCCAAGGAAATACAAGTATGGCTTCCAGCTTAAGCC
GTATAATCCTGACCATAAACCACCGAGCCCTAAAGATTTGGTGTATCTAGAGCCATCACCTGGTTTCTGCGAG
AAGAATCCGAAATTAGGGATACAAGGCACGCATGGAAGACAG?TGCAACGATACTTCAATAGGTGTTGATGGT
TGCGATTTAATGTGTTGTGGAAG?AGGTTACAGGACCCAGG????????????????????????????????
?????????????

Thinocharis

GGGAGAAGCCCAGCACTGAATCCCGTGGCCGAACCGGGAAATGTAGTGTTTGGGAGGGTCCGCCATCCATCGT
CCGACGCGTCCAAGTCCTTCTTGAACGGGGCCACATACCCACAGAGGGTGCCAGRCCCGATAGCCGGAGGATC
TCTCCTCAGAGTCGGGTTGCTTGAGAGTGCAGCCCTAAGTGGGTGGTAAACTCCATCTAAGGCTAAATATGAC
CACGAGACCGATAGCGAACAAGTACCKTGAGGGAAAGTTGAAAAGAACTTTGAAGAGAGAGTTCAATAGTACG
TGAAACCGTTCAGGGGTAAACCTGAGAAACCCGAAAGGTCGAATGGGGAGATTCAGCGTGTCTCGTTTCTGGT
CGCGTGACGGTGGTGCTCGCACCGTGCCGCGCCTTCCGGATCCGTAACCGGAGACGAACTCGTGCACTTCTCC
CCTAGTAGGACGTCGCGACCCGTTGGGTGCCGGTCTAAGGCAGGCGGTGGAGCCCCGGGG?CCCGGCCGGCCC
GCTCGACGGTAAGACAGAGGCGTGGGGTCGCTACGTTAGCGTCCGGCCCGTCACAAGTTCGGGCGCCTCGGAT
GTCGGACTTTTGTGCCGACCCCGAGCTCGCCGGCTGCTGGTGGCGGTGTCCTCGGACAGACTACACGCCGGTC
GGCGACGCTCTAGCTTTGGGTTTTCAGGACCCGTCTTGAAACACGGACCAAGGAGTCTAGCATGTGCGCGAGT
CATTGGGACCCGTACTAAACCTAAAGGCGAAATGAAAGTGAAGGCGTGCCGAGGGAGGATGGGTCGGGGGGCG
TCTCGTTCTCAACGCGAGGTGAGGCGCACCCAGAGCGTACACGC??????????????CGCTGATTTGTTCGA
?TCCCATCATTGAAGATTACCATACTGGCTTCAAGAAGAGCGATAAGCATCCCCCGAAGAACTGGGGTGATGT
AAACACCTTCGCCAATCTCGACCCAGCCGGTGAATATGTAGTCTCCACCCGCGTCCGTTGCGGCCGCTCCATG
GAAGGCTACCCCTTCAACCCGTGCTTAACCGAAGAGCAATACAAGGAGATGGAGACCAAAGTCGCCGGTACTC
TGTCCGGCATGGAAGGAGAACTCAAGGGTACATTCTACCCRTTGACCGGCATGGACAAGGCTACCCAGCAGAA
ACTCATCGACGACCACTTCCTGTTCAAGGAGGGTGACCGTTTCCTCCAGGCCGCGAATGCTTGCCGCTTCTGG
CCATCCGGACGTGGTATCTACCACAACGACAACAAAACCTTCTTGGTCTGGTGCAACGAAGAGGACCATCTCC
GCATCATCTCCATGCAGATGGGCGGCGATCTTGGCGAAGTCTACCGTCGCCTCGTGACCGCCGTCAACGAAAT
CGAGAAGCGCGTCCCGTTCTCCCACAATGACAGATTAGGTTTCCTCACCTTCTGCCCAACCAACTTGGGCACT
ACTGTACGTGCCTCTGTACACATCAAAGTACCTAAGCTCGCGGCCAACAAGGCCAAGCTCGATGAGGTCGCCG
GCAAGTACAACTTGCAAGTACGTGGTACCCGTGGC??????????????????????????????????????
?????????????GCTGGGGAGTTCGACTATTCTGGTTCGCAAGCAATTAAAGCGTTACAAGAAGAAAATATA
CAAACAGTACTAATTAATCCAAACATAGCTACTGTACAAACATCAAAAGGTTTAGCAGATAAAATTTACTTTT
TACCATTAGTGCCTGAATTTGTGGAACAAGTGATTAGAGTAGAACGTCCTGGCGGTGTTTTGTTAACATTTGG
CGGACAAACAGGGTTAAATTGTGGAGTGGAATTACAAAGAGCTGGTGTATTCGATAAATACGGTGTAAAAATT
TTGGGTACACCTATTCAAGCGATTATAGATACAGAGGATAGAAAAATTTTTAGTGAAAGAATATCATTAATCG
GAGAAAAAGTTGCTCCTAGTATGGCAGCATATTCAGTACAAGAAGCACTTGAAGCAGCTGATTTACTAGGCTA
TCCTGTCATGGCAAGAGCTGCTTTTTCTTTGGGTGGTTTAGGATCCGGTTTTGCGAATAACGATGAAGAACTT
AAATTACTATCTCAACAAGCTTTAGCACATTCC??
??????????????????????????????CCATACTTAAAAGAAGTAGACGATGACGAATTGAAAGAACC

TACTGATAAACGAATGTTTGTAATTGCGGCCGCTCTAAGAAATGGTTACAGCGTAGATAAGTTGTATGATTT?
AACAAAAATCGATCGCTGGTTCTTACAAAAAATGAAAAACATTATAGATTACAACACTCTTTTGGAATCAGTC
C??????ATAAGTTACAGAACTGTTCGAATACCTATAAACTTTTGTTGAAAGCGAAACAAATCGGTTTCAGTG
ATAAACAAATTGCTGTCGCAGTTAAAAGTACTGAACTTGCTATCAGAAAACAACGACAAGATTATGGTATAAC
TCCATATGTTAAACAAATTGATACTGTTGCCGCTGAATGGCCGGCTACTACAAATTATTTGTATTTAACGTAC
AATGCAGAAAGTCATGATTTAACTTTCAGTGATCAACACATAATGGTTATAGGATCTGGCGTTTACAGAATTG
GAAGTTCTGTTGAGTTTGATTGGTGTGCTGTTGGATGCTTAAGAGAACTTAGAAAGTTAAACAAAAAAACTAT
AATGGTCAATTATAATCCAGAAACTGTTAGTACAGATTATGATATGTCAGATAGATTATACTTTGAAGAAATT
TCATTTGAAGTTGTTATGGAT???TATTATTA
GACAAGCAAGAGGAAAAAAAGAAACATTTGGTTCTTTAGGAATAATTTATGCTATAATAGCAATTGGATTATT
AGGATTTGTTGTATGAGCTCACCATATATTTACAGTAGGAATAGATGTTGATACTCGAGCTTATTTTACTTCT
GCAACTATAATCATTGCAGTTCCTACTGGAATTAAAATTTTTAGATGATTAGCCACTCTTCATGGAACACAAA
TTAAATTTAATCCTCCAATACTTTGATCATTAGGGTTTGTTTTCTTATTCACAATTGGAGGACTTACTGGTGT
AATTCTAGCAAATTCATCAATTGATATTATTTTACATGACACTTATTATGTTGTTGCACATTTTCATTATGTT
CTATCCATAGGGGCGGTATTTGCAATTATAGCTGGATTAGTTCAATGATTCCCTCTTTTTACTGGATTAATAA
TAAATGAATATTTATTAAAAATTCAATTTTTCATTATATTTATTGGAGTAAATTTAACTTTTTTCCCTCAGCA
TTTTCTTGGACTAGCGGGTATACCTCGACGTTATTCTGATTATCCAGATGCTTACACTCCATGAAATATAATT
TCATCAATTGGCTCATTAATTTCAATAATTAGAATTTTTATTTTATTATTTATTTTATGAGAAAGATTTACAT
CTATTCGAATAAATATTTCATCAAAAAATTTTTCCACATCAATTGAATGATTAC???????????????????
???????????????????????????????AAAGATTATGTAGTAGTATTTGATTTCCTCGGT?AAAGATTC
CATTAGATATTACAATGAGGTACCTGTGGAAAAACGTGTCTTTAAAAACCTTCAGTTGTTTATGGAAAACAAA
TCACCAGGCGATGATTTATTTGATAGATTAAACACAGCTGTGATGAACAAACATTTAAATGAGTTAATGGAAG
GTTTAACTGCCAAGGTGTTTCGTACTTACAATGCTTCTTGGACTTTGCAACAACAACTCGACAAATTGACCAA
TCCAGATGATTCCATATCTGAGAAAATTTTATCATACAACCGTGCGAACAGAGCGGTAGCTATACTCTGTAAC
CATCAACGTGCTGTACCTAAAGGCCATCAGAAGTCCATGGAGAAACTTAAAGAGAAAATCGACGCTAAACGGG
ACACAATTAAAGACGCCGAGAGACAAGTTAAAGACGCCCAGAAAGATGCTAAACA???CGGAAGCGTCAAAGA
GAAGCAGATCTACGAGAAGAAGAAGAAGATGCTGGAGAGGATGAGGGAGCAACTGGCTAAATTGGAGATTCAG
GAAACGGACCGCGACGAGAACAAGACCATCGCCCTTGGCACGTCCAAGCTGAACTATTTGGACCCTAGAATCT
CGGTCGCCTGGTGCAAGAAGTTCGAGGTGCCCATTGAA??????????????????????TGGATGCGGCTGCC
GCCGTTCCGCGTGATCGGCGACCACCTGAAGGACCGCTTCGACGGCGCCTCGCGCGTCATGCTCAGCAACTCG
GCGAGCTCGCGCGG????????????CAACGC???GAACCGGC???CGAAGCAGGACAAGCTCTCGAACAGCA
TCGCCTCGAACAGCATCCACAGCAAGCGCGAGAACAGGCCGCGCAAGTACWAGTATTGGTTCCAGCTGAAGCC
GTACAACCCGGACCACAAGCCGCCGAGTCCCAGGGACCTTGTGTWCCTGGAGCCGTCGCCCGGTTTCTGTTAG
AAGAACCCGAAGCTCGGCATACAGGGCACGCACGGGAGGCTG?TGCAACGAGACTTCGATCGGCGTCGACGGG
TGCGACCTGATGTGCTGCGGCAG?GGGGTACAGGAGCCAGGAGGTGATCGTCGTCGAGCGGTGCAACTGCACT
TTCCA????????

Thyreocephalus

GGGAAAAGCCCAGCACCGAATCCCGCGGTCGTGCCGGGAAATGTGGTGTTAGGGAGGAT?CACTGTCCGTCGT
GCGGCGCGTCCAAGTCCACCTTGAACGGGGCCACTTACCCATAGAGGGTGCCAGGCCCGGCAACGGGAGGATC
TCTCCTCAGAGTCGGGTTGCTTGAGAGTGCAGCCCTAAGTGGGTGGTAAACTCCATCTAAGGCTAAATATAAC
CACGAGACCGATAGCGAACAAGTACCGTGAGGGAAAGTTGAAAAGAACTTTGAAGAGAGAGTTCAACAGTACG
TGAAACCGTTCAGGGGTAAACCTGAGAAACTCGAAAGATCGAATGGGGAGATTCAGCGCGTCTCGGTGGCGGT
GATGTGACGGTGACGTTCGCGTTGG?CCGCCTCGCC?GTTACCGCAGTT??TGACGAACGTGTGCACTTCTCC
CCTAGTAGAAGGTCGTGACCCGTTGGGTGCCCGTCTACGGCCCGCGGTGGAGACCGTGCGTCCTGGCCGGCCC
GCTCGACGGTATGAAGTTGGCGAGGGGCCGCGATTTTCGCGTCCGGCACGCGACAAGCACGGACGATCTGTCT
GTCGGACCTG?GTGCCGACGGCGGATCCGTCGGCTGCTGGTTGTGTTGTCCTCGGACAGACCATACGCCTGTC
AGCGACGCCTTTGCATTGGGTTCTCAGGACCCGTCTTGAAACACGGACCAAGGAGTCTAGCATGTGCGCGAGT
CATTGGGACT??AGCGAAACCTAAAGGCGAAATGAAAGCAAAGGCGTGCCGAGGGAGGATGCGGAGTGGGGCG
TCTCGAGCTCATCGCGAGCTGAGGCGCACCTAGAGCGTACACGC?????????????????????????????
???
?????????GGCAACCTCGACCCCACTGGCGAGTTCGTCGTGTCTACTCGTGTCCGTTGCGGCCGCTCCATG
GAGGGATATCCCTTCAACCCTTGCCTCACTGAGGAGCAGTACAAGGAGATGGAACAGAAGGTTTCCGGCACTC
TGTCCGGTCTCGAGGCAGAACTCAAGGGTACCTTCTACCCCCTCACCGGCATGAGCAAGGAAGTCCAGCAGAA
GTTGATCGACGATCACTTCTTGTTCAAGGAGGGTGACCGTTTCCTCCAGGCCGCCAACGCTTGCCGTTTCTGG
CCCAGCGGCCGTGGCATCTACCACAACGATAACAAGACCTTCTTGGTCTGGTGCAACGAGGAGGATCACCTCC
GCATCATTTCCATGCGGATGGGCGGCGACTTGGGTGAAGTTTACCGCCGTCTGGTCACAGCCGTAAACGAGAT
CGAGAAGCGCGTCCCCTTCAGCCACAACGACAGGCTCGGCTTCCTCACCTTCTGCCCGACCAACTTGGGCACC

ACCGTTCGTGCCTCCGTGCACATCAAGGTCCCCAAGCTCGCCGCCAACAA???????????????????????
???????????????????????????????????GTTCCTAGAAAAGTGTTAATCATTGGTTCTGGTGGACT
TTCCATTGGTCAAGCAGGAGAATTCGATTACTCAGGGTCACAGGCAATAAAAGCTTTACAAGAGGAACACATT
CAAACCGTCCTCATTAATCCAAACATTGCTACTGTACAAACATCGAAAGGTTTGGCAGATAAAGTATACTTTT
TACCTCTCGTTCCCGAATACGTGGAAGAAGTGATTAGATCTGAACGTCCAGGAGGAGTACTTCTCACATTTGG
TGGCCAAACAGGTTTAAACTGTGGGGTGGAATTAGAAAAAGCTGGTGTATTTGCCAAATACAATGTGAAGATC
TTAGGAACACCAATTCAAGCTATCATAGACACGGAAGATAGGAAAGTTTTTAGTGAAAGAATTTCTTCAATTG
ATGAAAAGGTAGCTCCCAGTTTGGCAGCTTATTCGGTTCAAGAAGCTTTGGATGCTGCTGAAAAATTAGGGTA
TCCGGTTATGGCACGTGCAGCTTTTTCACTAGGAGGCTTAGGTTCCGGTTTTGCTGACACTGCTGATGAATTG
AAATCTTTAGCCCAACAAGCCTTGGCACATTCAACTCAATTAATCATTGATAAATCACTAAAGGGTTG?????
????????????????????????????????CCGTATATAAAAAATGTAAACGATGAAGAATTGAAAGAACC
CACAGACAAAAGGATGTTTGTTGTGGCTGCAGCACTCAAGCACGGATATACTGTAGACCAATTATATAATTT?
AACTAAAATTGATAGATGGTTCCTGCAAAAAATGAAAAACATCATTGACTACTTCTCATATTTAGAATCGATT
GATT??????GCATA???????????????TCAAAAGGGGTTCTTTTAAAAGCGAAACAACTTGGCTTCAGTG
ATAAACAAATCGCAGCTGCTGTTAAAAGCACAGAATTGGCTATTAGGAAGCAAAGGAATGATTTAAACATCAC
ACCATATGTAAAACAAATTGATACTGTAGCTGCTGAATGGCCAGCTACAACTAACTACCTGTATTTAACATAC
AACGCCAATTCCCACGATCTTACTTTCAATGATGCGCATACTATGGTTATCGGATCTGGCGTTTACAGAATTG
GTAGTTCTGTGGAGTTTGATTGGTGCGCCGTTGGTTGTTTAAGAGAATTAAGGAAGTTGAATAAAAAGACCAT
AATGGTTAACTACA???
???????????????????????????????????????TTACCTGGATTTGGAATAATTTCTCATATTATTA
GACAAGAAAGAGGAAAAAAGGAAGCATTCGGAACTTTAGGAATAATTTATGCAATAATAGCAATTGGTCTTTT
AGGTTTTATTGTTTGAGCTCATCACATATTTACAGTTGGAATAGATGTTGATACTCGAGCTTACTTTACTTCA
GCAACTATAATTATTGCTGTACCAACAGGAATTAAAATTTTCAGATGATTAGCCACTCTACATGGAACACAAA
TTAATTATTCTCCCTCTATGCTTTGAGCTTTAGGGTTTGTATTTTTATTTACTGTTGGAGGACTGACAGGAGT
AATTCTAGCTAATTCATCAATTGATATTATTTTACATGATACCTACTATGTAGTAGCTCATTTTCATTATGTG
CTATCTATAGGAGCAGTATTTGCTATTATAGCAGGATTAGTACATTGATTTTCTCTATTTACTGGTTTAACTT
TAAACGAAAAATTCCTAAAAATTCAATTTTTAACTATATTTATTGGAGTAAATTTAACCTTTTTTCCCCAACA
TTTCTTAGGATTAGCTGGTATACCTCGACGATACTCTGATTATCCTGACGCTTACACTCCTTGAAATGTTCTT
TCTTCTATTGGTTCATTAATTTCATTAAGAAGAGTTTTCTTCCTTCTATTTATTATTTGAGAAGGATTTGTTG
CAAAACGAAAAAGAATTTCGGCTTTAAATTTACCTTCCTCAATTGAATGACTTCAAGAAATACCTCCTGCTGA
GCATAGCTAT?????????????????????AAGGATTATGTTGTCGTCTTCGACTTCCTAGGT?AAAGATTC
CATAAGGTACTACAATGAGGTGCCTGTGGAAAAGAGAGTATTCAAGAACCTCCAATTGTTCATGGAGAACAAA
TCGCCGGGTGATGATCTGTTTGATAGACTGAACACTGGCGTGATGAACAAGCATTTAAACGAGCTCATGGAAG
GGCTTACAGCGAAAGTGTTCCGTACGTATAATGCCTCATTCACACTGCAGCAGCAATTAGACAAGCTTACAAA
CCCCGATGATTCCCTCTCCGAGAAGATCTTGGCGTACAACCGGGCGAATCGCGCTGTTGCCATCCTCTGTAAC
CATCAGCGCGCCGTTCCCAAGGGCCACCAGAAGTCAATGGAGAAGCTAAAGGAGAAGATCGACTCAAAACGAG
ACGCAATATCAGATGCTGAGCGCCAAGTTTCGGATGCAAAACGCGATGCCAAGCA???CGGCAGCGTCAAAGA
AAAGGTTGTTTACGAGAAGAAGAAGAAAATGCTGGAGAGACTGCGTGAGCAGCTTAGCAAGCTCGAGATCCAA
GAGACAGACAGGGACGAGAACAAAACAATAGCGCTGGGCACGTCCAAGCTCAATTATCTGGACCCCCGTATCT
CGGTGGCCTGGTGCAAGAAGTACGATGTGCCCATCGAGAAAATCTACAACAAAACCCAG?????????TTACC
ACCATTCAGGGTCATCGGTGACAACCTCAAAGATCGTTTCGATGGCGCCACTAGGGTGATGCTCAGCAACACT
GCAAACTCCCGCAACCTAAACGGTCCAAACTC???CAATAGAC???CTAAACAAGACAAACTATCAAAYGCCA
TATCCTCCAATAGCATCCACAGTAAACGCGAGAACAAACCCCGAAAATACAAATACGGCTTCCAGCTCAAACC
TTACAATCCTGACCATAAACCTCCAAGCCCCAAAGACTTGGTGTATCTGGAACCATCGCCAGGCTTCTGCGAA
CGCAACCCCAAGCTAGGCATCCAAGGCACCAATGGTAGACAG?TGCGATGACACTTCAGTGGGAGTAGATGGC
TGCGATCTG??
?????????????

Acanthoglossa

???
???
???
???
???
???
???
???
???

??
??
??
??
??
??
??
??
??
??
??
??
??
??
??
??
??
??
??
??
??
??
??
??
??
??
??
??
??
??
??
??
??
??
??
??
??
??
??
??
??
??
??
??
??
??
??
??
??
??
??
??
??
??
??
??
??
??
??
??
??

??
?????????????

Diminudon

??
??
??
??
??
??
??
??
??
??
??
??
??
??
??
??
??
??
??
??
??
??
??
??
??
??
??
??
??
??
??
??
??
??
??
??
??
??
??
??
??
??
??
??
??
??
??
??
??
??
??
??
??
??
??
??

???
???
???
???
???
???
???
???
???
???
???
???
?????????????

Luzea

???
???
???
???
???
???
???
???
???
???
???
???
???
???
???
???
???
???
???
???
???
???
???
???
???
???
???
???
???
???
???
???
???
???
???
???
???
???
???
???
???

??
??
??
??
??
??
??
??
??
??
??
??
??
??
??
??
??
??
??
??
??
??
??
?????????????

Micrillus

??
??
??
??
??
??
??
??
??
??
??
??
??
??
??
??
??
??
??
??
??
??
??
??
??
??
??
??
??
??
??
??

???
???
???
???
???
???
???
???
???
???
???
???
???
???
???
???
???
???
???
???
???
???
???
???
???
???
???
???
???
???
???
???
???
???
?????????????
Midinudon_gen_nov
???
???
???
???
???
???
???
???
???
???
???
???
???
???
???
???
???
???
???
???
???

??
??
??
??
??
??
??
??
??
??
??
??
??
??
??
??
??
??
??
??
??
??
??
??
??
??
??
??
??
??
??
??
??
??
??
??
??
??
??
??
??
??
??
??
??
?????????????

Ophioomma

??
??
??
??
??
??
??
??
??
??

??
??
??
??
??
??
??
??
??
??
??
??
??
??
??
??
??
??
??
??
??
??
??
??
??
??
??
??
??
??
??
??
??
??
??
??
??
??
??
??
??
??
??
??
??
??
??
??
??
??
??
??
??
??
??
??
?????????????

Scymbalium
???
???
???
???
???
???
???
???
???
???
???
???
???
???
???
???
???
???
???
???
???
???
???
???
???
???
???
???
???
???
???
???
???
???
???
???
???
???
???
???
???
???
???
???
???
???
???
???
???
???
???
???
???
???
???
???

???
???
???
???
???
???
???
???
???
???
?????????????

Achenomorphus
0011110101010100003010001010000010101000001121000100000110011110110001001
1000200200000102001000111002110001010100000000001
Astenus
0011111000010200113010000110101010011000121111111101101110011100011211121
1000221100001020010000110121100011100110000000001
Astenus_USA
00111110000102001130100000?10101010011000121111111101101110011100011211121
100022110000102001000011012110001110?--000000001
Cylindroxystus
0010110100010100012010000010011000001000041120001200000110001010100001001
1102210200101011010101110021000000000110100000011
Dibelonetes
0011111000011200113010010110101010011000121111110101101100011110011211121
1002220000000102001000011012110001010011000000001
Diochus
00011001100001000030100010100000000100000110010001000120101100000000111000
01030011110001-----011010001100010110--000000000
Domene
0011110100000100021010001010000010010000101110001101100100001000100001001
1101200200101002011101111021010100000--000000011
Dysanabatium
0011110100000100021010001011000000101000101120101100000110010000100001001
1101200000000100311010010002100000010011000000001
Echiaster
00100201010112001130100101101011100110001211011112011002-
00111000112111211001201201001020010011110021100010100--000000001
Enallagium
0011110101010200021010000111000110011000001121111100000110011010110001001
10022002000010011101011010210001001110000000000?
Eustilicus
0011110101011200023010000111100110111000021101101101100100011010110000001
100210010000102011000111002110001010100000000001
Haplonazeris
0011110101010200013010011110101110111000120111101101100110011010111010121
0002210200001020010101110011100010100--000000?00
Hyperomma
0011110100010100022110100010000000001000100030001200000100011000000001011
1002100200101000010001010001000010100--000000110
Lathrobium
0011110100000100021010001010000010101000101120001300000100000000100001001
1101200200001002011101101021010100000--000000011
Leptobium
0010010100010100014111001010000000000000100030001210000100011000000001011
1002200200001003110101010011000010101--000000110

Lithocharis 00111101010100-
01130100000100001101010000011210001000001000110000000010011000200200000102
0010000111002110001010l--000001001
Lobrathium
00111101000001000210100010100000001010001011201011000001000110001000010001
1101200200101002011101101021010100000000000000011
Lordithon 01000201000000-
010010010101?01001010000110004000000010000110000000001100200130001001001-
----011010000-00010111000004000000
Medon
00100201010102000130100001101001100010000011210001000001000110001100010001
1102200200000102001000111002110001010011000000001
Medonina_Russia
00111101010001000130100011110001000010000011211001000001100110001100010001
1002100100001000110001111021100000101110000000001
Neolindus 00100201000100-
00120100200100110010010000411200012000001000010101000010111002210200101 01
101010110002l0?000010000010000011
Neosclerus
00100211010101000130100011100001001010000011100001000001000110101100010001
1002200200000102001000111002110001010100000000001
Notobium
00101101000001000011100010110000001010000011301011000001000010101000010001
1102200200101003110100010021000100001000000000000
Ochthephilum
10111101000101000221100000100000000010001000300012000001000010100000000000
0002100200001001010011010011000010100110000000111
Oedichirus
0011100100010100125201001010000000000000113001000110010 02-
000100000000001010031000000010031002011001110100101l0--011010010
Orus
00111201010102000130100311110001010010000011211011010001100110001100010001
1002200200001001010011110021100000101--000000001
Oxyporus
00100201000001111001000212010001100000111000400001001001000001000100110 02
1013020110001l-----011011000-00100000--000001001
Paederus
0010010100010100014111001110000000000000100020001101000 2-
00100010000000001100212120000100101010011011110001010011000000110
Pinophilus
00111001000101001252010010100000101000011000200001101000001110100000000010
1102100001001003110201110111010010111000000010001
Pseudolathra
00101101000001000110100000100000001010001011200012000001000000101000010001
1001200200101012110101100021000000001010000000011
Pseudomedon
00111201010101000130100001101001100010000011210001001001000110101100010001
1001200200000102001010111002110001010111000000001
Quedius
01001111000101010001001000100001101011101000400000101000011000010001110 02
0003000211000l-----101010000-0001011100100201022
Ronetus
0010020101011200113010010110101110011000121101111201100 2-
0011000011211121100320120100102001001111002110001010 0--000000001
Rugilus
00100101010112000230100101101001100110000210011001010001000110001100000001
1002101101001020010001110021100010100110000000001

```
      Sciocharis
0010000101011200023010010?1010001001100000111110010000010001100011000100 1
10021001000010 2??1000111002110000010100000000001
```

```
END;

BEGIN MRBAYES;
      set autoclose=yes nowarn=yes autoreplace=no;

charset 28S = 1-847;
charset ArgK_1 = 848-1568\3;
charset ArgK_2 = 849-1568\3;
charset ArgK_3 = 850-1568\3;
charset CADA_1 = 1569-2222\3;
charset CADA_2 = 1570-2222\3;
charset CADA_3 = 1571-2222\3;
charset CADC_1 = 2223-2868\3;
charset CADC_2 = 2224-2868\3;
charset CADC_3 = 2225-2868\3;
charset COI_1 = 2869-3681\3;
charset COI_2 = 2870-3681\3;
charset COI_3 = 2871-3681\3;
charset TP_1 = 3682-4366\3;
charset TP_2 = 3683-4366\3;
charset TP_3 = 3684-4366\3;
charset Wg_1 = 4367-4831\3;
charset Wg_2 = 4368-4831\3;
charset Wg_3 = 4369-4831\3;
charset morpho = 4832-4952;


      PARTITION partition = 5 : 28S, Wg_2 ArgK_2 Wg_1 CADC_2 CADA_1 TP_2
COI_1 ArgK_3 COI_2 CADC_3 CADA_2 TP_3, Wg_3 TP_1 ArgK_1, CADA_3 CADC_1
COI_3, morpho ;
      exclude COI_3;

      set PARTITION = partition;
      outgroup Oxyporus;
      set autoclose = yes;

      [general]
      prset applyto=(all) ratepr=variable;
      prset applyto = ( 1,2,3,4,5 ) ;

      [par 1 - GTR+I+G]
      prset applyto = (1) statefreqpr = dirichlet (1,1,1,1);
      lset applyto = (1) nst = 6 rates = invgamma;

      [par 2 - GTR+I+G]
      prset applyto = (2) statefreqpr = dirichlet (1,1,1,1);
      lset applyto = (2) nst = 6 rates = invgamma;

      [par3 - GTR+I+G]
      prset applyto = (3) statefreqpr = dirichlet (1,1,1,1);
      lset applyto = (3) nst = 6 rates = invgamma;

      [par 4 - GTR+I+G]
      prset applyto = (4) statefreqpr = dirichlet (1,1,1,1);
      lset applyto = (4) nst = 6 rates = invgamma;

      [par4 - GTR+G+I]
```

```
      lset applyto = (5) rates = gamma coding = variable;

      unlink shape = ( all ) statefreq = ( all ) revmat = ( all ) tratio
= (all) pinvar = (all);
      mcmc ngen = 8000000 printfreq = 1000 samplefreq = 1000 nchains = 4
nruns = 2 temp = 0.08 savebrlens = yes;
      sump;
      sumt;

END;

#Script for MrBayes v3.2.6 (Ronquist et al. 2012) for resolving the
phylogenetic position of Midinudon gen. nov. using morphological and
molecular data
#Cite: Tokareva A., Koszela K., Ferreira V.S., Yamamoto S., Żyła D. 2022.
The oldest case of paedomorphosis in Staphylinidae: a new genus of
Paederinae from Cretaceous amber
#Author: Katarzyna Koszela
```
